# ﻿Advances in Legume Systematics 14. Classification of Caesalpinioideae. Part 2: Higher-level classification

**DOI:** 10.3897/phytokeys.240.101716

**Published:** 2024-04-03

**Authors:** Anne Bruneau, Luciano Paganucci de Queiroz, Jens J. Ringelberg, Leonardo M. Borges, Roseli Lopes da Costa Bortoluzzi, Gillian K. Brown, Domingos B. O. S. Cardoso, Ruth P. Clark, Adilva de Souza Conceição, Matheus Martins Teixeira Cota, Else Demeulenaere, Rodrigo Duno de Stefano, John E. Ebinger, Julia Ferm, Andrés Fonseca-Cortés, Edeline Gagnon, Rosaura Grether, Ethiéne Guerra, Elspeth Haston, Patrick S. Herendeen, Héctor M. Hernández, Helen C. F. Hopkins, Isau Huamantupa-Chuquimaco, Colin E. Hughes, Stefanie M. Ickert-Bond, João Iganci, Erik J. M. Koenen, Gwilym P. Lewis, Haroldo Cavalcante de Lima, Alexandre Gibau de Lima, Melissa Luckow, Brigitte Marazzi, Bruce R. Maslin, Matías Morales, Marli Pires Morim, Daniel J. Murphy, Shawn A. O’Donnell, Filipe Gomes Oliveira, Ana Carla da Silva Oliveira, Juliana Gastaldello Rando, Pétala Gomes Ribeiro, Carolina Lima Ribeiro, Felipe da Silva Santos, David S. Seigler, Guilherme Sousa da Silva, Marcelo F. Simon, Marcos Vinícius Batista Soares, Vanessa Terra

**Affiliations:** 1 Institut de recherche en biologie végétale and Département de Sciences biologiques, Université de Montréal, 4101 Sherbrooke E., Montreal (QC) H1X 2B2, Canada Université de Montréal Montreal Canada; 2 Universidade Estadual de Feira de Santana, Departamento de Ciências Biológicas, Av. Transnordestina s/n, Campus, Novo Horizonte. 44036-900, Feira de Santana, BA, Brazil Universidade Estadual de Feira de Santana Feira de Santana Brazil; 3 Department of Systematic and Evolutionary Botany, University of Zurich, Zollikerstrasse 107, 8008 Zurich, Switzerland University of Zurich Zurich Switzerland; 4 School of Geosciences, University of Edinburgh, Old College, South Bridge, Edinburgh EH8 9YL, UK University of Edinburgh Edinburgh United Kingdom; 5 Universidade Federal de São Carlos, Departamento de Botânica, Rodovia Washington Luís, Km 235, 13565-905, São Carlos, SP, Brazil Universidade Federal de São Carlos São Carlos Brazil; 6 Programa de Pós-graduação em Produção Vegetal, Universidade do Estado de Santa Catarina, Centro de Ciências Agroveterinárias, Avenida Luiz de Camões 2090, 88520-000, Lages, Santa Catarina, Brazil Universidade do Estado de Santa Catarina Santa Catarina Brazil; 7 Queensland Herbarium and Biodiversity Science, Department of Environment and Science, Toowong, Queensland, 4066, Australia Queensland Herbarium and Biodiversity Science Toowong Australia; 8 Instituto de Pesquisas Jardim Botânico do Rio de Janeiro, Pacheco Leão 915, 22460-030, Rio de Janeiro, RJ, Brazil Instituto de Pesquisas Jardim Botânico do Rio de Janeiro Rio de Janeiro Brazil; 9 Programa de Pós-Graduação em Biodiversidade e Evolução (PPGBioEvo), Instituto de Biologia, Universidade Federal de Bahia (UFBA), Rua Barão de Jeremoabo, s.n., Ondina, 40170-115, Salvador, BA, Brazil Universidade Federal de Bahia Salvador Brazil; 10 Accelerated Taxonomy Department, Royal Botanic Gardens, Kew, Richmond, TW9 3AE, UK Royal Botanic Gardens Richmond United Kingdom; 11 Programa de Pós-graduação em Diversidade Vegetal, Universidade do Estado da Bahia, Herbário HUNEB, Campus VIII, Rua do Gangorra 503, 48608-240, Paulo Afonso, Bahia, Brazil Universidade do Estado da Bahia Bahia Brazil; 12 Center for Island Sustainability and Sea Grant, University of Guam, UOG Station, Mangilao, 96923, Guam University of Guam Mangilao Guam; 13 Centro de Investigación Científica de Yucatán, A.C. (CICY), Calle 43 No. 130 x 32 y 34, Chuburná de Hidalgo; CP 97205, Mérida, Yucatán, Mexico Centro de Investigación Científica de Yucatán, A.C. Mérida Mexico; 14 Eastern Illinois University, Charleston, IL 61920, USA Eastern Illinois University Charleston United States of America; 15 Department of Ecology, Environment and Plant Sciences, 10691, Stockholm University, Stockholm, Sweden Stockholm University Stockholm Sweden; 16 Department of Integrative Biology, University of Guelph, 50 Stone Road, Guelph (ON) N1G 2W1, Canada Royal Botanic Garden Edinburgh Edinburgh United Kingdom; 17 Chair of Phytopathology, Technical University Munich, 85354 Freising, Germany University of Guelph Guelph Canada; 18 Royal Botanic Garden Edinburgh, 20A Inverleith Row, Edinburgh, EH3 5LR, UK Technical University Munich Freising Germany; 19 Departamento de Biología, Universidad Autónoma Metropolitana-Iztapalapa, Apdo. Postal 55-535, 09340 Ciudad de México, Mexico Universidad Autónoma Metropolitana-Iztapalapa Ciudad de México Mexico; 20 Universidade Federal do Rio Grande do Sul, Programa de Pós-Graduação em Botânica, Av. Bento Gonçalves 9500, Bloco IV - Prédio 43433, Porto Alegre, RS, 91501-970, Brazil Universidade Federal do Rio Grande do Sul Porto Alegre Brazil; 21 Chicago Botanic Garden, 1000 Lake Cook Road, Glencoe, IL 60022, USA Chicago Botanic Garden Glencoe United States of America; 22 Departamento de Botánica, Instituto de Biología, Universidad Nacional Autónoma de México, Cd. Universitaria, 04510 Ciudad de México, Mexico Universidad Nacional Autónoma de México Ciudad de México Mexico; 23 Herbario Alwyn Gentry (HAG), Universidad Nacional Amazónica de Madre de Dios (UNAMAD), AV. Jorge Chávez N°1160, Madre de Dios, Peru Universidad Nacional Amazónica de Madre de Dios Madre de Dios Peru; 24 Department of Biology & Wildlife & Herbarium (ALA) at the University of Alaska Museum of the North, University of Alaska Fairbanks, P.O. Box 756960, Fairbanks AK 99775-6960, USA University of Alaska Fairbanks Fairbanks United States of America; 25 Programa de Pós-Graduação em Fisiologia Vegetal, Universidade Federal de Pelotas, Instituto de Biologia, Campus Universitário Capão do Leão, Passeio André Dreyfus, Departamento de Botânica, Prédio 21, Pelotas, Rio Grande do Sul, 96010-900, Brazil Universidade Federal de Pelotas Pelotas Brazil; 26 Evolutionary Biology & Ecology, Université Libre de Bruxelles, Faculté des Sciences, Campus du Solbosch - CP 160/12, Avenue F.D. Roosevelt, 50, 1050 Bruxelles, Belgium Université Libre de Bruxelles Bruxelles Belgium; 27 Instituto Nacional da Mata Atlântica / INMA-MCTI, Av. José Ruschi, 4, Centro, 29650-000, Santa Teresa, Espírito Santo, Brazil Instituto Nacional da Mata Atlântica Santa Teresa Brazil; 28 Department of Biological and Environmental Sciences, University of Gothenburg, Gothenburg, Sweden University of Gothenburg Gothenburg Sweden; 29 School of Integrative Plant Science, Plant Biology Section, Cornell University, 215 Garden Avenue, Roberts Hall 260, Ithaca, NY 14853, USA Cornell University Ithaca United States of America; 30 Natural History Museum of Canton Ticino, Viale C. Cattaneo 4, 6900 Lugano, Switzerland Natural History Museum of Canton Ticino Lugano Switzerland; 31 Western Australian Herbarium, Department of Biodiversity, Conservation and Attractions, Locked Bag 104, Bentley Delivery Centre, Western Australia, 6983, Australia Western Australian Herbarium Bentley Delivery Centre Australia; 32 Singapore Herbarium, 1 Cluny Road, Singapore, Singapore Singapore Herbarium Singapore Singapore; 33 Instituto de Recursos Biológicos, CIRN–CNIA, INTA. N. Repetto & Los Reseros s.n., Hurlingham, Buenos Aires, Argentina Instituto de Recursos Biológicos Buenos Aires Argentina; 34 Consejo Nacional de Investigaciones Científicas y Técnicas (CONICET), Godoy Cruz 2290 (C1425FQB), Ciudad Autónoma de Buenos Aires, Argentina Consejo Nacional de Investigaciones Científicas y Técnicas Ciudad Autónoma de Buenos Aires Argentina; 35 Royal Botanic Gardens Victoria, Melbourne, Victoria, 3004, Australia Royal Botanic Gardens Victoria Victoria Australia; 36 Geography and Environmental Sciences, Northumbria University, Ellison Place, Newcastle upon Tyne, NE1 8ST, UK Northumbria University Newcastle upon Tyne United Kingdom; 37 Programa de Pós-graduação em Ciências Ambientais, Universidade Federal do Oeste da Bahia, Rua Professor José Seabra Lemos 316, 47800-021, Barreiras, Bahia, Brazil Universidade Federal do Oeste da Bahia Barreiras Brazil; 38 Department of Plant Biology, University of Illinois, Urbana, IL 61801, USA University of Illinois Urbana United States of America; 39 Instituto de Biologia, Universidade Estadual de Campinas, Campinas, 13083-876, São Paulo/SP, Brazil Universidade Estadual de Campinas São Paulo Brazil; 40 Empresa Brasileira de Pesquisa Agropecuária (Embrapa) Recursos Genéticos e Biotecnologia, Parque Estação Biológica, Caixa Postal 02372, 70770-917, Brasília/DF, Brazil Empresa Brasileira de Pesquisa Agropecuária Brasília Brazil; 41 Instituto de Biologia, Universidade Federal de Santa Maria, 97105-900, Santa Maria/RS, Brazil Universidade Federal de Santa Maria Santa Maria Brazil

**Keywords:** Classification, diversity, Fabaceae, Leguminosae, Mimosoideae, phylogenomics, taxonomy

## Abstract

Caesalpinioideae is the second largest subfamily of legumes (Leguminosae) with ca. 4680 species and 163 genera. It is an ecologically and economically important group formed of mostly woody perennials that range from large canopy emergent trees to functionally herbaceous geoxyles, lianas and shrubs, and which has a global distribution, occurring on every continent except Antarctica. Following the recent re-circumscription of 15 Caesalpinioideae genera as presented in Advances in Legume Systematics 14, Part 1, and using as a basis a phylogenomic analysis of 997 nuclear gene sequences for 420 species and all but five of the genera currently recognised in the subfamily, we present a new higher-level classification for the subfamily. The new classification of Caesalpinioideae comprises eleven tribes, all of which are either new, reinstated or re-circumscribed at this rank: Caesalpinieae Rchb. (27 genera / ca. 223 species), Campsiandreae LPWG (2 / 5–22), Cassieae Bronn (7 / 695), Ceratonieae Rchb. (4 / 6), Dimorphandreae Benth. (4 / 35), Erythrophleeae LPWG (2 /13), Gleditsieae Nakai (3 / 20), Mimoseae Bronn (100 / ca. 3510), Pterogyneae LPWG (1 / 1), Schizolobieae Nakai (8 / 42–43), Sclerolobieae Benth. & Hook. f. (5 / ca. 113). Although many of these lineages have been recognised and named in the past, either as tribes or informal generic groups, their circumscriptions have varied widely and changed over the past decades, such that all the tribes described here differ in generic membership from those previously recognised. Importantly, the approximately 3500 species and 100 genera of the former subfamily Mimosoideae are now placed in the reinstated, but newly circumscribed, tribe Mimoseae. Because of the large size and ecological importance of the tribe, we also provide a clade-based classification system for Mimoseae that includes 17 named lower-level clades. Fourteen of the 100 Mimoseae genera remain unplaced in these lower-level clades: eight are resolved in two grades and six are phylogenetically isolated monogeneric lineages. In addition to the new classification, we provide a key to genera, morphological descriptions and notes for all 163 genera, all tribes, and all named clades. The diversity of growth forms, foliage, flowers and fruits are illustrated for all genera, and for each genus we also provide a distribution map, based on quality-controlled herbarium specimen localities. A glossary for specialised terms used in legume morphology is provided. This new phylogenetically based classification of Caesalpinioideae provides a solid system for communication and a framework for downstream analyses of biogeography, trait evolution and diversification, as well as for taxonomic revision of still understudied genera.

## ﻿Preface

Anne Bruneau^1^, Luciano Paganucci de Queiroz^2^, Jens J. Ringelberg^3,4^

In 1981, the first volumes of the Advances in Legume Systematics (ALS) series were published as Parts 1 and 2. Edited by Roger M. Polhill and Peter H. Raven, the intention was to disseminate new research results and information about the taxonomy, systematics and evolution of Leguminosae. It is a testimony to the success of this series, that more than four decades later, as part of the ALS series, we here present a 14^th^ volume, published in two parts, dedicated to the classification of subfamily Caesalpinioideae.

The first part of Advances in Legume Systematics 14 (Classification of Caesalpinioideae Part 1: new generic delimitations) was published in 2022 as a special issue of PhytoKeys (https://phytokeys.pensoft.net/issue/3247/) edited by Colin E. Hughes, Luciano P. de Queiroz and Gwilym P. Lewis. ALS14 Part 1 included 16 papers focused on generic delimitation in 15 clades of Caesalpinioideae based on new phylogenomic analyses presented there. Circumscriptions of 15 genera were revised to deal with issues of non-monophyly. In this second part of Advances in Legume Systematics 14, we present a higher-level classification of Caesalpinioideae that includes a fully updated synopsis of the 163 genera now recognised (ca. 4680 species) which are placed in 11 newly described or reinstated tribes, alongside a clade-based classification for the largest of these tribes, the recircumscribed Mimoseae.

As editors of Part 2 of Advances in Legume Systematics 14, we thank the 45 contributors from 14 countries for their diligence and collaborative spirit. This ambitious project that garners the expertise from colleagues around the world could only be possible with the willingness and hard work of all, several of whom contributed to revisions of texts in addition to their own. In particular, we thank Colin Hughes, Erik Koenen and Gwilym Lewis for discussions and ideas that initiated this project, and Colin Hughes and Gwilym Lewis for their important contributions to writing, revision, and editing of texts. We thank colleagues and organisations around the world who have shared images of Caesalpinioideae. We are indebted to the PhytoKeys editorial team for their support and careful reviews, and in particular Sandy Knapp, the handling editor, and two anonymous reviewers, as well as Fabian Michelangeli and Tiina Särkinen, for their comments that helped to improve this compendium. Finally we acknowledge financial support from institutions and funding sources that supported Open Access publication costs: Université de Montréal (Canada); Universidade Estadual de Feira de Santana (Brazil); Royal Botanic Gardens, Kew (UK); Royal Botanic Gardens, Edinburgh (UK); Chicago Botanic Garden (USA); Center for Island Sustainability and Sea Grant of the University of Guam; Universidad Nacional Autónoma de México; Technical University Munich (Germany); Royal Botanic Gardens Victoria, Melbourne (Australia).

## ﻿﻿Introduction - Classification of subfamily Caesalpinioideae

Anne Bruneau^1^, Luciano Paganucci de Queiroz^2^, Jens J. Ringelberg^3,4^, Gwilym P. Lewis^10^, Colin E. Hughes^3^

Citation: Bruneau A, Queiroz LP, Ringelberg JJ, Lewis GP, Hughes CE (2024) Introduction - Classification of subfamily Caesalpinioideae. In: Bruneau A, Queiroz LP, Ringelberg JJ (Eds) Advances in Legume Systematics 14. Classification of Caesalpinioideae. Part 2: Higher-level classification. PhytoKeys 240: 7–31. https://doi.org/10.3897/phytokeys.240.101716

With close to 800 genera and more than 22,000 species (LPWG 2023), the Leguminosae (Fabaceae) is the third largest angiosperm family in number of species after Asteraceae and Orchidaceae. Legumes include a large set of economically important food crops that provide highly nutritious sources of plant protein and micronutrients, which can greatly benefit health and livelihoods. They have been domesticated alongside grasses in different areas of the world since the beginnings of agriculture and have played a key role in its early development. Legumes are also important sources of fodder and green manure in both temperate and tropical regions, and are used for their wood, tannins, oils and resins, in the manufacture of varnishes, paints, dyes and medicines, and in the horticultural trade. Legume diversification probably started close to the Cretaceous-Paleogene boundary (ca. 66 Ma) (Koenen et al. 2020b, 2021), giving rise to one of the most spectacular examples of evolutionary diversification in plants. Modern legumes are exceptionally diverse, morphologically, physiologically and ecologically (Lewis et al. 2005; LPWG 2017).

In 2017, the Legume Phylogeny Working Group (LPWG 2017) revised the higher-level classification of the family and recognised six monophyletic subfamilies within the monophyletic Leguminosae. Under the LPWG classification, subfamily Caesalpinioideae DC. was re-circumscribed, and Cercidoideae LPWG, Detarioideae Burmeist., Dialioideae LPWG and Duparquetioideae LPWG (all of which were previously part of Caesalpinioideae sensu lato at different ranks) were recognised as distinct subfamilies along with an unchanged Papilionoideae DC. The former subfamily Mimosoideae DC., which is phylogenetically nested within Caesalpinioideae, was subsumed within the re-circumscribed Caesalpinioideae and has since been referred to as the mimosoid clade (LPWG 2017). The idea that Leguminosae comprises six main lineages, corresponding to these six subfamilies, is now widely accepted and has been confirmed by recent phylogenomic analyses of large nuclear gene and plastome DNA sequence datasets (Koenen et al. 2020b; Zhang et al. 2020; Zhao et al. 2021), which show robust support for all six subfamilies. Phylogenomic evidence suggests that the six subfamilies likely diverged very rapidly such that gene tree conflict obscures relationships among some of the subfamilies, but Papilionoideae is supported as sister to Caesalpinioideae (Koenen et al. 2020b, 2021).

Within subfamilies, new phylogenies of many legume groups have unequivocally demonstrated the non-monophyly of the tribes recognised in the classifications of Polhill and Raven (1981), Polhill (1994) and Lewis et al. (2005), and the need for new classifications (LPWG 2013, 2017). Following publication of the LPWG (2017) subfamily classification, phylogenetically-based tribal and clade-based higher-level classifications were developed for subfamily Detarioideae (Estrella et al. 2018) and informally for Cercidoideae (Sinou et al. 2020), but complete higher-level phylogenetically-based classifications are still lacking for Caesalpinioideae and Papilionoideae. Subfamily Duparquetioideae, comprising a single species, requires no classification, and Dialioideae, with just 18 genera, may not be easily amenable to, or in need of, additional higher-level subdivisions, although phylogenetic studies are ongoing (e.g., Falcão et al. 2023). For Papilionoideae, the largest of the subfamilies, despite ongoing progress in the understanding of phylogenetic relationships (Wojciechowski et al. 2004; Cardoso et al. 2012, 2013; Wojciechowski 2013; Zhao et al. 2021; Choi et al. 2022), more data and more complete taxon sampling are needed before a robust and stable phylogenetically-based classification system can be fully developed. For Caesalpinioideae, although some questions persist about the monophyly and placement of a small subset of genera and some on-going uncertainty surrounding generic delimitation and relationships (LPWG 2013, 2017; Koenen et al. 2020a), recent work has clarified most of these problems (Ringelberg et al. 2022), many of which were resolved in a series of papers in Advances in Legume Systematics 14, Part 1 (Hughes et al. 2022a).

A new higher-level classification of subfamily Caesalpinioideae is therefore now both feasible and timely. Here we use the phylogenomic backbones for subfamily Caesalpinioideae from Koenen et al. (2020a) and Ringelberg et al. (2022) as the basis for developing a new higher-level classification of the subfamily. This new phylogenetic classification provides a solid system for communication and a framework for downstream analyses of biogeography, trait evolution and diversification (e.g., Faria et al. 2022; Ringelberg et al. 2022, 2023), as well as for guiding efforts towards fully revising the taxonomy of still understudied genera.

### ﻿﻿Subfamily Caesalpinioideae: Diversity and distribution

**Figure 1.**
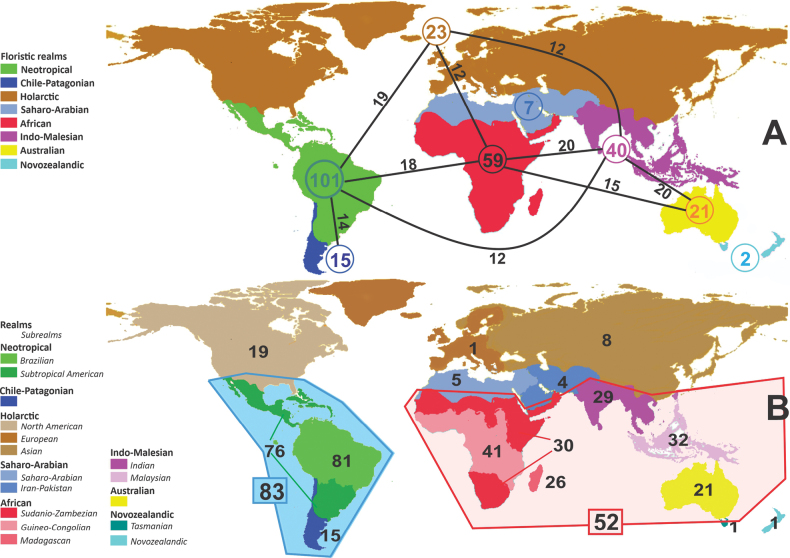
**A**Caesalpinioideae genus richness across floristic realms (according to Liu et al. 2023). The numbers within the circles represent the total number of genera in each realm. The numbers on the lines represent the number of genera shared between two realms (> 10 genera) **B** Number of Casalpinioideae genera in the floristic subrealms (sensu Liu et al. 2023). The numbers associated with the two polygons indicate the number of genera restricted to the two major blocks of tropical and subtropical areas in the New World and the Old World (maps modified from Liu et al. 2023, CC BY 4.0).

**Figure 2.**
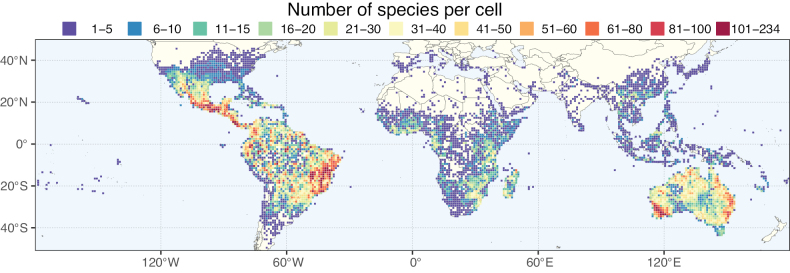
Map showing the global distribution of Caesalpinioideae species richness. Numbers of Caesalpinioideae species per one degree latitude / longitude grid cell. Infraspecific taxa are not counted individually but are included at the species level. All maps in this special issue are based on quality-controlled occurrence data from digitised herbarium specimens and floristic surveys (see Suppl. material 1 for details on occurrence data and methods used to generate maps).

**Figure 3.**
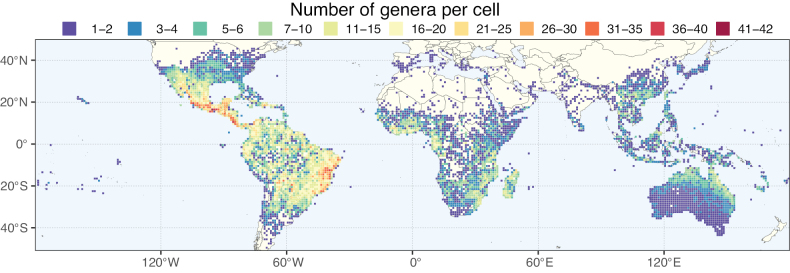
Map showing the global distribution of Caesalpinioideae genus richness. Numbers of Caesalpinioideae genera per one degree latitude / longitude grid cell.

Caesalpinioideae sensu LPWG (2017) is the second largest subfamily of legumes with ca. 4680 species placed in 163 genera (Hughes et al. 2022a; LPWG 2022; Ringelberg et al. 2022). Within this subfamily, ca. 3500 species and 100 genera are placed in the former subfamily Mimosoideae (the mimosoid clade of LPWG 2017), which we here recognise as the reinstated, but newly circumscribed, tribe Mimoseae, a tribal name first published by Bronn in 1822 (see below). Caesalpinioideae date to the late Paleocene when the subfamily is known from fossil bipinnate leaves from Colombia (Wing et al. 2009; Herrera et al. 2019). These fossils indicate that Caesalpinioideae were an abundant element in the earliest Neotropical rain forests in the Paleocene and time-calibrated legume phylogenies suggest that Caesalpinioideae started to diversify around 58 million years ago (Lavin et al. 2005; Bruneau et al. 2008; Koenen et al. 2021). Caesalpinioideae have thus diversified throughout the Cenozoic and now comprise diverse, abundant, and sometimes dominant elements across all major lowland tropical biomes, including rain forests, savannas and seasonally dry forests (Figs 1, 2, 3).

Caesalpinioideae are almost entirely woody perennials, but they are extremely diverse in stature and habit – including lianas, trees of all sizes, up to rain forest canopy emergents (e.g., *Cedrelinga* Ducke, *Dinizia* Ducke), shrubs, functionally herbaceous geoxyles, and two herbaceous aquatic species (*Neptunia* Lour.). Similarly, the subfamily is highly diverse in floral and fruit morphology. One of the hallmarks of Caesalpinioideae, as in many other plant groups, is repeated morphological and ecological convergences whereby similar leaf, flower and fruit morphologies, and ecological adaptations, have apparently been reinvented multiple times across lineages and through time (Ringelberg et al. 2022, 2023).

**Scheme 1.**
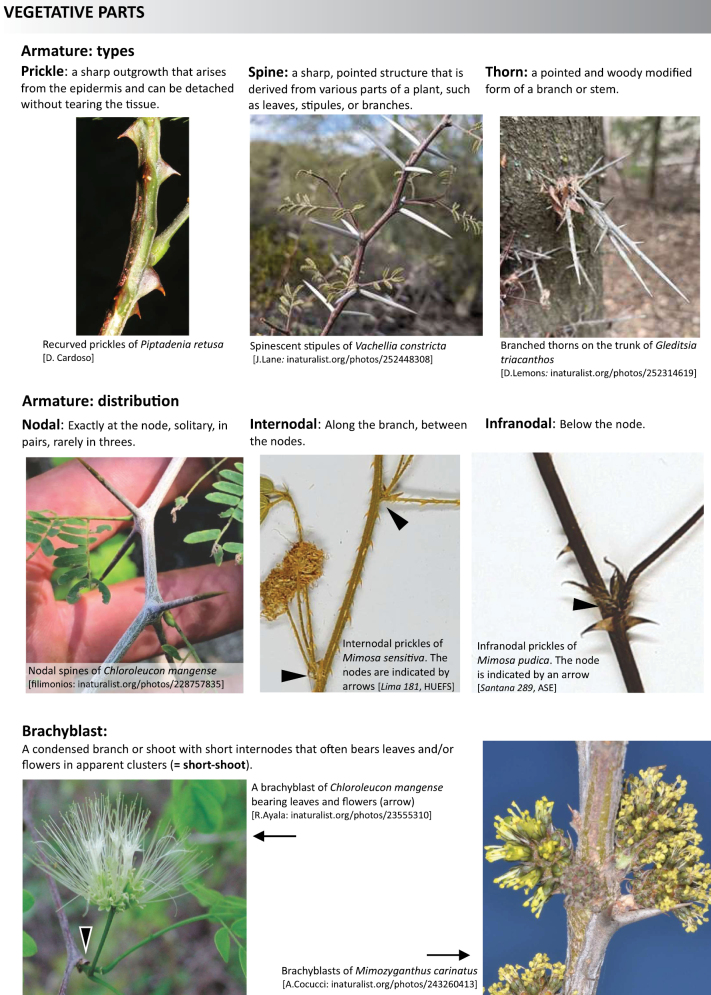


**Scheme 2.**
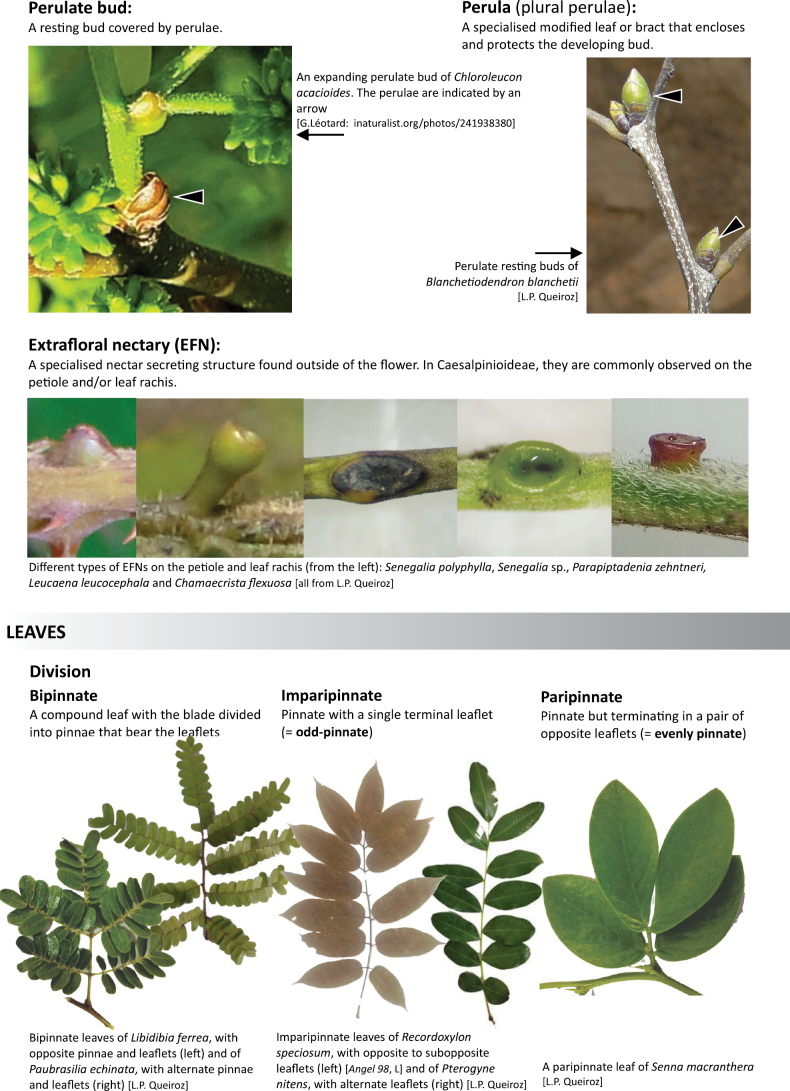


**Scheme 3.**
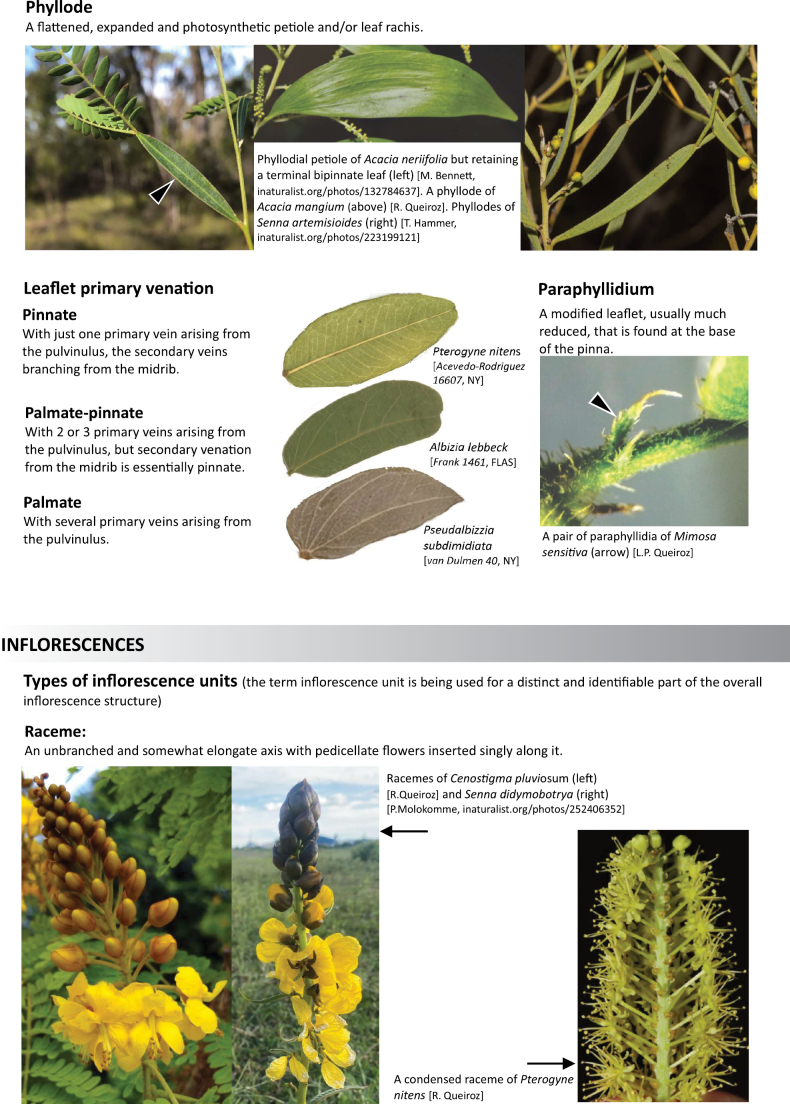


**Scheme 4.**
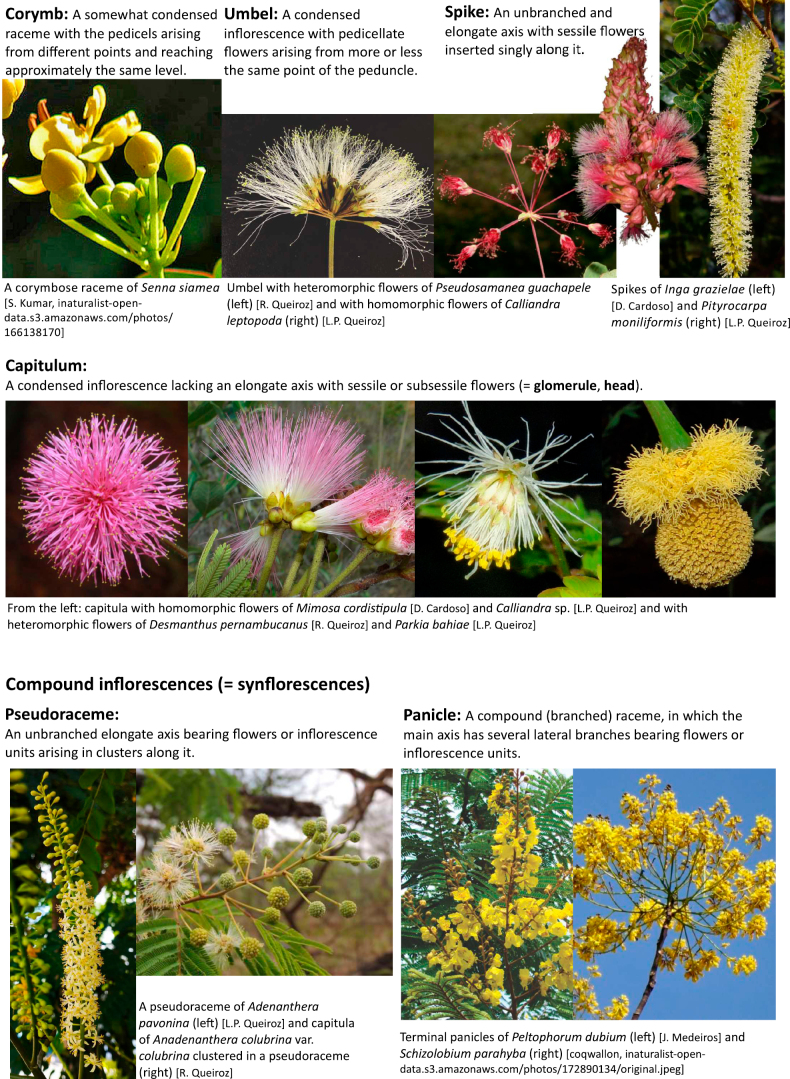


**Scheme 5.**
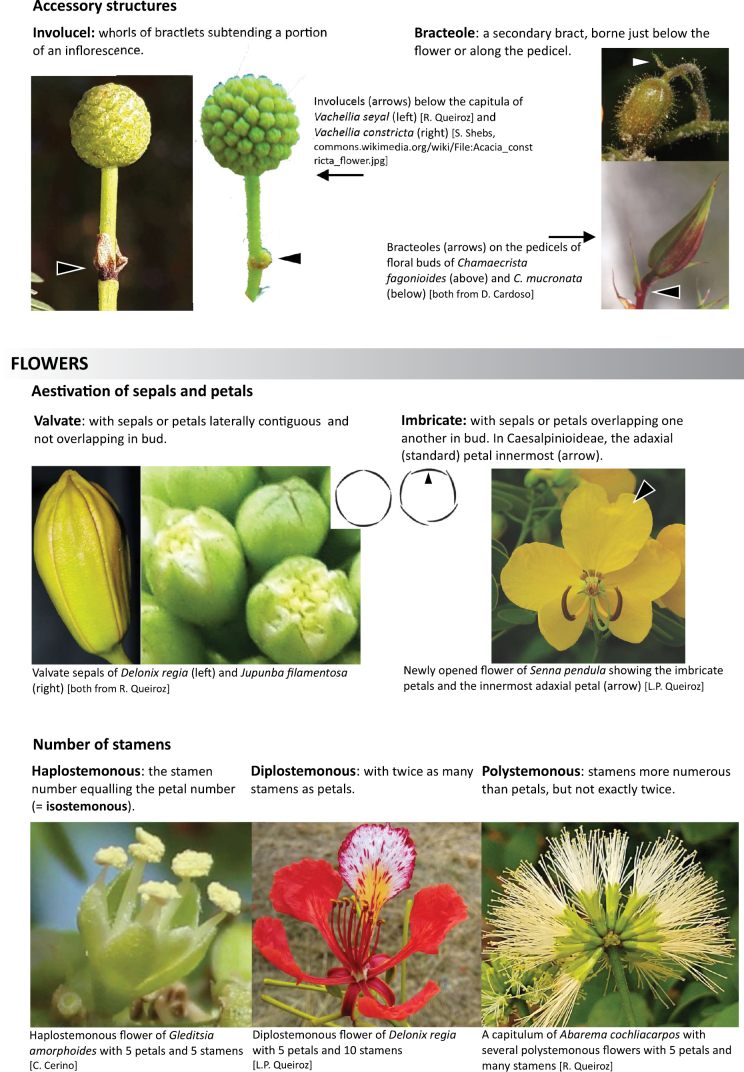


**Scheme 6.**
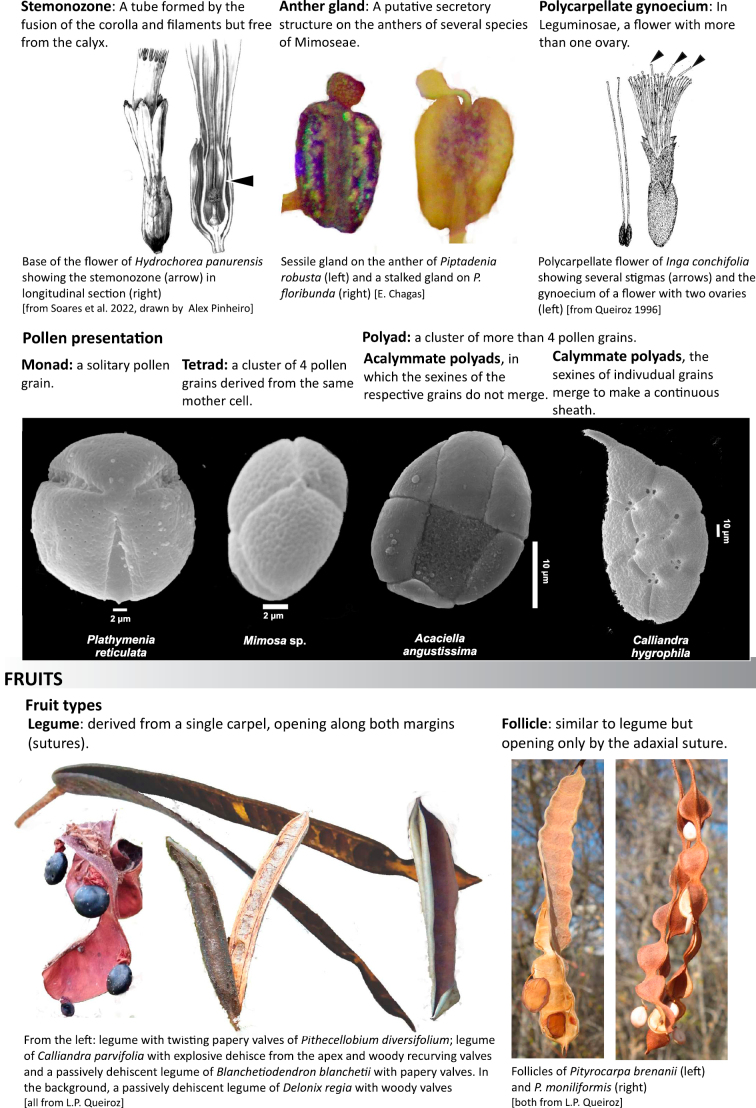


**Scheme 7.**
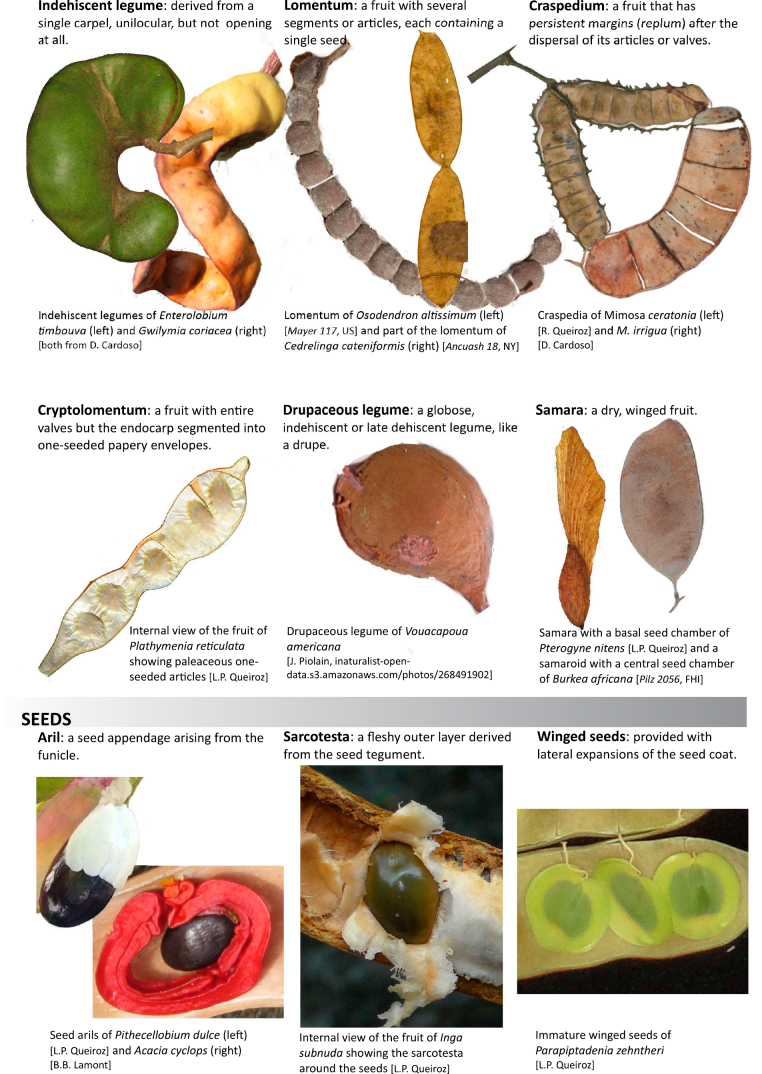


Caesalpinioideae is the only legume subfamily that has bipinnate leaves, which are prevalent but not universal across the subfamily (see Glossary, Schemes 1–7). A minority of genera have species with pinnate leaves, and leaves modified into phyllodes occur in most species of the large, mainly Australian, genus *Acacia* Mill. and in a few species in other unrelated genera including *Senna* Mill. and *Mimosa* L. The leaves themselves, especially the bipinnate leaf, can be extremely large (e.g., *Schizolobium* Vogel leaves are > 1 m long) to highly reduced; aphyllous, or nearly aphyllous, species occur in some genera [e.g., *Acacia*, *Senna*, *Chamaecrista* (L.) Moench, *Neltuma* Raf., *Prosopidastrum* Burkart]. Across all legumes, seismonasty, i.e., leaf movements prompted by touch, is known only within subfamily Caesalpinioideae, in the genera *Mimosa* and *Neptunia* (tribe Mimoseae). Extrafloral nectaries (EFNs) are present in the majority of Caesalpinioideae (Scheme 2), are morphologically extremely diverse, and often conspicuous and abundant on the petiole or leaf rachides between pinnae or leaflet pairs, and in a few genera (e.g., *Archidendron* F. Muell., *Macrosamanea* Britton & Rose) on floral bracts (Marazzi et al. 2019). A subset of Caesalpinioideae genera are armed with prickles, spines or thorns (Scheme 1), but armature is highly variable, has clearly evolved multiple times across the subfamily, and can vary within clades and even within genera (Hughes et al. 2022a; Ringelberg et al. 2022). Most genera of the mimosoid clade (tribe Mimoseae here) are confirmed nodulators, whereas just nine of the 63 non-mimosoid genera in the subfamily are currently known to nodulate. These nine genera are phylogenetically intermingled with confirmed non-nodulating genera, suggesting multiple evolutionary transitions between non-nodulating and nodulating lineages (Faria et al. 2022). Analyses of gene duplications have shown that several whole genome duplications (WGDs) occurred during the early evolution of the family Leguminosae (Cannon et al. 2015; Stai et al. 2019; Koenen et al. 2021; Zhao et al. 2021), although the exact number and placement of these WGDs remain uncertain. Within Caesalpinioideae, polyploidisation has also occurred numerous times during the Neogene in several genera across the subfamily [e.g., *Leucaena* Benth., *Dichrostachys* (A. DC.) Wight & Arn., *Neptunia*, *Vachellia* Wight & Arn., *Mimosa*; Dahmer et al. 2011; Govindarajulu et al. 2011a; Simon et al. 2011].

Across the subfamily, inflorescences and flowers are morphologically highly variable. The inflorescences can be racemose, paniculate or in fascicles and the Mimoseae have characteristic capitate or spicate and frequently heteromorphic inflorescences, often with some sterile flowers, some of which develop showy staminodia (Schemes 3, 4). Although usually bisexual, flowers can also be unisexual, and in the Mimoseae inflorescences can include a mixture of both bisexual and unisexual flowers with or without sterile flowers. The flowers are generally pentamerous, but there are many variations [3–6 (8) sepals or petals], and in some species, sepals and/or petals are absent (*Ceratonia* L.). Flowers are generally radially symmetrical in several Caesalpinioideae tribes, including the Mimoseae, but in other clades the flowers are bilaterally symmetrical or asymmetrical. Although a majority of Caesalpinioideae flowers are bee pollinated, specialised bat, bird, butterfly and moth pollinated flowers are also common (Arroyo 1981). In addition to having species with pollen in the more typical tricolporate monads, Caesalpinioideae is the only subfamily of legumes with taxa where pollen is arranged in polyads (Scheme 6). In Mimoseae the pollen arrangement is extremely variable across and sometimes within genera, with pollen in monads, tetrads, bi-tetrads and polyads. Fruit morphology is particularly homoplasious, and in the Mimoseae has proved misleading for generic delimitation (Borges et al. 2022; Ringelberg et al. 2022; Souza et al. 2022b). This diversity of fruit morphology (Schemes 6, 7) reflects adaptations to different seed dispersal syndromes, including passive, elastic and explosive dehiscence, as well as seed dispersal by water, wind, large herbivores, ants, and birds.

The subfamily is most diverse in lowland tropical and subtropical regions, only rarely occurring above 2500 m elevation, but a minority of genera have species in warm temperate zones that are not prone to severe frosts across the Americas, Europe, Asia, and Australia. More than half of Caesalpinioideae genera naturally occur in the Americas (104 of 163 genera), of which 84 are endemic. Africa (including Madagascar) has the second highest number of Caesalpinioideae genera, with 59 genera, 29 of which are endemic, followed by Asia (40 genera, 7 endemic), and Australia and the Pacific (27 genera, 6 endemic; See details in Tables 1, 2).

**Table 1.** Caesalpinioideae genera richness across global floristic realms and subrealms (according to Liu et al. 2023).

Floristic realms/subrealms	Present genera: n (%*)	Endemic genera: n (%**)
**Neotropical**	101 (61.96%)	71 (70.30%)
- Brazilian	81 (49.69%)	25 (30.86%)
- Subtropical American	76 (46.63%)	17 (22.37%)
**African**	59 (36.20%)	30 (50.85%)
- Madagascan	26 (15.95%)	6 (23.08%)
- Guineo-Congolian	41 (25.15%)	14 (34.15%)
- Sudanio-Zambezian	30 (18.40%)	5 (17.24%)
**Indo-Malesian**	40 (24.54% hi)	11 (27.50%)
- Malaysian	32 (19.63%)	5 (15.63%)
- Indian	29 (17.79%)	4 (14.29%)
**Holarctic**	23 (14.11%)	0 (0%)
- North American	19 (11.66%)	0 (0%)
- European	1 (0.61%)	0 (0%)
- Asian	8 (4.91%)	0 (0%)
**Australian**	21 (12.88%)	1 (4.76%)
**Chile-Patagonian**	15 (9.20%)	1 (6.67%)
**Saharo-Arabian**	7 (4.29%)	0 (0%)
- Iran-Pakistan	4 (2.45%)	0 (0%)
- Saharo-Arabian	5 (3.07%)	0 (0%)
**Novozealandic**	2 (1.23%)	0 (0%)
Novozealandic	1 (0.61%)	0 (0%)
Tasmanian	1 (0.61%)	0 (0%)

* Percentage of the total number of genera (= 163); ** Percentage of the number of genera present in the realm/subrealm.

**Table 2.** Distribution of the genera of Caesalpinioideae across different floristic realms and subrealms.

Genus	Realms	Saharo-Arabian		Holarctic			Chile-Patagonian	Neotropical		Indo-Malesian		African			Australian	Novozealandic	
	Sub-realms	Iran-Pakistan	Saharo-Arabian	North American	European	Asian		Brazilian	Subtropical American	Malaysian	Indian	Madagascan	Guineo-Congolian	Sudanio-Zambezian		Novozealandic	Tasmanian
	Total	4	5	19	1	8	15	81	76	32	29	26	41	30	21	1	1
	%	2.45	3.07	11.66	0.61	4.91	9.20	49.69	46.63	19.63	17.79	15.95	25.15	18.40	12.88	0.61	0.61
*Abarema*								##									
*Acacia*										+		+			+		+
*Acaciella*				+				+	+								
*Acrocarpus*										#	#						
*Adenanthera*										+	+	+			+		
*Adenopodia*									+				+	+			
*Afrocalliandra*														##			
*Alantsilodendron*												##					
*Albizia*		+								+	+	+	+	+	+		
*Amblygonocarpus*													#	#			
*Anadenanthera*								#	#								
*Anonychium*													##				
*Arapatiella*								##									
*Archidendron*										+	+				+		
*Archidendropsis*										##							
*Arcoa*									##								
*Arquita*							+	+	+								
*Aubrevillea*													##				
*Balsamocarpon*							#										
*Batesia*								##									
*Biancaea*						+				+							
*Blanchetiodendron*								##									
*Boliviadendron*								##									
*Burkea*													#	#			
*Bussea*												#	#				
*Caesalpinia*								#	#								
*Calliandra*				+			+	+	+								
*Calliandropsis*									##								
*Calpocalyx*													##				
*Campsiandra*								##									
*Cassia*								+	+	+	+	+	+	+	+		
*Cedrelinga*								##									
*Cenostigma*								#	#								
*Ceratonia*			+		+									+			
*Chamaecrista*				+		+	+	+	+	+	+	+	+	+	+		
*Chidlowia*													##				
*Chloroleucon*								#	#								
*Cojoba*								#	#								
*Colvillea*												##					
*Conzattia*									##								
*Cordeauxia*														+			
*Coulteria*								#	#								
*Cylicodiscus*													##				
*Delonix*												#	#	#			
*Denisophytum*				+				+	+			+		+			
*Desmanthus*				+				+	+								
*Dichrostachys*										+	+	+	+	+	+		
*Dimorphandra*								##									
*Dinizia*								##									
*Diptychandra*								##									
*Ebenopsis*									##								
*Entada*								+	+	+	+	+	+	+	+	+	
*Enterolobium*								#	#								
*Erythrophleum*										+	+		+	+	+		
*Erythrostemon*				+			+	+	+								
*Faidherbia*			+										+	+			
*Falcataria*										+					+		
*Fillaeopsis*													##				
*Gagnebina*												##					
*Gelrebia*													#	#			
*Gleditsia*				+		+			+	+							
*Gretheria*									##								
*Guilandina*						+		+	+	+	+	+		+	+		
*Gwilymia*								##									
*Gymnocladus*				+		+					+						
*Haematoxylum*								+	+					+			
*Havardia*									##								
*Heliodendron*															##		
*Hererolandia*														##			
*Hesperalbizia*									##								
*Heteroflorum*									##								
*Hoffmannseggia*				+			+	+	+								
*Hultholia*											##						
*Hydrochorea*								+	+				+				
*Indopiptadenia*											##						
*Inga*								#	#								
*Jacqueshuberia*								##									
*Jupunba*								#	#								
*Kanaloa*										##							
*Lachesiodendron*								#	#								
*Lemurodendron*												##					
*Leucaena*				+				+	+								
*Leucochloron*								##									
*Libidibia*								#	#								
*Lophocarpinia*									##								
*Lysiloma*									##								
*Macrosamanea*								##									
*Mariosousa*									##								
*Marlimorimia*								##									
*Melanoxylum*								##									
*Mezcala*									##								
*Mezoneuron*										+	+	+	+	+	+		
*Microlobius*								#	#								
*Mimosa*				+			+	+	+		+	+	+				
*Mimozyganthus*							+	+									
*Moldenhawera*								##									
*Mora*								#	#								
*Moullava*										+	+		+				
*Naiadendron*								##									
*Neltuma*				+			+	+	+								
*Neptunia*				+				+	+	+	+	+	+	+	+		
*Newtonia*													#	#			
*Osodendron*													##				
*Pachyelasma*													##				
*Painteria*									##								
*Parapiptadenia*								##									
*Pararchidendron*										+					+		
*Parasenegalia*							+	+	+								
*Paraserianthes*										+					+		
*Parkia*								+	+	+	+	+	+		+		
*Parkinsonia*				+			+	+	+				+	+			
*Paubrasilia*								##									
*Peltophorum*								+	+	+	+			+	+		
*Pentaclethra*								+	+				+				
*Piptadenia*								#	#								
*Piptadeniastrum*													##				
*Piptadeniopsis*								#	#								
*Pithecellobium*								#	#								
*Pityrocarpa*								#	#								
*Plathymenia*								##									
*Pomaria*				+					+					+			
*Prosopidastrum*									##								
*Prosopis*		+	+								+						
*Pseudalbizzia*								#	#								
*Pseudoprosopis*													##				
*Pseudosamanea*								#	#								
*Pseudosenegalia*								##									
*Pterogyne*								#	#								
*Pterolobium*						+				+	+		+	+			
*Punjuba*								#	#								
*Recordoxylon*								##									
*Ricoa*									##								
*Robrichia*								#	#								
*Samanea*								#	#								
*Sanjappa*											##						
*Schizolobium*								#	#								
*Schleinitzia*										##							
*Senegalia*		+	+	+			+	+	+	+	+	+	+	+	+		
*Senna*			+	+		+	+	+	+	+	+	+	+	+	+		
*Serianthes*									#	#							
*Sphinga*								#	#								
*Stachyothyrsus*													##				
*Stenodrepanum*							##										
*Strombocarpa*				+			+	+	+								
*Stryphnodendron*								#	#								
*Stuhlmannia*												#	#				
*Sympetalandra*										##							
*Tachigali*								#	#								
*Tara*								#	#								
*Tetrapleura*													##				
*Tetrapterocarpon*												##					
*Thailentadopsis*											##						
*Ticanto*						+				+	+				+		
*Umtiza*														##			
*Vachellia*		+		+			+	+	+	+	+	+	+	+	+		
*Viguieranthus*												##					
*Vouacapoua*								##									
*Wallaceodendron*										##							
*Xerocladia*														##			
*Xylia*											+	+	+				
*Zapoteca*								#	#								
*Zuccagnia*									##								
*Zygia*								#	#								

+ : present but not endemic; # : endemic to the realm; ## : endemic to the subrealm.

The Neotropical floristic realm (sensu Liu et al. 2023) has 101 Caesalpinioideae genera with native species, of which 71 are endemic to this realm (Table 1). Liu et al. (2023) divided the Neotropical realm into the tropical Brazilian subrealm (81 genera, 25 endemic) and the Subtropical American subrealm (76 genera, 17 endemic). The Subtropical American subrealm includes the northern (69 genera, 13 endemic) and southern (35 genera, 3 endemic) extremes of the Neotropical region (Fig. 1). The genera *Strombocarpa* (Benth.) Engelm. & A. Gray and *Prosopidastrum* Burkart are restricted to this subrealm and have an amphitropical distribution, whereas the genera *Erythrostemon* Klotzsch and *Desmanthus* Willd. have a similar distribution, but with a few species reaching the Brazilian subrealm. The second largest floristic realm for Caesalpinioideae genera is the African one, with 59 genera, of which 30 are endemic, primarily in the Guineo-Congolian subrealm (41 genera, 14 endemic). Within the Indo-Malesian realm, there are 40 genera (11 endemic), almost evenly distributed between the Indian (29 genera, 4 endemic) and the Malaysian subrealms (32 genera, 5 endemic).

Nine genera have a pantropical distribution, occurring in all tropical floristic realms [*Cassia* L., *Chamaecrista*, *Entada* Adans., *Guilandina* L., *Neptunia*, *Peltophorum* (Vogel) Benth., *Senegalia* Raf., *Senna*, *Vachellia*; Table 2]. The genera *Mimosa* and *Parkia* R. Br. occur in all tropical floristic realms except for the Australian realm. Seven genera (*Adenopodia* C. Presl, *Denisophytum* R. Vig., *Haematoxylum* L., *Hydrochorea* Barneby & J.W. Grimes, *Parkinsonia* L., *Pentaclethra* Benth., *Pomaria* Cav.) represent transatlantic disjunctions as they are exclusively distributed in the Neotropical and African realms.

The genera in Caesalpinioideae are geographically arranged across two major continental blocks. The American block, including the entire Neotropical realm, the Chile-Patagonian realm, and the southern part of the Holarctic realm (North American subrealm), has 83 genera restricted to it, with eight of these genera distributed across the Neotropical and Chile-Patagonian realms, some slightly extending to the southern portion of the North American subrealm (*Acaciella* Britton & Rose, *Calliandra* Benth., *Desmanthus*, *Erythrostemon*, *Hoffmannseggia* Cav., *Mimozyganthus* Burkart, *Neltuma*, and *Strombocarpa*). The second major block includes the African, Indo-Malesian, and Australian realms, with 52 genera restricted to this block and with *Adenanthera* L., *Dichrostachys*, *Erythrophleum* Afzel. ex R. Br., and *Mezoneuron* Desf. occurring in all three of these realms. *Acacia* weakly extends into the Madagascan (La Réunion and Mauritius) and Tasmanian subrealms.

Species richness, determined from occurrence records, indicate south-central Mexico and Central America, central-eastern Brazil, and southwestern Australia to be the most diverse regions (Fig. 2), with multiple one-degree grid cells in these regions containing more than a hundred Caesalpinioideae species. However, patterns of generic richness (Fig. 3) show that the high species diversity in Australia is largely attributable to the hyper-diverse genus *Acacia*, with over a thousand species. Australia is therefore characterised by high species but low generic richness. Important hotspots of Caesalpinioideae diversity also occur in continental Africa and Madagascar. Asia is the least diverse tropical continent, although it contains multiple species-rich lineages such as *Archidendron*, especially in South East Asia. The spatially biased availability of digitised occurrence data (Meyer et al. 2016) leads to an underestimation of Caesalpinioideae richness across large parts of tropical Africa, India, continental South East Asia, and the Amazon, as is apparent in Figs 2, 3. Nevertheless, our analyses (Figs 2, 3) represent an accurate depiction of relative differences in Caesalpinioideae continental richness patterns (Table 1).

The wide geographical distribution of Caesalpinioideae is matched by an equally wide ecological amplitude across the full precipitation spectrum of the tropics, spanning a 100-fold gradient in mean annual precipitation from arid deserts to seasonally dry tropical forests and savannas, and tropical rainforests (Schrire et al. 2005a; Gagnon et al. 2019; Ringelberg et al. 2023). Although Caesalpinioideae species are important components of many wet regions of the world, it is notable that some of the major hotspots of species and especially generic diversity coincide with areas dominated by seasonally dry vegetation in southern Mexico, north-eastern Brazil, and northern and south-western Madagascar, these being key areas of the succulent biome sensu Schrire et al. (2005a) and Ringelberg et al. (2020), plus seasonally dry subtropical south-western Australia. The subfamily thus has no obvious overriding wet or dry affinity, but rather has switched between wet and dry tropical biomes multiple times and has diversified substantially within each (Ringelberg et al. 2023). This ecological adaptability is undoubtedly, at least in part, a function of the evolutionary lability of life history strategies, including adaptations to fire, which has allowed Caesalpinioideae species to become important, diverse, and abundant elements of all lowland tropical biomes. It is also clear that Caesalpinioideae have been able to disperse across oceans numerous times to reach all tropical continents and the majority of lower latitude islands and island archipelagos (Figs 1, 2, 3). In contrast to this wide adaptability across tropical precipitation regimes and vegetation types, Caesalpinioideae show high tropical niche conservatism and very limited adaptability to cold temperatures and frost with just a small subset of lineages and species extending into temperate vegetation (Ringelberg et al. 2023).

### ﻿Reference phylogeny

Stability is one of the most important qualities of any taxonomic classification. It is therefore crucial that the phylogenetic framework used for assigning names to clades is robust and unlikely to change with sampling of additional taxa or genomic regions in the future. Based on the number and identity of the taxa included, the size of the genomic dataset, and the phylogenomic methods used to infer the phylogeny and assess its robustness, the phylogenetic framework employed here is currently the best available for taxonomic classification of Caesalpinioideae. This is confirmed by its overall agreement with previous smaller-scale phylogenies (see details below) and other recent independent phylogenomic studies (Zhang et al. 2020; Zhao et al. 2021). Furthermore, throughout this compendium the phylogeny is presented in such a way to allow easy assessment of underlying genomic support for all nodes subtending named clades, and in general clades named here are subtended by well-supported nodes on long branches. Absolute stability can never be guaranteed, and sampling of additional taxa might well result in different topologies and generic re-delimitation in some parts of the tree, such as in the Senegalia grade (Terra et al. 2022) or the Archidendron clade (Brown et al. 2022; Demeulenaere et al. 2022). Nevertheless, we consider the phylogenomic framework robust, and an adequate basis for the new classification presented here.

**Figure 4.**
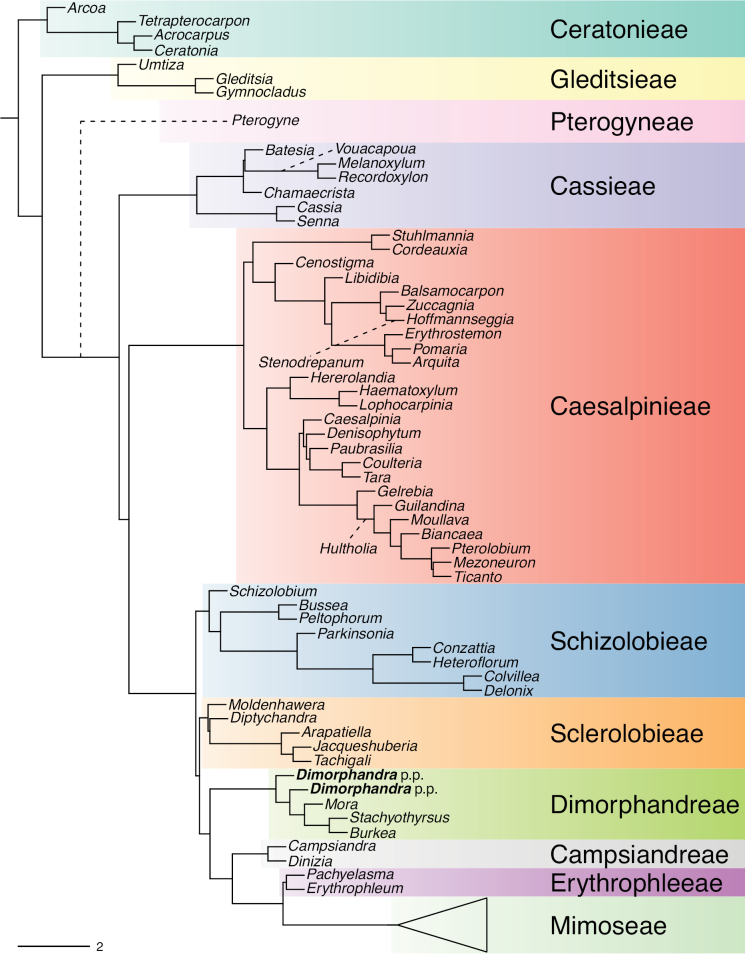
Phylogeny of Caesalpinioideae showing the tribal classification presented here. The names and phylogenetic placements of all 63 non-Mimoseae Caesalpinioideae genera are shown and known generic non-monophyly is indicated with terminal names of non-monophyletic genus in bold. The most likely placements for four unsampled genera are indicated with dashed lines; see respective treatments for details. Tribe Mimoseae has been collapsed (see Fig. 5). Branch lengths are expressed in coalescent units, and terminal branch lengths have been assigned an arbitrary uniform length for visual clarity. Monophyletic genera are represented by single branches; see Suppl. material 2 for a phylogeny with all accessions. See Suppl. material 3 for gene tree support across the phylogeny. The phylogeny is a pruned version of the backbone phylogeny of Ringelberg et al. (2023), where full details of the data and phylogenomic analysis methods are presented.

**Figure 5.**
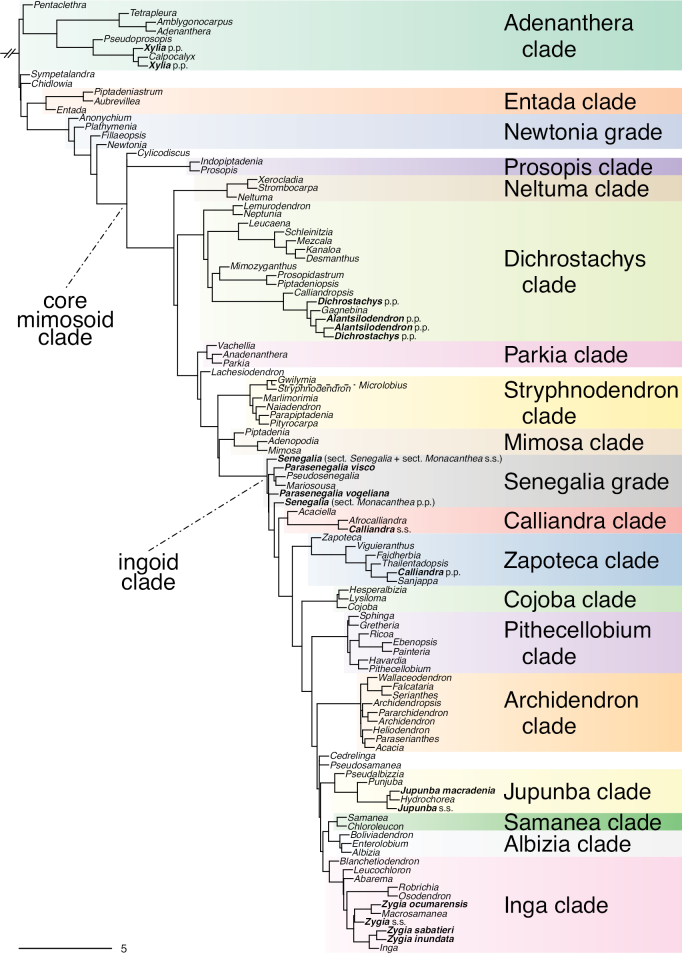
Phylogeny of tribe Mimoseae showing the clade-based classification of the tribe with two named higher-level and 17 named lower-level clades. The names and phylogenetic placements of all 100 Mimoseae genera are shown, and known generic non-monophyly is indicated with terminal names of non-monophyletic genera in bold. The most likely placement of the unsampled genus *Microlobius* is indicated with a dashed line; see Stryphnodendron clade treatment (page 319) for details. Branch lengths are expressed in coalescent units, and terminal branch lengths have been assigned an arbitrary uniform length for visual clarity. Monophyletic genera are represented by single branches; see Suppl. material 2 for a phylogeny with all accessions. See Suppl. material 3 for gene tree support across the phylogeny. The phylogeny is a pruned version of the backbone phylogeny of Ringelberg et al. (2023) where full details of the data and phylogenomic analysis methods are presented.

The classification proposed here uses as its framework the most comprehensively sampled phylogenetic analysis of Caesalpinioideae to date (Figs 4, 5, Suppl. materials 2, 3). This new phylogeny is based on Koenen et al. (2020a) and Ringelberg et al. (2022, 2023). By developing a clade-specific bait set for targeted enrichment of 964 nuclear genes, Koenen et al. (2020a) generated a DNA sequence dataset an order of magnitude larger than those used previously, thereby providing the greatly enhanced phylogenetic resolution required for classifying tribe Mimoseae. Capitalising on these foundations using a slightly modified version of the gene set covering 997 nuclear genes, and importantly extending the taxon sampling to include 300 additional species covering not only Mimoseae but also most genera of non-mimosoid Caesalpinioideae, as well as conducting transcontinental sampling of genera that occur across different continents, Ringelberg et al. (2022, 2023) established a robust phylogenomic hypothesis for subfamily Caesalpinioideae as a whole. These studies revealed or confirmed the non-monophyly of 22 genera, and this was the basis for the re-circumscription of 15 of these genera presented in Advances in Legume Systematics 14, Part 1 (Hughes et al. 2022a).

The phylogenomic analysis presented here includes 420 Caesalpinioideae species representing all but five of the 163 genera. The five missing genera are: *Vouacapoua* Aubl., which has three species and is likely a member of tribe Cassieae (e.g., Bruneau et al. 2008; LPWG 2017; Kates et al. 2024); *Pterogyne* Tul., placed here in a phylogenetically isolated, monospecific tribe (e.g., Bruneau et al. 2001, 2008; Manzanilla and Bruneau 2012; Zhang et al. 2020; Zhao et al. 2021); *Stenodrepanum* Harms and *Hultholia* Gagnon & G.P. Lewis, both monospecific genera of tribe Caesalpinieae (Gagnon et al. 2016); and *Microlobius* C. Presl, a monospecific genus in the Stryphnodendron clade of tribe Mimoseae (Lima et al. 2022). Although only about 10% of species were sampled in the analyses underlying the phylogenies presented here, several lower-level phylogenetic analyses of specific clades have been published and provide additional support for the groupings presented (Ringelberg et al. 2022). Furthermore, taxon sampling was specifically designed to cover taxonomic diversity spanning the root nodes of subclades and genera (Koenen et al. 2020a; Ringelberg et al. 2022, 2023).

A common feature of phylogenomic analyses employing large numbers of genes is the presence of conflict among gene trees, i.e., phylogenies based on individual genes (Salichos and Rokas 2013; Koenen et al. 2020a, 2020b, 2021; Zhang et al. 2020). Such gene tree conflict is widespread across many nodes in the Caesalpinioideae phylogeny presented here (Koenen et al. 2020a; Ringelberg et al. 2022, 2023). The main cause of this conflict appears to be lack of signal for many nodes in individual gene trees. In such cases, the relevant node in the species tree is only supported by a relatively small number of gene trees, but there is no strong support among the gene trees for any of the alternative, conflicting topologies. The presence of this type of gene tree conflict, indicative of lack of signal rather than true gene tree disagreement, does not preclude naming a clade subtended by such a node, as there is no strong reason to assume that including additional accessions or genomic regions would result in different relationships (Koenen et al. 2020a; Ringelberg et al. 2022). However, in a few places across the tree there is stronger support for alternative conflicting topologies (Koenen et al. 2020a; Ringelberg et al. 2022, 2023). In general, such instances of strong conflict are not found in nodes subtending clades named here, but rather in the relationships within clades [e.g., in parts of the Caesalpinieae (Fig. 34) and Archidendron clade (Fig. 225)] and between clades [e.g., the relationships between tribes Schizolobieae, Sclerolobieae, and Dimorphandreae (Suppl. material 3) or between the Adenanthera and Entada clades and *Sympetalandra* Stapf and *Chidlowia* Hoyle (Fig. 114)]. Similarly, strong gene tree conflict, including between the nuclear and chloroplast genomes, may affect generic delimitation in some parts of the tree, such as in *Senegalia* (Terra et al. 2022) and *Dimorphandra* Schott (Ringelberg et al. 2022). Where relevant for the new classification presented in this compendium, these instances of strong gene tree conflict are described below.

The reference phylogeny used here as the basis for the new classification was inferred using ASTRAL (Zhang et al. 2018), deploying the multi-species coalescent approach based on individual gene trees, which performs well on datasets with inter-genic conflict (Jiang et al. 2020). We always report the non-significant (i.e., > 0.05) outcomes of the ASTRAL polytomy test (Sayyari and Mirarab 2018), which tests for each node whether the polytomy null model can be rejected. Because conventional phylogenetic support metrics, such as bootstrap support, tend to be inflated in large phylogenomic datasets (Rokas and Carroll 2006), we report support for nodes in the species tree using measures of individual gene tree conflict and concordance, calculated using PhyParts (Smith et al. 2015) (Suppl. material 3). The impacts of phylogenomic methods and the presence of conflict among different gene and species trees on taxonomic decisions were further discussed in Ringelberg et al. (2022). Full details of the phylogenomic data and analyses were presented in Ringelberg et al. (2023).

### ﻿Integrating tribal and clade-based classifications

Under the LPWG (2017) subfamilial classification, subfamily Caesalpinioideae was the most difficult and controversial to delimit because of the inclusion of the formerly recognised, widely accepted and morphologically distinctive subfamily Mimosoideae. Abandoning the well-known Mimosoideae, an important disadvantage of adopting the six-subfamily classification, was mitigated by continuing to recognise this lineage as a named clade, informally referred to simply as the mimosoid clade until now (LPWG 2017), but here formally reinstated as the re-circumscribed and expanded tribe Mimoseae within the new Linnean tribal classification proposed here.

Although Mimoseae have traditionally been diagnosed by a series of diagnostic features, notably valvate petal aestivation and flowers with a reduced perianth and showy androecium, mostly clustered in compact inflorescences, the morphological distinctions between the mimosoid clade and some genera of the subtending grade of caesalpinioid lineages are not always clear-cut. For example, *Dinizia*, once considered to be in Mimosoideae, is placed outside the mimosoid clade in molecular phylogenetic and phylogenomic analyses (Luckow et al. 2003; Bruneau et al. 2008; Ringelberg et al. 2022). Conversely, *Chidlowia*, which has always been considered a non-mimosoid caesalpinioid legume (Polhill and Vidal 1981; Lewis 2005b), is placed within the mimosoid clade in all molecular phylogenetic analyses (Manzanilla and Bruneau 2012; LPWG 2017; Koenen et al. 2020a; Ringelberg et al. 2022). However, the long branch subtending what could be considered equivalent to the old subfamily Mimosoideae (plus or minus these few genera) (Fig. 4), together with the strong phylogenetic support for the clade, provide ample justification for recognising it at the tribal level.

The new classification proposed here thus follows a traditional Linnean approach but is complemented by a clade-based classification of the large tribe Mimoseae. Rank-free naming of clades within subfamilies and tribes has been prevalent in the legume literature, with many examples of clade names that have become widely used and accepted, such as the dalbergioid clade (Lavin et al. 2001) and the inverted repeat [IR]-lacking clade (Wojciechowski et al. 2000) of Papilionoideae; the Umtiza clade (Herendeen et al. 2003b) and the ingoid clade (Koenen et al. 2020a) of Caesalpinioideae; and the Bauhinia and Phanera clades of Cercidoideae (Sinou et al. 2020). Naming clades provides useful additional information even after a fully developed and stable subfamily and tribal classification is established. As noted by Wojciechowski (2013), use of Linnean names does not preclude a system that also defines and names clades and their overall relationships outside of the Linnean framework. Instead, the two can be considered complementary for developing a stable, flexible and useful classification of legumes.

### ﻿The new classification

The new classification of subfamily Caesalpinioideae comprises eleven tribes, which are either new, reinstated or re-circumscribed at this rank: Caesalpinieae Rchb., Cassieae Bronn, Campsiandreae LPWG, Ceratonieae Rchb., Dimorphandreae Benth., Erythrophleeae LPWG, Gleditsieae Nakai, Mimoseae Bronn, Pterogyneae LPWG, Schizolobieae Nakai, and Sclerolobieae Benth. & Hook. f. (Fig. 4). Although many of these lineages have been recognised and named in the past, either as tribes or informal generic groups, their circumscriptions have varied widely and changed over the past decades, such that all the tribes described here differ in generic membership from those previously recognised (Table 3).

**Table 3.** Comparison of the new phylogeny-based classification for Caesalpinioideae with classifications for these genera published in Advances in Legume Systematics, Part 1 (Polhill and Raven 1981) and Legumes of the World (Lewis et al. 2005).

Genera	Polhill and Vidal (1981); Irwin and Barneby (1981)	Lewis (2005a, 2005b)	New classification
*Arcoa* Urb.	Dimorphandra group (Caesalpinieae)	Umtiza clade (Caesalpinieae)	Ceratonieae
*Tetrapterocarpon* Humbert			
*Acrocarpus* Wight ex Arn.	Acrocarpus group (Caesalpinieae)		
*Ceratonia* L.	Ceratoniinae (Cassieae)		
*Umtiza* Sim	Detarieae		Gleditseae
*Gleditsia* J. Clayton	Gleditsia group (Caesalpinieae)		
*Gymnocladus* Lam.			
*Pterogyne* Tul.	Pterogyne group (Caesalpinieae)	Pterogyne group (Caesalpinieae)	Pterogyneae
*Batesia* Spruce ex Benth.	Peltophorum group (Caesalpinieae)	Batesia group (Caesalpinieae)	Cassieae
*Melanoxylum* Schott			
*Recordoxylon* Ducke			
*Vouacapoua* Aubl.		Unknown position	
*Chamaecrista* (L.) Moench	Cassiinae (Cassieae)	Cassiinae (Cassieae)	
*Cassia* L.			
*Senna* Mill.			
*Stuhlmannia* Taub.	Caesalpinia group (Caesalpinieae)	Caesalpinia group (Caesalpinieae)	Caesalpinieae
*Cordeauxia* Hemsl.			
*Cenostigma* Tul.			
*Libidibia* (DC.) Schltdl.			
*Balsamocarpon* Clos			
*Zuccagnia* Cav.			
*Hoffmannseggia* Cav.			
*Stenodrepanum* Harms			
*Erythrostemon* Klotzsch			
*Pomaria* Cav.			
*Haematoxylum* L.			
*Lophocarpinia* Burkart			
*Caesalpinia* L.			
*Coulteria* Kunth			
*Tara* Molina			
*Guilandina* L.			
*Moullava* Adans.			
*Pterolobium* R. Br. ex Wight & Arn.			
*Mezoneuron* Desf.			
*Arquita* Gagnon, G. P. Lewis & C. E. Hughes	New in 2015		
*Hererolandia* Gagnon & G. P. Lewis	New in 2016		
*Denisophytum* R. Vig.	Reinstated in 2016		
*Paubrasilia* Gagnon, H. C. Lima & G. P. Lewis	New in 2016		
*Gelrebia* Gagnon & G. P. Lewis	New in 2016		
*Hultholia* Gagnon & G. P. Lewis	New in 2016		
*Biancaea* Tod.	Reinstated in 2016		
*Ticanto* Adans.	Reinstated in 2022		
*Schizolobium* Vogel	Peltophorum group (Caesalpinieae)	Core-Peltophorum group (Caesalpinieae)	Schizolobieae
*Bussea* Harms			
*Peltophorum* (Vogel) Benth.			
*Parkinsonia* L.	Caesalpinia group (Caesalpinieae)		
*Conzattia* Rose			
*Heteroflorum* M. Sousa	New in 2005		
*Colvillea* Bojer ex Hook.	Peltophorum group (Caesalpinieae)		
*Delonix* Raf.			
*Moldenhawera* Schrad.		Moldenhawera group (Caesalp.)	Sclerolobieae
*Arapatiella* Rizzini & A. Mattos		Tachigali group (Caesalpinieae)	
*Jacqueshuberia* Ducke			
*Tachigali* Aubl.	Sclerolobium group (Caesalpinieae)		
*Diptychandra* Tul.		Unknown position	
*Dimorphandra* Schott	Dimorphandra group (Caesalpinieae)	Dimorphandra group (Caesalpinieae)	Dimorphandreae
*Mora* Benth.			
*Stachyothyrsus* Harms			
*Burkea* Benth.			
*Campsiandra* Benth.	Peltophorum group (Caesalpinieae)	Unknown position	Campsiandreae
*Dinizia* Ducke	Subf. Mimosoideae	Subf. Mimosoideae	
*Pachyelasma* Harms	Dimorphandra group (Caesalpinieae)	Dimorphandra group (Caesalpinieae)	Erythrophleeae
*Erythrophleum* Afzel. ex R. Br.			
*Sympetalandra* Stapf			Mimoseae
*Chidlowia* Hoyle		Unknown position	
Other Mimoseae genera	Subf. Mimosoideae	Subf. Mimosoideae	

Caesalpinioideae as defined here includes elements from three previously recognised major groups: part of old sense tribe Caesalpinieae, part of old sense tribe Cassieae, and the nested subfamily Mimosoideae. This broad clade has been referred to as the Mimosoideae-Caesalpinieae-Cassieae or MCC clade (Doyle 2011, 2012), or the Gleditsia-Chamaecrista-Mimosoideae or GCM clade (Marazzi et al. 2012). In 2017, Caesalpinioideae was chosen over Mimosoideae as the preferred name for this large clade, even though the two names were published at the same date (LPWG 2017). By choosing the name Caesalpinioideae, this left open the option of recognising the morphologically distinct mimosoid clade at the tribal level, as proposed here.

In their treatment of tribe Caesalpinieae in Advances in Legume Systematics Part 1, Polhill and Vidal (1981) recognised eight informal generic groups, based primarily on differences in floral morphology. Six of these generic groups, namely the Gleditsia group, Sclerolobium group, Peltophorum group, Caesalpinia group, Pterogyne group, and Dimorphandra group, are here recognised at the tribal level, albeit with modified generic compositions because most of Polhill and Vidal’s named groups have been shown to be non-monophyletic in subsequent phylogenetic analyses using molecular data (e.g., Bruneau et al. 2001, 2008) (Table 3). The monospecific Acrocarpus group of Polhill and Vidal (1981) groups with members of tribe Ceratonieae, rather than being considered a distinct tribe. In addition, the Caesalpinieae sensu Polhill and Vidal (1981) included the genus *Poeppigia* C. Presl. (as a distinct monogeneric group), which has since been shown to be placed in subfamily Dialioideae (Bruneau et al. 2001; LPWG 2017). Tribe Caesalpinieae of Polhill and Vidal (1981) had been considered to be paraphyletic, at least implicitly, for some time (Polhill and Vidal 1981; Lewis 1998) and this has since been confirmed by phylogenetic analyses which found the tribe to be polyphyletic (Bruneau et al. 2001). The other major group that forms part of Caesalpinioideae is what was considered tribe Cassieae by Irwin and Barneby (1981). Within the tribe, they recognised five disparate subtribes, Labicheinae H.S. Irwin & Barneby, Dialiinae H.S. Irwin & Barneby, Duparquetiinae H.S. Irwin & Barneby, Cassiinae Wight & Arn., and Ceratoniinae H.S. Irwin & Barneby, of which only the latter two have been placed in the Caesalpinioideae (sensu LPWG 2017) in phylogenetic analyses (Table 3). Labicheinae and Dialiinae together (along with *Poeppigia*) comprise subfamily Dialioideae, and the monospecific Duparquetiinae was raised to subfamily rank (LPWG 2017). Thus the grade of non-mimosoid caesalpinioid lineages that subtend tribe Mimoseae in Caesalpinioideae are here recognised as distinct tribes.

Below we provide a brief morphological description, overview of previous classification, and history of the phylogenetic understanding of each of the tribes proposed here. Additional details are given in the taxonomic accounts for each of these groups.

#### ﻿Tribe Ceratonieae

Tribe Ceratonieae comprises six species in four genera. The four genera had previously been placed in distinct generic groups and tribes: *Acrocarpus* Wight ex Arn. in its own generic group of Caesalpinieae by Polhill and Vidal (1981); the morphologically distinct, unisexual, and apetalous genus *Ceratonia* (Tucker 1992) in subtribe Ceratoniinae of Cassieae (Irwin and Barneby 1981); *Tetrapterocarpon* Humbert and *Arcoa* Urb. in the Dimorphandra group of Caesalpinieae, although none of these placements were considered definitive. Phylogenetic analyses of morphological and plastid sequence data showed the four genera to form a clade and to be closely related to the trigeneric clade here treated as tribe Gleditsieae, and together the two clades were placed in the informally named Umtiza clade (Herendeen et al. 2003a, 2003b; Haston et al. 2005; Bruneau et al. 2008), but subsequent combined plastid and nuclear sequence analyses did not support the monophyly of these two groups together (Manzanilla and Bruneau 2012; Zhang et al. 2020; Zhao et al. 2021). The phylogenomic analyses of Ringelberg et al. (2022) (Fig. 4) clearly indicate that each of these two clades is strongly supported as monophyletic but that they are not grouped together, supporting their recognition as distinct tribes.

These recent molecular analyses have highlighted several previously unsuspected morphological synapomorphies for Ceratonieae, the most striking of which is a bipinnate leaf (although mostly once pinnate in *Ceratonia*) terminating in a triad of pinnae arising from the same point at the apex of the rachis (Herendeen et al. 2003b; Herendeen and Herrera 2019). Tribe Ceratonieae has a highly disjunct and unusual geographic distribution occurring in Hispaniola (*Arcoa*), Madagascar (*Tetrapterocarpon*), tropical (South-)East Asia (*Acrocarpus*), and north-eastern Africa and the Mediterranean (*Ceratonia*) (Herendeen et al. 2003b; Tribe Ceratonieae, page 62).

#### ﻿Tribe Gleditsieae

The simplest of the informal generic groups recognised by Polhill and Vidal (1981) was the Gleditsia group comprising two primarily north temperate genera, *Gleditsia* J. Clayton and *Gymnocladus* Lam. The group is supported as monophyletic, and together with the South African *Umtiza* Sim, is here formally re-circumscribed as tribe Gleditsieae. *Umtiza* was previously placed in tribe Detarieae by Cowan and Polhill (1981) but was later resolved as sister to *Gleditsia* and *Gymnocladus* in phylogenetic analyses using plastid (Bruneau et al. 2001, 2008) and nuclear sequences (one locus, Manzanilla and Bruneau 2012), as well as in all recent phylogenomic analyses (Zhang et al. 2020; Zhao et al. 2021; Ringelberg et al. 2022; Fig. 4).

The 20 species of the three genera of tribe Gleditsieae occur in warm temperate regions, with several disjunctions between North and South America (*Gleditsia*), South Africa (*Umtiza*), and North America and Asia (*Gymnocladus*). Tribe Gleditsieae is characterised by several vegetative and floral synapomorphies, such as a tubular hypanthium and sepals with trichomes on the inner surface (Herendeen et al. 2003a; Tribe Gleditsieae, page 70).

#### ﻿Tribe Pterogyneae

Pterogyneae is here recognised as a new tribe comprising just the single species *Pterogynenitens* Tul. Although not included in the phylogenomic analyses of Ringelberg et al. (2022), previous molecular phylogenetic analyses based on plastid and/or nuclear DNA sequence data always resolve this monospecific genus as a phylogenetically isolated lineage, on a long branch, poorly supported relative to Cassieae and Caesalpinieae, but generally in the clade that comprises all Caesalpinioideae except Gleditsieae and Ceratonieae (Bruneau et al. 2001, 2008; Haston et al. 2003, 2005; Marazzi and Sanderson 2010; Manzanilla and Bruneau 2012; Zhang et al. 2020; Zhao et al. 2021). Pterogyneae thus appears to be a classic depauperon (Donoghue and Sanderson 2015), i.e., an old, species-poor lineage. The species is highly distinct morphologically (e.g., imparipinnate leaves with alternate leaflets and a well-formed rachis extension, small flowers in dense catkin-like racemes, the style laterally displaced at the apex of the ovary, fruits a one-seeded winged samara) and cytogenetically (2*n* = 20), sharing little in common with Cassieae, Caesalpinieae or other Caesalpinioideae (Tribe Pterogyneae, page 78). *Pterogynenitens* is an important tree of South American tropical and subtropical dry forests.

#### ﻿Tribe Cassieae

In Advances in Legume Systematics Part 1, Irwin and Barneby (1981) recognised five subtribes in their tribe Cassieae, including subtribe Cassiinae comprising the genera *Senna*, *Chamaecrista*, and *Cassia*. These three genera alongside *Batesia* Spruce ex Benth., *Melanoxylum* Schott, and *Recordoxylon* Ducke, previously placed in the Peltophorum group of Caesalpinieae by Polhill and Vidal (1981) (Table 3), form a robustly supported clade in phylogenetic analyses (Bruneau et al. 2008; Marazzi and Sanderson 2010; Manzanilla and Bruneau 2012; LPWG 2017; Zhang et al. 2020; Ringelberg et al. 2022) (Fig. 4), here recognised as tribe Cassieae. The genus *Vouacapoua* [also placed in the Peltophorum group by Polhill and Vidal (1981)], although not sampled by Ringelberg et al. (2022), is generally resolved as a member of this clade albeit with weak support (Bruneau et al. 2008; Marazzi and Sanderson 2010; Manzanilla and Bruneau 2012; LPWG 2017) and is here placed in the tribe Cassieae.

Tribe Cassieae is the largest non-mimosoid clade (in terms of species richness) in subfamily Caesalpinioideae, with 695 species, the vast majority of which are found in the genera *Chamaecrista* (361 species) and *Senna* (287 species) (Tribe Cassieae, page 83). Although broadly distributed across the tropics, most of the genera and species are found in the New World. The clade is characterised by singly pinnate or bifoliolate leaves and, in several genera, stomata on both sides of the leaflets (Herendeen et al. 2003a; Bruneau et al. 2008). Several taxa in this clade, including most species of *Senna* and *Chamaecrista*, as well as *Batesia* and *Vouacapoua*, are well-known for having notably prominent, conspicuous, abundant and unusual extrafloral nectaries (Marazzi and Sanderson 2010; Marazzi et al. 2019; Cota 2020a). Two Cassieae genera are known to nodulate, *Chamaecrista* and *Melanoxylum* (Faria et al. 2022). None of the genera in Ceratonieae, Gleditsieae, and Pterogyneae are known to nodulate.

#### ﻿Tribe Caesalpinieae

The Caesalpinia group defined by Polhill and Vidal (1981) is similar to Caesalpinieae recognised here, except that *Parkinsonia*, *Conzattia* Rose and *Lemuropisum* H. Perrier are now resolved in a separate clade (Bruneau et al. 2001, 2008; Haston et al. 2005), here treated as tribe Schizolobieae, although *Lemuropisum* has since been synonymised under *Delonix* Raf. (Bruneau and Babineau 2017). Long-standing uncertainty surrounding delimitation of the genus *Caesalpinia* L. and other genera in the Caesalpinia group (Lewis 1998; Lewis 2005b) has been resolved with the new generic system of Gagnon et al. (2015, 2016) and subsequent reinstatement of the genus *Ticanto* Adans. (Clark et al. 2022).

Tribe Caesalpinieae comprises ca. 223 species in 27 genera. Although two of these genera, *Stenodrepanum* and *Hultholia*, were not sampled by Ringelberg et al. (2022), the analyses of Gagnon et al. (2016) clearly resolved *Stenodrepanum* as sister to *Hoffmannseggia*, and *Hultholia* in a clade unresolved with *Guilandina* and the lineage that combines *Moullava* Adans., *Biancaea* Tod., *Ticanto*, *Pterolobium* R.Br. ex Wight & Arn. and *Mezoneuron*.

Species of Caesalpinieae are highly diverse in growth forms, defence mechanisms, fruit morphologies, and pollination and seed dispersal syndromes (Gagnon et al. 2016). Although there are no clear morphological synapomorphies for the tribe, a diagnostic combination of characteristics is often found, including the presence of glandular trichomes, prickles or spines, bilaterally symmetrical flowers with a modified boat-shaped lower sepal, and free stamens crowded around the pistil (Tribe Caesalpinieae, page 103). The clade is pantropically distributed, with a marked affinity for the succulent biome (Gagnon et al. 2019).

#### ﻿Tribe Schizolobieae

Tribe Schizolobieae as here circumscribed matches the core Peltophorum group (i.e., Peltophorum group s.s.) first recovered phylogenetically by Haston et al. (2003), and subsequently found in several other studies (Haston et al. 2005; Bruneau et al. 2008; Manzanilla and Bruneau 2012; Babineau and Bruneau 2017; Zhang et al. 2020; Zhao et al. 2021; Ringelberg et al. 2022). This clade differs from the informal Peltophorum group recognised by Polhill and Vidal (1981; 13 genera) and Polhill (1994; 16 genera), by excluding four genera now placed in tribe Cassieae (*Vouacapoua*, *Batesia*, *Melanoxylum*, and *Recordoxylon*), three now in tribe Sclerolobieae (*Moldenhawera* Schrad., *Jacqueshuberia* Ducke, and *Arapatiella* Rizzini & A. Mattos) and *Campsiandra* Benth. (now in tribe Campsiandreae). Schizolobieae as circumscribed here also includes *Parkinsonia* and *Conzattia*, two genera previously placed in the Caesalpinia group by Polhill and Vidal (1981), but which Lewis and Schrire (1995) had suggested might not be part of a more strictly defined Caesalpinia group, and which Polhill (1994) had included in the Peltophorum group. In addition, the tribe includes *Heteroflorum* M. Sousa, described by Sousa (2005). Although *Lemuropisum* was resolved as part of this clade (Haston et al. 2005; Bruneau et al. 2008), Babineau and Bruneau (2017) found it to be nested within *Delonix* and synonymised this monospecific genus under *Delonix*.

Tribe Schizolobieae contains ca. 42 species in eight genera. It has a pantropical distribution, and a considerable portion of the clade (i.e., the *Parkinsonia* – *Delonix* subclade; Fig. 4) is strictly conserved within the succulent biome (Ringelberg et al. 2020). Schizolobieae is not defined by any morphological synapomorphies, but most species in the clade have bipinnate leaves, yellow petals, narrow seeds, characteristic spreading umbrella-like, flat-topped tree crowns, and smooth, thin and either pale silvery metallic grey or green bark (Haston et al. 2005; Tribe Schizolobieae, page 146).

#### ﻿Tribe Sclerolobieae

The generic composition of tribe Sclerolobieae as treated here has not been recovered previously, although its constituent genera have often been associated with each other based on morphological and molecular data. The five genera of the Sclerolobieae were placed in two generic groups of tribe Caesalpinieae by Polhill and Vidal (1981) and Polhill (1994) based on morphology: *Diptychandra* Tul. and *Tachigali* Aubl. (now including *Sclerolobium* Vogel, but earlier considered distinct from *Tachigali*) were placed in the Sclerolobium group, whereas *Jacqueshuberia*, *Arapatiella*, and *Moldenhawera* were placed in the Peltophorum group. Subsequent molecular phylogenetic studies generally also resolved the five genera in two separate clades, but with different generic composition from those of the informal groups of Polhill and Vidal (1981) and Polhill (1994). One strongly supported clade grouped *Arapatiella*, *Jacqueshuberia* and *Tachigali*, as found here (Fig. 4), and a separate less well supported clade (or grade) included *Diptychandra* and *Moldenhawera* (Bruneau et al. 2008; Marazzi and Sanderson 2010; Manzanilla and Bruneau 2012; LPWG 2017; Zhang et al. 2020), which has sometimes been resolved as part of a grade subtending the mimosoid clade (Bruneau et al. 2008; Marazzi and Sanderson 2010; Manzanilla and Bruneau 2012). In the recent phylogenomic analyses of Ringelberg et al. (2022; Fig. 4), the tribe is subtended by a short branch with notable gene tree conflict, whereas the *Arapatiella*, *Jacqueshuberia* and *Tachigali* subclade is supported by a long branch. This short branch and gene tree conflict likely explain why the five genera have not been resolved as a clade, but rather as two separate clades in previous phylogenies. In addition, there is evidence to suggest that there may be cytonuclear discordance. In recent phylogenomic analyses, although not all genera have been sampled, plastid data strongly support the *Jacqueshuberia*, *Arapatiella* and *Tachigali* subclade, with *Diptychandra* and *Moldenhawera* forming a lineage subtending the mimosoid clade (Zhang et al. 2020), whereas nuclear sequence data group the two lineages as a clade (Zhao et al. 2021; Ringelberg et al. 2022).

As defined here, tribe Sclerolobieae is restricted to the Neotropics, and comprises ca. 113 species, most of which are in the genus *Tachigali* (80–90 species; Tribe Sclerolobieae, page 165). Several species of *Tachigali* are known to form close co-evolutionary associations with ants (Chomicki et al. 2015). Morphologically, each of the five genera is highly distinct in leaf morphology (pinnate or bipinnate leaves), floral symmetry (radial or bilateral), pollination syndrome (bees or birds), pollen presentation (monads or tetrads), fruit morphology, seed morphology (winged or non-winged), and dispersal syndrome (autochory, hydrochory, or anemochory). Thus, the tribe is not defined by obvious morphological synapomorphies, although there is a tendency for the occurrence of distinctively divided or foliaceous stipules (except for *Diptychandra*; Tribe Sclerolobieae, page 165) and nodulation with a fixation thread type of nodule anatomy in *Tachigali*, *Moldenhawera* and *Jacqueshuberia*, three of the nine non-mimosoid Caesalpinioideae genera known to nodulate (Sprent 2000; Faria et al. 2022).

#### ﻿Tribe Dimorphandreae

The four genera of the Dimorphandreae, *Dimorphandra*, *Mora* Benth., *Stachyothyrsus* Harms, and *Burkea* Benth., form a clade in most molecular phylogenetic studies (Bruneau et al. 2008; Marazzi and Sanderson 2010; Manzanilla and Bruneau 2012; Ringelberg et al. 2022), corresponding to the Dimorphandra group A of Bruneau et al. (2008). This is a narrower definition than the morphologically-based informal Dimorphandra group sensu Polhill and Vidal (1981), Polhill (1994) and Lewis (2005b), which also included *Erythrophleum* and *Pachyelasma* Harms (now tribe Erythrophleeae), *Sympetalandra* and *Chidlowia* (now placed in tribe Mimoseae), as well as *Arcoa* and *Tetrapterocarpon* (now in tribe Ceratonieae), a group subsequently shown to be non-monophyletic (Bruneau et al. 2001, 2008; Manzanilla and Bruneau 2012; Zhang et al. 2020). The Dimorphandra group sensu Polhill and Vidal (1981) comprised a diverse assemblage of genera, many of which share certain characteristics with tribe Mimoseae (e.g., bipinnate leaves, numerous, small, regular flowers in spiciform racemes, and introrse sagittate anthers; Elias 1981a; Polhill and Vidal 1981; Luckow et al. 2000) and was considered a ‘‘transitional link’’ between the then caesalpinioids and mimosoids (Polhill and Vidal 1981; Luckow et al. 2000, 2003). As newly circumscribed, tribe Dimorphandreae is morphologically more coherent, including four genera, all with spicate inflorescences and pentamerous, diplostemonous flowers.

The four genera of the Dimorphandreae contain 35 species. However, 26 are in *Dimorphandra* s.l., which is paraphyletic (e.g., LPWG 2017; Ringelberg et al. 2022; see Tribe Dimorphandreae, page 177), suggesting that generic re-delimitation will be necessary. The clade has an amphi-Atlantic distribution (Neotropics and tropical Africa) spanning a variety of biomes. Nodulation is reported in two species of *Dimorphandra*, whereas *Mora* and *Burkea* are confirmed as non-nodulators (Faria et al. 2022).

It is also notable that while tribes Sclerolobieae, Schizolobieae and Dimorphandreae are each supported as monophyletic in recent phylogenomic analyses, the relationships among these three lineages are weakly supported and characterised by high gene tree conflict (Suppl. material 3; Ringelberg et al. 2022: Fig. 3).

#### ﻿Tribe Campsiandreae

*Dinizia* and *Campsiandra*, previously placed in Mimosoideae and the Peltophorum group of Caesalpinieae respectively (Table 3), have only been recovered as sister genera (Fig. 4) in one previous study (Zhang et al. 2020), although the two genera have generally been resolved in the same large clade that included Mimosoideae and subtending lineages (Bruneau et al. 2008; Marazzi and Sanderson 2010; Manzanilla and Bruneau 2012). Morphologically, *Dinizia* was previously considered to be a member of subfamily Mimosoideae (Lewis and Elias 1981; Pohill 1994; Luckow 2005), but molecular phylogenetic studies have consistently placed the genus among the grade of non-mimosoid Caesalpinioideae genera subtending the mimosoid clade, albeit with varying sister group relationships (Luckow et al. 2000, 2003; Wojciechowski et al. 2004; Manzanilla and Bruneau 2012; Zhang et al. 2020; Ringelberg et al. 2022; Fig. 4). These two genera exhibit disparate morphology, although they share perigynous flowers with a tubular hypanthium and showy stamens exserted from the corolla. This morphological distinctiveness is mirrored in the molecular analyses, where relatively long branches subtend the two genera. Although we here place these two morphologically disparate genera together in tribe Campsiandreae, Ringelberg et al. (2022) noted that long-branch attraction could play a role in grouping *Dinizia* and *Campsiandra* together in a clade. A similar phylogenetic pattern is observed in the plastid phylogenomic analyses of Zhang et al. (2020), in which the two genera also form a clade subtended by a short branch.

Tribe Campsiandreae comprises 5 to 22 species (the genus *Campsiandra* needs to be revised because several species are of dubious taxonomic status), only two of which are in *Dinizia*. The tribe is restricted to tropical rainforests in South America. Nodulation is reported in one species each of *Dinizia* and *Campsiandra* (Faria et al. 2022), and is known to be absent in the other species of *Dinizia*.

#### ﻿Tribe Erythrophleeae

*Erythrophleum* and *Pachyelasma* have rarely been recovered as sister genera before (Herendeen et al. 2003a). Nevertheless, most previous studies based primarily on plastid sequence data placed these two genera as successive sisters to the mimosoid clade (Bruneau et al. 2001, 2008; Luckow et al. 2003; Bouchenak-Khelladi et al. 2010; Marazzi and Sanderson 2010; Manzanilla and Bruneau 2012; Kyalangalilwa et al. 2013; Zhang et al. 2020), as found in the plastid phylogeny of Ringelberg et al. (2022), indicating another case of possible cytonuclear discordance and potentially explaining why the two genera have not previously been grouped. No clear morphological synapomorphy has been identified for the clade, but *Erythrophleum* and *Pachyelasma* share a combination of morphological traits only rarely found in non-Mimoseae Caesalpinioideae (e.g., bipinnate leaves, small pedicellate perigynous flowers in dense spicate racemes), and both genera have highly toxic alkaloids and saponins (Tribe Erythrophleeae, page 193).

The Erythrophleeae, with 12 species in *Erythrophleum* and one species in *Pachyelasma*, is restricted to the Old World tropics (Africa, Asia and Australia). *Erythrophleum* is reported to nodulate.

#### ﻿Tribe Mimoseae

Recognising the mimosoid clade as the newly circumscribed tribe Mimoseae results in by far the largest tribe in subfamily Caesalpinioideae in terms of numbers of species (ca. 3500) and genera (100). Previous tribal classifications of the mimosoid clade (i.e., former subfamily Mimosoideae) recognised five tribes: Mimoseae, Acacieae Benth., Ingeae Benth., Parkieae (Wight & Arn.) Benth., and Mimozygantheae Burkart (Elias 1981a). In formulating the new tribal classification for subfamily Caesalpinioideae, the recognition of the mimosoid clade as the reinstated and re-circumscribed tribe Mimoseae is proposed for two main reasons. First, four of the five former tribes of Mimosoideae are now known to be non-monophyletic and the fifth (the monospecific Mimozygantheae) to be nested within the former Mimoseae (Luckow et al. 2003, 2005; LPWG 2013, 2017; Koenen et al. 2020a; Ringelberg et al. 2022). Second, the ladder-like phylogenetic structure within the mimosoid clade means that any finer-scale tribal divisions would inevitably result in an undesirable proliferation of many small Linnean tribes, including a large number of monogeneric tribes (Koenen et al. 2020a).

There has been ongoing debate about which genera are included in the mimosoid clade (Luckow et al. 2000, 2003; Lewis et al. 2005; Manzanilla and Bruneau 2012). As circumscribed here, tribe Mimoseae matches the former subfamily Mimosoideae (Bentham 1865; Hutchinson 1964; Polhill and Raven 1981; Lewis et al. 2005), with three exceptions (Koenen et al. 2020a; Ringelberg et al. 2022; Tribe Mimoseae, page 201). First, *Sympetalandra*, previously considered to be a non-mimosoid caesalpinioid based on morphology (Polhill and Vidal 1981; Polhill 1994; Lewis 2005b), is firmly nested within the Mimoseae as an isolated early-diverging lineage (Fig. 5; Ringelberg et al. 2022). Similarly, *Chidlowia*, once considered part of Caesalpinioideae (Lewis 2005b), is also nested within the Mimoseae, as initially found by Manzanilla and Bruneau (2012) and supported by LPWG (2017), Koenen et al. (2020a), and Ringelberg et al. (2022) (Fig. 5). Finally, *Dinizia* was previously considered a genus of Mimosoideae (Polhill and Vidal 1981; Polhill 1994; Luckow 2005), but is resolved in the non-mimosoid Caesalpinioideae (now in tribe Campsiandreae, page 187).

Tribe Mimoseae is diagnosed by valvate petal aestivation (with exceptions in *Chidlowia* and *Sympetalandra*), bipinnate leaves (except *Inga* and a few scattered species in other genera), flowers that are relatively small with reduced perianth and showy androecium, mostly clustered in compact inflorescences that are commonly capitate or spicate (Tribe Mimoseae, page 201), and the presence of symbiosome-type (as opposed to fixation-thread-type) root nodules (Faria et al. 2022).

Tribe Mimoseae, as circumscribed here, is robustly supported as monophyletic and is subtended by a relatively long branch (Fig. 4). Within Mimoseae the phylogeny takes the form of an extensive unbalanced ladder-like topology (Fig. 5), which is not readily amenable to division into a manageable number of rank-based Linnean taxa. However, given the large size of the tribe, some form of classificatory structure is needed, and here we present a clade-based classification system for the tribe with two nested higher-level named clades – a core mimosoid clade and the ingoid clade – alongside a set of 17 named lower-level clades following Koenen et al. (2020a) and Ringelberg et al. (2022) (Fig. 5). Fourteen of the 100 genera of Mimoseae remain unplaced in any of these lower-level clades, eight of which are resolved in two grades, and six of which are phylogenetically isolated monogeneric lineages (Fig. 5).

The core mimosoid clade as delimited by Koenen et al. (2020a) is well supported, subtended by a notably long branch, and includes all Mimoseae except the Adenanthera and Entada clades, the two monogeneric lineages *Sympetalandra* and *Chidlowia*, and the Newtonia grade (Fig. 5). The core mimosoid clade includes all Mimoseae with armature, with the exceptions of a single spinescent species of *Entada* and of two species of *Pseudoprosopis* Harms, which are armed with modified woody tendrils, and which occur outside the clade (Koenen et al. 2020a).

The ingoid clade, delimited by Koenen et al. (2020a), is also strongly supported, includes ca. 2000 species, i.e., almost two-thirds of Mimoseae species, and comprises genera of the Senegalia grade and nine named clades (Calliandra, Zapoteca, Cojoba, Pithecellobium, Archidendron, Samanea, Jupunba, Albizia, and Inga clades) (Fig. 5). The ingoid clade groups all genera of Mimoseae with polystemonous flowers except *Vachellia*. A synandrous androecium is exclusively found in this clade and characterises most, but not all of its genera (Koenen et al. 2020a). Based on the limited sample of mimosoid chloroplast genome sequences currently available, an expanded Inverted Repeat region of the chloroplast genome is also restricted to this clade (Dugas et al. 2015; Wang et al. 2017).

### ﻿Taxonomy

Based on the phylogeny of subfamily Caesalpinioideae presented here, we recognise eleven tribes (Fig. 4) and 17 formally named clades within tribe Mimoseae (Fig. 5). The new classification proposed here recognises clades that are strongly supported in the phylogenomic analyses of Koenen et al. (2020a) and Ringelberg et al. (2022), many of which were already known from earlier phylogenetic studies. This classification is proposed and endorsed by the legume systematics community as reflected in the use of the Legume Phylogeny Working Group (LPWG) as the authority of the new tribes. Although an uncommon practice in botanical nomenclature, ascribing the new tribe names to the collective known as the “Legume Phylogeny Working Group” is accepted under the botanical code as stipulated in Chapter VI, Section 1 (Author Citations) and follows the approach previously used for the LPWG (2017) subfamily classification. The Legume Phylogeny Working Group authorship gives due credit to the legume systematics community and reflects the important collaborative contributions from multiple research groups over the decades that have laid the foundations for this classification.

We provide a key to genera, as well as taxonomic descriptions and notes for tribes, named clades, and all 163 genera, and illustrate the diversity of growth forms, foliage, flowers and fruits for nearly all genera. We also provide a distribution map of the native range for each genus, based on quality-controlled herbarium specimen localities and floristic surveys. The occurrence data for Mimoseae are from Ringelberg et al. (2023), whereas the remainder were newly assembled here. See Suppl. material 1 for sources of occurrence data and detailed data cleaning protocols. All occurrence data have been made available on Zenodo (https://zenodo.org/doi/10.5281/zenodo.8407862), unless stated otherwise. A tree file of the Caesalpinioideae phylogeny presented here is available from Ringelberg et al. (2022, 2023).

## ﻿﻿1. Subfamily Caesalpinioideae

The Legume Phylogeny Working Group (LPWG)*

* Luciano Paganucci de Queiroz^2^, Anne Bruneau^1^ , Jens J. Ringelberg^3,4^, Leonardo M. Borges^5^, Roseli Lopes da Costa Bortoluzzi^6^, Gillian K. Brown^7^, Domingos B. O. S. Cardoso^8,9^, Ruth P. Clark^10^, Adilva de Souza Conceição^11^, Matheus Martins Teixeira Cota^2^, Else Demeulenaere^12^, Rodrigo Duno de Stefano^13^, John E. Ebinger^14^, Julia Ferm^15^, Andrés Fonseca-Cortés^2^, Edeline Gagnon^16,17,18^, Rosaura Grether^19^, Ethiéne Guerra^20^, Elspeth Haston^18^, Patrick S. Herendeen^21^, Héctor M. Hernández^22^, Helen C. F. Hopkins^10^, Isau Huamantupa-Chuquimaco^23^, Colin E. Hughes^3^, Stefanie M. Ickert-Bond^24^, João Iganci^20,25^, Erik J. M. Koenen^26^, Gwilym P. Lewis^10^, Haroldo Cavalcante de Lima^8,27^, Alexandre Gibau de Lima^8,28^, Melissa Luckow^29^, Brigitte Marazzi^30^, Bruce R. Maslin^31,32^, Matías Morales^33,34^, Marli Pires Morim^8^, Daniel J. Murphy^35^, Shawn A. O’Donnell^36^, Filipe Gomes Oliveira^2^, Ana Carla da Silva Oliveira^2^, Juliana Gastaldello Rando^37^, Pétala Gomes Ribeiro^2^, Carolina Lima Ribeiro^2^, Felipe da Silva Santos^2^, David S. Seigler^38^, Guilherme Sousa da Silva^39^, Marcelo F. Simon^40^, Marcos Vinícius Batista Soares^20^, Vanessa Terra^41^

Citation: Legume Phylogeny Working Group, Queiroz LP et al. (2024) 1. Subfamily Caesalpinioideae. In: Bruneau A, Queiroz LP, Ringelberg JJ (Eds) Advances in Legume Systematics 14. Classification of Caesalpinioideae. Part 2: Higher-level classification. PhytoKeys 240: 32–54. https://doi.org/10.3897/phytokeys.240.101716

### ﻿Subfamily Caesalpinioideae

Taxon classificationPlantaeFabalesFabaceae

DC., Prodr. [A. P. de Candolle] 2: 473. 1825.

Caesalpiniaceae R. Br., in M. Flinders, Voy. Terra Austral. 2: 551. 1814. Type: Caesalpinia L.

#### Type.

*Caesalpinia* L.

#### Description.

Trees, shrubs, lianas, suffruticose or functionally herbaceous, occasionally aquatic, either unarmed or commonly armed with prickles, spines, or thorns; specialised extrafloral nectaries often present on the petiole and/or on the primary and secondary leaf rachides, usually between pinnae or leaflet pairs, more rarely stipular or bracteal. **Stipules** in lateral position and free or absent, usually entire, less frequently divided or spinescent. **Leaves** usually pulvinate, bipinnate, otherwise pinnate (sometimes both types on the same plant) and then mostly paripinnate, rarely imparipinnate, less often bifoliolate, modified into phyllodes or lacking, arrangement of the pinnae and leaflets mostly opposite, rarely alternate; stipels rare and not to be confused with the more commonly present paraphyllidia. **Inflorescences** globose or ellipsoid capitula, spicate, paniculate, racemose or in fascicles; bracteoles commonly small or absent. **Flowers** usually bisexual, rarely unisexual (species dioecious or monoecious), or bisexual flowers combined with unisexual and/or sterile flowers in heteromorphic inflorescences (Mimoseae), radially, less frequently bilaterally symmetrical, or asymmetrical; hypanthium lacking or cupular, rarely tubular; sepals (3) 5 (6–8), free or fused; petals (3) 5 (6–8), free or fused, the sepals or petals or both sometimes lacking, aestivation valvate (Mimoseae) or imbricate and then the adaxial petal innermost; stamens commonly diplostemonous or haplostemonous, sometimes reduced to 3 or 4 (in some *Mimosa* species), frequently polystemonous (to 100+ in some Mimoseae), free or fused, sometimes heteromorphic, some or all sometimes modified or staminodial, anthers basifixed or dorsifixed, often with a stipitate or sessile apical gland, dehiscing via longitudinal slits or apical or basal poricidal slits or pores; pollen in tricolporate monads, or commonly in tetrads, bitetrads or polyads (most Mimoseae); gynoecium uni- or rarely polycarpellate, 1–many-ovulate. **Fruit** typically dry and dehiscent, either a legume (dehiscent along both sutures) or a follicle (dehiscent along the adaxial suture only), or dry and segmented into one-seeded articles, either without a persistent margin (a lomentum) or with a persistent margins forming a replum like a frame (a craspedium), sometimes indehiscent and somewhat fleshy (an indehiscent legume), or dry and winged (a samara), when dehiscent with papery, leathery, or woody valves, dehiscence passive (inert valves), elastic, or explosive (the valves becoming curved, spirally coiled, or arched backwards), less frequently with entire valves, but with the endocarp segmented into one-seeded articles (a cryptolomentum). **Seeds** usually with an open (U-shaped) or closed (O-shaped) pleurogram on both faces or lacking a pleurogram, sometimes with a fleshy aril or sarcotesta, sometimes winged, in cross section terete (with a 1:1 ratio), compressed (with more or less 2:1 ratio) or flattened (with > 4:1 ratio; Gunn 1991); hilum usually apical, lens usually inconspicuous; embryo straight.

#### Chromosome number.

2*n* mostly 24, 26, 28, but also reported 2*n* = 14, 16, 20, 22, 36, 52, 54, 56, 72, 78, 104, 112.

#### Included taxa.

Caesalpinioideae in its emended circumscription contains eleven tribes, 163 genera and ca. 4680 species. Tribes: Caesalpinieae Rchb. (27 genera / ca. 223 species), Campsiandreae LPWG (2 / 5–22), Cassieae Bronn (7 / 695), Ceratonieae Rchb. (4 / 6), Dimorphandreae Benth. (4 / 35), Erythrophleeae LPWG (2 / 13), Gleditsieae Nakai (3 / 20), Mimoseae Bronn (100 / ca. 3510), Pterogyneae LPWG (1 / 1), Schizolobieae Nakai (8 / 42–43), Sclerolobieae Benth. & Hook. f. (5 / ca. 113) (Fig. 4).

#### Clade-based definition.

The most inclusive crown clade containing *Arcoagonavensis* Urb. and *Mimosapudica* L., but not *Bobgunniafistuloides* (Harms) J.H. Kirkbr. & Wiersema, *Duparquetiaorchidacea* Baill., or *Poeppigiaprocera* C. Presl.

#### Distribution and ecology.

Pantropical, common in both wet and dry regions, with a handful of species extending to the temperate zone, less frequently frost-tolerant (*Gleditsia* J. Clayton, *Gymnocladus* Lam. and some species of *Acacia* Mill., *Desmanthus* Willd. and *Senna* Mill.). Caesalpinioideae species are infrequent above 2500 m in the tropics and are largely absent from mid- and high-elevation tropical montane forests. Generic diversity is highest in the Neotropics, and there are important centres of high species diversity in Mexico and Central America, central-east South America, Africa, Madagascar, parts of South East Asia and Australia (Fig. 3, Table 1).

#### Notes.

This clade was referred to as the Mimosoideae-Caesalpinieae-Cassieae (MCC clade) (Doyle 2011, 2012) or the Gleditsia-Chamaecrista-Mimosoideae (GCM clade) (Marazzi et al. 2012). In LPWG (2017), Caesalpinioideae was chosen over Mimosoideae as the preferred name for the MCC clade, leaving open the option for naming the morphologically distinct mimosoid clade at the tribal level, as is done here (Fig. 5; see Mimoseae, page 201).

In the following treatments, the type species of different taxa are presented. Their homotypic (nomenclatural) synonyms are indicated by the identity symbol (≡) and heterotypic (taxonomic) synonyms by the equality symbol (=), as specified in the International Code of Nomenclature for algae, fungi, and plants (Turland et al. 2018).

### ﻿﻿Key to genera of subfamily Caesalpinioideae

Some sections of the key, for particular groups, were developed and modified from previous publications [e.g., Brenan (1955), Lewis and Elias (1981), Polhill and Vidal (1981), Nielsen (1992), Barneby and Grimes (1996), Queiroz (2009), Gagnon et al. (2016), Brown et al. (2022), Hughes et al. (2022b), Lima et al. (2022), Soares et al. (2022), Souza et al. (2022b), Tamayo-Cen et al. (2022)]. The distribution data used in some couplets refers to the native geographic range of the genus in question. Genera that key out in more than one place in the key are indicated by an asterisk (*). See Glossary, Schemes 1–7, for definitions and illustrations of selected morphological terms pertinent to Caesalpinioideae.

**Table:** 

1	Plants aphyllous or leaves reduced to phyllodes of petiolar or leaf rachis origin, lacking leaflets	**2**
–	Plants with developed leaves	**7**
2	Petals imbricate in bud, the uppermost petal in the inner position; some or all anthers dehiscing by pores; flowers solitary or in racemes	**3**
–	Petals valvate in bud; all anthers dehiscing through longitudinal slits; flowers densely clustered in capitate or spicate inflorescences	**4**
3	Erect herbs or subshrubs; stipules cordiform clasping the stem internodes; flowers isolated in stipule axils; pedicel with a pair of alternate bracteoles; fruits elastically dehiscent, the valves twisting upon dehiscence	* ***Chamaecrista***
–	Shrubs with hard woody branches; stipules small, not covering the stems; flowers in short corymbiform racemes; pedicels lacking bracteoles; fruits indehiscent or passively dehiscent, the valves not becoming twisted	* ***Senna***
4	Flowers polystemonous; seeds arilate	* ***Acacia***
–	Flowers diplostemonous; seeds not arilate	**5**
5	Aphyllous trees or shrubs due to precocious leaf falling, profusely armed with spine-tipped, rigid, straight, cylindrical thorns; fruits indehiscent, compressed-turgid, pulp mealy or spongy	* ***Neltuma***
–	Aphyllous or phyllodial subshrubs or shrubs, either unarmed or armed with stipular spines or prickles; fruit a craspedium, a lomentum or dehiscent along the ventral suture	**6**
6	Subshrubs or shrubs, unarmed or armed with scattered cauline and phyllodial recurved prickles; stems not striate; leaves modified into phyllodes, not caducous	* ***Mimosa***
–	Small shrubs armed with stipular spines, the shoots also often spinescent, tapered to a hard point; stems green, photosynthetic, striate with longitudinal, golden corky ridges; leaves not modified in phyllodia, early caducous	* ***Prosopidastrum***
7	All or some leaves bipinnate	**8**
–	Leaves once pinnate or bifoliolate	**179** (page 51)
8	Petals valvate in bud; seeds often with a U-shaped or O-shaped pleurogram	**9**
–	Petals imbricate in bud, the uppermost petal in the inner position; seeds lacking a pleurogram	**126** (page 46)
9	Flowers haplostemonous or diplostemonous, i.e., fertile stamens the same or exactly twice the number of petals	**10**
–	Flowers polystemonous, rarely less than twice but always with more than the number of petals	**64** (page 40)
10	Flowers in globose or ellipsoid capitula	**11**
–	Flowers in elongated spikes or spiciform racemes	**29**
11	Capitula with clearly marked floral differentiation, the basal flowers often but not always with elongated showy staminodes	**12**
–	Capitula without marked floral differentiation (flowers mostly hermaphrodite or staminate) and the basal flowers always without elongated showy staminodes	**15**
12	Trees; calyx with imbricate lobes; capitula either with sterile staminodial flowers at base, fertile flowers at apex and modified staminate, nectar-secreting flowers between, or capitula with fertile flowers at base and modified nectar-secreting flowers at apex	* ***Parkia***
–	Subshrubs, small shrubs, sometimes aquatic herbs; calyx with valvate sepals, capitula with sterile staminoidal flowers at base and fertile hermaphrodite flowers at apex	**13**
13	Fruits stipitate, oblong, seeds transversely oriented; stipules ovate to lanceolate and striately veined	***Neptunia***
–	Fruits subsessile, linear, seeds obliquely or longitudinally oriented; stipules subulate or setiform with an auriculate base	**14**
14	Fruits sub-cylindrical, tardily dehiscent along both sutures from the apex, valves lignified; anther glands present; these stipitate, terminal, or claviform (orbicular on a filiform stalk); pollen aggregated into tetrahedral tetrads	* ***Mezcala***
–	Fruits dorsi-ventrally ﬂattened, passively dehiscent, valves chartaceous or coriaceous; anther glands absent; pollen dispersed in monads	***Desmanthus***
15	Fruits breaking up into 1-seeded articles, leaving persistent margins or not, or the valves falling entire but leaving a frame made by persistent margins	**16**
–	Fruits indehiscent or dehiscent through one or both margins, not articulated	**18**
16	Fruit a craspedium, i.e., with persistent margins (like a frame) after articles dispersed or the entire valves have fallen; unarmed or branches armed with prickles (fruit rarely a lomentum, i.e. lacking persistent margins, but then the plant is always armed with prickles)	***Mimosa***
–	Fruits lomentaceous, the articles falling but the fruit lacking persistent margins; plants armed with stipular spines, sometimes additionally with branches modified into thorns, but lacking prickles	**17**
17	Fruits stipitate with margins undulate, not thickened; seeds compressed, discoid; stipular spines ± straight; stems slightly ribbed or not and not ending in a sharp point; Paraguay and southern Bolivia	***Piptadeniopsis***
–	Fruits subsessile with straight and thickened margins; seeds ± bulky; stipular spines ± curved; shoots spinescent, ribbed and green, ending in a sharp point; Mexico (Baja California) and Patagonian Argentina	* ***Prosopidastrum***
18	Plants armed with stipular spines; fruits indehiscent	**19**
–	Plants unarmed; fruits either dehiscent or indehiscent	**21**
19	Fruits woody, more or less tightly spirally coiled, irregularly and openly coiled or at least somewhat curved, always > 2-seeded; arid and semi-arid areas of North America (southern USA and northern Mexico) and South America (Peru to Bolivia, Argentina and Chile)	* ***Strombocarpa***
–	Fruits with papery valves, falcate ovate, not coiled or spiralled, 1 (2)-seeded	**20**
20	Calyx lobes imbricate in bud; stems not ribbed; leaves and almost sessile capitate inflorescences attached to brachyblasts arising between the straight spines; fruits narrowly winged along the upper suture; Argentina, Bolivia and Paraguay	***Mimozyganthus***
–	Calyx lobes valvate in bud; stems ribbed and green; capitula pedunculate, axillary; brachyblasts absent; spines curved; fruits with a broad arched wing along the lower suture; Namaqualand in South Africa and Namibia	***Xerocladia***
21	Leaves with exactly 1 pair of pinnae and 3 leaflets per pinna; capitula pedunculate and solitary or fasciculate on axillary brachyblasts; fruits obovate, 1-seeded, small (to 3.2 cm long), dehiscent through both margins; seeds compressed, cordiform; Kaho’olawe island (Hawaii)	***Kanaloa***
–	Leaves with more than one pair of pinnae or > 3 leaflets or both; brachyblasts present or absent; fruits larger (over 5 cm long) and mostly linear to oblong, dehiscent or indehiscent; seeds not cordiform	**22**
22	Plants from Africa, Asia and Malesia	**23**
–	Plants from America	**25**
23	Fruit valves coriaceous, narrowly winged along the margins, splitting at edges but not separating over seed-chambers; floral bracts peltate, persistent; Malesia and Papuasia	***Schleinitzia***
–	Fruit valves ligneous or stiffly coriaceous, elastically dehiscent from the apex through both sutures; floral bracts caducous; Africa (including Madagascar)	**24**
24	Leaves emerging from lateral brachyblasts; stipules joined at base and to the base of petiole, persistent; Madagascar	***Alantsilodendron***
–	Leaves on branches lacking brachyblasts; stipules free and caducous; mostly continental Africa (2 species in northern Madagascar)	***Xylia***
25	Fruits held erect above foliage and elastically dehiscent from the apex along both sutures; brachyblasts present sheathed in persistent stipules; dry vegetation of central Mexico	**26**
–	Fruits not erect, indehiscent or passively dehiscent along one or both margins; widely distributed from southern USA to South America	**27**
26	Shrubs to 1 m tall, profusely branched from the base; leaves and inflorescences emerging from lateral brachyblasts; stamens 5; fruits oblanceolate, plano-compressed, with thickened margins	***Calliandropsis***
–	Multi-stemmed treelet or large shrub to 3.5 m; inflorescences arising from leaf axils; stamens 10; fruits linear, terete or sub-terete, the margins not noticeably thickened	* ***Mezcala***
27	Fruit woody, dehiscent through one margin (a follicle), with the margins constricted between the seeds; seeds flat and orbicular with a narrowly winged margin; widespread deciduous trees in South America (naturalised in the West Indies)	***Anadenanthera***
–	Fruit with straight margins and chartaceous, coriaceous or woody valves, dehiscent through one or both margins, if woody and dehiscent from one margin then with bulky seeds; seeds flat or bulky but never orbicular with a winged margin	**28**
28	Calyx with imbricate lobes in bud; anthers with an apical gland and glabrous; fruit indehiscent or passively dehiscent with woody valves; seeds compressed or not; evergreen trees from Amazonia	* ***Parkia***
–	Calyx with valvate lobes in bud; anthers eglandular, sometimes the connective with a small rounded or hooded apiculum, usually pilose, occasionally glabrous; fruit with chartaceous or coriaceous valves, inertly dehiscent along one or usually both sutures; seeds flat-compressed; deciduous shrubs or small trees from North and Central America, one species in dry vegetation of northern and north-western South America (*L.leucocephala* a pantropical weed)	***Leucaena***
29	Fruits breaking into 1-seeded articles resulting from fragmentation of the entire fruit wall or from just the endocarp	**30**
–	Fruits not breaking into 1-seeded articles	**35**
30	Flowers with the pedicels articulated near the middle, persisting after the flowers fall; leaflets alternate; fruits dehiscing through both margins in two entire valves but the endocarp splitting into 1-seeded papery envelopes; unarmed trees	***Plathymenia***
–	Flowers sessile or the pedicel not articulated; leaflets mostly opposite; fruits with the margins framing 1-seeded articles or the entire valves which break away from the persistent margin (craspedium); trees, shrubs, geoxylic subshrubs or lianas, unarmed or armed with prickles or stipular spines	**31**
31	Leaves lacking extrafloral nectaries on petiole and leaf rachis	**32**
–	Leaves with extrafloral nectaries on petiole and/or leaf rachis or on shoot immediately beneath the base of stipules	**33**
32	Anther glands absent; plants unarmed or armed with infranodal or internodal prickles; flowers in capitula or spikes; epicarp remaining attached to endocarp on mature fruits	* ***Mimosa***
–	Anther glands usually present; plants unarmed, rarely (*Entadaspinescens*) with stipular spines; flowers in spikes; epicarp separating from the endocarp on mature fruits	* ***Entada***
33	Plants unarmed; epicarp separating from the endocarp on mature fruits; extrafloral nectaries mostly on the shoot immediately beneath the base of stipules, if present on petiole or leaf rachis then plant from Madagascar	* ***Entada***
–	Plants unarmed or armed with prickles; epicarp remaining attached to endocarp on mature fruits; extrafloral nectaries on the petiole and/or leaf rachis; plants from tropical America and continental sub-Saharan Africa	**34**
34	Habit variable; anther glands absent; flowers haplostemonous (in *Mimosamyriadenia*) or diplostemonous, pentamerous, or tetramerous, in capitula or spikes; tropical America	* ***Mimosa***
–	Lianas, rarely (in African *Adenopodiarotundifolia*) shrubs or treelets; anther glands present; flowers diplostemonous, pentamerous, in spikes; distributed in Mexico and Central America and disjunctly across sub-Saharan Africa	* ***Adenopodia***
35	Plants from the Americas	**36**
–	Plants from Africa, Asia, Malesia and Australia	**48**
36	Plants armed with stipular spines, solitary or paired thorns, spinescent shoots or prickles	**37**
–	Plants unarmed	**40**
37	Fruits dehiscing through both margins, with papery valves	**38**
–	Fruits indehiscent, woody or pulpy, rarely with a thin mesocarp, the endocarp fragmented into 1-seeded chambers	**39**
38	Branches armed with internodal or infranodal prickles; flowers with a mostly campanulate corolla	***Piptadenia***
–	Branches armed with paired recurved stipular spines; flowers with a slender tubular corolla with very short teeth	***Lachesiodendron***
39	Plants armed with stipular spines	* ***Strombocarpa***
–	Plants armed with axillary, uninodal, solitary or paired thorns or spinescent shoots	* ***Neltuma***
40	Flowers with 5 fertile stamens and 5–15 linear staminodes; fruits sickle-shaped and explosively dehiscing along both sutures, the robust woody valves twisting and becoming arched backwards	* ***Pentaclethra***
–	Flowers with 10 fertile stamens, staminodes absent; fruits never sickle-shaped, indehiscent or dehiscent through one or both margins, if dehiscent then passively so and the valves papery	**41**
41	Branches and leaves lacking ferruginous granular trichomes	**42**
–	Young branches and leaves usually covered with ferruginous granular trichomes	**45**
42	Fruits compressed turgid, indehiscent, with the mesocarp thick and pulpy or spongy and the endocarp segmented in one seeded chambers	* ***Neltuma***
–	Fruits compressed, dehiscent through one or both margins, with a thin mesocarp and an indistinct endocarp	**43**
43	Fruit a legume, dehiscing along both margins; flowers mostly with reddish petals and stamens	***Parapiptadenia***
–	Fruit a follicle, dehiscing along one margin only; flowers with greenish petals and whitish stamens	**44**
44	Extrafloral nectary between or just below the first pair of pinnae; spikes mostly solitary in axils of coevally developing leaves; fruits moniliform, with deeply constricted margins and thick coriaceous and pubescent valves	***Pityrocarpa***
–	Extrafloral nectary between the base and the middle of the petiole; spikes mostly clustered in terminal efoliate pseudoracemes or below the coeval leaves; fruits with a linear or oblong body, straight or shallowly sinuous margins and thin to thick woody and glabrous valves	***Marlimorimia***
45	Branches and leaves with strong garlic smell; ferruginous granular trichomes falling early; leaves with 1–3 pairs of pinnae, each pinna comprising a single pair of leaflets, petiolar nectary absent; fruit 4–7 × 1–1.5 cm; seeds white	***Microlobius***
–	Branches and leaves without evident garlic smell; ferruginous granular trichomes always present in brach tips; leaves always with more than one pair of pinnae, each pinna comprising 3 or more pairs of leaflets, petiolar nectary present; fruit 8–14 × 2–3.5 cm; seeds black, brown or ochre	**46**
46	Leaves with 2–4 (6) pairs of pinnae; leaflets 2.5–16 × 1.5–8 cm; spikes clustered in panicles (except in *G.coriacea* and *G.fissurata*)	***Gwilymia***
–	Leaves with (3) 5–32 pairs of pinnae; leaflets 0.6–1.2 × 0.3–0.6 cm; spikes grouped in terminal pseudoracemes or in fascicles below the leaves	**47**
47	Branches not striate; petiolar nectary 0.5–2 mm long; leaflets alternate, abaxial surface with a tuft of trichomes at the base of the midrib; petals fused at least for ½ of their length; fruit coriaceous or woody and indehiscent or splitting along a single margin (follicle)	***Stryphnodendron***
–	Branches strongly striate; petiolar nectary ca. 10 mm long; leaflets opposite, without a tuft of trichomes on the abaxial surface; petals fused only for ¹⁄3 of their length; fruit chartaceous, dehiscent along both margins (legume)	***Naiadendron***
48	Flowers of the base of the spikes sterile and with flat and showy staminodes	**49**
–	Flowers all fertile (hermaphrodite or staminate), or if sterile, then lacking showy staminodes	**50**
49	Short shoots terminated by spines or composed of many persistent fused stipules; fruits with sutural ribs not greatly enlarged, either elastically dehiscent from the apex and coiling after dehiscence with the pericarp coriaceous, or indehiscent and with the pericarp woody	***Dichrostachys***
–	Short shoots without terminal spines or fused stipules; fruits with sutural ribs greatly thickened or modified into flattened wings, either elastically dehiscent from the apex and recurved but not coiled after dehiscence, with the pericarp woody, or indehiscent or tardily inertly dehiscent and with the pericarp coriaceous to chartaceous	***Gagnebina***
50	Leaflets clearly alternate	**51**
–	Leaflets opposite or subopposite	**55**
51	Fruit linear, curved or spirally twisted, dehiscing into 2 coriaceous or subcoriaceous valves; seeds bulky with a hard bright red or red and black testa, unwinged; India to tropical South East Asia, Malesia, Australia and Madagascar (1 species widely cultivated)	***Adenanthera***
–	Fruit oblong to linear, indehiscent or dehiscent through both margins but then not spirally twisted, valves woody; seeds plano-compressed, winged or not, if bulky, then not red nor red and black; continental Africa	**52**
52	Fruit indehiscent, with a longitudinal crest or wing along the valves; flowers in spiciform racemes; seeds unwinged	**53**
–	Fruit dehiscent through both margins, lacking longitudinal wings; flowers in spikes; seeds winged	**54**
53	Anther-glands absent; fruit bluntly tetragonal or sub-cylindrical in cross section; pedicels and calyces glabrous; bracts not apparent	***Amblygonocarpus***
–	Anther-glands present, caducous; fruit with a thick longitudinal wing-like projection along each valve, thus cruciform in cross section; pedicels and calyces hairy; flowers subtended by persistent triangular bracts	***Tetrapleura***
54	Leaves lacking extrafloral nectaries; fruits oblong, 10–20 cm wide, dehiscent along both margins, with detaching endocarp; free-standing intrastaminal disk massive, clearly visible in open flower; petals lacking stellate trichomes; stamen filaments pubescent to near the middle	***Fillaeopsis***
–	Leaves with small sunken extrafloral nectaries on petiole and/or leaf-rachis; fruits linear-oblong, under 5 cm wide, dehiscing along only the ventral margin, the endocarp not detaching; intrastaminal disk present but not massive; petals stellate-pubescent; stamen filaments glabrous	***Cylicodiscus***
55	Flowers with 5 fertile stamens and 5–15 staminodes	* ***Pentaclethra***
–	Flowers with all 10 stamens fertile, lacking staminodes	**56**
56	Leaves lacking extrafloral nectaries	**57**
–	Leaves with extrafloral nectary on petiole and/or leaf-rachis	**59**
57	Flowers greenish; anther-glands absent; fruit laterally compressed, papyraceous, indehiscent, oblong, wind dispersed, twisted in its proximal end	***Aubrevillea***
–	Flowers red or bright yellow; anther-glands present; fruit dehiscent through one or both sutures with a woody or coriaceous pericarp	**58**
58	Flowers bright yellow; fruit woody, dehiscing elastically from apex downwards into two recurving valves; seeds unwinged; lianas with tendrilled leaves or small trees	***Pseudoprosopis***
–	Flowers red; fruit coriaceous, dehiscing along one margin only; seeds winged; big trees from rainforest canopy	***Piptadeniastrum***
59	Plants armed with internodal prickles	**60**
–	Plants unarmed	**61**
60	Fruit indehiscent, cylindrical or subterete, with a pulpy or fibrous mesocarp; largest leaﬂets < 1.5 × 1 cm; shoots with scattered prickles	***Prosopis***
–	Fruit dehiscent, plano-compressed, coriaceous, lacking a thick mesocarp; largest leaﬂets > 3 × 3 cm; shoots largely unarmed, occasionally with scattered prickles, mature stems with spine-tipped woody protuberances	***Indopiptadenia***
61	Fruits indehiscent with a thick spongy mesocarp; endocarp fragmented into 1-seeded chambers	***Anonychium***
–	Fruits dehiscing down one or both margins, woody, with a thin mesocarp; endocarp not fragmented	**62**
62	Seeds unwinged; fruit clavate to dolabriform, explosively dehiscent through both margins	***Calpocalyx***
–	Seeds winged; fruit dehiscing through one or both margins but passively so	**63**
63	Fruits opening through one margin only; flowers lacking an intrastaminal disk	***Newtonia***
–	Fruits opening through both margins; flowers with a distinct intrastaminal disk	***Lemurodendron***
﻿64	Stamens free or joined only at the very base	**65**
–	Stamens united into a staminal tube	**73**
65	Plants armed with stipular spines or prickles on stem and often also on the leaf petiole and rachis (spines or prickles may be absent or rare on individual specimens)	**66**
–	Plants unarmed	**68**
66	Plants armed with infranodal and/or internodal prickles; habit sometimes lianescent; peduncles lacking an obvious involucel (sometimes a small, caducous bract present)	***Senegalia***
–	Plants lacking prickles, armed with stipular spines, these sometimes swollen and hollow, and always restricted to the nodal regions; habit never lianescent; peduncles usually with an involucel, rarely lacking (in the monospecific *Faidherbia*)	**67**
67	Peduncles lacking an involucel; stamens united into a tube for ca. 1 mm, anthers eglandular	***Faidherbia***
–	Peduncles with involucel (usually medial or basal, rarely at base of inflorescence); stamens free to the base or occasionally shortly fused; anther glands often present	***Vachellia***
68	Funicle expanded into an aril at the point of attachment to the seed; plants native to Australia (but a few species cultivated, and sometimes invasive elsewhere)	* ***Acacia***
–	Seeds exarillate; plants from the Americas	**69**
69	Inflorescence a globose to sub-globose or ellipsoid capitulum (as broad as long or nearly so)	**70**
–	Inflorescence a cylindrical spike more than twice as long as wide	**71**
70	Leaves lacking extrafloral nectaries on petiole and rachis; stamens consistently 200+, the filaments often drying yellow, orange or pink	***Acaciella***
–	Leaves with extrafloral nectaries on petiole and/or rachis; stamens fewer than 150, the filaments drying straw-coloured	* ***Parasenegalia***
71	Twigs commonly with brachyblasts above most nodes; anther glands absent; endemic to Bolivia	***Pseudosenegalia***
–	Twigs lacking brachyblasts (except *Mariosousacompacta*, Mexico); anther glands present; widespread in the Americas	**72**
72	Fruit valves coriaceous; lenticels on twigs orange, orbicular to slightly elongated vertically, some more than 0.6 mm across	* ***Parasenegalia***
–	Fruit valves thinner, mostly chartaceous to cartilaginous; lenticels on twigs not orange, orbicular to slightly elongated horizontally, very rarely 0.6 mm across	***Mariosousa***
73	Leaves lacking extrafloral nectaries	**74**
–	Leaves with extrafloral nectaries on the petiole, leaf rachis, or both	**76**
74	Inflorescence units in spherical capitula; fruit with membranous to coriaceous valves; pollen in 16-celled, discoid and acalymmate polyads	* ***Zapoteca***
–	Inflorescence units mostly obconical capitula, less frequently spherical capitula, umbels or pseudoracemes; fruit with mostly woody valves, rarely coriaceous; pollen in (7) 8 (10)-celled and flattened-ovoid polyads	**75**
75	Plants from the New World; pollen in 8-celled and calymmate polyads, the basal cell with a conspicuous sticky appendage	* ***Calliandra***
–	Plants from tropical Africa; pollen in (7) 8 (10)-celled and acalymmate polyads, the basal cell with an extremely reduced sticky appendage	***Afrocalliandra***
76	Plants from the New World	**77**
–	Plants from continental Africa, tropical Asia, Malesia and Australia	**111**
77	Stems armed at all or most nodes with spines or thorns	**78**
–	Stems unarmed	**85**
78	Stems armed with axillary thorns; perulate resting buds axillary to most leaves	* ***Chloroleucon***
–	Stems armed with stipular spines; buds not perulate, protected by the adaxial side of the petiole	**79**
79	Petiolar nectary below the first pair of pinnae	**80**
–	Petiolar nectary at the point of origin of the first (or only) pair of pinnae	**82**
80	Trees or shrubs, generally sarmentose; flower buds flask-shaped, flowers opening at night; androecium up to 9 cm long	***Sphinga***
–	Erect trees or shrubs, never sarmentose; flower buds ovoid-pyriform, flowers opening during the day; androecium usually less than 3 cm long, very rarely up to 7 cm long	**81**
81	Calyx shallowly campanulate, 1–2 mm long; corolla lobes recurved at anthesis; ovary disk absent	***Havardia***
–	Calyx deeply campanulate, 2.8–3.4 mm long; corolla lobes erect at anthesis; ovary disk present (sometimes poorly developed)	***Gretheria***
82	Leaves with one pair of pinnae, leaflets 1 pair per pinna, if leaves with more pinnae and/or leaflets then seeds with fleshy, often brightly coloured arils	***Pithecellobium***
–	Leaves with 2 or more pairs of pinnae, never 1, leaflets 2–30 pairs per pinna; seeds lacking aril	**83**
83	Fruit cylindrical, woody, straight or slightly curved, deeply internally septate; seeds globose; growing in lowlands of Mexico and USA (Texas)	***Ebenopsis***
–	Fruit flattened or slightly subterete, subwoody and curved, without internal septa; seeds lentiform; growing in highlands of Mexico	**84**
84	Leaves with 1–2 pairs of pinnae; leaflets 3–12 per pinna, blades suborbicular, broadly oblong, or elliptic (then revolute); corolla lobes ascending	***Painteria***
–	Leaves with 3–7 pairs of pinnae; leaflets 10–20 per pinna, blades narrowly oblong, linear-oblong or lanceolate; corolla lobes recurving	***Ricoa***
85	Inflorescences cauliflorous, the spikes or capitula arising from efoliate nodes below the expanded leaves	**86**
–	Inflorescence units mostly axillary or clustered in efoliate pseudoracemes or panicles, sometimes on efoliate nodes of young stems	**87**
86	Calyx 14–20 mm long; bracts usually with patelliform nectaries, rarely lacking nectaries	* ***Macrosamanea***
–	Calyx up to a maximum 14 mm long, usually much shorter; bracts lacking patelliform nectaries	* ***Zygia***
87	Terminal and axillary resting buds ovoid, composed of closely imbricate, striately veined scales; flowers sessile in globose capitula	**88**
–	Resting buds absent or, if rarely present, the scales not striately veined, or loosely imbricate; flowers sessile or not, in spikes, racemes, capitula or umbels	**91**
88	Capitula grouped in efoliate pseudoracemes or panicles; fruit plano-compressed, tardily dehiscent with papery valves	***Blanchetiodendron***
–	Capitula solitary or fasciculate in leaf axils or at efoliate nodes below the expanding leaves; fruit dehiscent or indehiscent with thick or papery valves	**89**
89	Branches with brachyblasts; flowers in each capitulum dimorphic; fruits with firm valves, compressed but somewhat plump	* ***Chloroleucon***
–	Branches lacking brachyblasts; flowers homomorphic; fruits with stiff paper valves, plano-compressed	**90**
90	Lower leaflet surface with evident reticulate secondary and tertiary venation; eastern and central Brazil	***Leucochloron***
–	Lower leaflet surfaces with 1–2 (3) prominent primary veins, but otherwise the venation not evident; endemic to eastern Andean valleys in Bolivia	***Boliviadendron***
91	Leaflet venation palmate or the leaflets very narrow and 1-veined	**92**
–	Leaflet venation pinnate	**95**
92	Fruits indehiscent, somewhat woody and ear-shaped (reniform-auriculiform), internally septate into 1-seeded chambers	**93**
–	Fruits dehiscent through both margins or, if indehiscent, not ear-shaped, not internally septate into 1-seeded chambers	**94**
93	Indumentum of leaf rachis ferruginous; leaflets in 40–80 pairs per pinna, very small (2.5–6 mm long) and 1-veined; sepals and petals ferruginous-villous externally; ovary tomentose	***Robrichia***
–	Indumentum of leaf rachis pallid; leaflets either fewer pairs per pinna and/or larger, palmately-veined; sepals and petals puberulent, rarely sericeous, strigose-pilose, or glabrous; ovary at anthesis glabrous	***Enterolobium***
94	Flowers homomorphic, larger (corolla 11–63 mm long, androecium 30–70 mm long); seeds papery, lacking a pleurogram	* ***Macrosamanea***
–	Flowers dimorphic, smaller (corolla <6.5 mm long, androecium <20 mm long); seeds with a hard coat, presenting a U-shaped or O-shaped pleurogram	***Pseudalbizzia***
95	Capitula clustered into efoliate terminal panicles, the inflorescence units never subtended by a leaf; fruit a long plano-compressed papery loment (20–70 cm long), the articles twisted through ±90° at each isthmus	***Cedrelinga***
–	Capitula solitary, fasciculate in leaf axils, or clustered in axillary efoliate pseudoracemes, or the pseudoracemes at efoliate nodes below the leaves; fruits dehiscent or indehiscent but if indehiscent never with twisting articles	**96**
96	Fruits indehiscent or breaking into 1-seeded articles	**97**
–	Fruits dehiscent through one or both margins	**100**
97	Fruits with a strongly biconvex or sub-cylindrical body, the valves woody or pulpy and internally at least partially septate; flowers heteromorphic in each capitulum	**98**
–	Fruits mostly plano-compressed with papery, leathery or subligneous valves; flowers either homomorphic or heteromorphic	**99**
98	Androecium bicoloured, the stamens basally white and distally pink or reddish	***Samanea***
–	Androecium uniformly whitish	* ***Hydrochorea***
99	Fruits lomentiform, the ripe leathery or subligneous valves breaking into 1-seeded articles but leaving no persistent margins; flowers heteromorphic	* ***Hydrochorea***
–	Fruits indehiscent or breaking into 1-seeded articles leaving a persistent margin (craspedium); flowers homomorphic	***Lysiloma***
100	Flowers within each capitulum dimorphic	**101**
–	Flowers homomorphic	**103**
101	Fruit curved to spiral, chartaceous, plano-compressed, twisting at dehiscence to expose the reddish-orange endocarp	* ***Jupunba***
–	Fruit straight or slightly arched, the papery or woody valves not becoming spirally twisted during dehiscence	**102**
102	Fruit linear, the margins slender, sometimes immersed, the valves papery, barely dehiscent through one or both sutures	***Pseudosamanea***
–	Fruit oblong with raised margins framing plane and woody valves, late dehiscent along one suture (follicle)	* ***Hydrochorea***
103	Fruits elastically dehiscent from the apex, the chartaceous to weakly coriaceous valves becoming arched backwards; small erect or scandent shrubs	* ***Zapoteca***
–	Fruits dehiscent through one or both margins but the valves never recurving and becoming arched backwards; trees	**104**
104	Fruit falcately or spirally recurved, the valves compressed but elevated over each seed, twisting at dehiscence to reveal the reddish endocarp; seeds bicoloured (white and dark bluish) or with a translucent testa	105
–	Fruit compressed with flat valves, if cylindrical and torulose with reddish valves then with black or dark brown seeds	**107**
105	Inflorescence a dense globose capitulum; flowers sessile; fruits with a puberulent and ferruginous epicarp; seeds with a U-shaped median pleurogram	***Abarema***
–	Inflorescence in lax elongated or congested racemes or elongated spikes; flowers sessile to pedicellate; fruits without a puberulent epicarp; seeds with an apical-basal pleurogram or the pleurogram absent	**106**
106	Leaflets obovate, ovate, oblong, linear or rhombic, secondary veins not arched; inflorescences lax or congested racemes, rarely spikes but then in combination with 2–12 pairs of leaflets per pinna; pleurogram usually present	* ***Jupunba***
–	Leaflets elliptic to ovate-lanceolate, the secondary veins arched; inflorescences long-racemose or spiciform; pleurogram absent	***Punjuba***
107	Bracts with nectaries on the upper surface	* ***Macrosamanea***
–	Bracts lacking nectaries	**108**
108	Corolla slender tubular with very short teeth; fruits cylindrical, torulose or moniliform, the ripe valves externally red and coriaceous	* ***Cojoba***
–	Corolla campanulate, the teeth at least ¼ of the total length; fruits not as above	**109**
109	Leaves unijugate, each pinna with just one leaflet	* ***Zygia***
–	Leaves either with more pinnae or leaflets	**110**
110	Fruit with inconspicuously raised margins, the valves linear, plano-compressed, stiffly papery, inertly dehiscent through both sutures; capitula solitary or fasciculate in leaf axils; flowers homomorphic and sessile; tropical Mexico	***Hesperalbizia***
–	Fruit with markedly raised margins framing an oblong body, the valves plane and woody and tardily dehiscing through the upper margin only (follicle); capitula clustered in pseudoracemes; flowers heteromorphic, the peripheral ones pedicellate; South America	* ***Hydrochorea***
111	Fruit elastically dehiscent from apex downwards, the stiffly coriaceous valves becoming arched backwards	**112**
–	Fruits dehiscent through one or both margins or indehiscent or lomentiform, if dehiscent through both margins the valves never become arched backwards	**113**
112	Branches unarmed; Madagascar	***Viguieranthus***
–	Branches armed with stipular spines; north-east India and adjacent Bangladesh and Myanmar	* ***Calliandra***
113	Branches armed with stipular spines	**114**
–	Branches unarmed or with infranodal prickles	**115**
114	Capitula with homomorphic flowers; fruit submoniliform with leathery valves; Sri Lanka to Vietnam	***Thailentadopsis***
–	Capitula usually with heteromorphic flowers; fruit plano-compressed with papery valves; Malesia	* ***Albizia***
115	Leaflets alternate	**116**
–	Leaflets opposite or subopposite	**117**
116	Inflorescences spikes, racemes or capitula, these clustered in pseudoracemes, panicles or umbels; fruits woody, straight, indehiscent or late dehiscent	***Serianthes***
–	Inflorescences corymbiform umbels, solitary or fasciculate in leaf axils or on short-shoots; fruits chartaceous, dehiscing along one margin only, contorted to expose the reddish endocarp	***Pararchidendron***
117	Plants from Africa	**118**
–	Plants from Asia, Australia and the Pacific	**120**
118	Fruits with papery valves, usually dehiscent, non-septate	* ***Albizia***
–	Fruits septate, the valves thin and fibrous or thick and woody, indehiscent or lomentiform	**119**
119	Pedicels of peripheral flowers at least 1 mm long; leaves with (1) 2–3 pairs of pinnae; fruits lomentiform with seeds dispersed as 1-seeded articles	* ***Hydrochorea***
–	Pedicels of peripheral flowers up to 0.5 mm long; leaves with (3) 5–35 pairs of pinnae; fruits indehiscent or if lomentiform only tardily breaking up into articles	***Osodendron***
120	Seeds lacking a pleurogram; gynoecium uni- or pluricarpellate	**121**
–	Seeds with a pleurogram; gynoecium unicarpellate	**123**
121	Fruits dehiscing through one or both margins, spirally contorted, if straight then turgid, usually with the valves reddish outside and orangish-red within, sharply contrasting with the seeds; seeds black or bluish-black, unwinged	***Archidendron***
–	Fruits dehiscing through both margins, straight and plano-compressed, the valves not reddish inside; seeds strongly flattened with a narrow marginal wing	**122**
122	Inflorescence units capitula; calyx and corolla pubescent	***Heliodendron***
–	Inflorescence units spikes, spiciform racemes, racemes, rarely capitula but then with calyx and corolla glabrous	***Archidendropsis***
123	Inflorescence units capitula	* ***Albizia***
–	Inflorescence units racemes, spikes or spiciform racemes	**124**
124	Fruit plano-compressed with woody valves, the endocarp breaking into 1-seeded articles; flowers in lax axillary racemes	***Wallaceodendron***
–	Fruit with papery valves, the endocarp not breaking into 1-seeded articles; flowers in dense racemes or the racemes grouped in panicles	**125**
125	Racemes solitary or paired in leaf axils	***Paraserianthes***
–	Racemes or spikes clustered in large panicles	***Falcataria***
﻿126	Lowermost sepal modified and boat-shaped, usually making a hood in bud	**127**
–	Lowermost sepal not differentiated from the remaining ones	**152** (page 48)
127	Leaves terminating in a pair of pinnae plus a single terminal pinna	**128**
–	Leaves terminating in a pair of pinnae or with pinnae clearly alternate	**135**
128	Plant armed with scattered straight conical spines, and short, lateral spinescent shoots; fruits oblong to fusiform, glabrous, dehiscing along the middle of the valves or parallel to the margin	* ***Haematoxylum***
–	Plant unarmed; fruits not dehiscing along the middle of the valves	**129**
129	Sepals persistent in fruit	***Hoffmannseggia***
–	Sepals caducous in fruit	**130**
130	Fruits cylindrical-torulose; central and western Argentina, in subtropical wooded grassland and scrub, especially on salt flats	***Stenodrepanum***
–	Fruits never cylindrical-torulose, widespread in the Americas and South Africa	**131**
131	Stipules linear, persistent; androecium and gynoecium cupped in the lower cucullate sepal; lower lateral sepals forming a platform at right angles to the abaxial cucullate sepal; fruits with simple trichomes, glandular-punctate trichomes, and plumose, dendritic and/or stellate trichomes	***Pomaria***
–	Stipules caducous; androecium and gynoecium not cupped in the lower sepal, deflexed; lateral sepals not forming a platform; fruits glabrous or with simple and/or gland-tipped trichomes, the latter sometimes also dendritic or plumose	**132**
132	Fruits indehiscent, thickened and somewhat torulose; inflorescence a raceme or panicle, often corymbose; leaflets glabrescent and eglandular, or with glandular dots parallel to the midvein	* ***Libidibia***
–	Fruits dehiscent, often with twisting valves; inflorescence a raceme or panicle, sometimes pyramidal in shape; leaflets glabrescent to densely pubescent or with a stellate indumentum; leaflets eglandular, or with dark subepidermal glands, and/or with glandular dots sunken in the margins or parallel to the margin on the abaxial side of the leaflets	**133**
133	Leaflets alternate, or occasionally sub-opposite (rarely fully opposite), with dark subepidermal glands (best seen with a ×10 hand lens); stellate indumentum sometimes present on foliage and inflorescence rachis; fruit subligneous to woody, with thickened sutures	* ***Cenostigma***
–	Leaflets always opposite, without dark subepidermal glands; stellate indumentum never present on foliage or rachis; fruit coriaceous to subligneous, sutures not thickened	**134**
134	Shrubs or small to medium-sized trees varying from (0.5) 1–12 (20) m tall, occasionally functionally herbaceous subshrubs, woody at the base; flowers sometimes laterally compressed; petals yellow, red, pink or orange; ovary eglandular or covered in gland-tipped trichomes, the hairs never dendritic; widespread across low-elevation seasonally dry tropical forests and woodlands in Mexico, Central America, the Caribbean, and in Caatinga vegetation in Brazil, and in patches of dry forest, deserts, yungas-puna transition zones, and chaco-transition forests in Argentina, Bolivia, Chile and Paraguay	***Erythrostemon***
–	Small to medium-sized, often decumbent shrubs, 0.3–2.5 m tall; flowers never laterally compressed; petals yellow, sometimes all five petals streaked with red markings; ovary covered in gland-tipped trichomes, which are sometimes dendritic; occurring at mid elevations in dry inter-Andean valleys, in Ecuador, Peru, Bolivia and Argentina	***Arquita***
135	Plants unarmed	**136**
–	Plants armed	**139**
136	Fruit thin, plano-compressed, oblong-elliptic to elliptic, valves membranous to papyraceous, indehiscent; margin of the lower cucullate sepal pectinate-glandular; flowers unisexual; leaflets eglandular	***Coulteria***
–	Fruit oblong-elliptic, elastically dehiscent with twisting valves; margin of the lower cucullate sepal entire; flowers bisexual; leaflets eglandular or with red glands	**137**
137	Flowers nearly actinomorphic; trees, up to 25 m tall; leaflets eglandular or with red glands; eastern Africa (Kenya and Tanzania), and northern and north-western Madagascar	* ***Stuhlmannia***
–	Flowers clearly zygomorphic; shrubs or small trees, up to 5 m tall; leaflets eglandular; Cuba or northern Madagascar	**138**
138	Fruits laterally compressed; anthers glabrous; endemic to Cuba (near Moa, in the Sierra de Nipe)	* ***Caesalpinia***
–	Fruits inflated and hollow; anthers pubescent; endemic to the northern tip of Madagascar (Orangea peninsula, near Antsiranana)	* ***Denisophytum***
139	Trees or erect shrubs	**140**
–	Lianas or climbing or trailing shrubs	**144**
140	Fruits indehiscent, somewhat fleshy, turgid and coriaceous; lower cucullate sepal with a pectinate/fimbriate or entire margin	***Tara***
–	Fruits dehiscent, with valves twisting upon dehiscence, laterally-compressed and subligneous to woody; lower cucullate sepal with an entire margin	**141**
141	Fruits armed with woody prickles; stems with upturned thorns arising from woody protuberances; petals yellow, the standard with a conspicuous red blotch on the inner face	***Paubrasilia***
–	Fruits unarmed; stems with straight to deflexed prickles; petals yellow, white, pink, red or orange	**142**
142	Petals pink-purple to whitish pink; bracts broadly ovate to suborbicular with an aristate apex; fruits pyriform with rounded, oblique bases; leaflets sometimes with translucent dots on lower surface	***Gelrebia***
–	Petals yellow, red, orange or white; bracts lanceolate to linear with an acute to acuminate apex; fruits oblong-elliptic, short-stipitate, with cuneate base; leaflets eglandular	**143**
143	Petals orange, red, or white; Central America, Mexico, the Caribbean and the northern Andes (Peru to Colombia)	* ***Caesalpinia***
–	Petals yellow, sometimes with red markings on the standard (median petal); Somalia, Ethiopia, Argentina, Paraguay, Mexico, Florida and the Caribbean	* ***Denisophytum***
144	Fruits winged, although wing sometimes very narrow	**145**
–	Fruits without a wing	**148**
145	Fruit a samara with a basal 1-seeded chamber and a prolonged upper suture that is broadly winged	***Pterolobium***
–	Fruit 1 or more seeded, with a longitudinal (often narrow) wing along the upper suture, if 1-seeded then with a central seed-chamber	**146**
146	Fruit with a prominent wing 2 mm or more wide	***Mezoneuron***
–	Fruit with a less distinct wing, usually 2 mm wide or less (although occasionally up to 4 mm, or carinate)	**147**
147	Fruit oblong-elliptic, dehiscent, terminating in a sharp beak, 4–9-seeded	* ***Biancaea***
–	Fruit circular to sub-elliptic or lunate, indehiscent, 1 (rarely 2)-seeded	* ***Ticanto***
148	Plants with glands on stems, leaf rachis, inflorescence, and fruits; needle-like trichomes on inflorescence rachis and pedicels	***Hultholia***
–	Plants eglandular; stems with recurved prickles; pedicels and inflorescence peduncles with a few prickles near their bases	**149**
149	Fruit oblong to oblong-elliptic	**150**
–	Fruit broadly elliptic to circular	**151**
150	Fruit oblong, indehiscent, somewhat fleshy, sub-torulose, with thickened sutures, terminating in an acute apex, exocarp and endocarp strongly adnate; seeds sub-globose	***Moullava***
–	Fruit oblong to oblong-elliptic, laterally compressed, dehiscent, coriaceous to subligneous, with a smooth, regular outer surface, base often much narrower than the truncate apex which terminates in a sharp beak, exocarp and endocarp separate easily; seeds flattened to ellipsoidal	* ***Biancaea***
151	Flowers unisexual, segregated into pistillate and staminate racemes; fruits usually covered in spinescent bristles; seeds globose, with parallel fracture lines concentric with the small apical hilum	***Guilandina***
–	Flowers bisexual, in racemes; fruits not spinescent; seeds laterally compressed, smooth, without fracture lines	* ***Ticanto***
﻿152	Plants from the Americas	**153**
–	Plants from Africa, Asia and Australia	**165**
153	Androecium strongly dimorphic including fertile stamens and staminodes	**154**
–	Androecium homomorphic with all stamens fertile	**155**
154	Indumentum of rusty T-shaped trichomes; stipules pinnate; flowers showy with bright yellow clawed petals; fertile stamen 1, this clearly longer than the 9 staminodes	* ***Moldenhawera***
–	Indumentum lacking T-shaped trichomes; stipules linear or absent; flowers small, pale yellow to cream or dark orange to reddish, petals not clawed; fertile stamens 5; staminodes 5, spatulate, free or connate forming a dome	***Dimorphandra***
155	Stamens united basally into a tube deeply split on one side rendering the flowers slightly bilateral; fruit valves becoming recurved backwards upon dehiscence; stipules pinnate	***Jacqueshuberia***
–	Stamens free; fruits indehiscent or passively dehiscent, rarely with the valves becoming longitudinally twisted; stipules entire (not pinnate)	**156**
156	Inflorescence units spikes with densely packed flowers	**157**
–	Inflorescence units racemes or panicles	**159**
157	Sepals united into a gamosepalous calyx; fruits flat, coriaceous, indehiscent and marginally compressed or woody, elastically dehiscent along both sutures; South America in Amazonia and eastern Brazil	***Dinizia***
–	Sepals free; fruit thick-walled, indehiscent, subterete or flat but then with margins not compressed	**158**
158	Fruit compressed with a linear outline; plants from temperate North and South America	* ***Gleditsia***
–	Fruit sub-terete; plants from Hispaniola (Haiti and Dominican Republic)	***Arcoa***
159	Sepals and petals similar, greenish or whitish, the sepals not covering petals in bud	**160**
–	Sepals and petals clearly differentiated, the petals larger than sepals and yellow; petals covering petals in bud	**161**
160	Hypanthium elongate, sub-cylindrical; leaflet margins entire; fruit sessile	*Gymnocladus
–	Hypanthium campanulate, discoid or turbinate; leaflet margins crenulate, rarely entire; fruit stipitate	* ***Gleditisia***
161	Plants usually armed with spines or thorns, rarely unarmed; adaxial petal clearly differentiated in shape and/or colour, with a thicker claw	* ***Parkinsonia***
–	Plants unarmed; all petals more or less equal	**162**
162	Fruits dehiscent through both sutures	**163**
–	Fruits indehiscent	**164**
163	Fruit 1-seeded, flattened, spatulate, oblanceolate or spoon-shaped, the valves firm-coriaceous, with the endocarp released as a thin, papery wing-like envelope; rain forests of southern Mexico to South America	***Schizolobium***
–	Fruit (2) 3 (4)-seeded, plano-compressed, linear, the margins narrowly winged; dry forests of western and southern Mexico from Sonora and Baja California Sur south to Chiapas	***Conzattia***
164	Fruit flat, oblong or narrowly ellipsoid, the mesocarp thin and dry, tapering at both ends, with a firm wing-like extension on each suture, valves usually longitudinally striate; flowers bisexual; stigma peltate	* ***Peltophorum***
–	Fruit cylindrical, the mesocarp thickened and fibrous, valves smooth, pale orange-brown when ripe, with fibrous-spongy septae between the seeds forming marked seed cavities; flowers unisexual by reduction of the androecium or gynoecium, dioecious; stigma circular, flat or slightly concave	***Heteroflorum***
165	Flowers apetalous with a fleshy hypogynous disk wider than the calyx	* ***Ceratonia***
–	Flowers with sepals and petals	**166**
166	Flowers unisexual and tetramerous, with 4 sepals, 4 petals and 8 stamens (staminate flowers); fruit indehiscent with 4 membranous wings; Madagascar	***Tetrapterocarpon***
–	Flowers bisexual or unisexual but then with 5 (6) sepals and petals; fruits unwinged or with just two straight wings along the sutures, indehiscent or dehiscent along one or both margins	**167**
167	Sepals and petals very similar, greenish or lavender coloured, the narrow sepals not covering petals in bud	**168**
–	Sepals and petals clearly differentiated	**169**
168	Unarmed trees; hypanthium elongate, sub-cylindrical, 6–12 mm long; leaflet margins entire; fruit sessile	* ***Gymnocladus***
–	Trees usually armed with thorns and/or branched spines; hypanthium shortly campanulate, 1–4 mm long; leaflet margins crenulate, rarely entire; fruit stipitate	* ***Gleditsia***
169	Sepals valvate in bud	**170**
–	Sepals imbricate in bud	**171**
170	Flowers radial or weakly zygomorphic; all sepals free and reflexed at anthesis; petals showy and long-clawed	* ***Delonix***
–	Flowers strongly zygomorphic and resupinate; 4 sepals united into a lip making a spathaceous calyx; petals reduced	***Colvillea***
171	Adaxial petal differentiated in shape, colour or both, with a thicker claw; plants mostly armed with stipular spines, axillary thorns or spinescent leaf rachides, rarely unarmed	* ***Parkinsonia***
–	All petals similar, if clawed the claws equally thin; unarmed trees or shrubs	**172**
172	Flowers pedicellate in lax racemes; petals bright yellow, clawed, with wrinkled margins; style terminating in a peltate stigma	**173**
–	Flowers sessile or shortly pedicellate in dense spikes or spiciform racemes; petals white or greenish white, not clawed and/or with entire margins; style short and stigma porate	**174**
173	Fruit indehiscent, flat, oblong or narrowly ellipsoid, tapering at both ends, with a wing-like extension on each suture	* ***Peltophorum***
–	Fruit elastically dehiscent from the apex, narrowly oblong-obovate, compres­sed with greatly thickened margins, the valves woody, recurving	***Bussea***
174	Leaflets alternate	**175**
–	Leaflets opposite	**177**
175	Fruit 1 (2)-seeded, elliptical to oblong-elliptical, flat, thin-valved, indehiscent; petals reflexed at anthesis	***Burkea***
–	Fruit 2–15-seeded, linear or oblong-linear, valves stiffly coriaceous or woody, indehiscent or late dehiscent; petals erect at anthesis	**176**
176	Ovary long-stipitate; petals green to greenish-yellow; fruits not septate, with thin woody or leathery valves and margins not thickened	***Erythrophleum***
–	Ovary subsessile; petals red; fruits with woody resinous valves and thick, raised margins, the endocarp internally septate into 10–15 1-seeded envelopes	***Pachyelasma***
177	Petals basally united into a gamopetalous corolla; leaves terminating in a pair of pinnae	* ***Sympetalandra***
–	Petals free; leaves terminating in a solitary pinna	**178**
178	Stamens dimorphic, the 5 antepetalous shorter and with thin filaments, the 5 antesepalous longer with filaments wider towards the apex; flowers with sepals, petals and stamens whitish, in cylindrical erect spikes; West Africa	***Stachyothyrsus***
–	Stamens homomorphic, all with filiform filaments; flowers with showy red hypanthium and bright orange stamens in dense drooping racemes, the pedicels twisting so that all flowers face upwards; tropical Asia	***Acrocarpus***
﻿179	Petals valvate in bud; petals joined into a gamopetalous corolla; seeds with a U-shaped pleurogram	**180**
–	Petals imbricate in bud, the uppermost petal in inner position; petals free; seeds lacking a pleurogram	**185**
180	Leaves lacking nectaries	**181**
–	Leaf rachis with nectaries between the leaflets	**182**
181	Plants armed with triangular broad-based recurved prickles on the shoots; leaves densely fascicled on cushion-like brachyblasts, petiole flat and laterally expanded, leaflets small (0.2–0.6 cm long); fruit a craspedium with undulate margins, persisting after the 1-seeded articles disperse; Mexico (Coahuila)	* ***Mimosa***
–	Plants unarmed; leaves not fascicled, with a grooved unexpanded petiole and large leaflets (4–10 cm long); fruits elastically dehiscent from the apex, the woody valves becoming recurved backwards after dehiscence; coastal plains of the three Guianas	* ***Calliandra***
182	Plants armed with straight horizontal stipular spines; fruits elastically dehiscent from the apex; India (western Ghats)	***Sanjappa***
–	Plants unarmed; fruits indehiscent or passively dehiscent; Americas	**183**
183	Inflorescences cauliflorous	* ***Zygia***
–	Inflorescences solitary or fasciculate in leaf axils or on efoliate terminal shoots	**184**
184	Fruits indehiscent; seeds surrounded by a whitish sweet sarcotesta	***Inga***
–	Fruits late dehiscent through both sutures, terete to moniliform with bright red valves; seeds lacking an aril or a sarcotesta	* ***Cojoba***
185	Lowermost sepal modified and boat-shaped, usually forming a hood in bud (cucullate)	**186**
–	Lowermost sepal not differentiated from the other four	**194**
186	Armed shrubs or trees, with prickles scattered along the branches or in pairs below the stipules, or plants with short shoots modified into persistent thorns	**187**
–	Unarmed shrubs or trees	**190**
﻿187	Sepals persistent in fruit; fruit cylindrical, covered with resinous hairs; pairs of needle-like prickles inserted below the stipules and leaf petiole; endemic to northern Chile, from the Coquibo and La Serena valleys	***Balsamocarpon***
–	Sepals caducous; fruit flattened, non-resinous; plants armed with scattered straight spines on shoots or with curved deflexed prickles; widely distributed across Central America, Mexico, the Caribbean, South America and Namibia	**188**
188	Fruit a lomentum, with 4 coarsely serrate wings, breaking up into one-seeded articles; native to Paraguay and northern Argentina	***Lophocarpinia***
–	Fruit unsegmented, without wings; native to Namibia and Mexico to northern South America	**189**
189	Fruit sub-circular to sickle-shaped, tardily dehiscent along the sutures, finely pubescent and with robust patent trichomes; endemic to Namibia	***Hererolandia***
–	Fruit oblong to fusiform, dehiscent along the middle of the fruit valves or close to the fruit margin, but never along the sutures, lacking patent trichomes	* ***Haematoxylum***
190	Sepals persistent; fruit indehiscent, gall-like, covered with long bristles; north-west Argentina	***Zuccagnia***
–	Sepals caducous; fruits ovoid to elliptic, not gall-like, glabrous or covered in a different type of indumentum	**191**
191	Fruit elastically dehiscent, with valves twisting upon dehiscence, laterally-compressed and subligneous to woody, oblanceolate to oblong-elliptic	**192**
–	Fruit indehiscent, thickened and fleshy, ovoid or elliptic	**193**
192	Fruit subligneous, lacking a crest; sepals valvate; stellate indumentum lacking; restricted to Africa and Madagascar	* ***Stuhlmannia***
–	Fruit woody, with conspicuously thickened sutures, sometimes with a crest proximally on the adaxial side; stellate indumentum often present; sepals imbricate; restricted to the Neotropics	* ***Cenostigma***
193	Fruit elliptic, somewhat thick and fleshy, bright red at maturity, rounded at apex and base, 1–2-seeded; leaflets with black, sessile glands on the lower surface; seeds compressed-turgid; sepals imbricate; endemic to Hispaniola and Puerto Rico	* ***Libidibia***
–	Fruit ovoid, with a ligneous and brownish pericarp, apex beaked, 1–4-seeded; leaflets with red glands on the lower surface; seeds ovoid; sepals valvate; endemic to north-eastern Africa	***Cordeauxia***
194	Leaves imparipinnate and/or with alternate leaflets	**195**
–	Leaves paripinnate with opposite leaflets	**202**
195	Armed trees, with simple or compound thorns on trunk and branches (sometimes thornless in cultivated plants); leaflet margins crenulate, rarely entire; temperate and subtropical North and South America, eastern Asia and Caspian region	* ***Gleditsia***
–	Unarmed trees or shrubs; leaflet margins entire; tropical South America, Mediterranean region and Arabian Peninsula	**196**
196	Plants polygamo-dioecious; flowers apetalous, with a conspicuous disk wider than the calyx; Mediterranean region of northern Africa and southern Europe to Arabian Peninsula	* ***Ceratonia***
–	Plants with flowers hermaphrodite, with petals and a tiny disk or the disk absent; tropical South America	**197**
197	Flowers pentamerous, with 5 sepals, 5 petals and 10 stamens; filaments whitish or pale yellow; fruits indehiscent or passively dehiscent; seeds various but never with a spongy wing	**198**
–	Flowers with different numbers of sepals and/or petals and/or stamens; stamens 10–17 (25) per flower, filaments dark red and showy; fruits dehiscent with the valves loosely spiralling; seeds discoid, with a spongy wing	***Campsiandra***
198	Flowers small (ca. 3 mm diam.), entirely white or greenish-white, in spicate, axillary, catkin-like, racemes; fruit a 1-seeded samara with a style remnant laterally displaced at apex of the seed chamber	***Pterogyne***
–	Flowers > 7 mm diam with yellow petals; flowers in terminal panicles, petals pale yellow or bright yellow; fruit dehiscing through one or both margins	**199**
199	Leaves with a nectary on the petiole or leaf rachis, between leaflets; fruits dehiscing only through one suture; flowers small (to 15 mm diam); petals pale yellow, obovate, not clawed	**200**
–	Leaves lacking nectaries; fruits indehiscent or dehiscent along both sutures; flowers larger (> 20 mm diam.); petals with bright yellow blades widely expanded from the claws	**201**
200	Ovary 1-ovulate; fruit 1-seeded, globose to pyriform, subligneous, late dehiscent, the surface not ribbed	***Vouacapoua***
–	Ovary with more than 1 ovule; fruit inflated, oblong, woody, longitudinally ribbed, with 2–3 red seeds	***Batesia***
201	Fruit linear, narrowly winged along the upper suture, the wing up to 3 mm wide, late dehiscent along both margins, the endocarp not septate	***Recordoxylon***
–	Fruit oblong, dehiscent along both margins to release winged 1-seeded envelopes resulting from endocarp fragmentation	***Melanoxylum***
202	Anthers of some stamens or staminodes dehiscing through pores	**203**
–	All anthers dehiscing through longitudinal slits	**206**
203	Androecium with just one fertile stamen and 7 or 9 staminodes, the fertile stamen much longer and equalling the length of the style, the anther with a villous connective; staminodes short, barely extending beyond the ovary length; stipules pinnately compound; indumentum of rusty T-shaped trichomes	* ***Moldenhawera***
–	Androecium of 7–10 fertile stamens, staminodes up to 3, usually much shorter than the fertile ones; stipule blades never compound; indumentum various but never including T-shaped trichomes	**204**
204	Pedicels with a pair of alternate bracteoles near the middle; fruits elastically dehiscent, the valves twisting after dehiscence; anthers with pubescent sutures; extrafloral nectaries, when present, with a concave or flat head	* ***Chamaecrista***
–	Bracteoles when present at the base of the pedicels; fruits indehiscent or passively dehiscent, valves remaining straight after dehiscence; anther sutures glabrous; extrafloral nectaries, when present, with a convex head	**205**
205	Foliar nectaries absent; hypanthium solid, turbinate or conical; 3 abaxial stamens with sigmoidal filaments and longitudinally dehiscent anthers; adaxial anthers with basal pores; fruits woody to coriaceous, indehiscent, commonly cylindrical or less often compressed, rarely fragmenting into 1-seeded segments	***Cassia***
–	Foliar nectaries often present on the petiole and/or leaf rachis; hypanthium absent; all stamen filaments straight or curved (but not sigmoidal); abaxial and median stamens with apical pores, the 3 adaxial stamens sterile (staminodes); fruits dehiscent with chartaceous or papery valves or indehiscent, with a flat compressed to cylindrical body	* ***Senna***
206	Plants polygamo-dioecious; flowers apetalous, with a conspicuous disk wider than the calyx	* ***Ceratonia***
–	Flowers hermaphrodite; flowers with 5 petals and a tiny disk or a disk absent	**207**
207	Plants from tropical Africa and Malesia	**208**
–	Plants from tropical America	**211**
208	Plants armed with thorny branches or spinescent shoots	**209**
–	Plants unarmed	**210**
209	Leaflets 2–3 pairs, symmetrical and petiolulate; flowers showy, zygomorphic, petals clawed and white but the upper petal slightly larger and with a yellow blotch; sepals valvate; Madagascar	* ***Delonix***
–	Leaflets 5 or more pairs per leaf, markedly asymmetrical, sessile; flowers small, greenish, actinomorphic; calyx open in bud, not covering the petals; South Africa	***Umtiza***
210	Flowers wine-red in loose pendulous panicles; petals and stamens free to the hypanthium rim; fruits elastically dehiscent, the valves twisting; West African rainforests	***Chidlowia***
–	Flowers white to greenish-white in dense spiciform racemes; petals and stamens joined at the base; fruits indehiscent, longitudinally striate; Malesia	* ***Sympetalandra***
211	Fruits samaroid, oblong-elliptical, tapered at each end, with a central seed-nucleus and a thin marginal wing, the exocarp flaking when ripe; hypanthium cupular, either symmetrical (the ovary attached in the middle of the hypanthium) or asymmetrical (ovary laterally attached)	***Tachigali***
–	Fruits dehiscent, oblong to linear with woody valves; hypanthium funnel-shaped or disk-shaped and symmetrical	**212**
212	Stipules foliaceous, suborbicular and coriaceous, persistent or late caducous; flowers > 2 cm long in terminal corymbose panicles; fruits elastically dehiscent from apex, the valves rolling up backwards	***Arapatiella***
–	Stipules membranous and caducous, lanceolate; flowers < 1 cm long, in axillary racemes or spikes	**213**
213	Flowers distinctly pedicellate in racemes; sepals and petals reflexed; all 10 stamens fertile; fruits with thin ligneous valves, passively dehiscent; seeds plano-compressed with a wide marginal wing	***Diptychandra***
–	Flowers sessile or subsessile in dense spikes or spiciform racemes grouped in woody terminal panicles; sepals and petals erect or spreading; androecium of 5 fertile stamens alternating with 5 staminodes; fruits woody, the valves twisting after dehiscence; seeds large (mostly > 10 cm long and weighing about 1 kg), bulky, unwinged	***Mora***

### ﻿Glossary

﻿Illustrated glossary (Schemes 1–7) of morphological terms used in the key and in the descriptions of Caesalpinioideae.

## ﻿﻿2. Tribe Ceratonieae

Gwilym P. Lewis^10^

Citation: Lewis GP (2024) 2. Tribe Ceratonieae. In: Bruneau A, Queiroz LP, Ringelberg JJ (Eds) Advances in Legume Systematics 14. Classification of Caesalpinioideae. Part 2: Higher-level classification. PhytoKeys 240: 62–69. https://doi.org/10.3897/phytokeys.240.101716

### ﻿﻿Tribe Ceratonieae

Taxon classificationPlantaeFabalesFabaceae

Rchb., Fl. Germ. Excurs. 2(2): 544. 1832.

**Figure 6.**
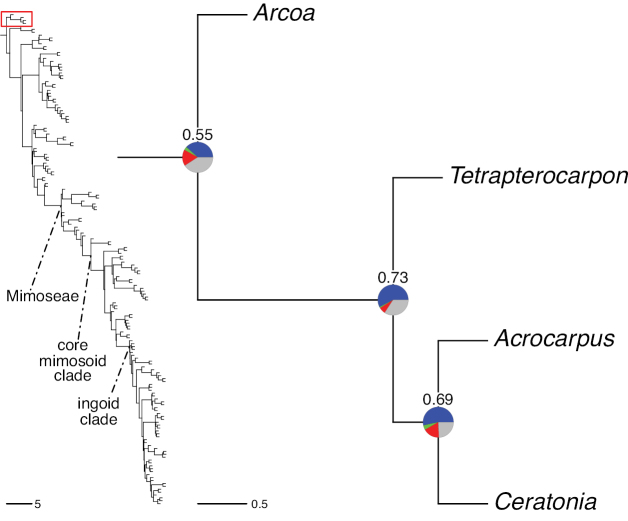
Generic relationships in tribe Ceratonieae. Left part of figure shows complete genus-level Caesalpinioideae phylogeny with the Ceratonieae indicated with a red rectangle. Branch lengths are expressed in coalescent units and terminal branches were assigned an arbitrary uniform length for visual clarity. Support for relationships is based on fractions of supporting and conflicting gene trees: pie charts show gene tree support and conflict per node (blue representing supporting gene trees, green gene trees supporting the most common alternative topology, red gene trees supporting further alternative topologies, grey gene trees uninformative for this node), and numbers above pie charts are Internode Certainty All support values [both calculated with PhyParts (Smith et al. 2015)]. If present, red numbers below pie charts are non-significant (i.e. > 0.05) outcomes of ASTRAL’s polytomy test (Sayyari and Mirarab 2018), which tests for each node whether the polytomy null model can be rejected. Monophyletic genera are represented by single branches; see Suppl. material 2 for a phylogeny with all accessions. See Suppl. material 3 for gene tree support across the phylogeny. The phylogeny is a pruned version of the backbone phylogeny of Ringelberg et al. (2023), where full details of the data and phylogenomic analysis methods are presented.

**Figure 7.**
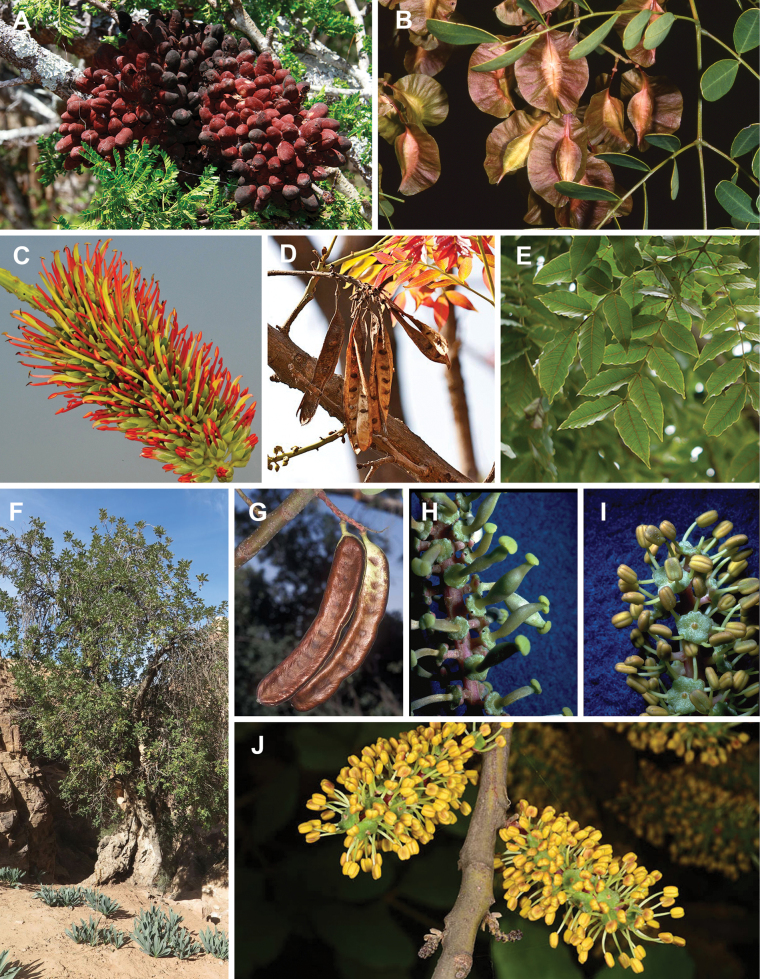
Flower, fruit, and vegetative characters of tribe Ceratonieae**A***Arcoagonavensis* Urb., foliage and fruits, Dominican Republic **B***Tetrapterocarpongeayi* Humbert, fruits and part of bipinnate leaf, Madagascar (*Du Puy M410*) **C–E***Acrocarpusfraxinifolius* Wight & Arn., India, cultivated tree **C** inflorescence **D** dehisced fruits and new flush foliage **E** part of a bipinnate leaf **F–J***Ceratoniasiliqua* L. **F** tree, Jordan, semi-desert near Petra **G** fruits, Israel, Mt. Scopus Botanical Gardens, Jerusalem **H** pistillate flowers, Crete **I** staminate flowers, Crete **J** staminate inflorescences, Israel, Jerusalem Botanical Gardens. Photo credits **A** F Jimenez R. **B** D Du Puy **C–E**https://efloraofindia.com/2011/02/01/acrocarpus-fraxinifolius/**E** D Valke **F, G, J** O Fragman-Sapir **H, I** G Lewis.

**Figure 8.**
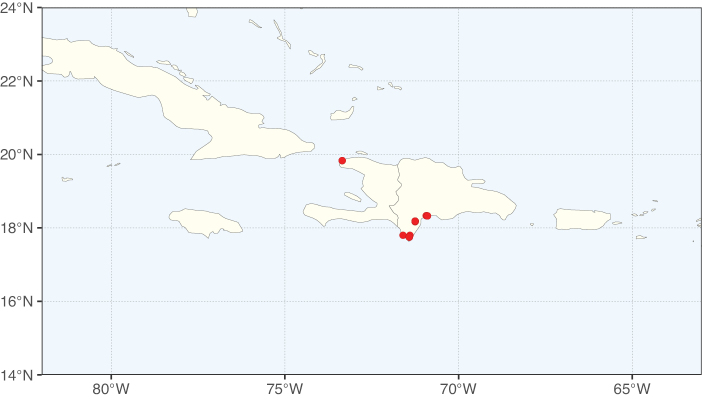
Distribution of *Arcoa* based on quality-controlled digitised herbarium records. See Suppl. material 1 for the source of occurrence data.

**Figure 9.**
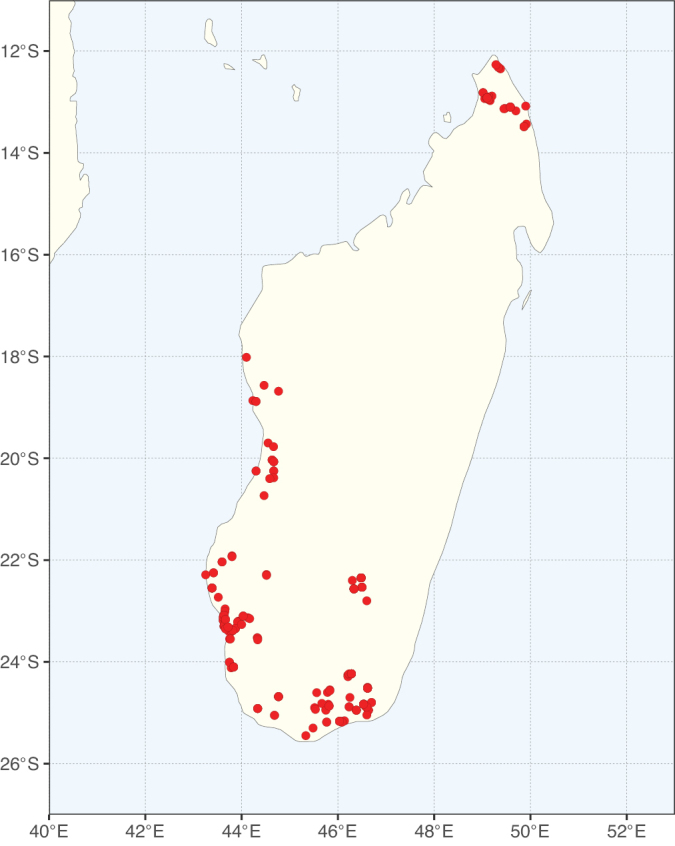
Distribution of *Tetrapterocarpon* based on quality-controlled digitised herbarium records. See Suppl. material 1 for the source of occurrence data.

**Figure 10.**
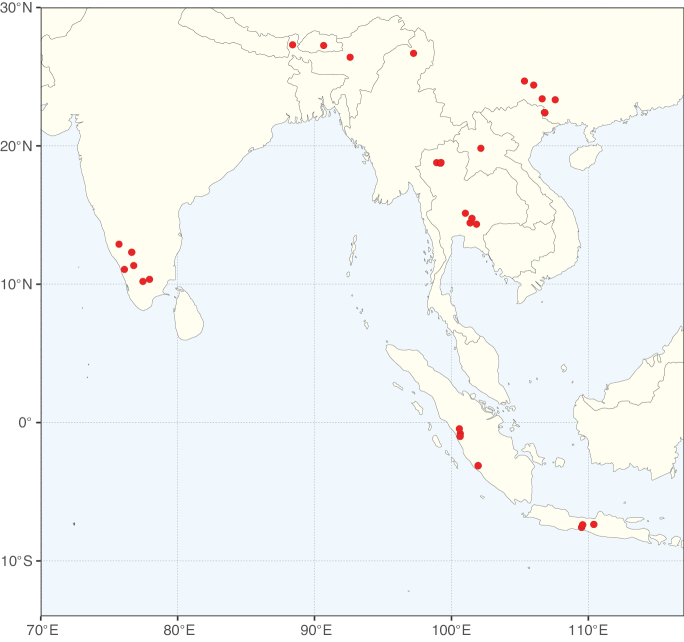
Distribution of *Acrocarpus* based on quality-controlled digitised herbarium records. See Suppl. material 1 for the source of occurrence data.

**Figure 11.**
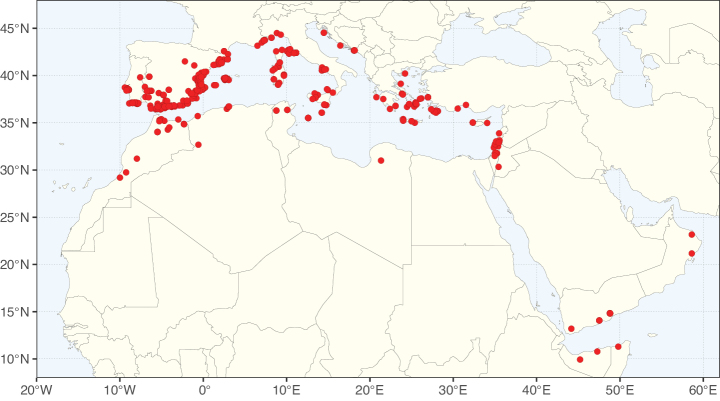
Distribution of *Ceratonia* based on quality-controlled digitised herbarium records. Note that many distribution points observed here are from cultivated *Ceratonia* specimens. The true native range of the genus *Ceratonia* is likely to be more restricted than depicted on the map. See Suppl. material 1 for the source of occurrence data.

Figs 6 , 7 , 8 , 9 , 10 , 11

Ceratoniinae H.S. Irwin & Barneby in R.M. Polhill & P.H. Raven, Adv. Legume System. 1: 98. 1981.

#### Type.

*Ceratonia* L.

#### Included genera

**(4).***Acrocarpus* Wight ex Arn. (1 species), *Arcoa* Urb. (1), *Ceratonia* L. (2), *Tetrapterocarpon* Humbert (2).

#### Description.

Unarmed (rarely the stipules of juvenile leaves spinescent) shrubs or trees. **Stipules** spinescent (*Arcoa*), minute, caducous or lacking. **Leaves** pinnate or bipinnate with a terminal pinna. **Inflorescences** erect or pendent racemes or panicles, sometimes clustered on short shoots or ramiflorous. **Flowers** unisexual or bisexual, a short hypanthium usually present, and sometimes a prominent central pulviniform disk; sepals valvate to slightly imbricate; petals absent or 4–5 (6) per flower; androecium haplo- to diplostemonous, or occasionally stamens more than 2× petal number. **Fruits** dehiscent or indehiscent, linear to linear-oblong with an adaxial narrow wing, or oblong-ellipsoid, or laterally compressed and 4-winged (the wings in two unequal pairs), 1–several-seeded. **Seeds** compressed, usually separated by areas of pulpy mesocarp, pleurogram lacking.

#### Distribution.

Highly disjunct in Hispaniola (*Arcoa*), Madagascar (*Tetrapterocarpon*), tropical (South-)East Asia (*Acrocarpus*), and north-eastern Africa, the Mediterranean, Oman, Yemen and Somalia (*Ceratonia*).

#### Clade-based definition.

The most inclusive crown clade containing *Arcoagonavensis* Urb. and *Ceratoniasiliqua* L., but not *Umtizalisteriana* Sim, *Dimorphandraconjugata* (Splitg.) Sandwith or *Mimosasensitiva* L. (Fig. 6).

#### Notes.

The tribal name Ceratonieae was first published by Reichenbach (1832) to accommodate the genus *Ceratonia*. Irwin and Barneby (1981) placed *Ceratonia* in its own new subtribe Ceratoniinae H.S. Irwin & Barneby of tribe Cassieae, and they would have accorded the genus tribal rank, i.e., a reinstatement of Reichenbachʼs (1832) taxon, “if not dissuaded by others” (Irwin and Barneby 1981: 98). Herendeen et al. (2003b), based on molecular and morphological data, grouped seven disparate caesalpinioid genera in their “Umtiza clade”, which included two major subclades: the Arcoa-Tetrapterocarpon-Acrocarpus-Ceratonia clade and the Gymnocladus-Umtiza-Gleditsia clade. More recent studies do not support the monophyly of these two clades together (Manzanilla and Bruneau 2012; LPWG 2013, 2017; Ringelberg et al. 2022) and they are here recognised under the two reinstated tribes Ceratonieae and Gleditsieae, respectively. Tribe Ceratonieae is resolved as sister to the rest of Caesalpinioideae (Fig. 6) and comprises six species in four genera (two of the genera are monospecific).

Only two characters have been found to be shared by members of tribe Ceratonieae: bipinnate leaves with a single terminal pinna (Fig. 7E; although *Ceratonia* leaves are usually singly pinnate) and three of the four genera (all except *Arcoa*, which is sister to the three other genera) have a large deletion in the plastid *trnL* intron (Bruneau et al. 2001). Three of the genera (*Acrocarpus* is the exception) are dioecious (although occasional bisexual flowers occur). *Ceratonia* and *Acrocarpus* are sister genera, both have simple inflorescences, a haplostemonous androecium, glabrous stamen filaments, alternate first seedling leaves, septate wood fibres, and a chromosome base number of *x* = 12. They differ in unisexual (*Ceratonia*) versus bisexual (*Acrocarpus*) flowers, absence of petals (*Ceratonia*) versus petals present (*Acrocarpus*), and fruit type: indehiscent (*Ceratonia*) versus dehiscent (*Acrocarpus*). Three of the four genera are confirmed as non-nodulating and the status of *Tetrapterocarpon* is not known (Sprent 2001; Faria et al. 2022).

### ﻿ Arcoa

Taxon classificationPlantaeFabalesFabaceae

Urb., Repert. Spec. Nov. Regni Veg. 19: 4. 1923.

Figs 7 , 8

#### Type.

*Arcoagonavensis* Urb.

#### Description.

Shrub or small tree, with well-developed brachylasts. **Stipules** spinescent, caducous. **Leaves** pinnate when juvenile, bipinnate with a terminal pinna when mature, leaflets opposite to subopposite, sessile. **Inflorescence** a sparsely branching panicle of spikes arising from a thickened woody brachyblast. **Flowers** unisexual, staminate flowers with a prominent pistillode, pistillate flowers with staminoidia; sepals free to a very short hypanthium; petals 5 (6); a short cupuliform disk present centrally; stamens (or staminodes) number more than twice sepal number (12 or more per flower); pollen markedly irregular and coarsely reticulate, porate with prominent pores; ovary with an appressed rust-coloured indumentum, stigma terminal and capitate. **Fruits** oblong-ellipsoid, subterete, thick-walled, indehiscent, 1–few-seeded. **Seeds** ovate, compressed, surrounded by copious pulp, pleurogram lacking (Fig. 7A).

#### Chromosome number.

Unknown.

#### Included species and geographic distribution.

Monospecific (*A.gonavensis*), endemic to Hispaniola (Dominican Republic, Haiti, and the Island of Gonâve) (Fig. 8).

#### Ecology.

Arid tropical vegetation on limestone hills and cliffs.

#### Etymology.

Named for Count George von Arco (ca. 1903).

#### Human uses.

Unknown.

#### Notes.

Originally placed by Urban (1923) in the Euphorbiaceae, *Arcoa* was subsequently transferred to the Leguminosae by Urban (1928). The genus was placed in the eclectic Dimorphandra group of Caesalpinieae by Polhill and Vidal (1981), along with *Tetrapetrocarpon*, both with unisexual flowers and petals not covered by the sepals in bud, but phylogenetic analyses clearly resolved *Arcoa* as part of the “Umtiza clade”, either sister to the Gleditsieae genera (Herendeen et al. 2003b) or sister to those of Ceratonieae (Bruneau et al. 2008), the latter as in Ringelberg et al. (2022).

#### Taxonomic references.

Lewis (2005b); Urban (1928).

### ﻿ Tetrapterocarpon

Taxon classificationPlantaeFabalesFabaceae

Humbert, Compt. Rend. Hebd. Séances Acad. Sci. 208: 374. 1939.

Figs 7 , 9

#### Type.

*Tetrapterocarpongeayi* Humbert

#### Description.

Unarmed trees or shrubs, dioecious; brachyblasts absent. **Stipules** inconspicuous, minute and caducous. **Leaves** bipinnate, ending in a terminal pinna; leaflets alternate. **Inflorescences** axillary, spike-like racemes of small, subsessile flowers, usually aggregated into panicles. **Flowers** unisexual, actinomorphic, 4-merous (sepals and petals 4 per flower), greenish; sepals equal, petals equal, both whorls imbricate in young bud; androecium diplostemonous in staminate flowers, with one whorl of 4 fertile stamens, their filaments with an apical tuft of hairs behind the anthers, and one whorl of 4 hairy staminodes, lacking anthers; pollen with a scabrate-punctate sculpture pattern; pistillate flowers with a stipitate, compressed-fusiform ovary, stigma capitate and bilobed. **Fruits** membranous, indehiscent, compressed, 4-winged (in two unequal pairs), 1-seeded (Fig. 7B). **Seeds** trapezoid, subterete, club-shaped, pleurogram lacking.

#### Chromosome number.

Unknown.

#### Included species and geographic distribution.

Two species, both endemic to Madagascar (Fig. 9).

#### Ecology.

Seasonally dry tropical to xerophytic forest and thicket, on limestone, basalt, or sand.

#### Etymology.

From Greek, *tetra*- (= four), *ptero*- (= winged) and *carpos* (= fruit), the fruits have four dry, papery wings in unequal pairs.

#### Human uses.

*Tetrapterocarpongeayi* is used locally for carpentry, cart construction and to make charcoal (Du Puy and Rabevohitra 2002).

#### Notes.

Although placed in the Dimorphandra group of the Caesalpinieae by Polhill and Vidal (1981), the genus was resolved as sister to an Acrocarpus-Ceratonia clade in a study of the “Umtiza clade” by Herendeen et al. (2003b), sharing with these two genera a bipinnate leaf with an unequal leaflet base, and this relationship is supported in the phylogenomic analysis of Ringelberg et al. (2022) (Fig. 6).

#### Taxonomic references.

Du Puy and Rabevohitra (2002); Lewis (2005b).

### ﻿ Acrocarpus

Taxon classificationPlantaeFabalesFabaceae

Wight ex Arn., Mag. Zool. Bot. 2(12): 547. 1839.

Figs 7 , 10

#### Type.

*Acrocarpusfraxinifolius* Wight & Arn.

#### Description.

Unarmed evergreen tree, bark pale grey, smooth, brachyblasts absent. **Stipules** not seen, presumed lacking, at least on mature leaves. **Leaves** large, bipinnate with a single terminal pinna, leaflets ovate-lanceolate, acuminate (Fig. 7E). **Inflorescences** densely flowered, erect, or drooping racemes, or panicles (Fig. 7C). **Flowers** bisexual, patent or nodding, 5-merous, sepals and petals green, sepals slightly imbricate, petals slightly longer; disk cupular, completely united with the red hypanthium; androecium haplostemonous, stamens five, exserted from corolla (Fig. 7C), filaments with basal half green, upper half orange-red, anthers dorsifixed, with introse slits; pollen with a scabrate-punctate sculpture pattern; ovary stipitate, the short style tapered and inflexed to a minute stigmatic pad. **Fruits** linear to linear-oblong, dehiscent, 2-valved, narrowly winged along upper suture, several- to many-seeded (Fig. 7D). **Seeds** ovate or circular, compressed, testa surface with concentric fracture lines, pleurogram lacking.

#### Chromosome number.

2*n* = 24 (Goldblatt 1981b).

#### Included species and geographic distribution.

Monospecific (*A.fraxinifolius*), native to South East Asia (from the Indian subcontinent to China and Indo-China) (Fig. 10).

#### Ecology.

Tropical and subtropical broad-leaved rainforest and evergreen gallery forest.

#### Etymology.

From Greek, *acro*- (= summit or top) and *carpos* (= fruit), most probably alluding to the long-stipitate ovaries and fruits.

#### Human uses.

Timber of *A.fraxinifolius* (pink cedar tree) is used to make tea boxes, furniture, and plywood; the species is widely grown as an ornamental and is also used for fodder, gums, and bee forage (for honey) (Lewis 2005b).

#### Notes.

*Acrocarpus* was earlier placed in its own Acrocarpus group of tribe Caesalpinieae (Polhill and Vidal 1981), but the first molecular phylogenetic analyses based on just a few plastid markers (Doyle et al. 2000; Bruneau et al. 2001; Kajita et al. 2001) suggested the genus was closely related to *Ceratonia* (then placed in Cassieae), a relationship that clearly is supported in the phylogenomic analyses of Ringelberg et al. (2022). A large range of flower size throughout its distribution range formerly led to the recognition of two species.

#### Taxonomic references.

Hou (1996a); Lewis (2005b).

### ﻿ Ceratonia

Taxon classificationPlantaeFabalesFabaceae

L., Sp. Pl. 2: 1026. 1753.

Figs 7 , 11

Siliqua Duhamel, Traité Arbr. Arbust. 2: 261. 1755, nom. superfl.
Ceratia Adans., Fam. Pl. 2: 319. 1763. Type not designated.

#### Type.

*Ceratoniasiliqua* L.

#### Description.

Long-lived, evergreen, small to medium-sized trees (to ca. 12 m) (Fig. 7F) and shrubs, polygamous or dioecious or occasionally hermaphrodite; brachyblasts absent. **Stipules** minute, caducous or lacking. **Leaves** usually once pinnate, although bipinnate leaves rarely occur in *C.siliqua*; leaflets opposite to alternate. **Inflorescences** short spike-like racemes, solitary or fasciculate, ramiflorous on main branches (Fig. 7J) or clustered on young growth. **Flowers** commonly unisexual, apetalous, staminate flowers haplostemonous and bearing a pistilloide (Fig. 7I), pistillate flowers sometimes bearing staminodes (Fig. 7H), functionally bisexual flowers rare, calyx lobes (4) 5, imbricate, shortly connate at base, a very short hypanthium and a large pulviniform (cushion-like) disk present (Fig. 7H, I); pollen tetracolporate (*C.siliqua*) or tricolporate (*C.oreothauma* Hillc., G.P. Lewis & Verdc.); ovary short-stipitate. **Fruits** oblong, thick, indehiscent, dark brown and sub-woody when mature, with a sweet pulpy mesocarp (*C.siliqua*, Fig. 7G), or coriaceous to sub-woody, laterally compressed but with the valves raised over the seed chambers, mesocarp dry (*C.oreothauma*), many-seeded. **Seeds** very hard, ovate to oblong or pyriform, laterally compressed, separated by a pulpy, sugary mesocarp (*C.siliqua*), pleurogram lacking.

#### Chromosome number.

2*n* = 24 (Goldblatt 1981b).

#### Included species and geographic distribution.

Two species, one (*C.siliqua*) native to north-eastern Africa and the eastern Mediterranean (its native range uncertain due to its long history of cultivation), and *Ceratoniaoreothauma*, with two distinct subspecies, one in Oman and Yemen and the other in the Somali Republic (Fig. 11).

#### Ecology.

Mediterranean scrubland (dry hillsides in garigue and coastal and submaritime maquis) (*C.siliqua*); rocky limestone slopes and gullies (*C.oreothauma*); 0–2000 m.

#### Etymology.

From ‘Ceratonia’, ‘ceronia’ or ‘ceratea’ (Greek names for *C.siliqua*), or possibly from *ceras* (Greek = horn) referring to the long, curved pods of *C.siliqua*.

#### Human uses.

*Ceratoniasiliqua* is widely cultivated in the Mediterranean for forest-forage and its nutritious fruits; the Romans were harvesting the species as early as 79 AD. Carob seeds are said to be the original carat used as a standard weight by jewellers. *Ceratoniasiliqua* is also used for wood, as a chocolate and coffee substitute, and occasionally to make alcohol. Carob seed gum is used in foods, cosmetics, medicines, photographic film emulsions, adhesives, paints, inks, and polishes (Lewis 2005b). Ceratoniaoreothaumasubsp.oreothauma is used locally in Oman as goat fodder (Hillcoat et al. 1980).

#### Notes.

The two *Ceratonia* species are differentiated, amongst other characters, by the pollen type, tetracolporate in *C.siliqua* and tricolporate in *C.oreothauma* (Ferguson 1980; Graham and Barker 1981). *Ceratoniasiliqua* is highly plastic in the sexuality of individual trees, in inflorescence branching pattern (racemose or cymose), in presence or absence of floral bracts, in organ number per whorl, missing floral organs, pollen grain form, and carpel cleft orientation (Tucker 1992).

#### Taxonomic references.

Hillcoat et al. (1980); Lewis (2005b); Meikle (1977); Thulin (1993).

## ﻿﻿3. Tribe Gleditsieae

Patrick S. Herendeen^21^, Anne Bruneau^1^

Citation: Herendeen PS, Bruneau A (2024) 3. Tribe Gleditsieae. In: Bruneau A, Queiroz LP, Ringelberg JJ (Eds) Advances in Legume Systematics 14. Classification of Caesalpinioideae. Part 2: Higher-level classification. PhytoKeys 240: 70–77. https://doi.org/10.3897/phytokeys.240.101716

### ﻿Tribe Gleditsieae

Taxon classificationPlantaeFabalesFabaceae

Nakai, Chosakuronbun Mokuroku [Ord. Fam. Trib. Nov.]: 253. 1943.

**Figure 12.**
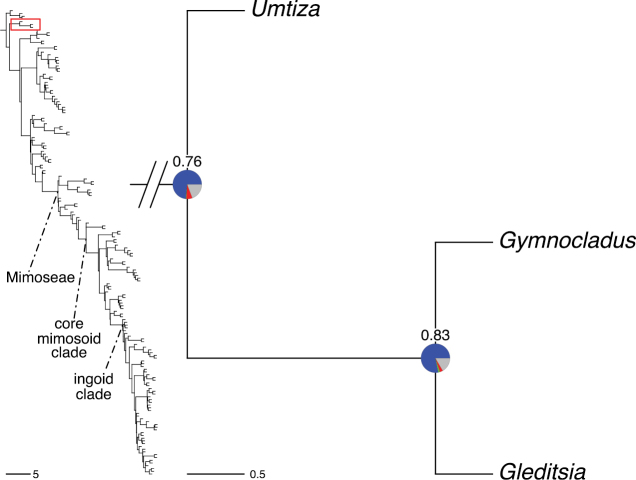
Generic relationships in tribe Gleditsieae. For description of phylogeny and support values, see Fig. 6 caption (page 63).

**Figure 13.**
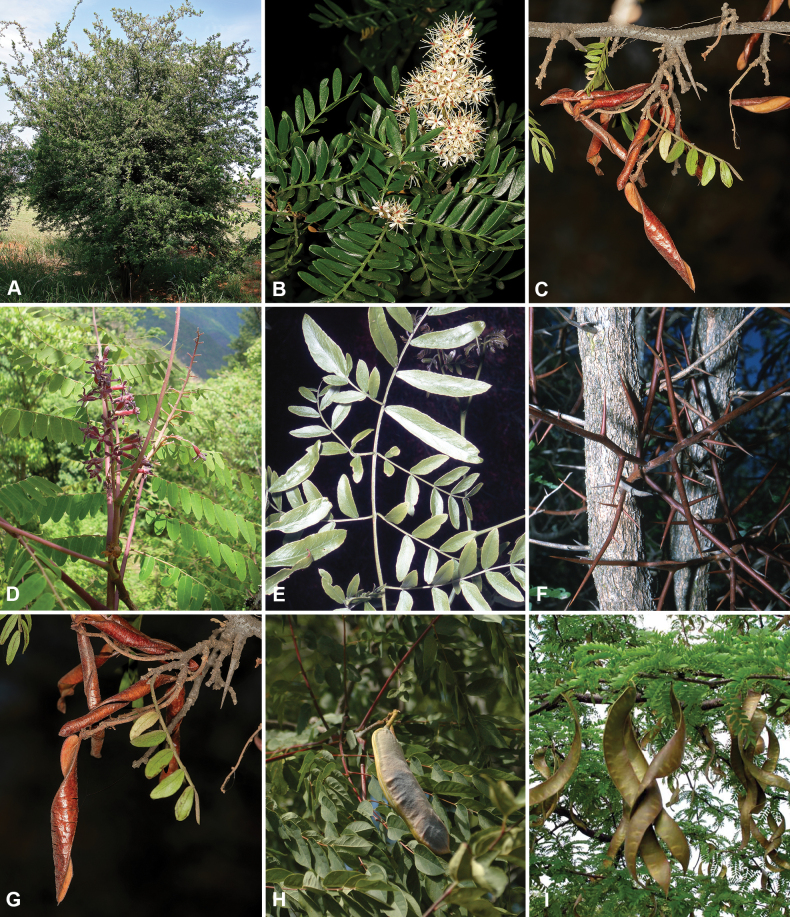
Gleditsieae, diversity of vegetative morphology and fruits **A–C***Umtizalisteriana* Sim **A** small tree **B** branch with pinnately compound leaves and inflorescence **C** branch with thorn at node **D***Gymnocladusassamicus* Kanjilal ex P.C. Kanjilal branch with bipinnate leaves and inflorescence **E***Gleditsiajaponica* Miq., bipinnate leaf with several pinnae replaced by single leaflets, cultivated at the Royal Botanic Gardens, Kew **F***Gleditsiaamorphoides* (Griseb.) Taub., branched thorns **G***Umtizalisteriana* dehisced fruits **H***Gymnocladusdioicus* L., fruit **I***Gleditsiatriacanthos* L., fruits. Photo credits **A–C, G** SAplants, Wikimedia Commons (CC-BY-SA 4.0) **D** B Choudhury **E, F** GP Lewis **H** Chicago Botanic Garden (photographer unknown) **I** A Schnabel.

**Figure 14.**
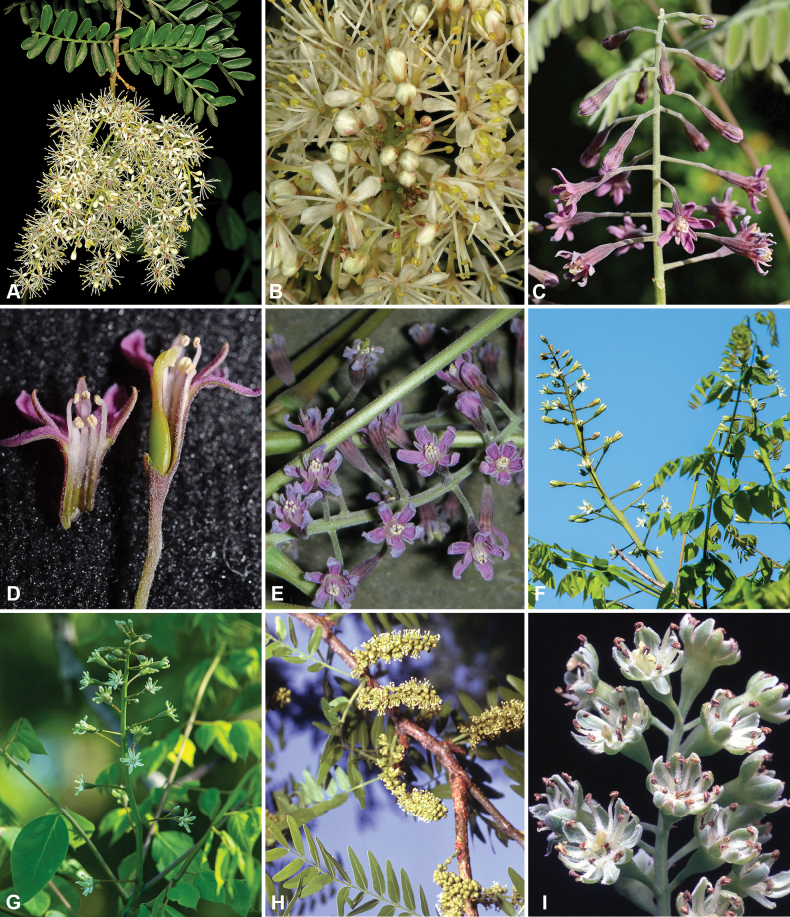
Gleditsieae, floral diversity **A, B***Umtizalisteriana* Sim **A** branch with inflorescence of hermaphrodite flowers **B** close-up of flowers **C, D***Gymnocladusassamicus* Kanjilal ex P.C. Kanjilal **C** inflorescence of hermaphrodite flowers **D** dissected hermaphrodite flower **E***Gymnocladuschinensis* Baill., inflorescence, cultivated at the US National Arboretum **F***Gymnocladusdioicus* L., cultivated at Chicago Botanic Garden, upright inflorescence of hermaphrodite flowers **G** branch with leaves and inflorescence **H***Gleditsiatriacanthos* L., inflorescence of staminate flowers **I***Gleditsiacaspica* Desf., inflorescence, cultivated at the Royal Botanic Gardens, Kew. Photo credits **A, B** SAplants, Wikimedia Commons (CC-BY-SA 4.0) **C, D** Murata **E** PS Herendeen **F, G** R Carlson **H** Royal Botanic Gardens, Kew (photographer unknown) **I** M Svanderlik.

**Figure 15.**
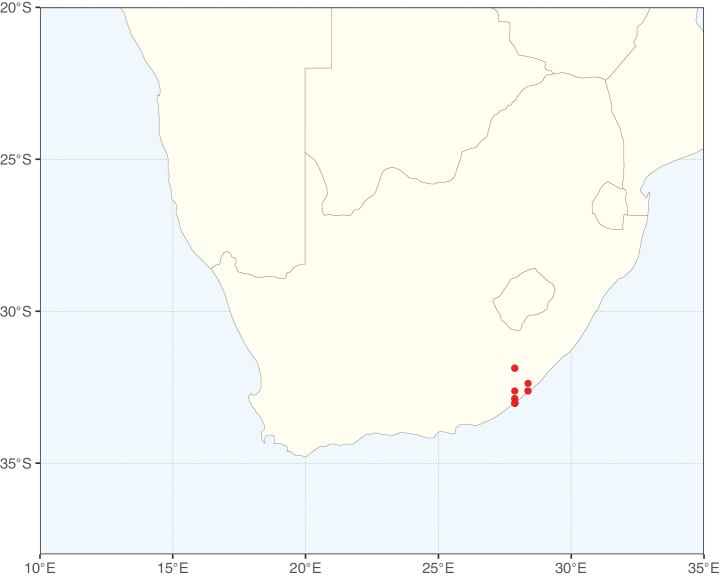
Distribution of *Umtiza* based on quality-controlled digitised herbarium records. See Suppl. material 1 for the source of occurrence data.

**Figure 16.**
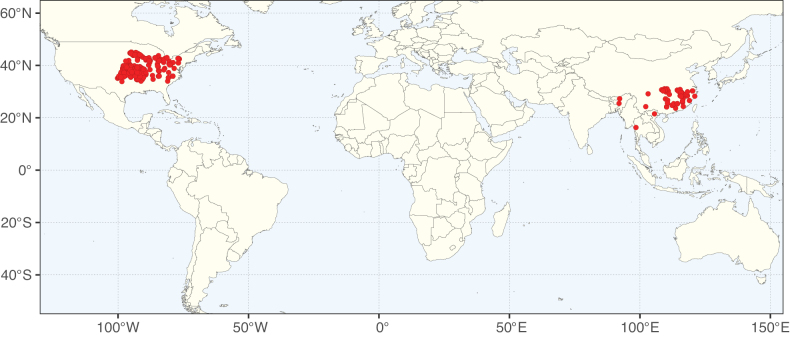
Distribution of *Gymnocladus* based on quality-controlled digitised herbarium records. See Suppl. material 1 for the source of occurrence data.

**Figure 17.**
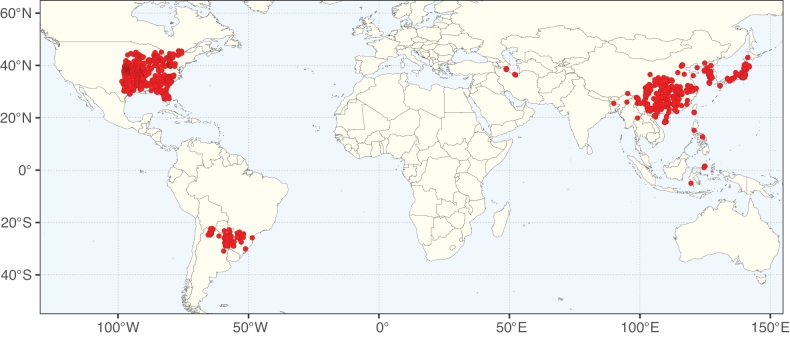
Distribution of *Gleditsia* based on quality-controlled digitised herbarium records. See Suppl. material 1 for the source of occurrence data.

Figs 12 , 13 , 14 , 15 , 16 , 17

#### Type.

*Gleditsia* J. Clayton

#### Included genera

**(3).***Gleditsia* J. Clayton (13 species), *Gymnocladus* Lam. (6), *Umtiza* Sim (1).

#### Description.

Deciduous or evergreen trees, with simple or branched thorns or unarmed; branches with or without lateral brachyblasts. **Stipules** inconspicuous (*Gleditsia*), foliaceous, caducous (*Gymnocladus*), or absent (*Umtiza*). **Leaves** bipinnate, pinnate, or intermediate with one or more pinnae replaced by a leaflet. **Inflorescences** panicles or racemes. **Flowers** regular, bisexual (*Umtiza*), or unisexual and then androdioecious or dioecious (*Gleditsia*, *Gymnocladus*), small, white or greenish to violet, mostly 5-merous; hypanthium short to elongate; calyx gamosepalous; petals small; stamens (6) 10, free; pollen in tricolporate monads, exine perforate, reticulate; ovary sessile or stipitate. **Fruit** compressed or turgid, papery, leathery or woody, dehiscent, tardily dehiscent or indehiscent, sometimes with pulpy interior, with few to 25 (40) seeds. **Seeds** compressed to subterete, orbicular or ovoid-elliptic.

#### Distribution.

Temperate to subtropical North America, South America, Asia, and South Africa.

#### Clade-based definition.

The most inclusive crown clade containing *Umtizalisteriana* Sim and *Gymnocladusdioicus* (L.) K. Koch, but not *Ceratoniasiliqua* L., *Dimorphandraconjugata* (Splitg.) Sandwith or *Mimosasensitiva* L. (Fig. 12).

#### Notes.

Tribe Gleditsieae was first named by Nakai (1943) to accommodate *Gleditsia* and *Gymnocladus*. A close relationship between the two genera has long been known (Polhill and Vidal 1981; Polhill 1994; Lewis 2005b). *Gleditsia* and *Gymnocladus* were resolved together with *Umtiza*, as well as with *Tetrapterocarpon* Humbert, *Arcoa* Urb., *Ceratonia* L. and *Acrocarpus* Wight ex Arn., in a clade that was informally named the Umtiza clade (Herendeen et al. 2003a, 2003b). The close relationship of *Gleditsia*, *Gymnocladus* and *Umtiza* has been confirmed by Manzanilla and Bruneau (2012), LPWG (2013, 2017) and Ringelberg et al. (2022), but the monophyly of the larger Umtiza clade is not supported (Fig. 12). *Umtiza* was previously placed in the Cynometra group of tribe Detarieae by Cowan and Polhill (1981) with some uncertainty. The small regular flowers and thorns in *Umtiza* are notable morphological similarities to *Gleditsia*. The three genera differ most in fruit structure and dehiscence.

### ﻿ Umtiza

Taxon classificationPlantaeFabalesFabaceae

Sim, Forest Fl. Cape Good Hope: 205. T. 52. 1907.

Figs 13 , 14 , 15

#### Type.

*Umtizalisteriana* Sim

#### Description.

Small evergreen trees to 12 m or shrubs, branches armed with stout spines that are frequently branched; branches with lateral brachyblasts (Fig. 13A–C). **Stipules** absent. **Leaves** paripinnate, alternate, (3) 5–9 (12) pairs of subopposite leaflets, sometimes with a lateral terminal leaflet. **Inflorescences** panicles, terminal on spur shoots; bracts minute, persistent. **Flowers** white, small, regular, bisexual, 5-merous or more rarely 4-merous (Fig. 14A, B); hypanthium short; calyx lobes subequal, sometimes one lobe longer; petals equal, free, inserted on hypanthium rim with the stamens; androecium diplostemonous, stamens opposite petals shorter than those alternate with petals; pollen exine perforate; ovary sessile bearing two ovules. **Fruit** dehiscent along both sutures, subligneous, valves twisting, not pulpy, usually one-seeded (Fig. 13G). **Seed** ovate with truncate base, compressed.

#### Chromosome number.

Unknown.

#### Included species and geographic distribution.

Monospecific (*U.listeriana*), from Eastern Cape region, South Africa, primarily along the Buffalo River between East London and King William’s Town (Fig. 15).

#### Ecology.

Subtropical dry forest, bushland and thicket.

#### Etymology.

Derived from ‘umthiza’, the African vernacular name for the single species, *U.listeriana*.

#### Human uses.

Used locally for medicine and has alleged magical properties, witchdoctors have used sticks of the tree as healing wands; the wood was once used to house propeller shafts in small boats because its oiliness provided constant lubrication (Ross 1977).

#### Notes.

The genus is monospecific with *Umtizalisteriana* endemic to the Eastern Cape region of South Africa where the species is endangered and protected. The Umtiza Nature Reserve is named for this species. Cowan and Polhill (1981) treated *Umtiza* as a member of the Detarieae, but with uncertainty, partially due to the absence of stipules. Typically, intrapetiolar stipules characterise Detarieae and Amherstieae, but their absence in *Umtiza* made tribal affinity uncertain. *Umtiza* is most similar to species of *Gleditsia* in the presence of branched and unbranched thorns derived from lateral shoots. *Umtiza* differs from the other two genera of the tribe in the presence of only hermaphrodite flowers and fully dehiscent fruits that lack pulp.

#### Taxonomic references.

Allen and Allen (1981); Ross (1977), with illustration.

### ﻿ Gymnocladus

Taxon classificationPlantaeFabalesFabaceae

Lam. Encycl. Meth., Bot. 1: 733. 1785.

Figs 13 , 14 , 16

#### Lectotype.

*Gymnocladuscanadensis* Lam., nom. illeg. [= *Gymnocladusdioicus* (L.) Koch (≡ *Guilandinadioica* L.; vide Britton and Rose 1930)]

#### Description.

Large to medium size deciduous trees, unarmed, stems often stout, androdioecious (individuals with either staminate flowers or hermaphrodite flowers) or dioecious. **Stipules** small, inconspicuous. **Leaves** even- or irregularly-bipinnate, pinnae 3–10 pairs, proximal-most sometimes reduced to single leaflets, opposite, subopposite or alternate; leaflets alternate, 6–30 per pinna, lamina margin entire (Figs 13D, 14F, G). **Inflorescences** terminal, racemes or panicles, 15–50 flowers (Fig. 14E, F); bracts and bracteoles usually absent, sometimes present, minute. **Flowers** white, greenish white, or purple, pedicellate, regular, hypanthium elongate; calyx lobes 5, narrow; corolla lobes 5, wider than sepal lobes; stamens 10, inserted on hypanthium; pollen exine perforate to reticulate; ovary sessile or short-stipitate, style short, thick (Fig. 14C, D). **Fruit** sessile, compressed, turgid, oblong, indehiscent or tardily dehiscent on placental suture, woody, pulpy between seeds, 1–8-seeded (Fig. 13H). **Seeds** subglobose to somewhat flattened, testa smooth to rough.

#### Chromosome number.

2*n* = 28 (Goldblatt 1981b).

#### Included species and geographic distribution.

Six species in eastern and central North America, China, Vietnam, India, Burma, Thailand (Fig. 16). The limits of the natural distribution of *G.dioicus* are unclear due to its widespread cultivation in eastern North America. *Gymnocladusassamicus* Kanjilal ex P.C. Kanjilal from the eastern Himalayan region of India is critically endangered.

#### Ecology.

Temperate bottomlands and riparian woodlands, tropical and subtropical montane woodlands and wooded hillsides (Chen et al. 2010c).

#### Etymology.

The genus name derives from the Greek (= naked branch) because *G.dioicus* is one of the latest trees to leaf out in the spring.

#### Human uses.

Multiple species of *Gymnocladus* are used by people in different parts of the world (Lee 1976; Allen and Allen 1981; Poindexter et al. 2022a). *Gymnocladusdioicus* is widely planted as an ornamental and street tree. The wood of *G.dioicus* and *G.chinensis* is used for fence posts, construction, railway ties and carpentry. Roasted seeds of *G.dioicus* were formerly used as a coffee substitute, hence the common name Kentucky Coffee Tree used in North America. Some species are used for medicine, insecticides, oils, and soap.

#### Notes.

The taxonomy of the species of *Gymnocladus* is well established, but the monophyly of the genus has not been adequately tested. Only *G.dioicus* and/or *G.chinensis* have been included in phylogenetic analyses (Herendeen et al. 2003b; Schnabel et al. 2003; Choudhury and Khan 2020; Feng et al. 2021; Ringelberg et al. 2022). Given the morphological variation among the species of *Gymnocladus* and similarities to *Gleditsia* it would be worthwhile to test the monophyly of *Gymnocladus* in an analysis that includes more species and samples. Lee (1976) recognised four species in the genus (two additional species were described subsequently), which he hypothesised to be of tropical origin. Choudhury et al. (2014) documented functional androdioecy in *G.assamicus*. The chloroplast genome of *G.chinensis* was characterised by Feng et al. (2021).

#### Taxonomic references.

Allen and Allen (1981); Poindexter et al. (2022a); Schnabel et al. (2003).

### ﻿ Gleditsia

Taxon classificationPlantaeFabalesFabaceae

J. Clayton in Linnaeus, Gen. Pl. 5: 476. 1754.

Figs 13 , 14 , 17

Melilobus Mitch., Diss. Gen. Pl.: 37. 1769. Type: Melilobusheterophyla Raf. [= Gleditsiatriacanthos L.]
Asacara Raf., Neogenyton: 2. 1825. Type: Asacaraaquatica (Marshall) Raf. [≡ Gleditsiaaquatica Marshall]
Garugandra Griseb., Abh. Königl. Ges. Wiss. Göttingen 24: 96. 1879. Type: Garugandraamorphoides Griseb. [≡ Gleditsiaamorphoides (Griseb.) Taub.]
Caesalpiniodes Kuntze, Rev. Gen. 1: 166. 1891. Type: Caesalpiniodestriacanthum (L.) Kuntze [≡ Gleditsiatriacanthos L.]
Pogocybe Pierre, Fl. Cochinch.: t. 392B. 1899. Type: Pogocybeentadoides Pierre. [= Gleditsiaaustralis Hemsl.]

#### Type.

*Gleditsiatriacanthos* L.

#### Description.

Large to medium size deciduous trees (*G.saxatilis* was described as evergreen), usually well-armed with simple or compound thorns on trunk and branches (Fig. 13F), sometimes thornless, especially in cultivated plants; branches with lateral brachyblasts; androdioecious (individuals with either staminate flowers or hermaphrodite flowers) or dioecious. **Stipules** small, inconspicuous. **Leaves** bipinnate, pinnate, or intermediate with one or more pinnae replaced by a leaflet, often all forms found on the same plant; bipinnate leaves, 2–10 pairs of pinnae, leaflets (1) 4–32 per pinna, lamina margins usually crenulate, rarely entire (Fig. 13E). **Inflorescences** terminal, axillary or cauliflorous racemes, few to many flowered; bracts present, caducous (Fig. 14H, I). **Flowers** greenish white, regular, staminate flowers smaller than bisexual flowers; hypanthium well developed, short, narrow; calyx bell-shaped, lobes 3–5, subequal; petals 3–5, subequal; stamen number variable, 3–10, haplostemonous or diplostemonous; pollen, exine perforate to reticulate; ovary sessile/stipitate. **Fruit** stipitate, flat or compressed, indehiscent, papery, leathery or cartilaginous, often with pulpy interior, 1–40-seeded (Fig. 13I). **Seeds** elliptic to quadrangular or ovate to circular, compressed to terete.

#### Chromosome number.

2*n* = 28 (Goldblatt 1981b).

#### Included species and geographic distribution.

Thirteen species and 1 hybrid taxon. Eastern and central North America to northern Mexico; Bolivia to northern Argentina; China, Tibet, Vietnam, Korea, Japan, Malaysia, Philippines, Azerbaijan (Fig. 17). Widespread cultivation of *G.triacanthos* has greatly expanded its range in North America and obscured its original native area.

#### Ecology.

Species are generally found in temperate and subtropical woodlands and thickets on sandy and rocky slopes, and lowland wet forest and swamp forest (Chen et al. 2010d; Poindexter et al. 2022b). Due to widespread cultivation of *G.triacanthos* in North America its original habitat preferences are unclear.

#### Etymology.

The genus name commemorates Johann Gottlieb Gleditsch, director of the Berlin Botanical Garden, who died in 1786.

#### Human uses.

Multiple species of *Gleditsia* are used by people in different parts of the world (Gordon 1966; Allen and Allen 1981; Poindexter et al. 2022b). The thornless form of *G.triacanthos* is widely cultivated as an ornamental and street tree in North America and elsewhere. The sweet fruits are used as a fodder for livestock. The rot resistant wood is used for fence posts, construction and railway ties. The thorns have a variety of historic uses (e.g., as needles and for carding wool). Fruits of some species are rich in saponins and have been used as soap substitutes.

#### Notes.

Phylogenetic analyses indicate that the genus is monophyletic (Herendeen et al. 2003b; Schnabel et al. 2003; Manzanilla and Bruneau 2012; LPWG 2013, 2017). The genus was monographed by Gordon (1966). The taxonomy of Chinese species was revised in the Flora of China, which recognised six species in the region (Chen et al. 2010d). In 2021, *G.saxatilis* Z.C. Lu, Y.S. Huang & Y. Liu was described from Guangxi, China (Lu et al. 2021). The species is notable in that it is described to be evergreen. Variability, especially in leaf form, flower structure, and thorn development has resulted in the description of numerous species, subspecies, varieties and forms that are now treated as synonyms under several recognised species, especially *G.japonica* Miq., *G.sinensis* Lam. and *G.triacanthos*.

#### Taxonomic references.

Allen and Allen (1981); Chen et al. (2010d); Gordon (1966); Poindexter et al. (2022b).

## ﻿﻿4. Tribe Pterogyneae

Luciano Paganucci de Queiroz^2^, Filipe Gomes Oliveira^2^

Citation: Queiroz LP, Oliveira FG (2024) I4. Tribe Pterogyneae In: Bruneau A, Queiroz LP, Ringelberg JJ (Eds) Advances in Legume Systematics 14. Classification of Caesalpinioideae. Part 2: Higher-level classification. PhytoKeys 240: 78–82. https://doi.org/10.3897/phytokeys.240.101716

### ﻿Tribe Pterogyneae

Taxon classificationPlantaeFabalesFabaceae

Legume Phylogeny Working Group, tribus nov.

urn:lsid:ipni.org:names:77339337-1

Figs 18 , 19 , 20

#### Diagnosis.

Differing from all other tribes of subfamily Caesalpinioideae by the combination of imparipinnate leaves with a well-formed rachis extension, alternate leaflets, compact catkin-like racemes, flowers small, polygamous and regular, the ovary with a marginal wing, style subterminal, fruit a samara with the basal seed chamber with a small subterminal beak (style remnant), and chromosome number 2*n* = 20.

#### Type.

*Pterogyne* Tul. (designated here).

#### Included genera.

Tribe Pterogyneae includes only the genus *Pterogyne* with one species.

#### Clade-based definition.

The most inclusive crown clade containing *Pterogynenitens* Tul., but not *Ceratoniasiliqua* L., *Umtizalisteriana* Sim or *Cassiafistula* L.

#### Notes.

**Figure 18.**
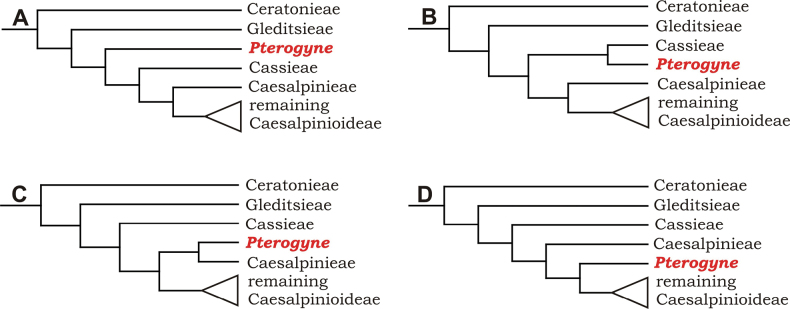
Contrasting phylogenetic positions for *Pterogyne* as supported by different analyses **A** sister to all Caesalpinioideae, except Ceratonieae and Gleditsiae (ca. 1500 nuclear genes; Zhao et al. 2021) **B** sister to Cassieae (SUSY nuclear gene and combined *trnL* intron + morphological data; Manzanilla and Brunau 2012, Herendeen et al. 2003a, respectively) **C** sister to Caesalpinieae (*trnL* intron and coding plastid genes; Bruneau et al. 2001; Zhang et al. 2020) **D** sister to the remaining Caesalpinioideae (plastid non-coding loci; Zhang et al. 2020).

The tribe Pterogyneae is being proposed here to include the single genus *Pterogyne*. This genus was previously ascribed to the tribe Cynometreae (currently subfamily Detarioideae) by Bentham (1865) and Taubert (1894). Later, it was recognised as morphologically distinct and placed in the phenetically isolated Pterogyne group within the broadly polymorphic and polyphyletic tribe Caesalpinieae (sensu Polhill and Vidal 1981; Lewis 2005b). This isolated position was also demonstrated by phylogenetic analyses using different morphological and molecular markers, in which *Pterogyne* is resolved in quite different positions in the Caesalpinioideae phylogeny (Fig. 18). Most plastid sequence data support a position of *Pterogyne* as sister to the Caesalpinieae (Caesalpinia clade in Bruneau et al. 2001, 2008; Fig. 18C), whereas combined analyses of plastid and morphological data (Herendeen et al. 2003a) and the nuclear sucrose synthase (SUSY) gene (Manzanilla and Bruneau 2012) suggest a closer relationship with the Cassieae (Fig. 18B). More recent analyses of the entire plastid genome of the Leguminosae also provided ambiguous results and found *Pterogyne* to cluster with the Caesalpinieae when considering only the coding genes (Fig. 18C) and sister to all remaining Caesalpinioideae when considering the non-coding loci (Fig. 18D; Zhang et al. 2020). Phylogenomic analyses of ca. 1500 nuclear genes supported *Pterogyne* as sister to all Caesalpinioideae, except the Ceratonieae and Gleditisieae clades (Fig. 18A; Zhao et al. 2021; Kates et al. 2024).

The uncertain phylogenetic position of *Pterogyne* probably reflects the short diversification time between the divergence of the four distinct lineages, *Pterogyne*, Cassieae, Caesalpinieae, and the remaining Caesalpinioideae, in the Paleocene, with *Pterogyne* representing a long branch diverging from those other lineages between 57 Ma and 60 Ma (Zhao et al. 2021).

The recognition of the monogeneric (and monospecific) tribe Pterogyneae is additionally supported by its unique combination of morphological features, as highlighted in the diagnosis above. This isolated evolutionary position is also corroborated by a chromosome number of 2*n* = 20 for *Pterogynenitens*, which is cytologically unique in subfamily Caesalpinioideae (Goldblatt 1981b).

### ﻿ Pterogyne

Taxon classificationPlantaeFabalesFabaceae

Tul., Ann. Sci. Nat., Bot., sér. 2, 20: 140. 1843.

Figs 19 , 20

#### Type.

*Pterogynenitens* Tul.

#### Description.

**Figure 19.**
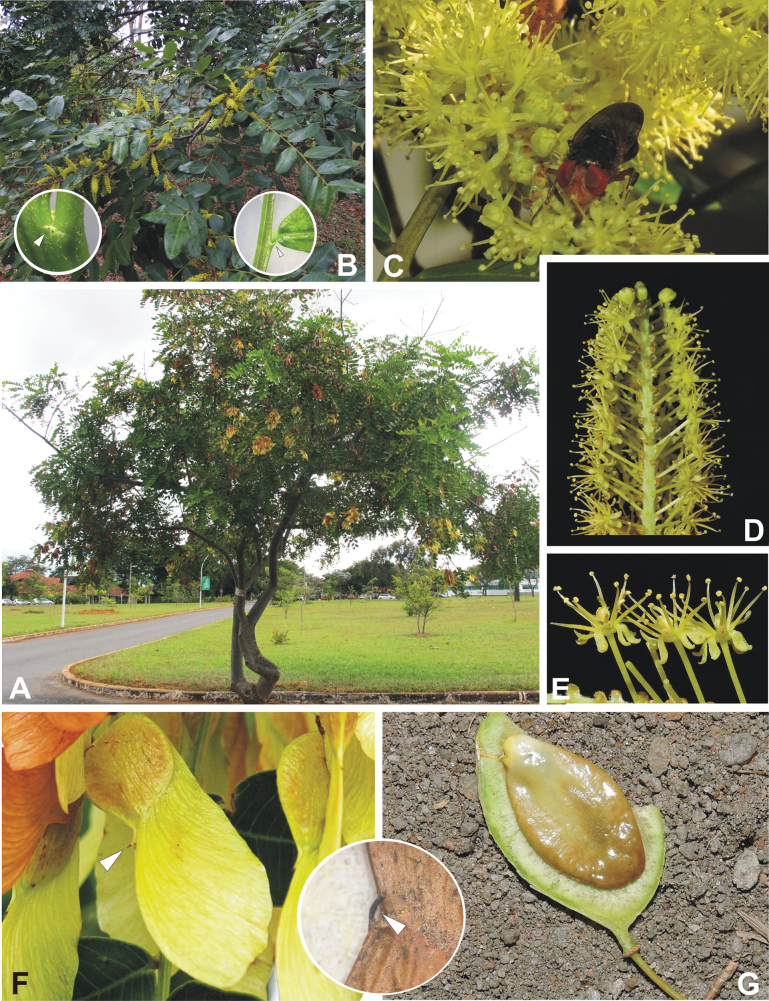
*Pterogynenitens* Tul., the only species of tribe Pterogyneae**A** cultivated tree in Brasília (Brazil) **B** flowering branch showing the foliage and inflorescences; note the alternate leaflets; the insets show an expanding leaf (left) highlighting the tiny caducous stipules (arrow) and a stipel at the leaflet attachment (right) **C** inflorescences with a visiting Syrphidae fly (Diptera) **D** part of the spicate raceme with some flowers removed **E** close-up of flowers **F** samaras, the inset showing the subterminal style remnant at the top of the seed chamber (arrows) **G** samara seed chamber opened to show the seed. Photo credits **A–G** RT Queiroz https://rubens-plantasdobrasil.blogspot.com/.

Medium to tall trees, mostly 4–8 m but sometimes to 20 m, unarmed (Fig. 19A); bark light grey; short shoots absent. **Stipules** very small and falling well before leaf expansion (Fig. 19B). **Leaves** imparipinnate, petiole and leaf rachis dorsally flattened and sulcate, the rachis extending beyond the distal leaflet, 7–13-foliolate, leaflets alternate, mostly elliptical, pinnately veined, secondary veins brochidrodromous; stipels setiform, small, caducous (Fig. 19B); extrafloral nectaries absent. **Inflorescence** bracteate, amentiform, densely multi-flowered, short racemes, these isolated or grouped in short axillary clusters (Fig. 19C, D). **Flowers** small (ca. 3–5 mm diam), actinomorphic, bisexual or unisexual (staminate), pedicellate, greenish-yellow (Fig. 19E); hypanthium absent; sepals 5, free; petals 5, free, imbricate, reflexed; stamens 10, free; pollen grains in monads, 3-colporate, exine psilate (Soares et al. 2020); intrastaminal disk short with an undulate rim; ovary shortly stipitate, pubescent, narrowly winged along the adaxial margin at the distal half, the style laterally attached near the apex of the ovary, staminate flowers lacking a gynoecium or provided with a pistilode composed by an ovary but lacking a style. **Fruit** a wind-dispersed one-seeded samara, flat compressed, straw coloured, stipitate; seed-chamber strongly veined with a small beak (style remnant) laterally displaced near the apex; wing paleaceous (Fig. 19F). **Seed** oblong, flat compressed, with a punctiform terminal hilum (Fig. 19G).

#### Chromosome number.

2*n* = 20 (Bandel 1974; Melloni 2010).

#### Included species and geographic distribution.

**Figure 20.**
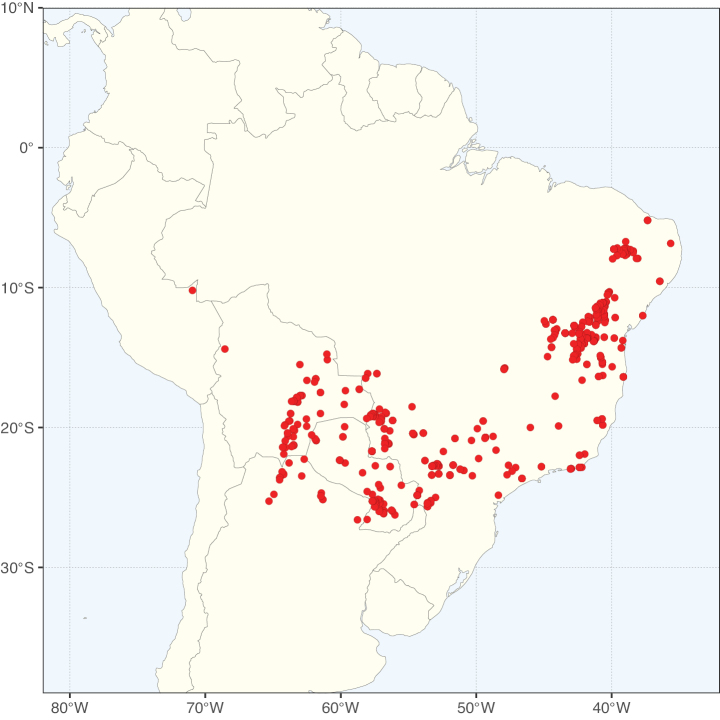
Distribution of *Pterogyne* based on quality-controlled digitised herbarium records. See Suppl. material 1 for the source of occurrence data.

Monospecific (*P.nitens*), distributed across north-eastern, eastern and central Brazil, northern Argentina and Paraguay and south-eastern Bolivia (Fig. 20).

#### Ecology.

*Pterogynenitens* is a tree of the South American tropical and subtropical seasonally dry forests of north-eastern Brazil (Caatinga), central Brazil (Cerrado) and chaquean forests (northern Argentina, Paraguay and southern Bolivia), also occurring in eastern Brazilian semi-deciduous forests of the Mata Atlântica. It occurs mostly as a pioneer tree colonizing degraded areas. It flowers in a very short period and the flowers are visited by a wide range of small insects (Pfeiffer 2018).

#### Etymology.

From *pteros* (Greek: πτέρυξ = wing) and *gynos*- (Greek: γυνή = woman, gynoecium) referring to the ovary provided with a narrow wing along one margin.

#### Human uses.

*Pterogynenitens* is planted as an urban tree (Fig. 19A). Its silica rich wood is rated as moderately durable in terms of decay resistance and easy to work both by hand and machine tools. It can be used for flooring, furniture, cabinetry, interior trim and turned objects (Lewis 2005b; Meier 2021). It is a fast growing tree occurring in soils of low natural fertility, and has been recommended for planting in riparian forest with periodic flooding, and resetting and restoration of degraded areas (Mauro et al. 2008). Preparations from the stem bark have been used by local Paraguayan populations in the therapy of ascariasis (Crivos et al. 2007). Several guanidine alkaloids with cytotoxic activity have been isolated from leaves and fruits (Regasini et al. 2009). Leaves are reported as toxic to livestock.

#### Notes.

The phylogenetic position of the genus is still uncertain and conflicting among different analyses (see Fig. 18 and discussion above).

The compact catkin-like racemes with rather small flowers and the samara fruits are similar to those found in the African-Asiatic genus *Pterolobium* R. Br. ex Wight & Arn. of tribe Caesalpinieae (Gagnon et al. 2016; see Caesalpinieae tribe, page 103), but *Pterogyne* is an unarmed tree with imparipinnate leaves and alternate leaflets, the ovary has a uniquely winged margin along the distal half and the style attachment is displaced to a subterminal position, while *Pterolobium* has branches armed with prickles, the leaves are bipinnate with opposite leaflets, the ovary is unwinged and the style is attached at the apex. The samara wing of *Pterogyne* is clearly derived from the expansion of the marginal wing of the ovary below the style. The style remains as a beak on the fruit and is laterally displaced above the seed-chamber (Polhill and Vidal 1981).

#### Taxonomic references.

Lewis (2005b); Polhill and Vidal (1981); Queiroz (2009); Tulasne (1843).

## ﻿﻿5. Tribe Cassieae

Juliana Gastaldello Rando^37^, Matheus Martins Teixeira Cota^2^, Alexandre Gibau de Lima^8,28^, Roseli Lopes da Costa Bortoluzzi^6^, Brigitte Marazzi^30^, Adilva de Souza Conceição^11^

Citation: Rando JG, Cota MMT, Lima AG, Bortolozzi RLC, Marazzi B, Conceição AS (2024) 5. Tribe Cassieae In: Bruneau A, Queiroz LP, Ringelberg JJ (Eds) Advances in Legume Systematics 14. Classification of Caesalpinioideae. Part 2: Higher-level classification. PhytoKeys 240: 83–102. https://doi.org/10.3897/phytokeys.240.101716

### ﻿Tribe Cassieae

Taxon classificationPlantaeFabalesFabaceae

Bronn, Form. Pl. Legumin.: 130. 1822.

Figs 21 , 22 , 23 , 24 , 25 , 26 , 27 , 28 , 29 , 30 , 31 , 32 , 33

Cassiaceae Vest, Anleit. Stud. Bot.: 270, 291. 1818. Type: Cassia L.
Irregulares Bronn, Form. Pl. Legumin.: 13. 1822. Type: Cassia L.
Cassiinae Wight & Arn., Prodr. Fl. Ind. Orient.: 280. 1834. Type: Cassia L.
Cassioideae Burmeist., Handb. Naturgesch.: 319. 1837. Type: Cassia L.
Melanoxyleae Nakai, Chosakuronbun Mokuroku [Ord. Fam. Trib. Nov.]: 254. 1943. Type: Melanoxylum Schott

#### Type.

*Cassia* L.

#### Included genera

**(7).***Batesia* Spruce ex Benth. (1 species), *Cassia* L. (39), *Chamaecrista* (L.) Moench (361), *Melanoxylum* Schott (1), *Recordoxylon* Ducke (3), *Senna* Mill. (287), *Vouacapoua* Aubl. (3).

#### Description.

Trees, shrubs, subshrubs or vines. **Stipules** diverse in shape and size, persistent or caducous. **Leaves** bifoliolate, paripinnate and/or imparipinnate, extrafloral nectaries present in *Batesia* and *Vouacapoua*, and in several species of *Chamaecrista* and *Senna*, on the petiole and/or leaf rachis, and/or peduncles of inflorescences; leaflets opposite, subopposite or alternate. **Inflorescences** racemes or panicles, terminal, axillary and sometimes ramiflorous. **Flowers** hypogynous or perigynous, bilaterally symmetrical, radially symmetrical or asymmetrical; sepals 5, free; petals 5, free, equal, subequal or differentiated; stamens 2–10, free, homomorphic or heteromorphic, anthers poricidal (*Cassia*, *Chamaecrista* and *Senna*) or longitudinally dehiscent; pollen unknown for most genera, 3-colporate with long apertures in *Chamaecrista* and *Senna*; ovary sessile or stipitate. **Fruit** a legume, follicle, indehiscent or dehiscent, with valves opening elastically or not. **Seeds** mostly compressed, exarillate, variable in shape and colour.

#### Distribution.

The tribe has a pantropical distribution in wet forests, seasonally dry forests and woodlands, savannas and deserts, a few species extending to temperate areas. The highest diversity of the richest genera (*Chamaecrista* and *Senna*) occurs in the Neotropical region.

#### Clade-based definition.

**Figure 21.**
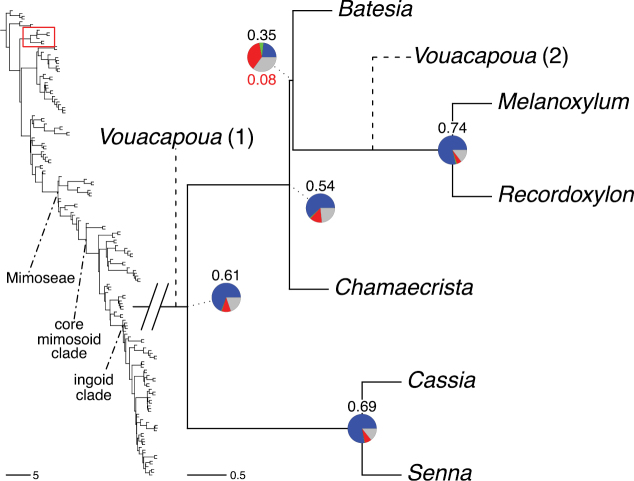
Generic relationships in tribe Cassieae. Two most likely positions of the unsampled genus *Vouacapoua* are indicated with dashed lines: 1) as sister to all Cassieae [following Bruneau et al. (2008) and Marazzi and Sanderson (2010)], and 2) as sister to *Melanoxylum* and *Recordoxylon* [following Manzanilla and Bruneau (2012) and Kates et al. (2024)]. For description of phylogeny and support values, see Fig. 6 caption (page 63).

The most inclusive crown clade containing *Cassiafistula* L. and *Melanoxylumbrauna* Schott, but not *Ceratoniasiliqua* L., *Dimorphandraconjugata* (Splitg.) Sandwith or *Mimosasensitiva* L. (Fig. 21).

#### Notes.

Tribe Cassieae sensu Irwin and Barneby (1981) was divided into five subtribes which included a total of 20 genera: Cassiinae Wight & Arn. (3 genera), Ceratoniinae H.S. Irwin & Barneby (1), Dialiinae H.S. Irwin & Barneby (13), Duparquetiinae H.S. Irwin & Barneby (1) and Labicheinae H.S. Irwin & Barneby (2). However, ever since the first molecular phylogenies, the tribe has never been supported as monophyletic (Doyle et al. 1997, 2000; Bruneau et al. 2001, 2008; Kajita et al. 2001; LPWG 2017) and all recent analyses support as monophyletic what was then considered subtribe Cassiinae (comprising *Cassia*, *Chamaecrista* and *Senna*), but only with the inclusion of four other genera previously placed in the Peltophorum group of tribe Caesalpinieae by Polhill and Vidal (1981) (e.g., Haston et al. 2003, 2005; Bruneau et al. 2008; Manzanilla and Bruneau 2012; Ringelberg et al. 2022).

**Figure 22.**
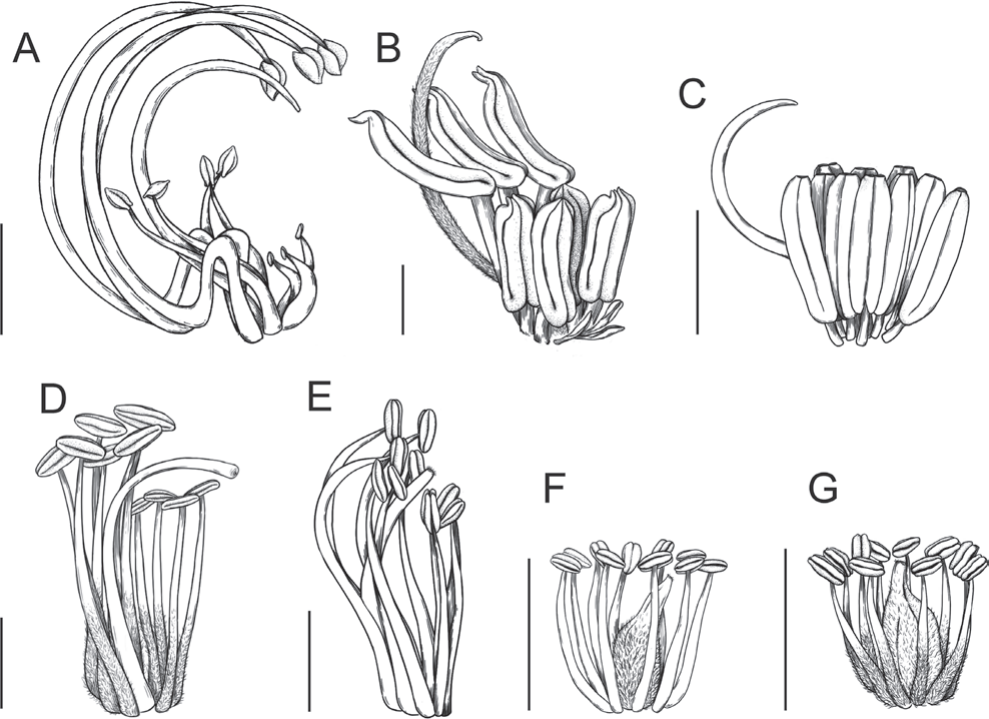
Examples of variation in stamens and pistil among Cassieae genera **A***Cassiamoschata* Kunth (*Rando et al. 1184*) **B***Sennamacranthera* (DC. ex Collad.) H.S. Irwin & Barneby (*Rando et al. 272*) **C***Chamaecristabahiae* (H.S. Irwin) H.S. Irwin & Barneby (*Rando et al. 1214*) **D***Melanoxylumbrauna* Schott (*Cardoso et al. 2439*) **E***Recordoxylonspeciosum* (Benoist) Gazel ex Barneby (*Pereira-Silva et al. 15631*) **F***Vouacapouaamericana* Aubl (*Nascimento 245*). **G***Batesiafloribunda* Spruce ex Benth (based on photographs of Projeto Flora Reserva Ducke, INPA/DFID, comm. Mike Hopkins). Scale: 1 cm. Drawn by Najla M.B. Scheidegger / @ilustre.nt.

*Cassia*, *Chamaecrista* and *Senna* are the largest genera in the tribe. Because of similarity in floral morphology, they were all treated under the single genus *Cassia* until they were segregated by Irwin and Barneby (1981) based on floral, mainly in the androecium configuration (Fig. 22), and fruit differences. The recognition of *Cassia*, *Chamaecrista* and *Senna* is now well established and each of the three genera is supported as monophyletic in well-sampled phylogenetic analyses (Marazzi et al. 2006; Conceição et al. 2009; Rando et al. 2016; LPWG 2017; Souza et al. 2021). Although the three genera were traditionally considered closely related, phylogenetic analyses have shown that they do not form a clade (Bruneau et al. 2008; LPWG 2017), *Chamaecrista* often being resolved as more closely related to *Batesia*, *Melanoxylum* and *Recordoxylon* (Fig. 21).

The position of *Vouacapoua* has proved more difficult to resolve. Even though not sampled by Ringelberg et al. (2022; Fig. 21), it is included here in Cassieae based on other phylogenetic analyses. Haston et al. (2005), who sampled two of the three species of *Vouacapoua*, found the genus to be not monophyletic, but this unexpected result was due to a sampling error [the sequence used [*Andiraaubletii* (=*Vouacapouaamericana*), GenBank accession AY899701.1] is from *Connarusconchocarpus* F. Muell (Connaraceae)]. In the LPWG (2017)*matK* analysis that includes all three species, the genus is supported as monophyletic and in all phylogenetic analyses, *Vouacapoua* is resolved as part of, or as sister, to the Cassieae clade (Bruneau et al. 2008; Marazzi and Sanderson 2010; Manzanilla and Bruneau 2012; LPWG 2017; Kates et al. 2024).

Cassieae is a morphologically heterogeneous group, with only a few features shared among all or nearly all genera. All Cassieae have once-pinnate or bifoliolate leaves, most species have yellow petals, with only a few having red, orange, pink or white ones. *Cassia*, *Senna* and *Chamaecrista* are characterised by poricidal anthers (Fig. 22). These three genera exhibit a suite of floral traits associated with the specialised pollination mode called buzz pollination, in which bees vibrate flowers to release pollen through the anther pores or slits (Gottsberger and Silberbauer-Gottsberger 1988). Recent phylogenies, which do not group these three genera together, suggest this specialised pollination mode likely evolved more than once within Cassieae. Another unusual feature is that some Cassieae species have stomata on both leaflet surfaces, otherwise present in only a few other caesalpinioid legume clades (Herendeen et al. 2003a; Bruneau et al. 2008). Extrafloral nectaries are reported in *Batesia*, *Chamaecrista*, *Senna* and *Vouacapoua* but are lacking in *Cassia*, *Melanoxylum* and *Recordoxylon* (Marazzi et al. 2019; Cota 2020a). Although in Caesalpinioideae the ability to nodulate rarely occurs outside tribe Mimoseae, two Cassieae genera, *Chamaecrista* and *Melanoxylum*, are known to nodulate (Sprent 2001; Faria et al. 2022). Not all species of *Chamaecrista* are known to nodulate and those that do differ in the type of nodule anatomy (Faria et al. 2022; Casaes et al. 2023).

### ﻿ Vouacapoua

Taxon classificationPlantaeFabalesFabaceae

Aubl., Hist. Pl. Guiane 2 (Suppl.): 9, pl. 373. 1775.

Figs 22 , 23 , 24

#### Type.

*Vouacapouaamericana* Aubl.

#### Description.

Unarmed trees. **Stipules** not observed. **Leaves** spiral, imparipinnate; petiole terete; extrafloral nectaries on pulvinus or between all pairs of leaflets, sessile, secretory surface convex, sometimes absent; leaflets 7–11, opposite. **Inflorescence** a panicle; bract 1, caducous, bracteoles 2, caducous. **Flowers** perigynous, radially symmetrical; hypanthium campanulate; sepals 5, free; petals 5, yellow, free; stamens 10, free, filaments glabrous, anthers longitudinally dehiscent; pollen unknown; ovary shortly stipitate, attached to the base of the hypanthium. **Fruit** obovoid to ellipsoid drupaceous legume, rugose and velutinous, one (rarely 2–3)-seeded, swollen over seed, dehiscent. **Seeds** globose to obovoid, with a brownish, smooth and glossy testa.

#### Chromosome number.

Unknown.

#### Included species and geographic distribution.

Three species, *V.americana*, *V.macropetala* Sandwith and *V.pallidior* Ducke, restricted to northern South America in the wet Amazonian forests of Brazil, British Guiana, French Guiana and Suriname (Ducke 1932a; Sandwith 1937; Cota 2020c; Fig. 24).

**Figure 23.**
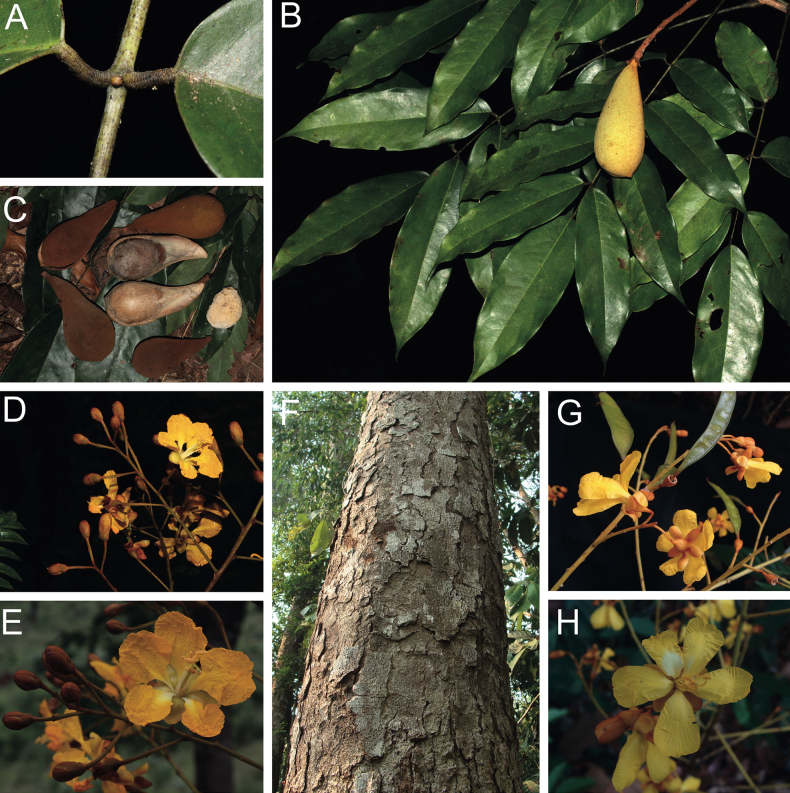
Examples of *Batesia*, *Melanoxylum*, *Recordoxylon* and *Vouacapoua* diversity **A***Batesiafloribunda* Spruce ex Benth. extrafloral nectary, petiolules and part of leaflets (*Cota 1158*) **B, C***Vouacapouaamericana* Aubl. (*Cardoso et al. 3450*) **B** leaves and immature fruit **C** mature fruits and seeds on the ground **D, E***Melanoxylumbrauna* Schott (*Cardoso et al. 2439*) **D** inflorescence, buds and flowers **E** flower **F–H***Recordoxylonspeciosum* (Benoist) Gazel ex Barneby (*Pereira-Silva et al. 15631*) **F** trunk of mature individual **G** flowers and immature fruit **H** flower. Photo credits **A–E** D Cardoso **F–H** G Pereira-Silva.

**Figure 24.**
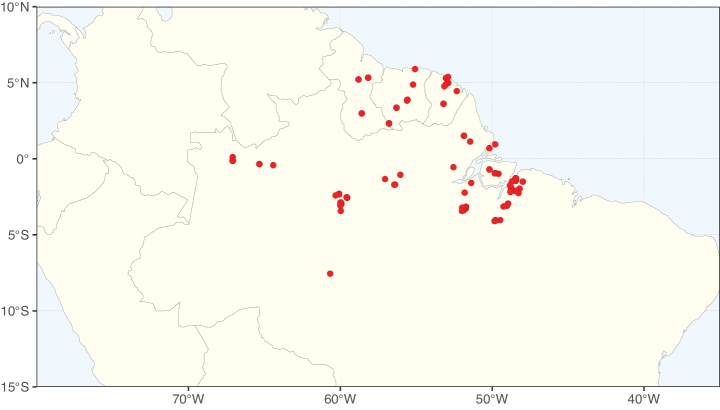
Distribution of *Vouacapoua* based on quality-controlled digitised herbarium records. See Suppl. material 1 for the source of occurrence data.

#### Ecology.

The genus is known only on well-drained soils from lowland tropical rainforests of the Amazon basin (“terra firme”).

#### Human uses.

The timber of *V.americana* is used in civil and naval constructions and furniture (Lewis 2005b).

#### Etymology.

The name originated with the Galibi (also called Kalinã), indigenous people from northern South America. “Vouacapoua” or “Voicapou” are Galibi names for *Vouacapouaamericana* (Aublet 1775; Mari-Mut 2018).

#### Notes.

*Vouacapoua* is characterised by a terete petiole, extrafloral nectaries on the pulvinus or between the pairs of leaflets, glabrous stamen filaments (Fig. 22), an obovoid to ellipsoid legume, swollen over the (mostly) single seed, and seeds globose to obovoid with a brownish testa. In the original description, *Vouacapoua* was described as having three bracteoles on the pedicels, but from observation of herbarium specimens, we certify that there are two bracteoles and one bract. *Vouacapoua* has extrafloral nectaries on leaves and flowers that are radially symmetrical, a characteristic also seen in *Batesia*. However, *Batesia* differs from *Vouacapoua* by having winged petioles, stamens with a villous filament base, a follicle rather than a drupaceous legume, and seeds with an orangish to reddish testa.

Seeds of *V.americana* are dispersed by small rodents (*Myoproctaexilis* and *Dasyproctaleporina*) that bury the fruits at short distances from the source tree (Forget 1990, 1994). Their fragrant flowers are pollinated by small bees (Apidae, Halictidae and Anthophoridae) and flies (Syrphidae; Maués et al. 1999).

#### Taxonomic references.

Aublet (1775); Cota (2020c); Ducke (1932a); Lewis (2005b); Sandwith (1937).

### ﻿ Batesia

Taxon classificationPlantaeFabalesFabaceae

Spruce ex Benth., Gen. Pl. 1: 563. 1865.

Figs 22 , 23 , 25

#### Type.

*Batesiafloribunda* Spruce ex Benth.

#### Description.

Unarmed trees. **Stipules** not observed. **Leaves** spiral, imparipinnate or, rarely, paripinnate; extrafloral nectaries present between the proximal pair of leaflets, sometimes also between the distal ones, secretory surface flat and disc shaped; petiole narrowly winged; leaflets 9–13, opposite. **Inflorescence** a panicle; bract 1, caducous, bracteoles 2, caducous. **Flowers** perigynous, radially symmetrical; hypanthium campanulate; sepals 5, free; petals 5, yellow, free; stamens 10, homomorphic, filaments villous at the base, anthers longitudinally dehiscent; pollen unknown; ovary shortly stipitate, attached to the base of the hypanthium. **Fruit** an ellipsoid to oblong-obovate follicle, turgid and slightly compressed, the valves fleshy-coriaceus with strongly raised veins, dehiscent. **Seeds** globose with a reddish and smooth testa.

#### Chromosome number.

Unknown.

#### Included species and geographic distribution.

**Figure 25.**
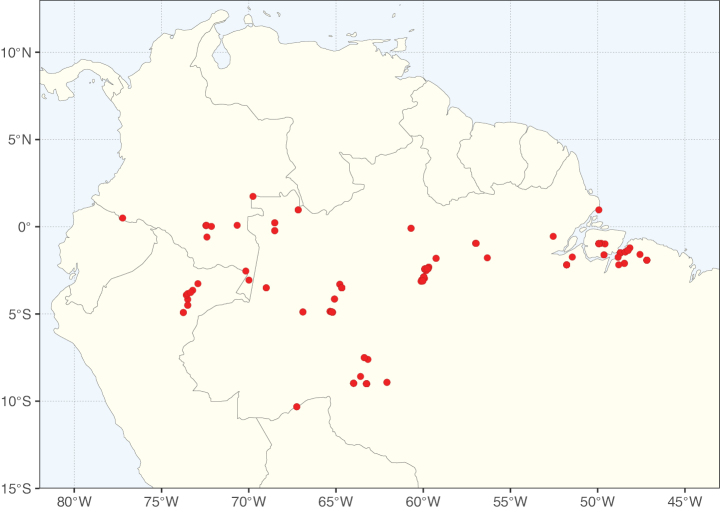
Distribution of *Batesia* based on quality-controlled digitised herbarium records. See Suppl. material 1 for the source of occurrence data.

Monospecific (*B.floribunda*), occurring in northern and north-western South America in the wet Amazonian forests of Brazil, French Guiana, Colombia and Peru (Fig. 25).

#### Ecology.

The genus is known only on well-drained soils from lowland tropical rainforests of the Amazon basin (‘terra firme”).

#### Human uses.

The timber of *B.floribunda* is used in the construction of fine furniture (Lewis 2005b).

#### Etymology.

Named after the English naturalist Henry Walter Bates, who explored the Amazon rainforests with A.R. Wallace in the 19^th^ century (Lewis 2005b).

#### Notes.

*Batesia* is characterised by the presence of winged leaf petioles, extrafloral nectaries between the leaflet pairs, and mainly by the fruit follicle with bright red seeds. In the original description, *Batesia* was described with three bracteoles on the pedicels, but observation of herbarium specimens lead us to the conclusion that there are two bracteoles and one bract. Herbarium specimens (vegetative or with fruits and seeds) of *B.floribunda* are often misidentified as *Ormosia* Jacks. (Papilionoideae). However, *Ormosia* displays terete leaf petioles, lacks extrafloral nectaries, and has a distinct fruit. The relationship of *Batesia*, in particular to *Chamaecrista* and a clade that groups *Recordoxylon* and *Melanoxylum*, remains poorly understood and requires further study (Fig. 21).

#### Taxonomic references.

Bentham (1865, 1866); Cota (2020b); Ducke (1949); Lewis (2005b).

### ﻿ Melanoxylum

Taxon classificationPlantaeFabalesFabaceae

Schott in K.F.A.von Schreibers, Nachr. Österr. Naturf. Bras. 2(Anh.): 52. 1822.

Figs 22 , 23 , 26

Melanoxylon Schott, Syst. Veg., ed. 16 [Sprengel] 4(2): Cur. Post. 406. 1827, orth. var.
Perittium Vogel, 1837. Linnaea 11: 408. 1837. Type: Perittiumferrugineum Vogel [= Melanoxylumbrauna Schott]

#### Type.

*Melanoxylumbrauna* Schott

#### Description.

Unarmed trees, bark thick. **Stipules** caducous. **Leaves** spiral, imparipinnate; extrafloral nectaries absent; leaflets 11–21, opposite to subopposite. **Inflorescence** a panicle; bracts and bracteoles caducous. **Flowers** perigynous, bilaterally symmetrical; hypanthium infundibuliform; sepals 5, free; petals 5, yellow, free, clawed, glabrous; stamens 10, slightly heteromorphic, filaments ferruginous tomentose at the base, anthers longitudinally dehiscent; pollen unknown; ovary 11–13-ovulate, ferruginous tomentose. **Fruit** an oblong, slightly curved, compressed legume, dehiscing through both margins, valves with raised transverse ribs, tomentose, endocarp breaking up into one seeded transversely oblong envelopes. **Seeds** oblong-depressed, with smooth, opaque and dark reddish testa.

#### Chromosome number.

Unknown.

#### Included species and geographic distribution.

**Figure 26.**
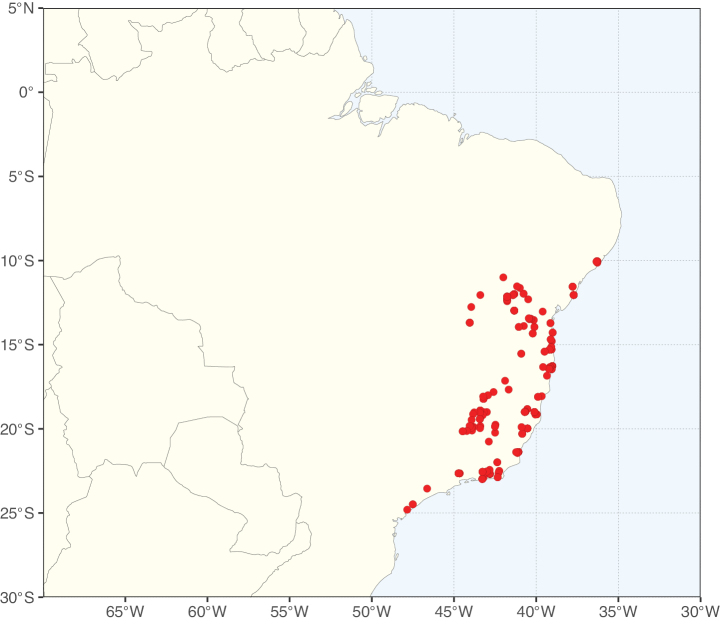
Distribution of *Melanoxylum* based on quality-controlled digitised herbarium records. See Suppl. material 1 for the source of occurrence data.

Monospecific (*M.brauna*), restricted to Brazil, occurring predominantly along the eastern Brazilian coast, but entering the interior in drier vegetations (Fig. 26).

#### Ecology.

*Melanoxylumbrauna* occurs preferentially in wet habitats, mostly in tropical rainforests; its occurrence in drier Brazilian vegetation (seasonally deciduous and semi-deciduous Forests) is apparently associated with wetter areas within these ecosystems.

#### Human uses.

The timber of *M.brauna* is largely used in the construction of fine furniture, and the bark is a source of tannin for medicinal purposes (Lewis 2005b).

#### Etymology.

*Melano* + *xylon* from Greek meaning “black” and “wood”, respectively. The name is related to the black heartwood of the plant (Lewis 2005b).

#### Notes.

*Melanoxylum* is characterised by its imparipinnate leaves, and by its dense inflorescence and characteristic fruit, an oblong legume, slightly curved with articulate endocarp, breaking up into transversely oblong parts.

#### Taxonomic references.

Lewis (2005b); Queiroz (2009); Rando et al. (2020a).

### ﻿ Recordoxylon

Taxon classificationPlantaeFabalesFabaceae

Ducke, Trop. Woods 39: 16. 1934.

Figs 22 , 23 , 27

#### Type.

*Recordoxylonamazonicum* (Ducke) Ducke [≡ *Melanoxylonamazonicum* Ducke (= *Recordoxylonspeciosum* (Benoist) Gazel ex Barneby)]

#### Description.

Unarmed trees. **Stipules** not observed. **Leaves** spiral, imparipinnate; leaflets 7–15, alternate to opposite; extrafloral nectaries absent. **Inflorescence** a panicle; bract 1, caducous, bracteoles 2, caducous. **Flowers** perigynous, bilaterally symmetrical, hypanthium campanulate; sepals 5, free; petals 5, the innermost petal yellow with white spot at the base, the others yellow, free, clawed; stamens 10, slightly heteromorphic, filaments glabrous, anthers longitudinally dehiscent; pollen unknown; ovary shortly stipitate, attached to the base of the hypanthium. **Fruit** oblong, straight, compressed legume, with a longitudinal rib close to the superior margin, dehiscent. **Seeds** elliptic-depressed, with a brownish and rugose testa.

#### Chromosome number.

Unknown.

#### Included species and geographic distribution.

**Figure 27.**
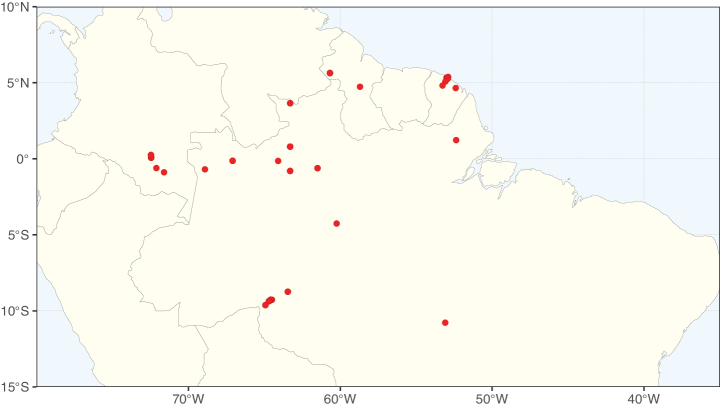
Distribution of *Recordoxylon* based on quality-controlled digitised herbarium records. See Suppl. material 1 for the source of occurrence data.

Three species, *R.pulcherrimum* Barneby, *R.speciosum*, and *R.stenopetalum* Ducke, restricted to northern South America in the wet Amazonian forests of Brazil, Guyana, French Guiana and Venezuela (Ducke 1949; Barneby 1993; Cota 2020a; Fig. 27).

#### Ecology.

The genus is known from lowland tropical rainforests of the Amazon basin, on well-drained soils (“terra firme”) to poorly drained soils (“igapó” forest).

#### Etymology.

Named after the American botanist Samuel James Record, an important wood anatomist who observed some wood structures in *R.speciosum* (as *Melanoxylumamazonicum*; Record 1932) that led Adolpho Ducke to revise the original classification and create the new genus *Recordoxylon* (Ducke 1932a, 1934).

#### Notes.

*Recordoxylon* is characterised by its bilaterally symmetrical flowers, clawed petals, glabrous stamen filaments, longitudinally dehiscent anthers (Fig. 22), straight legumes and elliptic-depressed seeds with rugose and brownish testa. Morphologically similar to *Melanoxylum*, they can be differed by the filament indument (ferruginous-tomentose at the base in *Melanoxylum* vs. glabrous in *Recordoxylon*) and fruit morphology (slightly curved and endocarp articulated vs. straight and endocarp not articulated, respectively). The two genera are always resolved as sister taxa when they are both included in phylogenetic analyses (Haston et al. 2005; Bruneau et al. 2008; Marazzi and Sanderson 2010; Manzanilla and Bruneau 2012; LPWG 2017; Ringelberg et al. 2022).

#### Taxonomic references.

Barneby (1993); Ducke (1932a, 1934, 1949); Record (1932).

### ﻿ Chamaecrista

Taxon classificationPlantaeFabalesFabaceae

(L.) Moench, Methodus: 272. 1794.

Figs 22 , 28 , 29

Cassia [infragen. unranked] Chamaecrista L., Sp. Pl. 1: 379. 1753. Type: Cassiachamaecrista L., nom. utique rejic. [≡ Cassiafasciculata Michx. (≡ Chamaecristafasciculata (Michx.) Greene)]
Cassia sect. Chamaecrista (L.) DC., Hist. Nat. Méd. Casses 24, 118.1816. Lectotype (designated by Britton and Rose 1930): Cassianictitans L. [≡ Chamaecristanictitans (L.) Moench]
Sooja Siebold, Verh. Batav. Genootsch. Kunst. 12: 56. 1830. Type: Soojanomame Siebold, nom. inval. (nom. nud.) [≡ Cassiamimosoidesvar.nomame Makino (≡ Chamaecristanomame (Makino) H. Ohashi)]
Disterepta Raf., Sylva Tellur.: 126. 1838. Type: Distereptapilosa (L.) Raf. [≡ Cassiapilosa L. (≡ Chamaecristapilosa (L.) Greene)]
Hepteireca Raf., Sylva Tellur.: 126. 1838. Type: Hepteirecaglandulosa (L.) Raf. [≡ Cassiaglandulosa L. (≡ Chamaecristaglandulosa (L.) Greene)]
Dialanthera Raf., Sylva Tellur.: 127. 1838. Type: Dialantheraglandulosa (L.) Raf. [≡ Cassiaglandulosa L. (≡ Chamaecristaglandulosa (L.) Greene)]
Xamacrista Raf., Sylva Tellur.: 127. 1838. Type: Xamacristatrifolia Raf. [= Cassiachamaecrista L. (≡ Chamaecristafasciculata (Michx.) Greene)]
Nictitella Raf., Sylva Tellur.: 128. 1838. Lectotype (designated by Irwin & Barneby, 1982): Nictitellaamena Raf. [= Cassianictitans L. (≡ Chamaecristanictitans (L.) Moench)]
Ophiocaulon Raf., Sylva Tellur.: 129. 1838. Lectotype (designated by Irwin & Barneby, 1982): Ophiocaulonserpens (L.) Raf. [≡ Cassiaserpens L. (≡ Chamaecristaserpens (L.) Greene)]
Cassia subg. Lasiorhegma Vogel ex Benth., Fl. Bras. 15(2): 129. 1870. Type not designated.

#### Type.

*Chamaecristanictitans* (L.) Moench [≡ *Cassianictitans* L.]

#### Description.

Trees, treelets, shrubs and subshrubs, lacking spines or prickles. **Stipules** diverse in shape and size, persistent or caducous. **Leaves** distichous or spiral, bifoliolate or paripinnate; extrafloral nectaries when present on petiole, generally on the rachis between the pairs of leaflets or in the axis of the inflorescence, sessile or stipitate, the secretory surface concave, rarely convex; leaflets 1–65 pairs. **Inflorescence** a fascicle, raceme or panicle; bract 1, caducous or persistent, bracteoles 2, alternate, located at mid-length or slightly above the pedicels, persistent. **Flowers** hypogynous, asymmetrical, hypanthium absent; sepals 5, free; petals 5, free, yellow, or yellow with red base, sometimes red, orange or pink; stamens 5–10, homomorphic, filaments glabrous, anthers dehiscent by apical pores, pubescent laterally, rarely with the indumentum covering the entire anther; pollen subprolate to prolate, syncopate, fused at the poles; ovary stipitate. **Fruit** a compressed legume, valves papyraceous or coriaceous, elastically dehiscent through both margins, becoming twisted after dehiscence. **Seeds** variable in shape and colour.

#### Chromosome number.

Haploid numbers *n* = 7, 8, 14, 16, 24 (Goldblatt and Johnson 1979–; Souza 2004).

#### Included species and geographic distribution.

**Figure 28.**
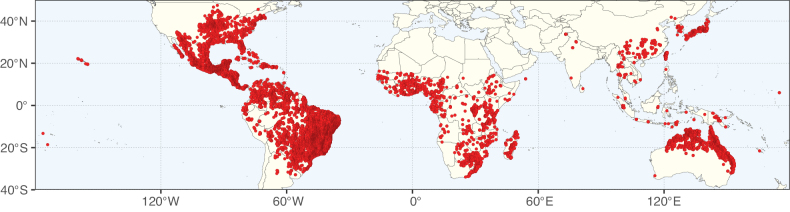
Distribution of *Chamaecrista* based on quality-controlled digitised herbarium records. See Suppl. material 1 for the source of occurrence data.

368 species, pantropical with a few species reaching temperate areas (Fig. 28).

#### Ecology.

*Chamaecrista* species typically occur in open environments. Although several species are widespread, such as *C.rotundifolia* (Pers.) Greene, *C.mimosoides* (L.) Greene and *C.flexuosa* (L.) Greene, a high diversity is concentrated in Brazilian savannas and in the “campos rupestres’’ vegetation (Irwin and Barneby 1978, 1982; Rando et al. 2020b). In these centers of diversity, several species have evolved underground systems that allow survival after fire and during long dry periods (Rando et al. 2016). One clade of arborescent *Chamaecrista* species is mostly restricted in the Amazon and Atlantic tropical rainforests.

**Figure 29.**
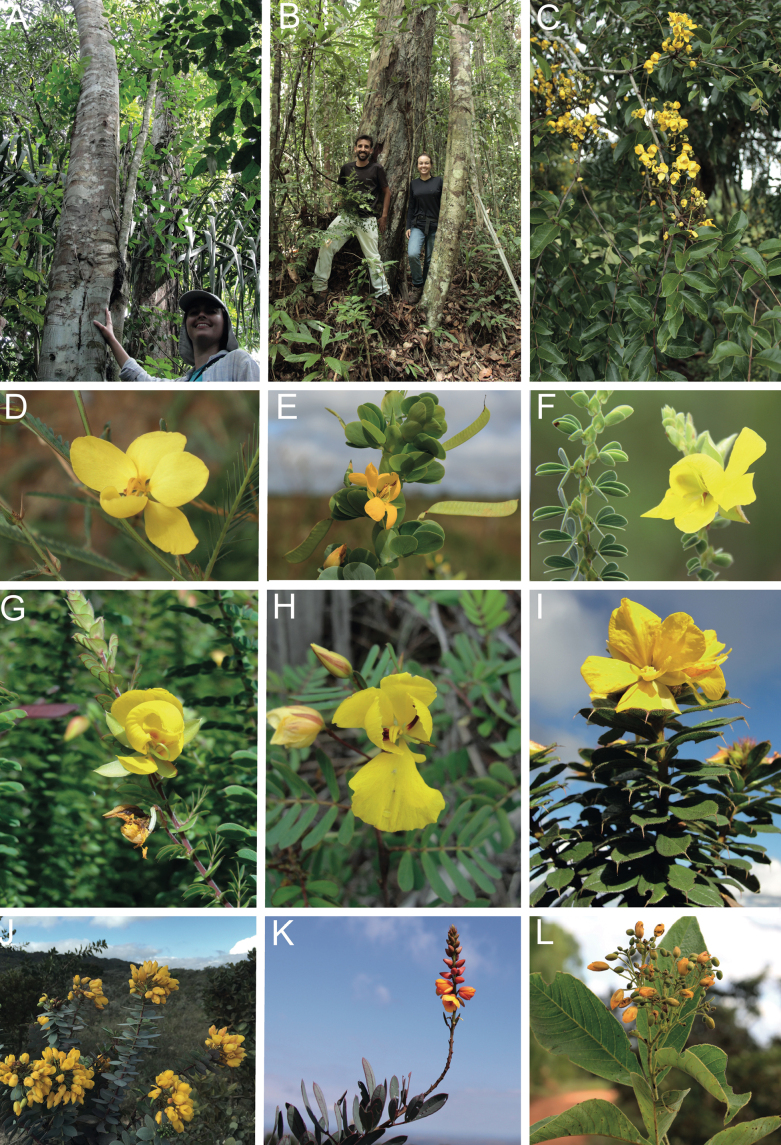
Examples of *Chamaecrista* diversity **A***Chamaecristaxinguensis* (Ducke) H.S. Irwin & Barneby trunk of mature individual (*Rando et al. 1208*) **B***C.compitalis* (H.S. Irwin & Barneby) H.S. Irwin & Barneby base of trunk of mature individual (*Rando et al. 1364*) **C***C.ensiformis* (Vell.) H.S. Irwin & Barneby flowering branch (*Rando & Cota 1366*) **D***C.flexuosa* (L.) Greene flower, leaves in background **E**C.desvauxiivar.latistipula (Benth.) G.P. Lewis, branch with flowers and fruit **F**C.ramosavar.curvifolia (Vogel) G.P. Lewis branches and flower **G***C.distichoclada* (Benth.) H.S. Irwin & Barneby flowering branch (*Rando et al. 1230*) **H***C.lineata* (Sw.) Greene leaves and flower (*Rando 964*) **I***C.andromedea* (Mart. ex Benth.) H.S. Irwin & Barneby branch with leaves and flowers (*Rando et al. 1251*) **J***C.vauthieri* (Benth.) H.S. Irwin & Barneby flowering branches (*Cardoso et al. 4096*) **K**C.ochnaceavar.purpurascens (Benth.) H.S. Irwin & Barneby inflorescence and leaves **L***C.scabra* (Pohl ex Benth.) H.S. Irwin & Barneby leaves and inflorescence (*Rando et al. 1266*). Photo credits **A, C, G–I** JG Rando **B** JG Jardim **D–F, K** H Moreira **J** D Cardoso **L** MF Simon.

#### Human uses.

Some species are used in traditional African medicine. For example, *C.absus* (L.) H.S. Irwin & Barneby is used as a purgative, for treating wounds and sores, and also against syphilis (Lewis 2005a). In China and Japan, *C.mimosoides* is used as a tea, and in Tanzania, against snake bites and scorpion stings (Lewis 2005a). In Brazil, dried leaflets and branches of some species [*C.choriophylla* (Vogel) H.S. Irwin & Barneby, *C.cotinifolia* (G.Don) H.S. Irwin & Barneby, *C.orbiculata* (Benth.) H.S. Irwin & Barneby and *C.rotundata* (Vogel) H.S. Irwin & Barneby] are used as decorative objects (Cota et al. 2020).

#### Etymology.

A composite name from the Greek *Chamae* (= small, of little growth), and the Latin *crista*, referring to the crest (Rizzini and Rizzini 1983; Radcliffe-Smith 1998). The name was applied in reference to the very short filaments of stamens forming a crest (Greene 1905).

#### Notes.

The largest genus of the tribe can be easily recognised by a set of features: the presence of two bracteoles on the pedicels, stamens generally homomorphic, poricidal anthers (Fig. 22) and elastically dehiscent fruits. Since the segregation of *Chamaecrista* from *Cassia*, the infrageneric classification of the genus remains the subject of intensive studies, changing the rank, expanding, restricting, or combining the names within the genus (e.g., Bentham 1871; Irwin 1964; Irwin and Rogers 1967; Irwin and Barneby 1976a, 1976b, 1977, 1978, 1982; Rando et al. 2016; Souza et al. 2021). In short, three highly supported clades with strong correlation in habit and habitat variation are currently well established in *Chamaecrista*: (i) a clade of arborescent species, with ramiflorous inflorescences and extrafloral nectaries; (ii) a clade of shrubs, with axillary and reduced racemes, distichous phyllotaxy, and with extrafloral nectaries; and (iii) the most diverse clade, embracing shrubs with terminal racemes or panicles, spiral phyllotaxy, without extrafloral nectaries, and commonly with glandular trichomes on branches, leaves and inflorescences (Conceição et al. 2009; Souza et al. 2021). Within these groups there are also some highly supported clades, consistent with morphology, and the trend has been to improve their circumscription to arrive at a stable and practical infrageneric classification. However, more molecular phylogenetic studies are needed to clarify relationships in the most diverse clades (ca. 200 species in savannas and rocky fields), in which recent diversification (ca. 5 Ma; Rando et al. 2016; Vasconcelos et al. 2020) complicates the understanding of relationships among species.

#### Taxonomic references.

Bentham (1871); Buril et al. (2011); Cota et al. (2020); Irwin (1964); Irwin and Barneby (1976a, 1976b, 1977, 1978, 1981, 1982); Irwin and Rogers (1967); Rando et al. (2016, 2020b); Souza et al. (2021).

### ﻿ Cassia

Taxon classificationPlantaeFabalesFabaceae

L., Sp. Pl.: 376. 1753.

Figs 22 , 30 , 31

Cathartocarpus Pers., Syn. Pl. 1: 459. 1805. Type: Cathartocarpusfistula (L.) Pers. [≡ Cassiafistula L.]
Bactyrilobium Willd., Enum. Pl.: 439. 1809. Type: Bactyrilobiumfistula (L.) Willd. [≡ Cassiafistula L.]
Cassiana Raf., Amer. Monthly Mag. & Crit. Rev. 1: 266. 1818. Type not designated.
Mac-leayia Montrouz., Mém. Acad. Imp. Sci. Lyon, Sect. Sci., sér. 2, 10: 198. 1860. Type: Mac-leayiamultiflora Montrouz. [= Cassiaartensis Beauvis.]

#### Type.

*Cassiafistula* L.

#### Description.

Trees or shrubs, lacking spines or prickles. **Stipules** 0.1–1.0 cm, in general caducous. **Leaves** distichous or spiral, paripinnate; extrafloral nectaries absent; leaflets 2–25 pairs, opposite. **Inflorescence** an axillary raceme; bract 1, caducous or persistent, bracteoles 2, at the base of pedicels, usually caducous. **Flowers** hypogynous, bilaterally symmetrical, hypanthium solid, turbinate or conic; sepals 5, free, reflexed at anthesis; petals 5, yellow or pink, less often red, white or mixed, the median petal a different colour from the rest; stamens 10, heteromorphic, 3 with long sigmoidal filaments and longitudinally dehiscent anthers, 7 adaxial ones varying in length, organised in groups of 5+2 or 4+3, anthers with basal poricidal dehiscence; pollen unknown; ovary shortly stipitate, attached to the base of the hypanthium. **Fruit** a linear-oblong, often long, cylindrical or quadrangular, woody, indehiscent legume (except in *C.hintoni* Sandwith). **Seeds** obovoid to ellipsoid, compressed, smooth and glossy with castaneous or brownish testa.

**Figure 30.**
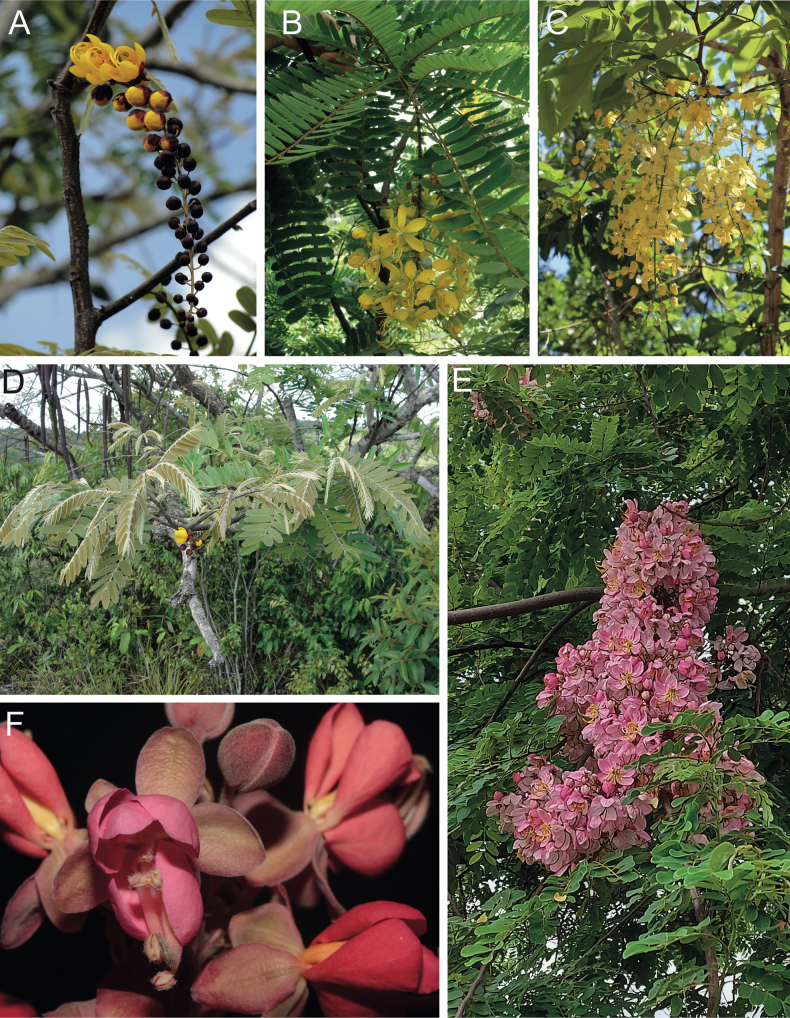
Examples of *Cassia* diversity **A***Cassiamoschata* Kunth inflorescence **B***C.ferruginea* (Schrad.) Schrad. ex DC. branch showing the spiraled leaves and an inflorescence (*Rando et al. 125*) **C***C.fistula* L. branch with leaves and an inflorescence **D***C.moschata* branches with leaves, an inflorescence and fruits (left corner) in the background (*Rando et al. 1184*) **E***C.javanica* L. branches with leaves and an inflorescence **F***C.grandis* L.f. flowers. Photo credits **A–D** JG Rando **E** D Gissi **F** D Cardoso.

#### Chromosome number.

Haploid numbers *n* = 12, 13, 14 (Goldblatt and Johnson 1979–).

#### Included species and geographic distribution.

**Figure 31.**
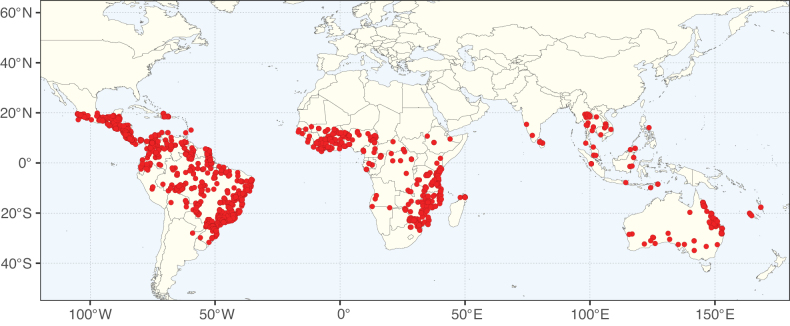
Distribution of *Cassia* based on quality-controlled digitised herbarium records. See Suppl. material 1 for the source of occurrence data.

Thirty-nine species (LPWG 2022), pantropical with two main centres of diversity, in the Neotropical region (13 species), mainly in the Amazon and Atlantic Forest, and in sub-Saharan Africa (10 species). Other species occur in South and South East Asia and in Oceania (Fig. 31).

#### Ecology.

*Cassia* occurs preferentially in tropical rainforests; a few species extend to or occur exclusively in temperate areas, such as *C.ferruginea* Schrad. ex DC. and *C.leptophylla* Vogel (Queiroz 2009; Scheidegger and Rando 2020).

#### Human uses.

Many uses are reported for several *Cassia* species as medicinal plants (Lewis 2005a). *Cassiafistula*, *C.grandis* L.f., *C.javanica* L. and *C.roxburghii* DC. are widely cultivated ornamentals across tropical and subtropical regions owing to the beautiful flowers and dense inflorescences (Scheidegger and Rando 2020).

#### Etymology.

Derived from the ancient Greek name *casia* for the aromatic and fragrant plants (Lewis 2005a).

#### Notes.

*Cassia* is characterised by showy dense inflorescences, flowers with an androecium with 3 long sigmoidal stamens with longitudinally dehiscent anthers and 7 adaxial stamens varying in length, organised in groups of 5+2 or 4+3, and these poricidal at the base of the thecae (instead of apically as in *Chamaecrista* and *Senna*) (Fig. 22). The fruits are also characteristic: indehiscent, woody, and cylindrical or quadrangular. There is no comprehensive phylogeny for the genus: fewer than 10 taxa have been sequenced, and mostly for different loci. Thus, the relationship among species, and the biogeography and morphological evolution, are poorly known.

#### Taxonomic references.

Irwin and Barneby (1982); Lewis (2005a); Queiroz (2009); Scheidegger and Rando (2020).

### ﻿ Senna

Taxon classificationPlantaeFabalesFabaceae

Mill., Gard. Dict. Abr. (ed. 4). 1754.

Figs 22 , 32 , 33

Chamaecassia Link, Handbuch 2: 139. 1831. Type: Chamaecassialaevigata (Willd.) Link [≡ Cassialaevigata Willd. (= Sennaseptemtrionalis (Viv.) H.S. Irwin & Barneby)]
Chamaefistula (DC. ex Collad.) G. Don, Gen. Hist. 2: 106. 1832. Type: Chamaefistulacorymbosa (Lam.) G. Don [≡ Cassiacorymbosa Lam. (≡ Sennacorymbosa (Lam.) H.S. Irwin & Barneby)]
Adipera Raf., Sylva Tellur.: 129. 1838. Type: Adiperaherbertiana (Lindl.) Raf. [= Senna×floribunda (Cav.) H.S. Irwin & Barneby]
Diallobus Raf., Sylva Tellur.: 128. 1838. Type: Diallobustora (L.) Raf. [≡ Cassiatora L. (≡ Sennatora (L.) Roxb.)]
Ditremexa Raf., Sylva Tellur.: 127. 1838. Lectotype (designated by Britton and Wilson 1924): Ditremexaoccidentalis (L.) Britton & Rose [≡ Cassiaoccidentalis L. (≡ Sennaoccidentalis (L.) Link)]
Emelista Raf., Sylva Tellur.: 127. 1838. Type: Emelistaobtusifolia (L.) Raf. [≡ Cassiaobtusifolia L. (≡ Sennaobtusifolia (L.) H.S. Irwin & Barneby)]
Herpetica Raf., Sylva Tellur.: 123. 1838. Type: Herpeticaalata (L.) Raf. [≡ Cassiaalata L. (≡ Sennaalata (L.) Roxb.)]
Isandrina Raf., Sylva Tellur.: 126. 1838. Type: Isandrinaarborescens Raf. [= Sennaatomaria (L.) H.S. Irwin & Barneby]
Panisia Raf., Sylva Tellur.: 128. 1838. Type: Panisiabiflora (L.) Raf. [≡ Cassiabiflora L., but typus not established, probably either = Sennapallida (Vahl) H.S. Irwin & Barneby *sensu lato* or S.angustisiliqua (Lam.) H.S. Irwin & Barneby]
Peiranisia Raf., Sylva Tellur.: 127. 1838. Type: Peiranisiaaversiflora (Herb.) Raf. [≡ Cassiaaversiflora Herb. (≡ Sennaaversiflora (Herb.) H.S. Irwin & Barneby)]
Scolodia Raf., Sylva Tellur.: 128. 1838. Type: Scolodiaviminea (L.) Raf. [≡ Cassiaviminea L. (≡ Sennaviminea (L.) H.S. Irwin & Barneby)]
Cassia subg. Senna (Mill.) Benth., Fl. Bras. 15(2): 83, 96. 1870. Type not cited, but inferred as Sennaalexandrina Mill.
Chamaesenna Raf. ex Pittier, Arb. Arbus. Orden Legum.: 130. 1928. Lectotype (designated by Britton and Rose 1930): Chamaesennareticulata (Willd.) Pittier [≡ Cassiareticulata Willd. (≡ Sennareticulata (Willd.) H.S. Irwin & Barneby)]
Cowellocassia Britton, N. Amer. Fl. 23: 251. 1930. Type: Cowellocassiascleroxyla (Britton) Britton [≡ Cassiascleroxyla Britton (= Sennadomingensis (Spreng.) H.S. Irwin & Barneby)]
Earleocassia Britton, N. Amer. Fl. 23: 247. 1930. Type: Earleocassiaroemeriana (Scheele) Britton [≡ Cassiaroemeriana Scheele (≡ Sennaroemeriana (Scheele) H.S. Irwin & Barneby)]
Echinocassia Britton & Rose, N. Amer. Fl. 23: 251. 1930. Type: Echinocassiaaculeata (Pohl ex Benth.) Britton & Rose [≡ Cassiaaculeata Pohl ex Benth. (≡ Sennaaculeata (Pohl ex Benth.) H.S. Irwin & Barneby)]
Desmodiocassia Britton & Rose, N. Amer. Fl. 23: 244. 1930. Type: Desmodiocassiavillosa (Mill.) Britton & Rose [≡ Cassiavillosa Mill. (≡ Sennavillosa (Mill.) H.S. Irwin & Barneby]
Gaumerocassia Britton, N. Amer. Fl. 23: 252. 1930. Type: Gaumerocassiaperalteana (Kunth) Britton [≡ Cassiaperalteana Kunth (≡ Sennaperalteana (Kunth) H.S. Irwin & Barneby)]
Leonocassia Britton, N. Amer. Fl. 23: 268. 1930. Type: Leonocassiastenophylla (Benth.) Britton [≡ Cassiastenophylla Benth. (≡ Sennastenophylla (Benth.) H.S. Irwin & Barneby)]
Palmerocassia Britton, N. Amer. Fl. 23: 253. 1930. Type: Palmerocassiawislizeni (A. Gray) Britton [≡ Cassiawislizeni A. Gray (≡ Sennawislizeni (A. Gray) H.S. Irwin & Barneby)]
Phragmocassia Britton & Rose, N. Amer. Fl. 23: 245. 1930. Type: Phragmocassiaskinneri (Benth.) Britton & Rose [≡ Cassiaskinneri Benth. (≡ Sennaskinneri (Benth.) H.S. Irwin & Barneby)]
Pseudocassia Britton & Rose, N. Amer. Fl. 23: 230. 1930. Lectotype (designated by Irwin and Barneby 1982): Pseudocassiaspectabilis (DC.) Britton & Rose [≡ Cassiaspectabilis DC. (≡ Sennaspectabilis (DC.) H.S. Irwin & Barneby)]
Pterocassia Britton & Rose, N. Amer. Fl. 23: 243. 1930. Type: Pterocassiagaleottiana (M. Martens) Britton & Rose [≡ Cassiagaleottiana M. Martens (≡ Sennagaleottiana (M. Martens) H.S. Irwin & Barneby)]
Tharpia Britton & Rose, N. Amer. Fl. 23: 246. 1930. Type: Tharpiapumilio (A. Gray) Britton & Rose [≡ Cassiapumilio A. Gray (≡ Sennapumilio (A. Gray) H.S. Irwin & Barneby)]
Vogelocassia Britton, N. Amer. Fl. 23: 258. 1930. Type: Vogelocassialeiophylla (Vogel) Britton [≡ Cassialeiophylla Vogel (≡ Sennaleiophylla (Vogel) H.S. Irwin & Barneby)]
Xerocassia Britton & Rose, N. Amer. Fl. 23: 246. 1930. Type: Xerocassiaarmata (S. Watson) Britton & Rose [≡ Cassiaarmata S.Watson (≡ Sennaarmata (S. Watson) H.S. Irwin & Barneby)]

#### Type.

*Sennaalexandrina* Mill.

#### Description.

Trees, treelets, erect or scandent shrubs, subshrubs, vines, rarely aphyllous shrubs with cladode-like branches, unarmed or rarely with spines or prickles. **Stipules** diverse, caducous or persistent, rarely with embedded extrafloral nectary tissue. **Leaves** distichous or spiral, bifoliolate or paripinnate, rarely absent or reduced to a petiolar phyllode; extrafloral nectaries when present, on the petiole and/or on the leaf rachis, sessile or stipitate, the secretory surface convex; leaflets 1–many pairs, opposite. **Inflorescences** racemes or panicles; bract 1, persistent or caducous, bracteoles absent. **Flowers** hypogynous, bilaterally symmetrical or asymmetrical; hypanthium absent; sepals 5, free; petals 5, free, yellow; androecium comprising 3 adaxial staminodes and (4) 6–7 heteromorphic stamens, grouped in 2 sets of 4 median and (0) 2–3 abaxial stamens, rarely all 10 stamens fertile and homomorphic, filaments glabrous, anthers apically poricidal; pollen prolate-spheroidal to prolate, not syncopate; ovary stipitate. **Fruit** an indehiscent legume, a follicle or a legume dehiscing through both margins, linear or oblong, cylindrical, laterally compressed or tetragonal, fleshy or dry. **Seeds** variable in shape and colour.

#### Chromosome number.

Haploid chromosome numbers *n* = 11, 12, 13, 14 and 28 (Goldblatt and Johnson 1979–), but *n* = 14 is the most frequent. A single accession of *S.rugosa* (G.Don) H.S. Irwin & Barneby was found to be tetraploid with *n* = 28 (Biondo et al. 2005b), in contrast to Coleman and Demenezes (1980) who reported *n* = 14 for this species.

#### Included species and geographic distribution.

287 species (LPWG 2022), most species distributed on the American continent, occurring in tropical, subtropical and (few) temperate areas (Irwin and Barneby 1982; Marazzi et al. 2006). Some species also distributed in Africa, Oceania and Asia (Lock 1988; Du Puy 1995; Randell 1988; Randell and Barlow 1998; Fig. 33).

**Figure 32.**
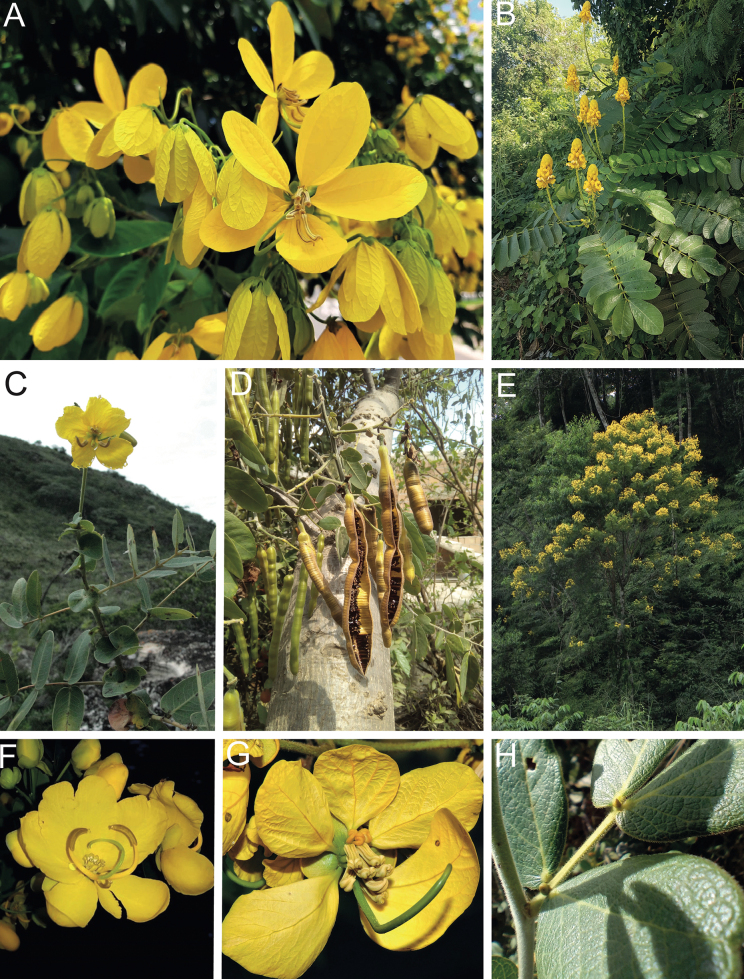
Examples of *Senna* diversity **A***Sennamacranthera* (DC. ex Collad.) H.S. Irwin & Barneby flowering branch (*Lima et al. 422*) **B***Sennaalata* (L.) Roxb. flowering branch **C***Sennacorifolia* (Benth.) H.S. Irwin & Barneby flowering branch (*Rando et al. 936*) **D***Sennaangulata* (Vogel) H.S. Irwin & Barneby fruits **E***Sennamultijuga* (Rich.) H.S. Irwin & Barneby mature individual (*Lima 408*) **F***Sennapendula* (Humb.& Bonpl. ex Willd.) H.S. Irwin & Barneby flower (*Lima et al. 435*) **G***Sennaspectabilis* (DC.) H.S. Irwin & Barneby flower (unvouchered) **H***Sennarugosa* (G. Don) H.S. Irwin & Barneby leaf with extrafloral nectaries between leaflets (*Lima et al. 538*). Photo credits **A, B, E, F, H** A Lima **C** JG Rando **D** F Logan **G** RT Queiroz https://rubens-plantasdobrasil.blogspot.com/.

**Figure 33.**
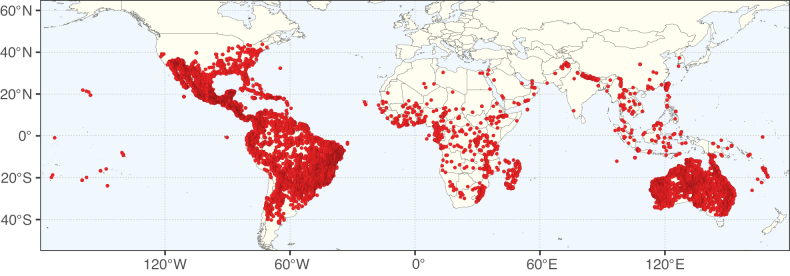
Distribution of *Senna* based on quality-controlled digitised herbarium records. See Suppl. material 1 for the source of occurrence data.

#### Ecology.

*Senna* occurs in a wide range of habitats, including wet forests, seasonally deciduous forests, seasonally semi-deciduous forests, savannas, deserts and also anthropised areas (Irwin and Barneby 1982).

#### Human uses.

Species of *Senna* are used in traditional and conventional medicine, as bee forage for honey, ornamentals and for timber. Seeds are roasted and ground as a coffee substitute (Miller 1754; Lewis 2005a).

#### Etymology.

The name *Senna* derives from the Arabic “sana” or “sanna” which refers to plants with cathartic properties (Miller 1754; Lewis 2005a).

#### Notes.

*Senna* is morphologically characterised by the paripinnate leaves, extrafloral nectaries present in many species (on leaves, stipules, bracts, and sepals; leaf nectaries with convex secretory surface, the others are embedded and externally not clearly visible), yellow petals, heteromorphic androecium usually with 6–7 fertile stamens (Fig. 22), indehiscent or dehiscent fruits, and valves not twisted after dehiscence. Irwin and Barneby (1982) recognised an infrageneric classification with six sections and 35 series within *Senna*. In the following years, new taxonomic revisions of *Senna* for Australia (Randell 1988, 1989, 1990) and Asia (Singh 2001) recognised new taxa, including three new series, totalling 38 series within *Senna*. Phylogenetic studies based on morphological and molecular data corroborated the monophyly of *Senna* (Bruneau et al. 2001; Herendeen et al. 2003a; Marazzi et al. 2006) although five of the six sections and part of the 38 series were not resolved as monophyletic (Marazzi et al. 2006). The only monophyletic section, Sennasect.Psilorhegma (Vogel) H.S. Irwin & Barneby, comprises species characterised by having all stamens fertile (i.e., a synapomorphy of this clade).

Phylogenies have consistently retrieved seven strongly supported clades supported by floral morphological features and presence or absence of extrafloral nectaries (Marazzi et al. 2006; Marazzi and Sanderson 2010). Species of the clade sister to the rest of the genus are characterised by symmetrically bilateral flowers, as also found in one of the more derived clades, in which monosymmetry evolved secondarily, with all other clades characterised by variably weakly or strongly asymmetric (enantiostylous) flowers (Marazzi and Endress 2008).

*Senna* is outstanding within legumes with respect to its specialisation in pollination mode. This specialisation is expressed especially in the androecium in which the diversity pertains to patterns of heteranthery and anther elaborations, including dehiscence patterns, pointing direction of the pores, and extension of the lateral furrow (Marazzi et al. 2007). Indeed, it can be regarded as the genus with the greatest androecial diversity among Cassieae. Although extrafloral nectaries are absent in two clades of the tribe, recently described stipular nectaries with the secretory tissue embedded within the tissue are a synapomorphy for another clade (Marazzi et al. 2013), and all other species, which are grouped together in a clade, have the well-known specialised leaf nectaries.

Molecular dating analyses (Marazzi and Sanderson 2010) indicate that *Senna* originated in the early Eocene and the lineages with specialised extrafloral nectaries evolved in the late Eocene, after the main radiation of ants. These extrafloral nectaries represent a relatively old key innovation in *Senna*, in which the association with ants provides protection to the plants, and may have promoted the colonisation of new habitats appearing with the early uplift of the Andes (Marazzi et al. 2006, 2013; Marazzi and Sanderson 2010).

#### Taxonomic references.

Bortoluzzi et al. (2020); Du Puy 1995; Irwin and Barneby (1982); Lima et al. (2023); Lock (1988); Miller (1754); Queiroz (2009); Randell (1988, 1989, 1990); Randell and Barlow (1998); Singh (2001).

## ﻿﻿6. Tribe Caesalpinieae

Edeline Gagnon^16,17,18^, Ruth P. Clark^10^, Jens J. Ringelberg^3,4^, Gwilym P. Lewis^10^

Citation: Gagnon E, Clark RP, Ringelberg JJ, Lewis GP (2024) 6. Tribe Caesalpinieae. In: Bruneau A, Queiroz LP, Ringelberg JJ (Eds) Advances in Legume Systematics 14. Classification of Caesalpinioideae. Part 2: Higher-level classification. PhytoKeys 240: 103–145. https://doi.org/10.3897/phytokeys.240.101716

### ﻿Tribe Caesalpinieae

Taxon classificationPlantaeFabalesFabaceae

Rchb., Fl. Germ. Excurs. 2(2): 544. 1832.

Figs 34 , 35 , 36 , 37 , 38 , 39 , 40 , 41 , 42 , 43 , 44 , 45 , 46 , 47 , 48 , 49 , 50 , 51 , 52 , 53 , 54 , 55 , 56 , 57 , 58 , 59 , 60 , 61 , 62 , 63 , 64 , 65

Poincianeae Nakai, Chosakuronbun Mokuroku [Ord. Fam. Trib. Nov.]: 253. 1943. Type: Poinciana L. [= Caesalpinia L.]

#### Type.

*Caesalpinia* L.

#### Included genera

**(27).***Arquita* Gagnon, G.P. Lewis & C.E. Hughes (5 species), *Balsamocarpon* Clos (1), *Biancaea* Tod. (6), *Caesalpinia* L. (9), *Cenostigma* Tul. (15), *Cordeauxia* Hemsl. (1), *Coulteria* Kunth (11), *Denisophytum* R. Vig. (8), *Erythrostemon* Klotzsch (31), *Gelrebia* Gagnon & G.P. Lewis (8), *Guilandina* L. (up to 20), *Haematoxylum* L. (5), *Hererolandia* Gagnon & G.P. Lewis (1), *Hoffmannseggia* Cav. (23), *Hultholia* Gagnon & G.P. Lewis (1), *Libidibia* (DC.) Schltdl. (7), *Lophocarpinia* Burkart (1), *Mezoneuron* Desf. (24), *Moullava* Adans. (4), *Paubrasilia* Gagnon, H.C. Lima & G.P. Lewis (1), *Pomaria* Cav. (16), *Pterolobium* R. Br. ex Wight & Arn. (10), *Stenodrepanum* Harms (1), *Stuhlmannia* Taub. (1), *Tara* Molina (3), *Ticanto* Adans. (9), *Zuccagnia* Cav. (1).

#### Description.

Trees, shrubs, subshrubs, or herbs, sometimes scandent, often with prickles, thorns, glands, or glandular hairs. **Stipules** (best seen on young flush foliage and on seedlings) variable across the tribe, ranging from minute, lanceolate-deltate to triangular, ovate, or orbicular, sometimes foliaceous, the margins sometimes ciliate-fimbriate, persistent, caducous, or apparently lacking (at least on mature leaves). **Leaves** pinnate or bipinnate. **Inflorescences** terminal and/or axillary racemes or panicles; bracteoles absent; pedicels often jointed. **Flowers** zygomorphic, or rarely almost actinomorphic; hypanthium usually present (rarely a short calyx tube); sepals generally free to hypanthium-rim, the lowermost sepal modified, often forming a hood (cucullate) over the other four sepals in bud, imbricate to valvate; petals 5, the median (innermost) petal usually clearly differentiated; stamens 10, all similar, the filaments usually hairy, especially basally, and sometimes glandular, anthers mostly dorsifixed, introrse; pollen tricolporate monads, mostly spherical, surface reticulate and with granular-membraned margos surrounding weakly developed colpi (a margocolpus); ovary subsessile to short-stipitate, stigma usually crateriform. **Fruits** diverse, 1–several-seeded. **Seeds**, flattened, or globose.

#### Distribution.

The tribe is pantropical, found predominantly in seasonally dry tropical forests and shrublands, but extending in a subset of clades into tropical and warm temperate savannas, tropical wet forests, and tropical coastal habitats (Gagnon et al. 2019).

#### Clade-based definition.

**Figure 34.**
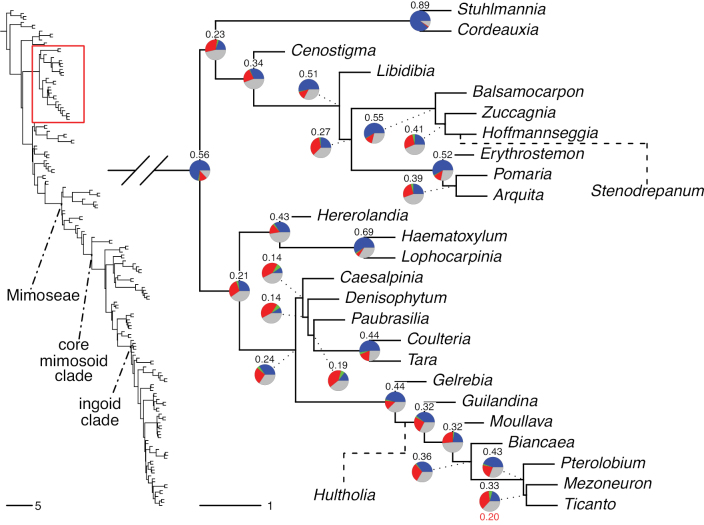
Generic relationships in tribe Caesalpinieae. The most likely positions of the unsampled genera *Stenodrepanum* and *Hultholia* are indicated with dashed lines [following Gagnon et al. (2019)]. For description of phylogeny and support values, see Fig. 6 caption (page 63).

The most inclusive crown clade containing *Caesalpiniabrasiliensis* L. and *Erythrostemongilliesii* (Hook.) Klotzsch, but not *Cassiafistula* L., *Dimorphandraconjugata* (Splitg.) Sandwith or *Mimosasensitiva* L. (Fig. 34).

#### Notes.

The tribe comprises ca. 223 species (the number of species of *Guilandina* is unresolved) in 27 genera. The tribe Caesalpinieae was first described by Reichenbach (1832). Bentham (1865: 562) recognised the Caesalpinieae as a suborder comprising 12 tribes, one of which, the Eucaesalpinieae, included 16 genera, six of which are retained in the present circumscription of tribe Caesalpinieae. Bentham (1865: 566) divided the genus *Caesalpinia* into ten sections, six of which have become additional genera currently recognised in tribe Caesalpinieae (*Guilandina*, *Erythrostemon*, *Pomaria*, *Balsamocarpon*, *Coulteria* and *Libidibia*). Polhill and Vidal (1981) divided the tribe into eight informal generic groups, one of which, the Caesalpinia group, comprised 16 genera. The Caesalpinia group was defined by Polhill and Vidal (1981) to include genera with species that have a large variety of glandular trichomes, prickles and spines as a defense mechanism, and possessing zygomorphic flowers with a somewhat modified lower sepal and stamens crowded around the pistil. This informal Caesalpinia group remained largely intact in the account of Caesalpinieae by Lewis (2005b), although a handful of additional segregate genera were recognised from within the genus *Caesalpinia* sensu lato, and three genera of Polhill and Vidal’s (1981) group were transferred to the core Peltophorum group [*Conzattia* Rose, *Lemuropisum* H. Perrier (now a synonym of *Delonix* Raf.) and *Parkinsonia* L., now tribe Schizolobieae; see page 146], so that the Caesalpinia group then comprised 21 genera. Studies by Gagnon et al. (2013, 2015) demonstrated the non-monophyly of some of these 21 genera and Gagnon et al. (2016), based on molecular phylogenetic analyses and a robust species-level sampling, published a new generic system for the pantropical Caesalpinia group. Gagnon et al.’s (2016)Caesalpinia group clade is strongly supported by the study of Ringelberg et al. (2022; Fig. 34) and is here reinstated as tribe Caesalpinieae in which the type genus *Caesalpinia* is nested.

**Figure 35.**
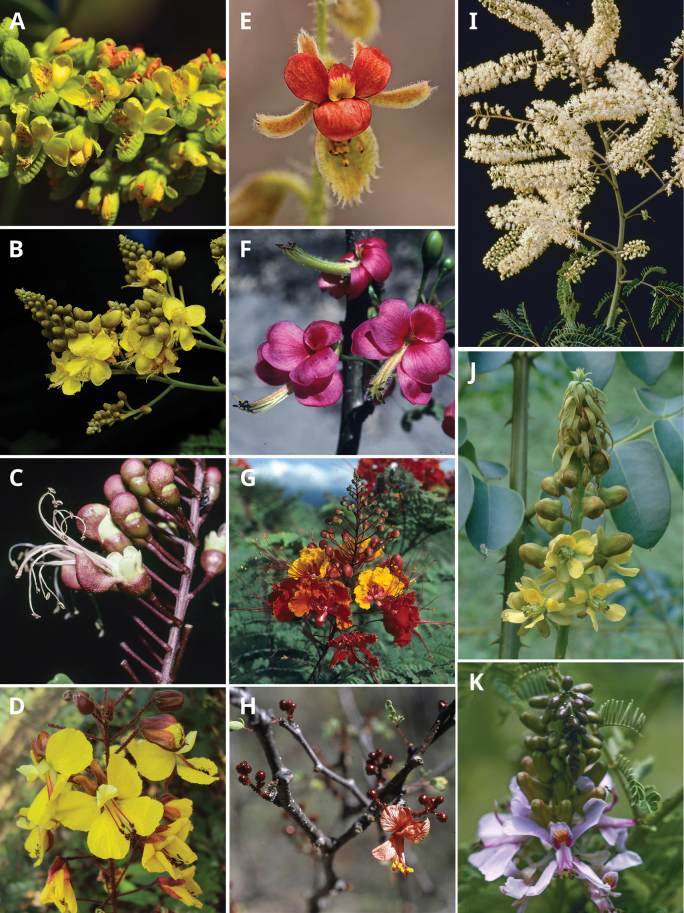
Flowers of Caesalpinieae**A***Taraspinosa* Britton & Rose, Peru, Ancash (*Hughes et al. 3043*) **B**Cenostigmapluviosum(DC.)Gagnon & G.P. Lewisvar.pluviosum, Bolivia, Santa Cruz (*Wood et al. 26552*) **C***Caesalpiniabahamensis* Lam., Cuba (*Lewis 1853*) **D***Hultholiamimosoides* (Lam.) Gagnon & G.P. Lewis, India **E***Pomariaburchellii* (DC.) B.B. Simpson & G.P. Lewis, Botswana Ghanzi district **F***Erythrostemoncoccineus* (G.P. Lewis & J.L. Contr.) Gagnon & G.P. Lewis, Mexico, Oaxaca (*Lewis et al. 1802*) **G***Caesalpiniapulcherrima* (L.) Sw., Honduras **H***Erythrostemonmelanadenius* (Rose) Gagnon & G.P. Lewis, Mexico, Oaxaca (*Hughes et al. 2091*) **I***Pterolobiumstellatum* (Forssk.) Brenan, Africa **J***Guilandinabonduc* L., India **K***Gelrebiatrothaei* (Harms) Gagnon & G.P. Lewis, Tanzania. Photo credits **A** E Gagnon **B, F–H** CE Hughes **C** GP Lewis **D** VR Vinayaraj, India Biodiversity Portal (https://indiabiodiversity.org/group/wild_orchids_of_india/observation/show/335155), the basionym of *Hultholiamimosoides***E** O Bourquin, Flora of Zimbabwe (https://www.zimbabweflora.co.zw/speciesdata/image-display.php?species_id=127200&image_id=6) **I** P Van Wyk **J** M Sanjappa **K** PJ Cribb.

**Figure 36.**
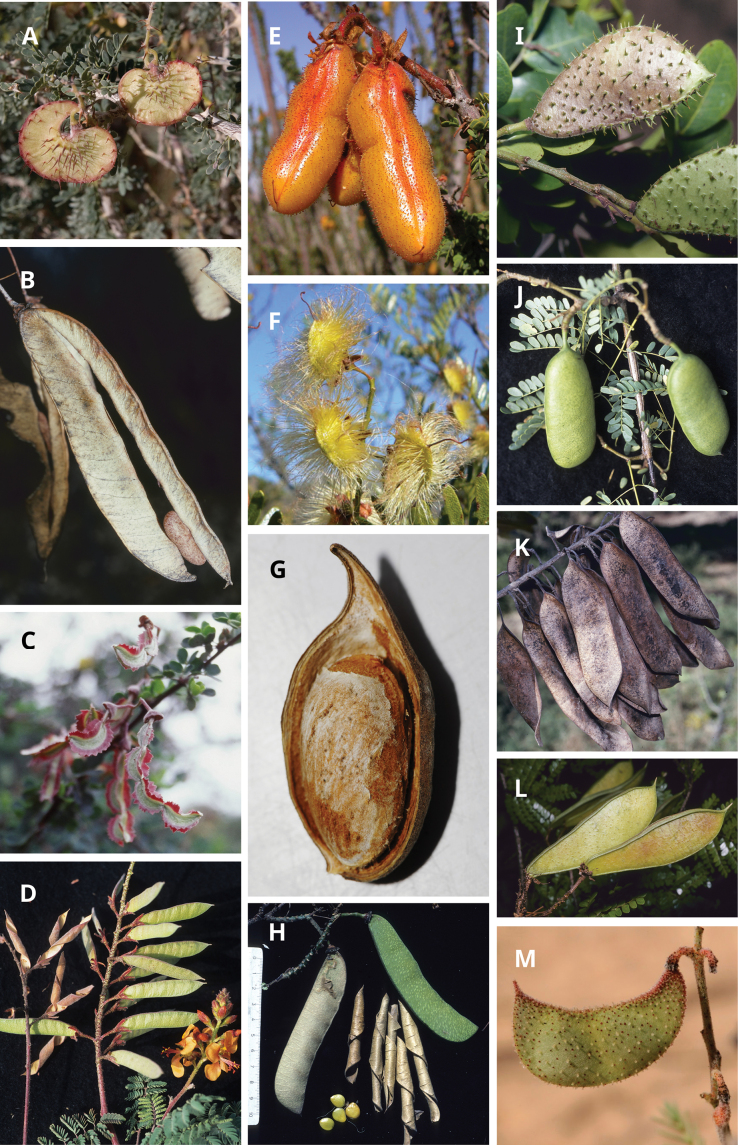
Fruits of Caesalpinieae**A***Hererolandiapearsonii* (L. Bolus) Gagnon & G.P. Lewis, Namibia, Sesriem Canyon **B***Haematoxylumbrasiletto* H. Karst., Mexico (*Lewis 2057*) **C***Lophocarpiniaaculeatifolia* (Burkart) Burkart, Paraguay, (*Fortunato 8650*) **D***Hoffmannseggiaarequipensis* Ulibarri, Peru, Arequipa, (*Hughes et al. 2342*) **E***Balsamocarponbrevifolium* Clos, Chile (*Baxter et al. DCI 1859*) **F***Zuccagniapunctata* Cav., Argentina, Mendoza **G***Cordeauxiaedulis* Hemsl., Somalia **H***Erythrostemoncoccineus* (G.P. Lewis & J.L. Contr.) Gagnon & G.P. Lewis, Mexico, Oaxaca (*Lewis et al. 1802*) **I***Paubrasiliaechinata* (Lam.) Gagnon, H.C. Lima & G.P. Lewis, Brazil **J***Libidibiaparaguariensis* (Parodi) G.P. Lewis, Bolivia, Santa Cruz (*Hughes 2475*) **K***Coulteriamollis* Kunth, Guatemala (*Lewis et al. 1714*) **L**Cenostigmapluviosumvar.cabralianum (G.P. Lewis) Gagnon & G.P. Lewis, Brazil (*Lewis et al. 2019*) **M***Pomariajamesii* (Torr. & A. Gray) Walp., USA. Photo credits **A** AA Dreyer **B, I, K, L** GP Lewis **C** RH Fortunato **D, H, J** CE Hughes **E** P Baxter **F** Italo Specogna, Flora mendocina (http://www.floramendocina.com.ar/clase_3/zuccagnia_punctata_p9558.html) **G** M Thulin **M** P Alexander, SEINet Arizona Chapter (https://swbiodiversity.org/seinet/imagelib/imgdetails.php?imgid=253949).

**Figure 37.**
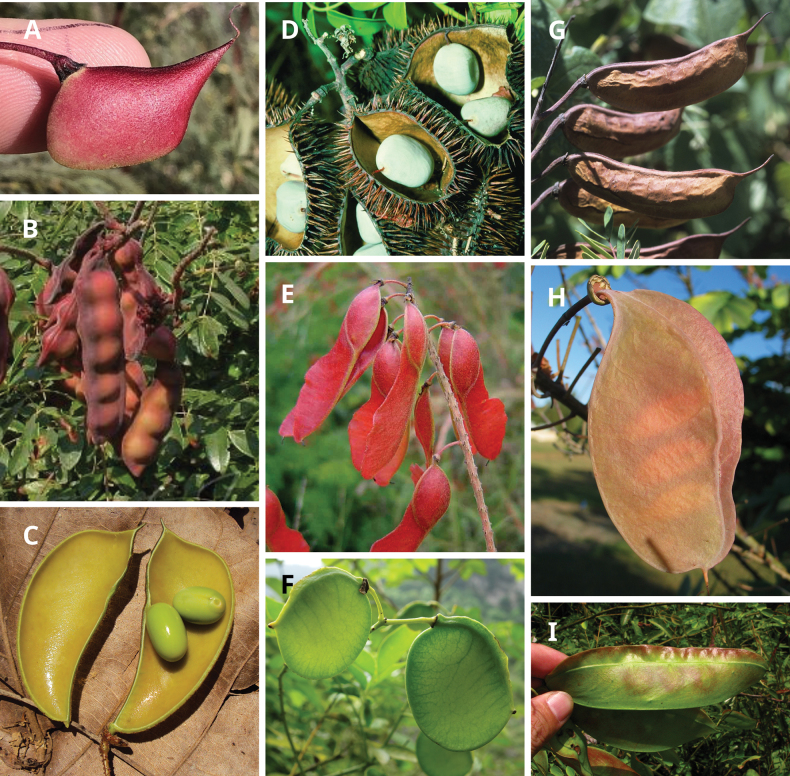
Fruits of the Caesalpinieae**A***Gelrebiarubra* (Engl.) Gagnon & G.P. Lewis, Namibia **B***Moullavaspicata* (Dalzell ex Wight) Nicolson, India, Maharashtra **C***Hultholiamimosoides* (Lam.) Gagnon & G.P. Lewis, India **D***Guilandinabonduc* L., Madagascar **E***Pterolobiumstellatum* (Forssk.) Brenan, Zimbabwe **F***Ticantosinensis* (Hemsley) R. Clark & Gagnon, China (*Clark 415*) **G***Biancaeadecapetala* (Roth) O. Deg., Peru, Ancash (*Hughes et al. 2227*) **H***Mezoneuronkauaiense* (H. Mann) Hillebr. **I***Mezoneuronandamanicum* Prain, Thailand (*Clark 251*). Photo credits **A** Dave U, iNaturalist (https://www.inaturalist.org/photos/41085094) **B** P Awale, Flowers of India (http://www.flowersofindia.net/) **C** VR Vinayaraj, India Biodiversity Portal (https://indiabiodiversity.org/observation/show/335158), the basionym of *Hultholiamimosoides***D** GP Lewis **E** BT Wursten, Flora of Zimbabwe (https://www.zimbabweflora.co.zw/speciesdata/image-display.php?species_id=127190&image_id=1) **F, I** P Suksathan **G** CE Hughes **H** D Eickhoff, https://www.flickr.com/photos/dweickhoff/4822012867/in/photostream/.

**Figure 38.**
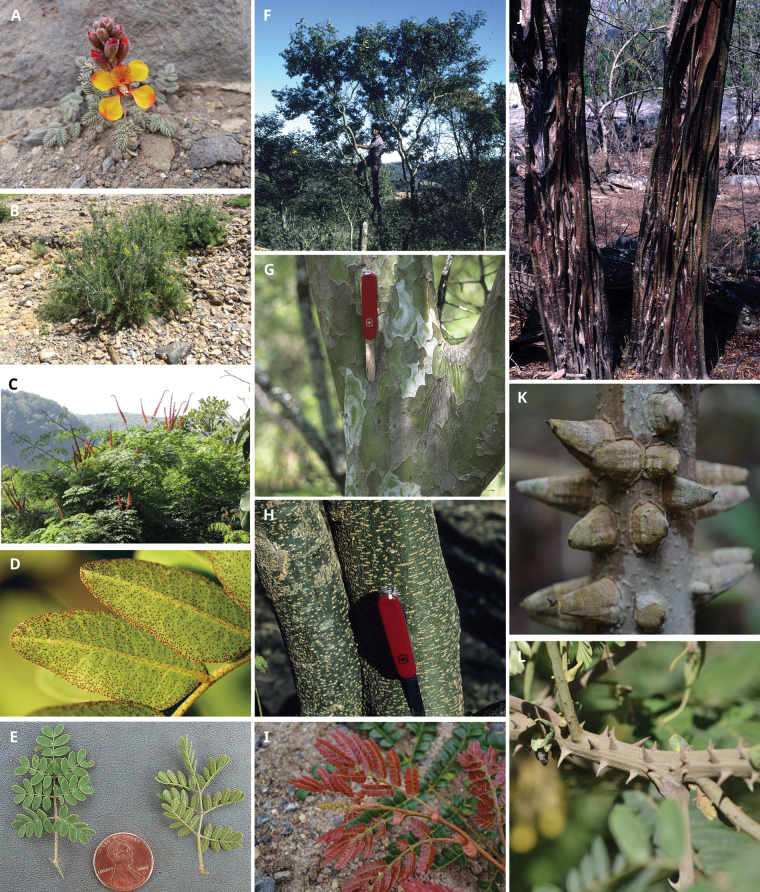
Vegetative traits of the Caesalpinieae**A***Hoffmannseggiaminor* (Phil.) Ulibarri, Bolivia **B***Hererolandiapearsonii* (L. Bolus) Gagnon & G.P. Lewis, Namibia, Sesriem Canyon **C***Moullavaspicata* (Dalzell ex Wight) Nicolson, India, Maharastra **D***Cordeauxiaedulis* Hemsl., undersurface of leaflets showing glands, Somalia **E***Pomariaaustrotexana* B.B. Simpson, USA, Texas **F** Small tree of *Erythrostemonnicaraguensis* (G.P. Lewis) Gagnon & G.P. Lewis, Nicaragua, Esteli (*Hawkins et al. 4*) **G** Leopard bark of *Libidibiaparaguariensis* (D. Parodi) G.P. Lewis, Bolivia, Santa Cruz (*Hughes 2475*) **H** Lenticelled bark of *Erythrostemonnicaraguensis* (G.P. Lewis) Gagnon & G.P. Lewis, Nicaragua, Esteli (*Hawkins et al. 4*) **I** Young flush of leaves of Cenostigmapluviosumvar.intermedium (G.P. Lewis) Gagnon & G.P. Lewis, Brazil, Bahia (*Lima et al. 7901*) **J** Fluted trunk of *Haematoxylumbrasiletto* H. Karst., Mexico, Oaxaca, (*Hughes 1947*) **K** Prickles on woody protuberances on a young trunk of *Paubrasiliaechinata* (Lam.) Gagnon, H.C. Lima & G.P. Lewis, Brazil, Bahia (*Lima et al. 7909*) **L** Recurved spines of *Biancaeadecapetala* (Roth) O. Deg., Peru, Ancash, (*Hughes et al. 3055*). Photo credits **A** GP Lewis **B** AA Dreyer **C** Shivaprakash, iNaturalist (https://inaturalist.ca/observations/81864757) **D** M Thulin **E** WR Carr **F–H, J** CE Hughes **I, K, L** E Gagnon.

Although there are no unique diagnostic morphological synapomorphies for the Caesalpinieae, it can be recognised by a combination of features, including the presence of glandular trichomes, prickles or spines, bilaterally symmetrical flowers with a somewhat modified lower sepal, and free stamens crowded around the pistil, although none of these characters are ubiquitous within the tribe. Flowers vary greatly (Fig. 35) and can be strongly modified depending on pollination system, and fruits across the tribe are extremely diverse (Figs 36, 37) reflecting a striking variation in seed dispersal strategies. Some leaf, armature and fruit characteristics can be used to distinguish genera and delimit the major clades of the tribe (Fig. 38). The phylogenomic analyses of Ringelberg et al. (2022) support two major clades in Caesalpinieae, also largely resolved in Gagnon et al. (2016), one primarily composed of species with bipinnate leaves [clade I of Gagnon et al. (2016) containing *Caesalpinia* s.s.] and the other grouping species with a terminal pinna [clade II of Gagnon et al. (2016) containing *Cenostigma* and sister genera] (Fig. 34). The two clades above also have differing defence strategies. With the exception of the unarmed *Coulteria*, the bipinnate leaf clade is characterised by the presence of spines and prickles along the branches, as well as having idioblasts. Although unresolved in Gagnon et al. (2016), the spinescent *Lophocarpinia*, *Haematoxylum* and *Hererolandia*, are now also shown to be resolved as sister to this clade in Ringelberg et al. (2022). Likewise, the other clade is characterised by the lack of thorns, and the presence of multicellular glandular structures on the stems, leaves and/or inflorescences (very rarely found elsewhere in the Caesalpinieae, such as in the genera *Coulteria*, *Tara* and *Hultholia*), and is here shown to also include the unarmed *Stuhlmannia* and *Cordeauxia*, two taxa that were unresolved in Gagnon et al. (2016). The nearly mutually exclusive distribution of external glands vs. spines+idioblasts gives some support to the idea that these structures constitute alternative plant defense strategies against herbivory (Lersten and Curtis 1994, 1996), even though the role and function of idioblasts and secretory glands in the Caesalpinieae have yet to be studied in detail.

At the generic level, fruits are highly variable and taxonomically more useful than flowers. Several of the genera can be differentiated based on fruit characteristics. For example, the fruits of *Balsamocarpon*, *Cordeauxia*, *Coulteria*, *Cenostigma*, *Guilandina*, *Haematoxylum*, *Hererolandia*, *Hultholia*, *Libidibia*, *Lophocarpinia*, *Moullava*, *Paubrasilia*, *Pterolobium* and *Zuccagnia* are all distinctive and provide useful diagnostic synapomorphies for these genera (Figs 36, 37). In contrast, only a few floral synapomorphies are diagnostic at the generic level: for example, *Guilandina* species have sepals that are valvate in bud; unisexual flowers are only present in the genera *Coulteria* and *Guilandina*; in *Balsamocarpon*, *Zuccagnia*, and *Hoffmannseggia*, sepals are persistent until fruiting (Fig. 36); and in *Pomaria* species, the androecium and gynoecium are cupped in the lower cucullate sepal (Fig. 35E). In general, however, floral morphology within subclades of the Caesalpinieae is highly variable reflecting differences in pollination syndromes, including examples of melittophily, chiropterophily, psychophily, phalaenophily and ornithophily, sometimes occurring among closely related congeneric species (e.g., in *Caesalpinia* s.s. and *Erythrostemon* – see Fig. 35). These repeated floral morphologies across disparate members of the Caesalpinieae suggest convergent evolution of similar pollination modes in multiple genera across the tribe.

The pollen details of individual genera are not easily extracted from the literature because so many generic names have been reinstated from within *Caesalpinia* s.l., or new genera described, since the palynological study of the Caesalpinioideae by Graham and Barker (1981). In addition, the surface ornamentation of the pollen varies according to pollinator type and structural similarities occur in the pollen of species in distinct genera within the tribe. In consequence, pollen type is not recorded in the generic descriptions of this treatment.

For an exhaustive list of accepted species names and synonyms in tribe Caesalpinieae, together with their currently accepted equivalents see LPWG (2022).

### ﻿ Stuhlmannia

Taxon classificationPlantaeFabalesFabaceae

Taub. in H.G.A. Engler, Pflanzenw. Ost.-Afr., C: 201. 1895.

Fig. 39

#### Type.

*Stuhlmanniamoavii* Taub.

#### Description.

Unarmed trees. **Stipules** minute conical projections, caducous. **Leaves** pinnate or bipinnate and then ending in a pair of pinnae, pinnae in (1) 2–10 opposite pairs, with reddish glands; leaflets in 3–12 opposite to subopposite pairs per pinna, eglandular or with red glands on the lower surface. **Inflorescence** a terminal or axillary raceme. **Flowers** bisexual, sub-actinomorphic; hypanthium persisting as a shallow cup at the pedicel apex as the fruit matures; sepals 5, caducous, valvate in bud, lowermost sepal not conspicuously differentiated in bud; petals 5, free, yellow, the median petal with red markings, slightly smaller than the other 4; stamens 10, filaments pubescent; ovary stipitate, with red sessile glands, glabrous to pubescent. **Fruit** a flattened, oblong, woody, elliptic legume, dehiscing along both sutures, valves twisting. **Seeds** flattened, sub-circular to ovate, brown.

#### Chromosome number.

Unknown.

#### Included species and geographic distribution.

**Figure 39.**
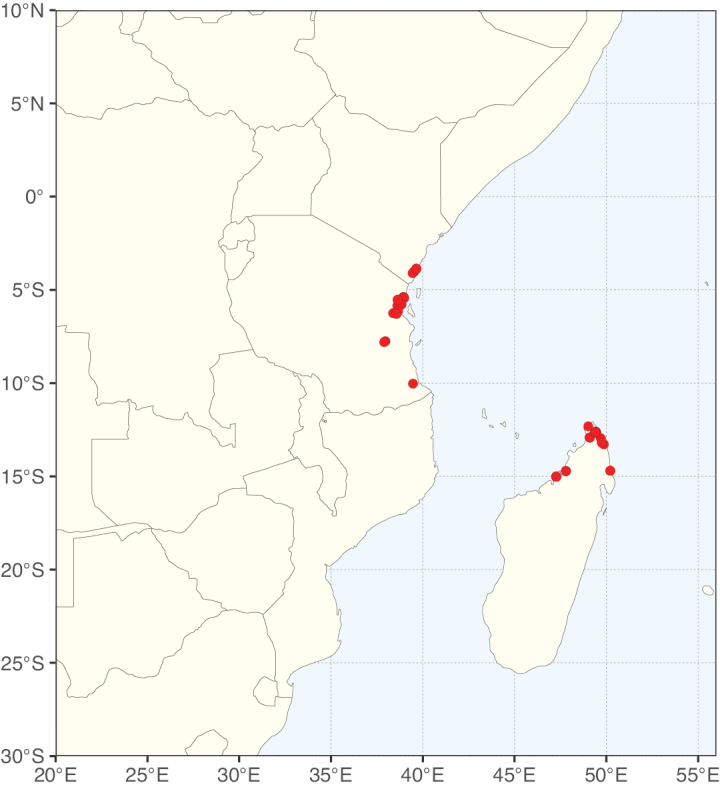
Distribution of *Stuhlmannia* based on quality-controlled digitised herbarium records. See Suppl. material 1 for the source of occurrence data.

Monospecific (*S.moavii*), in East Africa (Kenya and Tanzania) and northern Madagascar (Fig. 39).

#### Ecology.

Seasonally dry tropical forest, woodland on limestone and in riverine forest.

#### Etymology.

Named by Taubert for the German naturalist Franz Ludwig Stuhlmann (1863–1928).

#### Human uses.

Unknown.

#### Notes.

The genus is phylogenetically and morphologically closely related to *Cordeauxia* (Fig. 34), and species in both genera are known to have reddish secretory multicellular glands on the leaflets. Previous authors have recognised bipinnate and pinnate species as distinct taxa: *Caesalpiniainsolita* (Harms) Brenan & J.B. Gillett (≡*Hoffmannseggiainsolita* Harms) and *Caesalpiniadalei* Brenan & J.B. Gillett, respectively, but Lewis (1996), in support of his synonymising of the two species, commented that specimens have been collected with both pinnate and bipinnate leaves present on the same branch of an individual tree.

#### Taxonomic references.

Brenan (1967); Capuron (1967, under *C.insolita*); Du Puy and Rabevohitra (2002, under *C.insolita*); Gagnon et al. (2016); Lemmens (2010); Lewis (1996, 2005b).

### ﻿ Cordeauxia

Taxon classificationPlantaeFabalesFabaceae

Hemsl., Bull. Misc. Inform. Kew 1907: 361. 1907.

Figs 36 , 38 , 40

#### Type.

*Cordeauxiaedulis* Hemsl.

#### Description.

Multi-stemmed, unarmed, evergreen shrubs, red gland dots on stems. **Stipules** caducous or lacking (not seen). **Leaves** pinnate; leaflets in (1) 2–4 (6) pairs, coriaceous, with conspicuous red glands on the lower surface (Fig. 38D). **Inflorescence** a terminal, few-flowered raceme. **Flowers** bisexual, sub-actinomorphic; hypanthium persisting as a shallow cup at the pedicel apex as the fruit matures; sepals 5, caducous, with red glandular dots; petals 5, free, yellow; stamens 10, free, filaments pubescent; ovary with red gland dots. **Fruit** compressed-ovoid, ligneous, dehiscent, with very hard, thick valves, and a cornute beak, 1–4-seeded (Fig. 36G). **Seeds** ovoid.

#### Chromosome number.

2*n* = 24 (Miège and Miège 1978).

#### Included species and geographic distribution.

**Figure 40.**
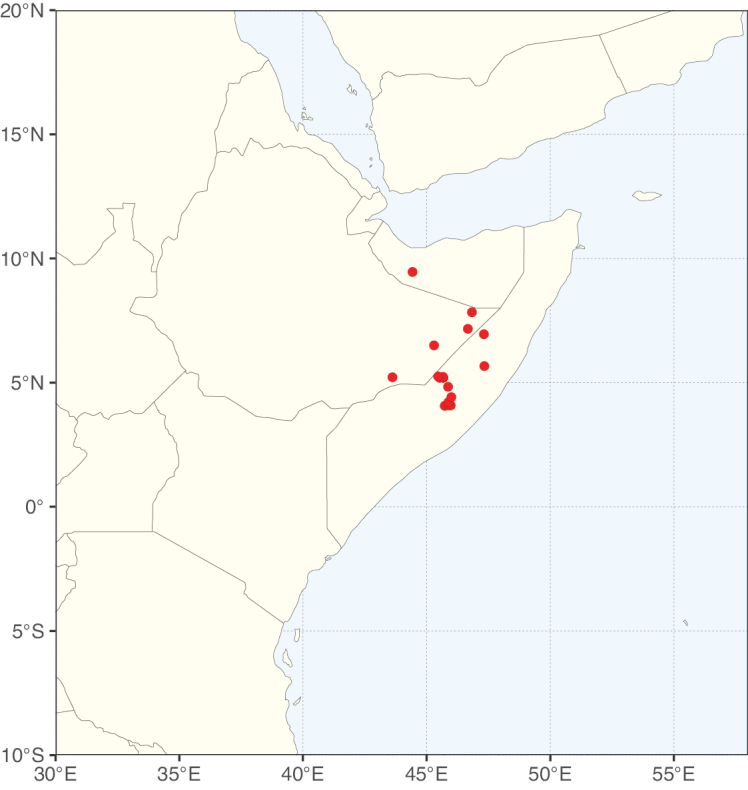
Distribution of *Cordeauxia* based on quality-controlled digitised herbarium records. See Suppl. material 1 for the source of occurrence data.

Monospecific (*C.edulis*), in north-eastern Africa (Somalia and Ethiopia). Introduced in Israel, Kenya, Sudan, Tanzania, and Yemen (Orwa et al. 2009; Fig. 40).

#### Ecology.

Seasonally dry tropical (semi-desert) bushland and thicket on sand.

#### Etymology.

Named by Hemsley for Captain H. E. S. Cordeaux (1870–1943), one time H. M. Commissioner in Somalia.

#### Human uses.

The seeds of *C.edulis* (yeheb nut) are used as human food and have potential as an arid-land food; also used as livestock fodder, production of a red dye, as medicine, wood, an insecticide, and a soap substitute (Lewis 2005b).

#### Notes.

*Cordeauxia* is closely related to the genus *Stuhlmannia* but is easily distinguished by its distinct habit: a shrub with a large tap-root (vs. medium-sized tree), and large, cornute, inertly dehiscent fruit with ovoid seeds (vs. non-cornute, explosively dehiscent fruit with compressed seeds).

#### Taxonomic references.

Brink (2006); Gagnon et al. (2016); Lewis (2005b); Roti-Michelozzi (1957); Thulin (1983, 1993).

### ﻿ Cenostigma

Taxon classificationPlantaeFabalesFabaceae

Tul., Ann. Sci. Nat., Bot., sér. 2. 20: 140. 1843.

**Figure 41.**
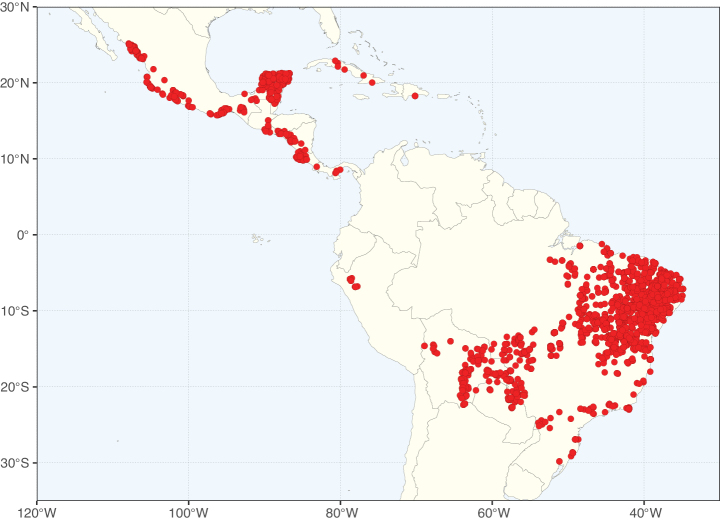
Distribution of *Cenostigma* based on quality-controlled digitised herbarium records. See Suppl. material 1 for the source of occurrence data.

Figs 35 , 36 , 38 , 41

#### Type.

*Cenostigmamacrophyllum* Tul.

#### Description.

Unarmed multi-stemmed shrubs, small compact trees, or large trees to 35 m, the larger trees with fluted trunks at maturity. **Stipules** filiform, spathulate-cucullate or lanceolate, caducous or sub-persistent, unknown for some species. **Leaves** pinnate or bipinnate, sometimes with stellate hairs or various types of sessile or stalked glands; species with pinnate leaves either with three leaflets or 2–9 pairs of opposite leaflets; species with bipinnate leaves with 1–11 pairs of opposite to alternate pinnae, plus a terminal pinna, each pinna with 3–29 alternate to subopposite (occasionally opposite), eglandular leaflets, or with black subepidermal glands on the undersurface, and/or with conspicuous, sessile or punctate glands on the undersurface or along the margins, in addition to stipitate glands. **Inflorescence** an axillary or terminal raceme, sometimes pyramidal in shape, sometimes aggregated into large showy panicles, pedicels articulated. **Flowers** bisexual, zygomorphic; hypanthium persisting as a small cup or wide shallow cup, or abscising as a ring around the pedicel apex or fruit stipe as the fruit matures; sepals 5, caducous, the lower cucullate sepal generally slightly longer than the other four; petals 5, free, bright yellow, the median petal with red or orange markings on the inner surface of the blade, the outer surface of the petal claw with short-stalked glands; stamens 10, free, filaments pubescent on lower portion, usually with short-stipitate glands along entire length; ovary pubescent with glands intermixed. **Fruits** laterally compressed, coriaceous to woody legumes with conspicuously thickened margins, dehiscent, sometimes explosively so, 2–6 (8)-seeded. **Seeds** ochre, brown, or mottled, shiny.

#### Chromosome number.

2*n* = 24 [*C.microphyllum* (Mart ex G. Don) Gagnon & G.P. Lewis, *C.pluviosum* (DC.) Gagnon & G.P. Lewis, *C.pyramidale* (Tul.) Gagnon & G.P. Lewis], 2*n* = 48 [*C.bracteosum* (Tul.) Gagnon & G.P. Lewis] (Alves and Custódio 1989; Beltrão and Guerra 1990; Rodrigues et al. 2014).

#### Included species and geographic distribution.

Twenty-three taxa in 15 species confined to the Neotropics. The genus extends around the Amazonian arc of dry forests and adjacent cerrado vegetation, as well as throughout Central America, and extending to the Caribbean, with endemics in Cuba and Hispaniola (Fig. 41).

#### Ecology.

Seasonally dry tropical forest, bushland, and thicket (restinga, caatinga, semi-arid thorn scrub), wooded grassland (cerrado and cerradão) and terra firme forest.

#### Etymology.

From *ceno*- (Greek = empty) and *stigma*, presumably alluding to the chambered stigma (a character of many species of the Caesalpinieae, and not restricted to *Cenostigma*).

#### Human uses.

*Cenostigmapluviosum* is often planted as an ornamental street tree in South America. Other species are used for their timber and production of charcoal, as well as for local medicine (Queiroz 2009).

#### Notes.

Based on phylogenetic and morphological evidence, Gagnon and Lewis in Gagnon et al. (2016) emended the description of *Cenostigma* and added several species from the disbanded genus *Poincianella*. Subsequently, C.pyramidalevar.diversifolium has been raised to the rank of species as *C.diversifolium* (Benth.) Gaem (Gaem 2021), thus increasing the number of recognised species in the genus to 15.

#### Taxonomic references.

Freire (1994); Gaem (2021); Gagnon et al. (2016); Lewis (1987, 1998, 2005b); Lewis et al. (2010); Queiroz (2009, under both *Cenostigma* and *Poincianella*); Ulibarri (1996); Warwick and Lewis (2009).

### ﻿ Libidibia

Taxon classificationPlantaeFabalesFabaceae

(DC.) Schltdl., Linnaea 5: 192. 1830.

Figs 36 , 38 , 42

Caesalpinia sect. Libidibia DC., Prodr. [A.P. de Candolle] 2: 483. 1825. Type: Caesalpiniacoriaria (Jacq.) Willd. [≡ Poincianacoriaria Jacq. (≡ Libidibiacoriaria (Jacq.) Schltdl.)]
Stahlia Bello, Anal. Soc. Esp. Hist. Nat. 10: 255. 1881. Type: Stahliamaritima Bello [= Libidibiamonosperma (Tul.) Gagnon & G.P. Lewis]

#### Type.

*Libidibiacoriaria* (Jacq.) Schltdl. [≡ *Poincianacoriaria* Jacq.]

#### Description.

Small to medium-sized or large unarmed trees; bark hard, smooth, with a patchwork of shades of grey, white, and pale green, often referred to as snake bark [except in *L.coriaria* and *L.monosperma* (Tul.) Gagnon & G.P. Lewis, where it is rough and fissured]. **Stipules** caducous or lacking (not seen). **Leaves** bipinnate, rarely pinnate (*L.monosperma*); bipinnate leaves with 2–10 pairs of opposite pinnae plus a single terminal pinna and 3–28(30) pairs of opposite leaflets per pinna; pinnate leaves with 4–6 pairs of opposite to subopposite leaflets; leaflets eglandular or with subsessile gland dots on the undersurface of the blades, on either side of the midvein. **Inflorescence** a terminal or axillary raceme or panicle, sometimes corymbose. **Flowers** bisexual, zygomorphic; hypanthium usually not persistent as the fruit matures; sepals 5, caducous, the lower sepal slightly longer and cucullate in bud; petals 5, free, yellow, or white, the median petal sometimes flecked or blotched orange or red; stamens 10, free, pubescent on the lower half of the filaments, eglandular [except for *L.ferrea* (Mart. ex Tul.) L.P. Queiroz, which has stipitate glands]; ovary eglandular. **Fruit** coriaceous to woody, straight (contorted in *L.coriaria*), indehiscent, eglandular, glabrous, black (red and somewhat fleshy in *L.monosperma*). **Seeds** somewhat laterally compressed.

#### Chromosome number.

2*n* = 24 [*L.coriaria*, *L.ferrea*, *L.paraguariensis* (D. Parodi) G.P. Lewis, *L.punctata* (Willd.) Britton], and 2*n* = 48 (*L.ferrea*) (Fedorov 1969; Beltrão and Guerrera 1990; Cangiano and Bernadello 2005).

#### Included species and geographic distribution.

**Figure 42.**
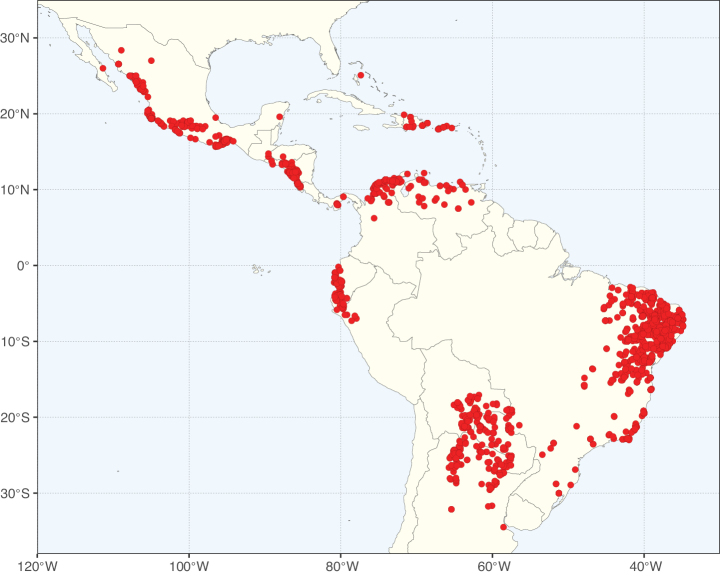
Distribution of *Libidibia* based on quality-controlled digitised herbarium records. See Suppl. material 1 for the source of occurrence data.

Ten taxa in seven species in the Neotropics. One species (*L.monosperma*, previously in the monospecific genus *Stahlia*) is endemic to Puerto Rico and the Dominican Republic. The other species are found across a circum–Amazonian arc of dry forests and adjacent cerrado vegetation, across the Andes, as well as throughout Central America (Fig. 42).

#### Ecology.

Seasonally dry tropical forests and thorn scrub (including Brazilian Caatinga) and savanna woodlands. *Libidibiamonosperma* occurs along the margins of mangrove swamps and in marshy deltas, in drier edaphic conditions.

#### Etymology.

The name *Libidibia* is derived from the vernacular name ‘libi-dibi’ or ‘divi-divi’ used for some species.

#### Human uses.

*Libidibia* species are widely used as ornamental park and street trees. Their fruits are rich in tannin and used commercially in the tanning industry and sometimes used for animal fodder, ink and local medicines. The wood and timber are prized in turnery and for parts of guitars and violins, as well as for decorative inlay and cabinet work. Some species are used in heavy construction (railway sleepers, beams, bridge supports), for tool handles and as firewood (Lewis 2005b).

#### Notes.

The genus needs revising; other species are perhaps waiting to be discovered and described, both in the field and in herbaria (Gagnon et al. 2016).

#### Taxonomic references.

Barreto Valdés (2013); Borges et al. (2012); Britton (1927); Britton and Rose (1930); Burkart (1936, as *Caesalpiniamelanocarpa* Griseb.); Gagnon et al. (2016); Lewis (2005b); Little and Wadsworth (1964); Macbride (1943); Queiroz (2009); Ulibarri (1996); U.S. Fish and Wildlife Service (1995).

### ﻿ Balsamocarpon

Taxon classificationPlantaeFabalesFabaceae

Clos in C. Gay, Fl. Chile. 2(2): 226. 1846 (publ. 1847).

Figs 36 , 43

#### Type.

*Balsamocarponbrevifolium* Clos

#### Description.

Shrub to 2 m tall, with 3–5 mm long, deflexed or patent, woody, nodal, often paired, sometimes caducous, woody spines. **Stipules** deltoid, glandular, caducous. **Leaves** pinnate, in fascicles on short brachyblasts; leaflets in 3–4 pairs, glabrous, fleshy. **Inflorescence** a short raceme. **Flowers** bisexual, sub-zygomorphic; a short hypanthium persisting (sometimes with the sepals still attached) and tightly adhering to the base of the fruit as it matures; sepals 5, fimbriate; petals 5, free, yellow, with glandular trichomes on the dorsal surface; stamens 10, free, filaments pubescent; ovary glandular and finely pubescent. **Fruit** thick, turgid, resinous, glandular, and indehiscent, 3–4-seeded. **Seeds** round, orange-brown.

#### Chromosome number.

Unknown.

#### Included species and geographic distribution.

**Figure 43.**
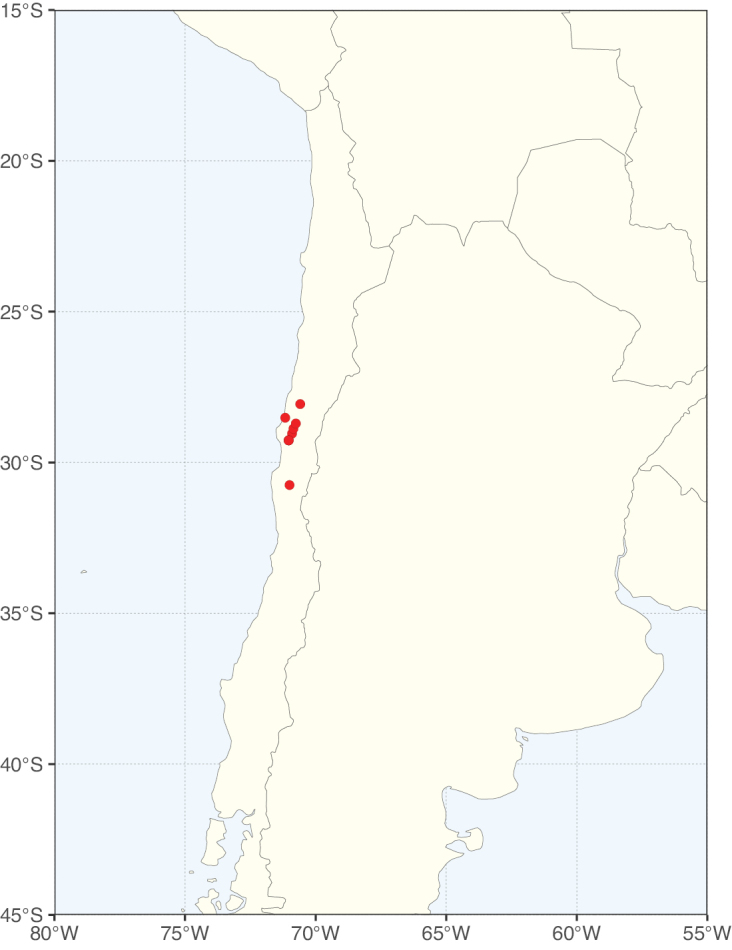
Distribution of *Balsamocarpon* based on quality-controlled digitised herbarium records. See Suppl. material 1 for the source of occurrence data.

Monospecific (*B.brevifolium*), endemic to northern Chile, from the Coquimbo and La Serena valleys (Fig. 43).

#### Ecology.

Desert scrub, rocky hillsides.

#### Etymology.

From *balsamo*- (Greek = balsam) and *carpos* (Greek = fruit), the fruits yield a sticky resin traditionally used for tanning.

#### Human uses.

Fruit resin used in the tanning industry, and wood locally used for charcoal production and as firewood (Estévez et al. 2010).

#### Notes.

Over-exploitation and increased fragmentation of the remaining populations of *B.brevifolium* mean that the species is vulnerable to extinction (Arancio and Marticorena 2008).

#### Taxonomic references.

Burkart (1940); Gagnon et al. (2016); Lewis (2005b); Nores et al. (2012); Ulibarri (1996, 2008).

### ﻿ Zuccagnia

Taxon classificationPlantaeFabalesFabaceae

Cav., Icon. 5: 2. 1799 nom. cons.

Figs 36 , 44

#### Type.

*Zuccagniapunctata* Cav.

#### Description.

Shrubs. **Stipules** caducous (not seen). **Leaves** pinnate; leaflets 5–13 pairs, subopposite, with glandular dots on both surfaces of the leaflet blades. **Inflorescence** a terminal, erect raceme. **Flowers** bisexual, zygomorphic; the calyx (hypanthium and sepals) persistent at fruit maturity; sepals 5, glabrous; the lower sepal cucullate and covering the other four in bud; petals 5, free, yellow, glandular trichomes on the dorsal surface of the petal blades; stamens 10, free, pubescent; ovary pilose. **Fruit** ovoid-acute, oblique, laterally compressed, indehiscent, 1-seeded, gall-like, on a short stipe and covered with long reddish-brown bristles at maturity. **Seeds** laterally compressed.

#### Chromosome number.

2*n* = 24 (Fedorov 1969).

#### Included species and geographic distribution.

**Figure 44.**
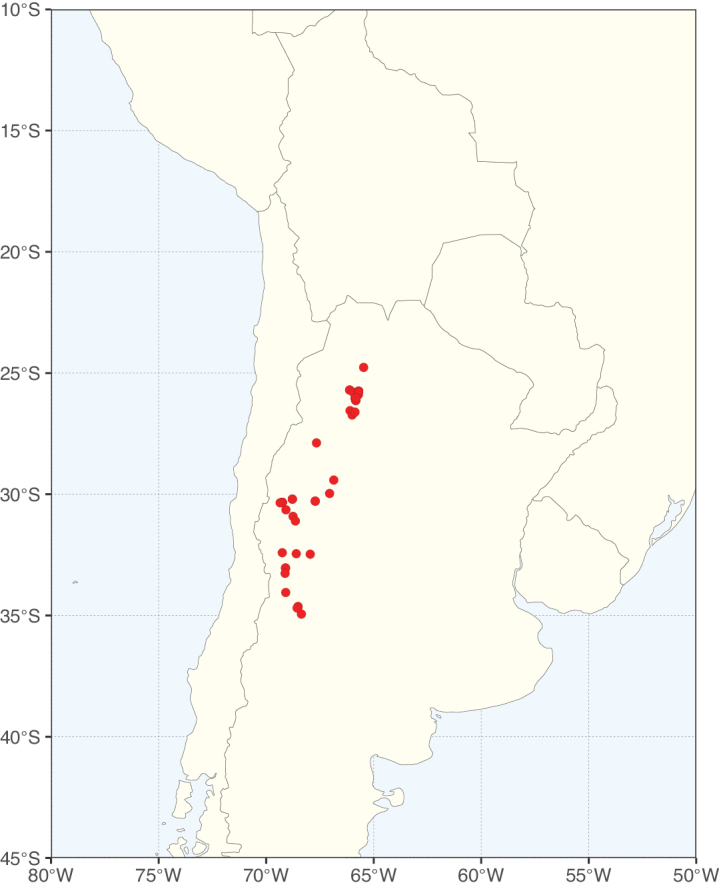
Distribution of *Zuccagnia* based on quality-controlled digitised herbarium records. See Suppl. material 1 for the source of occurrence data.

Monospecific (*Z.punctata*), restricted to north-western and central-western Argentina (Fig. 44).

#### Ecology.

Dry temperate upland and montane bushlands and thickets on sandy plains.

#### Etymology.

Named by Cavanilles for the Italian physician, traveller and plant collector, Attilio Zuccagni (1754–1807).

#### Human uses.

Minor local medicinal uses; the leaves yield a yellow dye (Lewis 2005b).

#### Notes.

Although recorded and described from Chile in the 19^th^ Century, the genus has been cited as doubtful for the flora of Chile (Marticorena and Quezada 1985; Ulibarri 2005).

#### Taxonomic references.

Burkart (1952); Gagnon et al. (2016); Kiesling et al. (1994); Lewis (2005b); Nores et al. (2012); Ulibarri (2005, 2008).

### ﻿ Hoffmannseggia

Taxon classificationPlantaeFabalesFabaceae

Cav., Icon. 4: 63. 1798 nom. cons.

Figs 36 , 38 , 45

Larrea Ortega, Nov. Rar. Pl. Descr. Dec.: 15. t. 2. 1797, nom. rej., non Larrea Cav., Anales Hist. Nat. 2(4): 119. 1800 [Zygophyllaceae]. Type: Larreaglauca Ortega [≡ Hoffmannseggiaglauca (Ortega) Eifert]
Moparia Britton & Rose, N. Amer. Fl. 23(5): 317. 1930. Type: Mopariarepens (Eastw.) Britton & Rose [≡ Caesalpiniarepens Eastw. (≡ Hoffmannseggiarepens (Eastw.) Cockerell)]

#### Type.

*Hoffmannseggiafalcaria* Cav., nom. superfl. [≡ *Hoffmannseggiaglauca* (Ortega) Eifert (≡ *Larreaglauca* Ortega)]

#### Description.

Perennial woody herbs, most species forming a basal rosette, or subshrubs, unarmed, often arising from bud-bearing and tuberous roots, shoots pubescent and with gland-tipped trichomes. **Stipules** lanceolate, ovate or deltate, acuminate, caducous or persistent. **Leaves** bipinnate, ending in a pair of pinnae plus a single terminal pinna (except for *Hoffmannseggiaaphylla* (Phil.) G.P. Lewis & Sotuyo); pinnae in 1–13 opposite pairs; leaflets small and numerous, in 2–15 (18) opposite pairs per pinna. **Inflorescence** a terminal or axillary raceme. **Flowers** bisexual, zygomorphic; calyx (hypanthium and sepals) usually persistent as the fruit matures; sepals 5, weakly imbricate; petals 5, free, yellow to orange, the median petal often with red markings; stamens 10, free, filaments pubescent; ovary glabrous to pubescent, eglandular to glandular. **Fruit** laterally compressed, straight or sometimes falcate, the sutures almost parallel, papery to leathery, glabrous to pubescent, eglandular or with glandular trichomes, indehiscent or dehiscent, with twisting valves. **Seeds** compressed, ovoid.

#### Chromosome number.

2*n* = 24 [*H.drepanocarpa* A. Gray, *H.eremophila* (Phil.) Burkart ex Ulibarri, *H.glauca* (Ortega) Eifert, *H.microphylla* Torr., *H.oxycarpa* Benth., *H.viscosa* (Ruiz & Pav.) Hook. & Arn.] (Fedorov 1969; Zhao 1996; Zanin and Cangiano 2001).

#### Included species and geographic distribution.

**Figure 45.**
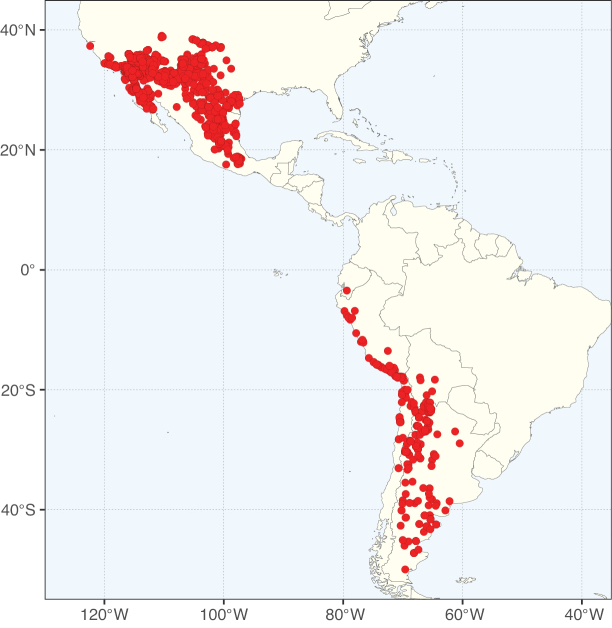
Distribution of *Hoffmannseggia* based on quality-controlled digitised herbarium records. See Suppl. material 1 for the source of occurrence data.

Twenty-five taxa in 23 species, with an amphitropical distribution in the Americas: 10 species restricted to North America (southern USA and Mexico), 12 in South America (mainly Andean), and one species (*H.glauca*) widespread throughout the range of the genus (Fig. 45).

#### Ecology.

Subtropical desert and semi-desert grassland, often in open areas and on disturbed sites, on sandy, rocky, or calcareous soils.

#### Etymology.

Named by Antonio José Cavanilles for the German botanist, entomologist and ornithologist, Johann Centurius Graf von Hoffmannsegg (1766–1849).

#### Human uses.

*Hoffmannseggiaglauca* produces tubers once eaten by indigenous groups in North America (but sometimes becomes a noxious weed); the roots of *H.intricata* Brandegee produce a reddish-brown dye (Lewis 2005b).

#### Notes.

A complete synopsis and key to species [except *H.aphylla* which was transferred to the genus by Lewis and Sotuyo (2010)] is available in Simpson and Ulibarri (2006).

#### Taxonomic references.

Britton and Rose (1930, under *Larrea* and *Moparia*); Burkart (1936); Gagnon et al. (2016); Lewis (1998, under *Caesalpiniapumilio* Griseb., 2005b); Lewis and Sotuyo (2010); Macbride (1943, under *Caesalpinia*); Simpson (1999); Simpson and Miao (1997); Simpson and Ulibarri (2006); Simpson et al. (2004, 2005); Ulibarri (1979, 1996).

### ﻿ Stenodrepanum

Taxon classificationPlantaeFabalesFabaceae

Harms, Notizbl. Bot. Gart. Berlin-Dahlem 7: 500. 1921.

Fig. 46

#### Type.

*Stenodrepanumbergii* Harms

#### Description.

Suffrutescent shrub, or perennial herb, (10) 20–40 cm, with bud-bearing and occasionally tuber-forming roots; glabrous, with globose sessile glands scattered along the branches. **Stipules** ovate. **Leaves** bipinnate, pinnae in 1–3 opposite pairs plus a single terminal pinna; leaflets in 5–9 opposite to subopposite pairs per pinna, embedded glands on the lower surface. **Inflorescence** a lax, terminal raceme. **Flowers** bisexual, zygomorphic; hypanthium persistent as a small cup at the apex of the pedicel as the fruit matures; sepals 5, caducous, glandular, the lower cucullate sepal covering the other four in bud; petals 5, free, yellow, the median petal with red markings, stipitate glands on the dorsal surface; stamens 10, free, filaments pubescent and glandular; ovary glandular. **Fruit** linear to slightly falcate, cylindrical, torulose, 1–5-seeded. **Seeds** ovoid.

#### Chromosome number.

2*n* = 24, 36 (Caponio et al. 2012).

#### Included species and geographic distribution.

**Figure 46.**
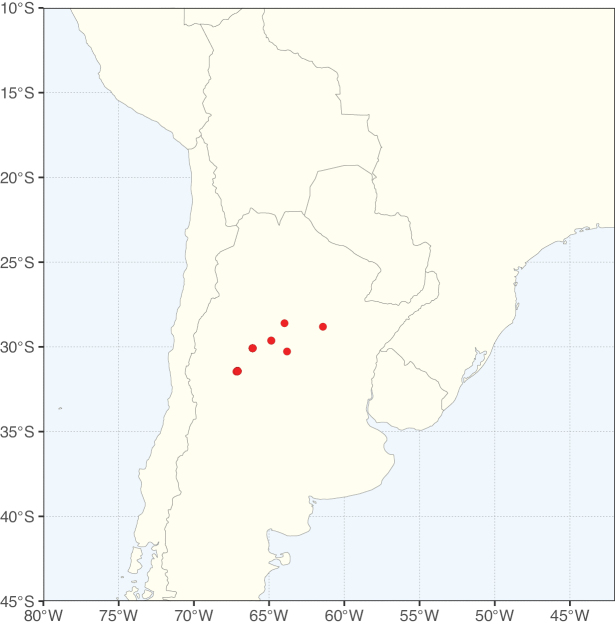
Distribution of *Stenodrepanum* based on quality-controlled digitised herbarium records. See Suppl. material 1 for the source of occurrence data.

Monospecific (*S.bergii*), endemic to central and western Argentina (Fig. 46).

#### Ecology.

Subtropical wooded grassland and scrub, especially close to salt pans.

#### Etymology.

From Greek, *steno*- (= narrow) and *drepano*- (= sickle), in allusion to the narrow sickle-shaped fruit.

#### Human uses.

Unknown.

#### Notes.

Morphologically similar in appearance to the genus *Hoffmannseggia* but with a distinctive linear to slightly falcate, cylindrical, torulose fruit. Resolved as sister to *Hoffmannseggia* in Gagnon et al. (2016), but not included in the analysis of Ringelberg et al. (2022).

#### Taxonomic references.

Caponio et al. (2012); Gagnon et al. (2016); Kiesling et al. (1994); Lewis (2005b); Nores et al. (2012); Ulibarri (1978, 1979, 2008).

### ﻿ Erythrostemon

Taxon classificationPlantaeFabalesFabaceae

Klotzsch in Link, Klotzsch & Otto, Icon. Pl. Rar. Horti. Berol. 2: 97, t. 39. 1844.

**Figure 47.**
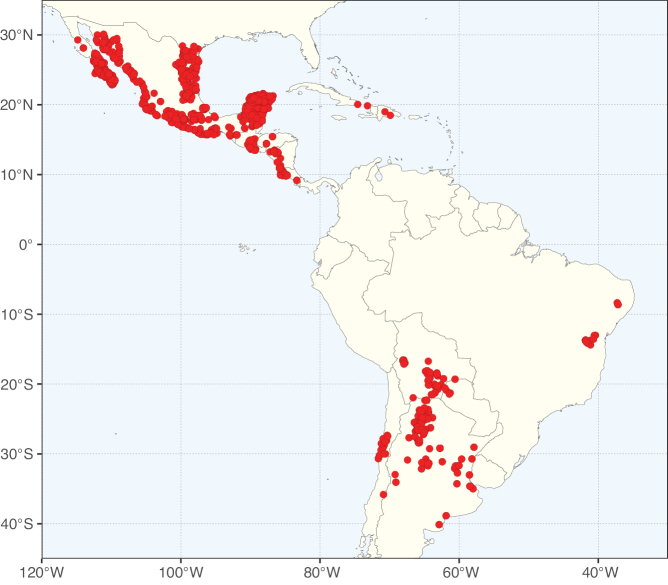
Distribution of *Erythrostemon* based on quality-controlled digitised herbarium records. See Suppl. material 1 for the source of occurrence data.

Figs 35 , 36 , 38 , 47

Poincianella Britton & Rose, N. Amer. Fl. 23(5): 327. 1930. Type: Poincianellamexicana (A. Gray) Britton & Rose [≡ Caesalpiniamexicana A. Gray (≡ Erythrostemonmexicanus (Rose) Gagnon & G.P. Lewis)]
Schrammia Britton & Rose, N. Amer. Fl. 23(5): 317. 1930. Type: Schrammiacaudata (A. Gray) Britton & Rose [≡ Hoffmannseggiacaudata A. Gray (≡ Erythrostemoncaudatus (A. Gray) Gagnon & G.P. Lewis)]

#### Type.

*Erythrostemongilliesii* (Hook.) Klotzsch [≡ *Poincianagilliesii* Hook.]

#### Description.

Shrubs or small to medium-sized trees, occasionally suffrutices, unarmed (except *E.glandulosus* (Bertero ex DC.) Gagnon & G.P. Lewis). **Stipules** ovate-lanceolate, ovate, or orbicular, acute to acuminate, sometimes foliaceous, cordate and auriculate at the base, caducous or less often persistent. **Leaves** bipinnate, usually ending in a single terminal pinna; pinnae in 1–6 (15), opposite pairs; leaflets in 2–13 (20) opposite pairs per pinna, leaflet blades eglandular or with conspicuous black sessile glands along the margin, these sometimes sunken in the sinuses of the crenulated margin. **Inflorescence** an axillary or terminal raceme. **Flowers** bisexual, zygomorphic; hypanthium persistent as a wide or narrow, shallow or deep cup, or sometimes abscising to form a free ring around the pedicel apex as the fruit matures; sepals 5, lower sepal cucullate in bud, all sepals caducous; petals 5, free, bright golden yellow to creamish yellow, salmon pink or pink-scarlet, the median petal often with red-orange markings, the corolla diverse in form; stamens 10, free, filaments pubescent, eglandular or with stipitate glands; ovary pubescent, eglandular or with sessile or stipitate glands. **Fruit** a chartaceous to coriaceous or slightly woody, laterally compressed legume, elastically dehiscent with twisting valves, eglandular or with stipitate glands, (1) 2–7 (8)-seeded. **Seeds** yellow to ochre-brown or mottled with grey and black.

#### Chromosome number.

2*n* = 24 [*E.exostemma* (Moc. & Sessé ex DC.) Gagnon & G.P. Lewis, *E.gilliesii*, *E.hughesii* (G.P. Lewis) Gagnon & G.P. Lewis, *E.melanadenius* (Rose) Gagnon & G.P. Lewis, *E.mexicanus* (Rose) Gagnon & G.P. Lewis, *E.nelsonii* (Britton & Rose) Gagnon & G.P. Lewis, *E.yucatanensis* (Greenm.) Gagnon & G.P. Lewis] (Fedorov 1969; Cangiano and Bernardello 2005; Mata-Sucre et al. 2020).

#### Included species and geographic distribution.

Thirty-four taxa in 31 species: 22 species are found across the southern USA, Mexico, and Central America, one occurs in the Caribbean (Cuba and Hispaniola), and eight occur in South America (Fig. 47).

#### Ecology.

Seasonally dry tropical forests across the Neotropics; also occurring in deserts, yungas-puna transition zones, and chaco-transition forests (Argentina, Bolivia, Chile, Paraguay).

#### Etymology.

From Greek, *erythro*- (= red) and *stemon* (= stamen), the type species *E.gilliesii* has long red exserted stamens, but this is unusual in the genus as circumscribed here.

#### Human uses.

*Erythrostemongilliesii* is widely cultivated as a garden ornamental and is hardy in Mediterranean and temperate regions (Lewis 2005b).

#### Notes.

Originally described as a monospecific genus, its circumscription was recently emended to include many species previously placed in Central American and Mexican *Poincianella* (Gagnon et al. 2016).

#### Taxonomic references.

Britton and Rose (1930); Burkart (1936); Gagnon et al. (2016); Lewis (1998, 2005b); Queiroz (2009); Ulibarri (1996).

### ﻿ Pomaria

Taxon classificationPlantaeFabalesFabaceae

Cav., Icon. 5: 1. 1799.

**Figure 48.**
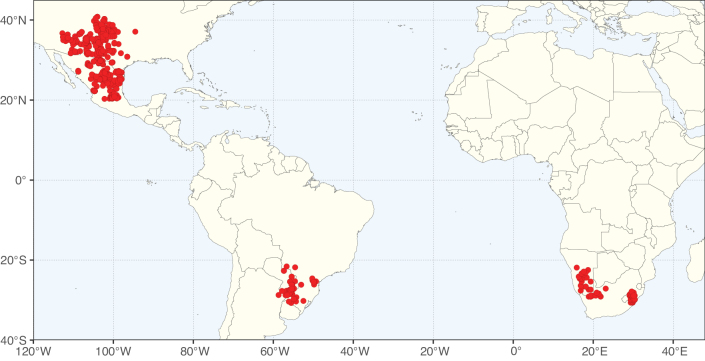
Distribution of *Pomaria* based on quality-controlled digitised herbarium records. See Suppl. material 1 for the source of occurrence data.

Figs 35 , 36 , 38 , 48

Melanosticta DC., Prodr. [A.P. de Candolle] 2: 485. 1825. Type: Melanostictaburchellii DC. [≡ Pomariaburchellii (DC.) B.B. Simpson & G.P. Lewis]
Cladotrichium Vogel, Linnea 11: 401. 1837. Lectotype (designated by Simpson and Lewis 2003): Cladotrichiumrubicundum Vogel [≡ Pomariarubicunda (Vogel) B.B. Simpson & G.P. Lewis]

#### Type.

*Pomariaglandulosa* Cav.

#### Description.

Small shrubs, subshrubs, or perennial herbs, with a moderate to dense indumentum of simple curled hairs, sometimes also scattered plumose trichomes, intermixed with sessile, oblate glands (drying black) on stems. **Stipules** mostly laciniate, glandular, persistent. **Leaves** bipinnate, pinnae in 1–8 (11) opposite pairs, plus a terminal pinna; leaflets small, 2–16 (27) opposite pairs per pinna, always with multiple sessile glands on their lower surface (these orange in the field, drying black). **Inflorescence** a terminal or axillary raceme. **Flowers** bisexual, zygomorphic; hypanthium persistent as a small shallow cup as the fruit matures; sepals 5, caducous, lanceolate, the lower sepal cucullate, covering the other 4 in bud, and closely embracing the androecium and gynoecium at anthesis; petals 5, free, yellow, white, red, or pink; stamens 10, filaments pubescent; ovary sparsely to densely hairy and glandular. **Fruit** a linear or sickle-shaped, laterally compressed legume, with a sparse to dense covering of plumose/dendritic or stellate trichomes (sometimes obscure and restricted to fruit margin) intermixed with sessile oblate glands (drying black), elastically dehiscent, with twisting valves. **Seeds** laterally compressed.

#### Chromosome number.

2*n* = 24 *P.rubicunda* (Vogel) B.B. Simpson & G.P. Lewis, *P.stipularis* (Vogel) B.B. Simpson & G.P. Lewis) (Fedorov 1969; Biondo et al. 2005a).

#### Included species and geographic distribution.

Seventeen taxa in 16 species: nine in North America, four in South America, and three in southern Africa (Fig. 48).

#### Ecology.

Mainly in subtropical dry grassland and in degraded sites, many on limestone.

#### Etymology.

Named by Cavanilles for Dominic Pomar, botanist from Valencia, and doctor to Philip III (1598–1621), King of Spain.

#### Human uses.

Unknown.

#### Notes.

Revisions of the species of *Pomaria* are available for North America (Simpson 1998), South America and Africa (Simpson and Lewis 2003), and southern Africa (under the name *Hoffmannseggia*, Brummitt and Ross 1974).

#### Taxonomic references.

Brummitt and Ross (1974, as *Hoffmannseggia*); Burkart (1936); Gagnon et al. (2016); Lewis (2005b); Simpson (1998); Simpson and Lewis (2003); Simpson et al. (2006); Ulibarri (1996, 2008).

### ﻿ Arquita

Taxon classificationPlantaeFabalesFabaceae

Gagnon, G.P. Lewis & C.E. Hughes, Taxon 64(3): 479. 2015.

Fig. 49

#### Type.

*Arquitamimosifolia* (Griseb.) Gagnon, G.P. Lewis & C.E. Hughes [≡ *Caesalpiniamimosifolia* Griseb.]

#### Description.

Small to medium-sized, often decumbent, shrubs, usually with glandular trichomes on various parts of the plant. **Stipules** ovate-obovate to deltoid, usually with a fimbriate-glandular margin, caducous. **Leaves** bipinnate; pinnae 1–5 pairs, usually with a single terminal pinna; leaflets in 4–12 opposite pairs per pinna, often with maroon/black glands in depressions on crenulated leaflet margins, and sometimes with occasional sessile black glands on the undersurface of leaflet blades. **Inflorescence** a leaf-opposed raceme. **Flowers** bisexual, zygomorphic; hypanthium persistent as a small shallow cup at the pedicel apex as the fruit matures; sepals 5, caducous, the lower sepal cucullate; petals 5, free, yellow to orange, median petal sometimes streaked red; stamens 10, free; ovary usually covered with gland-tipped trichomes. **Fruits** laterally compressed, lunate-falcate legumes, covered sparsely to densely with gland-tipped trichomes, these sometimes dendritic. **Seeds** laterally compressed, ovate-orbicular, the testa shiny olive-grey, sometimes mottled or streaked black.

#### Chromosome number.

2*n* = 24 (*A.mimosifolia*) (Cangiano and Bernardello 2005).

#### Included species and geographic distribution.

**Figure 49.**
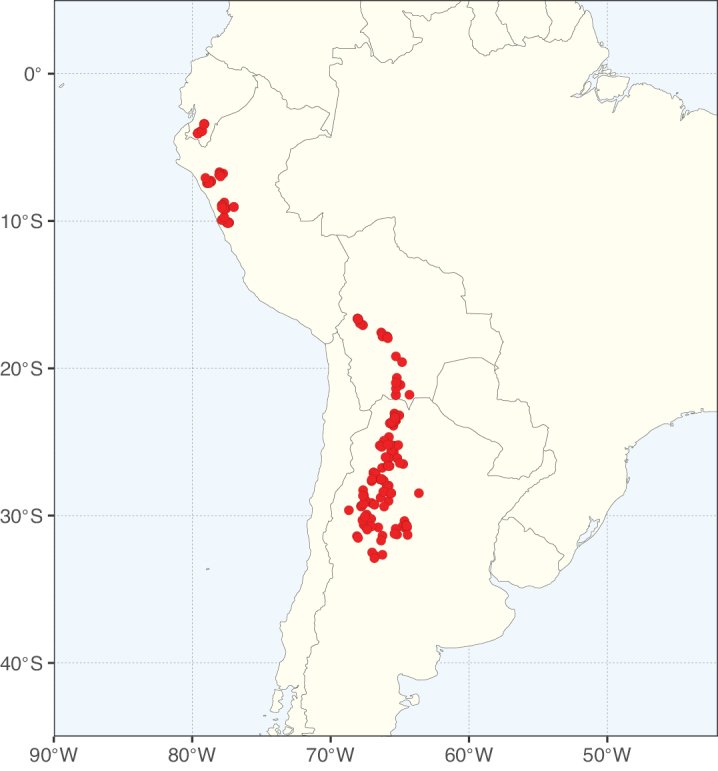
Distribution of *Arquita* based on quality-controlled digitised herbarium records. See Suppl. material 1 for the source of occurrence data.

Six taxa in five species restricted to the Andes in South America, in disjunct inter-Andean valleys, in Ecuador, Peru, Bolivia and Argentina (Fig. 49).

#### Ecology.

Tropical and subtropical seasonally dry, montane, and rupestral habitats.

#### Etymology.

*Arquita* is the vernacular name for *A.trichocarpa* (Griseb.) Gagnon, G.P. Lewis & C.E. Hughes in Argentina (Ulibarri 1996).

#### Human uses.

Unknown.

#### Notes.

A revision of *Arquita* with a key to species is available in Gagnon et al. (2015).

#### Taxonomic references.

Burkart (1936); Gagnon et al. (2015, 2016); Lewis (1998); Lewis et al. (2010); Ulibarri (1996).

### ﻿ Hererolandia

Taxon classificationPlantaeFabalesFabaceae

Gagnon & G.P. Lewis, PhytoKeys 71: 29. 2016.

Fig. 36 , 38 , 50

#### Type.

*Hererolandiapearsonii* (L. Bolus) Gagnon & G.P. Lewis [≡ *Caesalpiniapearsonii* L. Bolus]

#### Description.

Multi-stemmed shrubs armed with curved, deflexed prickles. **Stipules** not seen. **Leaves** pinnate, borne in fascicles on short woody brachyblasts that are usually subtended by a pair of tiny (sometimes obscure) prickles; leaflets (4) 5–7 (9) pairs, opposite, eglandular. **Inflorescence** a short raceme. **Flowers** zygomorphic, bisexual; hypanthium short, persistent as a ring around the stipe of the fruit; sepals 5, free, the lower sepal cucullate and covering the other 4 sepals in bud, all sepals caducous; petals 5, yellow, free; stamens 10, free, pubescent on the lower half; ovary pubescent. **Fruit** a thinly woody, laterally compressed, almost circular to strongly sickle-shaped legume, dehiscing along the sutures, finely pubescent and covered in robust trichomes, usually 1-seeded. **Seeds** laterally compressed.

#### Chromosome number.

Unknown.

#### Included species and geographic distribution.

**Figure 50.**
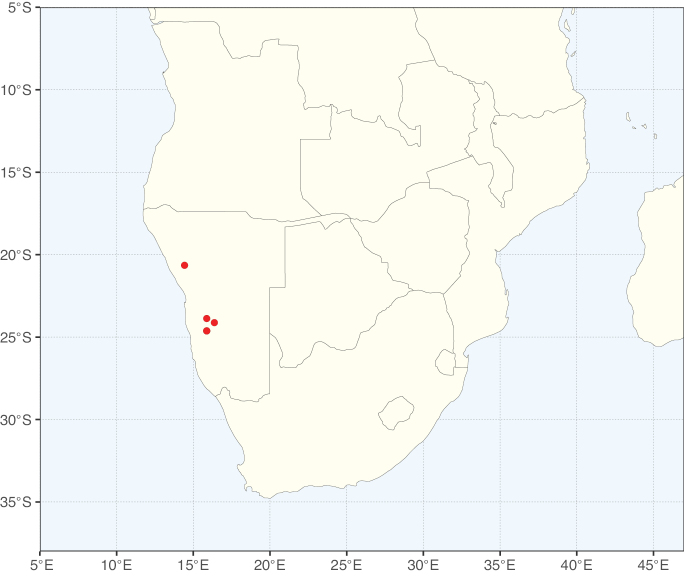
Distribution of *Hererolandia* based on quality-controlled digitised herbarium records. See Suppl. material 1 for the source of occurrence data.

Monospecific (*H.pearsonii*), endemic to Namibia, on the Great Escarpment (Fig. 50).

#### Ecology.

Semi-desert and desert areas, on stony, sandy soils.

#### Etymology.

The type locality of *H.pearsonii* is in the semiarid Hereroland, a region of eastern Namibia inhabited by the Herero people, who are nomadic cattle herders.

#### Human uses.

Unknown.

#### Notes.

The genus was described by Gagnon et al. (2016), based on its isolated and unresolved position in the Caesalpinia group phylogeny, and its distinctive sickle-shaped to circular, 1-seeded legume covered in robust trichomes. In Ringelberg et al. (2022) the genus is resolved as sister to a clade comprising *Haematoxylum* and *Lophocarpinia*.

#### Taxonomic references.

Bolus (1920); Curtis and Mannheimer (2005); Gagnon et al. (2016); Nkowki and Swelankomo (2003).

### ﻿ Haematoxylum

Taxon classificationPlantaeFabalesFabaceae

L., Sp. Pl. 1: 384. 1753.

Figs 36 , 38 , 51

Haematoxylon L., Philosophia Botanica: 34. 1764, orth. var.
Cymbosepalum Baker, Bull. Misc. Inform. Kew 1895 (100–101): 103. 1895. Type: Cymbosepalumbaronii Baker [= Haematoxylumcampechianum L.]

#### Type.

*Haematoxylumcampechianum* L.

#### Description.

Multi-stemmed shrubs to medium-sized trees, armed with scattered straight conical spines, and short, lateral spinescent shoots; mature trees with conspicuously fluted trunks, shrubs often with ribbed branches. **Stipules** minute, acuminate, caducous. **Leaves** pinnate or bipinnate (both can be present on the same individual in some species), eglandular; pinnate leaves with 2–6 pairs of leaflets; bipinnate leaves with 1–3 pairs of pinnae plus a terminal pinna, each pinna with 2–5 (6) pairs of opposite leaflets. **Inflorescence** a terminal or axillary raceme or panicle. **Flowers** bisexual, actinomorphic to zygomorphic; the short hypanthium persisting in fruit as a small cup; sepals 5, free, the lower sepal cucullate and slightly covering the other 4 in bud, sepals caducous; petals 5, yellow to pale yellow or white, free; stamens 10, free, filaments pubescent, particularly on the lower half; ovary glabrous to pubescent. **Fruit** laterally flattened, membranous to chartaceous, dehiscing along the middle of the valves, or near the margin of the fruit, but never along the sutures, 1–3-seeded. **Seeds** oblong to reniform, flattened.

#### Chromosome number.

2*n* = 24 (*H.campechianum*) (Fedorov 1969).

#### Included species and geographic distribution.

**Figure 51.**
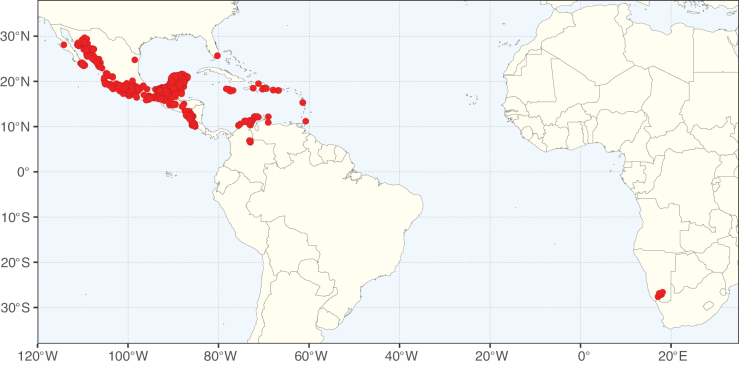
Distribution of *Haematoxylum* based on quality-controlled digitised herbarium records. See Suppl. material 1 for the source of occurrence data.

Five species: two in Central America (Salvador to Costa Rica), Mexico, South America (Colombia and Venezuela) and the Caribbean (perhaps introduced), two endemic to Mexico, and one in Southern Africa (Namibia; Fig. 51).

#### Ecology.

Deserts, seasonally dry tropical semi-deciduous scrub and thorn scrub, sandy riverbeds, and dry rocky hillsides. One species (*H.campechianum*) is known to grow in frequently inundated marshy areas by rivers.

#### Etymology.

From Greek, *haemato*- (= bloody) and *xylon* (= wood), alluding to the blood-red heartwood of *H.campechianum* which produces a brilliant red dye.

#### Human uses.

The heartwood of *H.campechianum* is the source of a colourless chemical, haematoxylin, which upon oxidation turns to haematein, a commercial dark violet dye used for wool, silk, cotton, fur, leather, bone and synthetic fibre dying, and with iron chromium mordants to obtain red and black; also used as a stain in microscopical preparations (particularly to show up cell nuclei), and ink for writing and painting and the rich red colour has been used to adulterate wine. Species are also used medicinally, as ornamentals and living hedges and the bee flowers yield a high-quality honey (Lewis 2005b).

#### Notes.

This genus is easily diagnosable by the ascending secondary veins of its leaflets, which form a sharp angle with the primary vein. There is a key to species by Durán and Sousa (2014).

#### Taxonomic references.

Barreto Valdés (2013); Curtis and Mannheimer (2005); Durán and Ramírez (2008); Durán and Sousa (2014); Gagnon et al. (2016); Lewis (2005b); Ross (1977); Roux (2003); Standley and Steyermark (1946).

### ﻿ Lophocarpinia

Taxon classificationPlantaeFabalesFabaceae

Burkart, Darwiniana 11: 256. 1957.

Figs 36 , 52

#### Type.

*Lophocarpiniaaculeatifolia* (Burkart) Burkart [≡ *Cenostigmaaculeatifolium* Burkart]

#### Description.

Shrubs, armed with scattered straight, conical, 2–5 mm long spines on shoots; leaves and inflorescences crowded on brachyblasts. **Stipules** acuminate, caducous. **Leaves** pinnate, leaflets in 2 (3) opposite pairs, eglandular, with a pair of small prickles at the insertions of the leaflets. **Inflorescence** a short, corymbiform, pubescent raceme, each with 3–6 flowers. **Flowers** zygomorphic, bisexual; hypanthium turbinate, fleshy, persistent at the apex of the pedicel as the fruit matures; sepals 5 caducous, lower sepal cucullate and covering the other 4 sepals in bud, embracing the androecium and gynoecium at anthesis; petals 5, yellow to yellow-orange, free, the median petal differentiated from the rest by a fleshy claw and wavy blade margins, pubescent; stamens 10, free, filaments pubescent; ovary glabrous. **Fruit** a lomentum, with 1–5 segments, falcate, with 4 coarsely serrate wings. **Seeds** ellipsoid to reniform, smooth.

#### Chromosome number.

Unknown.

#### Included species and geographic distribution.

**Figure 52.**
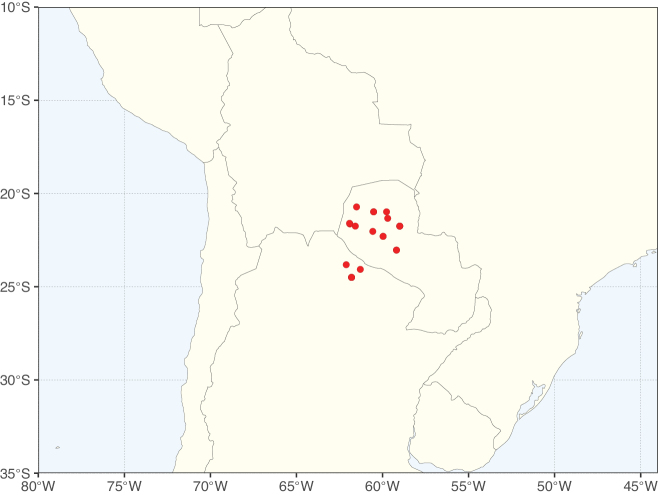
Distribution of *Lophocarpinia* based on quality-controlled digitised herbarium records. See Suppl. material 1 for the source of occurrence data.

Monospecific, restricted to Argentina and Paraguay (Fig. 52).

#### Ecology.

Chaco woodlands and seasonally dry tropical to subtropical forests.

#### Etymology.

From Greek, *lopho*- (= combed or crested) and *carpos* (= fruit), the fruit has 4 crested wings, the ending -inia signifies a close relationship with *Caesalpinia*.

#### Human uses.

Unknown.

#### Notes.

*Lophocarpinia* is closely related to the genus *Haematoxylum* but has a distinctive lomentaceous fruit with coarsely serrated wings.

#### Taxonomic references.

Burkart (1957); Gagnon et al. (2016); Lewis (2005b); Nores et al. (2012); Ulibarri (2008).

### ﻿ Caesalpinia

Taxon classificationPlantaeFabalesFabaceae

L., Sp. Pl. 1: 380. 1753.

Figs 35 , 53

Poinciana L., Sp. Pl. 1: 380. 1753. Type: Poincianapulcherrima L. [≡ Caesalpiniapulcherrima (L.) Sw.]
Caesalpinia sect. Brasilettia DC., Prodr. [A.P. de Candolle] 2: 481. 1825. Type: Caesalpiniabrasiliensis L.
Brasilettia (DC.) Kuntze, Revis. Gen. Pl. 1: 164. 1891. Type: Brasilettiabrasiliensis (L.) Kuntze [≡ Caesalpiniabrasiliensis L.]

#### Type.

*Caesalpiniabrasiliensis* L.

#### Description.

Shrubs or small trees, usually armed with curved deflexed prickles. **Stipules** minute, caducous or apparently lacking. **Leaves** bipinnate, pinnae (1) 2–6 pairs, opposite; leaflets 3–13 pairs per pinna, alternate to opposite. **Inflorescence** a terminal or axillary raceme or panicle. **Flowers** pedicellate, bisexual, zygomorphic; hypanthium persistent as a cup at the apex of the pedicel as the fruit matures, if the fruit stipitate then the stipe exerted from the hypanthial cup, or the whole calyx persistent (e.g., in *Caesalpiniapulcherrima* (L.) Sw.); sepals 5, caducous or persistent, eglandular, the lower sepal strongly cucullate and covering the other 4 sepals in bud; petals 5, variable in colour (yellow, white, red, orange or green), the corolla also variable in shape; stamens 10, free, the filaments pubescent; ovary glabrous and eglandular. **Fruit** a wingless, unarmed, coriaceous, glabrous, eglandular, explosively dehiscent legume, with twisting valves, 3–7-seeded. **Seeds** laterally compressed.

#### Chromosome number.

2*n* = 24 (*C.bahamensis* Lam., *C.pulcherrima*) (Darlington and Wylie 1956; Fedorov 1969).

#### Included species and geographic distribution.

**Figure 53.**
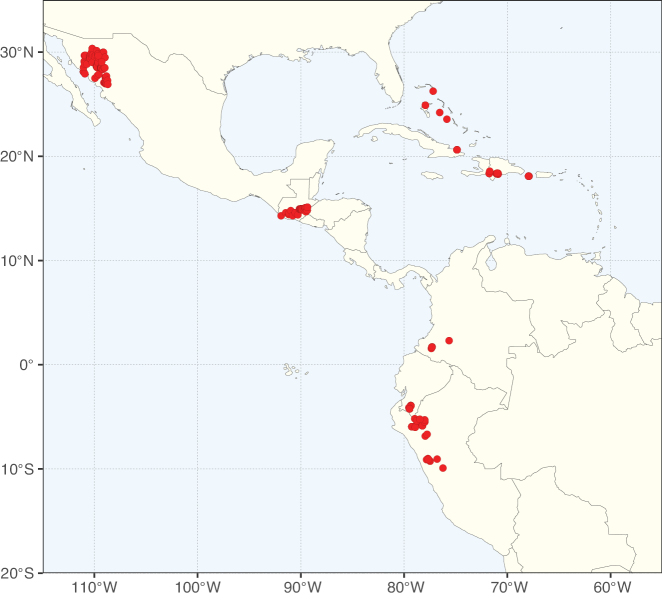
Distribution of *Caesalpinia* based on quality-controlled digitised herbarium records. The distribution of *Caesalpiniapulcherrima* in Sonora and Guatemala is based on specimen records that morphologically appear to be non-cultivated, but the exact native distribution of the species remains difficult to ascertain because it has been widely cultivated for a long time. See Suppl. material 1 for the source of occurrence data.

Nine species restricted to the Neotropics (apart from the pantropically cultivated *C.pulcherrima*). One species (*C.cassioides* Willd.) occurs in the northern Andes from Peru to Colombia, one (*C.pulcherrima*) is likely native in Guatemala and the state of Sonora in Mexico (but is widely cultivated), *C.nipensis* Urb. is endemic to Cuba, and all other species are also Caribbean in distribution (Fig. 53).

#### Ecology.

Seasonally dry tropical forests, coastal thicket, bushlands and thorn scrubs, dry plains, and riparian woodlands, on soils derived from limestone or sandstone.

#### Etymology.

Named by Linnaeus for Andrea Cesalpino (1519–1603), Italian naturalist, botanical collector, systematist and philosopher, physician to Pope Clement VIII, professor of medicine and botany in Pisa and Rome.

#### Human uses.

*Caesalpiniapulcherrima* is widely cultivated pantropically as a garden and park ornamental and has various medicinal properties (Lewis 2005b). The species includes red, orange, and yellow-flowered forms and cultivated specimens are usually unarmed and lack bristles (unlike wild specimens which are armed and bristly).

#### Notes.

*Caesalpinia*, as recently re-circumscribed (Gagnon et al. 2016), is reduced to nine species, although a detailed taxonomic revision is needed to properly delimit species and synonymy. Palaeotropical species previously included in *Caesalpinia* s.s., sensu Lewis (2005b), have been transferred to other genera, notably *Denisophytum* and *Gelrebia* (Gagnon et al. 2016). While *Brasilettia* (DC.) Kuntze is a synonym of *Caesalpinia*, the genus *Brasilettia* sensu Britton and Rose (1930) included eight species which are now all placed in the genus *Coulteria* Kunth. The variation in corolla colour and shape in *Caesalpinia* species is related to different pollination systems: bees, butterflies, birds, and bats.

#### Taxonomic references.

Barreto Valdés (2013); Britton and Rose (1930); Gagnon et al. (2016); Lewis (2005b); Macbride (1943); Ulibarri (1996).

### ﻿ Denisophytum

Taxon classificationPlantaeFabalesFabaceae

R. Vig., Notul. Syst. (Paris) 13(4): 349. 1948.

Fig. 54

#### Type.

*Denisophytummadagascariense* R. Vig.

#### Description.

Shrubs or small trees, armed with straight or curved, deflexed prickles, scattered along shoots and in pairs at the petiole base (except *D.madagascariense* which is unarmed). **Stipules** minute, or foliaceous and conspicuous, caducous or persistent. **Leaves** bipinnate, pinnae in 1–6 opposite pairs; leaflets 2–10 (11) opposite pairs per pinna. **Inflorescence** a terminal or axillary raceme. **Flowers** bisexual, zygomorphic; a short hypanthium persistent at the pedicel apex as the fruit matures; sepals 5, caducous, lower sepal cucullate and covering the other 4 sepals in bud; petals 5, free, yellow, the median petal sometimes with red markings on the inner face of the blade; stamens 10, free, filaments pubescent and eglandular; ovary glabrous. **Fruits** coriaceous, laterally compressed (inflated in *D.madagascariense*), glabrous, eglandular, stipitate legumes, elastically dehiscent, with twisting valves. **Seeds** laterally compressed.

#### Chromosome number.

2*n* = 24 [*D.pauciflorum* (Griseb.) Gagnon & G.P. Lewis] (Darlington and Wylie 1956).

#### Included species and geographic distribution.

**Figure 54.**
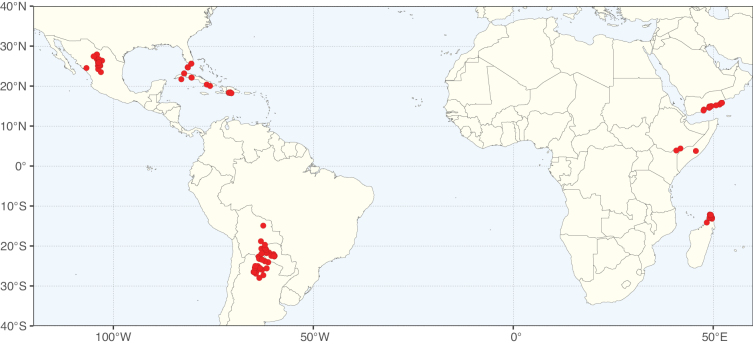
Distribution of *Denisophytum* based on quality-controlled digitised herbarium records. See Suppl. material 1 for the source of occurrence data.

Nine taxa in eight species. Three species are distributed in Mexico, Florida, and the Caribbean, one species is endemic to Paraguay, Bolivia, and Argentina, one is endemic to northern Madagascar, and the other three occur in northern Kenya, Somalia, and Arabia (Fig. 54).

#### Ecology.

Low deciduous seasonally dry tropical woodlands or scrublands, also in open pine woodlands, coastal plains and foothills. Species in Madagascar and Africa grow in limestone soils.

#### Etymology.

It has been hypothesised that *Denisophytum* honours Marcel Denis, a botanist with expertise in the genus *Euphorbia* L. in Madagascar, and a friend and collaborator of René Viguier, the genus author (Gagnon et al. 2016).

#### Human uses.

Unknown.

#### Notes.

An evaluation of species limits is needed for this genus. It has a highly disjunct trans-continental distribution typical of lineages occupying the succulent biome sensu Schrire et al. (2005b).

#### Taxonomic references.

Barreto Valdés (2013); Brenan (1967); Britton and Rose (1930); Burkart (1936); Capuron (1967); Du Puy and Rabevohitra (2002); Gagnon et al. (2016); Roti-Michelozzi (1957); Thulin (1983, 1993); Ulibarri (1996); Viguier (1949).

### ﻿ Paubrasilia

Taxon classificationPlantaeFabalesFabaceae

Gagnon, H.C. Lima & G.P. Lewis, PhytoKeys 71: 36. 2016.

Figs 36 , 38 , 55

#### Type.

*Paubrasiliaechinata* (Lam.) Gagnon, H.C. Lima & G.P. Lewis [≡ *Caesalpiniaechinata* Lam.]

#### Description.

Medium sized to large tree, armed with small to large, upturned prickles, these usually arising from woody protuberances, bark flaking in large woody plates; heartwood red, with the trunk exuding a red sap when injured. **Stipules** caducous (on seedlings lanceolate, acute to acuminate). **Leaves** bipinnate, ending with a pair of pinnae; pinnae in (2) 3–20 alternate pairs; leaflets alternate, (2) 3–19 (21) leaflets per pinna (generally the number of leaflets is inversely proportional to their size). **Inflorescence** a terminal, or occasionally axillary, raceme or panicle, with 15–40 flowers. **Flowers** bisexual, zygomorphic; hypanthium persistent as a shallow cup or abscising to form a small free ring around the pedicel apex as the fruit matures; sepals 5, caducous, the lowest sepal cucullate, covering the other 4 in bud; petals 5, free, bright yellow, eglandular, the median petal with a blood-red blotch on the inner face; stamens 10, free, eglandular, the filaments densely pubescent on lower half; ovary pubescent with bristles intermixed. **Fruit** a spiny, finely pubescent, sub-lunate, woody, elastically dehiscent legume with twisting valves, 1–2-seeded. **Seeds** laterally compressed.

#### Chromosome number.

2*n* = 24 (Beltrão and Guerra 1990)

#### Included species and geographic distribution.

**Figure 55.**
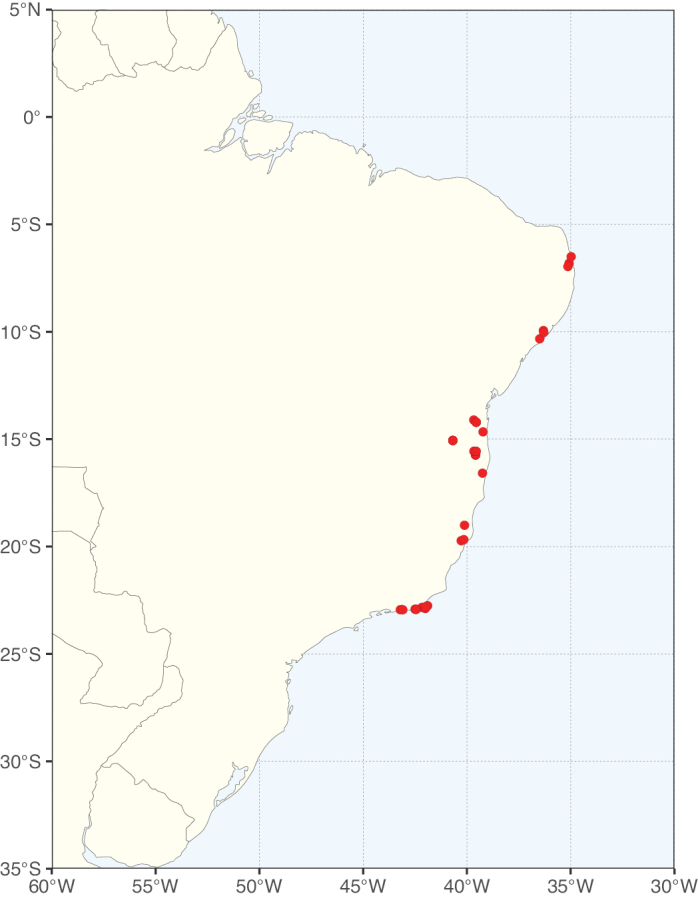
Distribution of *Paubrasilia* based on quality-controlled digitised herbarium records. See Suppl. material 1 for the source of occurrence data.

Monospecific (*P.echinata*), endemic to eastern Brazil, from the state of Rio Grande do Norte to Rio de Janeiro (Fig. 55).

#### Ecology.

Dry coastal cactus scrub often on rocky outcrops, inland in Mata Atlântica, and in tall restinga on well-drained sandy soil.

#### Etymology.

“Pau-brasil” is the national tree of Brazil and has long been associated with the country. The Latinization of its well-known and much used common name recognises the importance of the species to Brazil.

#### Human uses.

Widely cultivated in Brazil as an ornamental street or park tree, and sometimes in plantations. The tree’s red sap was once used for dying cotton and cloth and its wood is much prized for the manufacture of high-quality violin bows (Bueno 2002).

#### Notes.

Originally described as *Caesalpiniaechinata* by Lamarck (1785), this phylogenetically isolated taxon was placed in its own monospecific genus by Gagnon et al. (2016). A detailed account of this iconic species is available in Bueno (2002).

#### Taxonomic references.

Bueno (2002); Cardoso et al. (1998, 2005); Gagnon et al. (2016); Lewis (1998).

### ﻿ Coulteria

Taxon classificationPlantaeFabalesFabaceae

Kunth, Nov. Gen. Sp. 6: 328. 1824.

Figs 36 , 56

Guaymasia Britton & Rose, N. Amer. Fl. 23(5): 322. 1930. Type: Guaymasiapumila Britton & Rose [≡ Coulteriapumila (Britton & Rose) Sotuyo & G.P. Lewis]

#### Lectotype

**(designated by Gagnon et al. 2016).***Coulteriamollis* Kunth

#### Description.

Trees or shrubs, unarmed, dioecious. **Stipules** minute, caducous or lacking. **Leaves** bipinnate, pinnae in 2–6 opposite to subopposite pairs; leaflets in (2) 4–12 (14) opposite to alternate pairs per pinna, eglandular, glabrous to velvety pubescent. **Inflorescence** an axillary or terminal raceme. **Flowers** unisexual, zygomorphic; hypanthium either persisting as a shallow cup or not persistent as the fruit matures; sepals 5, caducous, with a lower glandular-pectinate, cucullate sepal, covering the other 4 in bud; petals 5, yellow, free; staminate flowers with 10 free stamens, filaments pubescent, eglandular; pistillate flower with ovary pubescent or glabrous. **Fruit** chartaceous to papyraceous, laterally-compressed, oblong to elliptic (occasionally suborbicular), indehiscent (or sometimes opening along one suture), wingless, often persisting to next flowering season, eglandular, glabrous to densely velutinous, 1–6-seeded. **Seeds** ovate, orbicular, or sub-quadrate, compressed.

#### Chromosome number.

2*n* = 24 (Mata-Sucre et al. 2020).

#### Included species and geographic distribution.

**Figure 56.**
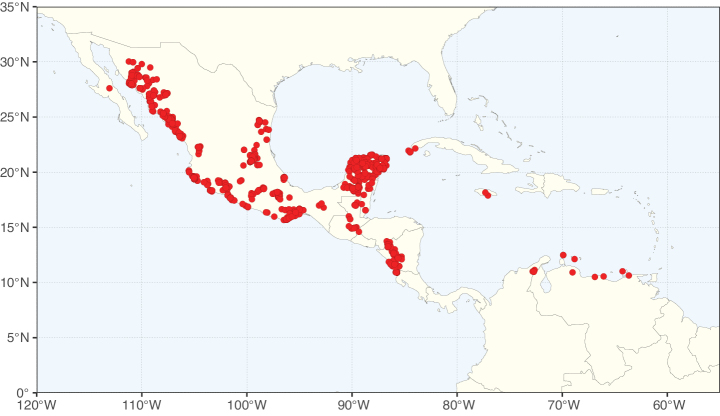
Distribution of *Coulteria* based on quality-controlled digitised herbarium records. See Suppl. material 1 for the source of occurrence data.

Eleven species in Mexico and Central America, one species extending to Belize, Aruba, Cuba, Jamaica and Curaçao, one to Venezuela (including Isla Margarita) and Colombia (Fig. 56).

#### Ecology.

Seasonally dry tropical forest, semi-deciduous and deciduous woodlands, xerophytic scrub forests and thorn scrub, on sandy, calcareous, or metamorphic substrates, some species endemic on limestone.

#### Etymology.

Named by Kunth for the Irish botanist Thomas Coulter (1793–1846) who collected in central Mexico (1825–1834) and was curator of the herbarium at Trinity College, Dublin, Ireland.

#### Human uses.

A preferred local firewood in its native range; some species are used as ornamentals in living fences (Lewis 2005b).

#### Notes.

Gagnon et al. (2016) presented a preliminary account of the genus *Coulteria*. This was followed by a synopsis of the genus by Sotuyo et al. (2017). Since 2017, four new species have been published: *C.rosalindamedinae* R. Torres, A. Saynes & P. Tenorio (Torres-Colín and Saynes-Vásquez 2020); *C.lewisii* (originally as “*lewisiae*”) Sotuyo & J.L. Contr. (Sotuyo and Contreras-Jiménez 2021a; Sotuyo et al. 2021), *C.delgadoana* Sotuyo & J.L. Contr. (Sotuyo and Contreras-Jiménez 2021b), and *C.sousae* Sotuyo, J.L. Contreras & L. Rico (Sotuyo et al. 2022). *Brasilettia* sensu Britton & Rose (1930), which included eight species, is a synonym of *Coulteria*. The genus has a distinctive seed chemistry (the seeds produce substituted phenylalanines derived from a shikimic acid metabolic pathway; Kite and Lewis 1994) and prismatic crystals in ray cells and a chambered axial parenchyma in its wood (Gasson et al. 2009).

#### Taxonomic references.

Britton and Rose (1930); Gagnon et al. (2016); Lewis (2005b); Sotuyo and Contreras-Jiménez (2021a, 2021b); Sotuyo et al. (2017, 2021, 2022); Torres-Colín and Saynes-Vásquez (2020); Ulibarri (1996); Zamora Villalobos (2010).

### ﻿ Tara

Taxon classificationPlantaeFabalesFabaceae

Molina, Saggio Chili: 283. 1789.

Figs 35 , 57

Nicarago Britton & Rose, N. Amer. Fl. 23(5): 319. 1930. Type: Nicaragovesicaria (L.) Britton & Rose [≡ Caesalpiniavesicaria L. (≡ Taravesicaria (L.) Molinari, Sánchez Och. & Mayta)]
Russellodendron Britton & Rose, N. Amer. Fl. 23(5): 320. 1930. Type: Russellodedroncacalaco (Bonpl.) Britton & Rose [≡ Caesalpiniacacalaco Bonpl. (≡ Taracacalaco (Bonpl.) Molinari & Sánchez Och.)]

#### Type.

*Taratinctoria* Molina [= *Taraspinosa* (Molina) Britton & Rose]

#### Description.

Shrubs or trees, armed with deflexed prickles. **Stipules** minute, deltate-lanceolate, caducous, or lacking. **Leaves** bipinnate, ending with a pair of pinnae, sometimes armed with prickles at the base of the pinnae and leaflets; pinnae in 2–5 opposite pairs; leaflets 1–8 opposite pairs per pinna. **Inflorescence** a terminal or axillary raceme or panicle. **Flowers** bisexual, zygomorphic; hypanthium persistent as a shallow cup at the pedicel apex or abscising as a narrow free ring as the fruit matures; sepals 5, eglandular, caducous, lower sepal cucullate covering the other 4 sepals in bud, with a pectinate, fimbriate or entire margin; petals 5, free, yellow, the median petal with red markings; stamens 10, free, the filaments pubescent, eglandular; ovary puberulent to glabrescent. **Fruit** an indehiscent legume, straight, oblong, laterally compressed, slightly turgid and somewhat fleshy, coriaceous. **Seeds** ellipsoid, brown, shiny.

#### Chromosome number.

2*n* = 24 (all three species) (Mata-Sucre et al. 2020).

#### Included species and geographic distribution.

**Figure 57.**
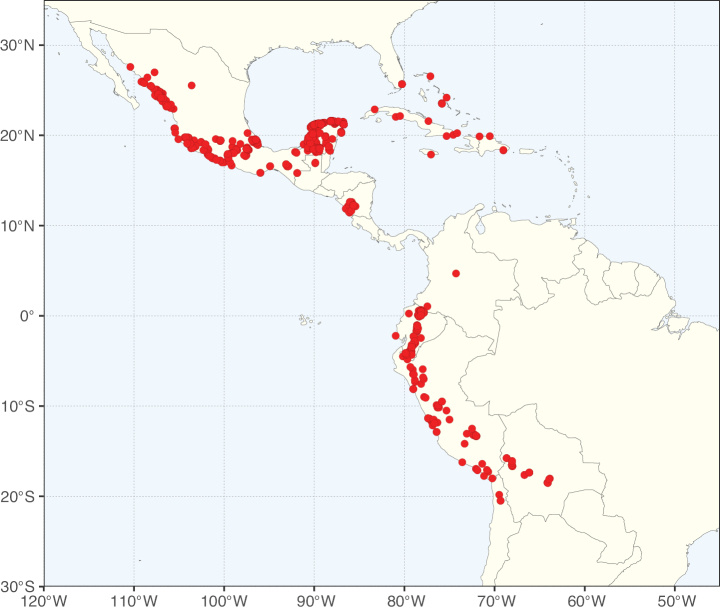
Distribution of *Tara* based on quality-controlled digitised herbarium records. See Suppl. material 1 for the source of occurrence data.

Three species, one in South America (*T.spinosa*, which occurs from central and south-western Colombia through Ecuador and Peru into Bolivia), one in Mexico [*T.cacalaco* (Bonpl.) Molinari & Sánchez Och.] and one in Mexico, Guatemala, Nicaragua and extending into the Caribbean [*T.vesicaria* (L.) Molinari, Sánchez Och. & Mayta] (Fig. 57).

#### Ecology.

Seasonally dry tropical forests to semi-arid thorn scrubs.

#### Etymology.

Derived from the vernacular name ‘tara’ in Peru, Bolivia, and Chile.

#### Human uses.

*Taraspinosa* is widely cultivated across the tropics and subtropics (including in the Canary Islands) as a source of tannins, gums and firewood, and occasionally as an ornamental (Lewis 2005b).

#### Notes.

As originally described by Molina, the genus *Tara* contained a single binomial. The genus *Coulteria*, as described by Kunth (1824), contained three species, one, *C.mollis*, designated as the type of *Coulteria*, the other two clearly shown to be more closely related to the type species of *Tara* (Gagnon et al. 2013, 2016). Based on Gagnon et al. (2013), Molinari-Novoa and Sánchez Ocharan (2016a) transferred *Caesalpiniacacalaco* and *Caesalpiniavesicaria* to the genus *Tara*.

#### Taxonomic references.

Barreto Valdés (2013); Britton and Rose (1930); Gagnon et al. (2016); Lewis (2005b); Macbride (1943, as *Caesalpiniaspinosa*); Molinari-Novoa and Sánchez Ocharan (2016); Sprague (1931); Ulibarri (1996).

### ﻿ Gelrebia

Taxon classificationPlantaeFabalesFabaceae

Gagnon & G.P. Lewis, PhytoKeys 71: 54. 2016.

Figs 35 , 37 , 58

#### Type.

*Gelrebiarubra* (Engl.) Gagnon & G.P. Lewis [≡ *Hoffmannseggiarubra* Engl.]

#### Description.

Erect to scrambling shrubs, armed with scattered, straight, or curved, deflexed prickles. **Stipules** minutes, caducous or lacking. **Leaves** bipinnate, ending in a pair of pinnae; pinnae 1–17 opposite pairs; leaflets 1–33 opposite pairs per pinna [except in *G.glandulosopedicellata* (R.Wilczek) Gagnon & G.P. Lewis], lower surface of the blades with numerous subepidermal glands or translucent dots. **Inflorescence** a terminal or axillary raceme. **Flowers** bisexual, zygomorphic; hypanthium persisting as a wide shallow cup at the pedicel apex as the fruit matures; sepals 5, caducous, lower sepal strongly cucullate (occasionally with a beaked apex), covering the other 4 sepals in bud before anthesis; petals 5, free, dark pinkish mauve to light pinkish white, eglandular; stamens 10, free, filaments pubescent and eglandular; ovary glabrous. **Fruit** a coriaceous, broadly oblong-ovate to obliquely pyriform legume. **Seeds** laterally compressed.

#### Chromosome number.

Unknown.

#### Included species and geographic distribution.

**Figure 58.**
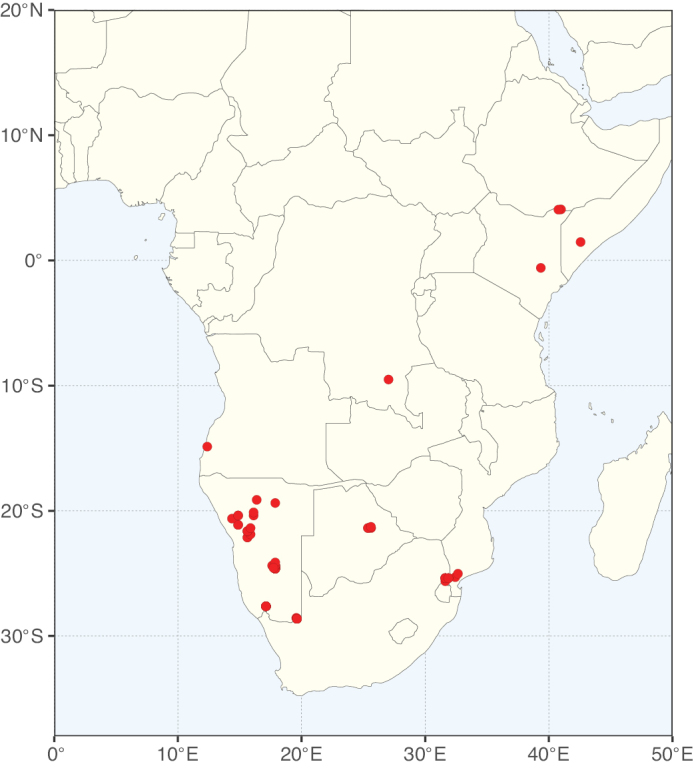
Distribution of *Gelrebia* based on quality-controlled digitised herbarium records. See Suppl. material 1 for the source of occurrence data.

Nine taxa in eight species, restricted to Africa, in Namibia, Angola, Botswana, South Africa, Mozambique, northern Kenya, Ethiopia, Somalia, and the Democratic Republic of the Congo (Zaire, Katanga) (Fig. 58).

#### Ecology.

Deciduous bushlands, dry woodlands, on rocky ridges, often along dry riverbeds, or on sandy valley floors. One species also found in degraded savannas.

#### Etymology.

Gelreb or gelrib is the Somali name for Gelrebiatrothaeisubsp.erlangeri (Harms) Gagnon & G.P. Lewis, meaning ‘camel trap’ and clearly alluding to the robust deflexed prickles characteristic of the species, and indeed the whole genus, which can hinder the passage of camels.

#### Human uses.

Unknown.

#### Taxonomic references.

Brenan (1963a, 1967); Brummitt et al. (2007); Curtis and Mannheimer (2005); Gagnon et al. (2016); Germishuizen (1991); Nkowki and Swelankomo (2003); Ross (1977); Roti-Michelozzi (1957); Thulin (1980, 1983, 1993); Wilczek (1951).

### ﻿ Guilandina

Taxon classificationPlantaeFabalesFabaceae

L., Sp. Pl.: 381. 1753.

Figs 35 , 37 , 59

Bonduc Mill., Gard. Dict. Arb. Ed. 4: 28. 1754.
Caesalpinia subg. Guilandina (L.) Gillis & Proctor, J. Arnold. Arbor. 55(3): 426. 1974. Type: Caesalpiniabonduc (L.) Roxb. [≡ Guilandinabonduc L.]

#### Type.

*Guilandinabonduc* L.

#### Description.

Lianas or scrambling shrubs, generally densely armed with robust recurved prickles. **Stipules** subulate to compound-foliaceous, caducous to persistent. **Leaves** bipinnate, with recurved prickles on leaf and pinnae rachides; pinnae in 3–11 opposite pairs; leaflets 3–24 opposite to alternate pairs per pinna, eglandular. **Inflorescence** a terminal or axillary raceme, often branched near the base. **Flowers** unisexual, segregated on separate staminate and pistillate racemes (sometimes the staminate flowers cryptically hermaphrodite but with the anthers lacking pollen); hypanthium either not persistent in fruit or persisting as a narrow tube closely adhering to the fruit stipe; sepals 5, caducous, valvate in bud, the lower sepal slightly cucullate; petals 5, free, yellow, barely exceeding the sepals; stamens 10, free, pubescent near the filament base; ovary usually covered in bristly trichomes. **Fruits** oblong elliptic, inflated legumes, usually armed with 5–10 mm long slender spinescent bristles. **Seeds** obovoid to globular, ca. 2 cm in diameter, smooth, grey, pale to dark brown, or orange, with parallel fracture lines concentric with the small apical hilum.

#### Chromosome number.

2*n* = 24 (*G.bonduc*) (Fedorov 1969).

#### Included species and geographic distribution.

**Figure 59.**
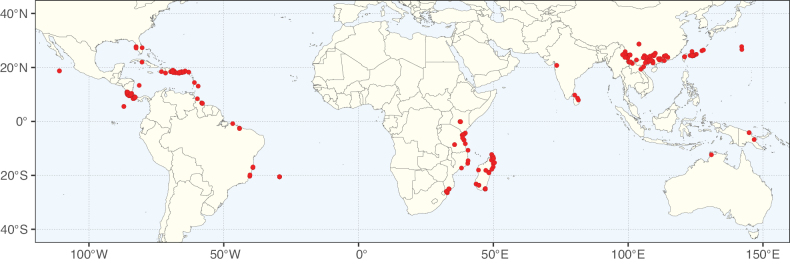
Distribution of *Guilandina* based on quality-controlled digitised herbarium records. See Suppl. material 1 for the source of occurrence data.

Up to 20 species (see notes), widely distributed as far north as some of the southern and eastern Japanese islands, south to South Africa, Madagascar, and Australia. Also occurring in South America and the Caribbean, China, India, Myanmar (Burma), Indo China, Hong Kong, and Taiwan (Fig. 59).

#### Ecology.

Coastal thickets on sand, in secondary forests, and lowland rainforests, occasionally on limestone.

#### Etymology.

Named by Linnaeus for Melchior Wieland (1515–1589), Prussian naturalist, traveller, and scholar from Königsberg, who settled in Italy and italianised his name to ‘Guilandini’, or Guilandinus in Latin; he was sent to the Levant, Asia, and Africa (1559–1560), was captured by pirates and finally ransomed by Gabriele Falloppio.

#### Human uses.

There are reports of the genus being poisonous, although some species are used medicinally. The large globose seeds of *G.bonduc* and *G.major* are used as marbles, necklace beads and buttons (Lewis 2005b).

#### Notes.

*Guilandina* lacks a recent global taxonomic account and there are doubts about the number of species, with estimates ranging from seven to 20. The genus needs to be revised with particular attention given to species delimitation, synonymy, and geographical distribution. Six *Caesalpinia* binomials included as belonging to *Guilandina* in Gagnon et al. (2016), together with *Caesalpiniarobusta* (C.T. White) Pedley, were formally transferred to *Guilandina* by Lewis (2020b). One of those, *Guilandinahomblei* (R. Wilczek) G.P. Lewis, is now recognised as a synonym of *Guilandinabonduc* L. (Robrecht et al. 2021).

#### Taxonomic references.

Brenan (1967); Britton and Rose (1930); Chen et al. (2010a); Du Puy and Rabevohitra (2002); Gagnon et al. (2016); Gillis and Proctor (1974); Hattink (1974); Lewis (2005b, 2020b); Vidal and Hul Thol (1976); Wilczek (1951).

### ﻿ Hultholia

Taxon classificationPlantaeFabalesFabaceae

Gagnon & G. P. Lewis, PhytoKeys 71: 58. 2016.

Figs 35 , 37 , 60

#### Type.

*Hultholiamimosoides* (Lam.) Gagnon & G. P. Lewis [≡ *Caesalpiniamimosoides* Lam.]

#### Description.

Climbing woody shrub; branches densely armed with short, robust, needle-like trichomes; young stems pubescent, with rust-coloured, hyaline hairs, and dome-shaped glands, topped with a few hairs. **Stipules** subulate, caducous. **Leaves** bipinnate; pinnae in 10–30 opposite pairs, with a pair of deflexed prickles at the insertion of the pinnae on the leaf rachis and at the insertion of leaflets on the pinnae rachides; leaflets 7–20 pairs per pinna, opposite, glabrous, eglandular. **Inflorescence** a terminal or leaf-opposed, lax raceme, with 50 or more flowers. **Flowers** bisexual, zygomorphic; hypanthium persisting as a wide shallow cup at the pedicel apex as fruit matures; sepals 5, caducous, the lower sepal strongly cucullate; petals 5, free, bright yellow, dark glands present on the blade, median petal smaller than the 4 lateral petals; stamens 10, free, filaments pubescent at least on the lower half; ovary densely pubescent and with glandular dots (often obscured by the dense pubescence). **Fruit** an obovoid, falcate, vesicular, unarmed, dehiscent legume, sparsely pubescent, particularly along the margin, and with a few obscure stellate hairs, and covered in gland dots, 1–3-seeded. **Seeds** sub-globose, grey.

#### Chromosome number.

2*n* = 24 (Fedorov 1969).

#### Included species and geographic distribution.

**Figure 60.**
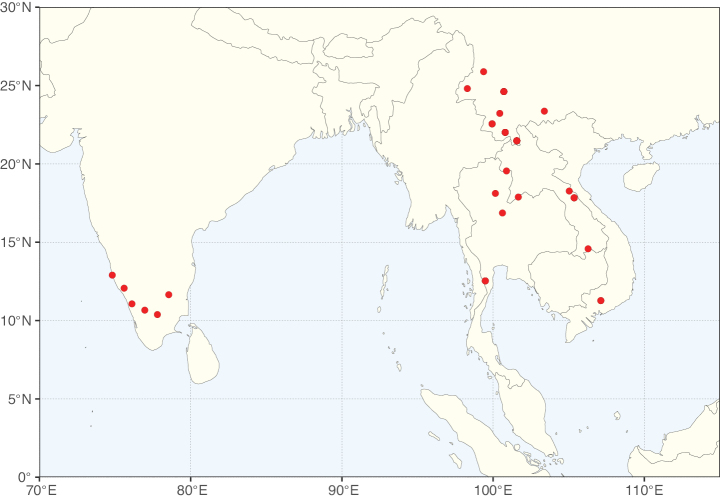
Distribution of *Hultholia* based on quality-controlled digitised herbarium records. See Suppl. material 1 for the source of occurrence data.

Monospecific (*H.mimosoides*), distributed across Asia, in China (Yunnan), Bangladesh, India, Laos, Myanmar (Burma), Thailand and Vietnam (Fig. 60).

#### Ecology.

In secondary thickets and clearings, often on roadsides.

#### Etymology.

The name *Hultholia* honours the Cambodian botanist Dr. Sovanmoly Hul Thol (born 1946), whose doctoral thesis, “Contribution à la révision de quelques genres de Caesalpiniaceae, representés en Asie” (Hul Thol 1976), is an important revision of the Asian species and genera of the Caesalpinieae, and particularly the genus *Pterolobium*.

#### Human uses.

Although *Hultholiamimosoides* is not known to be cultivated, the young, pungent, flowering shoots are sold as a vegetable in markets in Vientiane (Laos) (Vidal and Hul Thol 1976).

#### Notes.

This monospecific genus was described by Gagnon et al. (2016) and has an unresolved phylogenetic position within a clade of lianescent Old World genera.

#### Taxonomic references.

Chen et al. (2010a); Gagnon et al. (2016); Vidal and Hul Thol (1976).

### ﻿ Moullava

Taxon classificationPlantaeFabalesFabaceae

Adans., Fam. Pl. 2: 318. 1763.

Figs 37 , 38 , 61

Almeloveenia Dennst., Schlüssel Hortus Malab.: 32. 1818. Type: Almeloveeniaspinosa Dennst. [= Moullavaspicata (Dalzell ex Wight) Nicolson]
Cinclidocarpus Zoll. & Moritzi, Natuur-Geneesk. Arch. Ned.-Indië iii: 81. 1846. Type: Cinclidocarpusnitidus Zoll. & Moritzi [= Moullavatortuosa (Roxb.) Gagnon & G.P. Lewis]
Wagatea Dalzell, Hooker’s J. Bot. Kew Gard. Misc. 3: 90. 1851. Type: Wagateaspicata Dalzell ex Wight [≡ Moullavaspicata (Dalzell ex Wight) Nicolson]
Caesalpinia sect. Cinclidocarpus (Zoll. & Moritzi) Benth. & Hook., Gen. Pl. 6.: 565–567. 1865. Type not designated.

#### Type.

*Moullavaspicata* (Dalzell ex Wight) Nicolson [≡ *Wagateaspicata* Dalzell ex Wight]

#### Description.

Lianas and scrambling shrubs, armed with deflexed prickles. **Leaves** bipinnate, ending with a pair of pinnae; pinnae in 7–20 opposite pairs; leaflets in 5–40 opposite pairs per pinna. **Stipules** caducous or lacking (not seen). **Inflorescence** an elongated terminal or axillary raceme, the racemes sometimes aggregated into panicles. **Flowers** bisexual, sub-actinomorphic or zygomorphic; hypanthium persisting either as a distinct cup or as a wide shallow calyx remnant at the pedicel apex as the fruit matures; sepals 5, caducous, eglandular, glabrous, the lower sepal strongly cucullate, covering the other 4 sepals in bud; petals 5, free, yellow, the median (innermost upper) and lateral petals sometimes streaked red, eglandular; stamens 10, free, barely exserted beyond the corolla, densely pubescent on lower half of filaments; ovary glabrous or pubescent. **Fruit** fleshy, oblong-elliptic, unarmed, indehiscent, sub-torulose, with thickened sutures, drying black (immature fruits of *M.spicata* red-tomentose), exocarp and endocarp strongly adnate, glabrous, 1–4-seeded. **Seeds** sub-globular, olive-brown to black.

#### Chromosome number.

2*n* = 24 [*M.digyna* (Rottler) Gagnon & G.P. Lewis, *M.spicata*] (Rice et al. 2015).

#### Included species and geographic distribution.

**Figure 61.**
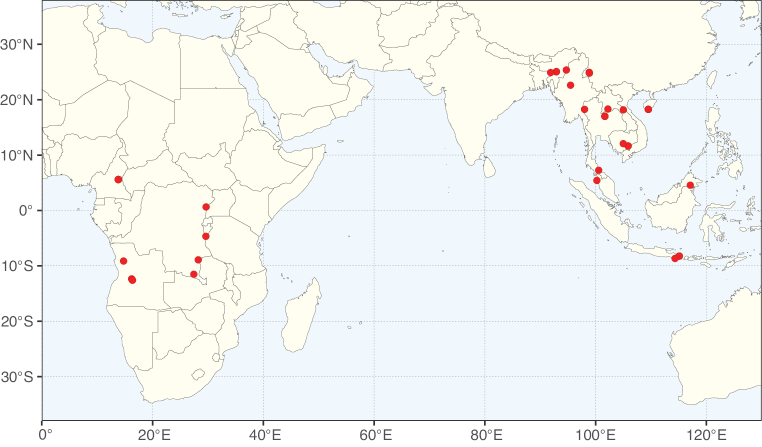
Distribution of *Moullava* based on quality-controlled digitised herbarium records. See Suppl. material 1 for the source of occurrence data.

Four species, three in southern Asia and one in Africa (Fig. 61).

#### Ecology.

The Asian species are found in seasonally dry tropical semi-evergreen forest margins, secondary thickets, and on mountain slopes, up to 1200 m elevation. The African species occurs mostly in riverine habitats in lowland rainforests.

#### Etymology.

Derived from the vernacular name of *Moullavaspicata*, “mulu” (Malayalam: spiny), a spiny climber.

#### Human uses.

*Moullavaspicata* is used for medicine (Lewis 2005b).

#### Notes.

First described as a monospecific genus from India, its description was emended in Gagnon et al. (2016) to include three other species, all with similar torulose fruits, not found elsewhere in the Caesalpinieae.

#### Taxonomic references.

Ansari (1990); Brenan (1963a, 1967); Brummitt et al. (2007, see both *Moullava* and *Mezoneuronwelwitschianum* Oliv.); Chen et al. (2010a); Gagnon et al. (2016); Hattink (1974); Lewis (2005b); Nicolson (1980); Sanjappa (1992); Vidal and Hul Thol (1976).

### ﻿ Biancaea

Taxon classificationPlantaeFabalesFabaceae

Tod., Nuovi Gen. Sp. Orto Palermo: 21. 1860.

Figs 37 , 38 , 62

Campecia Adans., Fam. Pl. (Adanson) 2: 318. 1763 (no type species designated, and no species names ever published in this genus. It is thus not possible to apply this name which is rejected against Biancaea).
Caesalpinia sect. Sappania DC., Prodr. [A. P. de Candolle] 2: 484. 1825. Type not designated.

#### Type.

*Biancaeascandens* Tod. [= *Biancaeadecapetala* (Roth) Deg.]

#### Description.

Lianas, climbing or trailing shrubs or small trees, armed with short, slightly recurved prickles. **Stipules** lanceolate-oblong to broadly ovate, sometimes amplexicaul at base, caducous or persistent. **Leaves** bipinnate, ending with a pair of pinnae, rachis armed with pairs of prickles at the base of each pinna, sometimes also scattered on the rachis; pinnae in 4–19 opposite or alternate pairs; leaflets in 5–20 opposite or alternate pairs per pinna. **Inflorescence** a terminal or axillary raceme or panicle. **Flowers** bisexual, zygomorphic; hypanthium persisting as a small cup or wider shallow cup or occasionally as an abscised free ring around the pedicel apex as the fruit matures; sepals 5, caducous, usually pubescent, the lower sepal cucullate and covering the other 4 in bud; petals 5, free, yellow to white, eglandular, the median petal smaller than the other 4, and inrolled towards the centre; stamens 10, filaments densely pubescent especially at the base; ovary densely velutinous. **Fruit** a coriaceous, pubescent, glabrescent or glabrous, eglandular, dehiscent, wingless, laterally compressed (but somewhat inflated and often with a narrow wing along the upper suture in *B.decaptala*), 1–9-seeded legume. **Seeds** flat, black, or brown.

#### Chromosome number.

2*n* = 24 (*B.decapetala*, *B.sappan*) (Kumari and Bir 1989).

#### Included species and geographic distribution.

**Figure 62.**
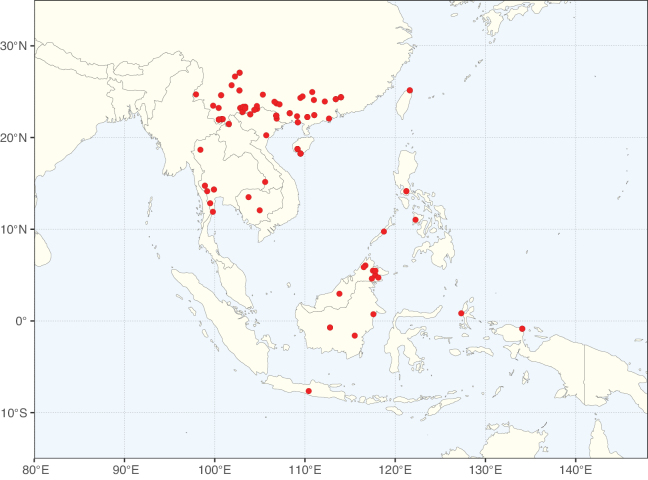
Distribution of *Biancaea* based on quality-controlled digitised herbarium records of native specimens. See Suppl. material 1 for the source of occurrence data.

Six species widespread across southern Asia. *Biancaeadecapetala*, native to Asia, has been widely introduced across the tropics as a hedge plant or ornamental and is invasive in South Africa and Hawaii (Fig. 62).

#### Ecology.

Primary forest and forest margins, grasslands, scrub vegetation, riverine habitats, secondary thickets, and clearings. From the coast to mountain slopes.

#### Etymology.

It is presumed that the Italian botanist Agostino Todaro (1818–1892) named *Biancaea* for his fellow countryman and botanist Giuseppe Bianca (1801–1883).

#### Human uses.

*Biancaeadecapetala* often grown as a living fence (Hattink 1974).

#### Notes.

Gagnon and Lewis in Gagnon et al. (2016) re-established and emended the description of the genus. A new species, *Biancaeascabrida* L.M. Choo, was published in 2021 (Choo 2021).

#### Taxonomic references.

Brummitt et al. (2007); Chen et al. (2010a); Gagnon et al. (2016); Hattink (1974); Jansen (2005); Molinari-Novoa et al. (2016b); Vidal and Hul Thol (1976).

### ﻿ Pterolobium

Taxon classificationPlantaeFabalesFabaceae

R. Br. ex Wight & Arn., Prodr: 283. 1834 nom. cons.

Figs 35 , 37 , 63

Cantuffa J.F. Gmel., Syst. Nat., ed 13[bis]. 2(1): 677. 1791, nom. rej. vs. Pterolobium R. Br. ex Wight & Arn. Type: Cantuffaexosa J.F. Gmel. [≡ Pterolobiumexosum (J.F. Gmel.) Baker f. (= Pterolobiumstellatum (Forssk.) Brenan)]
Reichardia Roth, Nov. Pl. Sp.: 210. 1821, nom. illeg., non Roth, Bot. Abh. Beobacht. 35. 1787, nec Roth, Catal. Bot. 2: 64. 1800. Lectotype: Reichardiahexapetala Roth [≡ Pterolobiumhexapetalum (Roth) Santapau & Wagh]

#### Type.

*Pterolobiumlacerans* R. Br. ex Wight & Arn., nom. illeg. [*Pterolobiumexosum* (J.F. Gmel.) Baker f. [≡ *Cantuffaexosa* J.F. Gmel. (= *Pterolobiumstellatum* (Forssk.) Brenan)]

#### Description.

Lianas or scrambling / trailing shrubs, armed with prickles. **Stipules** small, inconspicuous, subulate to triangular-subulate, caducous. **Leaves** bipinnate; pinnae in 5–20 opposite pairs; leaflets in 6–25 opposite pairs per pinna. **Inflorescence** a terminal or axillary raceme, often aggregated into panicles. **Flowers** bisexual, sub-actinomorphic to zygomorphic; hypanthium persisting as a minute cup or ridge at the pedicel apex as the fruit matures; sepals 5, caducous, the lower sepal cucullate, covering the other 4 sepals in bud; petals 5, free, yellow to white, equal to slightly differentiated, the median petal sometimes in-rolled; stamens 10, free, filaments pubescent (occasionally glabrous); ovary pubescent. **Fruit** a red to brown samara, 1 (2)-seeded. **Seeds** ovate-oblong, sub-compressed.

#### Chromosome number.

2*n* = 24 (*P.stellatum*) (Rice et al. 2015).

#### Included species and geographic distribution.

**Figure 63.**
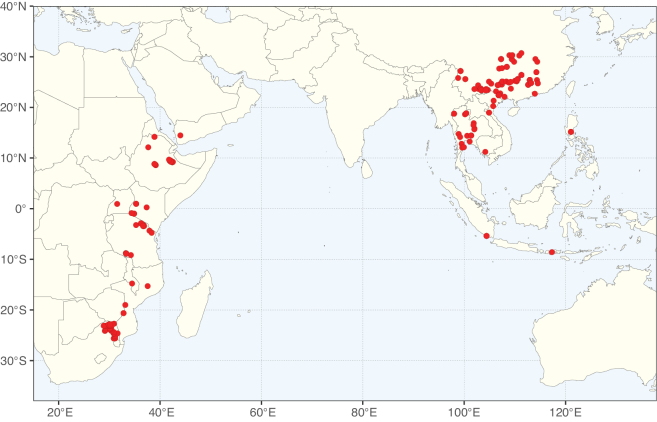
Distribution of *Pterolobium* based on quality-controlled digitised herbarium records. See Suppl. material 1 for the source of occurrence data.

Ten species; one in southern tropical Africa, East Africa, and Arabia, nine in South East Asia (Fig. 63).

#### Ecology.

Seasonally dry tropical upland evergreen forests, riverine and humid forests, woodlands, and wooded grasslands.

#### Etymology.

From Greek, *ptero*- (= wing) and *lobion* (= fruit), in reference to the fruit which resembles a samara.

#### Human uses.

The leaves of *P.stellatum* are used in parts of Africa as a leather dye, for ink and as medicine; plants are grown as living fences (Lewis 2005b).

#### Notes.

Vidal and Hul Thol (1974) published a revision of *Pterolobium*, with a key to species.

#### Taxonomic references.

Brenan (1967); Chen et al. (2010b); Gagnon et al. (2016); Hou (1996c); Hul Thol and Hideux (1977); Lewis (2005b); Roti-Michelozzi (1957); Vidal and Hul Thol (1974, 1976).

### ﻿ Mezoneuron

Taxon classificationPlantaeFabalesFabaceae

Desf., Mém. Mus. Hist. Nat. 4: 245. 1818.

Figs 37 , 64

Mezonevron Desf., Mém. Mus. Hist. Nat. 4: 245. 1818, orth. var.
Mezoneurum DC., Prodr. [A.P. de Candolle] 2: 484. 1825, orth. var.
Caesalpinia subg. Mezoneuron (Desf.) Vidal ex Herend. & Zarucchi, Ann. Missouri Bot. Gard. 77(4): 854. 1990.

#### Type.

*Mezoneuronglabrum* Desf. [= *Mezoneuronpubescens* Desf.]

#### Description.

Scrambling shrubs or lianas, occasionally medium-sized trees, usually armed with recurved prickles on stem and leaves, rarely unarmed. **Stipules** triangular, often caducous. **Leaves** bipinnate, ending in a pair of pinnae; pinnae in (1) 2–18 opposite to sub-opposite pairs; leaflets in 1–15 opposite to alternate pairs per pinna. **Inflorescence** a terminal or axillary raceme (often aggregated into panicles). **Flowers** bisexual, zygomorphic; hypanthium persisting as a small cup or wide shallow cup as the fruit matures; sepals 5, imbricate, the lower sepal cucullate, and overlapping the other 4 in bud, caducous; petals 5, free, usually yellow with red markings on the median petal, or occasionally red, pink or cream, the median petal somewhat modified (either with a fleshy ligule or a patch of hairs on the inner surface between the blade and claw, or the petal bilobed); stamens 10, free, usually all pubescent or villose on lower half, or one or all glabrous; ovary glabrous to hairy. **Fruit** laterally compressed, indehiscent, chartaceous, coriaceous or woody, venose, longitudinally (and often broadly) winged along the upper suture, the wing 2–20 mm wide, 1–13-seeded. **Seeds** ± transversely arranged in seed chamber, compressed.

#### Chromosome number.

2*n* = 22 (*M.cucullatum*, *M.kauaiense*) (Rice et al. 2015).

#### Included species and geographic distribution.

**Figure 64.**
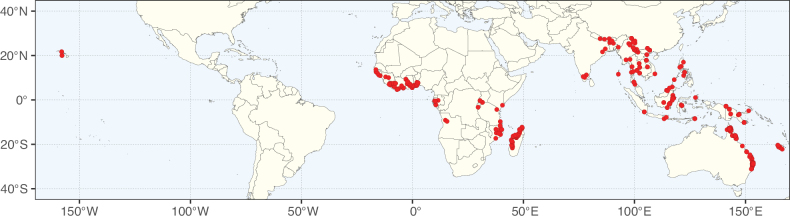
Distribution of *Mezoneuron* based on quality-controlled digitised herbarium records. See Suppl. material 1 for the source of occurrence data.

Twenty-four species, mainly in Asia, extending to Australia, Polynesia (including Hawaii), New Caledonia, Madagascar and Africa (Fig. 64).

#### Ecology.

Tropical and subtropical riverine forests, lowland rainforests, swamp forests, seasonally dry forests, thickets, vine forests and wooded grasslands, especially along forest and river margins.

#### Etymology.

From Greek, *meso*- (= middle) or *meizon* (= greater) and *neuron* (= nerve), the upper suture of the fruit is bordered by a usually broad longitudinal wing so that the suture appears as a prominent sub-central nerve or vein.

#### Human uses.

Used for medicine; the wood of *M.kauaiense* (Mann.) Hillebr. (“uhiuhi”) from Hawaii was once used locally for spears and in house construction (Lewis 2005b).

#### Notes.

*Mezoneuron* is absent from the American tropics and subtropics today, but fossil records show that the genus was widespread across North America, as well as in Europe, during the middle Eocene, about 45 Ma (Herendeen and Dilcher 1991). A revision of *Mezoneuron* by Clark (2016) provides full synonymy, a key to species, and a list of fossil taxa associated with this genus.

#### Taxonomic references.

Brenan (1967); Brummitt et al. (2007); Clark (2016); Clark and Gagnon (2015); Du Puy and Rabevohitra (2002); Gagnon et al. (2016); George (1998); Hattink (1974); Herendeen and Zarucchi (1990); Lock (1989); Pedley (1997); Verdcourt (1979); Vidal and Hul Thol (1976); Wagner et al. (1999).

### ﻿ Ticanto

Taxon classificationPlantaeFabalesFabaceae

Adans., Fam. Pl. 2: 319. 1763.

Figs 37 , 65

Caesalpinia sect. Nugaria DC., Prodr. [A.P. de Candolle] 2: 481. 1825. Type not designated.

#### Type.

*Guilandinapaniculata* Lam. [= *Ticantocrista* (L.) R. Clark & Gagnon]

#### Description.

Scandent shrubs or lianas to 15 m. Stems usually with scattered, recurved prickles. **Stipules** triangular, caducous. **Leaves** bipinnate, leaf rachis with recurved prickles at base of pinnae and usually scattered in between, pinnae in 1–16 opposite pairs; leaflets in 2–15 opposite pairs per pinna. **Inflorescence** a terminal or axillary raceme or panicle, bracts (at base of racemes) and bracteoles (at base of pedicels) caducous; pedicels articulated. **Flowers** bisexual, zygomorphic; hypanthium persisting as a small cup as the fruit matures; sepals 5, caducous, the lower lobe cucullate over the others in bud; petals 5, the median petal distinct from the others in shape, usually with a ± circular patch of hairs on the inner surface, the lateral petals glabrous or with few hairs; stamens 10, free, the basal half tomentose; ovary 1–2-ovuled, glabrous or hairy, stigma funnel-shaped and more or less papillate, or truncate. **Fruit** coriaceous or ligneous, dehiscent or indehiscent, elliptic, lunate, or suborbicular, the upper suture lacking a wing, or with a narrow wing up to 4 mm wide, one species with a carinate wing 5–6 mm deep, fruit usually glabrous, otherwise tomentose, 1 (2)-seeded. **Seeds** circular to reniform, compressed.

#### Chromosome number.

2*n* = 24 (*T.crista*) (Fedorov 1969).

#### Included species and geographic distribution.

**Figure 65.**
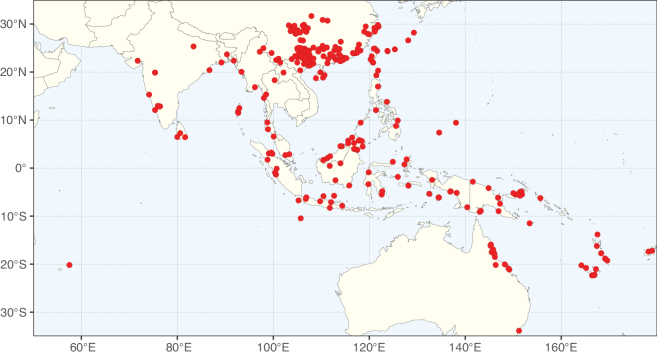
Distribution of *Ticanto* based on quality-controlled digitised herbarium records. See Suppl. material 1 for the source of occurrence data.

Nine species, mainly in southern and central China, with a few records in Laos, Myanmar, northern Thailand, and northern Vietnam, and one species (*T.crista*) throughout South East Asia, extending into Australia and Oceania (Fig. 65).

#### Ecology.

Scrub or (riverine) forests, on limestone or in sandy parts of mangroves, up to 1800 m elevation.

#### Etymology.

*Ticanto* was a vernacular name used for these plants by the Brachmanes (Brahmans or Brahmins), a sector of Hinduism.

#### Human uses.

Numerous studies report pharmacological uses of *Caesalpiniacrista* but appear to concern the species *Guilandinabonduc* to which the name *Caesalpiniacrista* is often erroneously applied. Confirmed uses of true *C.crista* are not known to the present authors.

#### Notes.

*Ticanto* is a genus recently re-instated by Clark et al. (2022), who provide a taxonomic revision of the genus including a key to the species. Previously, a single binomial was published under this genus name, *Ticantonuga* (L.) Medik., now a synonym of *Ticantocrista*.

#### Taxonomic references.

Adanson (1763); Chen et al. (2010a); Clark et al. (2022); Gagnon et al. (2016); Hattink (1974); Rumphius (1747); Van Rheede (1686); Vidal and Hul Thol (1976).

## ﻿﻿7. Tribe Schizolobieae

Colin E. Hughes^3^, Elspeth Haston^18^

Citation: Hughes CE, Haston E (2024) 7. Tribe Schizolobieae In: Bruneau A, Queiroz LP, Ringelberg JJ (Eds) Advances in Legume Systematics 14. Classification of Caesalpinioideae. Part 2: Higher-level classification. PhytoKeys 240: 146–164. https://doi.org/10.3897/phytokeys.240.101716

### ﻿Tribe Schizolobieae

Taxon classificationPlantaeFabalesFabaceae

Nakai, Chosakuronbun Mokuroku [Ord. Fam. Tribe. Nov.]: 251. 1943.

Figs 66 , 67 , 68 , 69 , 70 , 71 , 72 , 73 , 74 , 75 , 76 , 77

#### Type.

*Schizolobium* Vogel, Linnaea 11: 399. 1837.

#### Included genera

**(8).***Bussea* Harms (7 species), *Colvillea* Bojer ex Hook. (1), *Conzattia* Rose (1), *Delonix* Raf. (12), *Heteroflorum* M. Sousa (1), *Parkinsonia* L. (12), *Peltophorum* (Vogel) Benth. (6–7), *Schizolobium* Vogel (2).

#### Description.

Trees or occasionally shrubs, (3) 5–40 m and up to 1.5 m stem diameter, typically branching low down with spreading, flat-topped crowns, some species of *Delonix* with swollen trunks; outer bark generally thin, pale silvery grey, or green, inner bark green; mainly unarmed, occasionally armed with axillary thorns, stipular spines and/or spinescent leaf rachides and spinescent side shoots; brachyblasts usually absent, rarely present. **Stipules** absent, minute, setiform, acicular and caducous, or lobed, pinnatifid or bipinnatifid, and persistent, or spinescent and persistent. **Leaves** bipinnate, variable in leaf formula, sometimes large, rarely paripinnate and much reduced. **Inflorescences** axillary racemes or terminal panicles, pedicels usually jointed. **Flowers** usually showy, almost always yellow, occasionally orange, whitish-pink or red, usually bisexual, but sometimes unisexual (*Heteroflorum*, *Conzattia*, and *Parkinsoniaanacantha* Brenan are at least partially dioecious); a distinct hypanthium always present, this generally short, discoid or shallowly campanulate, but larger and obliquely turbinate in *Schizolobium*; sepals 5, subequal, either imbricate or valvate, free or partially fused, usually strongly reflexed; petals usually 5, occasionally 1, subequal or variable, longer or shorter clawed, usually crinkled, often with erose margins; stamens 10, usually spreading, rarely clustered around the ovary; pollen in oblate tricolporate monads with moderately to coarsely reticulate surface ornamentation. **Fruits** diverse, 1–many-seeded, usually flat, but sometimes terete or thickened, generally linear or linear-oblong or spathulate oblanceolate, the valves papery, coriaceous or woody, sutures sometimes thickened or with a narrow wing, indehiscent or dehiscent along both sutures, sometimes tardily so, usually inertly so, or rarely elastically from the apex. **Seeds** oblong-ellipsoid or ovate, discoid, lenticular and flattened to sub-spherical or globose, albuminous or ex-albuminous, integument hard.

#### Distribution.

Tribe Schizolobieae occupies a strikingly disjunct pantropical distribution spanning dry and wet tropical forests and savannas.

#### Clade-based definition.

**Figure 66.**
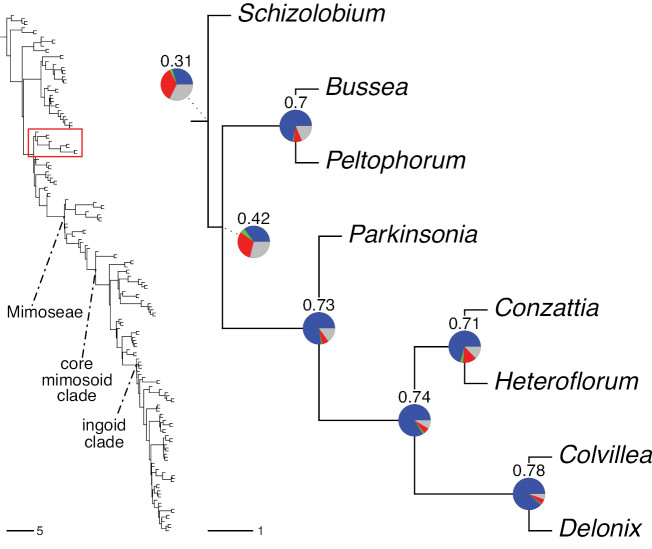
Generic relationships in tribe Schizolobieae. For description of phylogeny and support values, see Fig. 6 caption (page 63).

The most inclusive crown clade containing *Schizolobiumparahyba* (Vell.) S.F. Blake and *Delonixdecaryi* (R. Vig.) Capuron, but not *Caesalpiniabrasiliensis* L., *Dimorphandraconjugata* (Splitg.) Sandwith, or *Mimosasensitiva* L. (Fig. 66).

#### Notes.

Tribe Schizolobieae is here delimited to include eight genera and ca. 45 species. The monophyly and generic contents of this clade were first established using molecular data by Haston et al. (2003, 2005), and confirmed by Manzanilla and Bruneau (2012) and LPWG (2017), as well as in more recent phylogenomic analyses (Ringelberg et al. 2022) which show robust support for this clade (Fig. 66). Previously, these eight genera were spread across the informal Peltophorum and Caesalpinia groups of Polhill and Vidal (1981), while later Polhill (1994) expanded his concept of the informal Peltophorum group to include eight of these genera, but also eight other genera that are now placed in three other tribes, reflecting the poor understanding of generic relationships at that time across the then broadly circumscribed tribe Caesalpinieae.

Tribe Schizolobieae is equivalent to the Peltophorum group s.s. of Haston et al. (2005) and the informal core Peltophorum group of Lewis (2005b) with three minor modifications. First, the addition of the genus *Heteroflorum* which, although mentioned by Lewis (2005b) and Haston et al. (2005), was only formally published that same year (Sousa 2005). Second, new name combinations in *Parkinsonia* are now available for all species previously placed in the genus *Cercidium* Tul. (Romão and Mansano 2021), which was shown to be nested within *Parkinsonia* (Haston et al. 2003, 2005). Third, the genus *Lemuropisum* H. Perrier was shown to be nested within *Delonix* and sunk within that genus by Babineau and Bruneau (2017). In recent phylogenomic analyses (Ringelberg et al. 2022), generic relationships within the tribe are also robustly supported (Fig. 66).

**Figure 67.**
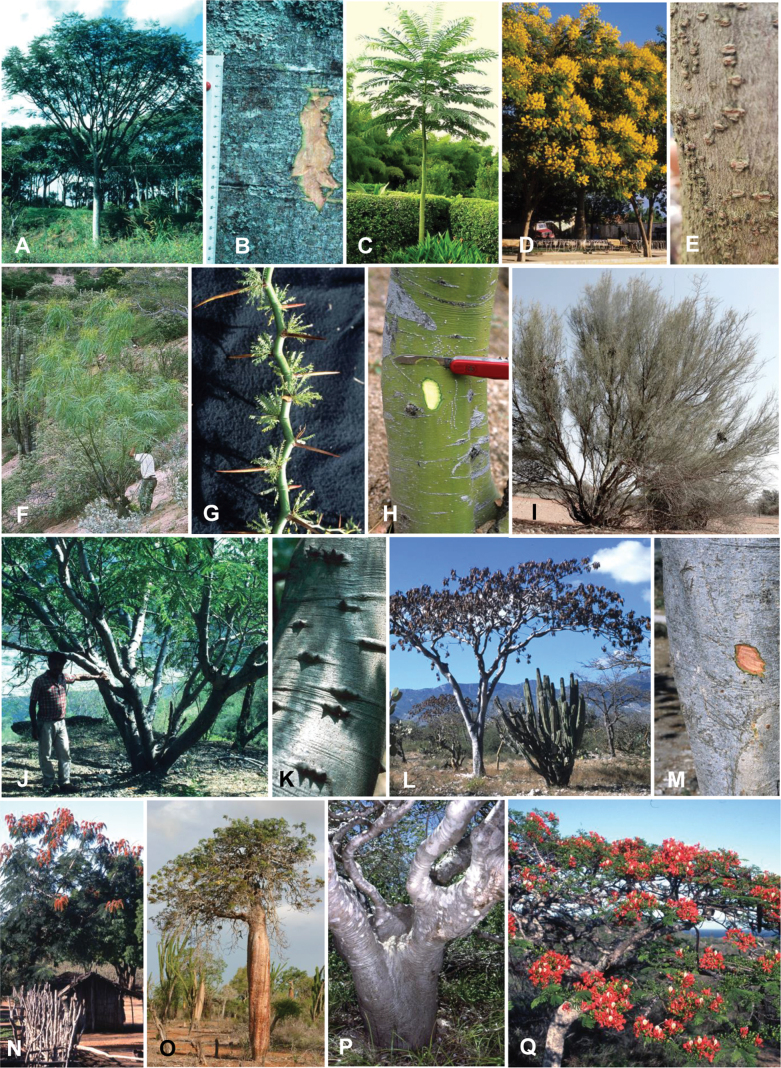
Variation in tree form and bark among genera of tribe Schizolobieae**A, B***Schizolobiumamazonicum* Huber ex Ducke, tree to 20 m, Veracruz, Mexico (*Hughes 1880*) **C** young sapling of *Schizolobiumamazonicum* showing typical ‘tree fern habit’ with large leaves and green bark **D***Peltophorumdubium* (Spreng.) Taub., cultivated as an ornamental, central plaza in Saipina, Santa Cruz, Bolivia (*Hughes 2461*) **E** bark of *Peltophorumafricanum* Sond. **F***Parkinsoniaperuviana* C.E. Hughes, Daza & Hawkins, small tree in dry thorn scrub in the upper Marañon valley, Peru (*Hughes 2213*) **G** green shoot and axillary thorns, *Parkinsoniaandicola* (Griseb.) Varjão & Mansano, Chuquisaca, Bolivia (*Hughes 2313*) **H** green bark of *Parkinsoniapraecox* (Ruiz & Pavon ex Hook.) Hawkins, Oaxaca, Mexico (*Hughes 1298*) **I** tree of *Parkinsoniaafricana* Sond., South Africa **J, K** small tree of *Heteroflorumsclerocarpum* M. Sousa showing typical low forked branching habit, spreading crown and pale metallic grey bark with storied protuberences, Guerrero, Mexico (*Hughes 1845*) **L, M***Conzattiamultiflora* Standl., showing typical umbrella crown habit and pale grey metallic bark in succulent-rich seasonally dry forest, Puebla, Mexico (*Hughes 1824*) **N** small tree of *Colvillearacemosa* Bojer, Madagascar (*Du Puy 102*) **O** bottle tree of *Delonixfloribunda* (Baill.) Capuron, south-western Madagascar (*Bruneau 1405*) **P** twisted and stunted growth form of *Delonixpumila* Du Puy, Phillipson & R. Rabev. in south-western Madagascar (*Du Puy 456*) **Q** small tree of *Delonixregia* (Bojer) Raf. with a spreading crown, flowering preceding or coinciding with leaf flush, N Madagascar (*Du Puy 578*). Photo credits **A, B, D, F, G, H, J, K, L, M** CE Hughes **C** A Bayer Tamayo File:Tambor (Schizolobiumparahyba) (14101065740).jpg - Wikimedia Commons **E** W Coville (https://plantnet.org/) **I** Wi Boshoff http://hdl.handle.net/11660/5647**N, P, Q** D Du Puy **O** A Bruneau.

Although varying markedly in stature, from shrubs and small trees to gigantic, buttressed trees up to 40 m tall in *Schizolobium*, all species of tribe Schizolobieae share a characteristic tree form and bark (Fig. 67). Trees are typically forked low down with spreading umbrella-like, flat-topped crowns (Fig. 67). The outer bark is generally smooth, thin and either pale silvery metallic grey, or, in most species of *Parkinsonia* and young trees of *Schizolobium*, green (Fig. 67).

Leaf formula of the mainly bipinnate leaves (only *Delonixedule* has once-pinnate leaves) is highly variable among genera of Schizolobieae, ranging from highly reduced leaves with very short rachides and very reduced leaflets in some species of *Parkinsonia*, to what are some of the largest leaves of any genus in subfamily Caesalpinioideae, at 1–2 m long on young saplings of *Schizolobium* (Fig. 67C) and in *Colvillea*, which has leaves on non-flowering juvenile shoots with up to 2,000 leaflets.

**Figure 68.**
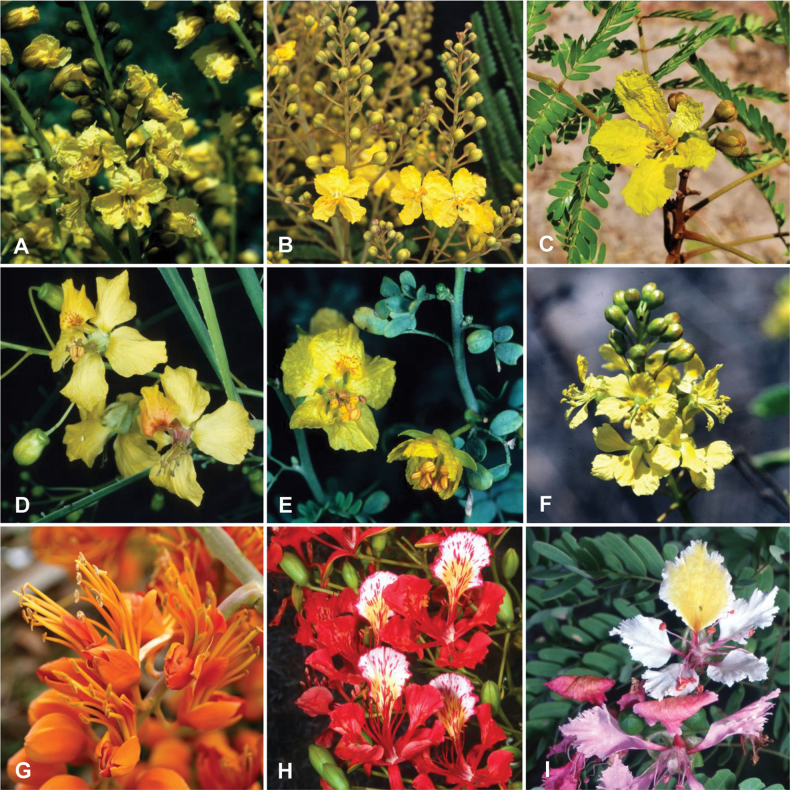
Flowers of tribe Schizolobieae**A***Schizolobiumamazonicum* Huber ex Ducke, Veracruz, Mexico (*Hughes 1880*) **B***Peltophorumdubium* (Spreng.) Taub., Santa Cruz, Bolivia (*Hughes 2461*) **C***Busseaperrieri* R. Vig., western Madagascar **D***Parkinsoniaaculeata* L., Zacapa, Guatemala (*Hughes 1222*) **E***Parkinsoniaflorida* (Benth. ex A. Gray) S. Watson, Sonora, Mexico (*Hughes 1562*) **F***Conzattiamultiflora* Standl., Puebla, Mexico (*Hughes 931*) **G***Colvillearacemosa* Bojer, south-western Madagascar (*Bruneau 1403*) **H***Delonixregia* (Bojer) Raf., northern Madagascar (*Du Puy 578*) **I**Delonixleucanthasubsp.gracilis Du Puy, Phillipson & R. Rabev., Madagascar (*Du Puy 87*). Photo credits **A, B, D–F** CE Hughes **C** feno, iNaturalist (https://www.inaturalist.org/photos/103942022) **G** A Bruneau **H, I** D Du Puy.

The petals of flowers of all genera are showy, mainly bright yellow, but in some species of *Delonix* and *Peltophorum* orange, whitish-pink or red (Fig. 68). Species of several genera are widely introduced and cultivated as ornamental trees in streets and gardens across the tropics. This includes the Flamboyant or Flame tree [*Delonixregia* (Bojer ex Hook.) Raf.] (Fig. 67Q), one of the most widely cultivated ornamental trees in the tropics (Lewis 2020a), several species of *Peltophorum* (Fig. 67D) and, in more arid regions, *Parkinsonia*, especially *P.aculeata* L. The showy flowers of several Schizolobieae have been the focus of botanical paintings (e.g., *Colvillearacemosa* Bojer, Bojer 1834; *Delonixregia*, Bojer 1829; Lewis 2020a). Species of several genera are naturalised and invasive following introductions, most notably *Parkinsoniaaculeata* in northern Australia (Hawkins et al. 2007).

While Schizolobieae can be found in all tropical vegetation types, the greatest diversity of genera and species occurs in the trans-continental succulent biome sensu Schrire et al. (2005a) which spans the Neotropics, Africa and Madagascar (Fig. 67F, I, L, O). Indeed, the robustly supported core clade comprising the genera *Parkinsonia* (amphi-Atlantic), *Conzattia* and *Heteroflorum* (endemic to Mexico), and *Colvillea* and *Delonix* (restricted to East Africa and Madagascar), represents one of the most striking examples of trans-continental phylogenetic succulent biome conservatism (Ringelberg et al. 2020). The only exceptions in Schizolobieae to this overall predilection for the dry tropics are *Schizolobium* with two species in Neotropical wet forests and subsets of species of *Bussea* and *Peltophorum* in wet forests in Africa and Asia.

### ﻿ Schizolobium

Taxon classificationPlantaeFabalesFabaceae

Vogel, Linnaea 11: 399. 1837.

**Figure 69.**
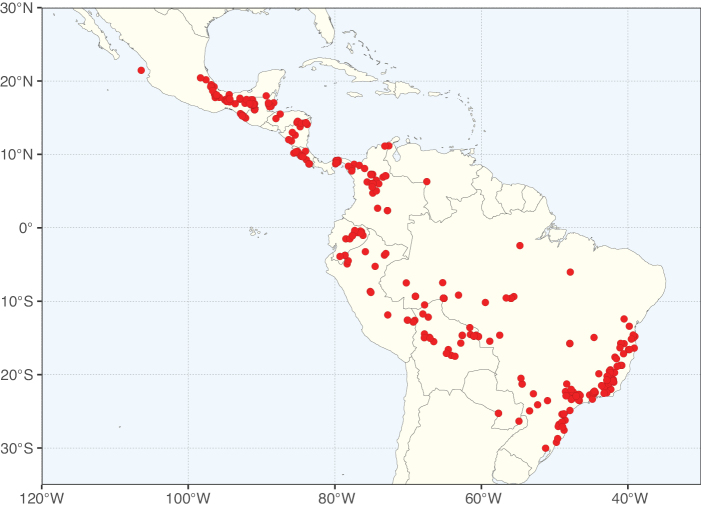
Distribution of *Schizolobium* based on quality-controlled digitised herbarium records. See Suppl. material 1 for the source of occurrence data.

**Figure 70.**
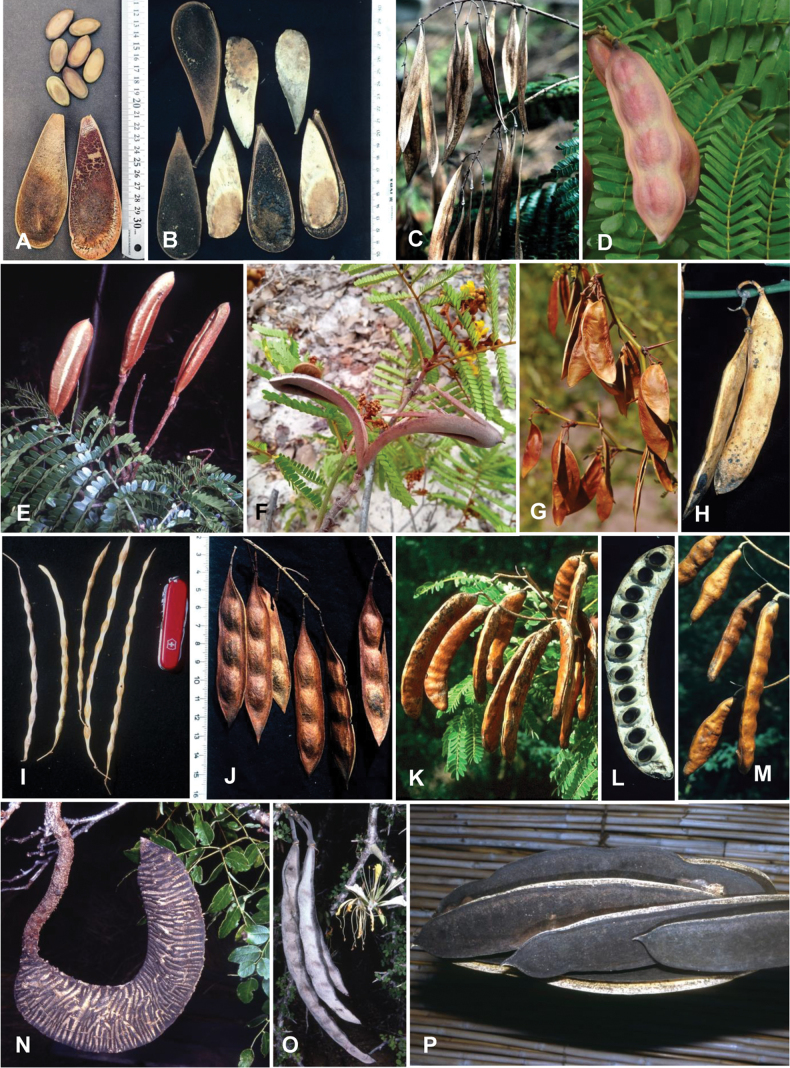
Fruits of tribe Schizolobieae**A, B** dehisced fruits of *Schizolobiumamazonicum* Huber ex Ducke showing the papery endocarp surrounding the single seed, Veracruz, Mexico (*Hughes 1880*) **C***Peltophorumdubium* (Spreng.) Taub., Chiapas, Mexico (*Hughes 1685*) **D***Peltophorumpterocarpum* (DC.) Backer ex K. Heyne, India **E***Busseasakalava* Du Puy & R. Rabev., Madagascar (*Du Puy 248*) **F** dehisced fruit of *Busseaperrieri* R. Vig., north-west Madagascar **G** ripe, tardily dehiscent fruits of *Parkinsoniaandicola* (Griseb.) Varjão & Mansano, Chuquisaca, Bolivia (*Hughes 2619*) **H***Parkinsoniaflorida* (Benth. ex A. Gray) S. Watson, Sonora, Mexico (*Hughes 1562*) **I** ripe indehiscent fruits of *Parkinsoniaperuviana* C.E. Hughes, Daza & Hawkins, La Libertad, Peru (*Eastwood 82*) **J***Conzattiamultiflora* Standl., Puebla, Mexico (*Hughes 1815*) **K–M** indehiscent ripe fruits of *Heteroflorumsclerocarpum* M. Sousa, Guerrero, Mexico (*Hughes 1845*) **L***Heteroflorumsclerocarpum* Guerrero, Mexico (*Hughes 1845*) showing a legume that has been broken apart manually **N***Delonixboiviniana* (Baill.) Capuron, Madagascar (*D Du Puy 301*) **O***Delonixedule* (H. Perrier) Babineau & Bruneau, ripe fruits, Madagascar (*Du Puy 449*) **P***Colvillearacemosa* Bojer, ripe dehiscing fruits, Madagascar (*Du Puy 304*). Photo credits **A–C, G–M** CE Hughes **D** D Valke File:Peltophorumpterocarpum (2745008096).jpg - Wikimedia Commons **E, N–P** D Du Puy **F** Andry.A.R, iNaturalist (https://www.inaturalist.org/photos/28896062).

Figs 67 , 68 , 69 , 70

#### Type.

*Schizolobiumexcelsum* Vogel [= *Schizolobiumparahyba* (Vell.) S.F. Blake]

#### Description.

Unarmed trees, 25–40 m, the trunk frequently 50 cm and up to 150 cm diameter, often buttressed, saplings typically unbranched to 2–3 m bearing at the apex a cluster of huge, tree fern-like leaves (Fig. 67C), mature trees with a large spreading somewhat flat-topped crown and angular branching (Fig. 67A); bark on young trees green (Fig. 67C, H), later the outer bark thin, smooth silvery-grey, inner bark green (Fig. 67B). **Stipules** absent. **Leaves** bipinnate, petiolar gland absent, on young saplings leaves usually very large, up to 2 m long, on mature trees with 15–20 (25) pairs of pinnae and 10–20 pairs of leaflets per pinna, these narrowly-oblong, firm. **Inflorescences** massive terminal racemose-panicles, pedicels jointed above the middle (*S.amazonicum* Huber ex Ducke) or not jointed (*S.parahyba*); bracts caducous. **Flowers** bisexual with an obliquely turbinate hypanthium; sepals 5, free, only weakly imbricate in bud, subequal, reflexing; petals 5, subequal, spreading, clawed, bright yellow, glabrous, oblong-obovate (Fig. 68A); stamens 10, free, equal, equal to or shorter than petals, anthers dorsifixed, glabrous; pollen in oblate tricolporate monads with moderately reticulate surface ornamentation; ovary sessile, affixed to one side of the hypanthium, many ovulate, style filiform, the stigma minute, terminal. **Fruits** single-seeded flattened, spathulate, oblanceolate or spoon-shaped, rounded at the apex and narrowed at the base to the short stipe (Fig. 70A, B), dehiscent, the valves firm-coriaceous, coarsely reticulately-veined and rugose, dark blackish-brown when mature, from which the endocarp is released intact as a thin, papery wing-like envelope the same shape as the fruit (Fig. 70B). **Seeds** large, hard, ca. 20 × 12 mm, oblong-ellipsoid, compressed, pale yellow-brown becoming blackish-brown (Fig. 70A).

#### Chromosome number.

2*n* = 26 (Goldblatt 1981b).

#### Included species and geographic distribution.

Two species, one widespread across the Neotropics from south-central Mexico, Central America, and Amazonia and the second in coastal Brazil from Santa Catarina north to Bahia (Fig. 69).

#### Ecology.

Mainly confined to terra firme in tropical moist forest and often common in secondary forest. Deciduous, at least briefly, usually flowering when leafless just before leaf flush. Seeds wind-dispersed.

#### Etymology.

From Greek, *schizo*- (= split or divided) and -*lobion* (= fruit), in reference to the splitting of the exocarp from the endocarp at maturity.

#### Human uses.

Renowned for its fast growth rate and very light soft wood. Widely cultivated as an ornamental and sometimes as coffee or cacao shade; young saplings, with their up to 2m-long leaves resemble tree ferns (Fig. 67C) and are hence sometimes referred to in English as fern trees.

#### Notes.

*Schizolobium* is sister to the clade comprising the rest of the tribe (Fig. 66). The distinctive spathulate, oblanceolate or spoon-shaped fruits are unique among Caesalpinioideae (Fig. 70A, B). Although Barneby (1996) reduced the two long-recognised species to varieties of *S.parahyba*, this was based on limited material, and it is now clear that these two entities are morphologically distinct and occupy allopatric distributions meriting recognition as separate species.

#### Taxonomic references.

Barneby (1996); Bentham (1870), illustration; Standley and Steyermark (1946).

### ﻿ Bussea

Taxon classificationPlantaeFabalesFabaceae

Harms, Bot. Jahrb. Syst. 33: 159. 1902.

Figs 68 , 70 , 71

#### Type.

*Busseamassaiensis* (Taub.) Harms [≡ *Peltophorummassaiense* Taub.]

#### Description.

Unarmed shrubs or more usually treelets or trees, (2) 6–25 m, with an erect branching habit, the trunk 70–100 cm in diameter, bark smooth, pale whitish-grey, young shoots with a brown or rusty indumentum. **Stipules** small, subulate, caducous. **Leaves** bipinnate, petiolar and rachis glands absent, (1) 8–14 pairs of pinnae and (5) 8–11 (26) pairs of opposite leaflets, these rhombic and unequal-sided, the base truncate, the midvein oblique, dark green and glossy above. **Inflorescences** terminal or subterminal in the upper leaf axils, paniculate or racemose, often combined into a compound terminal inflorescence, the axes minutely velvety brown, pedicels not jointed, bracts mostly caducous (persistent in B.massaiensissubsp.rhodesica). **Flowers** bisexual, weakly zygomorphic; hypanthium shallowly campanulate; sepals 5, imbricate in bud, becoming reflexed, the 3 inner sepals with membranous erose hyaline margins; petals 5, the lateral pairs sub-equal, the upper petal reduced, all petals crinkled, the margins erose, tapering at the base to a short robust claw, bright yellow (Fig. 68C); stamens 10, free, porrect and clustered around the gynoecium, shorter than petals, anthers dorsifixed, glabrous (hairs present in *B.occidentalis*); pollen in oblate tricolporate monads with coarsely reticulate surface ornamentation with sinuous, slightly convoluted muri; ovary subsessile, ovoid, 2–4 ovules, style short and flared to a large peltate capitate stigma. **Fruits** erect, narrow oblong-obovate, compressed with greatly thickened margins, valves woody, with a longitudinal furrow, densely rusty-brown pubescent, elastically dehiscent from the apex, the valves recurving, 1–3-seeded (Fig. 70E, F). **Seeds** compressed, oblong discoid.

#### Chromosome number.

2*n* = 22 (Goldblatt 1981b).

#### Included species and geographic distribution.

**Figure 71.**
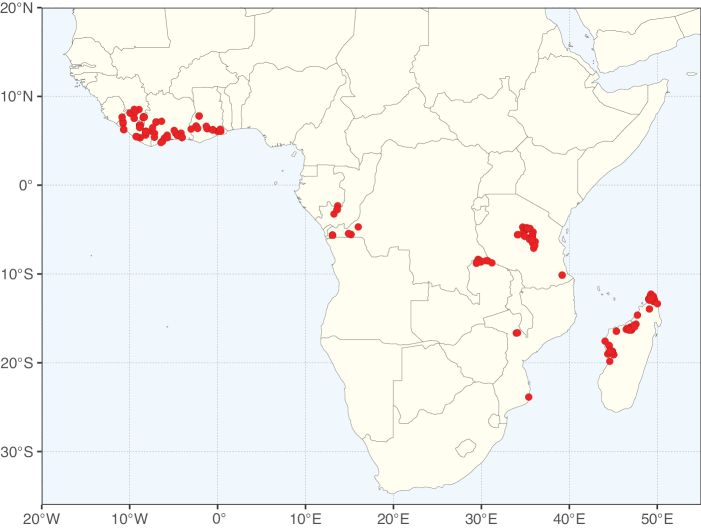
Distribution of *Bussea* based on quality-controlled digitised herbarium records. See Suppl. material 1 for the source of occurrence data.

Seven species, five from Africa (one endemic to Mozambique, one in West Africa, two in Tanzania and one in Angola – Congo), plus two species endemic to western and northern Madagascar (Fig. 71).

#### Ecology.

Seasonally dry tropical forest, thickets and deciduous bushland, often on sandy soils, moist semi-deciduous forest and rainforest.

#### Etymology.

Named in honour of Walter Carl Otto Busse (1865–1933), German botanist and ecologist who collected material of the type species in East Africa in 1901.

#### Human uses.

The wood is hard and used for construction and firewood.

#### Notes.

Robustly supported as sister to *Peltophorum* (Fig. 66) (Haston et al. 2005; Ringelberg et al. 2022), the two genera sharing distinctive peltate stigmas.

#### Taxonomic references.

Brenan (1967); Du Puy and Rabevohitra (2002) both with illustrations; see also Intkey for an illustration.

### ﻿ Peltophorum

Taxon classificationPlantaeFabalesFabaceae

(Vogel) Benth., J. Bot. (Hooker) 2(10): 75. 1840 nom. cons.

**Figure 72.**
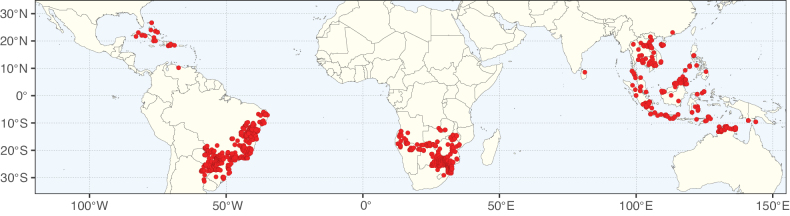
Distribution of *Peltophorum* based on quality-controlled digitised herbarium records. See Suppl. material 1 for the source of occurrence data.

Figs 67 , 68 , 70 , 72

Baryxylum Lour., Fl. Cochinch. 1: 266–267. 1790, nom rej. against Peltophorum (Vogel) Benth. Type: Baryxylumrufum Lour. [= Peltophorumdasyrhachis(Miq.)Kurzvar.dasyrhachis]
Caesalpinia sect. Peltophorum Vogel, Linnaea 11(3): 406. 1837. Type: Caesalpiniadubia Spreng. [≡ Peltophorumdubium (Spreng.) Taub.]

#### Type.

*Peltophorumvogelianum* Walpers, nom. illeg. [= *Peltophorumdubium* (Spreng.) Taub.]

#### Description.

Unarmed trees (Fig. 67D), 15–35 (60) m, and up to 80 (100) cm stem diameter, sometimes buttressed, the buttresses flat to 1 m, whole plant frequently with a rusty indumentum. **Stipules** simple, lobed or subulately branched, pinnatafid or bipinnatafid, often persistent, or small and caducous. **Leaves** bipinnate, rachis to 40 cm, 3–15 (25) pairs of pinnae, (6) 8–15 (28) pairs of sessile opposite oblong leaflets per pinna. **Inflorescences** terminal and/or axillary, simple racemes or panicles, 15–30 (40) cm long; bracts present, persistent or caducous. **Flowers** bisexual, showy; hypanthium very short, discoid or campanulate and somewhat obscure; sepals 5, free, imbricate in bud, reflexed at anthesis; petals 5, usually bright yellow, rarely pink or white, suborbicular or obovate, more or less equal, imbricate, spreading, often much crinkled, margins sometimes erose, basally clawed (Fig. 68B); stamens 10, free, filaments thickened and pilose at least towards the base, usually shorter than the petals, anthers uniform, dorsifixed, glabrous or pubescent, longitudinally dehiscent; pollen in oblate tricolporate monads with coarsely reticulate surface ornamentation with sinuous, slightly convoluted muri; ovary sessile or sub-stipitate, 3–8-ovuled, style filiform, incurved, stigma prominent peltate or capitate. **Fruits** flat, oblong or narrow ellipsoid, tapering at both ends, hard, indehiscent, or eventually splitting longitudinally, often persistent, with a firm wing-like extension on each suture, valves usually longitudinally striate, 1–6 (8)-seeded (Fig. 70C, D). **Seeds** oblong, flat or lenticular, compressed, exalbuminous.

#### Chromosome number.

2*n* = 26 (Van-Lume and Souza 2018).

#### Included species and geographic distribution.

Six (or seven) species. One (or two) in the Neotropics, one in southern Africa, four in South East Asia, one of which, *P.pterocarpum* (DC.) Backer ex K. Heyne, reaching N Australia (Fig. 72). Three species (*P.dasyrhachis*, *P.dubium* and *P.pterocarpum*) have been introduced more widely.

#### Ecology.

In lowland deciduous and evergreen moist forests, coastal vegetation along beaches and mangrove edges, frequent in forest clearings and secondary vegetation. Evergreen or often deciduous. Flowering often immediately preceding leaf flush, indehiscent narrowly-winged fruits probably wind-dispersed. Some introduced and cultivated species weakly naturalised in places, the true native distribution of *P.dubium* in some doubt in some parts of the Neotropics, e.g., in Bolivia (Barneby 1996).

#### Etymology.

From Greek, *pelte*- (= shield) and -*phoros* (= bearing), in reference to the large peltate shield-like centrally-attached stigma.

#### Human uses.

Several species are introduced and cultivated as ornamentals (Fig. 67D) and as shade trees over coffee and cacao, including Asian *P.pterocarpum* and *P.dasyrhachis* Kurz ex Baker in Africa, *P.africanum* Sond. beyond its range limits within Africa and in the New World, e.g., Florida, and *P.dubium* (Spreng.) Taub. across the Americas.

#### Notes.

The genus *Peltophorum* resembles *Schizolobium* in potentially gigantic stature, abruptly bipinnate leaves, massive terminal racemose-paniculate inflorescence of handsome yellow flowers, and almost equal calyx-lobes, but differs in the grossly peltate stigma which *Peltophorum* shares with *Bussea* which is its sister genus (Fig. 66), and its linear-ellipsoid, as opposed to oblanceolate, spoon-shaped fruits (Fig. 70A–D). Two species have been recognised in the Neotropics, but these are doubtfully distinct.

#### Taxonomic references.

Barneby (1996); Brenan (1967); Hou (1996b) including an illustration; Isely (1975); Larsen et al. (1980) including an illustration.

### ﻿ Parkinsonia

Taxon classificationPlantaeFabalesFabaceae

L., Sp. Pl. 1: 375. 1753.

**Figure 73.**
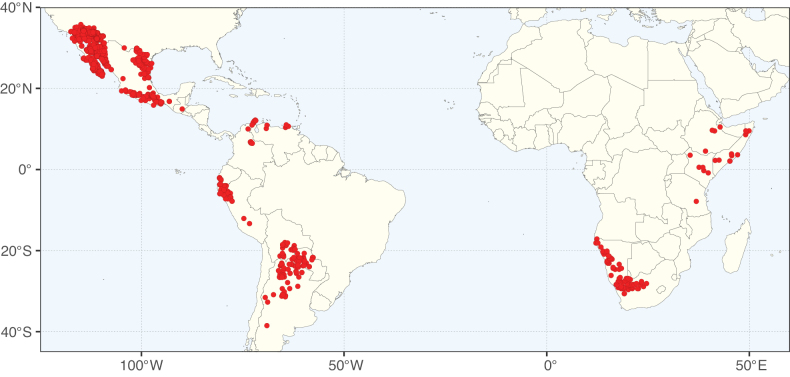
Distribution of *Parkinsonia* based on quality-controlled digitised herbarium records [excluding *P.aculeata* whose native range remains uncertain, and which is weedy and invasive in many areas of Neotropics and elsewhere – see Hawkins et al. (2007)]. See Suppl. material 1 for the source of occurrence data.

Figs 67 , 68 , 70 , 73

Cercidium Tul., Arch. Mus. Hist. Nat. 4: 133. 1844. Type: Cercidiumspinosum Tul. [= Parkinsoniapraecox (Ruiz & Pav.) Hawkins]
Rhetinophloeum H. Karst., Fl. Columb. 2: 25. 1862. Type: Rhetinophloeumviride H. Karst. [= Parkinsoniapraecox (Ruiz & Pav.) Hawkins]
Peltophoropsis Chiov., Ann. Bot. (Rome) 13(3): 385. 1915. Type: Peltophoropsisscioana Chiov. [≡ Parkinsoniascioana (Chiov.) Brenan]
Cercidiopsis Britton & Rose, N. Amer. Fl. 23(5): 306. 1930. Type: Cercidiopsismicrophylla (Torr.) Britton & Rose [≡ Parkinsoniamicrophylla Torr.]

#### Type.

*Parkinsoniaaculeata* L.

#### Description.

Highly branched shrubs or small trees (Fig. 67F, I), most species with green bark (Fig. 67H), this sometimes becoming grey-black and shallowly fissured with age; armature extremely variable among species, either unarmed, or armed with stipular spines, axillary thorns (Fig. 67G) and in some species additionally with much-reduced spinescent leaf rachides. **Stipules** either small and caducous or spinescent and persistent. **Leaves** bipinnate, but sometimes with a highly reduced spinescent rachis and flattened pinnae rachides and then superficially appearing pinnate, pinnae 1–10 pairs, the pinnular rachis winged or cylindrical; leaflets 1–80 (or more) pairs per pinna, oblong, orbicular, elliptic or ovate, oblique, rounded, equilateral or attenuate at the base, acute or mucronate at the apex, usually small and sometimes highly reduced in species with flattened photosynthetic rachides. **Inflorescences** solitary racemes in leaf axils; pedicels jointed about mid-way along length; bracts deltoid to lanceolate, rapidly deciduous. **Flowers** usually bisexual (unisexual in *P.anacantha*), showy; hypanthium shallowly campanulate, sometimes weakly oblique; sepals 5, free, reflexed, green; petals 5, these auriculate or not, the adaxial petal ovate to orbicular, lateral petals elliptic, ovate or orbicular, yellow, streaked or blotched orange in the centre (Fig. 68D, E); stamens 10, free, shorter than petals, anthers oblong or elliptic, dorsifixed, glabrous; pollen in oblate tricolporate monads with moderately reticulate surface ornamentation; ovary linear, glabrous or villous with 5–12 ovules, stigma truncate. **Fruits** plano-compressed (Fig. 70G, H) or turgid and terete (Fig. 70I), linear or oblong, straight or weakly falcate, sometimes constricted between the seeds, tardily dehiscent or indehiscent, valves generally papery or thinly coriaceous, 1–8-seeded. **Seeds** oblong to globose.

#### Chromosome number.

2*n* = 28 (Goldblatt 1981b).

#### Included species and geographic distribution.

Twelve species, eight confined to the New World (including two named hybrid species), and four in Africa. *Parkinsonia* occupies a striking disjunct amphi-Atlantic distribution across arid and semi-arid parts of the New World from the southern USA to Argentina and disjunctly in arid parts of north-eastern and south-western Africa (Fig. 73). One species, *P.aculeata* (not mapped here) is pantropically cultivated, naturalised and in places invasive (e.g., in northern Australia) and of uncertain regional nativity across its wide New World distribution (Hawkins et al. 2007).

#### Ecology.

*Parkinsonia* is confined to seasonally dry, semi-arid and arid climates, growing in Chaco woodlands, seasonally dry tropical forests, deserts and semi-deserts, occupying most enclaves of the disjunct trans-continental succulent biome distribution (sensu Schrire et al. 2005a; Ringelberg et al. 2020), except south-western Madagascar and the Caatinga in north-eastern Brazil. The true native range of *P.aculeata* is uncertain but is certainly confined to the New World and hypothesised to be primarily in seasonally inundated, and sometimes saline former lake-bed and river flood-plain habitats often on deeply cracking black vertisols (Hawkins et al. 2007), i.e., very different habitats from the remaining species.

#### Etymology.

Named in honour of John Parkinson (1567–1650), the British apothecary and herbalist to King James I of England.

#### Human uses.

*Parkinsoniaaculeata* is widely cultivated as an ornamental street tree in arid zones.

#### Notes.

*Parkinsonia* is robustly supported as sister to the clade comprising *Heteroflorum* + *Conzattia* + *Delonix* + *Colvillea* (Fig. 66) (Ringelberg et al. 2022). Until recently some species were referred to the genus *Cercidium*, but it is now clear that *Cercidium* is phylogenetically nested within *Parkinsonia* (Haston et al. 2005) and new name combinations for all species are now available in *Parkinsonia* (Romão and Mansano 2021). In the New World, *Parkinsonia* species are frequently referred to as Palo Verdes, because of their characteristic green bark.

#### Taxonomic references.

Brenan (1980) with illustration; Carter (1974); Hawkins et al. (2007); Karsten (1862) with illustration; Romão and Mansano (2021) with illustrations.

### ﻿ Conzattia

Taxon classificationPlantaeFabalesFabaceae

Rose, Contrib. U.S. Natl. Herb. 12(9): 407. 1909.

**Figure 74.**
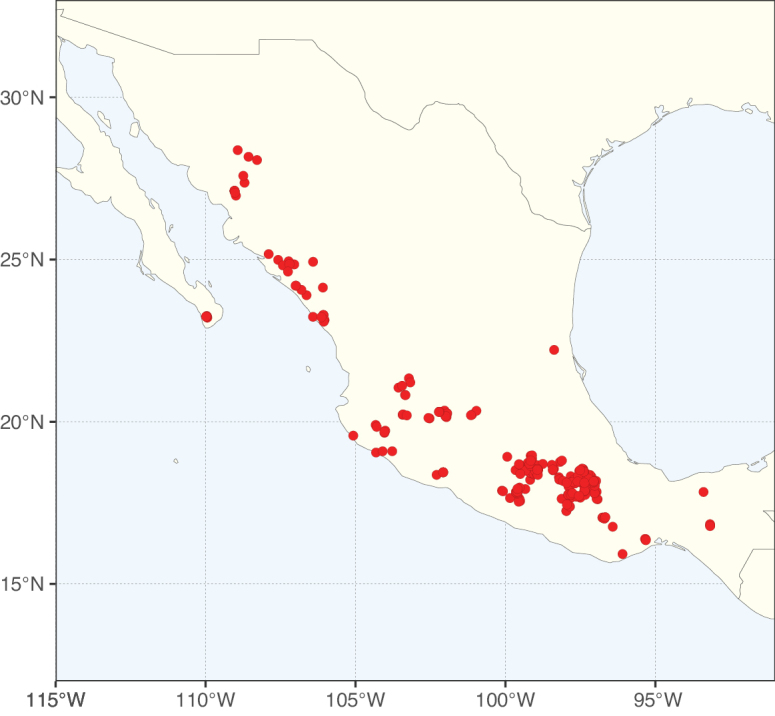
Distribution of *Conzattia* based on quality-controlled digitised herbarium records. See Suppl. material 1 for the source of occurrence data.

Figs 67 , 68 , 70 , 74

#### Type.

*Conzattiaarborea* Rose [= *Conzattiamultiflora* (B.L. Rob.) Standl.]

#### Description.

Unarmed, small to medium-sized tree, 3–10 (15) m, generally forking low down with a large spreading flat-topped ‘umbrella’ crown (Fig. 67L), the trunk 20–50 (75) cm in diameter, the bark thin, pale silver-grey (Fig. 67M), inner bark green, whole plant largely glabrous. **Stipules** minute. **Leaves** bipinnate, with 10–15 pairs of pinnae, 9–20 pairs of leaflets per pinna, leaflets oblong, apex acute, oblique at base. **Inflorescences** slender, erect, 6–12 (25) cm long axillary racemes clustered near branch tips, pedicels jointed just below the flower; bracts caducous. **Flowers** unisexual by reduction of the androecium or gynoecium, usually dioecious but some individuals carry both functionally male and female flowers; hypanthium shallowly campanulate; sepals 5, free, imbricate only in bud, strongly reflexed, subequal; petals 5, yellow, more or less equal (Fig. 68F); stamens 10, free, slightly shorter than petals, anthers dorsifixed, glabrous; pollen in oblate tricolporate monads with moderately reticulate surface ornamentation; ovary densely pubescent, stipitate, 6-ovuled (ovaries of functionally male flowers smaller, not fully developed), style filiform, usually straight in functionally female flowers, swan-necked in functionally male flowers, stigma ciliate. **Fruits** plano-compressed, glabrous, the margins narrowly winged, acuminate at apex, dehiscent along both sutures, (2) 3 (4)-seeded (Fig. 70J). **Seeds** oblong, 10–12 mm long, brown, albuminous.

#### Chromosome number.

Unknown.

#### Included species and geographic distribution.

Monospecific, endemic to seasonally dry western and southern Mexico from Sonora and Baja California Sur south to Chiapas (Fig. 74).

#### Ecology.

Confined to seasonally dry tropical forest. Strongly deciduous, fruits ripening when leafless (Fig. 67L), and flowering preceding leaf flush.

#### Etymology.

Named in honour of the Italian-born Mexican botanist Cassiano Conzatti (1862–1951), a prodigious plant collector in Mexico and Chile and director of the Escuela Normal in Oaxaca, Mexico.

#### Human uses.

*Conzattia* has been listed as an important source of medicine and wood for construction and fuel, and immature fruits are used as a minor food in Mexico (Arellano 1987). The presence of seeds of *Conzattiamultiflora* at several archaeological sites in south-central Mexico and the frequent occurrence of trees around pre-Colombian temple sites suggest use as a minor food spanning several millennia (Zárate 2000).

#### Notes.

*Conzattia* is robustly supported as sister to *Heteroflorum* (Fig. 66; Haston et al. 2005; Ringelberg et al. 2022), both genera endemic to Mexico and sharing seasonally dry tropical ecology, and both with similar low, flat-topped tree crowns (Fig. 67J, L), smooth grey bark (Fig. 67K, M), yellow flowers and frequently at least partial dioecy, but readily distinguished by their radically different fruits (Fig. 70J–M). Three species of *Conzattia* have been described based on minor differences in leaf indumentum, quantitative variation in leaf formula and stipules, none of which are fixed or correlated with geography, prompting recognition of a single somewhat variable species *C.multiflora* (Haston 2003).

#### Taxonomic references.

Haston (2003); Lewis (2005b) with illustration; Standley (1922).

### ﻿ Heteroflorum

Taxon classificationPlantaeFabalesFabaceae

M. Sousa, Novon 15: 213. 2005.

Figs 67 , 70 , 75

#### Type.

*Heteroflorumsclerocarpum* M. Sousa

#### Description.

Unarmed small trees to 15 m, forked near the base, forming a wide spreading umbrella crown (Fig. 67J), the trunk with horizontal lines of conical corky protuberances, these not or barely spinescent, otherwise bark smooth pale to mid metallic grey (Fig. 67K). **Stipules** acicular to setiform, caducous. **Leaves** bipinnate, petiole eglandular, canaliculate, sulcate with (2) 4–7 pairs of pinnae, 10–14 pairs of alternate leaflets per pinna, these elliptic-oblong. **Inflorescences** more or less erect axillary racemes, flowers subtended by jointed pedicels, bracts persistent. **Flowers** unisexual by reduction of the androecium or gynoecium, dioecious, a well-developed hypanthium at the base; sepals 5, free; petals 5, homomorphic elliptic to obovate, yellow, lacking a claw; stamens 10, free, exserted beyond the corolla, inserted at the apex of the hypanthium; pollen not studied; ovary sessile, 9–13 ovules, style tubular, widened at apex, stigma circular, broad, flat or slightly concave. **Fruits** indehiscent, cylindrical, hard, weakly woody, the mesocarp thickened and fibrous, the valves smooth, glabrous, pale orange-brown when ripe (Fig. 70K, M), with fibrous-spongy septae between the seeds forming marked seed cavities (Fig. 70L). **Seeds** discoid to subspherical, integument hard, hilum apical.

#### Chromosome number.

Unknown.

#### Included species and geographic distribution.

**Figure 75.**
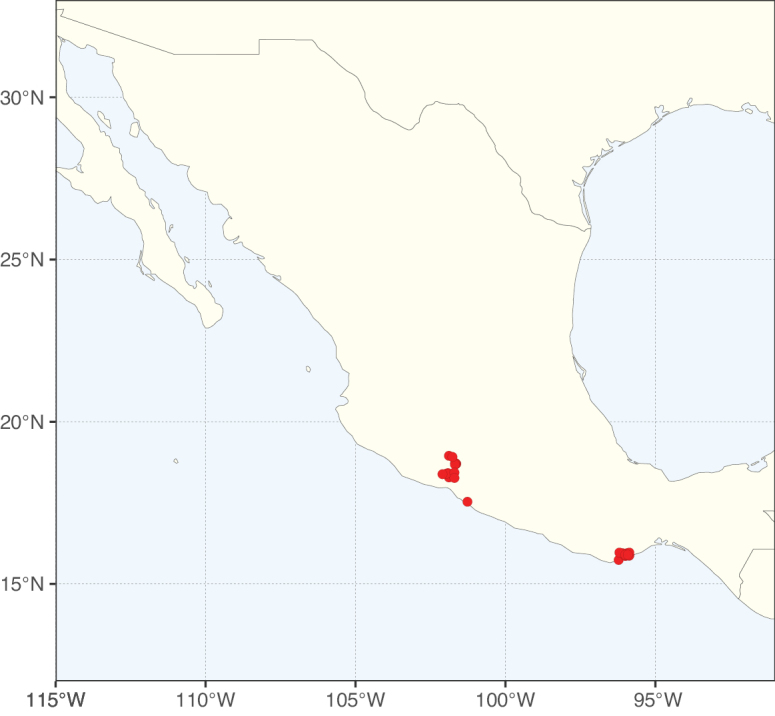
Distribution of *Heteroflorum* based on quality-controlled digitised herbarium records. See Suppl. material 1 for the source of occurrence data.

Monospecific. A narrowly restricted endemic of southern Mexico, disjunctly distributed close to the Pacific coast in the lower reaches of the Río Balsas watershed around El Infiernillo (Michoacán and Guerrero) and in coastal Oaxaca (Fig. 75). *Heteroflorumsclerocarpum* and hence the genus as a whole, is categorised as Endangered (Machuca-Machuca et al. 2021).

#### Ecology.

Confined to seasonally dry tropical forest, occasionally forming dominant stands (Urrea-Galeano et al. 2018). Trees are strongly deciduous, flowering when leafless or as new leaves flush. Fruits indehiscent and reported to be consumed by terrestrial mammals facilitating release of the seeds and accelerating germination (Urrea-Galeano et al. 2018), and potentially endozoochorous seed dispersal. Flowers pollinated by large *Xylocopa* bees.

#### Etymology.

The name *Heteroflorum* is derived from *hetero*- (Greek = different, uneven) and -*florum* (plural of *flos*; Latin = flower) in reference to the different sized functionally male and female flower forms associated with dioecy.

#### Human uses.

Unknown.

#### Notes.

*Heteroflorum* is robustly supported as sister to a second monospecific Mexican endemic genus, *Conzattia* (Fig. 66; Haston et al. 2005; Ringelberg et al. 2022). These two genera share similar tree growth habit and bark (Fig. 67J–M), and flowers, but have completely different fruits (Fig. 70K–M).

#### Taxonomic references.

Sousa (2005) with illustration.

### ﻿ Colvillea

Taxon classificationPlantaeFabalesFabaceae

Bojer ex Hook., Bot. Mag. 61: t.3325 & t.3326. 1834.

**Figure 76.**
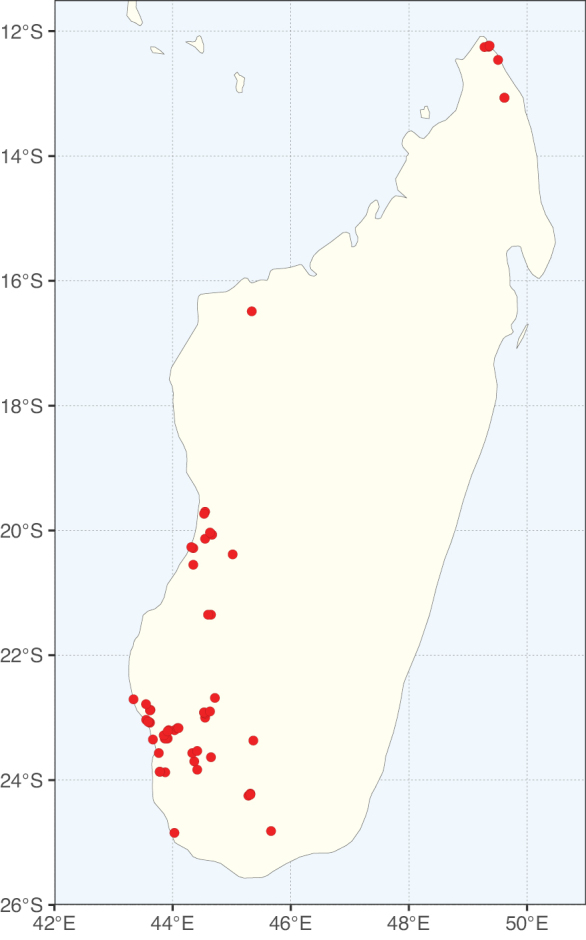
Distribution of *Colvillea* based on quality-controlled digitised herbarium records. See Suppl. material 1 for the source of occurrence data.

Figs 67 , 68 , 70 , 76

#### Type.

*Colvillearacemosa* Bojer ex Hook.

#### Description.

Unarmed, small to medium-sized trees, 8–15 (20) m and 50–70 (90) cm stem diameter (Fig. 67N); bark thin, pale grey, inner bark green. **Stipules** minute, setaceous and caducous. **Leaves** bipinnate, petiole glands absent, (6) 9–16 pairs of pinnae with (12) 15–30 pairs of oblong leaflets per pinna, apex rounded, base asymmetrical. **Inflorescences** suberect, large, robust, many-flowered terminal panicles, the secondary axes pendulous, pedicels jointed, bracts mostly caducous. **Flowers** bisexual, very distinctive, strongly zygomorphic and inverted (resupinate), bright orange (Fig. 68G); calyx of 5 subequal valvate segments, which are leathery and thickened, especially towards the tips, 4 of the segments fused for most of their length like a spadix; corolla with 5 reduced petals, the median petal (held lowermost in the flower) enclosed within the sepal cup, the lamina rolled into a nectar-containing conical receptacle; stamens 10, free, longer than petals, clustered curving out from the upper side of the inverted flower, anthers dorsifixed, glabrous; pollen in oblate tricolporate monads with coarsely reticulate surface ornamentation with sinuous, slightly convoluted muri; ovary short stipitate, flat, exserted beyond stamens. **Fruits** large, linear-oblong, pendulous, the valves partially separating allowing the seeds to be shaken out, the valves relatively thin-textured, strongly flattened and barely lignified (Fig. 70P). **Seeds** oblong-ovate, flattened.

#### Chromosome number.

2*n* = 28 (Goldblatt 1981b).

#### Included species and geographic distribution.

Monospecific, endemic to western Madagascar (Fig. 76). Cultivated elsewhere, including in southern Africa.

#### Ecology.

Seasonally dry forests and spiny thickets. Trees are deciduous. The flowers are nectariferous and although often eaten by lemurs, they are pollinated by Siouimanga sunbirds (Du Puy and Rabevohitra 2002).

#### Etymology.

Named in honour of Sir Charles Colville (1770–1843), distinguished Scottish officer under Wellington in the Napoleonic wars and governor of Mauritius, 1828–1834.

#### Human uses.

Sometimes planted as an ornamental in villages (Fig. 67N).

#### Notes.

The status of *Colvillea* as a genus distinct from *Delonix* remains questionable as its placement either as sister to *Delonix*, or nested within it is unstable in phylogenies that have densely sampled *Delonix* (Haston et al. 2005; Babineau and Bruneau 2017), albeit in recent analyses based on larger gene sets *Colvillea* is clearly resolved as sister to *Delonix* (Kates et al. 2024). In contrast to *Delonix*, *Colvillea* has large terminal panicles of resupinate flowers with orange petals, four fused calyx segments and clustered stamens.

#### Taxonomic references.

Bojer (1834); Du Puy and Rabevohitra (2002) both with illustrations.

### ﻿ Delonix

Taxon classificationPlantaeFabalesFabaceae

Raf., Fl. Tellur. 2: 92. 1837.

**Figure 77.**
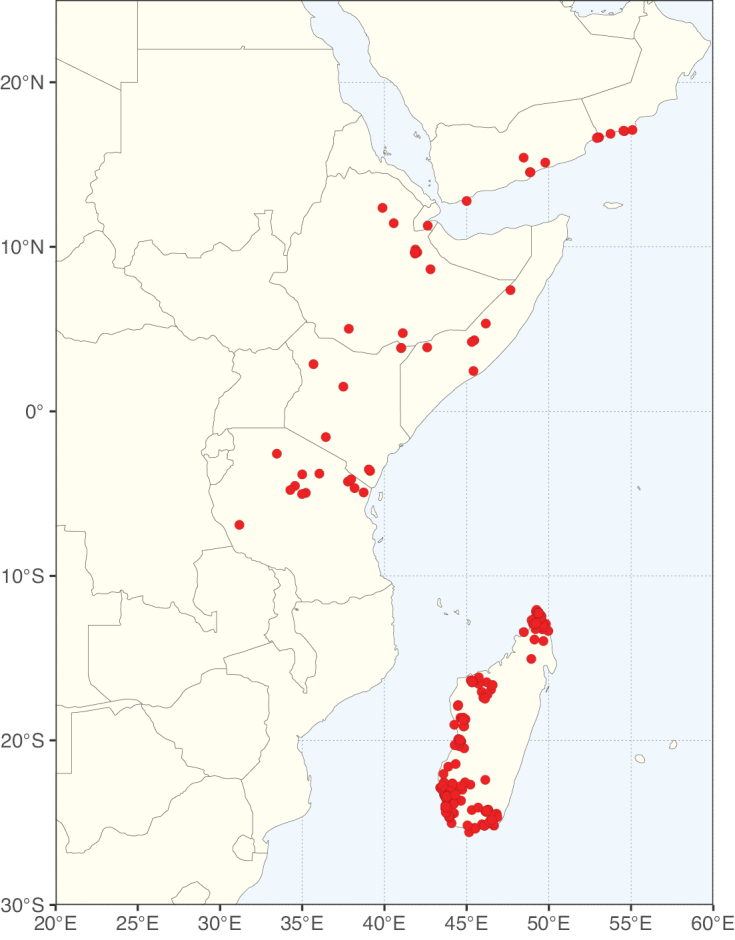
Distribution of *Delonix* based on quality-controlled digitised herbarium records. See Suppl. material 1 for the source of occurrence data.

Figs 67 , 68 , 70 , 77

Aprevalia Baill., Bull. Mens. Soc. Linn. Paris 1: 428. 1884. Type: Aprevaliafloribunda Baill. [≡ Delonixfloribunda (Baill.) Capuron]
Lemuropisum H. Perrier, Bull. Soc. Bot. France 85: 494. 1939. Type: Lemuropisumedule H. Perrier [≡ Delonixedulis (H. Perrier) Babineau & Bruneau]
Delonix sect. Aprevalia (Baill.) Capuron, Adansonia, n.s. 8(1): 12. 1968. Type: Delonixfloribunda (Baill.) Capuron [≡ Aprevaliafloribunda Baill.]

#### Type.

*Delonixregia* (Bojer ex Hook.) Raf. [≡ *Poincianaregia* Bojer ex Hook.]

#### Description.

Shrubs (*D.edule*) or small to medium-sized trees to 30 m, often forking low down and forming spreading, flat-topped umbrella crowns (Fig. 67P, Q), or in a subset of species, the trunk fusiform with distinctive ‘bottle tree’ growth form constricted at the base, swollen above and only tapering immediately below the rounded crown, reminiscent of baobabs (Fig. 67O); generally unarmed, but in *D.edule* armed with spinescent shoots. **Stipules** generally minute or absent, but pinnate or bipinnate in *D.regia*. **Leaves** generally bipinnate, 2–13 pairs of pinnae, and (6) 12–20 (40) pairs of leaflets per pinna, much reduced and simply paripinnate in *D.edule* with just 2–3 pairs of small leaflets. **Inflorescences** axillary racemes usually produced at several consecutive nodes and flowers generally held above the foliage (Fig. 68Q), pedicels jointed, bracts caducous (or often persistent in *D.regia*). **Flowers** bisexual, usually large and showy (Figs 67Q, 68H, I); hypanthium short, turbinate; sepals 5, free, subequal, valvate, strongly reflexed, leathery and thickened especially towards the tips; petals usually 5, these spreading, round or ovate with a long claw, the margins crisped and frilled when fully expanded, somewhat erose or deeply lacerate, the median petal usually larger, differently coloured, and held in front of the other petals, its claw with inrolled margins, forming a narrow funnel containing nectar, petals white, pale pink or bright scarlet (*D.regia*), the upper petal with a central yellow and white blotch (Fig. 68I), and the whole petal suffusing red with age or post pollination (Fig. 68H), but in two species the petals much reduced and in *D.floribunda* (Baill.) Capuron the lateral petals absent and only a single erect, narrow yellow petal is present; stamens 10, free, equal, longer than petals, spreading, not clustered around the ovary, anthers dorsifixed, glabrous; pollen in oblate tricolporate monads with coarsely reticulate surface ornamentation with sinuous, slightly convoluted muri; ovary stipitate, style filiform, stigma small and glabrous or ciliate. **Fruits** usually large, many-seeded, linear-oblong, somewhat flattened, woody, pendulous or suberect, either long, straight or weakly falcate and strap-like (Fig. 70O) or shorter and tightly curved (Fig. 70N), tardily dehiscent until after they have fallen, eventually splitting into 2 flat (non-twisting) valves, or (in non-Madagascan species) the valves thinner, coriaceous and less robust, and (in *D.edule*) lacking separate seed chambers, the valves thinner, coriaceous, twisting at dehiscence. **Seeds** ellipsoidal or subspherical to ovoid, pale brown, finely mottled, in individual chambers or, in *D.edule*, oblong, truncate, the testa cream-white and leathery.

#### Chromosome number.

2*n* = 26 or 28 (Goldblatt 1981b; Van-Lume and Souza 2018).

#### Included species and geographic distribution.

Twelve species, ten endemic to south-western, western and northern Madagascar, one widespread in eastern and north-eastern Africa extending to adjacent Arabia, and one restricted to northern Kenya and Somalia (Fig. 77).

#### Ecology.

Seasonally dry and arid thickets including the spiny dry forests of south-western Madagascar, *Acacia*-*Commiphora* woodland in north-eastern Africa, and semi-desert scrub. A few species have distinctive swollen trunks and ‘bottle tree’ habits (Fig. 67O). Deciduous, or largely so, often flowering when leafless or as new flush leaves emerge. Pollination is thought to be by moths in the white-flowered species and by Souimanga sunbirds (*Nectarinia*) in the yellow and red-flowered species.

#### Etymology.

From Greek, *delo*- (= evident, conspicuous) and -*onyx* (= claw or nail) in reference to the fact that the petals have very long claws.

#### Human uses.

The well-known Flamboyant or Flame tree, *Delonixregia* with its magnificent showy racemes of scarlet flowers is a favourite in cultivation as an ornamental street and garden tree throughout the tropics (Lewis 2020a). Less well-known is that the seeds of *D.edule* (formerly *Lemuropisumedule* H. Perrier) are eaten when young and have potential as a food crop (Du Puy and Rabevohitra 2002).

#### Notes.

The genus *Delonix* is sister to the morphologically similar *Colvillea*. These two genera together are robustly supported as sister to the clade comprising two monospecific endemic Mexican genera *Conzattia* and *Heteroflorum* (Fig. 66; Ringelberg et al. 2022), providing a striking example of trans-continental phylogenetic biome conservatism to seasonally dry and arid tropical climates (Ringelberg et al. 2020). The monospecific genus *Lemuropisum* was found to be nested within *Delonix* and sunk within that genus by Babineau and Bruneau (2017), adding further to the already considerable heterogeneity of flower and fruit morphology in the genus. The inflorescences of *Delonix* although superficially resembling those of *Peltophorum*, *Bussea* and *Colvillea* with the flowers generally held above the foliage, never form a true terminal panicle, but are rather axillary racemes usually produced at several consecutive nodes.

#### Taxonomic references.

Babineau and Bruneau (2017); Brenan (1967), with illustration; Du Puy and Rabevohitra (2002), with illustration; Du Puy et al. (1995), with illustration; Lewis (2020a), with illustration.

## ﻿﻿8. Tribe Sclerolobieae

Haroldo Cavalcante de Lima^8,27^, Isau Huamantupa-Chuquimaco^23^, Domingos B. O. S. Cardoso^8,9^

Citation: Lima HC, Huamantupa-Chuquimaco I, Cardoso DBOS (2024) 8. Tribe Sclerolobieae. In: Bruneau A, Queiroz LP, Ringelberg JJ (Eds) Advances in Legume Systematics 14. Classification of Caesalpinioideae. Part 2: Higher-level classification. PhytoKeys 240: 165–176. https://doi.org/10.3897/phytokeys.240.101716

### ﻿Tribe Sclerolobieae

Taxon classificationPlantaeFabalesFabaceae

Benth. & Hook. f., Gen. Pl. 1: 436. 1865.

Figs 78 , 79 , 80 , 81 , 82 , 83 , 84 , 85 , 86

Tachigalieae Nakai, Chosakuronbun Mokuroku [Ord. Fam. Tr. Nov.]: 252. 1943. Type: Tachigali Aubl.

#### Type.

*Sclerolobium* Vogel [= *Tachigali* Aubl.]

#### Included genera

**(5).***Arapatiella* Rizzini & A. Mattos (2 species), *Diptychandra* Tul. (2), *Jacqueshuberia* Ducke (7), *Moldenhawera* Schrad. (12), *Tachigali* Aubl. (80–90).

#### Description.

Trees or shrubs, unarmed. **Stipules** present, persistent or caducous. **Leaves** pinnate or bipinnate, usually paripinnate, rarely imparipinnate; myrmecophilous domatia present or absent; leaflets opposite; extrafloral nectaries absent. **Inflorescences** terminal or lateral racemes or panicles. **Flowers** bisexual, bilaterally or radially symmetrical; hypanthium cupular or cylindrical; sepals 5, free or rarely connate at base; petals 5, free, equal or the dorsal, standard petal differentiated; stamens often 10 (18), rarely only one stamen and 7 or 9 staminodes, free or connate, monomorphic or dimorphic, anthers longitudinally dehiscent, rarely opening through pores; pollen in monads, rarely in tetrads or associated with viscin threads, the exine scabrate-punctate to reticulate; ovary sessile or stipitate; nectary disk absent. **Fruit** dehiscent along both sutures or indehiscent with flaking exocarp. **Seeds** ellipsoid to obovoid, laterally compressed or complanate, sometimes winged, seed coat hard or soft, endosperm scarce.

#### Distribution.

Mostly Neotropical, extending from Central to South America, in tropical rainforests, tropical cloud forests, seasonally dry forests, and fire-prone savannas.

#### Clade-based definition.

**Figure 78.**
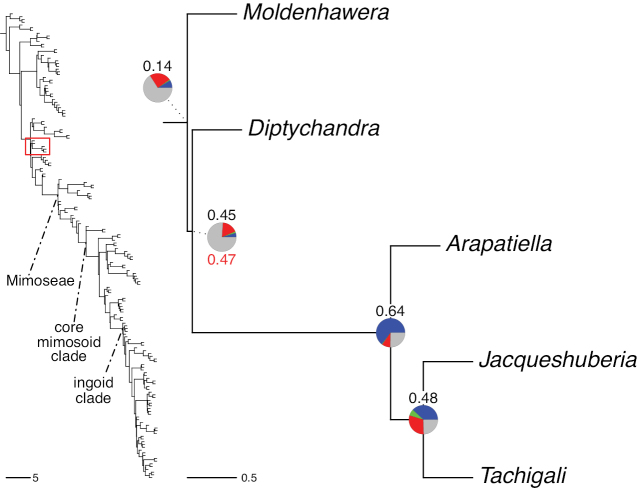
Generic relationships in tribe Sclerolobieae. For description of phylogeny and support values, see Fig. 6 caption (page 63).

The most inclusive crown clade containing *Moldenhawerafloribunda* Schrad. and *Tachigaliguianensis* (Benth.) Zarucchi & Herend., but not *Caesalpiniabrasiliensis* L., *Dimorphandraconjugata* (Splitg.) Sandwith or *Mimosasensitiva* L. (Fig. 78).

#### Notes.

Although the generic name *Sclerolobium* is currently treated under *Tachigali*, the tribe name Sclerolobieae (Bentham 1865) has priority over Tachigalieae (Nakai 1943). Tribe Sclerolobieae, as herein circumscribed based on phylogenomic analyses (Ringelberg et al. 2022), includes five genera (Fig. 78), most of which were never closely associated with each other in traditional classifications of Caesalpinioideae (Polhill and Vidal 1981; Polhill 1994; Lewis 2005b; Ulibarri 2008). For example, Polhill and Vidal (1981) treated *Tachigali* and *Diptychandra* as part of the Sclerolobium group of the old sense tribe Caesalpinieae, whereas *Arapatiella*, *Jacqueshuberia*, and *Moldenhawera* were classified together with ten other Caesalpinioideae genera in the Peltophorum group of the same tribe.

**Figure 79.**
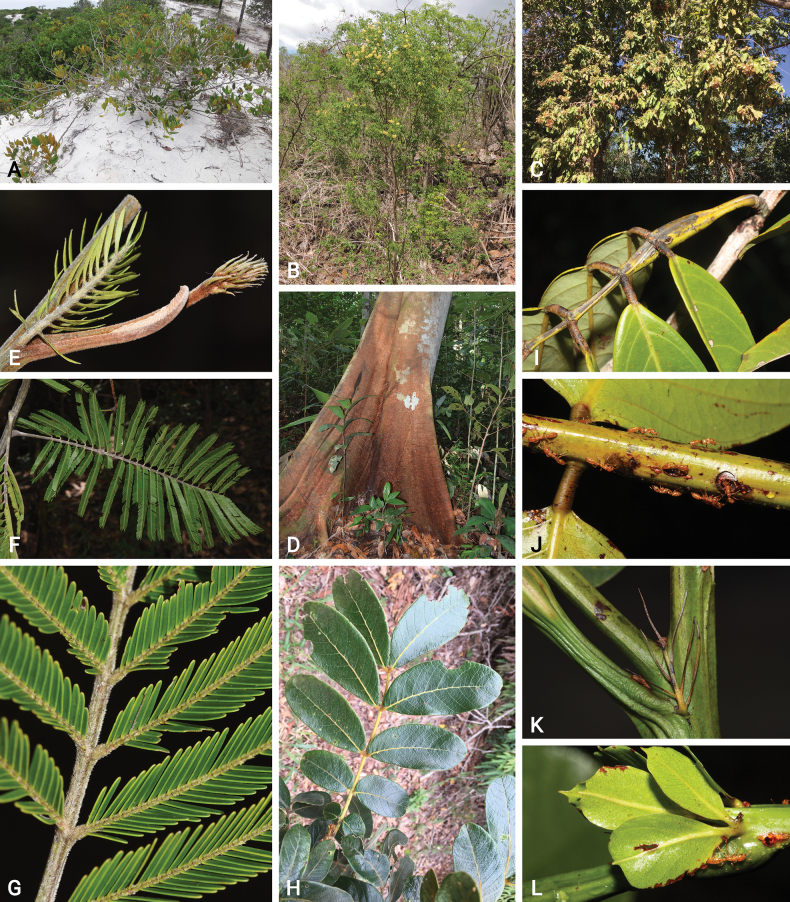
Vegetative morphology of Sclerolobieae**A***Moldenhaweranutans* L.P. Queiroz, G.P. Lewis & Allkin shrubby individual in a Restinga sand dune field **B***Diptychandraaurantiaca* (Mart.) Tul. tree in a Caatinga seasonally dry setting **C***Arapatiellapsilophylla* (Harms) R.S. Cowan flowering tree **D***Tachigaliamplifolia* (Ducke) Barneby large, buttressed tree **E***Jacqueshuberiapurpurea* Ducke pinnate stipule **F** leaf **G** detail of a leaf **H***Tachigalirugosa* (Mart. ex Benth.) Zarucchi & Pipoly leaf with characteristic inversely symmetrical leaflets **I, J***Tachigali* sp. details of leaf domatia **K, L** variation in stipule shape. Photo credits **A, C, E–L** D Cardoso **B** RT Queiroz https://rubens-plantasdobrasil.blogspot.com/**D** I Huamantupa.

**Figure 80.**
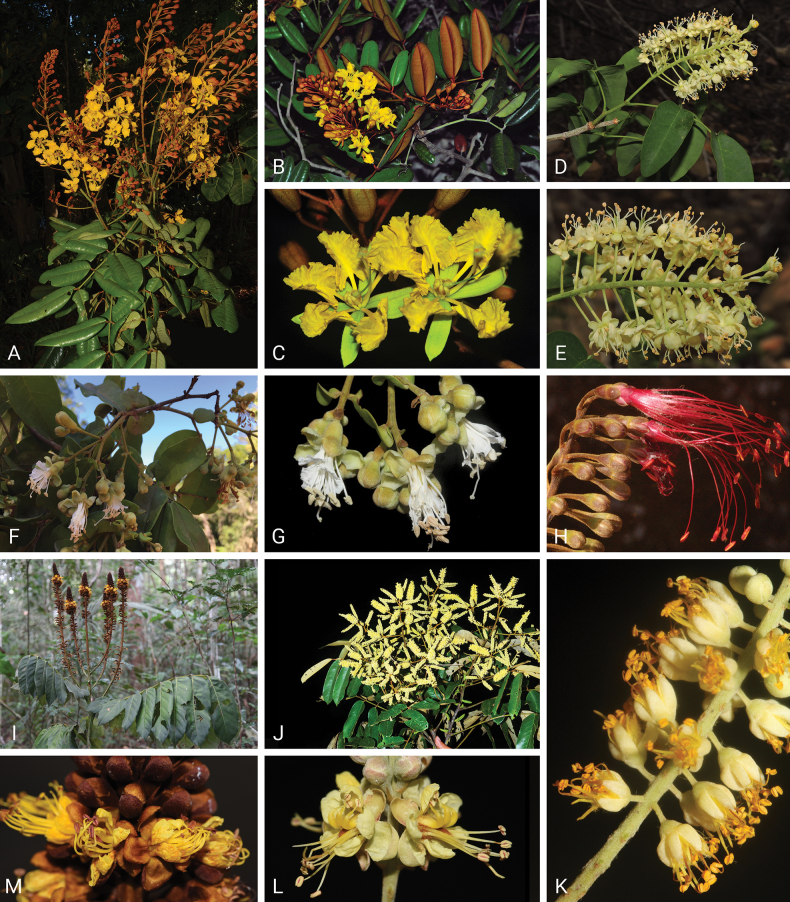
Floral morphology of Sclerolobieae**A***Moldenhaweralushnathiana* Yakovlev highly branched inflorescence **B***Moldenhaweranutans* L.P. Queiroz, G.P. Lewis & Allkin flowering branch, showing the typical navicular leaves with rust-coloured leaflet abaxial surface **C** flowers **D***Diptychandraaurantiaca* (Mart.) Tul. flowering branch **E** inflorescence **F***Arapatiellapsilophylla* (Harms) R.S. Cowan inflorescences **G** flowers **H***Jacqueshuberiapurpurea* Ducke flower **I***Tachigalimacrostachya* Huber candelabrum-like inflorescences **J***Tachigaliamarumayu* Huamantupa, H.C. Lima & D.B.O.S. Cardoso densely paniculate inflorescences **K***Tachigaliparaensis* (Huber) Barneby radially symmetrical flowers **L***Tachigalipaniculata* Aubl. bilaterally symmetrical flowers **M***Tachigalimacrostachya* bilaterally symmetrical flowers. Photo credits **A** LP Queiroz **B, C, F, G, I, K–M** D Cardoso **J** I Huamantupa **D, E** RT Queiroz https://rubens-plantasdobrasil.blogspot.com/**H** M Cohn-Haft.

**Figure 81.**
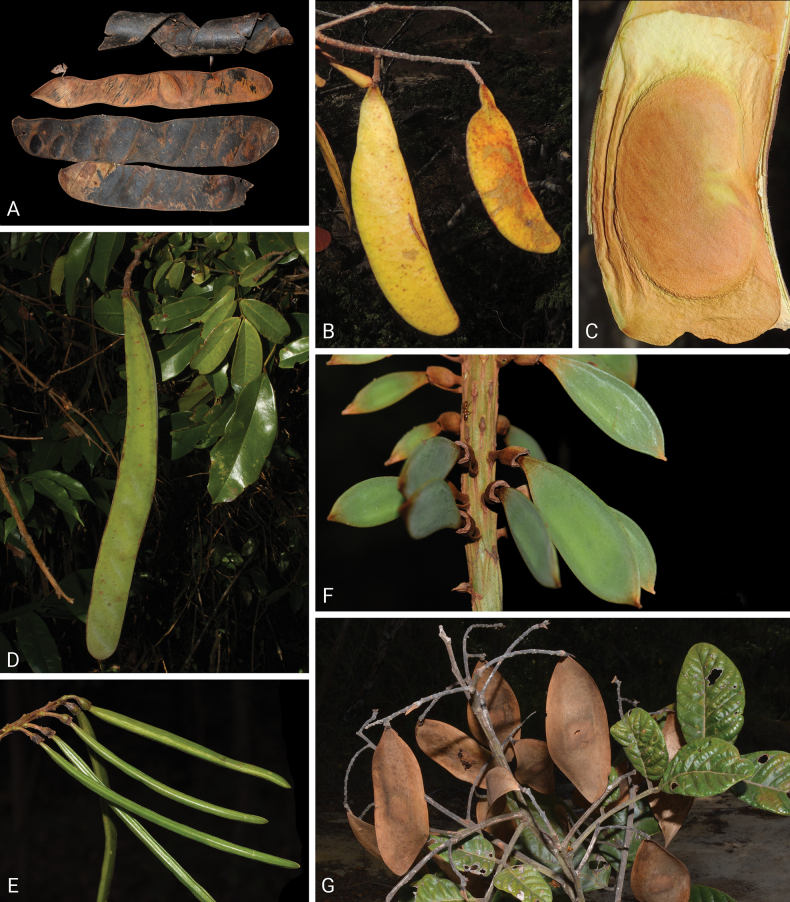
Fruit morphology of Sclerolobieae**A***Moldenhawerapolysperma* (Vell.) Stellfeld old fruit valves **B***Diptychandraaurantiaca* (Mart.) Tul. fruits **C** fruit opened showing the characteristic winged seed **D***Arapatiellapsilophylla* (Harms) R.S. Cowan immature fruit **E***Jacqueshuberiapurpurea* Ducke immature fruits **F***Tachigalimacrostachya* Huber immature fruits **G***Tachigalirugosa* (Mart. ex Benth.) Zarucchi & Pipoly mature fruits. Photo credits **A** C Vivas **B, C** RT Queiroz https://rubens-plantasdobrasil.blogspot.com/**D–F** D Cardoso **G** LP Queiroz.

Not only have the relationships amongst Sclerolobieae genera now been strongly resolved for almost all nodes (Fig. 78), but also their monophyly is strongly supported by morphological characters and more densely sampled phylogenies based on a few plastid and nuclear loci (Haston et al. 2003, 2005; Bruneau et al. 2008; Chomicki et al. 2015; Huamantupa-Chuquimaco 2020). The tribe is not defined by obvious synapomorphies, although there is a tendency for the occurrence of unusually divided or foliaceous stipules (except for *Diptychandra*). The genera are distinct in leaf morphology (pinnate or bipinnate leaves) (Fig. 79), floral symmetry (radial or bilateral), pollination syndrome (bees or birds), pollen presentation (monads or tetrads), fruit morphology, seed morphology (winged or non-winged), and dispersal syndrome (autochory, hydrochory, or anemochory). The high morphological variation found within Sclerolobieae in terms of floral architecture (Fig. 80) and fruit type (Fig. 81) may explain why its constituent genera have long remained unnoticed as belonging to the same natural group. For example, *Tachigali* and *Diptychandra* have similarly small flowers, which are mostly radially symmetrical, but ontogenetically the apparently morphologically homogeneous flowers of *Tachigali* display considerable variation (Casanova et al. 2020) and are quite different from those of *Diptychandra*. Many *Tachigali* species also possess bilaterally symmetrical flowers, with a curved hypanthium similar to the much bigger flowers of *Arapatiella* and *Jacqueshuberia*. The *Moldenhawera* flowers are striking in their more or less radially symmetrical Malpighiaceae-like architecture, where the often-yellow petals are marginally crimped and long-clawed, and the cluster of staminodes are much smaller than the single bearded fertile stamen and resemble the oil glands (elaiophores) typical of the Malpighiaceae flowers (Queiroz et al. 2023). The fruits are wind-dispersed, compressed cryptosamaras in *Tachigali*, whereas all other genera in the tribe have elastically dehiscent, laterally compressed pods. Within Sclerolobieae there is also a high degree of variation in pollen (Graham and Barker 1981; Banks et al. 2003; Banks and Lewis 2018), which can be colporate, in monads or tetrads, and with scabrate-punctate to reticulate ornamentation; pollen connecting viscin threads are present in *Arapatiella* and *Jacqueshuberia*. Faria et al. (2022) report nodulation with a fixation thread type of nodule anatomy in at least one species each of *Moldenhawera*, *Tachigali*, *Jacqueshuberia*, and a confirmed absence of nodulation in *Arapatiella* (unknown in *Diptychandra*).

### ﻿ Moldenhawera

Taxon classificationPlantaeFabalesFabaceae

Schrad., Gött. Gel. Anz. 1: 718. 1821.

**Figure 82.**
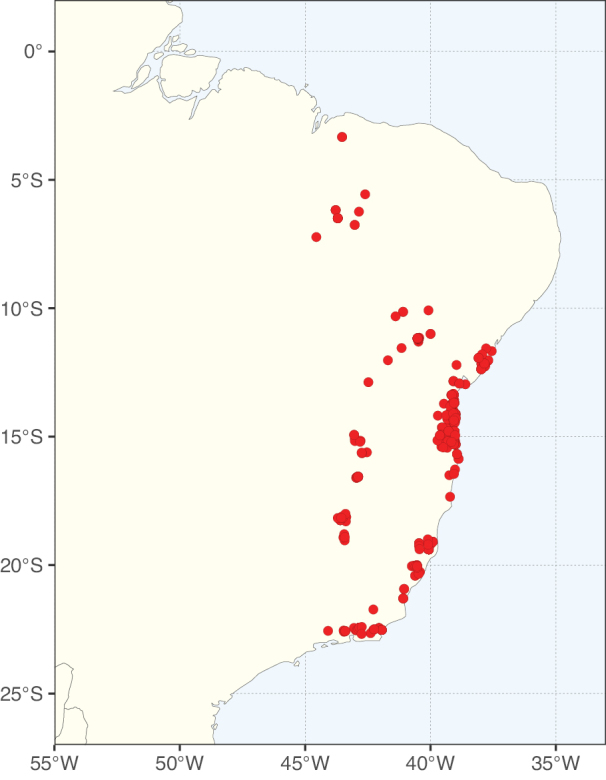
Distribution of *Moldenhawera* based on quality-controlled digitised herbarium records. See Suppl. material 1 for the source of occurrence data.

Figs 79 , 80 , 81 , 82

Dolichonemia Nees, Flora 4 (19): 303. 1821. Type: Dolichonemiaspeciosa Nees [= Moldenhawerafloribunda Schrad.]

#### Type.

*Moldenhawerafloribunda* Schrad.

#### Description.

Trees or shrubs, the young branches often rust-coloured with T-shaped trichomes. **Stipules** often pinnate. **Leaves** paripinnate; bipinnate, partially bipinnate; leaflets opposite. **Inflorescences** in terminal corymbiform racemes, fasciculate or solitary; bracts and bracteoles setaceous, caducous. **Flowers** apparently radially symmetrical, but essentially bilaterally symmetrical because of the single large fertile stamen; hypanthium infilled; sepals (4) 5, free; petals (4) 5, yellow, rarely pink (*M.acuminata* Afr. Fern. & P. Bezerra), clawed, the margins crimped; androecium dimorphic, fertile stamen 1, filament elongated, connective hairy, anther dehiscing through longitudinal slits, staminodes 7 or 9, filaments short or elongated, anthers dehiscing by longitudinal slits or pores, or indehiscent; pollen grains in monads, perforate to an almost vermiculate-reticulate tectum, scabrate-punctate; ovary sessile, stigma truncate. **Fruit** a dehiscent, linear, compressed legume, the two valves woody and coiling at dehiscence, 4–8-seeded. **Seeds** ovate to oblong, compressed.

#### Chromosome number.

Unknown.

#### Included species and geographic distribution.

Twelve species and one variety, exclusively in Brazil, mostly along the eastern Atlantic coast (Bahia, Espírito Santo and Rio de Janeiro states), but two species are in montane areas of inland Bahia and Minas Gerais states and one species is in northern Brazil (Maranhão and Piauí states) (Fig. 82).

#### Ecology.

Most species occur in the Atlantic Forest, commonly in moist coastal forest or associated with coastal vegetation on sand soil (restinga). Three species occur in savanna vegetation, mainly in rocky grasslands.

#### Etymology.

The genus is named after Johann Jakob Moldenhawer (1766–1827), German professor of botany at Kiel and one of the founders of plant anatomy.

#### Human uses.

The yellow densely flowered inflorescences make the genus potentially of interest as an ornamental for gardens and as street trees. The red timber from *M.intermedia* G.P. Lewis & L.P. Queiroz is used for furniture (Lewis and Queiroz 2010).

#### Notes.

*Moldenhawera* is characterised by the Malpighiaceae-like bilaterally symmetrical flowers, hypanthium infilled and the unusual androecium with a single fertile stamen with bearded connective and seven or nine short staminodes. The genus presents variation in the leaf division, with pinnate and/or bipinnate leaves, sometimes in the same species. The last taxonomic revision of the genus, including a key to all species, was provided by Vivas and Queiroz (2020).

#### Taxonomic references.

Lewis and Queiroz (2010); Queiroz et al. (1999); Vivas and Queiroz (2020); Yakovlev (1975).

### ﻿ Diptychandra

Taxon classificationPlantaeFabalesFabaceae

Tul., Ann. Sci. Nat., Bot. sér. 2, 20: 139. 1843.

**Figure 83.**
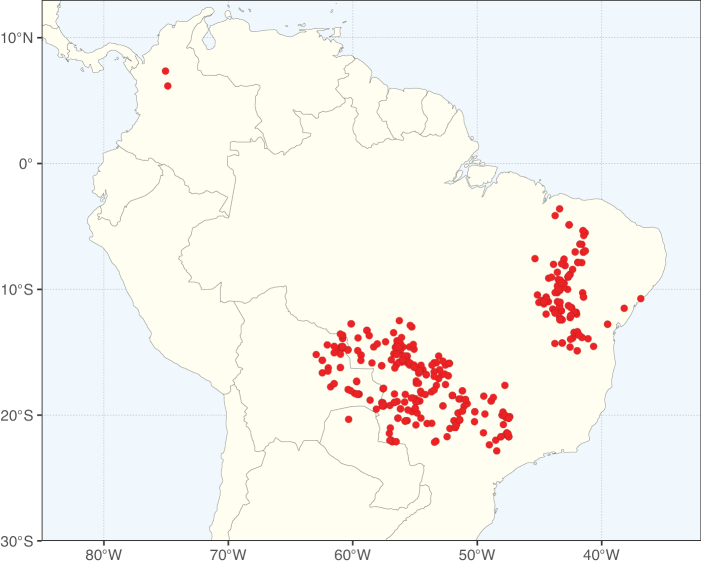
Distribution of *Diptychandra* based on quality-controlled digitised herbarium records. See Suppl. material 1 for the source of occurrence data.

Figs 79 , 80 , 81 , 83

#### Type.

*Diptychandraaurantiaca* Tul.

#### Description.

Trees or shrubs, unarmed. **Stipules** scale-like. **Leaves** paripinnate; leaflets 2–4, opposite. **Inflorescences** in terminal unbranched racemes, 3.5–20 cm long; bracts and bracteoles minute, caducous. **Flowers** radially symmetrical; hypanthium cupulate; sepals 5, free, often with red dots; petals 5, yellow, clawed; androecium monomorphic, stamens 10, free; pollen in tetrads, moderately reticulate; ovary stipitate, stigma punctiform. **Fruit** a dehiscent, linear-oblong, compressed legume, the two valves ligneous with resinous dots, 1–2 (4)-seeded. **Seeds** ellipsoid-complanate, winged.

#### Chromosome number.

Unknown.

#### Included species and geographic distribution.

Three species, one of which is widespread throughout most of western Brazil extending to Bolivia and Paraguay; and the remaining two are each endemic to north-eastern Brazil and Colombia (Fig. 83).

#### Ecology.

Mostly in savannas and seasonally dry forests, except for *D.granadillo* C. Romero & Arbeláez which occurs in Andean mountain forests.

#### Etymology.

The generic name is derived from Greek, *diptycho*- (= twice folded) and -*andro* (= stamen), the stamen filaments are twice folded in bud.

#### Human uses.

Species of the genus are used as timber for construction and charcoal. The wood of *D.granadillo* has been reported to be very hard and difficult to saw (Romero-Hernández and Arbeláez 2017).

#### Notes.

*Diptychandra* is characterised by the paripinnate leaves and the flowers radially symmetrical with cupulate hypanthium and sepals often with red dots. The fruits are dehiscent, linear-oblong, bearing valves with resinous dots and 1–2(4) winged seeds. The last taxonomic revision of the genus, including a key to all species, was provided by Escobar (2018).

#### Taxonomic references.

Escobar (2018); Lima et al. (1990); Lima and Kuntz (2020); Romero-Hernández and Arbeláez (2017); Tulasne (1843).

### ﻿ Arapatiella

Taxon classificationPlantaeFabalesFabaceae

Rizzini & A. Mattos, Rev. Bras. Biol. 32(3): 323. 1972.

**Figure 84.**
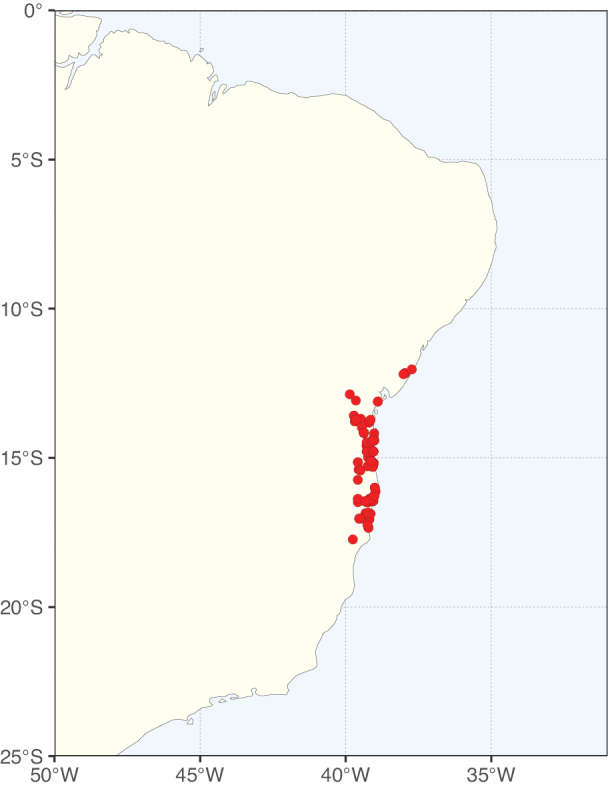
Distribution of *Arapatiella* based on quality-controlled digitised herbarium records. See Suppl. material 1 for the source of occurrence data.

Figs 79 , 80 , 81 , 84

#### Type.

*Arapatiellatrepocarpa* Rizzini & A. Mattos [= *Arapatiellapsilophylla* (Harms) R.S. Cowan]

#### Description.

Trees, unarmed. **Stipules** foliaceous, orbiculate to reniform. **Leaves** paripinnate; leaflets 2–4, opposite. **Inflorescences** in terminal paniculate racemes, 4–10 cm long; bracts and bracteoles small, caducous. **Flowers** radially symmetrical; hypanthium turbinate; sepals 5, free; petals 5, white, clawed; androecium monomorphic, stamens 10, free; pollen grains in monads, associated with viscin threads, coarsely reticulate; ovary stipitate, stigma disciform-peltate. **Fruit** a linear-oblanceolate, laterally compressed legume, elastically dehiscent, the woody valves coiling backwards from the apex at dehiscence, 4–5 (8)-seeded. **Seeds** ellipsoid-compressed.

#### Chromosome number.

Unknown.

#### Included species and geographic distribution.

Two species, *A.emarginata* R.S. Cowan and *A.psilophylla*, both occurring in eastern Brazil (Fig. 84).

#### Ecology.

The genus is only known from lowland Atlantic moist coastal forests on “tabuleiro terciário” of southern Bahia and northern Espírito Santo states in Brazil.

#### Etymology.

The generic name is derived from the local vernacular name “arapati”.

#### Human uses.

The species of the genus are used as timber for construction.

#### Notes.

*Arapatiella* is characterised by the foliaceous stipules, orbiculate to reniform, leaves paripinnate, and flowers radially symmetrical with turbinate hypanthium and ten exserted stamens. The fruits are elastically dehiscent from the apex, the woody valves coiling backwards at dehiscence. A taxonomic study of the genus, including a key to all species, was provided by Lima and Kuntz (2020).

#### Taxonomic references.

Cowan (1973, 1981b); Lewis (2005b); Lima et al. (2020a); Rizzini and Mattos (1972).

### ﻿ Jacqueshuberia

Taxon classificationPlantaeFabalesFabaceae

Ducke, Arch. Jard. Bot. Rio de Janeiro 3: 118. 1922.

**Figure 85.**
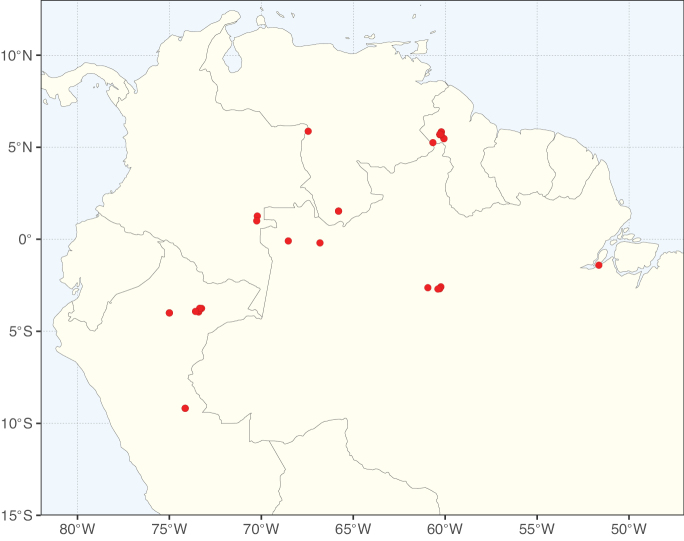
Distribution of *Jacqueshuberia* based on quality-controlled digitised herbarium records. See Suppl. material 1 for the source of occurrence data.

Figs 79 , 80 , 81 , 85

#### Type.

*Jacqueshuberiaquinquangulata* Ducke

#### Description.

Small trees or shrubs, unarmed. **Stipules** pinnate. **Leaves** bipinnate; pinnae and leaflets opposite, (2) 4–30 pairs of pinnae, 7–80 pairs of leaflets per pinna. **Inflorescences** terminal racemes, elongated or corymbiform; bracts and bracteoles setaceous, caducous. **Flowers** bilaterally symmetrical; hypanthium cupulate or campanulate, slightly ribbed; sepals 5, free; petals 5, yellow or dark purplish red, ovate, slightly unequal, sessile, lacking a claw; androecium monomorphic, stamens 10, joined in lower part; pollen in monads, associated with viscin threads, coarsely reticulate; ovary sessile, stigma oblique capitate. **Fruit** a dehiscent, linear, compressed legume, ribbed on the edges, the two ligneous valves coiling backwards from the apex at dehiscence, 4–8-seeded. **Seeds** oblong-ellipsoid, compressed.

#### Chromosome number.

Unknown.

#### Included species and geographic distribution.

Seven species across north-western South America, where two are endemic in Amazonian Brazil, two in Venezuelan Guayana, one in Guyana, one in Colombia and one in Peru (Fig. 85).

#### Ecology.

White sand forests of the Amazon basin, montane forests on sandstone and savannas in the Guiana Shield.

#### Etymology.

The generic name honours Jacques E. Huber (1867–1914), a Swiss botanist who explored the Amazon region.

#### Human uses.

Potentially of interest as an ornamental because of its wide range of flower colour, including red, purple, and yellow.

#### Notes.

*Jacqueshuberia* is characterised by the combination of foliaceous and pinnate stipules, bipinnate leaves, the pinnae and leaflets opposite, flowers bilaterally symmetrical with a cupular hypanthium and ten exserted stamens. The fruits are dehiscent, linear, with 4–8 seeds. The genus was last revised by Silva and Graham (1980), including a key to all species. Four species were subsequently described (Cowan 1985; Barneby 1990; Stergios and Berry 1996).

#### Taxonomic references.

Barneby (1990); Cowan (1985); Ducke (1922); Silva and Graham (1980); Stergios and Berry (1996).

### ﻿ Tachigali

Taxon classificationPlantaeFabalesFabaceae

Aubl., Hist. Pl. Guiane: 372. 1775.

**Figure 86.**
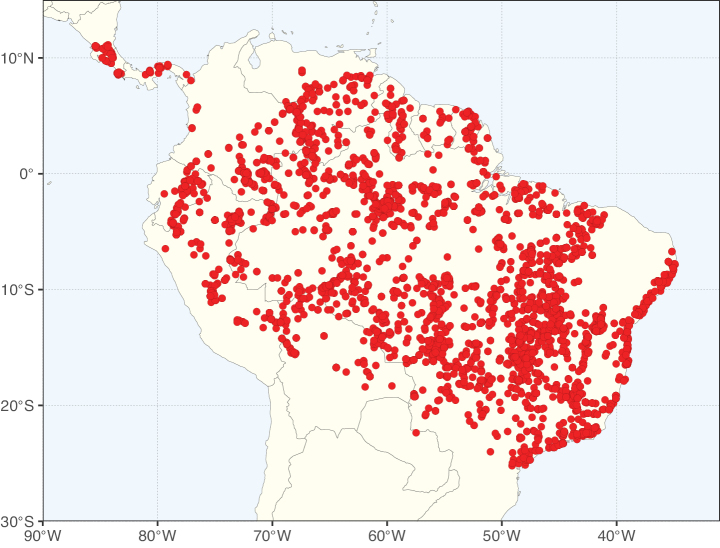
Distribution of *Tachigali* based on quality-controlled digitised herbarium records. See Suppl. material 1 for the source of occurrence data.

Figs 79 , 80 , 81 , 86

*Cuba* Scop., Intr. Hist. Nat.: 300. 1777. Type not designated.
Cubaea Schreb., Gen. Pl.: 278. 1789. Type not designated.
Valentinia Neck., Elem. Bot. 2: 450. 1790, opus utique oppr.
Tachia Pers., Syn. Pl. 1: 459. 1805, non Tachia Aublet, Hist. Pl. Guiane: 75. 1775. (Gentianaceae). Type: Tachiapaniculata Pers. [≡ Tachigalipaniculata Aubl.]
Sclerolobium Vogel, Linnaea 11: 395. 1837. Type: Sclerolobiumdenudatum Vogel [≡ Tachigalidenudata (Vogel) Oliveira-Filho]

#### Type.

*Tachigalipaniculata* Aubl.

#### Description.

Trees, unarmed**. Stipules** foliaceous, pinnate or pectinate; persistent to caducous. **Leaves** paripinnate; leaflets 2–20 pairs, opposite and inversely symmetrical; petiole and/or rachis usually with myrmecophilous domatia. **Inflorescences** in paniculate terminal racemes or in leaf axils of terminal branches; bracts equal in shape to, but smaller than, the stipules; bracteoles minute, lanceolate or subulate. **Flowers** radially or bilaterally symmetrical; hypanthium cupulate or obliquely cylindrical; sepals 5, free; petals 5, yellow or orange, lineate, lanceolate or spathulate, sometimes clawed; stamens 10, rarely 15–16, monomorphic with equal filaments, or dimorphic with 7 filaments longer, subulate, and 3 shorter, falcate or sigmoidal; pollen in monads, finely reticulate; ovary stipitate, stigma truncate. **Fruit** an indehiscent, compressed, oblong-elliptic or oblong, 1–3-seeded cryptosamara; exocarp flaking at maturity; mesocarp surrounded by a subligneous and thin wing; endocarp hyaline and membranous. **Seeds** oblong-ellipsoid, compressed.

#### Chromosome number.

2*n* = 24 (Coelho 2014).

#### Included species and geographic distribution.

Seventy-eight formally described species, but recent taxonomic estimates suggest the genus may include more than 90 species (Huamantupa-Chuquimaco et al. 2019). *Tachigali* is a Neotropical genus, widely distributed from Honduras through Central America to southern Brazil and Bolivia in South America (Fig. 86).

#### Ecology.

Three species occur in evergreen and semi-deciduous lowland forests of Central America, *T.costaricensis* (N. Zamora & Pùveda) N. Zamora & van der Werff, *T.panamensis* van der Werff & N. Zamora, and *T.versicolor* Standl. & L.O. Williams (Foster 1977; van der Werff and Zamora 2010). The South American species occur mainly in the Amazon region, extending to the Brazilian Atlantic coastal rainforests. The greatest diversity is concentrated in the Amazon rainforest with 60 species, but other biomes such as the savannas of central/north-east Brazil and the Atlantic rainforests in southern Brazil also have high levels of richness and endemism (Dwyer 1954, 1957; van der Werff 2008; Silva et al. 2016; Huamantupa-Chuquimaco et al. 2020). Many Amazonian forest *Tachigali* species are ant-housing plants, which have important biotic interactions with big-eyed arboreal ants (Pseudomyrmecinae) (Ducke 1949; Dwyer 1954). Monocarpy is well established in *Tachigali* and has been reported for *T.versicolor* (Foster, 1977), *T.vasquezii* (Poorter et al. 2005), *T.argyrophylla* Ducke, *T.chrysaloides* van der Werff, *T.melinonii* (Harms) Zarucchi & Herend. and *T.loretensis* van der Werff (Huamantupa-Chuquimaco 2020).

#### Etymology.

The generic name is derived from the vernacular name “tachi” for stinging ants.

#### Human uses.

Used as timber for construction and charcoal. *Tachigalivulgaris* L.F. Gomes da Silva & H.C. Lima is planted in forest restoration (Ramos et al. 2021) and the bark of *T.tinctoria* (Benth.) Zarucchi & Herend. is used in tanning and as a dye.

#### Notes.

*Tachigali* is recognisable by the combination of paripinnate leaves, the leaflets inversely symmetrical, stipules foliaceous and mostly pinnate, the petiole and/or rachis usually with myrmecophilous domatia, and very distinct strongly laterally compressed wind-dispersed fruits (cryptosamaras). Floral symmetry marks a major subdivision within *Tachigali*, and was once used to define two genera (Dwyer 1954, 1957; Polhill and Vidal 1981), in which the more radially symmetrical-flowered old sense genus *Sclerolobium* was treated as separate from the bilaterally symmetrical-flowered *Tachigali* s.s. However, increasing evidence from wood anatomy (Gasson et al. 2003; Macedo et al. 2014), pollen morphology (Graham and Barker 1981; Banks and Lewis 2018), comparative flower development (Casanova et al. 2020), and overall floral, fruit, and leaf morphology (van der Werff 2008; Huamantupa-Chuquimaco et al. 2020) strongly support the merging of the two genera. The grouping of the two genera is also supported by recent phylogenetic analyses, which show no support for generic separation (LPWG 2017; Huamantupa-Chuquimaco 2020). A taxonomic synopsis of the genus in northern South America was provided by van der Werff (2008) and the Brazilian species are currently being studied by Huamantupa-Chuquimaco et al. (2020).

#### Taxonomic references.

Dwyer (1954, 1957); Lewis (2005b); Huamantupa-Chuquimaco et al. (2020); Polhill and Vidal (1981); van der Werff (2008).

## ﻿﻿9. Tribe Dimorphandreae

Guilherme Sousa da Silva^39^, Marcelo F. Simon^40^

Citation: Silva GS, Simon MF (2024) 9. Tribe Dimorphandeae. In: Bruneau A, Queiroz LP, Ringelberg JJ (Eds) Advances in Legume Systematics 14. Classification of Caesalpinioideae. Part 2: Higher-level classification. PhytoKeys 240: 177–186. https://doi.org/10.3897/phytokeys.240.101716

### ﻿Tribe Dimorphandreae

Taxon classificationPlantaeFabalesFabaceae

Benth., J. Bot. (Hooker) 2: 74. 1840.

**Figure 87.**
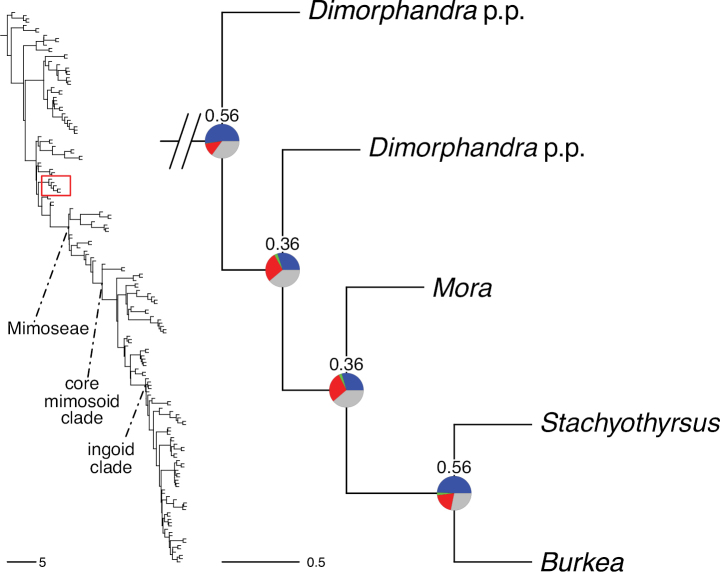
Generic relationships in tribe Dimorphandreae. For description of phylogeny and support values, see Fig. 6 caption (page 63).

**Figure 88.**
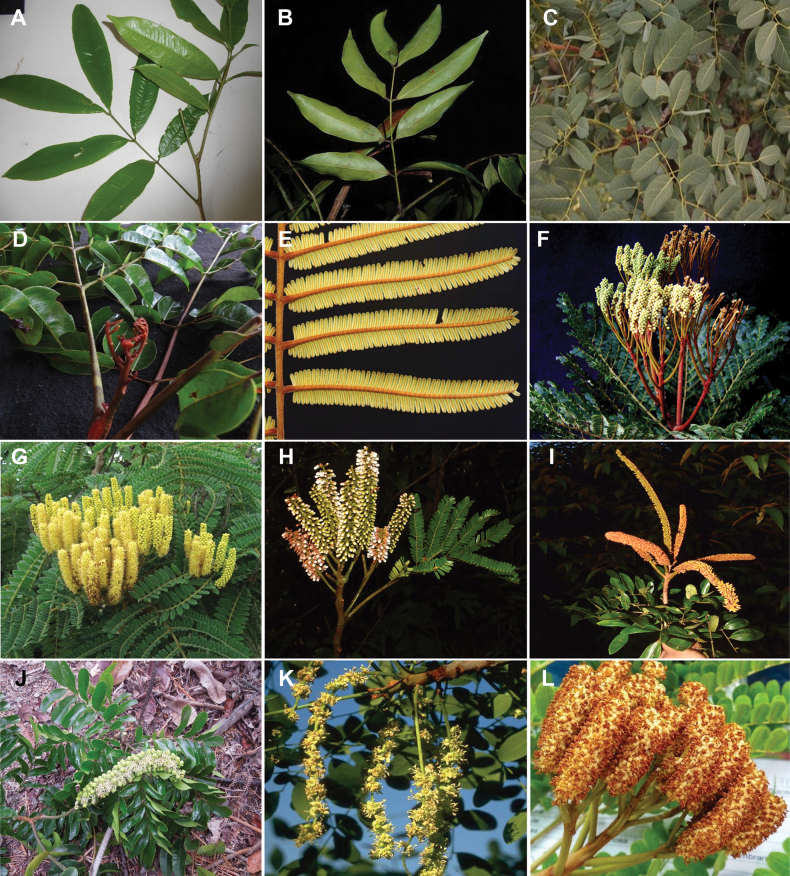
Leaves and inflorescences of tribe Dimorphandreae**A***Moraparaensis* (Ducke) Ducke (*Simon 1663*) **B***Stachyothyrsusstaudtii* Harms **C***Burkeaafricana* Hook. **D***Dimorphandramediocris* Ducke (*Simon 4209*) **E***Dimorphandracuprea* Sprague & Sandwith (*Farroñay 1804*) **F***Dimorphandraparviflora* Spruce ex Benth. (*Simon 1176*) **G***Dimorphandramollis* Benth. **H***Dimorphandrapennigera* Tul. **I***Dimorphandraignea* Ducke **J***Dimorphandravernicosa* Spreng. ex Benth. (*Cardoso 3279*) **K***Burkeaafricana***L***Dimorphandragardneriana* Tul. (*Silva 21*). Photo credits **A, D, F, G** MF Simon **B** Nicolas Texier (CC-BY-NC-ND-3.0) **C** AR Lecuona (CC-BY-NC-4.0) **E, H, I** F Javier Farroñay Pacaya **J** D Cardoso **K** AE van Wyk and S Malan **L** G Sousa da Silva.

**Figure 89.**
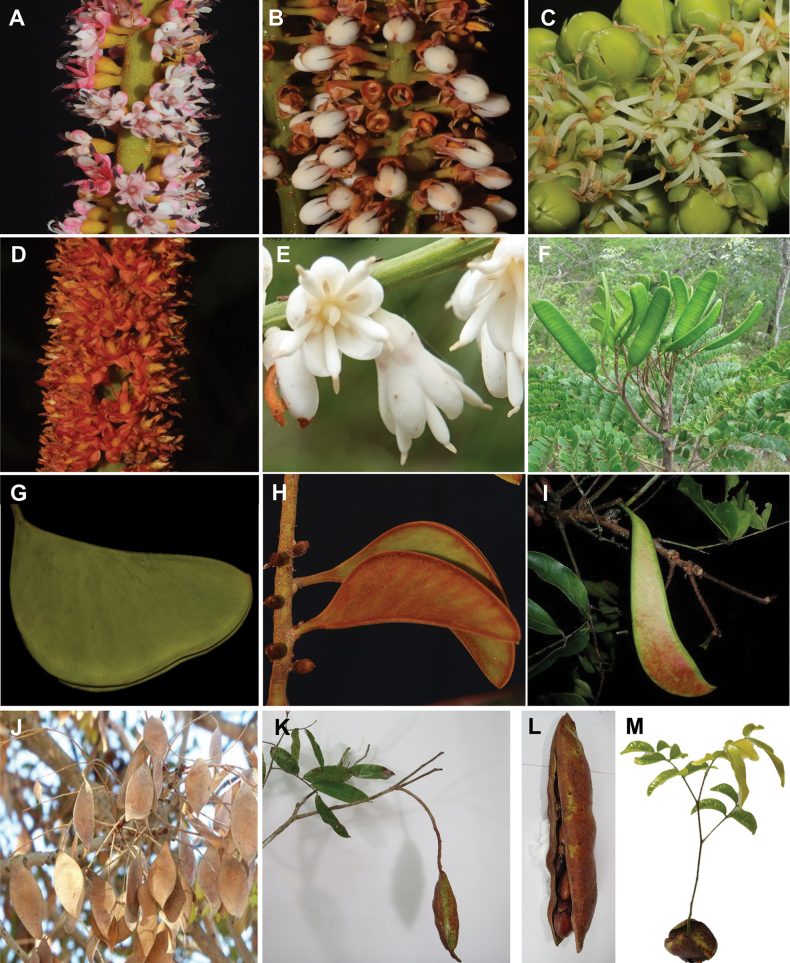
Flowers and fruits of the tribe Dimorphandreae**A***Dimorphandracuprea* Sprague & Sandwith (*Farroñay 1804*) **B***Dimorphandrapennigera* Tul. **C***Dimorphandravernicosa* Spreng. ex Benth. (*Cardoso 3279*) **D***Dimorphandraignea* Ducke **E***Stachyothyrsusstaudtii* Harms **F***Dimorphandragardneriana* Tul. (*Simon 2715*) **G***Dimorphandramacrostachya* Benth. (*Silva 540*) **H***Dimorphandracuprea* Sprague & Sandwith (*Farroñay 1804*) **I***Stachyothyrsusstaudtii* Harms **J***Burkeaafricana* Hook. **K–M***Moraparaensis* (Ducke) Ducke (**K, L***Costa 28*). Photo credits **A, B, D, H** F Javier Farroñay Pacaya **C** D Cardoso **E** Flora of the world **F** MF Simon **G** G Sousa da Silva **I** N Texier (CC-BY-NC-ND-3.0) **J** C Sydes (CC-BY-NC-4.0) **K, L** J Barbosa Pedrosa Costa **M** A Rocha Dantas.

**Figure 90.**
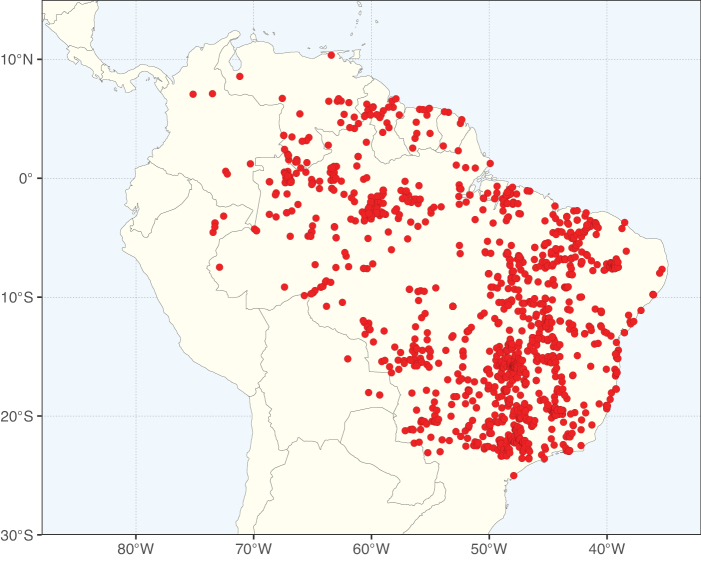
Distribution of *Dimorphandra* based on quality-controlled digitised herbarium records. See Suppl. material 1 for the source of occurrence data.

**Figure 91.**
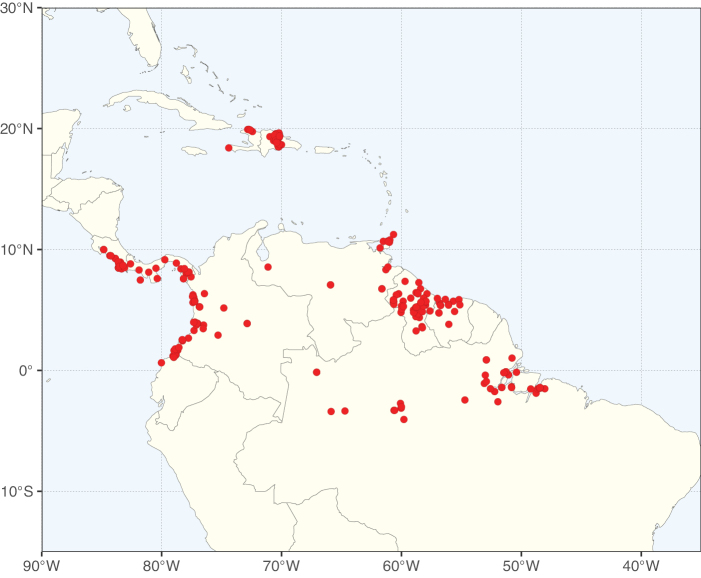
Distribution of *Mora* based on quality-controlled digitised herbarium records. See Suppl. material 1 for the source of occurrence data.

**Figure 92.**
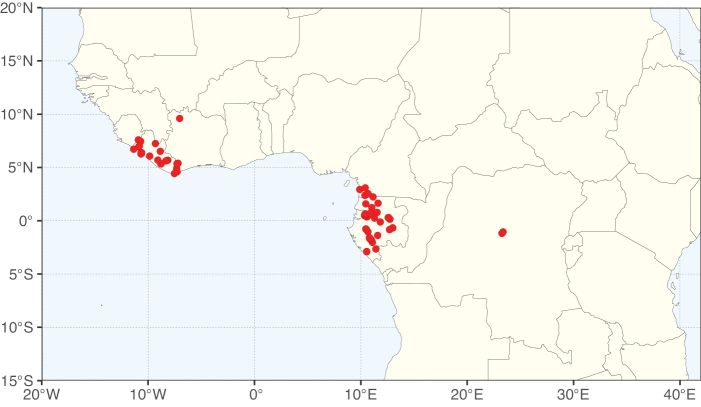
Distribution of *Stachyothyrsus* based on quality-controlled digitised herbarium records. See Suppl. material 1 for the source of occurrence data.

**Figure 93.**
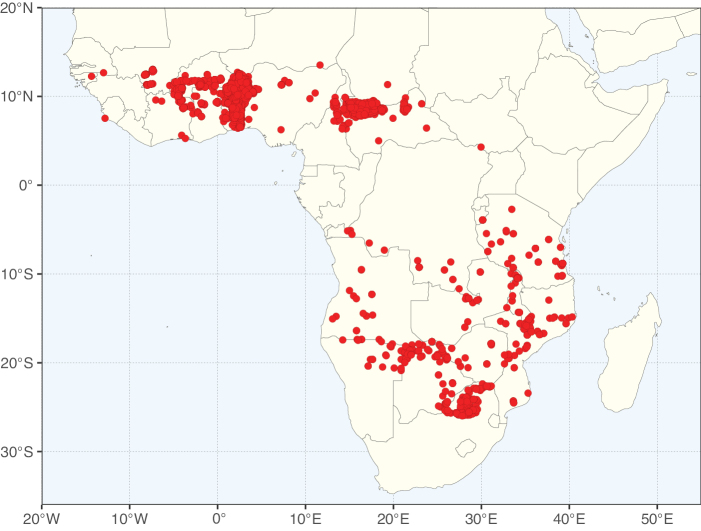
Distribution of *Burkea* based on quality-controlled digitised herbarium records. See Suppl. material 1 for the source of occurrence data.

Figs 87 , 88 , 89 , 90 , 91 , 92 , 93

Dimorphandrinae Walp., Repert. Bot. Syst. 1: 854. 1843. Type: Dimorphandra Schott
Moreae Britton & Rose, N. Amer. Fl. 23: 201, 217. 1930. Type: Mora Benth.

#### Type.

*Dimorphandra* Schott

#### Included genera

**(4).***Burkea* Hook. (1 species), *Dimorphandra* Schott (26), *Mora* Benth. (6), *Stachyothyrsus* Harms (2).

#### Description.

Unarmed shrubs, treelets to canopy trees up to 40 m high; trunk buttressed or not; brachyblasts absent; branches glabrous or pilose. **Stipules** present or absent. **Leaves** pinnate or bipinnate; extrafloral nectaries on the petiole occurring only in *Stachyothyrsus*; pinnae (in bipinnate leaves) 1-many pairs; leaflets 1-many pairs, opposite or alternate, variable in size and shape. **Inflorescences** short or elongated spiciform racemes or spikes, often arranged in corymbose or paniculate synflorescences; bracteoles small or absent. **Flowers** 5-merous, diplostemonous; stamens alternate, sometimes with 5 fertile and 5 staminodes; anther glands absent or present (*Burkea*); pollen tricolpate monads. **Fruit** a typical legume or samara (*Burkea*), dehiscent or indehiscent, variable in size and shape, 1-multiseeded. **Seeds** flat-compressed to ovoid, with a hard or thin testa, areolas absent, present on both sides only in *Burkea*.

#### Distribution.

Rainforests, seasonally dry forests, and savannas, in tropical regions of the Americas and Africa.

#### Clade-based definition.

The most inclusive crown clade containing *Dimorphandragardneriana* Tul. and *Burkeaafricana* Hook., but not *Campsiandracomosa* Benth., *Tachigaliguianensis* (Benth.) Zarucchi & Herend. or *Schizolobiumparahyba* (Vell.) S.F. Blake (Fig. 87).

#### Notes.

The informal Dimorphandra group of the old sense subfamily Caesalpinioideae, which originally included 10 genera (Polhill and Vidal 1981; Polhill 1994), has been shown to be non-monophyletic (Bruneau et al. 2001, 2008; LPWG 2017), but the four genera included here in Dimorphandreae have been supported as a clade in these previous analyses, albeit with low support and poor resolution amongst the genera. The phylogenomic analyses of Ringelberg et al. (2022) support the monophyly of the tribe and reveals an amphi-Atlantic distribution, with the two South American genera, *Dimorphandra* and *Mora*, sequentially sister to the two African genera, *Burkea* and *Stachyothyrsus*.

Members of the Dimorphandreae (Figs 88, 89) are unarmed trees, with leaves lacking extrafloral nectaries (except *Stachyothyrsus*). They have spicate inflorescences, 5-merous diplostemonous flowers lacking anther glands (except *Burkea*), with a short style, and tricolpate single-celled pollen grains. Within Dimorphandreae, *Mora* is morphologically similar to *Dimorphandra*, sharing flowers with five fertile stamens and five staminodes, but can be readily distinguished by once pinnate, glabrous leaves (vs. bipinnate and pubescent in *Dimorphandra*), and seed size (much larger in *Mora*). The African sister genera *Stachyothyrsus* and *Burkea* share stamens arising at the insertion or just slightly above the ovary insertion (Cowan 1981a). Within the tribe, *Stachyothyrsus* can be recognised by the dimorphic stamens and presence of petiolar nectaries, while *Burkea* is distinguished by inflorescences and young leaves clustered at the tip of shoots and fruits bearing a single seed. Bees are the main pollinating agents for species of the tribe, as described for *Burkea* and some species of *Dimorphandra* and *Mora* (Silva 1986), and possibly *Stachyothyrsus* (Brink 2010). A survey including several representatives of the tribe recorded single-grain, tricolpate pollen (Banks and Lewis 2009). Seed dispersal varies in the group.

Although Dimorphandreae forms a well-supported clade, *Dimorphandra*, the largest genus in the tribe, was recovered as non-monophyletic (Fig. 87), and new generic circumscriptions are required.

### ﻿ Dimorphandra

Taxon classificationPlantaeFabalesFabaceae

Schott, Syst. Veg. [Sprengel] 4(2): 404. 1827.

Figs 88 , 89 , 90

#### Type.

*Dimorphandraexaltata* Schott

#### Description.

Unarmed small or medium trees 3–5 (7) m, to canopy trees 40 (50) m; trunk buttressed or not; brachyblasts absent; usually pubescent in all parts with reddish to dark or light brown hairs. **Stipules** present or absent, caducous. **Leaves** bipinnate, rachis 5–90 cm long; pinnae 1–40 or more pairs; leaflets 1–50 pairs, opposite or alternate, commonly glabrous above and pubescent below, venation brochidodromous. **Inflorescences** short or elongated spiciform racemes, often arranged in paniculate or corymbose synflorescences; bracteoles caducous or absent. **Flowers** small, pale yellow to cream or dark orange to reddish, fragrant, 5-merous, diplostemonous; calyx cupuliform, tubular or campanulate; petals obovate, oblong or spatulate, glabrous or pilose; fertile stamens 5, filaments thin, with oblong, dorsifixed anthers, anther glands absent; staminodes 5, spatulate, free or connate forming a dome, with or without a rudimentary anther at the apex, usually deciduous at anthesis; pollen tricolpate monads; ovary sessile, subsessile, or stipitate, multi-ovulate, glabrous or densely hairy, style short to absent, stigma conical, terminal. **Fruit** dehiscent or indehiscent legumes, linear-oblong, curved or suborbicular, flat, valves leathery or woody. **Seeds** orbicular, flat, cylindrical or oblong.

#### Chromosome number.

2*n* = 28 (Muniz et al. 2020).

#### Included species and geographic distribution.

26 species, including four subspecies (Silva 1986; Silva 2019), restricted to the Neotropics, occurring mostly in Brazil (23 species) and also in Bolivia, Colombia, Guyana, Peru and Venezuela (Fig. 90).

#### Ecology.

Predominates in tropical rainforests, including both terra firme and seasonally flooded (igapó) forests, white-sand forests (campinarana), savannas, and less often in seasonally dry forests. Fruits of *D.mollis* Benth. are important food resources for tapirs (Bizerril et al. 2005).

#### Etymology.

*Dimorphandra* refers to the androecium that has five stamens alternating with five staminodes, that is, two morphological forms of the stamens.

#### Human uses.

*Dimorphandra* species have the ability to fix nitrogen (Fonseca et al. 2012) and are therefore important for improving soil fertility. Fruits are used in traditional medicine (Féres et al. 2006). Extracts from *D.gardneriana* Tul. and *D.mollis* fruits containing the bioflavonoids rutin and quercetin are used by cosmetic and pharmaceutical industries, sometimes causing decline in natural populations (Filizola 2013).

#### Notes.

The genus is classified into three subgenera: *Dimorphandra* (11 species), *Phaneropsia* Tul. (5 species) and *Pocillum* Tul. (10 species), which can be differentiated by leaf morphology, inflorescence architecture and fruit shape (Silva 1986; Silva 2019). *Dimorphandra* seeds are dispersed by authocory, hydrochory or zoochory (Silva 1986). *Dimorphandra* is not recovered as monophyletic in recent phylogenomic analyses (Fig. 87) in which representatives of subgenera *Phaneropsia* and *Pocillum* (*D.davisii* Sprague & Sandwith and *D.macrostachya* Benth., respectively) form a clade independent from subgenus Dimorphandra (*D.gardneriana*). Other studies with increased taxonomic sampling found similar results, but relationships were uncertain because of phylogenetic conflict (Rocha et al. 2024). Future studies with an expanded taxonomic sample and robust topology are required for a new circumscription of the genus.

#### Taxonomic references.

Bentham (1840); Ducke (1925); Ragonese (1973); Silva (2019); Silva and Hopkins (2018); Silva (1981, 1986); Taubert (1894).

### ﻿ Mora

Taxon classificationPlantaeFabalesFabaceae

Benth., Trans. Linn. Soc. London 18(2): 210, pl. 16–17. 1839.

Figs 88 , 89 , 91

#### Type.

*Moraexcelsa* Benth.

#### Description.

Unarmed large trees, 15–45 m; trunk buttressed; brachyblasts absent; glabrous. **Stipules** small, caducous. **Leaves** paripinnate, alternate; petiole 2–6 cm long, rachis 5–10 cm long; leaflets (1) 2–6 (7) pairs, opposite, large, long-acuminate, glabrous and smooth, secondary veins inconspicuous. **Inflorescences** spiciform racemes arranged in paniculate synflorescences. **Flowers** small, white or yellow, 5-merous, diplostemonous; bracteoles small, caducous; calyx with a very short tube and short ciliated lobes; petals oblong or ovate, finely ciliated at the apex; fertile stamens 5, filaments thick, anthers covered with caducous white hairs, anther glands absent; staminodes 5; pollen tricolpate monads; ovary sessile or nearly so, few-ovuled, style compressed with a thin terminal stigma. **Fruit** a dehiscent legume, flat, elliptic or oblong, coriaceous to woody, the valves twist after dehiscence. **Seeds** large, flattened or suborbicular, with a membranous testa.

#### Chromosome number.

Unknown.

#### Included species and geographic distribution.

The genus comprises six species occurring in Central America (Panama, Costa Rica), northern South America (Brazil, Colombia, Ecuador, Guyana, Suriname, Venezuela) and Greater Antilles (Haiti, Dominican Republic and Trinidad Tobago) (Fig. 91).

#### Ecology.

*Mora* species generally occur in periodically flooded forests, swamps and mangroves. *Moraexcelsa* forms monodominant forests in the Guianas (Ducke 1925).

#### Etymology.

Derived from the widely used Arawak vernacular name ‘mora’.

#### Human uses.

*Mora* species are valuable timber in the Guianas, being used in construction, industrial flooring and for charcoal (Lewis 2005b), while *M.paraensis* (Ducke) Ducke is used in house construction by Brazilian riverine communities (Ducke 1925). The seeds of *M.megistosperma* (Pittier) Britton & Rose are a local source of a red dye (Schembera 2004).

#### Notes.

*Mora* has a complex taxonomic history, having been treated under *Dimorphandra* by several authors. *Mora* can be readily distinguished from *Dimorphandra* by the paripinnate leaves, which are always glabrous (vs. bipinnate, often pubescent); anthers with conspicuous caducous white hairs (vs. glabrous); style longer than the ovary (vs. shorter); large, soft seeds, with a membranous testa (vs. small seeds with a hard testa) (Ducke 1925; Sandwith 1932). The large fruits and seeds of *Mora* species are well adapted to water dispersal (ter Steege 1994). Seeds of *M.megistosperma*, ca. 18 × 12 cm, are among the largest dicotyledonous seeds (Lewis 2005b).

#### Taxonomic references.

Bentham (1839, 1840); Ducke (1925); Britton and Rose (1930); Sandwith (1932); Tulasne (1844); ter Steege (1990).

### ﻿ Stachyothyrsus

Taxon classificationPlantaeFabalesFabaceae

Harms, Nat. Pflanzenfam. Nachtr. 1: 198. 1897.

Figs 88 , 89 , 92

Kaoue Pellegr., Bull. Soc. Bot. France 80: 464. 1933. Type: Kaouestapfiana (A. Chev.) Pellegr. [≡ Oxystigmastapfiana A. Chev. (≡ Stachyothyrsusstapfiana (A. Chev.) J. Léonard & Voorh.)]

#### Type.

*Stachyothyrsusstaudtii* Harms

#### Description.

Unarmed trees 20–25 (30) m; trunk not buttressed; brachyblasts absent; glabrous. **Stipules** small, caducous. **Leaves** bipinnate, rachis 4–20 cm; pinnae 2 pairs; leaflets 3–5 pairs per pinna, opposite, glabrous, venation reticulate. **Inflorescences** spiciform racemes, sometimes arranged in paniculate synflorescences; bracts triangular-rounded, persistent. **Flowers** small, whitish, fragrant, 5-merous, diplostemonous; calyx short, cupuliform; petals obovate-oblong; stamens dimorphic, the 5 antesepalous slightly longer than the 5 antepetalous (shorter), anther glands absent; pollen tricolpate monads; ovary short, 2–3-ovulate, style short, stigma slightly bilobed. **Fruit** dehiscent legume, curved, flat, 1–2-seeded. **Seeds** irregular shaped, dark.

#### Chromosome number.

Unknown.

#### Included species and geographic distribution.

Two species, restricted to the central-west coast of Africa in Ivory Coast, Liberia and Sierra Leone (*S.stapfiana* J. Leonard & Voorhoeve), and Cameroon, Gabon, Equatorial Guinea and Congo (*S.staudtii*) (Fig. 92).

#### Ecology.

*Stachyothyrsus* occurs in tropical rainforests, including swamps, along river banks, and secondary forests.

#### Etymology.

From Greek, *Stachys* (= spike) and *thyrsus* (= wand, panicle), in reference to the spicate inflorescences aggregated into showy panicles.

#### Human uses.

*Stachyothyrsusstapfiana* leaves are used for thatching, while its wood has only limited importance (Brink 2010).

#### Notes.

*Stachyothyrsus* is most closely related to *Burkea* (the genera have stamens arising at the insertion or just slightly above the ovary insertion), but they are easily distinguished by the opposite, less numerous and larger leaflets in *Stachyothyrsus*. In addition, *Stachyothyrsus* species occur only in rainforests along the west coast of Africa, whereas *Burkea* is widely distributed in African savannas and dry forests.

#### Taxonomic references.

Aubréville (1959); Hutchinson and Dalziel (1958); Savill and Fox (1967); Voorhoeve (1979).

### ﻿ Burkea

Taxon classificationPlantaeFabalesFabaceae

Hook., Icon. Pl. 6: t. 593. 1843.

Figs 88 , 89 , 93

#### Type.

*Burkeaafricana* Hook.

#### Description.

Unarmed shrubs or small trees, exceptionally reaching 20–30 (35) m; trunk not buttressed; brachyblasts absent; indumentum on leaves and inflorescences composed by simple ferrugineous trichomes. **Stipules** minute, caducous. **Leaves** bipinnate, clustered at the tip of the branches; petiole and rachis together 7–32 cm long; pinnae (1) 2–5 (7) pairs; leaflets 5–15 (18) per pinna, alternate. **Inflorescences** elongate spiciform racemes 5–30 cm long, crowded at the tip of branches. **Flowers** small, whitish, sweet-scented, 5-merous, diplostemonous; calyx campanulate; petals obovate or elliptic-obtuse, glabrous; stamens 10, homomorphic; anthers oblong, anther glands present; pollen tricolpate monads; ovary subsessile, hirsute, 1–2-ovulate, style very short, thick, stigma conspicuous, capitate. **Fruit** samaroid, oblong or elliptical, flat, indehiscent, 1 (2)-seeded. **Seed** obovate, compressed, albuminous, cotyledon thin, flat, radicle short.

#### Chromosome number.

2*n* = 28 (Turner and Fearing 1959).

#### Included species and geographic distribution.

Monospecific (*B.africana*), widespread in Africa (except for the rainforest regions), occurring mainly in the west, centre and south of the continent, extending into Senegal, Sudan and Uganda, south to Namibia, Botswana and northern South Africa (Fig. 93).

#### Ecology.

The species inhabits savannas and seasonally dry forests at elevations from 40–1740 m. Trees with a bole diameter above 12.5 cm are fire resistant, being sufficiently protected by their corky bark. It is frequent and abundant in many regions (e.g., southern Africa), but often occurs in a dispersed and non-aggregated pattern.

#### Etymology.

Named after the British botanist Joseph Burke (1812–1873), who collected plants and animals (especially in South Africa) for the Earl of Derby.

#### Human uses.

An edible gum is produced from the stem, tender young leaves are cooked and eaten as a vegetable while young flowers are eaten in sauces. The bark, roots and leaves are commonly used in traditional medicine (Mathisen et al. 2002). The bark is used to treat fevers, coughs, colds, catarrh, pneumonia, stomach obstruction, menorrhoea, headaches, inflammation of tongue and gums, poisoning and skin diseases. The powdered bark is applied externally to ulcers and wounds, and to treat scabies. *Burkeaafricana* yields durable timber used in construction, carpentry and for charcoal (Neya et al. 2004; Lewis 2005b).

#### Notes.

*Burkea* is the most unusual genus within the tribe, with young leaves and inflorescences clustered at the tip of shoots, capitate stigma, and samaroid, monospermic fruits, whereas the other genera have leaves and inflorescence which are not clustered at the tip of shoots, small stigma, and multi-seeded legumes. The dispersal of the flat and dry fruits of *Burkea* is possibly by wind since the trees occur in dry and open environments (Wilson and Witkowski 2003).

#### Taxonomic references.

Arbonnier (2004); Brummitt et al. (2007); Burkill (1995); Dry (1993); Oliver (1871); Palmer and Pitman (1974).

## ﻿﻿10. Tribe Campsiandreae

Gwilym P. Lewis^10^

Citation: Lewis GP (2024) 10. Tribe Campsiandreae. In: Bruneau A, Queiroz LP, Ringelberg JJ (Eds) Advances in Legume Systematics 14. Classification of Caesalpinioideae. Part 2: Higher-level classification. PhytoKeys 240: 187–192. https://doi.org/10.3897/phytokeys.240.101716

### ﻿Tribe Campsiandreae

Taxon classificationPlantaeFabalesFabaceae

Legume Phylogeny Working Group, tribus nov.

urn:lsid:ipni.org:names:77339338-1

**Figure 94.**
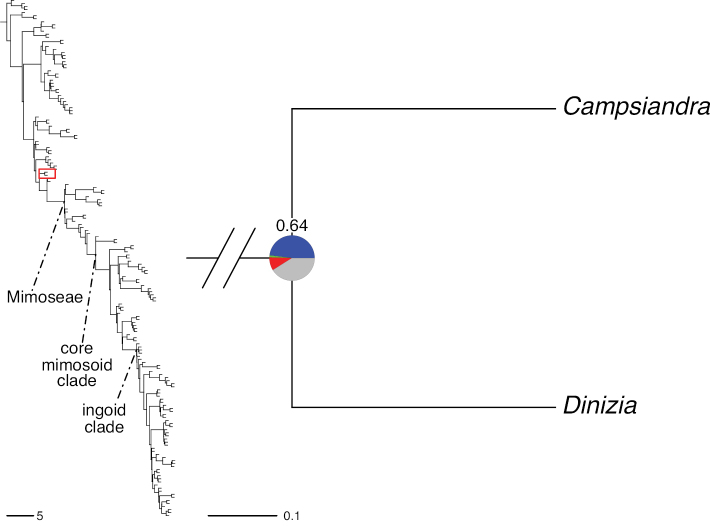
Generic membership and phylogenetic position of tribe Campsiandreae. For description of phylogeny and support values, see Fig. 6 caption (page 63).

**Figure 95.**
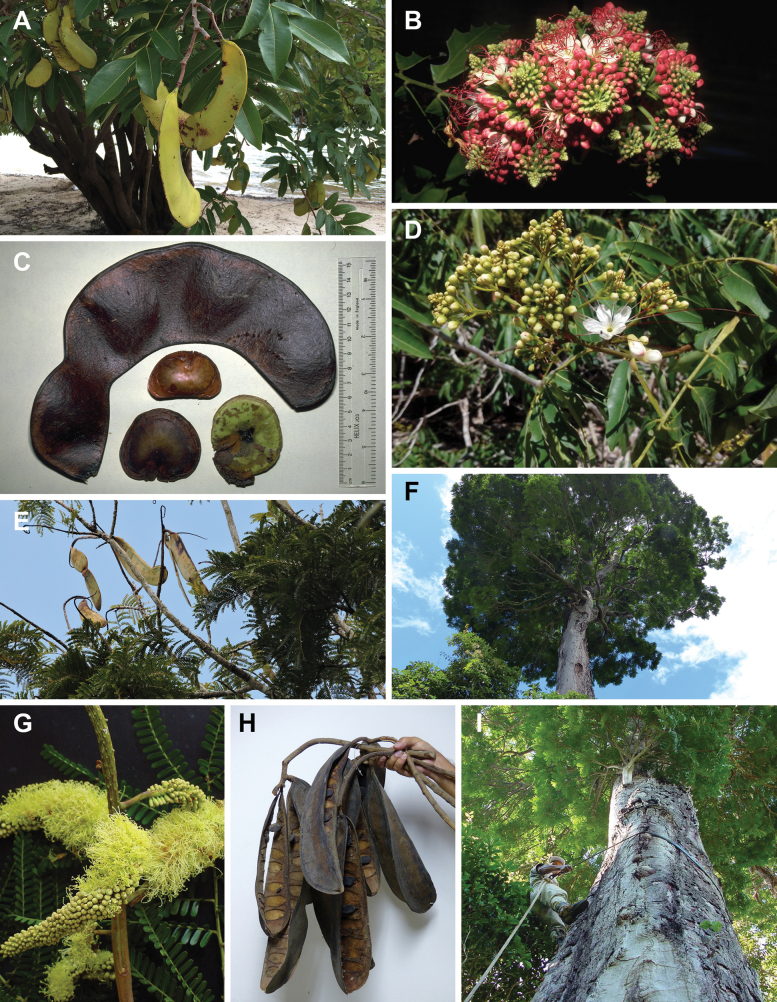
Flower, fruit and vegetative characters of tribe Campsiandreae**A** foliage and fruits, *Campsiandracomosa* Benth., Brazil **B** compound inflorescence, *Campsiandraangustifolia* Spruce ex Benth., Peru **C** fruit and seed, *Campsiandralaurifolia* Benth., cultivated Rio de Janeiro Botanic Gardens, Brazil **D** inflorescence and foliage, *Campsiandra* sp., Amazonas, Brazil **E–I***Diniziajueirana facao* G.P. Lewis & G.S. Siqueira, Reserva Natural Vale, Espírito Santo, Brazil **E** foliage and fruits **F** trunk and crown of mature tree **G** inflorescences **H** mature fruits (hand for scale) **I** rough bark of trunk. Photo credits **A** D Cardoso **B** T Pennington **D** M Falção **E, G, I** D Folli **C, F, H** GP Lewis.

**Figure 96.**
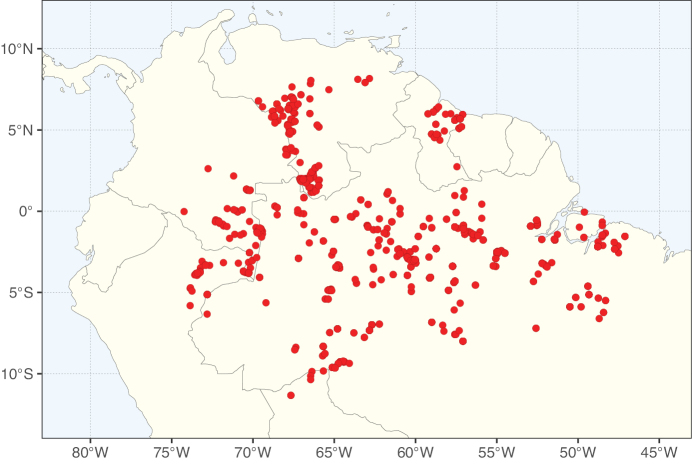
Distribution of *Campsiandra* based on quality-controlled digitised herbarium records. See Suppl. material 1 for the source of occurrence data.

**Figure 97.**
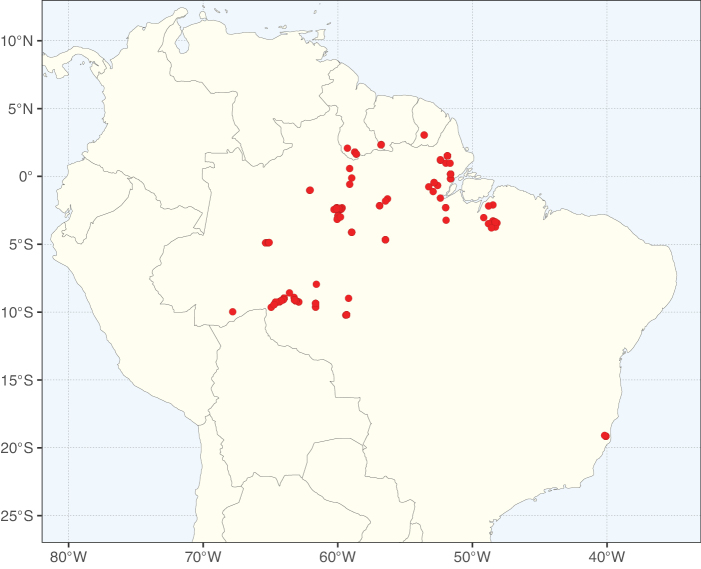
Distribution of *Dinizia* based on quality-controlled digitised herbarium records. See Suppl. material 1 for the source of occurrence data.

Figs 94 , 95 , 96 , 97

#### Diagnosis.

Campsiandreae is characterised by a combination of flowers with a cupular hypanthium, imbricate petals and stamens long-exserted from the corolla. The genus *Dinizia* is similar in appearance to genera of tribes Dimorphandreae and Mimoseae by its bipinnate leaves and small flowers in dense spicate racemes but differs by the combination of alternate leaflets, imbricate petal aestivation and all stamens fertile (in Mimoseae petals are valvate and genera of Dimorphandreae with bipinnate leaves and spicate inflorescences have opposite leaflets and an androecium comprising 5 fertile stamens and 5 staminodes). The genus *Campsiandra* is similar in appearance to *Arapatiella* Rizzini & A. Mattos (tribe Sclerolobieae) in having paripinnate leaves and regular perigynous flowers with a turbinate hypanthium and exserted stamens but differs by the variable number of stamens (vs. stamens 10 in *Arapatiella*), fruits indehiscent or inertly dehiscent, the valves not strongly twisting after dehiscence (vs. fruits elastically dehiscent from the apex, the valves rolling inwards in *Arapatiella*).

#### Type.

*Campsiandra* Benth.

#### Included genera

**(2).***Campsiandra* Benth. (3 to ca. 20 species), *Dinizia* Ducke (2).

#### Description.

Medium to large trees. **Stipules** caducous or lacking. **Leaves** pinnate with opposite, often gland-dotted leaflets, or bipinnate with alternate, eglandular leaflets; extrafloral nectaries absent. **Inflorescence** a compound spiciform raceme, or showy multi-flowered panicle. **Flowers** hermaphrodite or functionally staminate, pedicellate (the pedicels up to 3 cm long in *Campsiandra*); calyx with 5 imbricate lobes or a tube with 5 broadly triangular lobes; petals 5, imbricate, whitish (sometimes with rose-reddish markings), cream coloured or yellow; stamens 10–17 (25) per flower, exserted from the corolla, anthers eglandular; pollen in monads or tetrads; ovary stipitate. **Fruit** laterally compressed, coriaceous or woody, inertly dehiscent or indehiscent. **Seeds** discoid with a marginal spongy wing, or elliptic to obovate, hard, and wingless.

#### Distribution.

South America, mainly in the Amazon and Orinoco basins in flooded forests and swamp forests (*Campsiandra*) or in non-flooded Amazonian forests (*Diniziaexcelsa* Ducke), or semi-deciduous Atlantic rainforest (*Diniziajueirana-facao* G.P. Lewis & G.S. Siqueira).

#### Clade-based definition.

The most inclusive crown clade containing *Campsiandralaurifolia* Benth. and *Diniziaexcelsa* Ducke, but not *Delonixdecaryi* (R. Vig.) Capuron, *Dimorphandraconjugata* (Splitg.) Sandwith or *Mimosasensitiva* L. (Fig. 94).

#### Notes.

Phylogenetic analyses of a few molecular markers had suggested that the genus *Dinizia* could be more closely related to the Dimorphandra group of the old sense subfamily Caesalpinioideae, but with low support (Luckow et al. 2000, 2003; Wojciechowski et al. 2004; Bruneau et al. 2008; LPWG 2017). The position of *Dinizia* as sister to the Amazonian genus *Campsiandra* is more clearly supported in the phylogenomic analyses of Zhang et al. (2020) and Ringelberg et al. (2022), however both genera are subtended by a long branch. *Campsiandracomosa* Benth. and *Diniziajueirana-facao* are confirmed to nodulate with a fixation thread type of nodule anatomy (Faria et al. 2022). However, *Diniziaexcelsa* is reported to be non-nodulating (Sprent 2001; Lewis et al. 2017), making *Dinizia* one of the few Caesalpinioideae genera presently known to contain both nodulating and non-nodulating species.

### ﻿ Campsiandra

Taxon classificationPlantaeFabalesFabaceae

Benth., J. Bot. (Hooker) 2: 93. 1840.

Figs 95 , 96

#### Type

(here designated). *Campsiandralaurifolia* Benth.

#### Description.

Medium to large trees (6–25 m tall). **Stipules** inconspicuous and caducous. **Leaves** imparipinnate, 7–13-foliolate, leaflets in opposite pairs plus a single terminal leaflet, often gland-dotted although these frequently obscured by a waxy epidermal layer; extrafloral nectaries absent. **Inflorescence** a multi-flowered, often showy, terminal panicle (Fig. 95B, D). **Flowers** actinomorphic to zygomorphic, hermaphrodite, pedicellate, the pedicels 1–3 cm long and articulated just below the calyx; calyx with 5 imbricate lobes; petals 5, imbricate, white or with rose-reddish markings; stamens 10–17 per flower (Stergios 1998) or up to 25 (Polhill and Vidal 1981), the filaments dark red and showy, exserted from the corolla; pollen in perforate monads; gynoecium glabrous, stipitate. **Fruit** a large, coriaceous to sub-woody, straight to falcate, laterally compressed legume (Fig. 95A, C), the two valves usually coiling upon dehiscence. **Seeds** discoid, the testa expanded into a spongy wing (Fig. 95C) which circles the seed and aids flotation and thus water dispersal.

#### Chromosome number.

Unknown.

#### Included species and geographic distribution.

The number of species in the genus varies greatly depending on the treatment consulted. Polhill and Vidal (1981) considered the genus to include only four taxa in three species. Stergios (1998) recognised 22 species, with 19 of those recorded from Venezuela. Stergios (2001) subsequently added one new species. Lewis (2005b) slightly amended the overall number to a total of 19 species. Stergios (2012) and Stergios and Niño (2012) then added a further two new species. It is probable that the higher numbers of species recorded are an overestimate, and a phylogenetic study and taxonomic revision of the genus are certainly needed. The full geographical range of the genus includes Colombia, southern Venezuela, Guyana, Suriname, Peru, northern Brazil, and Bolivia [13 endemic to Venezuela, five in Venezuela and Brazil (of which four species extend variously to Colombia, Bolivia and Peru), and one restricted to the Guianas] (Fig. 96).

#### Ecology.

Mainly in the Amazon and Orinoco basins in flooded forests, swamp forests (of both black and white-water rivers), on alluvial plains, white sand riverine beaches and embankments, with seeds being mostly water dispersed.

#### Human uses.

The seeds of a few species are used locally to make a flour and medicinally (Lewis 2005b).

#### Etymology.

From Greek, *campso*, *campsis* (= bending, a bend) and *andro*- (= man, anther) referring to the long wavy stamens of especially the first described species.

#### Notes.

*Campsiandra* was originally placed in the informal Peltophorum group of tribe Caesalpinieae sensu Polhill and Vidal (1981), but shown to be more closely related to Dimorphandra group genera in Bruneau et al. (2008), and its position was left unresolved with respect to placement in the broad tribe Caesalpinieae in Lewis (2005b).

#### Taxonomic references.

Lewis (2005b); Polhill and Vidal (1981); Stergios (1996, 1998, 2001, 2012); Stergios and Niño (2012); Stergios and Stergios (1997).

### ﻿ Dinizia

Taxon classificationPlantaeFabalesFabaceae

Ducke, Arch. Jard. Bot. Rio de Janeiro 3: 76. 1922.

Figs 95 , 97

#### Type.

*Diniziaexcelsa* Ducke

#### Description.

Large forest canopy-emergent unarmed trees (Fig. 95F) (some individuals over 60 m), sometimes with buttresses, bark breaking off in large woody plates. **Stipules** subulate, caducous (*D.excelsa*) or unknown (*D.jueirana-facao*). **Leaves** bipinnate, eglandular, the pinnae alternate to subopposite, leaflets alternate. **Inflorescence** a compound spiciform raceme (Fig. 95G). **Flowers** actinomorphic, hermaphrodite or functionally staminate, shortly pedicellate; hypanthium short; calyx valvate in bud, with 5 short, broadly triangular lobes; petals 5, free, imbricate; stamens 10, essentially free, anthers eglandular, dorsifixed; pollen in monads or in gemmate permanent tetrads; a nectarial ring at the hypanthium base; ovary short-stipitate, style apically dilated with a large terminal, tubular to funnel-shaped stigma. **Fruit** coriaceous or woody, indehiscent and marginally compressed or dehiscent along both sutures (Fig. 95E, H). **Seeds** hard, laterally compressed, lacking a pleurogram.

#### Chromosome number.

2*n* = 26 (28) (*D.excelsa*) (Santos et al. 2012).

#### Included species and geographic distribution.

Two species, one widespread in northern and central-western Amazonian Brazil, Guyana, and Suriname (*D.excelsa*), the other narrowly restricted to a small area of Eastern Brazil in Espirito Santo state (*D.jueirana-facao*) (Fig. 97).

#### Ecology.

Non-flooded Amazonian forests (*D.excelsa*), or semi-deciduous Atlantic rainforest (*D.jueirana-facao*).

#### Etymology.

Named by Ducke for his friend José Picanço Diniz, doctor-in-law and philanthropist.

#### Human uses.

The wood of *D.excelsa* is very resistant and has been widely used in civil and naval construction, for railway sleepers, cabinetwork and joinery, as well as for battens, props, beams, girders, posts, stakes, door and window frames, floor-boards, carts, wagons and bridges (Lewis et al. 2017 and other references therein).

#### Notes.

The genus was placed in its own Dinizia group of tribe Mimoseae by Lewis and Elias (1981). Luckow et al. (2003), based on morphological and molecular data, found the genus to be more closely related to non-mimosoid caesalpinioid genera than to any genus in the Mimosoideae. Bruneau et al. (2008) and LPWG (2017) resolved *Dinizia* as occurring firmly outside the Mimoseae clade, close to some genera of the Dimorphandra group of Polhill and Vidal (1981), a placement that is supported by the study of Ringelberg et al. (2022) who resolved the genus as sister to *Campsiandra*. In addition to the striking difference in nodulation (nodulating in *D.jueirana-facao* vs not in *D.excelsa*), the two species also differ importantly in pollen (monads vs tetrads) and in ecology (Atlantic rainforest vs non-flooded Amazonian forests).

#### Taxonomic references.

Ducke (1922); Lewis et al. (2017).

## ﻿﻿11. Tribe Erythrophleeae

Luciano Paganucci de Queiroz^2^, Anne Bruneau^1^

Citation: Queiroz LP, Bruneau A (2024) 11. Tribe Erythrophleeae. In: Bruneau A, Queiroz LP, Ringelberg JJ (Eds) Advances in Legume Systematics 14. Classification of Caesalpinioideae. Part 2: Higher-level classification. PhytoKeys 240: 193–200. https://doi.org/10.3897/phytokeys.240.101716

### ﻿Tribe Erythrophleeae

Taxon classificationPlantaeFabalesFabaceae

Legume Phylogeny Working Group, tribus nov.

urn:lsid:ipni.org:names:77339339-1

**Figure 98.**
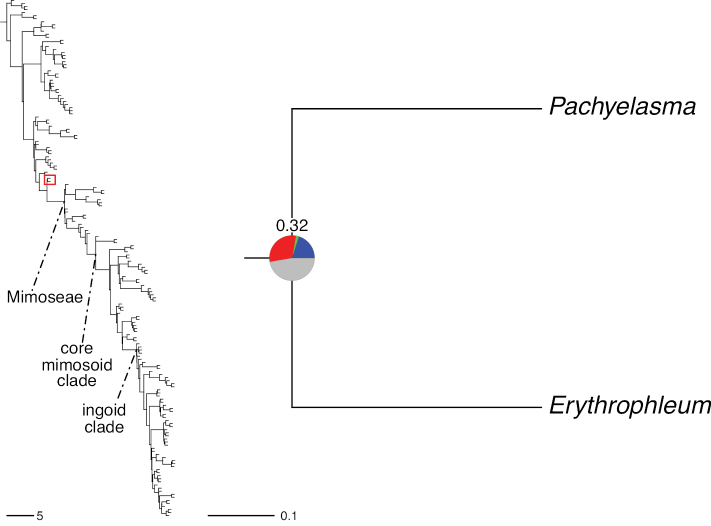
Generic membership and phylogenetic position of tribe Erythrophleeae. For description of phylogeny and support values, see Fig. 6 caption (page 63).

**Figure 99.**
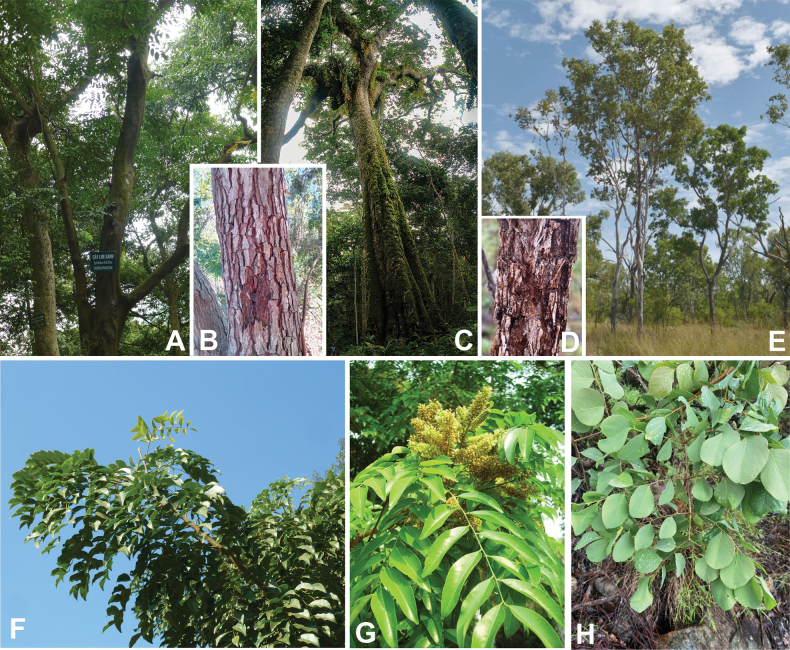
Habit, bark and foliage of Erythrophleeae**A***Erythrophleumfordii* Oliv. at Hùng Temple, Vietnam **B** bark of *Erythrophleumcouminga* Baill. in Madagascar **C***Pachyelasmatessmannii* (Harms) Harms in Congolian rainforest **D, E, H***Erythrophleumchlorostachys* Baill. **D** rugous bark **E** tree in northern Australian savannas **H** foliage **F***Erythrophleumlasianthum* Corbishley foliage **G***Erythrophleumsuaveolens* (Guill. & Perr.) Brenan foliage and terminal inflorescences, Ntumbachusi falls, Zambia. Photo credits **A** Hungda (https://tropical.theferns.info/image.php?id=Erythrophleum+fordii) **B** Solofo Eric Rakotoarisoa, iNaturalist (https://www.inaturalist.org/photos/42581094) **C** Bart Wursten (https://www.flickr.com/photos/zimbart/8212743587/in/photolist-dvJsxn-dvJnwi) **D, E, H** G Mahajan (https://alchetron.com/Erythrophleum-chlorostachys) **F** JMK (https://wikiwand.com/en/Erythrophleum_lasianthum) **G** MG Bingham (https://malawiflora.com/speciesdata/image-display.php?species_id=126540&image_id=8).

**Figure 100.**
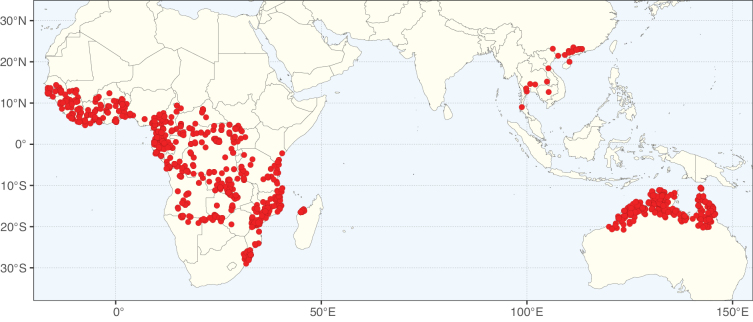
Distribution of *Erythrophleum* based on quality-controlled digitised herbarium records. See Suppl. material 1 for the source of occurrence data.

**Figure 101.**
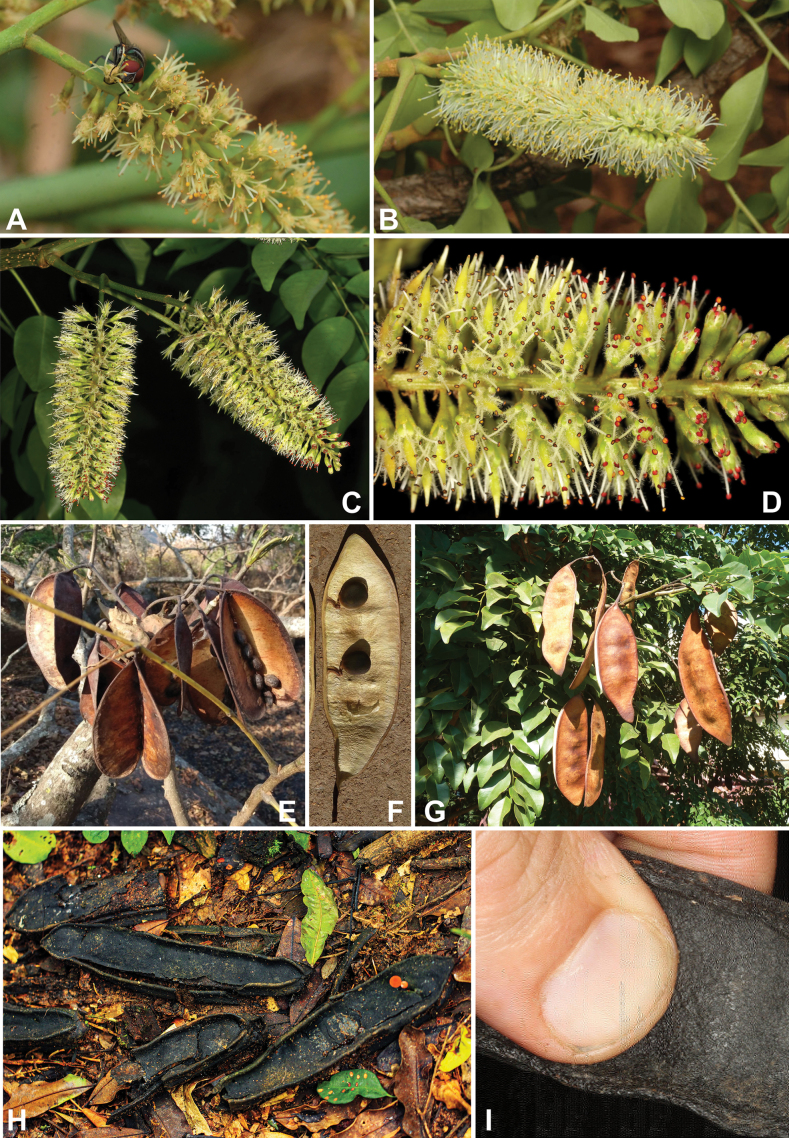
Tribe Erythrophleeae inflorescences, fruits and seeds **A–D** Spicate racemes of species of *Erythrophleum* with small flowers, erect sepals and petals and hairy stamen filaments **A***E.suaveolens* (Guill. & Perr.) Brenan with a visiting fly **B***E.chlorostachys* (F. Muell.) Baill. (Ironwood) **C, D***E.lasianthum* Corbishley **E–I** Fruits and seeds **E** dehisced fruits of *Erythrophleumsuaveolens***F, G** ripe fruits of *Erythrophleumlasianthum***H, I** dispersed fruits of *Pachyelasmatessmannii* (Harms) Harms on the forest floor, showing the flat valves and raised margins. Photo credits: **A** AlkalIn (https://commons.wikimedia.org/wiki/File:Flowers_of_Erythrophleum_suaveolens.jpg) **B** T Harley (https://territorynativeplants.com.au/erythrophleum-chlorostachys-ironwood) **C, D** SAplants **E** Oliver Haumann, iNaturalist (https://www.inaturalist.org/photos/60428121) **F, G** JMK (https://commons.wikimedia.org/wiki/File:Erythrophleum_lasianthum,_loof_en_peule,_Manie_van_der_Schijff_BT,_a.jpg) **H** B Wursten (https://flickr.com/photos/zimbart/8212726705) **I** T Stévart (https://tropicos.org/ImageDownload.aspx?imageid=100336252).

**Figure 102.**
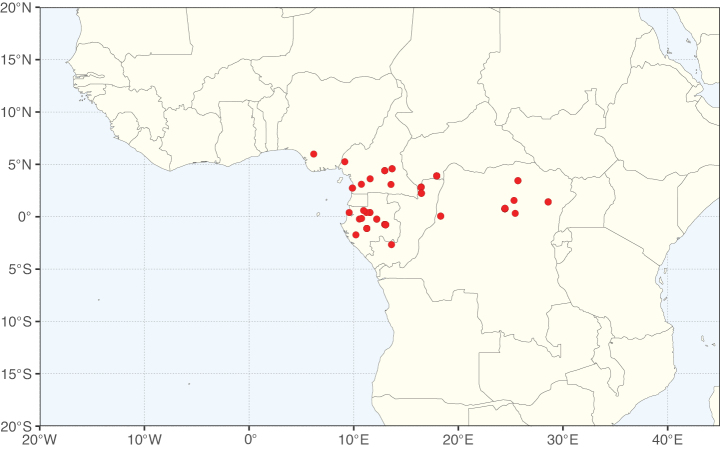
Distribution of *Pachyelasma* based on quality-controlled digitised herbarium records. See Suppl. material 1 for the source of occurrence data.

Figs 98 , 99 , 100 , 101 , 102

#### Diagnosis.

Unarmed trees or treelets with macrophyllidious bipinnate leaves, alternate leaflets, flowers shortly pedicellate, densely packed in elongate spicate racemes, small, regular, with a short cupular hypanthium, sepals and petals ascending, almost erect. Similar to the genera *Adenanthera* L., *Amblygonocarpus* Harms and *Tetrapleura* Benth. (Adenanthera clade, tribe Mimoseae) in habit of unarmed trees with ample bipinnate leaves with alternate leaflets and small pedicellate flowers in spicate racemes, but differing by the ascending perianth giving a closed aspect to the flowers (vs. flowers open because sepals and petals are reflexed backwards) and seeds lacking a pleurogram. Also differentiated from *Adenanthera* by fruits straight or slightly curved with thick woody valves (vs. valves thin coriaceous and twisted after dehiscence) and from *Amblygonocarpus* and *Tetrapleura* by the fruits with flat valves [vs. tetragonal with a median rib (*Amblygonocarpus*) or wing (*Tetrapleura*) on each valve].

#### Type

**(designated here).***Erythrophleum* Afzel. ex R. Br.

#### Included genera

**(2).***Erythrophleum* Afzel. ex R. Br. (12 species), *Pachyelasma* Harms (1).

#### Description.

Unarmed trees or treelets; trunk with rough bark and a reddish sap when cut, brachyblasts absent. **Stipules** inconspicuous, mostly caducous. **Leaves** bipinnate, ample, macrophyllidious, with few pinnae and few leaflets per pinna, leaflets alternate, elliptical to oblong, frequently asymmetrical, pinnately veined. **Inflorescences** spicate racemes clustered in terminal or axillary panicles. **Flowers** perigynous, shortly pedicellate, 5-merous, bisexual, sepals and petals ascending, almost erect, the perianth almost cylindrical; stamens 10, free, the filaments glabrous or pubescent, anthers dehiscing through longitudinal slits; pollen in tricolporate monads; ovary stipitate, pluriovulate, style conical to cylindrical. **Fruit** dehiscent or, rarely, indehiscent, valves stiffly coriaceous or resinous, endocarp not septate nor breaking into one-seeded envelopes. **Seeds** slightly compressed, without pleurogram.

#### Clade-based definition.

The most inclusive crown clade containing *Erythrophleumsuaveolens* (Guill. & Perr.) Brenan and *Pachyelasmatessmannii* (Harms) Harms, but not *Campsiandralaurifolia* Benth., *Dimorphandraconjugata* (Splitg.) Sandwith or *Pentaclethramacrophylla* Benth. (Fig. 98).

#### Distribution.

Tropical Africa (including Madagascar), eastern and south-eastern Asia and Australia.

#### Notes.

The new tribe Erythrophleeae is here proposed to include *Erythrophleum* and *Pachyelasma*, two genera which were previously included in the informal Dimorphandra group of old sense tribe Caesalpinieae (sensu Polhill and Vidal 1981). The two genera have been resolved as part of a grade subtending the mimosoid legumes (tribe Mimoseae) in previous phylogenetic analyses using few molecular markers (e.g., Bruneau et al. 2001, 2008; Luckow et al. 2003; Bouchenak-Khelladi et al. 2010; Marazzi and Sanderson 2010; Manzanilla and Bruneau 2012), but rarely found to group together (Herendeen et al. 2003a). Plastome genomic data also resolved *Erythrophleum* and *Pachyelasma* as successively diverging lineages subtending the Mimoseae (Zhang et al. 2020). However, phylogenomic analyses of low copy nuclear genes have resolved the two genera together in a clade sister to the Mimoseae, but with each genus subtended by a long branch and with a short internode supporting the clade (Koenen et al. 2020a; Ringelberg et al. 2022).

*Erythrophleum* and *Pachyelasma* share a combination of morphological traits only rarely found in non-Mimoseae Caesalpinioideae, such as bipinnate leaves and small pedicellate perigynous flowers clustered in dense spicate racemes. Structural extrafloral nectaries that were characterised as parenchymatous and elevated with a small domed structure with a central pore (Pascal et al. 2000; Marazzi et al. 2019) are found in some species of *Erythrophleum*. Other than being rather cryptic, the presence of these extrafloral nectaries on the leaf rachis, combined with bipinnate leaves, are another morphological similarity with Mimoseae. The two genera also share a reddish sap and very toxic alkaloids and saponins. *Erythrophleum* is reported to be nodulating with fixation threads, as is typical of nodulating non-Mimoseae Caesalpinioideae (Faria et al. 2022; status unknown for *Pachyelasma*).

### ﻿ Erythrophleum

Taxon classificationPlantaeFabalesFabaceae

Afzel. ex R. Br., Denham, Clapperton & Oudney, Narr. Travels Africa: 235. 1826.

Figs 99 , 100 , 101

Erythrophleum Afzel. ex G. Don, Gen. Hist. 2: 424. 1832. Type: Erythrophleumguineense G. Don [= Erythrophleumsuaveolens (Guill. & Perr.) Brenan], nom. superfl.
Fillaea Guill. & Perr., Fl. Seneg. Tent. 1: 242, pl. 55. 1832. Type: Fillaeasuaveolens Guill. & Perr. [≡ Erythrophleumsuaveolens (Guill. & Perr.) Brenan]
Mavia G. Bertol., Mem. Reale Accad. Sci. Ist. Bologna 2: 570. 1850. Type: Maviajudicialis G. Bertol. [= Erythrophleumsuaveolens (Guill. & Perr.) Brenan]
Laboucheria F. Muell., J. Proc. Linn. Soc., Bot. 3: 158. 1859. Type: Laboucheriachlorostachya F. Muell. [≡ Erythrophleumchlorostachys (F. Muell.) Baill.]

#### Type.

*Erythrophleumsuaveolens* (Guill. & Perr.) Brenan

#### Description.

Unarmed trees to 30 m, treelets, rarely shrubs; trunk with rough bark; short shoots absent. **Stipules** very small, caducous. **Leaves** bipinnate; extrafloral nectaries present (7 species) or absent (3), with the secretory surface sunken in a pit capped by a small round pore (Pascal et al. 2000); pinnae 2–7 pairs, opposite or subopposite, articulated with leaf rachis; leaflets (4) 7–17 per pinna, alternate, mostly elliptical or ovate to oblong with an acuminate to acute, rarely rounded apex, petiolulate, pinnately veined, mostly asymmetrical because of slightly displaced midvein; stipels absent. **Inflorescences** densely flowered, elongate spicate racemes, usually clustered in ample terminal panicles; bracts small, caducous; bracteoles absent. **Flowers** small, actinomorphic, bisexual, shortly pedicellate, subsessile, greenish-yellow or white; hypanthium cupular to tubular; sepals 5, joined at the base or to the middle, lobes imbricate, usually open from an early stage; petals 5, free, imbricate, sessile, pubescent, oblanceolate, narrowed towards base; stamens 10, free, more or less equal or alternately longer and shorter, filaments glabrous or pubescent, anthers dehiscing longitudinally; pollen in tricolporate, psilate monads; intrastaminal disk absent; ovary long-stipitate, tomentose or densely pubescent, tapering into a short conical style, stigma minute, punctiform, minutely ciliolate, ovules numerous. **Fruits** two-valved legumes, flattened, straight or slightly curved, oblong or oblong-elliptic, 2–11-seeded, dehiscing through both margins, not internally septate; valves thin, woody or coriaceous, not becoming twisted, mostly smooth, without prominent venation. **Seeds** globose to ellipsoid, only slightly compressed, pleurogram absent.

#### Chromosome number.

2*n* = 24, 28 (Goldblatt 1981b; Okeyo 2006).

#### Included species and geographic distribution.

Twelve species, five in tropical sub-Saharan Africa and Madagascar, four in eastern and south-eastern Asia (south-eastern China, Cambodia, Laos, Vietnam, Thailand, Taiwan) and three in northern Australia (Fig. 100). Introduced in Malaya and Sri Lanka.

#### Ecology.

Tropical lowland wet forests in western Africa and south-eastern Asia, seasonally dry forests, woodlands and savannas in central-southern Africa and Australia.

#### Etymology.

From Greek, *erythros* (= red) and *phloio* (= bark), in reference to the red juice which flows from the trunk when cut.

#### Human uses.

Almost all parts of the plants of species of *Erythrophleum* are highly toxic for humans and livestock, due mostly to alkaloids, which have a *Digitalis*-like action on the heart, and to saponins (Margaret 1959; Ha et al. 2017). Many species are used for therapeutic purposes in various local communities in Africa and Asia (reviewed in Son 2019; Barrett and Barrett 2023), especially for cardiovascular diseases [*E.africanum* (Welw. ex Benth.) Harms, *E.suaveolens*], leishmaniasis (*E.suaveolens*), to invigorate and promote blood circulation (*E.fordii* Oliv.), or for anticonvulsant and anti-inflammatory properties (*E.suaveolens*). The powdered bark of *E.lasianthum* Corbishley is taken as a snuff to relieve headaches, as a remedy for other pains and fever, and to cure lung sickness in cattle.

The crushed bark of some species (*E.lasianthum* and *E.suaveolens*) is used as a fish and rat poison and crushed seeds are used as a component of arrow poison (Lewis 2005b). West African species are known as ‘ordeal trees’ or ‘poison d’épreuve’ because a poisonous concoction made of the bark of some species is used in “Sassywood”, a drink used in a form of trial by ordeal that was in use in West Africa (Leeson and Coyne 2012).

Timber of some species, usually named as ‘Tali’, is used for railway sleepers, boat building and canoes, heavy construction and joinery, firewood, and charcoal (Lewis 2005b). *Erythrophleumchlorostachys* (including *E.pubescens* R.L. Barrett & M.D. Barrett and *E.arenarium* R.L. Barrett & M.D. Barrett) is one of the densest native timbers of Australia (Barrett and Barrett 2023). Several species of *Erythrophleum* are threatened by over-exploitation for medicinal or timber uses (Barrett and Barrett 2023).

#### Notes.

Together the twelve species of *Erythrophleum* comprise a morphologically cohesive genus, diagnosed by the combination of macrophyllidious bipinnate leaves, pedicellate flowers with straight and almost erect sepals and petals, and a long stipitate ovary tapering to a short conical style. The genus can be differentiated from *Pachyelasma* by the length of the ovary stipe (long in *Erythrophleum* vs. short in *Pachyelasma*), flower colour (green to greenish-yellow vs. reddish) and fruits (thin woody or leathery valves and margins not thick vs. resinous valves and thick, raised margins).

There is some controversy regarding the date and place of publication and the type species of the genus. Don (1832) provided an elaborate description of the genus and proposed the new species *E.guineense* G. Don, and this has sometimes been accepted as the original publication. However, the earlier publication by Brown (1826), which included only a very brief description of the genus characters, is nonetheless considered sufficient to make the publication valid, and this is adopted here, following Barrett and Barrett (2023). *Erythrophleum* Afzel. ex G. Don is thus considered a later homonym and therefore invalidly published.

Seven of the twelve species have been included in various molecular phylogenetic and genetic studies, all of which support the monophyly of the genus as sampled (Bruneau et al. 2001, 2008; Herendeen et al. 2003a; Duminil et al. 2013; LPWG 2017; Koenen et al. 2020a; Zhang et al. 2020; Ringelberg et al. 2022; Barrett and Barrett 2023).

Extrafloral nectaries were first reported in the 1980’s and they are now known to be present on the leaf rachis of at least seven of the twelve species (Pascal et al. 2000). Although they have a simpler structure than extrafloral nectaries of the Mimoseae, they have a similar histological structure and probably play a similar role in mediating ant-plant defense systems (Pascal et al. 2000; Marazzi et al. 2019). Pollen grains of *Erythrophleum* are reported to be the smallest found in Caesalpinioideae, together with those of *Burkea* (Banks and Lewis 2009). Small insects such as beetles, bees and wasps are the main visitors of flowers of *E.fordii* Oliv. and flies were reported on flowers of *E.suaveolens* (Zhu et al. 2013). Seeds in fresh pods are surrounded by a mucilage that might have a nutritive value; this is supported by the finding of seeds in the faeces of primates, including gorillas (Duminil et al. 2016).

#### Taxonomic references.

Aubréville (1968); Barrett and Barrett (2023); Brenan (1967, with illustration); Larsen et al. (1980); Ross (1977).

### ﻿ Pachyelasma

Taxon classificationPlantaeFabalesFabaceae

Harms, Bot. Jahrb. Syst. 49: 428. 1913.

Figs 99 , 101 , 102

#### Type.

*Pachyelasmatessmannii* (Harms) Harms [≡ *Stachyothyrsustessmannii* Harms]

#### Description.

Unarmed trees, frequently emergent above forest canopies, to 60 m and 2.5 m diameter (Fig. 99C), frequently buttressed; bark greyish and rugose, thick, peeling off in irregular flakes; short shoots absent. **Stipules** inconspicuous, caducous. **Leaves** bipinnate, petiole and leaf rachis cylindrical; pinnae 2–3 pairs, opposite or, rarely, subopposite, articulated with leaf rachis; leaflets 9–14 per pinna, alternate, oblong or oblong-lanceolate, coriaceous, pinnately veined, shortly petiolulate; stipels not seen; extrafloral nectaries absent. **Inflorescences** densely flowered spicate racemes, clustered in short axillary panicles, the peduncle short or absent and a short rachis. **Flowers** small (ca. 5 mm long), wine red with a yellowish base, actinomorphic, bisexual or unisexual (staminate), pedicellate; hypanthium short, cupuliform; sepals 5, free; petals 5, free, imbricate, obovate, margins ciliate; stamens 10, free, equally long, anthers dehiscing through longitudinal slits, dorsally attached to a massive connective; pollen in isopolar, trizonocolporate monads, exine perforate to finely rugulate; intrastaminal disk attached to the hypanthium surface; ovary shortly stipitate, glabrous, ovules numerous (15–20), style cylindrical, stigma punctiform. **Fruit** indehiscent or late dehiscent, with a thick body, flat compressed, straight or slightly curved, oblong or oblong-lanceolate, each margin provided with two thick erect ribs; valves thick, smooth, the mesocarp gelatinous-resinous, the endocarp internally septate into 10–15 one-seeded envelopes. **Seeds** ellipsoid, only slightly compressed, testa bright, pleurogram absent.

#### Chromosome number.

Unknown.

#### Included species and geographic distribution.

Monospecific (*P.tessmannii*), distributed in west tropical Africa (Nigeria, Cameroon, Central African Republic, Equatorial Guinea, Gabon, Congo, Democratic Republic of Congo; Fig. 102).

#### Ecology.

Tropical lowlands rainforests of the Guinean and Congolian ecoregions.

#### Etymology.

From the Greek, *pachy*- (= thick) and *elasmos* (= plate), in reference to the thick pod.

#### Human uses.

*Pachyelasmatessmannii*, locally known as Mekogho and Mundumbula in Gabon, is used medicinally in various capacities. Fruits are used in traditional folk medicine to cure diarrhoea and abdominal pain (Betti 2002). The root bark exhibits potent molluscicidal properties against the schistosomiasis snail *Biomphalariaglabrata* (Say, 1818) (Nihei et al. 2005). Crushed pods and bark are used as an abortifacient and fish poison (Mouele 2022).

#### Notes.

*Pachyelasmatessmannii* is a dominant tree in West African rainforests, where it is one of the tallest trees, frequently emergent beyond the forest canopy. Its flowers are described as having a very unpleasant odour at night, which can be detected even from a distance of 300 m (*Breteler 1026*, P).

#### Taxonomic references.

Harms (1913) with illustration.

## ﻿﻿12. Tribe Mimoseae

Luciano Paganucci de Queiroz^2^, Erik J. M. Koenen^26^, Colin E. Hughes^3^, Melissa Luckow^29^, Gwilym P. Lewis^10^, Jens J. Ringelberg^3,4^, Anne Bruneau^1^

Citation: Queiroz LP, Koenen EJM, Hughes CE, Luckow M, Lewis GP, Ringelberg JJ, Bruneau A (2024) 9. Tribe Mimoseae. In: Bruneau A, Queiroz LP, Ringelberg JJ (Eds) Advances in Legume Systematics 14. Classification of Caesalpinioideae. Part 2: Higher-level classification. PhytoKeys 240: 201–206. https://doi.org/10.3897/phytokeys.240.101716

### ﻿Tribe Mimoseae

Taxon classificationPlantaeFabalesFabaceae

Bronn, Form. Pl. Legumin.: 78, 127, 130. 1822.

Mimosaceae R. Br., in M. Flinders, Voy. Terra Austral. 2: 551. 1814, nom. cons. Type: Mimosa L.
Mimosoideae DC., Prodr. [A.P. de Candolle] 2: 424. 1825. Type: Mimosa L.
Acacieae Dumort., Anal. Fam. Pl.: 40. 1829. Type: Acacia Mill., nom. cons.
Acaciinae Wight & Arn., Prodr. Fl. Ind. Orient.: 267. 1834. Type: Acacia Mill., nom. cons.
Parkiinae Wight & Arn., Prodr. Fl. Ind. Orient.: 279. 1834. Type: Parkia R. Br.
Acaciaceae E. Mey., Comm. Pl. Afr. Austr. 1: 164. 1836. Type: Acacia Mill., nom. cons.
Desmanthinae Benth., J. Bot. (Hooker) 2: 128. 1840. Type: Desmanthus Willd.
Parkieae Endl., Gen. Pl.: 1323. 1840. Type: Parkia R. Br.
Adenantherinae Benth., J. Bot. (Hooker) 4: 331. 1841. Type: Adenanthera L.
Mimosineae J. Presl, Nowočeská Bibl. [Wšobecný Rostl.] 7: 346: 421. 1846. Type: Mimosa L.
Adenanthereae Benth. & Hook.f., Gen. Pl. 1: 437. 1865. Type: Adenanthera L.
Ingeae Benth. & Hook.f., Gen. Pl. 1: 437. 1865. Type: Inga Mill.
Piptadenieae Benth., Trans. Linn. Soc. London 30: 343, 358. 1875. Type: Piptadenia Benth.
Desmantheae Kuntze, in von Post & Kuntze, Lex. Gen. Phan.: 646. 1903. Type: Desmanthus Willd.
Mimozygantheae Burkart, Darwiniana 3: 447. 1939. Type: Mimozyganthus Burkart
Albizieae Nakai, Chosakuronbun Mokuroku [Ord. Fam. Trib. Nov.]: 251. 1943. Type: Albizia Durazz.
Affonseeae Nakai, Chosakuronbun Mokuroku [Ord. Fam. Trib. Nov.]: 251. 1943. Type: Affonsea A. St.-Hil. [= Inga Mill.]

#### Type.

*Mimosa* L.

#### Description.

Trees, shrubs, lianas, suffruticose or herbs, occasionally aquatic; unarmed or armed with prickles, spines or thorns. **Stipules** lateral and free or absent. **Leaves** bipinnate, less frequently paripinnate or modified into phyllodes (many *Acacia*, some *Mimosa*), rarely absent; pinnae and leaflets mostly opposite, rarely alternate; paraphyllidia (reduced basal leaflet pair on the pinnae) present or absent; specialised extrafloral nectaries often present on the petiole and/or on the primary and secondary rachides. **Inflorescence** globose, ellipsoid, umbelliform or corymbiform capitula, spikes or spiciform racemes; arising singly, paired or many from axillary fascicles, more frequently clustered in diversely arranged synflorescences. **Flowers** bisexual or frequently bisexual flowers combined with unisexual and/or sterile flowers in heteromorphic inflorescences, radially symmetrical; hypanthium mostly lacking; sepals and petals (3) 5 (6–8), mostly fused, sepals valvate in bud, rarely imbricate (*Mimozyganthus*, *Parkia*, *Pentaclethra*), petal aestivation valvate, rarely imbricate (*Chidlowia*, *Sympetalandra*), frequently the base of petals and stamens joined into a tube (stemonozone); stamens diplostemonous, haplostemonous or polystemonous, sometimes modified into showy staminodia, free or the filaments fused, anthers basifixed or dorsifixed, dehiscing via longitudinal slits, often with a stipitate or sessile apical gland; pollen commonly in tetrads, bitetrads or polyads, rarely in monads; gynoecium uni- or rarely polycarpellate, 1–many ovulate. **Fruits** 1–many-seeded, indehiscent or dehiscent along one or both sutures, often explosively or elastically dehiscent, also often lomentum or craspedium, the endocarp indistinct or separate and fragmented into 1-seeded envelopes. **Seeds** usually with an open (U-shaped) or closed (O-shaped) pleurogram on both faces, sometimes with a fleshy aril or sarcotesta; sometimes winged, hilum usually apical, lens usually inconspicuous; embryo straight. Root nodules present, indeterminate, and always symbiosome-type, or absent (at least 7 genera).

#### Included genera

**(100).***Abarema* Pittier (2 species), *Acacia* Mill. (1082), *Acaciella* Britton & Rose (15), *Adenanthera* L. (12), *Adenopodia* C. Presl (7), *Afrocalliandra* E.R. Souza & L.P. Queiroz (2), *Alantsilodendron* Villiers (11), *Albizia* Durazz. (ca. 90), *Amblygonocarpus* Harms (1), *Anadenanthera* Speg. (2–4), *Anonychium* (Benth.) Schweinf. (1), *Archidendron* F. Muell. (ca. 120), *Archidendropsis* I.C. Nielsen (11), *Aubrevillea* Pellegr. (2), *Blanchetiodendron* Barneby & J.W. Grimes (1), *Boliviadendron* E.R. Souza & C.E. Hughes (1), *Calliandra* Benth. (140), *Calliandropsis* H.M. Hern. & P. Guinet (1), *Calpocalyx* Harms (11), *Cedrelinga* Ducke (1), *Chidlowia* Hoyle (1), *Chloroleucon* (Benth.) Britton & Rose (10), *Cojoba* Britton & Rose (13–19), *Cylicodiscus* Harms (1), *Desmanthus* Willd. (23), *Dichrostachys* (DC.) Wight & Arn. (13–14), *Ebenopsis* Britton & Rose (3), *Entada* Adans. (40), *Enterolobium* Mart. (8), *Faidherbia* A. Chev. (1), *Falcataria* (I.C. Nielsen) Barneby & J.W. Grimes (3), *Fillaeopsis* Harms (1), *Gagnebina* Neck. ex DC. (7), *Gretheria* R. Duno & Torke (2), *Gwilymia* A.G. Lima, Paula-Souza & Scalon (7), *Havardia* Small (3), *Heliodendron* Gill.K. Br. & Bayly (3), *Hesperalbizia* Barneby & J.W. Grimes (1), *Hydrochorea* Barneby & J.W. Grimes (10), *Indopiptadenia* Brenan (1), *Inga* Mill. (ca. 300), *Jupunba* Britton & Rose (37), *Kanaloa* Lorence & K.R. Wood (1), *Lachesiodendron* P.G. Ribeiro, L.P. Queiroz & Luckow (1), *Lemurodendron* Villiers & Guinet (1), *Leucaena* Benth. (24), *Leucochloron* Barneby & J.W. Grimes (4), *Lysiloma* Benth. (8), *Macrosamanea* Britton & Rose ex Britton & Killip (12), *Mariosousa* Seigler & Ebinger (14), *Marlimorimia* L.P. Queiroz, L.M. Borges, Marc.F. Simon & P.G. Ribeiro (6), *Mezcala* C.E. Hughes & J.L. Contr. (1), *Microlobius* C. Presl (1), *Mimosa* L. (615), *Mimozyganthus* Burkart (1), *Naiadendron* A.G. Lima, Paula-Souza & Scalon (1), *Neltuma* Raf. (30), *Neptunia* Lour. (22), *Newtonia* Baill. (11), *Osodendron* E.J.M. Koenen (3), *Painteria* Britton & Rose (2), *Parapiptadenia* Brenan (6), *Pararchidendron* I.C. Nielsen (1), *Parasenegalia* Seigler & Ebinger (11), *Paraserianthes* I.C. Nielsen (1), *Parkia* R. Br. (ca. 35), *Pentaclethra* Benth. (3), *Piptadenia* Benth. (28), *Piptadeniastrum* Brenan (1), *Piptadeniopsis* Burkart (1), *Pithecellobium* Mart. (19), *Pityrocarpa* (Benth.) Britton & Rose (7), *Plathymenia* Benth. (1), *Prosopidastrum* Burkart (ca. 6), *Prosopis* L. (3), *Pseudalbizzia* Britton & Rose (17), *Pseudoprosopis* Harms (7), *Pseudosamanea* Harms (3), *Pseudosenegalia* Seigler & Ebinger (2), *Punjuba* Britton & Rose (5), *Ricoa* R. Duno & Torke (1), *Robrichia* (Barneby & J.W. Grimes) A.R.M. Luz & E.R. Souza (3), *Samanea* (Benth.) Merr. (3), *Sanjappa* E.R. Souza & M.V. Krishnaraj (1), *Schleinitzia* Warb. ex J.C. Willis (4), *Senegalia* Raf. (219), *Serianthes* Benth. (18), *Sphinga* Barneby & J.W. Grimes (3), *Strombocarpa* Engelm. & A. Gray (10), *Stryphnodendron* Mart. (28), *Sympetalandra* Stapf (5), *Tetrapleura* Benth. (2), *Thailentadopsis* Kosterm. (3), *Vachellia* Wight & Arn. (164), *Viguieranthus* Villiers (18), *Wallaceodendron* Koord. (1), *Xerocladia* Harv. (1), *Xylia* Benth. (9), *Zapoteca* H.M. Hern. (22), *Zygia* P. Browne (ca. 60).

#### Distribution.

Pantropical, with a few species extending marginally into warm temperate regions in North America and Asia, and extratropical South America, southern Africa and Australia.

#### Clade-based definition.

The most inclusive crown clade containing *Mimosasensitiva* L. and *Pentaclethramacrophylla* Benth., but not *Pachyelasmatessmannii* (Harms) Harms, *Dimorphandraconjugata* (Splitg.) Sandwith or *Delonixdecaryi* (R. Vig.) Capuron (Fig. 5).

#### Notes.

Tribe Mimoseae as circumscribed here broadly coincides with the limits of the old sense subfamily Mimosoideae as adopted in several classical works (e.g., Bentham 1865; Taubert 1894; Hutchinson 1964; Polhill and Raven 1981; Lewis et al. 2005). The Mimosoideae then comprised a morphologically distinct subfamily defined by a syndrome of morphological traits including bipinnate leaves mostly with specialised extrafloral nectaries, flowers relatively small usually packed in dense inflorescences, corolla with valvate aestivation, the relatively long and showy stamens as the most conspicuous part of the flowers, and seeds usually with a pleurogram. Despite having scattered exceptions to almost all of these traits, it was relatively easy to recognise species as being members of the subfamily (here treated as a tribe).

Phylogenetic studies have since shown that most of the genera included in the Mimosoideae comprise a monophyletic group, but nested in a paraphyletic old-sense subfamily Caesalpinioideae (LPWG 2013, 2017). The morphological links between the two then accepted subfamilies were exemplified by a series of mimosoid-like genera with bipinnate leaves and small flowers clustered in dense spicate inflorescence, such as *Dimorphandra* Schott and *Erythrophleum* Afzel. ex R. Br., then classified in the Dimorphandra group of tribe Caesalpinieae (Caesalpinioideae; Polhill and Vidal 1981). This morphological transition was also observed in the genus *Dinizia*, then placed in tribe Mimoseae of subfamily Mimosoideae, but which has the imbricate ascending petal aestivation typical of the non-mimosoid Caesalpinioideae.

When revising the subfamilial classification for the Leguminosae, the Legume Phylogeny Working Group (LPWG 2013) acknowledged that one of the central problems was how to deal with the large clade that included several (old-sense) Caesalpinioideae lineages and which had the Mimosoideae nested within it. The proposed solution was to subsume subfamily Mimosoideae into a re-circumscribed subfamily Caesalpinioideae that recognised the mimosoid clade in an integrated clade-based phylogenetic classification system (LPWG 2017). This option was considered more likely to remain stable through time and is the classification system proposed here, in which, within subfamily Caesalpinioideae, a tribal rank is formally ascribed to the entire mimosoid clade (sensu LPWG 2017). Tribe Mimoseae, as circumscribed here, thus broadly corresponds to the old sense subfamily Mimosoideae with three minor changes in generic attribution. The genus *Dinizia*, once placed in tribe Mimoseae (Lewis and Elias 1981), has been resolved outside of the mimosoid clade in all phylogenetic analyses (Luckow et al. 2000, 2003; Wojciechowski et al. 2004; Bruneau et al. 2008; LPWG 2017), but shown only recently to group with the genus *Campsiandra* (tribe Campsiandreae, page 187) based on phylogenomic data (Zhang et al. 2020; Ringelberg et al. 2022). *Sympetalandra* and *Chidlowia*, classified in tribe Caesalpinieae by Polhill and Vidal (1981), are now clearly supported as members of Mimoseae, even though their respective positions within the tribe are not well resolved. Since LPWG (2017) was published, 19 genera have been newly described or re-instated and four have been reduced into synonymy based on newly available phylogenetic data. Recent phylogenies suggest that nine genera (*Alantsilodendron*, *Archidendron*, *Calliandra*^1^, *Calpocalyx*, *Dichrostachys*, *Parasenegalia*, *Senegalia*, *Xylia*, *Zygia*) are non-monophyletic and require taxonomic revision to recognise only monophyletic genera (Ringelberg et al. 2022). As newly circumscribed, tribe Mimoseae currently includes 100 genera and ca. 3510 species.

In Advances in Legume Systematics Part 1, five tribes were recognised in subfamily Mimosoideae: Parkieae, Mimoseae, Mimozygantheae, Acacieae and Ingeae (Elias 1981a). The small tribe Parkieae was shown to be non-monophyletic, with both genera *Parkia* and *Penthaclethra*, as well as the monospecific tribe Mimozygantheae, found to be nested in different positions within Mimoseae (Luckow et al. 2003, 2005). Similarly, the two large tribes Acacieae and Ingeae (Elias 1981a; Lewis 2005c; Lewis and Rico Arce 2005), which grouped the polystemonous mimosoid legumes, have also been shown to be non-monophyletic in several phylogenetic analyses (e.g., Miller et al. 2003; Brown et al. 2008; Bouchenak-Khelladi et al. 2010). Genera of tribe Ingeae were grouped in five informal alliances by Barneby and Grimes (1996), a system that was later elaborated to six alliances by Lewis and Rico Arce (2005), but which have all also been shown to be non-monophyletic except one. The recognition at the generic level of isolated lineages and segregates of *Pithecellobium* initiated by Nielsen (1981a) and Barneby and Grimes (1996, 1997), and pursued in Advances in Legume Systematics 14, Part 1 (Hughes et al. 2022a), has resolved many issues of generic non-monophyly. Even though the classification of Mimosoideae has been known to be unsatisfactory for the last two decades, lack of support and conflicting hypotheses of relationships between studies using different molecular markers and taxonomic sampling (e.g., Luckow et al. 2003, 2007; Miller et al. 2003; Brown et al. 2008; Bouchenak-Khelladi et al. 2010; LPWG 2017) meant that no new taxonomic arrangement could be proposed. The phylogenomic analyses of Koenen et al. (2020a), subsequently confirmed by Ringelberg et al. (2022) with broader taxon sampling, have enhanced resolution, prompting recognition of two nested higher-level clades subtended by relatively long internodes. The core mimosoid clade groups the majority of the Mimoseae, including all of the larger genera, and almost all of the armed mimosoids (genera and species with stipular spines, spinescent shoots, and/or prickles) (Koenen et al. 2020a). The ingoid clade includes all genera of tribes Ingeae and Acacieae (sensu Elias 1981a; Lewis 2005c; Lewis and Rico Arce 2005), except *Vachellia*, and thus recognises as a clade all genera with polystemonous flowers (except *Vachellia*) and a synandrous androecium (Koenen et al. 2020a), although neither of these characters are universal within the clade. However, relationships amongst the lineages of the ingoid clade remain difficult to resolve even with large phylogenomic datasets, likely the consequence of rapid speciation leading to low phylogenetic signal and a putative hard polytomy comprising six or seven lineages (Koenen et al. 2020a).

Despite this putative hard polytomy along the backbone of the ingoid clade, the phylogenomic backbone of the Mimoseae of Koenen et al. (2020a) and Ringelberg et al. (2022) (Fig. 5) provides a solid framework for recognizing 17 lower-level clades that together include 86 of the 100 genera in tribe Mimoseae. Two of these clades were not recognised by Koenen et al. (2020a) nor Ringelberg et al. (2022), and are added here following disintegration of the genus *Prosopis* proposed by Hughes et al. (2022b). The phylogenetic positions of five genera were poorly or not resolved in terms of their closest relatives: *Chidlowia* and *Sympetalandra* with respect to the Adenanthera clade and the remainder of Mimoseae; *Cylicodiscus* relative to the Prosopis clade and the remainder of the core mimosoids; and *Cedrelinga* and *Pseudosamanea* with respect to their positions in the ingoid clade. A sixth taxon, *Lachesiodendron*, is resolved as sister to a big clade that includes the Stryphnodendron, Mimosa and ingoid clades (60 genera) and is considered here as an isolated lineage. In addition, eight genera are placed in sequential order in two grades rather than being resolved in one of the 17 clades. Four genera are part of a grade that subtends the core mimosoid clade and is here informally designated as the Newtonia grade and four genera, constituting the earliest-diverging lineages in the ingoid clade, are part of a grade that is here informally referred to as the Senegalia grade.

The alternative solution for classification of this group, that of recognising multiple tribes within the mimosoid clade, is untenable given the imbalanced, “ladder-like” phylogenomic backbone of the mimosoid legumes (Fig. 5) (Koenen et al. 2020a; Ringelberg et al. 2022), with eight genera forming grades subtending large clades and several genera with unresolved or phylogenetically isolated positions. This alternative solution would result in a system of more than 30 tribes, of which more than one third would be monogeneric and many others would comprise only two to five genera, which would be impractical and cumbersome and lead to an unnecessary proliferation of supra-generic Linnean names. We thus chose to recognise the entire mimosoid clade as one tribe, the Mimoseae, with a circumscription roughly equivalent to the old-sense subfamily Mimosoideae. The following treatments provide formal descriptions and information for the 17 well-supported lower-level clades, each formally defined and named after a characteristic genus of the clade; sequentially ordered single genus lineages in two grades, these informally labelled also by a genus characteristic of the grade; and six monogeneric lineages whose phylogenetic placements are either unresolved or isolated in Mimoseae.

Thus, in the following taxonomic arrangement, 25 treatments are presented for tribe Mimoseae (the numbers between brackets refer to the number of genera):

Tribe Mimoseae

13. Adenanthera clade (7 genera)

14. *Sympetalandra* (1)

15. *Chidlowia* (1)

16. Entada clade (3)

17. Newtonia grade (4)

**Core mimosoid clade**

18. *Cylicodiscus* (1)

19. Prosopis clade (2)

20. Neltuma clade (3)

21. Dichrostachys clade (14)

22. Parkia clade (3)

23. *Lachesiodendron* (1)

24. Stryphnodendron clade (7)

25. Mimosa clade (3)

**Ingoid clade**

26. Senegalia grade (4)

27. Calliandra clade (3)

28. Zapoteca clade (5)

29. Cojoba clade (3)

30. Pithecellobium clade (7)

31. Archidendron clade (9)

32. *Cedrelinga* (1)

33. *Pseudosamanea* (1)

34. Jupunba clade (4)

35. Samanea clade (2)

36. Albizia clade (3)

37. Inga clade (8)

## ﻿﻿13. Adenanthera clade

Melissa Luckow^29^

Citation: Luckow M (2024) 13. Adenanthera clade. In: Bruneau A, Queiroz LP, Ringelberg JJ (Eds) Advances in Legume Systematics 14. Classification of Caesalpinioideae. Part 2: Higher-level classification. PhytoKeys 240: 207–224. https://doi.org/10.3897/phytokeys.240.101716

**Adenanthera clade**

Figs 103–113

**Included genera (7).***Amblygonocarpus* Harms (1 species), *Adenanthera* L. (12), *Calpocalyx* Harms (11), *Pentaclethra* Benth. (3), *Pseudoprosopis* Harms (7), *Tetrapleura* Benth. (2), *Xylia* Benth. (9).

**Description.** Predominantly large trees, occasionally lianas and small trees, unarmed except for uncinate lignified tendrils in climbing lianas of *Pseudoprosopis*; brachyblasts absent. **Stipules** small, linear to triangular, caducous. **Leaves** bipinnate, macrophyllous to microphyllous; pinnae opposite, subopposite, or alternate, few to many pairs per leaf; foliar nectaries absent except in *Xylia* and *Calpocalyx*, where they are present between the proximal pair of pinnae and sometimes between additional pinnae pairs, mounded or sunken into the petiole; leaflets opposite, subopposite, or alternate, usually petiolulate but sessile in *Pentaclethra*. **Inflorescence** usually racemose but sometimes spicate (*Pentaclethra*, *Calpocalyx*), and capitate or umbellate in *Xylia*; the primary inflorescences either axillary and solitary or paired, and immersed in the foliage, but more often grouped into terminal paniculiform secondary inflorescences exserted from the foliage; pedicels sometimes jointed where they join the calyx and remaining as peg-like structures when the flowers abscise. **Flowers** 5-merous, usually all hermaphrodite and appearing bisexual, functionally pistillate flowers reported in some species of *Calpocalyx*; hypanthium absent; sepals valvate in bud, imbricate in one genus (*Pentaclethra*), connate, often attenuate proximally and forming a pseudopedicel; petals valvate in bud, free or loosely joined at base, sometimes adnate to the stamens and forming a stemonozone; stamens 10, free, anthers bearing stipitate glands (absent in *Amblygonocarpus*); pollen usually in 8–32-grained calymmate polyads (tricolporate monads in *Pentaclethra*); ovary sessile or stipitate, stigma porate. **Fruits** variable, the valves woody and explosively dehiscent through both sutures in *Pentaclethra*, *Xylia*, *Pseudoprosopis*, and *Calpocalyx*, coriaceous to chartaceous and the valves curling post-dehiscence in *Adenanthera*; indehiscent in *Tetrapleura* and *Amblygonocarpus*. **Seeds** unwinged, the testa hard and bearing a pleurogram in most genera, recalcitrant with a papery testa and lacking a pleurogram in *Pentaclethra* and *Calpocalyx*.

**Distribution.** Humid forests of Africa and Asia, with only one species (*Pentaclethramacroloba* (Willd.) Kuntze) in the New World.

**Figure 103.**
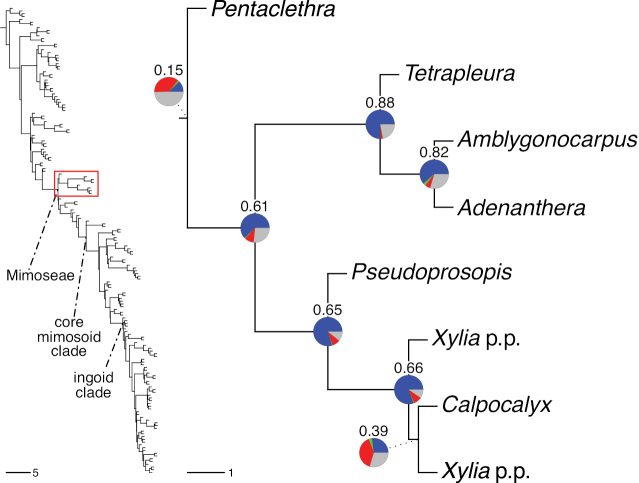
Generic relationships in the Adenanthera clade (tribe Mimoseae). For description of phylogeny and support values, see Fig. 6 caption (page 63).

**Notes.** The Adenanthera clade includes all genera from the Adenanthera group of Luckow (2005) plus its sister group *Pentaclethra*. The clade, named the Xylia clade by Koenen et al. (2020a), is resolved as sister to the remainder of Mimoseae, but with poorly resolved relationships with *Chidlowia* Hoyle, *Sympetalandra* Stapf and the Entada clade (Koenen et al. 2020a; Ringelberg et al. 2022). The clade includes two distinctive subclades, one comprising *Adenanthera*, *Tetrapleura*, and *Amblygonocarpus*, and the other including *Xylia*, *Pseudoprosopis*, and *Calpocalyx* (Koenen et al. 2020a; Fig. 103), both of which are well characterised morphologically.

**Clade-based definition.** The most inclusive crown clade containing the most recent common ancestor of *Pentaclethramacrophylla* Benth. and *Xyliatorreana* Brenan, but not *Sympetalandraschmutzii* Steenis, *Entadapervillei* (Vatke) R. Vig. or *Pachyelsamatessmannii* (Harms) Harms (Fig. 103).

The transitional nature of the Adenanthera clade is evidenced by the presence of characters that are very unusual among other members of the Mimoseae such as imbricate sepals and alternate leaflets. Flowers of *Pentaclethra* are distinct from other genera in the group having imbricate sepals, five staminodia alternating with fertile stamens within each flower, and a unique hooded anther gland unlike any other in Mimoseae. What *Pentaclethra* shares with most other members of the Adenanthera clade are woody, explosively dehiscent pods, another character uncommon outside this clade, and which may be the ancestral fruit state of the Adenanthera clade.

The relationship between *Tetrapleura* and *Amblygonocarpus* is morphologically strong. The fruits are quite similar, being indehiscent and four-angled in cross-section, with the angles elaborated into wings in *Tetrapleura* (Fig. 106B, E). Both share petiolate, alternate leaflets and often pinnae with *Adenanthera*, and the three genera are difficult to distinguish without flowers or fruits. *Amblygonocarpus* is the sole genus in the *Adenanthera* clade to consistently lack anther glands. Fruits and seeds of *Adenanthera* are quite distinct from the woody valves found in other members of the clade; the valves are coriaceous to chartaceous and coil after dehiscence to expose the persistent bright red or red and black seeds (Fig. 106A). It is likely that these taxa also share a unique anther gland anatomy, along with *Xylia* and *Calpocalyx*, although more thorough sampling is needed to confirm this (Luckow and Grimes 1997). Species of this clade are distributed in Africa, Madagascar, and Asia.

**Figure 104.**
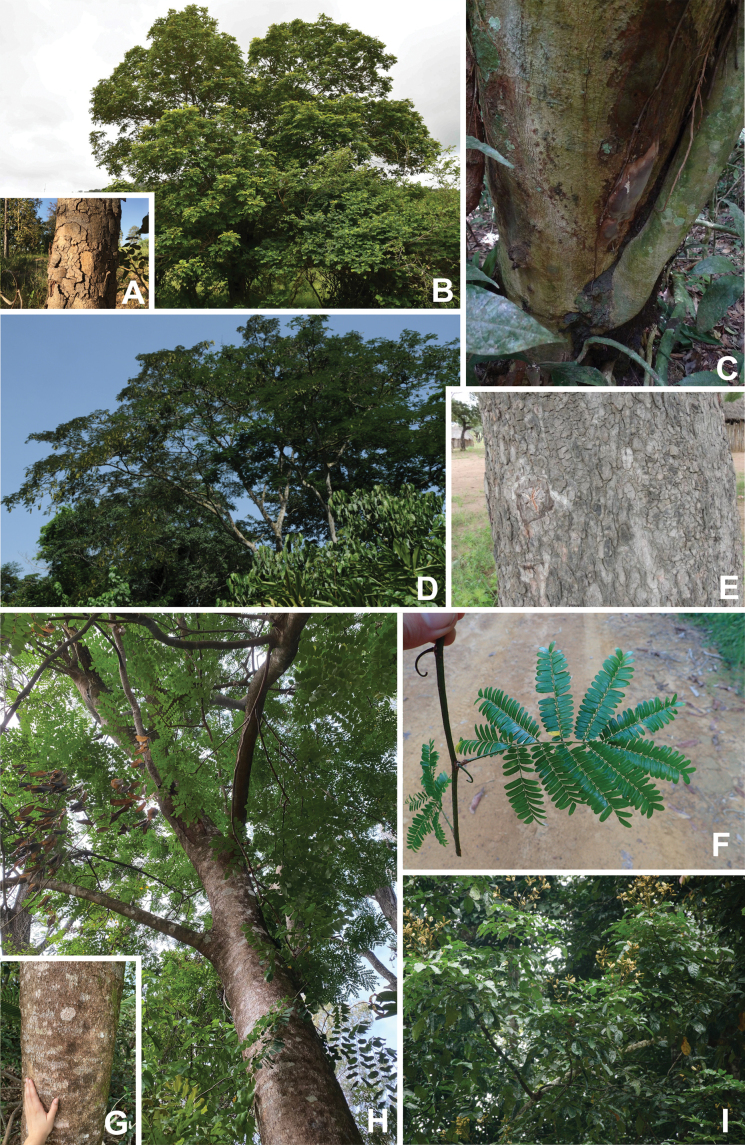
Habit and growth form in the Adenanthera clade **A**Xyliaxylocarpavar.kerrii (Craib & Hutch.) I.C. Nielsen scaly bark **B***Xyliatorreana* Brenan canopy **C***Pentaclethramacroloba* (Willd.) Kuntze trunk showing buttresses **D***Tetrapleuratetraptera* (Schumach. & Thonn.) Taub. canopy (*Harris 9659*) **E***Amblygonocarpusandongensis* (Welw. ex Oliv.) Exell & Torre pale grey, scaly bark (*Catarino 2067*) **F***Pseudoprosopisgilletii* (De Wild.) Villiers liana with leaves and lignified tendrils (*Texier 1558*) **G***Adenantherapavonina* L. thin scaly bark **H** habit **I***Amblygonocarpusandongensis* (Welw. ex Oliv.) Exell & Torre growing in savanna (*Coates Palgrave M806*). Photo credits **A** Dechaphaetkrathok **B** F du Randt **C** D DeMelo **D** DJ Harris **E** L Catarino **F** N Texier **G, H** Shelbyfarmer **I** M Coates Palgrave.

**Figure 105.**
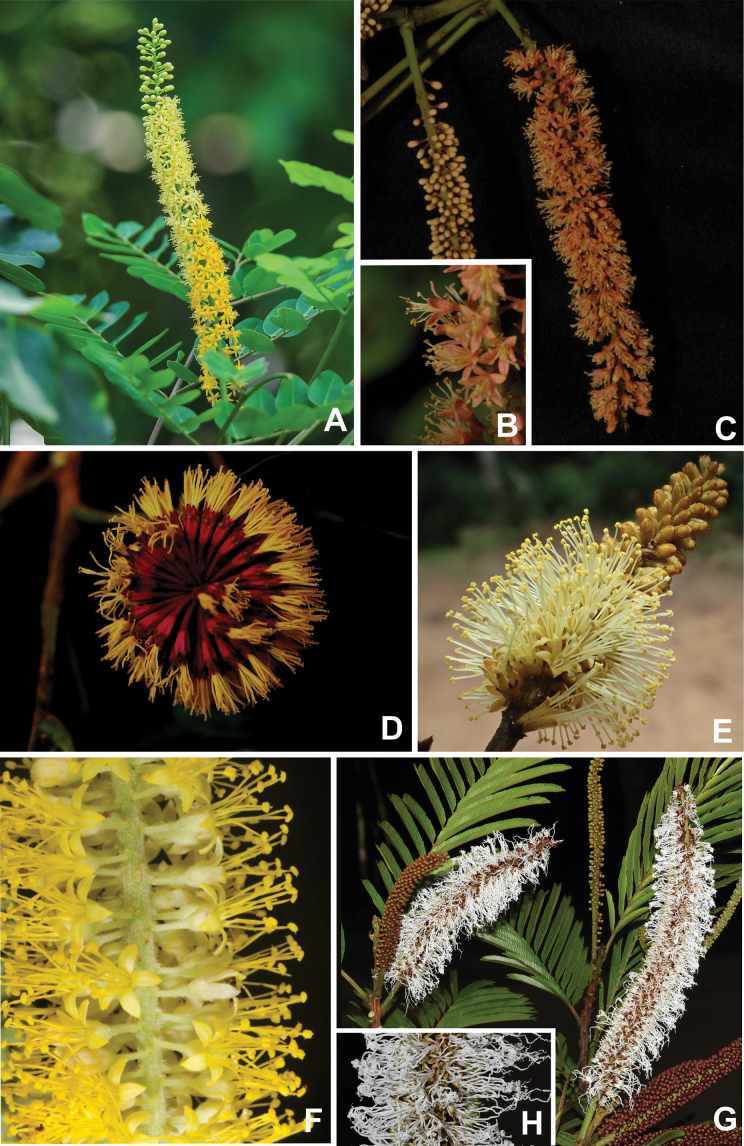
Flowers and inflorescences in the Adenanthera clade **A***Adenantherapavonina* L. inflorescence **B, C***Tetrapleuratetraptera* (Schumach. & Thonn.) Taub. (*Harris 9663*) **B** reflexed petals and white filaments on the flowers **C** racemose inflorescence **D***Xyliahoffmannii* (Vatke) Drake umbellate inflorescence (*Ratovoson 838*) **E***Calpocalyxklainei* Pierre ex Harms inflorescence (*Texia 976*) **F***Pseudoprosopisbampsiana* Lisowski flower detail showing pseudopedicels (*Bidault 2515*) **G, H***Pentaclethramacroloba* (Willd.) Kuntze **G** inflorescences congested at the ends of branches **H** close-up of the inflorescence showing the long filamentous staminodes. Photo credits **A** CheongWeei Gan **B, C** DJ Harris **D** F Ratovoson **E** N Texia **F** E Bidault **G, H** D Cardoso.

The *Pseudoprosopis*, *Xylia* and *Calpocalyx* clade shares the character of leaflets and pinnae opposite one another. *Pseudoprosopis* stands out as the only genus in the Adenanthera clade to have a liana habit, and some species have unique lignified tendrils for climbing (Fig. 104F) *Xylia* and *Calpocalyx* are the only genera in the clade to bear foliar nectaries and both genera consistently have only a single pair of pinnae. *Calpocalyx* has centrifugal floral maturation along the spike in some species, not seen elsewhere in this group (Fig. 105E). *Xylia* has been distinguished from *Calpocalyx* as having capitate or umbellate inflorescences as opposed to spicate ones (Fig. 105D). The two genera are not monophyletic in the recent phylogeny of Ringelberg et al. (2022) and we will undertake a realignment of the genera pending additional nomenclatural and morphological study. The generic name *Esclerona* Rafinesque predates the name *Xylia* which has long been used as a generic name. Given the wide distribution of the genus in Africa, Madagascar, and Asia, *Xylia* should be conserved against *Esclerona* to maintain nomenclatural stability. The name *Xylia* predates the name *Calpocalyx*, and so generic recircumscription also awaits nomenclatural conservation. We have called this clade the Adenanthera clade rather than the Xylia clade in contrast to Koenen et al. (2020a) also due to the unresolved nomenclatural issues.

**Figure 106.**
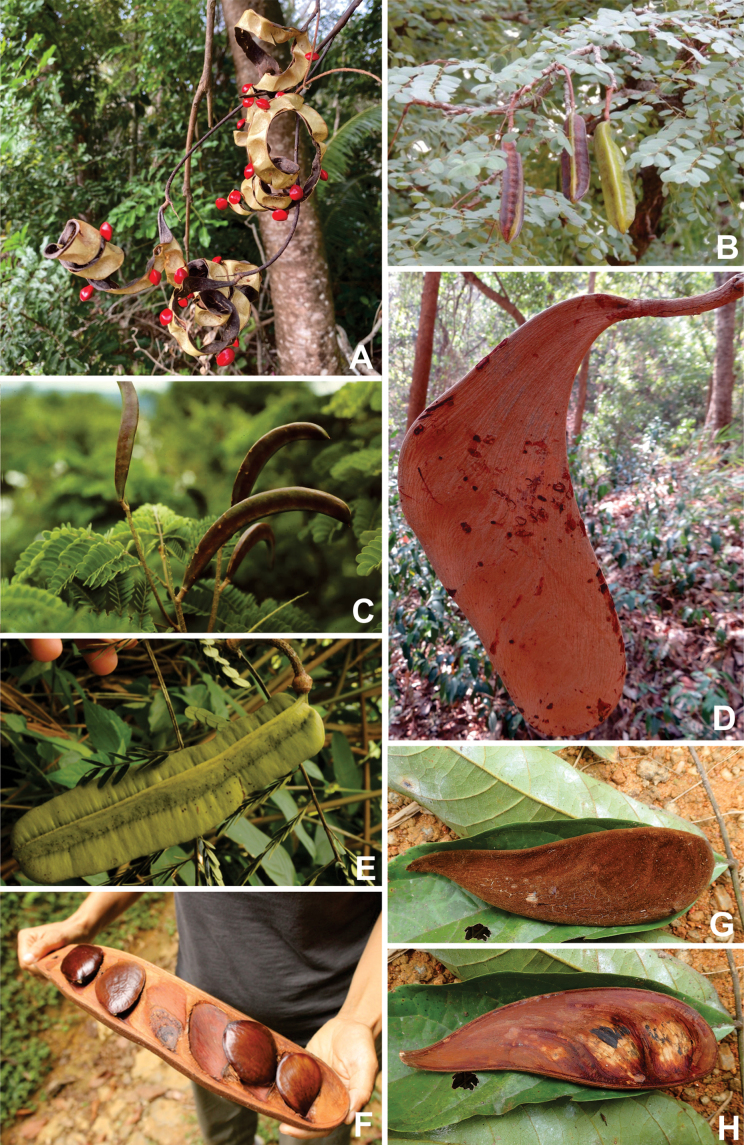
Fruits and seeds in the Adenanthera clade **A***Adenantherapavonina* L. dehisced fruits with exposed persistent red seeds **B***Amblygonocarpusandongensis* (Welw. ex Oliv.) Exell & Torre Indehiscent four-angled fruits (*Catarino 2067*) **C***Pseudoprosopisfischeri* Harms young fruit (*Bingham 8055*) **D**Xyliaxylocarpavar.kerrii (Craib & Hutch.) I.C.Nielsen young dolabriform pod **E***Tetrapleuratetraptera* (Schumach. & Thonn.) Taub. immature winged, indehiscent pod (*Harris 9659*) **F***Pentaclethramacrophylla* Benth. pod and recalcitrant seeds **G, H***Calpocalyxngouniensis* Pellegr. woody valves from dehisced pod (*Texier 695*). Photo credits **A** Shelbyfarmer **B** L Catarino **C** MG Bingham **D** B Peroth **E** DJ Harris 9659 **F** CJ Porto, Fundación Tierra Ibérica **G H** N Texier.

Most Adenanthera clade species inhabit humid forests, all in Africa and Asia, except for one New World species of *Pentaclethra*. Some species tolerate littoral and gallery evergreen forests, but *Xyliaxylocarpa* (Roxb.) W. Theob. is the only species to grow in drier semi-deciduous forests and *Amblygonocarpusandongensis* (Welw. ex Oliv.) Exell & Torre is the only one that has moved into the savannas of Africa (Fig. 104I). Many morphological characters are likely adaptations to humid forests, such as tall habit with buttresses and woody explosively dehiscent pods and recalcitrant seeds. Most species are described as having very fragrant flowers.

### ﻿ Pentaclethra

Taxon classificationPlantaeFabalesFabaceae

Benth., J. Bot. (Hooker) 2: 127. 1840.

**Figure 107.**
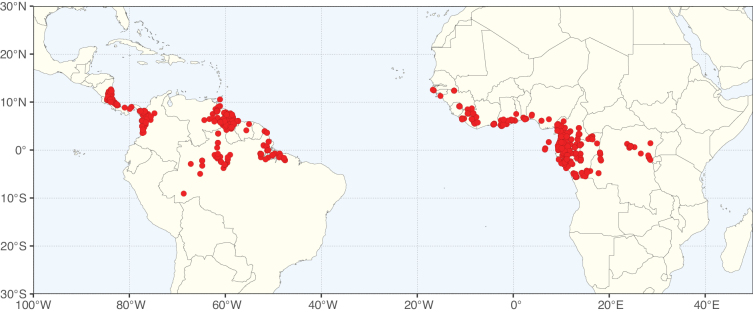
Distribution of *Pentaclethra* based on quality-controlled digitised herbarium records. See Suppl. material 1 for the source of occurrence data.

Figs 104 , 105 , 106 , 107

#### Type.

*Pentaclethrafilamentosa* Benth. [≡ *Pentaclethramacroloba* (Willd.) Kuntze (≡ *Acaciamacroloba* Willd.)]

#### Description.

Large, unarmed, often buttressed trees 10–35 m tall, (Fig. 104C) to 1.3 m in diameter, adventitious roots sometimes present; bark mottled grey/brown, smooth; young stems with dark brown stripes when dried, pubescent with rusty or golden appressed hairs, brachyblasts absent. **Stipules** small, linear, caducous. **Leaves** large, bipinnate, foliar nectaries absent; pinnae 5–20 pairs per leaf, opposite, petiole and pinnae adaxially pubescent with erect golden or rusty hairs; leaflets 10–60 pairs per pinna, opposite, sessile, oblong to linear-falcate, glabrous. **Inflorescence** a terminal panicle of spikes (rarely solitary) (Fig. 105G), axis bearing hundreds of tiny sessile flowers, the spike 5–20 (30) cm long, to 3.5 cm broad at anthesis. **Flowers** 5-merous; sepals connate, calyx campanulate, lobed, glabrous, green to yellow, calyx lobes imbricate in bud; petals connate, valvate in bud, glabrous, fleshy, the margins of the lobes inrolled and the apex of each petal keeled, white, green, or pink to red in colour; androecium fused to the petals forming a stemonozone, filaments sometimes connate above the petals as well, each flower with 5 fertile stamens alternating with 5 sterile staminodia, the latter opposite the petals, filamentous, white to cream, exserted beyond the corolla, fertile stamens with the filaments white, anthers dorsifixed, oval, opening by pouches, each bearing a large anther gland at the apex; pollen of tricolporate monads; ovary sessile, rusty-pubescent, linear to ovate, style equal to or shorter than the fertile stamens, stigma punctate. **Fruit** a woody, explosively dehiscent legume, valves curling after dehiscence, 15–50 (65) cm long, oblong, clavate, 5–8-seeded; exocarp dark brown, glabrous, striate with longitudinal veins; endocarp smooth, light brown, fibrous, not partitioned between the seeds. **Seeds** large, recalcitrant, inserted obliquely, orbicular to ovate, 4–7 × 2.5–3.5 cm, testa papery, pleurogram absent (Fig. 106F).

#### Chromosome number.

2*n* = 26 (Fedorov 1969; Goldblatt and Johnson 1990).

#### Included species and geographic distribution.

Three species, one in Latin America, widespread from Central America (Guatemala, Costa Rica, and Panama) into South America (Venezuela, Colombia, and Brazil), and the islands of Trinidad and Tobago; two species in West Africa, from Senegal to Angola and the Congo, also the islands of Principe and San José (Fig. 107).

#### Ecology.

Lowland humid and sub-humid forests, often on riverine or waterlogged soils, sometimes the dominant tree species particularly in the Americas. Seeds are demonstrated to be hydrochorous in *Pentaclethramacroloba* (Williamson and Costa 2000).

#### Etymology.

Claimed to be from the Greek, *pente* (= five) and *kleithro* (= bolt), alluding to five imbricate sepals and five petals joined at their bases (Barroso et al. 1991).

#### Human uses.

*Pentaclethramacrophylla* has many traditional uses, and is often planted around homes to shade gardens, improve soil fertility, and provide food, medicine, and timber. Known as the African oil bean tree, Owala oil tree, or Atta bean, the seeds are either roasted for up to 12 hours or fermented for several days to make an edible paste called ugba, high in protein and oil (Achinewhu 1982). Oil from the seeds also has antimicrobial properties and is a promising natural ointment for wounds (Ugbogu and Akukwe 2009). Leaves and bark are used medicinally as an anti-diarrheal, the scientific basis of which has been demonstrated (Akah et al. 1999). Although nitrogen fixation was not confirmed scientifically until 1993 (Ladipo et al. 1993), the trees have long been grown in Nigeria as a green manure crop. *Pentaclethramacroloba* has many of the same traditional uses as the African species, as a source of wood, medicinals, and oil. The oil from this tree is known as “Pracaxi oil” or “Pracachy oil” and is high in behenic acid, a compound important to the cosmetic industry for its moisturizing properties. It is being commercially marketed as a natural botanical for skin problems.

#### Notes.

*Pentaclethra* is often considered a transitional genus between the core mimosoids and the rest of the Caesalpinioideae. It shares a number of characters with the genus *Dimorphandra* Schott such as woody, clavate pods, flowers bearing staminodia alternating with fertile stamens, and imbricate sepals; however recent studies have shown the floral characters to be independently derived (Barros et al. 2017).

The genus has been considered a classic example of a Gondwanan distribution, being disjunct between western Africa and Central and South America. The very short-lived (recalcitrant) seeds with a thin, papery testa coupled with a heavy, elastically dehiscent fruit seems to preclude a dispersal event across the Atlantic, although the much younger age now estimated for the legume family argues against a vicariant distribution. It is intriguing that *P.macrophylla* occurs on islands off the west coast of Africa and *P.macroloba* in Trinidad and Tobago. Seeds of *P.macroloba* are hydrochorous in freshwater (Williamson and Costa 2000; Rocha-Dantas et al. 2021) and two of the species are quite common on coastal plateaus. It would be interesting to test if the seeds remain viable in salt water, thus providing a means of amphi-Atlantic dispersal.

#### Taxonomic references.

Flores (2002); Schery (1950), illustration; Villiers (1989), illustration.

### ﻿ Tetrapleura

Taxon classificationPlantaeFabalesFabaceae

Benth., J. Bot. (Hooker) 4: 345. 1841.

**Figure 108.**
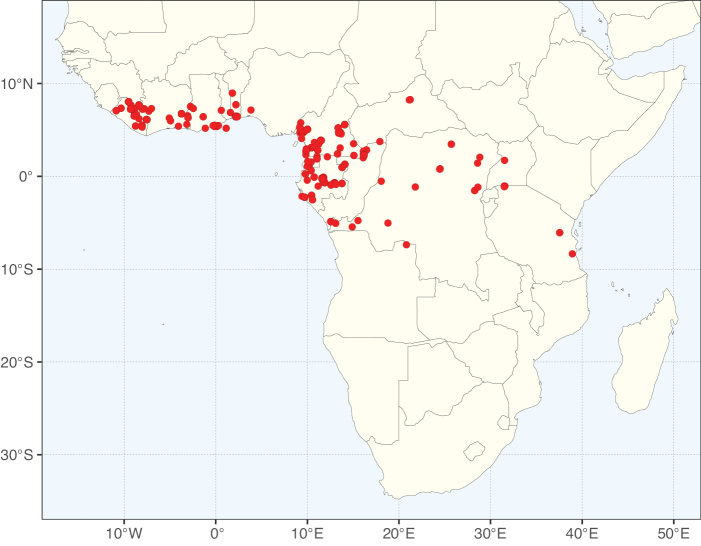
Distribution of *Tetrapleura* based on quality-controlled digitised herbarium records. See Suppl. material 1 for the source of occurrence data.

Figs 104 , 105 , 106 , 108

#### Type.

*Tetrapleurathonningii* Benth., nom. illeg. [= *Tetrapleuratetraptera* (Schumach. & Thonn.) Taub. (≡ *Adenantheratetraptera* Schumach. & Thonn.)]

#### Description.

Unarmed trees or shrubs 8–25 m tall (Fig. 104D), to 60–90 cm in diameter, usually lacking buttresses but occasionally with short, sharp buttresses; bark smooth, dark brown, very thin, slightly spongy; brachyblasts absent. **Stipules** not observed. **Leaves** bipinnate, 10–40 cm long, foliar glands absent; pinnae 2–10 (15) pairs per leaf, opposite to subopposite; leaflets alternate, 6–26 per pinna, petiolulate. **Inflorescences** of solitary or paired spiciform axillary racemes, 5–10 cm long (Fig. 105C), usually borne on older wood and immersed in the foliage, but the leaves sometimes suppressed and the racemes forming a pseudopanicle. **Flowers** hermaphrodite, pedicellate, the pedicel abscising with the flowers, small persistent triangular bracts at the base of each flower; calyx conical, shallow, 5-toothed, valvate in bud; petals 5, free, linear-lanceolate, cream to pale pink aging orange-red, valvate in bud (Fig. 105B,C); stamens 10, free, slightly exserted above the petals, filaments white, anthers with a caducous apical gland; pollen in 16-grained calymmate polyads; ovary oblong, sessile, glabrous, stigma porate. **Fruits** straight, oblong, woody, indehiscent, 10–20 cm long, four-winged with flattened sutural ribs and longitudinal wings running down the centre of the valve (Fig. 106E), each wing to 1 cm broad, two of the wings filled with pulp, cruciform in cross-section, dark brown, pods internally septate between the seeds with spongy, fibrous endocarp. **Seeds** inserted transversely, brown, smooth, unwinged, testa hard, pleurogram present.

#### Chromosome number.

2*n* = 26 (Fedorov 1969).

#### Included species and geographic distribution.

Two species in tropical Africa, in the Guineo-Congolese forest from Senegal to Sudan, Uganda, and Kenya, south to Angola and Tanzania (Fig. 108).

#### Ecology.

Common in secondary forest but growing best in undisturbed rainforest; high forest zones, riverine forests, southern savanna woodland, and as forest outliers in the African plains.

#### Etymology.

From the Greek, *tetra* (= four) and *pleura* (= ribs), referring to the four ribs on the fruits.

#### Human uses.

A valued forest species for its fragrant fruits and seeds which are used to season food. The fruits, seeds, leaves, and bark are all used in folk medicine to treat a wide variety of ailments. The chemistry of the fruits has been studied and found to be a potential treatment for diabetes and to reduce inflammation (Orwa et al. 2009; Kuate et al. 2015).

#### Notes.

*Tetrapleura* differs from *Amblygonocarpus* in minor characters as described below under *Amblygonocarpus*. Although they might be treated as a single genus, the two genera tend to be ecologically distinct. *Tetrapleura* is a rainforest tree that only occasionally moves into the savanna as a forest outlier whereas *Amblygonocarpus* is a true inhabitant of savannas and deciduous forests.

#### Taxonomic references.

Brenan (1959) with illustration; Kemigisha et al. (2018).

### ﻿ Amblygonocarpus

Taxon classificationPlantaeFabalesFabaceae

Harms, Nat. Pflanzenfam. Nachtr. II–IV 1: 191. 1897.

**Figure 109.**
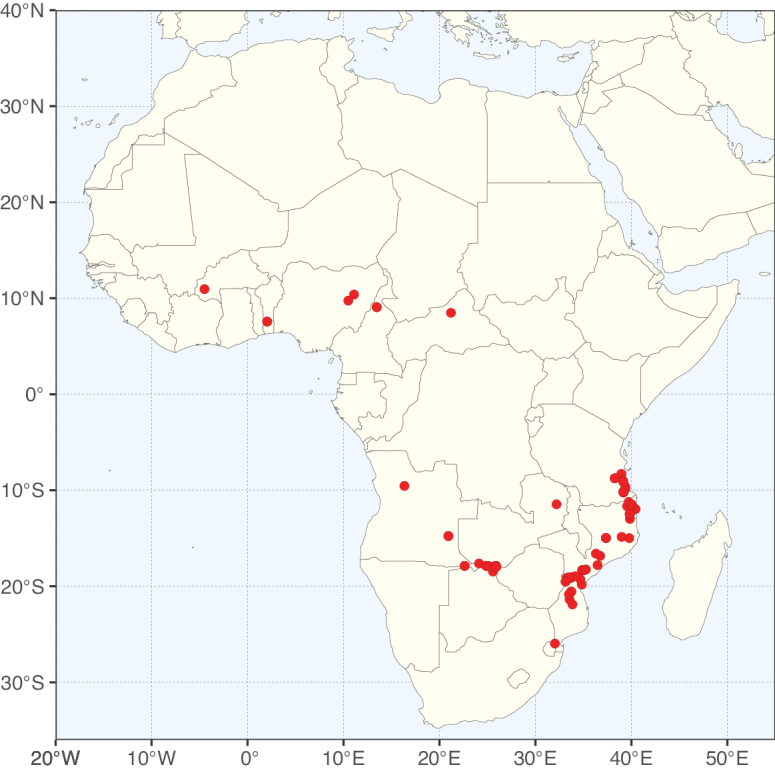
Distribution of *Amblygonocarpus* based on quality-controlled digitised herbarium records. See Suppl. material 1 for the source of occurrence data.

Figs 104 , 106 , 109

#### Type.

*Amblygonocarpusschweinfurthii* Harms [= *Amblygonocarpusandongensis* (Welw. ex Oliv.) Exell & Torre (= *Tetrapleuraandongensis* Welw. ex Oliv.)]

#### Description.

Unarmed trees 8–20 m tall, 25–75 cm diameter, with a broad, umbrella-shaped crown (Fig. 104I), buttresses absent, bark grey, scaly (Fig. 104E); plant glabrous throughout; brachyblasts absent. **Stipules** minute, linear, caducous. **Leaves** bipinnate, 20–30 cm long, foliar nectaries absent; pinnae opposite or alternate, 2–6 pairs per leaf; leaflets alternate, 8–16 per pinna, petiolulate, elliptic to obovate-elliptic. **Inflorescences** axillary, solitary or paired racemes, borne on old wood and subtending the current flush of leaves and thus immersed in the foliage, 5–13 cm long, flowers ca. 50–75 per raceme. **Flowers** seemingly all bisexual, pseudopedicellate, these abscising with the flowers, ebracteolate; calyx broad, shallow, 5 (6) lobed, valvate in bud; petals lanceolate, 5 (6), free, cream to white, aging yellow, valvate in bud; stamens 10 (12), free, filaments white, anthers dorsifixed, oblong-ovate, eglandular; pollen in calymmate 16 or 32-grained polyads; ovary oblong, sessile, glabrous. **Fruits** pendent, stipitate, straight, oblong, woody, indehiscent, 10–15 cm long, bluntly tetragonal or subterete in cross section (Fig. 106B), each side of the fruit ca. 2 cm wide, dark brown, glossy, internally septate between the seeds with spongy, fibrous endocarp, ca. 6–8-seeded. **Seeds** inserted transversely in the pod, brown, smooth, unwinged, testa hard, pleurogram present.

#### Chromosome number.

2*n* = 28 (Goldblatt and Davidse 1977).

#### Included species and geographic distribution.

One species (*A.andongensis*), widespread in savannas from northern Ghana east to Sudan, in the south from Angola through Zambia and Botswana to Tanzania and Mozambique (Fig. 109).

#### Ecology.

Savannas and deciduous woodlands, frequently on sandy soils, often associated with *Senegalia* Raf., *Burkea* Benth. and *Albizia* Durazz.

#### Etymology.

From the Greek, *ambly*- (= blunt), *gonia*- (= angle), -*carpus* (= fruit), in reference to the angled fruits.

#### Human uses.

The seeds of *Amblygonocarpus* are harvested in the wild and eaten roasted. The roots, bark, and leaves are used in folk medicine to treat a wide variety of ailments. The wood is extremely hard and is used to make furniture, small implements, and heavy-duty flooring; also used as a fuel and to make charcoal (Fern 2023).

#### Notes.

*Amblygonocarpus* is closely related to *Tetrapleura* and *Adenanthera* in the recent phylogeny of Ringelberg et al. (2022). The flowers and foliage of *Tetrapleura* and *Amblygonocarpus* are quite similar and are sometimes confused when not in fruit. *Amblygonocarpus* is glabrous throughout with reddish brown petiole, rachis, and pinnae whereas *Tetrapleura* has pubescence on the petiole, rachis, pinnae, and often leaflets. Flowers are completely glabrous in *Amblygonocarpus* and the anthers are eglandular; the calyx is always pubescent in *Tetrapleura* and the anther is equipped with an apical gland. Both genera have tetragonal fruits, but the margins form well-developed wings in *Tetrapleura* whereas they are bluntly angled in *Amblygonocarpus*. *Adenanthera* is rarely confused with the other two genera because of its dehiscent fruits with red seeds and Asian distribution.

#### Taxonomic references.

Brenan (1959), with illustration.

### ﻿ Adenanthera

Taxon classificationPlantaeFabalesFabaceae

L., Sp. Pl.: 384. 1753.

**Figure 110.**
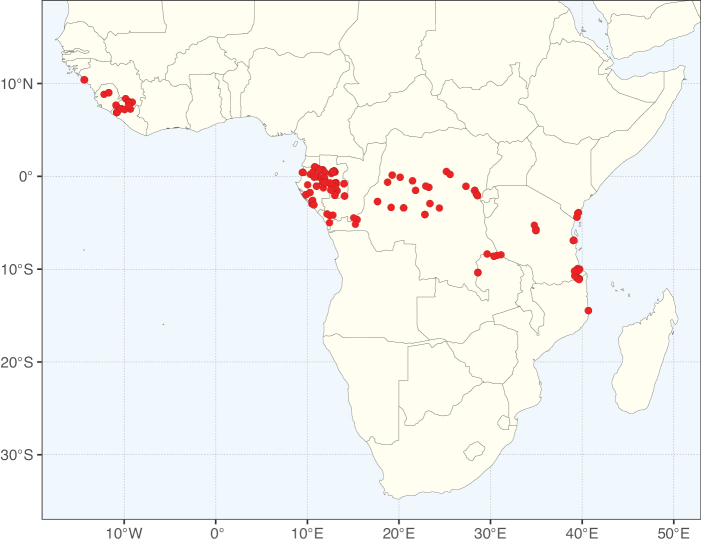
Distribution of *Adenanthera* based on quality-controlled digitised herbarium records. See Suppl. material 1 for the source of occurrence data.

Figs 104 , 105 , 106 , 110

Gonsii Adans., Fam. Pl. 2: 318. 1763. Type not designated.

#### Type.

*Adenantherapavonina* L.

#### Description.

Unarmed trees or shrubs (*A.marina* Nielsen), (3) 20–45 m tall, to 1 m diameter (Fig. 104H), buttresses present in some species, bark brown-grey, peeling or flaking (Fig. 104G); brachyblasts absent. **Stipules** inconspicuous and caducous. **Leaves** bipinnate, foliar nectaries absent; pinnae opposite or subopposite, (1) 2–9 pairs per leaf, 3–25 (40) cm long; leaflets alternate, 4–25 per pinna, petiolulate. **Inflorescences** of pendulous or erect, pedunculate, spiciform racemes (Fig. 105A), (3.5) 5–30 cm long, solitary or paired in the leaf axils, borne near the ends of the branches, leaves sometimes suppressed and the inflorescence a narrow panicle of racemes, pedicels jointed and leaving a peg-like structure when the flowers abscise. **Flowers** fragrant, many per raceme, all hermaphrodite and seemingly bisexual; sepals connate, calyx campanulate, 5-lobed, valvate in bud; petals 5, free or basally connate, sometimes loosely connate to the stamens but soon separating, cream, pale yellow or pink, reflexed at anthesis, valvate in bud; stamens 10, free, anthers bearing an apical stipitate gland; nectary disk absent; pollen in calymmate polyads with (4) 16 (32) grains; ovary linear, sessile or stipitate, glabrous or pubescent, stigma porate. **Fruits** linear-oblong, 8–25 × 0.8–2 cm, straight, curved or spirally twisted prior to dehiscence, usually with 6–10 seeds per pod, up to 25 in *A.pavonina*, valves coriaceous to chartaceous, dehiscent through both sutures, spiraling after dehiscence to expose the persistent seeds; exocarp dark brown to black; endocarp pale yellow, smooth, raised over the seeds (Fig. 106A). **Seeds** red or bicoloured red (in hilar end) and black, obliquely inserted, ellipsoid, obovoid, orbicular or ovoid-ellipsoid, biconvex, compressed, testa hard, funicle thickened, pleurogram present.

#### Chromosome number.

2*n* = 26, 28 (Goldblatt 1981b).

#### Included species and geographic distribution.

Twelve species in tropical Asia, Australasia, Melanesia, Solomon Islands, and Madagascar (Fig. 110). *Adenantherapavonina* is widely cultivated throughout the tropics as an ornamental; it is either native to India or was introduced there in prehistoric times.

#### Ecology.

Primary and secondary rainforests 100–700 m elevation, as a canopy tree in dipterocarp forests (one species an understory tree), peat swamps and swampy forests, forest margins, savannahs, alluvial forests, and two species coastal.

#### Etymology.

Greek *Aden*- (= gland) and *anthera*, referring to the gland present at the apex of the anther.

#### Human uses.

Planted in villages as an ornamental, used as shade trees for coffee and as a nitrogen-fixer. The seeds are toxic fresh but can be cooked and eaten, as can the leaves. The seeds are also used for making jewelry and in India to make a red dye. Leaves, bark, and seeds have been used in traditional medicine, and recent studies of seed extracts have shown *A.pavonina* to have antibacterial and anti-inflammatory effects (Olajide et al. 2004; Dholvitayakhun et al. 2012). The leaves are rich in saponins and used to make soap. Wood is sometimes used for indoor construction such as furniture.

#### Notes.

*Adenantherapavonina* is widely naturalised in Africa and parts of Asia, and in the New World in the Caribbean, Florida, and northern South America. The red or bicoloured seeds are persistent after the pods dehisce and are bird-dispersed.

#### Taxonomic references.

Nielsen (1992); Nielsen and Guinet (1992), illustrations.

### ﻿ Pseudoprosopis

Taxon classificationPlantaeFabalesFabaceae

Harms, Bot. Jahrb. Syst. 33: 152. 1902.

**Figure 111.**
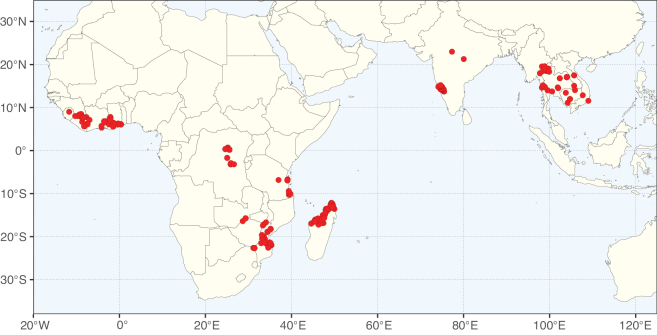
Distribution of *Pseudoprosopis* based on quality-controlled digitised herbarium records. See Suppl. material 1 for the source of occurrence data.

Figs 104 , 105 , 106 , 111

#### Type.

*Pseudoprosopisfischeri* (Taub.) Harms [≡ *Prosopisfischeri* Taub.]

#### Description.

Large woody lianas or scandent shrubs or small trees, 3–6 m, coppicing in one species; unarmed but sometimes having large, hooked, lignified tendrils for climbing (Fig. 104F), stems longitudinally striate with corky ridges; brachyblasts absent. **Stipules** small, linear to triangular, caducous; the leaf node often bearing small, mounded glands on either side of the stipule scars. **Leaves** bipinnate, foliar nectaries absent; pinnae opposite to subopposite, 1–3 in species with macrophyllous leaflets, 3–12 in those with smaller leaflets, 3.5–18 cm long; leaflets opposite, short petiolulate, 2–4 pairs per pinna in macrophyllous species, lanceolate, 7–28 pairs in the microphyllous ones, oblong to linear. **Inflorescences** racemes 4–13 cm long, solitary or 2–3 per node, usually grouped in leafless terminal pseudopanicles borne above the foliage, inflorescence axes densely pubescent. **Flowers** hermaphrodite, fragrant, pedicellate, jointed between the pedicel and the attenuate calyx, the pedicels remaining as small mounds or pegs when the flowers abscise (Fig. 105F), floral bracts 3-lobed, enlarged; calyx connate, 5-lobed, obconic to cupulate, attenuate basally forming a pseudopedicel, valvate in bud; petals 5, free, white or cream, often abaxially pubescent and rusty or brown, reflexed, valvate in bud; stamens 10, free, filaments and anthers bright yellow, anther gland present, stipitate; pollen in calymmate 8 or 16-grained polyads; ovary sessile or stipitate, stigma porate. **Fruit** a woody, explosively dehiscent legume, valves reflexed after dehiscence, clavate to elliptical (Fig. 106C), 6–16 × 1–3 cm, 6–11-seeded; exocarp dark brown to black, obliquely striate. **Seeds** inserted obliquely, testa hard, pleurogram present.

#### Chromosome number.

Unknown.

#### Included species and geographic distribution.

Seven species in the Guineo-Congolian region to Tanzania and south through Mozambique (Fig. 111).

#### Ecology.

Lowland humid and sub-humid forests in Guineo-Congolian region, where they may occur in primary forests and also in secondary forests and riparian areas. In Tanzania and Mozambique, they are found on the edges of evergreen and deciduous gallery forests, or in thickets in cutover regions.

#### Etymology.

*Pseudo* (false) and *Prosopis*, referring to its similarity to the genus *Prosopis* L.

#### Human uses.

Used as a fish poison in the Democratic Republic of Congo (Villiers 1983).

#### Notes.

*Pseudoprosopis* is the only genus in the Adenanthera clade having the liana habit. The uncinate lignified tendrils on the stems, although not universal in the genus, are also distinctive. Two species, *Ps.bampsiana* Lisowski, and *Ps.euryphylla* Harms are listed as vulnerable and near-threatened respectively by IUCN.

#### Taxonomic references.

Brenan (1959); Lisowski (1982); Villiers (1983), all with illustrations.

### ﻿ Xylia

Taxon classificationPlantaeFabalesFabaceae

Benth., J. Bot. (Hooker) 4: 417. 1842.

**Figure 112.**
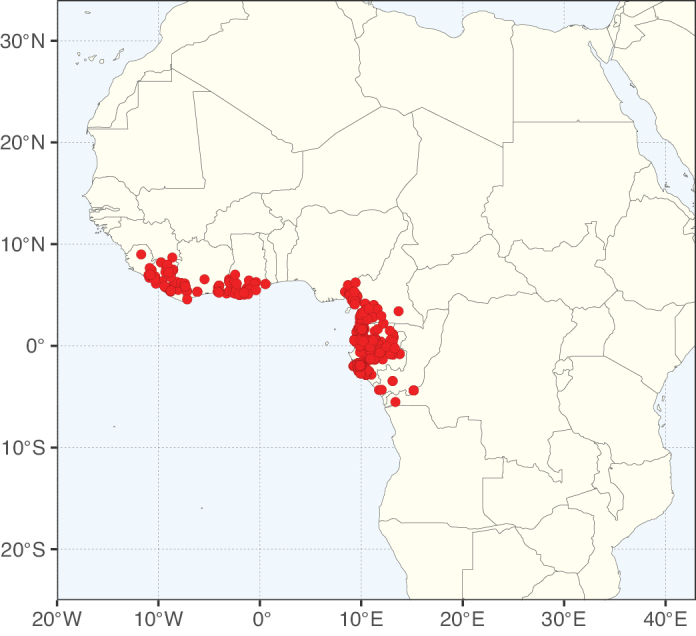
Distribution of *Xylia* based on quality-controlled digitised herbarium records. See Suppl. material 1 for the source of occurrence data.

Figs 104 , 105 , 106 , 112

Esclerona Raf., Sylva Tellur.: 120. 1838. Type: Escleronamontana Raf. [= Xyliaxylocarpa (Roxb.) Taub. (≡ Mimosaxylocarpa Roxb.)]
Xylolobus Kuntze, Lex. Gen. Phan.: 598. 1903, nom. superfl. Type not designated.

#### Type.

*Xyliadolabriformis* Benth. [= Xyliaxylocarpa(Roxb.)Taub.var.xylocarpa (≡ *Mimosaxylocarpa* Roxb.)]

#### Description.

Shrubs or trees, (6) 15–30 m tall, to 40 cm diameter, unarmed, evergreen or deciduous (Fig. 104B); golden pubescent on the calyx, corolla, and young stems; bark dark red brown to grey, rough or scaly (Fig. 104A); brachyblasts absent. **Stipules** lanceolate-linear, to 1 cm, precocious on new growth, sessile, caducous. **Leaves** large, bipinnate, nectary borne at the apex of the rachis between the pinnae, round, mound-shaped, nectaries usually also variably borne between distal pairs of leaflets, and sometimes between all pairs of leaflets; pinna 1 pair, 5–30 cm long; leaflets 3–20 pairs per pinna, opposite, the proximal pair sometimes reduced to a single leaflet, petiolulate, oblanceolate to lanceolate. **Inflorescences** of capitula or congested umbels (Fig. 105D) on longish, usually flattened (exception *X.hoffmannii* (Vatke) Drake) peduncles, 1–2 per axil, borne in the axils of coeval leaves or more often aggregated into paniculiform secondary inflorescences on older branches from which the leaves have abscised, flower-bearing branches apparently indeterminate. **Flowers** sessile or appearing pedicellate in some species by elongation of the receptacle, floral bracts peltate and pedicellate, pubescent; calyx connate, 5-lobed, valvate or slightly imbricate in bud; petals 5, free or connate, pale yellow to cream or dark red, valvate in bud; stamens 10, the filaments sometimes flattened and ribbon-like, anthers dorsifixed, usually bearing a caducous, stalked anther gland; pollen in calymmate 8, 12, or 16- grained polyads; ovary sessile, densely pilose, the style attached to one side of the ovary, style exserted, stigma porate. **Fruit** a woody legume, explosively dehiscent from the apex through both sutures, dolabriform (Fig. 106D), 5–15 × 5–6 cm, ca. 4–10-seeded, the valves recurved after dehiscence, the exocarp dull, cracking and falling away to expose a longitudinally veined, fibrous mesocarp, interior of fruit smooth. **Seeds** obliquely or transversely inserted, sunken into indentations in the pod, brown, funicles fleshy and enlarged, testa hard, pleurogram present.

#### Included species and geographic distribution.

Nine species, West Africa from Guinea to Ghana; central and southern Africa from Democratic Republic of the Congo to Tanzania, Mozambique, and South Africa, Madagascar, India, and South East Asia (Fig. 112).

#### Chromosome number.

*n* = 12 (Moore 1974, 1977; Goldblatt 1981b).

#### Ecology.

Evergreen, semi-deciduous and gallery forests.

#### Etymology.

From the Greek *xylon*, referring to the very hard wood in this genus.

#### Human uses.

Valued as a timber tree. The wood is very hard and is used in heavy construction, for houses, bridges, ship building, and tools as well as for fuel and charcoal. Used in reforestation in South East Asia. Seeds are eaten as a vegetable, and the bark and seeds are used in folk medicine to treat a wide variety of ailments. *Xyliaxylocarpa* is grown as a shade tree in India (Fern 2023).

#### Notes.

*Xylia* has distinctive woody, dolabriform legumes, seen elsewhere only in *Pentaclethra*, *Calypocalyx*, *Pseudoprosopis* and *Dimorphandra*. The inflorescences vary from 1–2 heads borne in the axils of coeval, well-developed leaves in some species to complex paniculiform inflorescences with numerous aggregated heads and suppressed leaves in other species. In a few species, simple, linear bracts are present in the inflorescence instead of leaves and the peduncles may bear 1-several bracts below the developing inflorescences. Generally, these bracts are caducous. Inflorescences are also characterised by having precocious development of the foliar glands which reach their full size on very small leaves. The glands are conspicuous in the inflorescence and possibly serve either to distract potential floral predators from the developing flowers or to attract protectors. Likewise, the terminus of the rachis is precociously developed, overtopping the pinnae and leaflets, and appearing bract-like. As discussed in more detail in the Adenanthera clade notes, *Calpocalyx* is nested within *Xylia*, rendering the latter non-monophyletic (Fig. 103).

#### Taxonomic references.

Brenan (1959); Villiers (2002).

### ﻿ Calpocalyx

Taxon classificationPlantaeFabalesFabaceae

Harms, Nat. Pflanzenfam. Nachtr. [Engler & Prantl] I: 191. 1897.

**Figure 113.**
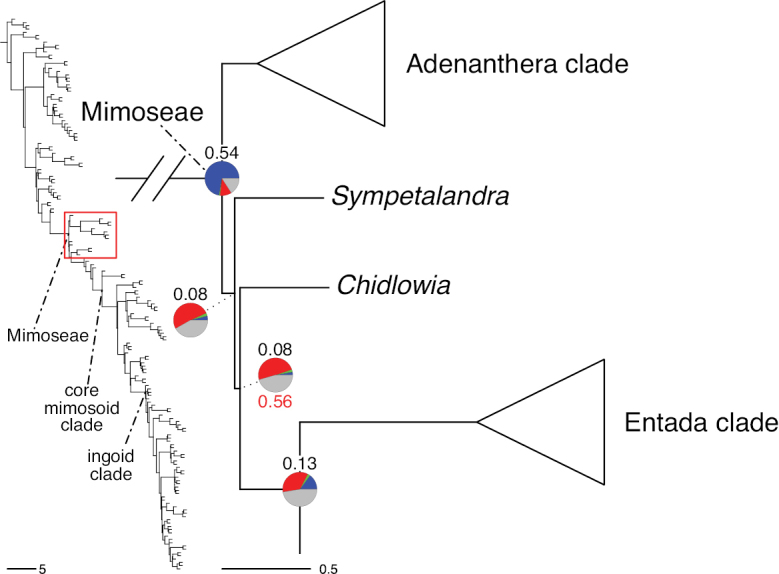
Distribution of *Calpocalyx* based on quality-controlled digitised herbarium records. See Suppl. material 1 for the source of occurrence data.

Figs 105 , 106 , 113

#### Type species.

*Calpocalyxdinklagei* (Taub.) Harms [≡ *Erythrophloeumdinklagei* Taub.]

#### Description.

Small trees or shrubs 5–20 m to tall forest trees to 50 m or more, the latter often with buttresses or water roots, unarmed, glabrous or pubescent; brachyblasts absent. **Stipules** small, linear, caducous and absent from most specimens. **Leaves** bipinnate, petiole usually terete, occasionally slightly sulcate, petiolar and rachis glands present, usually sunken into the petiole; pinnae 1 pair, 10–30 (50) cm long, opposite, articulated to the petiole; leaflets opposite, 1–9 pairs per pinna, the proximal pair of leaflets usually reduced to a single leaflet, macrophyllous, obovate to elliptic, petiolulate, the petiolules articulate to the rachis. **Inflorescences** of spikes, 2.5–11 cm long, oblong, subtended by triangular bracts, either solitary and axillary or more often aggregated into terminal complex-branched paniculiform secondary inflorescences with the spikes arranged in fascicles of 1–5, these subtended by three-parted bracteoles which bear an enlarged circular gland on the center bract, panicle immersed or exserted above the foliage; entire inflorescence usually fuscous to golden pubescent, anthesis often with centrifugal maturation (Fig. 105E). **Flowers** sessile; calyx cylindrical, connate, 5-lobed, valvate in bud; petals 5, connate, pale pink to cream, brownish-yellow, or brown, valvate in bud; stamens 10, not flattened, free or basally connate, sometimes adnate to the petals and forming a short stemonozone, anthers dorsifixed, bearing a caducous apical gland; pollen in calymmate 8-grained polyads; ovary sessile, densely gold-pubescent, style asymmetrically inserted, stigma porate. **Fruits** claviform to dolabriform, 10–25 × 2.5–9 cm, 4–8-seeded, valves woody (Fig. 106G, H), dehiscent along both sutures and curling after dehiscence, dorsiventrally flattened, not internally septate but the endocarp ridged and intruding between the seeds, epicarp usually papery, black-brown, exfoliating; endocarp smooth, reddish brown to dull brown, mesocarp longitudinally fibrous. **Seeds** recalcitrant, smooth, unwinged, testa papery, shiny, pleurogram absent.

#### Chromosome number.

*n* = 12 (Goldblatt and Davidse 1977).

#### Included species and geographic distribution.

Eleven species, native to the humid Guinean-Congolese forests of West Africa (Fig. 113).

#### Ecology.

Littoral and coastal forests, and rainforest. Shrubs and treelets are generally in the understory of undisturbed forest but also flourish in older secondary forests. Larger buttressed trees occur in evergreen lowland forests, two species have hollow branchlets and are inhabited by ants, and cauliflory is reported in one species.

#### Etymology.

From Greek, *calpo* (= urn) and *kylix* (= drinking cup), referring to the urn-shaped calyx.

#### Human uses.

The wood is a valuable source of lumber and is used in construction for flooring, shipbuilding, furniture, and agricultural implements. The bark is used in traditional medicine to treat wounds and *C.dinklagei* has recently been found to contain powerful anti-inflammatory compounds (Kapche et al. 2017).

#### Notes.

Species boundaries in *Calpocalyx* are difficult and more sampling is necessary to properly delimit species. The genus has a number of unique features in the Adenanthera clade. Ant-associations, while common among the members of the Adenanthera clade, have not resulted in the formation of domatia except in *C.cauliflorus* Hoyle and *C.winkleri* (Harms) Harms. Mimoseae usually have synchronous flowering within an inflorescence, but some species of *Calpocalyx* demonstrate remarkable centrifugal maturation within a spike. Most species in the genus are listed as vulnerable by the IUCN, including *C.atlanticus* Villiers, *C.brevifolius* Villiers, *C.cauliflorus* Hoyle, *C.heitzii* Pellegr., *C.klainei* Pierre ex Harms, *C.letestui* Pellegr., and *C.ngouniensis* Pellegr. *Calpocalyx* is here recovered as nested within *Xylia* (Fig. 103), as discussed in the Adenanthera clade notes.

#### Taxonomic references.

Villiers (1984), illustrations.

## ﻿﻿14. *Sympetalandra*

Anne Bruneau^1^

Citation: Bruneau A (2024) 14. Sympetalandra. In: Bruneau A, Queiroz LP, Ringelberg JJ (Eds) Advances in Legume Systematics 14. Classification of Caesalpinioideae. Part 2: Higher-level classification. PhytoKeys 240: 225–227. https://doi.org/10.3897/phytokeys.240.101716

### ﻿ Sympetalandra

Taxon classificationPlantaeFabalesFabaceae

Stapf, Hooker’s Icon. Pl. 28: t. 2721. 1891.

**Figure 114.**
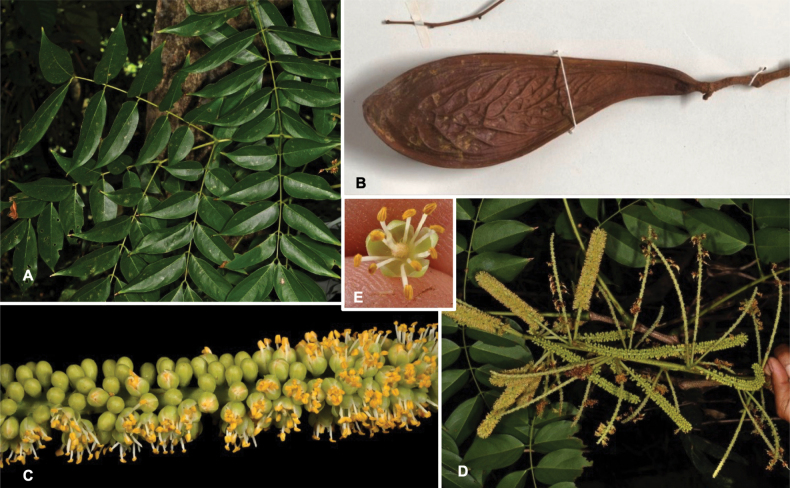
Phylogenetic position of *Sympetalandra* and *Chidlowia* in tribe Mimoseae. For description of phylogeny and support values, see Fig. 6 caption (page 63).

**Figure 115.**
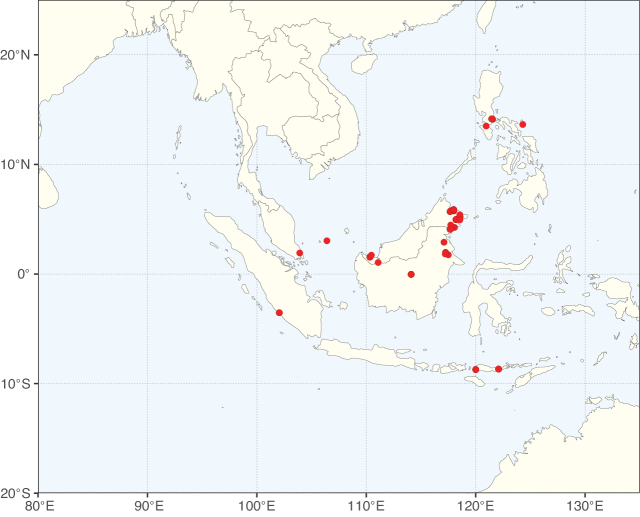
*Sympetalandraunijuga* (Airy Shaw) Steenis **A** bipinnate leaves with opposite pinnae and opposite leaflets **B** fruit, Malaysia (*Madani 83436*) **C** flowers in bud and early anthesis **D** slender, branched inflorescences **E** close-up of flower. Photo credits **A, C–E** Digital Flora of the Philippines (Pelser et al. 2011) **B** Naturalis Biodiversity Center, https://medialib.naturalis.nl/file/id/L.2027025/format/large (CC0-1.0).

**Figure 116.**
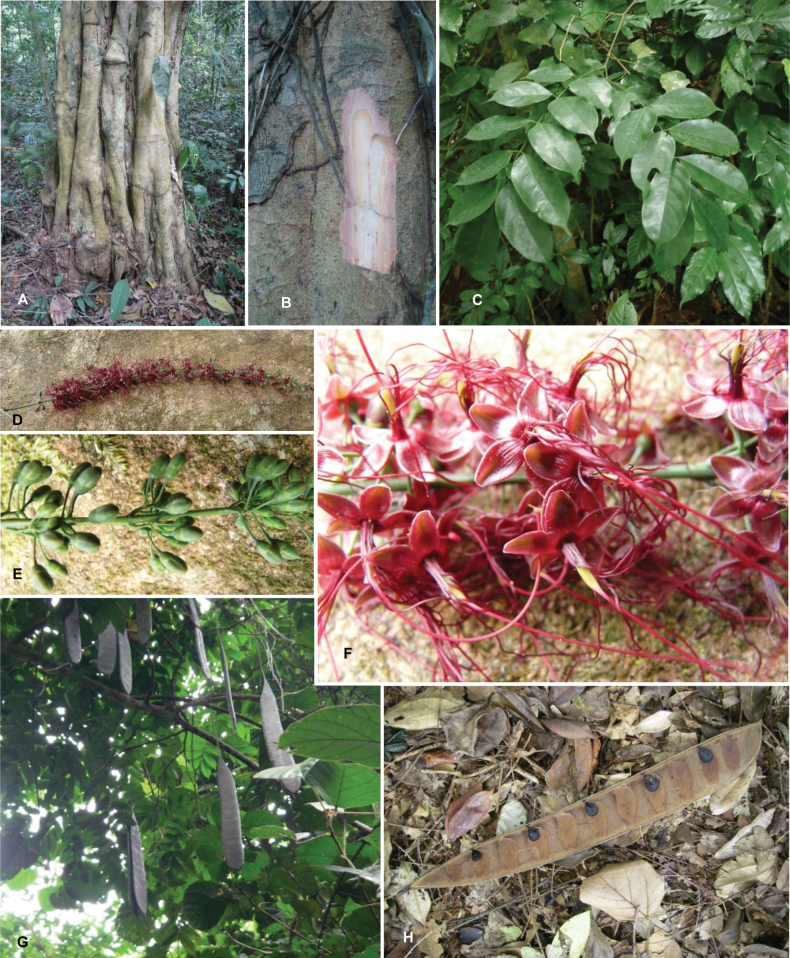
Distribution of *Sympetalandra* based on quality-controlled digitised herbarium records. See Suppl. material 1 for the source of occurrence data.

Figs 114 , 115 , 116

#### Type.

*Sympetalandraborneensis* Stapf

*Sympetalandra* is here considered a monogeneric lineage, whose relationship to other Mimoseae remains difficult to determine. Although historically considered a member of non-mimosoid Caesalpinioideae (Stapf 1891; Polhill and Vidal 1981; Polhill 1994), when first included in a molecular phylogenetic analysis the genus was resolved as a member of the mimosoid clade in the plastid *matK* analyses presented by LPWG (2017), a position that is now supported in the phylogenomic analyses of Ringelberg et al. (2022). As with *Chidlowia* Hoyle, the genus occurs nested between the Adenanthera and Entada clades, but the relationships between these two genera and these two clades are not clearly resolved (Fig. 114), as evidenced by the very short branches and possible polytomy at the base of the Mimoseae.

#### Description.

Unarmed, small to medium-sized tree (to 30 m), buttressed. **Stipules** minute or caducous. **Leaves** bipinnate (Fig. 115A), rarely parapinnate (*S.borneensis*), with 1–3 pairs opposite or subopposite pinnae; leaflets large and few, opposite, 3–6 pairs, pellucid glands present. **Inflorescences** dense, terminal and axillary racemes arranged in panicles towards the tip (Fig. 115C, D); bracts persistent beyond anthesis; bracteoles absent at anthesis. **Flowers** (Fig. 115E) small, 4 mm long, flower buds ovoid, crowded; pedicel ca. 1.2 mm, slender; calyx cup-shaped, hairy, with 5 small overlapping lobes, pellucid-glandular; petals 5 equal, imbricate, oblong, pellucid-glandular, joined at base; stamens 10, 5 long and 5 short in bud, free, adnate to petals at base; pollen in monads, spheroidal, ornamentation psilate to psilate-finely perforate (Banks and Lewis 2009); ovary stipitate, 2–6-ovulate, style long. **Fruit** thickly woody, elongate to 70 cm, flattened but bulging strongly at the big oval seeds (marked by an open reticulum of nerves), tardily dehiscent; valves with visible seed chambers, 1–3 seeds (Fig. 115B). **Seeds** orbicular to ellipsoid, testa osseous; funiculus less than 1 mm long; hilum concealed by funicular remnant; pleurogram absent.

#### Chromosome number.

Unknown, but recent phylogenomic analyses suggest the genus may be polyploid (Ringelberg et al. 2022, 2023).

#### Included species and geographic distribution.

Five species [*S.borneensis*, *S.densiflora* (Elmer) Steenis, *S.hildebrandii* Steenis, *S.schmutzii* Steenis, *S.unijuga* (Airy Shaw) Steenis] restricted to Malesia (Fig. 116): Sumatra, Malay Peninsula, Borneo, Philippines, Lesser Sunda Islands (Flores).

#### Ecology.

Lowland tropical forests. *Sympetalandradensiflora*, known as Kamatog, is considered near-threatened under the IUCN Red List in the Philippines.

#### Human uses.

The bark of *S.schmutzii* is used as a fish poison (Lewis 2005b). The wood of *S.densiflora* is of good quality and used in house constructions and furniture (Energy Development Corporation 2020).

#### Etymology.

The generic name refers to the petals that are shortly connate at their base.

#### Notes.

*Sympetalandra* was placed in the Dimorphandra group of tribe Caesalpinieae by Polhill and Vidal (1981), Polhill (1994), Lewis (2005b), and suggested to have affinity with this group of genera by Stapf (1891), but has been resolved as part of the mimosoid clade in molecular phylogenetic and phylogenomic analyses (LPWG 2017; Ringelberg et al. 2022). Although *Sympetalandra* lacks valvate petal aestivation, often considered a synapomorphy for core mimosoids, the genus has bipinnate leaves (paripinnate only in *S.borneensis*), racemose or paniculiform inflorescences, and small and regular flowers reminiscent of Mimoseae. Van Steenis (1975) noted morphological similarities in inflorescences and flower type with the genus *Adenanthera* L. (Adenanthera clade), and remarked on their very similar, almost equal, narrow petals. The monospecific African *Chidlowia*, which occurs in a similar phylogenetic position, also has flowers similar to those of *Sympetalandra* in which the base of the calyx, petals and stamens are joined and thickened into a structure simulating a hypanthium (Hoyle 1932).

*Sympetalandra* is subtended by a relatively long branch in the phylogenomic studies of Ringelberg et al. (2022) who noted a significant number of gene duplications, suggesting that the genus is likely polyploid. The occurrence of polyploidy, and particularly allopolyploidy, could be a factor contributing to the gene tree conflict observed among *Sympetalandra*, *Chidlowia* and the Adenanthera and Entada clades, which are the four first-branching lineages of Mimoseae. Given the short branches separating these four lineages, the high levels of gene tree conflict, and the slightly different relationships between these lineages found in the phylogenomic analyses of Ringelberg et al. (2022) and Kates et al. (2024), the phylogenetic relationships of the early-diverging Mimoseae lineages might for now best be depicted as a polytomy.

#### Taxonomic references.

Hou (1996d); Stapf (1891); van Steenis (1975).

## ﻿﻿15. *Chidlowia*

Anne Bruneau^1^

Citation: Bruneau A (2024) 15. Chidlowia. In: Bruneau A, Queiroz LP, Ringelberg JJ (Eds) Advances in Legume Systematics 14. Classification of Caesalpinioideae. Part 2: Higher-level classification. PhytoKeys 240: 228–230. https://doi.org/10.3897/phytokeys.240.101716

### ﻿ Chidlowia

Taxon classificationPlantaeFabalesFabaceae

Hoyle, Bull. Misc. Inform. Kew 1932 (2): 101. 1932.

**Figure 117.**
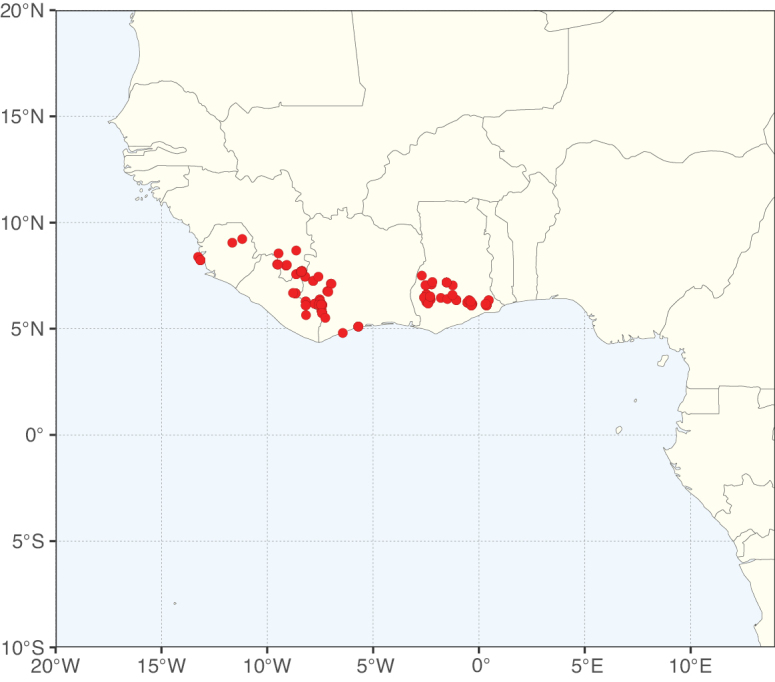
*Chidlowiasanguinea* Hoyle **A** tree with steep buttresses and twisted trunk **B** bark greyish to brownish with many lenticels, inner bark pink to reddish brown **C** parapinnate leaves with 4–6 pairs of leaflets **D** inflorescence, long and pendulous panicle **E** tip of inflorescence showing multiple buds **F** deep red flowers with long stamens and style **G** fruit, with coriaceous woody valves **H** dehisced fruit showing under-developed seed remnants. Photo credits **A–C** C Jongkind (WAG) **D–H** X van der Burgt (K).

**Figure 118.**
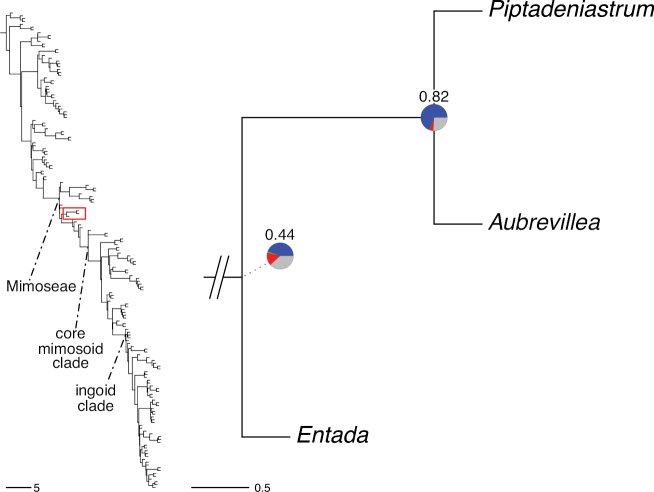
Distribution of *Chidlowia* based on quality-controlled digitised herbarium records. See Suppl. material 1 for the source of occurrence data.

Figs 114 , 117 , 118

#### Type.

*Chidlowiasanguinea* Hoyle

*Chidlowia* is here considered a monospecific lineage, whose relationship to other Mimoseae remains difficult to determine. In all phylogenetic analyses in which the genus is included, it is resolved as a distinct lineage, generally on a long branch, but strongly supported within Mimoseae (Manzanilla and Bruneau 2012; LPWG 2017; Koenen et al. 2020a; Ringelberg et al. 2022). Here it is resolved as nested between the Adenanthera and Entada clades, in a grade with another distinct lineage, *Sympetalandra* Stapf, but with no clear affinities between these two genera or with these two clades (Fig. 114).

#### Description.

Unarmed tree, small to medium sized, up to 25 (30) m (Fig. 117A) with many adventitious stems; bark very rough, grey to brown with many lenticels (Fig. 117B). **Stipules** small, caducous. **Leaves** parapinnate (Fig. 117C), up to 25 cm long; petiole terete, up to 2.5 cm long; leaflets 4–6 pairs opposite or subopposite, 4–12 × 2–5 cm. **Inflorescence** a slender, pendulous panicle (Fig. 117D, E), to 40 cm long, usually on old wood or terminal on young branchlets; lateral branchlets numerous and short, 5–7-flowered; bracts and bracteoles minute, caducous. **Flowers** deep red (Fig. 117F); pedicels slender, 3–3.5 mm; calyx campanulate, ca. 2 mm long with 5 short rounded teeth; disk fleshy, campanulate, adnate to base of calyx tube; petals free, 5 subequal, 6–7 mm long; stamens 10, partly joined at the base, red; pollen in monads, coarsely reticulate exine with foramina (Banks et al. 2003); ovary stipitate, style long and red. **Fruit** oblong-linear, acute at both ends, up to 60 cm long and 6 cm broad, valves coriaceous woody (Fig. 117G, H), dehiscing elastically along both sutures, separately spirally twisting, 9–14 (15) seeds. **Seeds** suborbicular, flat, shining red-brown, testa coriaceous; funiculus less than 1 mm long; hilum concealed by funicular remnant; pleurogram absent.

#### Chromosome number.

Unknown.

#### Included species and geographic distribution.

Monospecific (*C.sanguinea*), West tropical Africa in Ghana, Guinea, Ivory Coast, Liberia, Sierra Leone (Fig. 118).

#### Ecology.

Trees of evergreen and moist semi-deciduous Guineo-Congolian forest.

#### Etymology.

Named in honour of English silviculturist, Chidlow Vigne, who worked at the Gold Coast Forest Service, and was the first collector to recognise the distinctiveness of specimens of this species.

#### Human uses.

In Ivory Coast the wood, which is very hard and known as ‘bala’, is locally used for joinery, stakes and rifle butts (Lemmens 2010).

#### Notes.

*Chidlowia* was placed in the Dimorphandra group of tribe Caesalpinieae by Polhill and Vidal (1981), Polhill (1994) and Lewis (2005b) but resolved as part of the mimosoid clade when first sampled in a molecular phylogenetic analysis (Manzanilla and Bruneau 2012), a placement that has subsequently been confirmed with additional data (LPWG 2017; Koenen et al. 2020a; Ringelberg et al. 2022). Morphologically, members of the informal Dimorphandra group of Polhill and Vidal (1981) were known to have similarities to mimosoid legumes and the group was considered a ‘‘transitional link’’ between the caesalpinioids and mimosoids (Polhill and Vidal 1981; Luckow et al. 2000, 2003).

In *Chidlowia*, the singly pinnate leaves, relatively large flowers with showy red petals which are strongly imbricate in bud, the large explosively dehiscent woody fruits, and seeds lacking a pleurogram are all more suggestive of placement outside the mimosoids. Hoyle (1932) had suggested an affinity with the genus *Schotia* Jacq. (subfamily Detarioideae), but the regular flowers with equally sized petals, the showy red stamen filaments partly joined at the base (described as free by Hoyle (1932) in the genus protologue), and the small campanulate, gamosepalous calyx, support placement in Mimoseae. *Chidlowia* also stands out in having dorsifixed (not basifixed) anthers.

*Chidlowia* has flowers similar to those found in *Sympetalandra* with a fleshy and thick floral disc joined to the base of the calyx, petals and stamens simulating a hypanthium (Hoyle 1932). These two genera could be considered as morphologically aberrant in Mimoseae, because of the paripinnate leaves and the imbricate ascending petals. However, paripinnate leaves occur in other Mimoseae genera, such as the speciose genus *Inga* Mill. and in some species of *Zygia* P. Browne. Additionally, *Chidlowia* shares with other nodulating Mimosae a symbiosome root nodule anatomy, whereby rhizobia are symplastically retained in the host cell cytoplasm within membrane-bound symbiosomes (Faria et al. 2022).

#### Taxonomic references.

Hoyle (1932); Hutchinson and Dalziel (1958).

## ﻿﻿16. Entada clade

Shawn A. O’Donnell^36^, Gwilym P. Lewis^10^

Citation: O’Donnell SA, Lewis GP (2024) 16. Entada clade. In: Bruneau A, Queiroz LP, Ringelberg JJ (Eds) Advances in Legume Systematics 14. Classification of Caesalpinioideae. Part 2: Higher-level classification. PhytoKeys 240: 231–240. https://doi.org/10.3897/phytokeys.240.101716

**Entada clade**

Figs 119–125

**Included genera (3).***Aubrevillea* Pellegr. (2 species), *Entada* Adans. (40), *Piptadeniastrum* Brenan (1).

**Description.** Trees, shrubs, lianas and geoxylic suffrutices, unarmed (except for *Entadaspinescens* Brenan with spinescent stipules). **Stipules** inconspicuous, setaceous, or in *E.spinescens* rigid, subconical, divaricate, spinescent, or in *Piptadeniastrumafricanum* (Hook. f.) Brenan linear, densely pubescent, caducous. **Leaves** bipinnate, lacking extrafloral nectaries (except in a few species of *Entada*). **Inflorescences** spiciform racemes or spikes, axillary or terminal, sometimes clustered into fascicles or panicles. **Flowers** 5-merous, bisexual or staminate, sessile or pedicellate; calyx gamosepalous, campanulate or cupuliform; petals free or slightly united at their bases, stemonozone present; stamens (8) 10, anthers with a sessile or stipitate gland, or eglandular; pollen tricolporate, infratectum columellate, exine perforate to finely reticulate, released as monads; ovary multi-ovulate. **Fruit** papyraceous, with a basal twist, indehiscent, or coriaceous and dehiscent along one suture, or a craspedium breaking up to leave the sutures as a persistent replum. **Seeds** globular or laterally compressed, winged or not, with or without a pleurogram.

**Distribution.** Widespread across the tropics, with highest species diversity in sub-Saharan Africa (*Aubrevillea* and *Piptadeniastrum* are confined to tropical Africa), reaching subtropical latitudes in southern Africa and eastern Asia.

**Figure 119.**
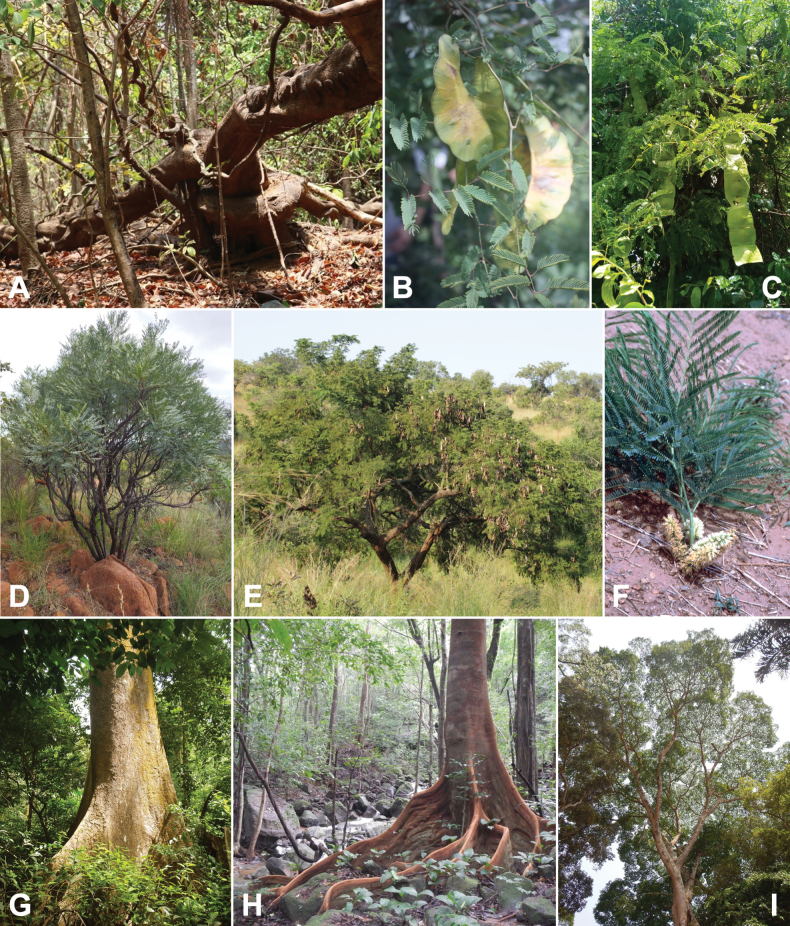
Generic relationships in the Entada clade (tribe Mimoseae). For description of phylogeny and support values, see Fig. 6 caption (page 63).

**Notes.** As then known, species of the Entada clade had been distributed by Bentham (1875) in *Entada*, *Elephantorrhiza* Benth. and *Piptadenia* Benth., and placed in Piptadenieae Benth. due to the largely shared characters of anther glands and seeds without endosperm. Brenan (1955) later erected the segregate monospecific genus *Piptadeniastrum* to accommodate, on the basis of the distinctiveness of its winged seeds and large anther glands, *Piptadeniaafricana* Hook. f. [≡ *Piptadeniastrumafricanum* (Hook. f.) Brenan], the only species of the former *Piptadenia* that is resolved as part of the Entada clade. *Aubrevillea* was first shown to be related to the Entada clade in the LPWG (2017)*matK* phylogenetic analyses.

**Clade-based definition.** The most inclusive crown clade containing *Entadaphaseoloides* (L.) Merr. and *Piptadeniastrumafricanum* (Hook. f.) Brenan, but not *Chidlowiasanguinea* Hoyle, *Pentaclethramacroloba* (Willd.) Kuntze or *Prosopisafricana* (Guill. & Perr.) Taub. (Fig. 119).

Hutchinson (1964) departed slightly from Bentham (1875) by dividing the genera with valvate aestivation and 10 or fewer stamens into tribes Mimoseae Bronn and Adenanthereae Benth. based upon the lack or presence of anther glands, respectively, and thus subsuming Piptadenieae into the latter. Accordingly, Hutchinson (1964) placed *Aubrevillea* in Mimoseae, and *Piptadeniastrum*, *Elephantorrhiza* and *Entada* in Adenanthereae. Elias (1981a) and Lewis and Elias (1981) sunk Adenanthereae (sensu Hutchinson 1964, i.e., including Piptadenieae) into a broadened Mimoseae with the view that presence of an anther gland was an unsatisfactory character for tribal delineation, and organised the constituent genera into 12 informal groups. In Lewis and Elias’s (1981) classification of tribe Mimoseae, *Aubrevillea* was thought to be “basal” due to the presence of a hypanthium, a character shared with *Dinizia* Ducke, which was considered by Lewis and Elias (1981) to be another basal member of Mimoseae (*Dinizia* now placed outside the Mimoseae, in tribe Campsiandreae, page 187). *Piptadeniastrum* was placed by Lewis and Elias (1981) in their Newtonia group, though the genus was the only member of the group lacking foliage glands. *Entada* and *Elephantorrhiza* formed the Entada group, unified by shared craspedial fruits, usually opposite leaflets, presence of a stemonozone, pollen dispersed as monads and tubular stigmas.

A sister relationship between *Piptadeniastrumafricanum* and the Entada group, rather than with the Newtonia group as Lewis and Elias (1981) had postulated, was recovered in Luckow et al.’s (2003) duo-locus plastid phylogeny of 134 mimosoid taxa, albeit with low bootstrap support and few clear morphological links. Similarly, Lewis and Elias’s (1981) isolated placement of *Aubrevillea* as close to non-mimosoid genera in Caesalpinioideae, exemplifies the difficulty in identifying possible morphological synapomorphies for the Entada clade. That said, the genera of the Entada clade all share the widespread mimosoid traits of bipinnate leaves and valvate aestivation. With the exception of *Entadaspinescens*, the genera of the Entada clade also lack armature, all species bear flowers in spikes or spiciform racemes, disperse their pollen as monads and, apart from a subset of species of *Entada* in Madagascar, lack foliage glands.

### ﻿ Entada

Taxon classificationPlantaeFabalesFabaceae

Adans., Fam. Pl. 2: 318. 1763.

**Figure 120.**
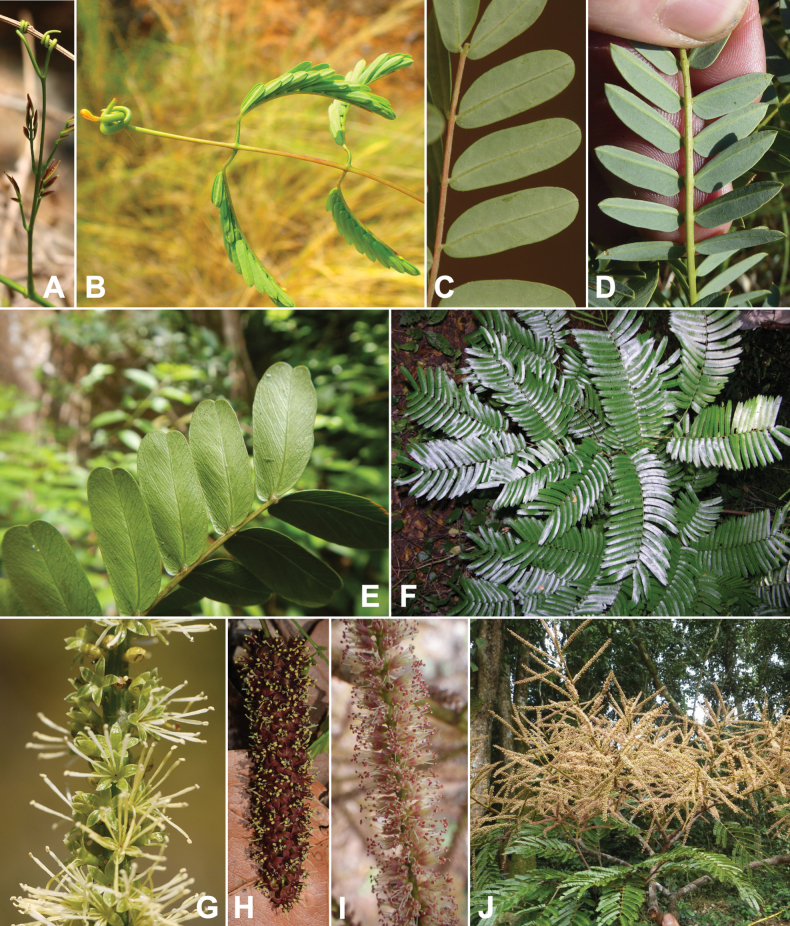
Entada clade variation in habit **A***Entadarheedei* Spreng., huge woody liana, India **B***Entadawahlbergii* Harv., thin woody climber in fruit, Benin **C***Entadaleptostachya* Harms, scandent shrub in fruit, Madagascar **D***Entadaburkei* (Benth.) S.A. O’Donnell & G.P. Lewis, erect shrub, South Africa **E***Entadaabyssinica* Steud. ex A. Rich., small tree, Rwanda **F***Entadaelephantina* (Burch.) S.A. O’Donnell & G.P. Lewis, geoxylic suffrutex, South Africa **G***Aubrevilleaplatycarpa* Pellegr., large tree with plank buttresses, Guinea **H***Piptadeniastrumafricanum* (Hook.f.) Brenan, large tree with aliform buttresses, Guinea **I***P.africanum*, large tree in forest canopy, Sierra Leone. Photo credits **A** Shiwali Samant, iNaturalist (https://www.inaturalist.org/photos/58810243) **B** M Schmidt, Dressler et al. (2014)**C** merveille, iNaturalist (https://www.inaturalist.org/photos/102007815) **D** pete_leroux, iNaturalist (https://www.inaturalist.org/photos/184645340) **E** jordivanoort, iNaturalist (https://www.inaturalist.org/photos/31303989) **F** AE van Wyk **G–I** X van der Burgt.

**Figure 121.**
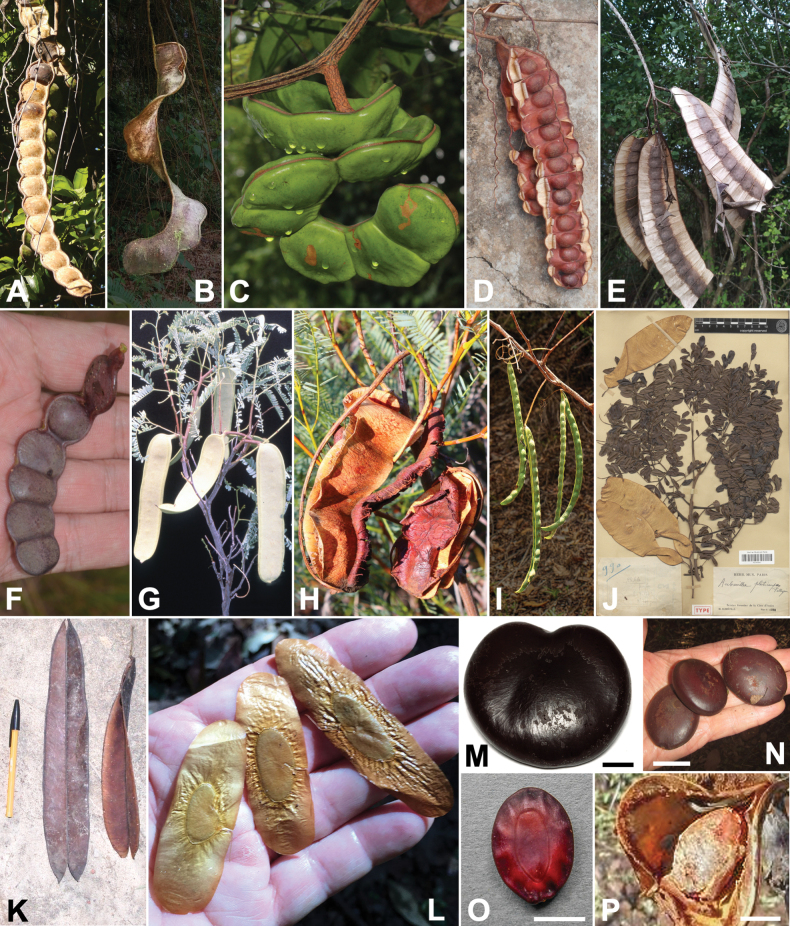
Entada clade variation in foliage and flowers **A***Entadarheedei* Spreng., young bipinnate leaf terminating in a bifurcate tendril (modified terminal pinnae pair), India **B***Entadatuberosa* R. Vig., bipinnate leaf terminating in a bifurcate, thickened tendril (modified terminal pinnae pair), and rachis and rachillae terminating in a yellowish glandular mucro, Madagascar **C***Entadamannii* (Oliv.) Tisser., oblong leaflets with rounded apices and midvein positioned centrally, Republic of Congo **D***Entadaobliqua* (Burtt Davy) S.A. O’Donnell & G.P. Lewis, asymmetric leaflets with acute apices and midvein positioned closer to distal margin, South Africa **E***Aubrevilleaplatycarpa* Pellegr., oblong-obovate leaflets with emarginate apices, Guinea **F***Piptadeniastrumafricanum* (Hook.f.) Brenan, finely divided bipinnate leaves, Benin **G***E.rheedei*, subsessile flowers with greenish-yellow corollas and cream-coloured stamen filaments, India **H***Entadastuhlmannii* (Taub.) Harms, spiciform raceme of flowers with deep red corolla and stamen filaments, Mozambique **I***P.africanum*, spiciform raceme of flowers with yellowish-white corolla (base of petals tinged pinkish) and stamen filaments, and red anthers with a white apical anther gland, Republic of Congo **J***P.africanum*, terminal panicles of spiciform racemes, Republic of Congo. Photo credits **A** Shiwalee Sawant, iNaturalist (https://www.inaturalist.org/photos/58810126) **B** Andry.A.R, iNaturalist (https://www.inaturalist.org/photos/30264619) **C** DJ Harris, Dressler et al. (2014)**D** Andrew Hankey, iNaturalist (https://www.inaturalist.org/photos/3213785) **E** X van der Burgt **F** G Georgen, Dressler et al. (2014)**G** Dinesh Valke, iNaturalist (https://www.inaturalist.org/photos/133409879) **H** © Warren McCleland, all rights reserved, iNaturalist (https://www.inaturalist.org/photos/152390385) **I–J** X van der Burgt.

**Figure 122.**
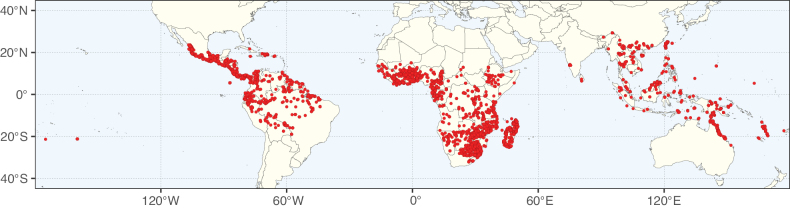
Entada clade variation in fruits and seeds **A***Entadarheedei* Spreng., mature, gigantic, torulose, slightly curved, segmented craspedium with woody endocarp, India **B***Entadagigas* (L.) Fawc. & Rendle, mature, laxly spirally twisted, segmented craspedium, Costa Rica **C***Entadaspiralis* Ridl., immature, tightly spirally twisted, segmented craspedium, Singapore **D***Entadaafricana* Guill. & Perr., mature, segmented craspedia, distinctly umbonate over seeds, with exocarp peeling away, Togo **E***Entadapolystachya* (L.) DC., mature, segmented craspedia, slightly umbonate over seeds, with exocarp already shed, Costa Rica **F***Entadadolichorrhachis* Brenan, mature, small, torulose, slightly curved, segmented craspedium, Zambia **G***Entadaburkei* (Benth.) S.A. O’Donnell & G.P. Lewis, immature craspedia, not segmented, South Africa **H***E.burkei*, mature craspedia, not segmented, entire valves breaking away from replum, exocarp peeling away, South Africa **I***Entadagoetzei* (Harms) Harms, immature, elongate craspedia, not segmented, distinctly umbonate over seeds, Mozambique **J***Aubrevilleaplatycarpa* Pellegr., papery, indehiscent fruits with twisted bases (holotype *A Aubreville 990*, MNHN-P-P00418246), Côte d’Ivoire **K***Piptadeniastrumafricanum* (Hook.f.) Brenan, coriaceous pods, dehiscent along single suture, Democratic Republic of Congo **L***P.africanum*, flattened, oblong seeds surrounded by broad, membranous wing, with funicle attached at middle of long axis of seed, Uganda **M***E.gigas*, large, laterally compressed, cordate seed without pleurogram, collected in beach wrack, USA **N***E.rheedei*, large, laterally compressed, globular seeds without pleurogram, South Africa **O***E.africana*, laterally compressed, elliptic seeds with closed pleurogram, Togo **P***E.burkei*, globular seed without pleurogram, South Africa. Scale bars: 1 cm (**M**); 2 cm (**N**); 5 mm (**O, P**). Photo credits **A** Dinesh Valke, iNaturalist (https://www.inaturalist.org/photos/159405372) **B** Pedro Blanco, iNaturalist (https://www.inaturalist.org/photos/181372721) **C** Cerlin Ng CC BY-NC-SA 2.0 **D** B Eichhorn, Dressler et al. (2014)**E** Marvin López M, iNaturalist (https://www.inaturalist.org/photos/181531329) **F** W McCleland, Dressler et al. (2014)**G** P van Wyk; **H** tjeerd, iNaturalist (https://www.inaturalist.org/photos/64689073) **I** BT Wursten, Hyde et al. (2022)**J** MNHN (2022) (CC BY 4.0) **K** P Latham, Dressler et al. (2014)**L** David Bygott, iNaturalist (https://www.inaturalist.org/photos/62615168) **M** Robb Deans, iNaturalist (https://www.inaturalist.org/photos/2611187) **N** Ricky Taylor, iNaturalist (https://www.inaturalist.org/photos/44440338) **O** B Eichhorn, Dressler et al. (2014)**P** Joseph Heymans, iNaturalist (https://www.inaturalist.org/photos/123571608).

**Figure 123.**
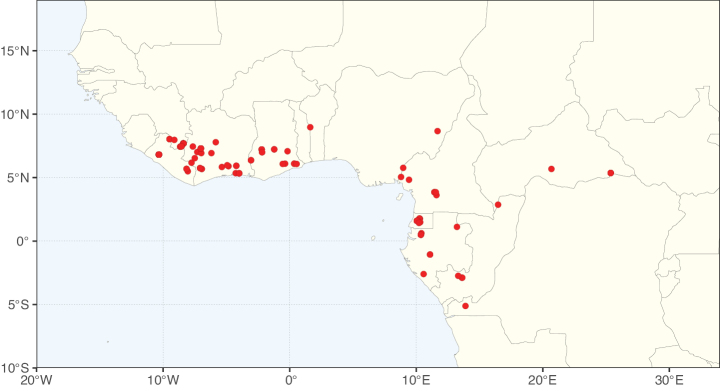
Distribution of *Entada* based on quality-controlled digitised herbarium records. See Suppl. material 1 for the source of occurrence data.

Figs 120 , 121 , 122 , 123

Gigalobium P. Browne, Civ. Nat. Hist. Jamaica: 362. 1756, nom. rej. vs. Entada Adans.. Lectotype (designated by Panigrahi in Taxon 34 : 714. 1985): Entadagigas (L.) Fawc. & Rendle [≡ Mimosagigas L.]
Perima Raf., Sylva Tellur.: 118. 1838. Type: Perimaodorata Raf., nom. illeg. [= Mimosascandens L. (= Entadaphaseoloides (L.) Merr.)]
Strepsilobus Raf., Sylva Tellur.: 117. 1838. Type: Strepsilobusscandens (L.) Raf. [≡ Mimosascandens L. (= Entadaphaseoloides (L.) Merr.)]
Elephantorrhiza Benth., J. Bot. (Hooker) 4: 344. 1841. Type: Elephantorrhizaburchellii Benth., nom. illeg. [≡ Acaciaelephantorrhiza Burch. ex DC., nom. illeg. (= Entadaelephantina (Burch.) S.A. O’Donnell & G.P. Lewis)]
Pusaetha L. ex Kuntze, Revis. Gen. Pl. 1: 204. 1891. Type: Pusaethascandens (L.) Kuntze [≡ Mimosascandens L. (= Entadaphaseoloides (L.) Merr.)]
Entadopsis Britton, N. Amer. Fl. 23: 191. 1928. Type: Entadopsispolystachya (L.) Britton [≡ Mimosapolystachya L. (≡ Entadapolystachya (L.) DC.)]

#### Type.

*Entadamonostachya* DC., nom. illeg. [≡ *Mimosaentada* L. (≡ EntadarheedeiSpreng.subsp.rheedei)]

#### Description.

Lianas (Fig. 120A), scandent shrubs (Fig. 120C), small trees (Fig. 120E) or geoxylic suffrutices (Fig. 120F), unarmed except for spinescent stipules in *E.spinescens*. **Stipules** inconspicuous, setaceous, or in *E.spinescens* rigid, subconical, divaricate, spinescent, 1.5–3.5 mm long. **Leaves** (Fig. 121A–D) bipinnate; primary and secondary axes either eglandular or, in some Madagascan species, with extrafloral nectaries and at least in *E.phaseoloides*, with unusual ‘pit’ nectaries on stems at nodes adjacent to petiole; rachis in lianescent taxa terminating in a bifurcating tendril (modified terminal pinnae pair) (Fig. 121A); pinnae 1–many pairs per leaf; leaflets 1–many pairs per pinna, lamina often asymmetric and apically mucronate or emarginate. **Inflorescence** (Fig. 121G, H) spiciform racemes or spikes, axillary to supra-axillary, solitary or clustered, sometimes into terminal panicles. **Flowers** (Fig. 121G, H) sessile to shortly pedicellate, 5-merous, staminate or bisexual, cream-coloured, yellow, green, red or purple; calyx gamosepalous, campanulate, the fused sepals distinctly toothed or not; petals free to basally connate, adnate basally with the stamens and a perigynous disc forming a stemonozone; stamens 10, fertile, free or basally united, anthers usually with a caducous spheroidal, apical, sessile to stipitate gland; pollen tricolporate, tectum finely reticulate to striate, infratectum columellate, dispersed as monads; ovary glabrous and multi-ovulate, style tapering to a tubular to rarely cupuliform stigma. **Fruit** (Fig. 122A–I) a craspedium, torulose or not, compressed to flattened, straight to curved to rarely spirally twisted, sometimes gigantic (up to 2 m long in taxa with sea-drifted seeds); epicarp woody to thinly coriaceous; endocarp woody to parchment-like; splitting along transverse septa into one-seeded segments upon ripening or valvately dehiscent, the entire valve breaking away from the replum and the epicarp also separating from the endocarp. **Seeds** (Fig. 122M–P) globular to elliptic, usually laterally compressed, longest axis up to 6 cm in large-fruited taxa, dark brown, smooth, with or without areole, pleurogram (when present) usually open.

#### Chromosome number.

2*n* = 28 (Santos et al. 2012).

#### Included species and geographic distribution.

Forty species, widespread, primarily tropical, but reaching subtropical latitudes in southern Africa and eastern Asia (Fig. 123). Twenty-nine species in sub-Saharan Africa (including six species in Madagascar, three of which are endemic); nine species in Asia; four species in the Americas. Three species with large fruits and seeds are very widely distributed by ocean currents (*E.rheedei* circum-Indian Ocean, South East Asia and northern Australia; *E.gigas* in Central and northern South America to west and central Africa; *E.phaseoloides* from East and South East Asia to north-east Australia and south-west Pacific Ocean islands).

#### Ecology.

Frequently in riparian and littoral vegetation including at the landward fringes of mangroves, though also in savanna, open woodland, thickets and open, dry to dense, humid forest, often on sandy substrates.

#### Etymology.

Likely from the indigenous name for the plant in Malabar, India (Luckow 2005); the alternative derivation of the name given in the same publication seems less likely and is omitted here.

#### Human uses.

Seeds and bark of several species are ground and used in traditional medicines, as soap, and as fish poison; leaves and detoxified seeds are eaten as famine food (Lungu 1995). Tannins in roots of species formerly included in *Elephantorrhiza* are used in tanning leather and as dye (Grobler 2012). Various species are used as fodder; for fibre; for firewood and charcoal; large, sea-drifted seeds are used in jewellery (Luckow 2005).

#### Notes.

The study of Luckow et al. (2003) was the first to suggest that *Elephantorrhiza* might be phylogenetically nested within *Entada*, a relationship that has subsequently received robust molecular support (LPWG 2017; Koenen et al. 2020a; Ringelberg et al. 2022). Based on these results, all eight species previously placed in *Elephantorrhiza* were subsumed within *Entada* by O’Donnell et al. (2022).

#### Taxonomic references.

Barneby (1996); Braga et al. (2016); Brenan (1959, 1966, 1970); Cowan (1998); Grobler (2012); Lungu (1995); Nielsen (1981a, 1992); O’Donnell et al. (2022); Ohashi et al. (2010); Ross (1974, 1975a); Tateishi et al. (2008); Villiers (2002); Wu and Nielsen (2010).

### ﻿ Aubrevillea

Taxon classificationPlantaeFabalesFabaceae

Pellegr., Bull. Soc. Bot. France 80: 466. 1933.

**Figure 124.**
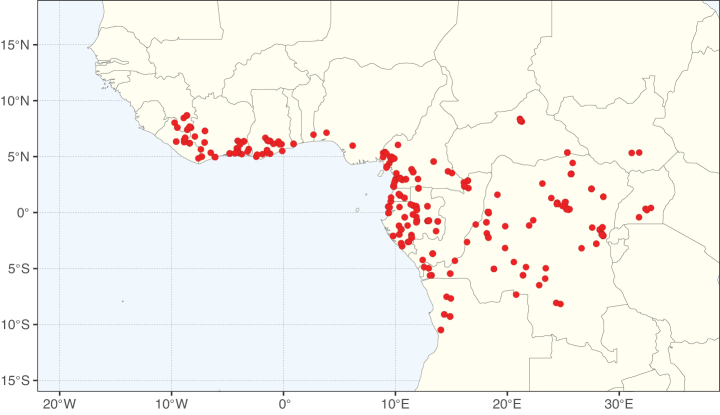
Distribution of *Aubrevillea* based on quality-controlled digitised herbarium records. See Suppl. material 1 for the source of occurrence data.

Figs 120 , 121 , 122 , 124

#### Type.

*Aubrevilleakerstingii* (Harms) Pellegr. [≡ *Piptadeniakerstingii* Harms]

#### Description.

Tall trees (Fig. 120G), unarmed. **Stipules** inconspicuous, setaceous. **Leaves** bipinnate, eglandular; pinnae 4–8 pairs per leaf; leaflets 8–30 opposite pairs per pinna, sessile, oblong–oblanceolate or falcate, apex often emarginate, base asymmetric (Fig. 121E). **Inflorescence** a panicle of spiciform racemes, terminal or axillary. **Flowers** shortly pedicellate, bisexual, pale green to pale yellow or white; calyx gamosepalous, cupuliform, shallowly toothed, puberulous; hypanthium as long as the calyx tube; petals lanceolate, basally connate, puberulous outside, adnate basally with the stamens and a perigynous disc forming a stemonozone; stamens (8–)10, fertile, filaments basally connate, anthers eglandular; pollen tricolporate, finely reticulate, dispersed as monads; ovary villose, ovules 5–7, style short, stigma widely porate. **Fruit** (Fig. 122J) laterally compressed, papyraceous, indehiscent, oblong, its proximal end twisted. **Seeds** flat, reniform, lacking endosperm.

#### Chromosome number.

Unknown.

#### Included species and geographic distribution.

Two species (*A.kerstingii* and *A.platycarpa* Pellegr.) from tropical west and central Africa, from Liberia and Guinea east to eastern Central African Republic and south to the Democratic Republic of Congo (Fig. 124).

#### Ecology.

Rainforest, with outliers in seasonally dry Sudanian woodland and wooded grassland.

#### Etymology.

Named for Prof. A. Aubreville (1897–1982), a noted French forester, ecologist and taxonomist (Luckow 2005).

#### Human uses.

Used in traditional medicines, for timber, and as a shade tree (Luckow 2005).

#### Notes.

Pellegrin (1933) established the genus *Aubrevillea* to accommodate a newly described tree with indehiscent, papery fruits with a twisted base from Côte d’Ivoire, *A.platycarpa*, and proposed the new combination *A.kerstingii* for a tree with similar fruits from Togo and Côte d’Ivoire in light of new fruiting material that Harms (1907) had not seen when he tentatively placed this taxon in *Piptadenia*.

#### Taxonomic references.

Villiers (1989).

### ﻿ Piptadeniastrum

Taxon classificationPlantaeFabalesFabaceae

Brenan, Kew Bull. 10: 179. 1955.

**Figure 125.**
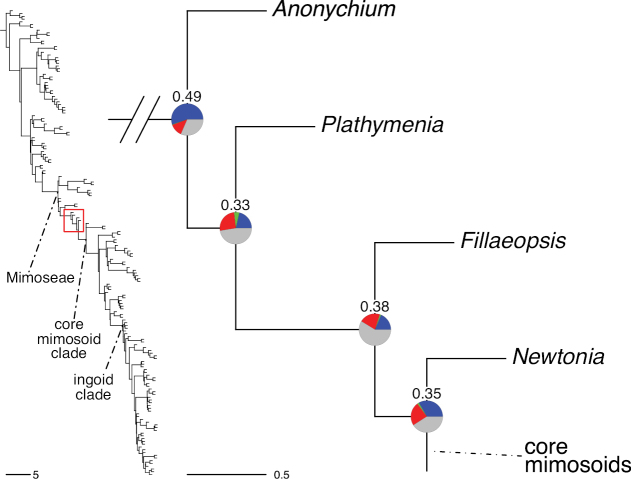
Distribution of *Piptadeniastrum* based on quality-controlled digitised herbarium records. See Suppl. material 1 for the source of occurrence data.

Figs 120 , 121 , 122 , 125

#### Type.

*Piptadeniastrumafricanum* (Hook. f.) Brenan [≡ *Piptadeniaafricana* Hook. f.]

#### Description.

Tall trees, unarmed, with ramified, aliform buttresses often more than 3 m high (Fig. 120H, I). **Stipules** linear, densely pubescent, 5.5–9 mm long, apex sharp, caducous. **Leaves** (Fig. 121F) bipinnate, eglandular; pinnae often alternate, 10–19 (23) pairs per leaf; leaflets (26) 30–58 (61) pairs per pinna, sessile, linear or falcate, apex obtuse, base asymmetric. **Inflorescence** (Fig. 121I, J) a panicle of fascicled spiciform racemes, terminal. **Flowers** (Fig. 121I) pedicellate with abscission zone near pedicel apex, bisexual, yellow or yellowish-white; calyx gamosepalous, cupuliform, distinctly toothed; petals free, glabrous, adnate basally with the stamens and a perigynous disc forming a stemonozone; stamens 10, filaments red, anther connective terminating in a large globular, sessile, caducous gland; pollen tricolporate, finely reticulate, dispersed as monads; ovary glabrous, red, ovules 9, style slender, stigma porate and slightly dilated. **Fruit** (Fig. 122K) flattened, straight to slightly curved, dehiscent along a single suture, the valves remaining attached along the other, exocarp coriaceous. **Seeds** (Fig. 122L) flattened, surrounded by a broad membranous wing, oblong, funicle inserted near the middle of a long margin.

#### Chromosome number.

2*n* = 26, diploid (Lewis and Elias 1981; Santos et al. 2012).

#### Included species and geographic distribution.

One species, *P.africanum*, in tropical Africa, from Liberia and Guinea east to South Sudan and Uganda, and south to Angola (Fig. 125).

#### Ecology.

Rainforest and in riparian vegetation within more seasonally dry tropical forest.

#### Human uses.

Used for commercial timber, charcoal, fish and rodent poison, in traditional medicines and in rituals (Luckow 2005).

#### Notes.

In his revision of the African members of *Piptadenia* (sensu Baker 1930), Brenan (1955) created the segregate monospecific genus *Piptadeniastrum* to house *Piptadeniastrumafricanum*, a tall, unarmed tree from west-central sub-Saharan Africa reminiscent of *Newtonia* Baill. in its fruits that dehisce along one suture while the valves remain attached along the other, and in its winged seeds, though differing in its often-alternate pinnae, glabrous floral structures and point of funicular attachment at the middle edge of the longest axis of the seed.

#### Taxonomic references.

Brenan (1959); Villiers (1989).

## ﻿﻿17. Newtonia grade

Colin E. Hughes^3^, Melissa Luckow^29^, Gwilym P. Lewis^10^

Citation: Hughes CE, Luckow M, Lewis GP (2024) 17. Newtonia grade. In: Bruneau A, Queiroz LP, Ringelberg JJ (Eds) Advances in Legume Systematics 14. Classification of Caesalpinioideae. Part 2: Higher-level classification. PhytoKeys 240: 241–249. https://doi.org/10.3897/phytokeys.240.101716

**Newtonia grade**

Figs 126–131

**Included genera (4).***Anonychium* (Benth.) Schweinf. (1 species), *Fillaeopsis* Harms (1), *Newtonia* Baill. (11), *Plathymenia* Benth. (1).

**Figure 126.**
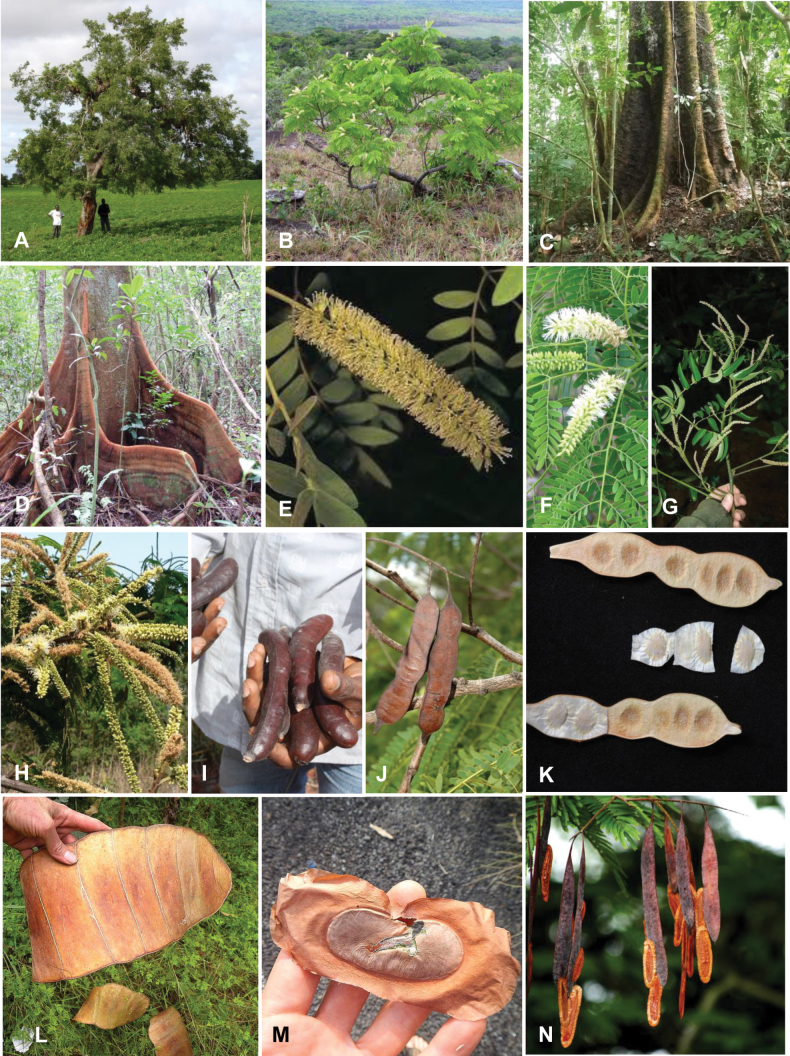
Phylogenetic relationships across the grade Newtonia of four genera subtending the core mimosoid clade in tribe Mimoseae. For description of phylogeny and support values, see Fig. 6 caption (page 63).

The genera *Anonychium*, *Plathymenia*, *Fillaeopsis* and *Newtonia*, the first three monospecific, and the four together comprising a total of 14 species, form a grade with respect to the core mimosoid clade (Figs 5, 126). This paraphyly, with the genus *Newtonia* as sister to the core mimosoid clade (Ringelberg et al. 2022; Fig. 126), has been seen in previous phylogenies (Catalano et al. 2008; Koenen et al. 2020a). In the absence of any additional phylogenetic structure, these four genera remain unplaced in any named terminal clade and are here presented in the order in which they appear in the phylogeny.

All four of these genera comprise unarmed trees, the plesiomorphic condition in Mimoseae (Ringelberg et al. 2022). An evolutionary shift from unarmed to armed occurs on the branch subtending the core mimosoid clade, where genera and species of the first-branching lineages of that clade, i.e., *Cylicodiscus* Harms, plus the Prosopis and Neltuma clades, are all armed, and as such, the core mimosoid clade represents the first evolution of armature along the phylogenetic backbone of tribe Mimoseae (Koenen et al. 2020a; Ringelberg et al. 2022).

### ﻿ Anonychium

Taxon classificationPlantaeFabalesFabaceae

(Benth.) Schweinf., Reliq. Kotschy.: 7. 1868.

Figs 127 , 128

Prosopis sect. Anonychium Benth., J. Bot. (Hooker) 4: 347. 1841. Type: Prosopisoblonga Benth. [= Anonychiumafricanum (Guill. & Perr.) C.E. Hughes & G.P. Lewis]

#### Type.

*Anonychiumlanceolatum* (Benth.) Schweinf. [≡ *Prosopislanceolata* Benth. (= *Anonychiumafricanum* (Guill. & Perr.) C.E. Hughes & G.P. Lewis)]

#### Description.

**Figure 127.**
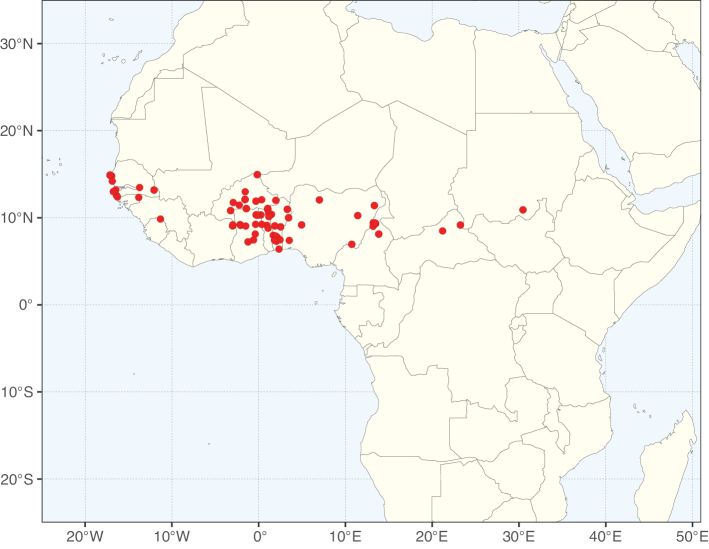
Habit, habitats, inflorescences, and fruits of *Newtonia* and allies **A** small tree of *Anonychiumafricanum* (Guill. & Perr.) C.E. Hughes & G.P. Lewis in Senegal, West Africa **B** small twisted treelet of *Plathymeniareticulata* Benth. in savanna grasslands (Cerrado) in eastern Bolivia **C** large, buttressed tree base of *Fillaeopsisdiscophora* Harms in tropical wet forest, Ebo, Cameroon **D** large buttressed tree base of *Newtoniabuchananii* (Baker f.) G.C.C. Gilbert & Boutique in semi-evergreen forest in Mozambique **E** spicate inflorescence of *Anonychiumafricanum***F** spicate inflorescences of *Plathymeniareticulata*, eastern Bolivia **G** spicate inflorescences of *Fillaeopsisdiscophora*, Gabon **H** spicate inflorescences of *Newtoniabuchananii* in Malawi **I** indehiscent pods of *Anonychiumafricanum* collected as livestock feed in Burkina Faso, West Africa **J** tardily dehiscent pods with chartaceous or weakly coriaceous valves of *Plathymeniareticulata*, eastern Bolivia **K** pod valves and papery endocarp packets surrounding seeds of *Plathymeniareticulata*, Bahia, Brazil **L** tardily dehiscent pods with chartaceous valves of *Fillaeopsisdiscophora* in tropical wet forest in Mayombe, Congo (Brazzaville) **M** large winged seeds of *Fillaeopsisdiscophora* in tropical wet forest in Ambam, Cameroon **N** fruits and winged seeds of *Newtoniabuchananii* in cultivation in South Africa. Photo credits **A** S Christensen www.westafricanplants.senckenberg.de**B, F, J, K, N** CE Hughes **C, L, M** X van der Burgt **D** Stefaan Dondeyne **E** M Arbonnier https://agritrop.cirad.fr/**G** E Bidault, Missouri Botanical Garden, http://legacy.tropicos.org/Image/100618759**H** G Baumann www.westafricanplants.senckenberg.de**I** M Schmidt www.westafricanplants.senckenberg.de.

Unarmed trees 4–20 m high (Fig. 127A), brachyblasts absent. **Stipules** inconspicuous, caducous as young leaves develop. **Leaves** somewhat pendulous, bipinnate; fleshy, cup or tub-shaped, porate extrafloral nectaries between most pinnae pairs and similar, but smaller, nectaries often between some or most leaflet pairs; pinnae 1–4 pairs; leaflets 4–13 pairs per pinna, opposite, mid-vein sub-centric. **Inflorescences** spiciform racemes, solitary or in pairs in axils of coevally developing leaves, densely flowered (Fig. 127E); pedicels 0.5 mm. **Flowers** small, yellowish or greenish-white, sweetly scented; calyx gamosepalous, ca. 1 mm long; petals 5, free, glabrous; stamens 10, anthers apically broadened with an anther gland borne ventrally between the thecae forming a triangular hood-shaped protrusion made up of papillate cells; pollen in tricolporate monads with costae on the pores and a smooth (perforated) exine with columellae; ovary sessile. **Fruits** indehiscent, straight or sub-falcate, dark reddish-brown to blackish, shiny, subterete in cross-section (Fig. 127I), exocarp hard, 1–2 mm thick, mesocarp spongy, thick, dry, endocarp segmented, segments enclosing the seeds, thin, transverse (at right angles to fruit length), in one row, seeds many. **Seeds** dark brown to black, compressed, rattling within the pod when ripe, pleurogram present 75%, testa hard.

#### Chromosome number.

2*n* = 28 (Bukhari 1997).

#### Included species and geographic distribution.

**Figure 128.**
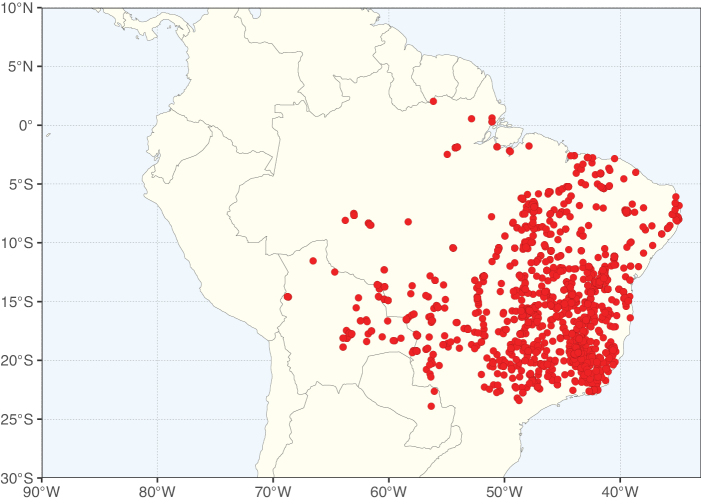
Distribution of *Anonychium* based on quality-controlled digitised herbarium records. See Suppl. material 1 for the source of occurrence data.

Monospecific (*A.africanum*), widespread across Sahelian Africa, from Senegal in the west to Sudan in the east (Fig. 128).

#### Ecology.

Native across the whole Sahelian savanna belt. Seeds dispersed by herbivores.

#### Etymology.

*Anonychium* from the Latin or Greek *onych* = *ónyx* (= nail or claw), meaning the absence of nails or claws, in reference to the lack of armature of this genus.

#### Human uses.

Trees of *Anonychium* are maintained and managed by farming and pastoralist communities in traditional silvo-pastoral and agroforestry systems throughout the African Sahel (Figs 127A, 128), providing essential products, including wood, fuel, food, livestock fodder and medicines and enhancing soil fertility (Leakey and Last 1980). The highly nutritious indehiscent fruits are eagerly consumed by large herbivores including all forms of livestock, such as camels, cattle and goats at the end of the dry season and harvested by farmers to feed to their animals; cow dung (containing viable seeds) is used to fertilise fields.

#### Notes.

The genus *Anonychium* was resurrected by Hughes et al. (2022b), along with the genera *Strombocarpa* Engelm. & A. Gray and *Neltuma* Raf., to account for the non-monophyly of the genus *Prosopis* L. Prior to that, *Prosopisafricana* (Guill. & Perr.) Taub. had long been considered anomalous within *Prosopis*, having been previously placed in its own genus, *Anonychium* by Schweinfurth (1868) under the name *Anonychiumlanceolatum* Schweinf. and, later, its own section Anonychium of *Prosopis* (Bentham 1841b; Burkart 1976). Unlike *Prosopis* and its other segregate genera *Strombocarpa* and *Neltuma*, *Anonychium* lacks armature, has internally glabrous petals, pollen with costae, V-shaped anthers with small stomia forming short pockets on the ventral surface of the anthers and unusual sessile anther glands borne ventrally between the thecae, rather than stipitate glands borne apically or dorsally from the connective between the thecae as in most other Mimoseae, and forming triangular hood-shaped protrusions made up of papillate cells which are also unique amongst mimosoid anther glands (Luckow and Grimes 1997). This distinctive combination of morphological features separating *Anonychium* from *Prosopis* is reflected in the phylogenetic placement of *Anonychium* as an isolated lineage subtending the grade of other unarmed genera, *Plathymenia*, *Fillaeopsis* and *Newtonia*, which together are paraphyletic with respect to the core mimosoid clade (Fig. 126; Ringelberg et al. 2022).

#### Taxonomic references.

Bentham (1841b); Brenan (1959), with illustration; Burkart (1976); Hughes et al. (2022b); Luckow and Grimes (1997).

### ﻿ Plathymenia

Taxon classificationPlantaeFabalesFabaceae

Benth., J. Bot. (Hooker) 4: 333. 1841.

Figs 127 , 129

Pirottantha Speg., Anales Soc. Ci. Argent. 82: 226. 1916 (publ. 1917). Type: Pirottanthamodesta Speg. [= Plathymeniareticulata Benth.]

#### Type.

*Plathymeniareticulata* Benth.

#### Description.

Unarmed deciduous trees (Fig. 127B), 2.5–12 (40) m; stems dark, terete, glabrous to glaucous, waxy; brachyblasts absent. **Stipules** rudimentary or caducous. **Leaves** bipinnate; extrafloral nectary absent or occasionally with an inconspicuous lump on the petiole; pinnae 3–10 pairs, opposite, sub-opposite or distinctly alternate, terminating in an adaxial, crateriform nectary; leaflets 6–20 per pinna, mostly alternate, conspicuously brochidodromous. **Inflorescences** spiciform racemes in leaf axils or more frequently above the axils (Fig. 127F). **Flowers** pedicellate, pedicels persistent and peg-like after abscission of unfertilised flowers; calyx 5-toothed, campanulate; petals 5, pale green, glabrous or sparsely pubescent on the apex, apparently separate to base; stamens 10, filaments white, free to the base, connective somewhat enlarged, anther glands large, apical, stipitate, round; pollen in tricolporate monads with a smooth (perforated) exine with columellae; ovary stipitate, stigma tubular. **Fruits** linear-oblong, tardily dehiscent through both sutures, strongly dorsiventrally flattened, the valves coriaceous (Fig. 127J), endocarp chartaceous forming membranous rectangular packets around the seeds, separating from the valves at maturity, and dispersed with the seeds enclosed (Fig. 127K). **Seeds** dorsiventrally flattened, the testa hard, pleurogram present, closed.

#### Chromosome number.

2*n* = 26 (Goldblatt 1981b).

#### Included species and geographic distribution.

**Figure 129.**
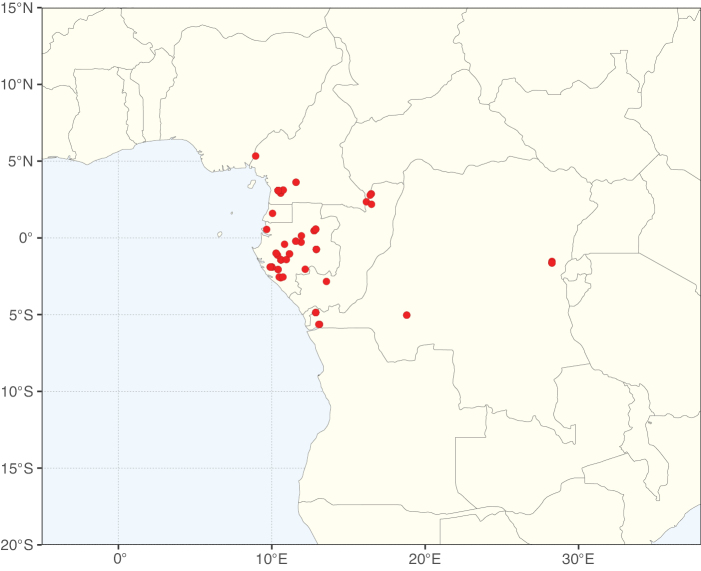
Distribution of *Plathymenia* based on quality-controlled digitised herbarium records. See Suppl. material 1 for the source of occurrence data.

Monospecific (*P.reticulata*), restricted to South America, primarily in central and eastern Brazil but extending to east-central Paraguay, Bolivia, and southern Suriname (Fig. 129).

#### Ecology.

Mainly in deciduous rocky cerrado (savanna) (Fig. 127B) and sparse cerradão woodland, extending weakly into wetter coastal Mata Atlântica forests and seasonally dry tropical forests, mainly 300–1100 m elevation.

#### Etymology.

From Greek, *platy* (= flat) and *hymen* (= membrane), in reference to the membranous endocarp breaking up into papery square envelopes in which the seeds are dispersed (Fig. 127K).

#### Human uses.

The timber of *P.reticulata* is used for furniture and fence posts. The bark has been used medicinally (Warwick and Lewis 2003).

#### Notes.

*Plathymenia* forms its own monogeneric (and monospecific) lineage embedded in the grade that subtends the core mimosoid clade (Fig. 126). In fruit, *Plathymenia* is unusual among Mimoseae in its endocarp forming papery packets around the seeds (Fig. 127K). Previously two species were recognised in the genus, however Warwick and Lewis (2003) concluded that there is only a single polymorphic species, noting that the various morphological variants do not correlate with geography or ecology.

#### Taxonomic references.

Bentham (1876); Warwick and Lewis (2003), both with illustrations.

### ﻿ Fillaeopsis

Taxon classificationPlantaeFabalesFabaceae

Harms, Bot. Jahrb. 26: 258. 1899.

Figs 127 , 130

#### Type.

*Fillaeopsisdiscophora* Harms

#### Description.

Large, often buttressed, trees 24–40 m, 40–80 (100) cm diameter (Fig. 127C), unarmed, bark smooth with vertical fissures, slash exuding pale yellow sticky latex, brachyblasts absent. **Stipules** not observed. **Leaves** bipinnate, extrafloral nectaries absent; pinnae 1–3 pairs, opposite; leaflets alternate, 4–6 (10) per pinna, venation brochidodromous. **Inflorescence** a panicle of spiciform racemes, axillary or terminal (Fig. 127G), flowers abscising to leave peg-like pedicels. **Flowers** very waxy and shiny on herbarium sheets; calyx broadly open, 5-lobed, probably somewhat succulent; petals 5, fleshy, free; stamens 10, free, anthers ovate with a sessile, globose apical gland; pollen in tetrahedral tetrads with tricolporate grains, exine smooth (perforated), columellae present; large intra-staminal nectary disk surrounding the ovary; ovary sessile, stigma porate. **Fruits** very large, 60–80 × 15–20 cm, broadly oblong (Fig. 127L), flattened, dehiscent along both sutures, 8–10-seeded; valves papery to coriaceous, outer layer waxy, with raised reticulate venation, endocarp smooth, papery, brown with whitish fibrous layers between the seeds which are transverse in legume. **Seeds** large, flattened, membranous-winged, 10–15 × 2.5–4 cm, wing to 2.5–3 cm wide (Fig. 127M), testa membranous, funicle attached near the middle of the seed, pleurogram absent, endosperm lacking.

#### Chromosome number.

Unknown.

#### Included species and geographic distribution.

**Figure 130.**
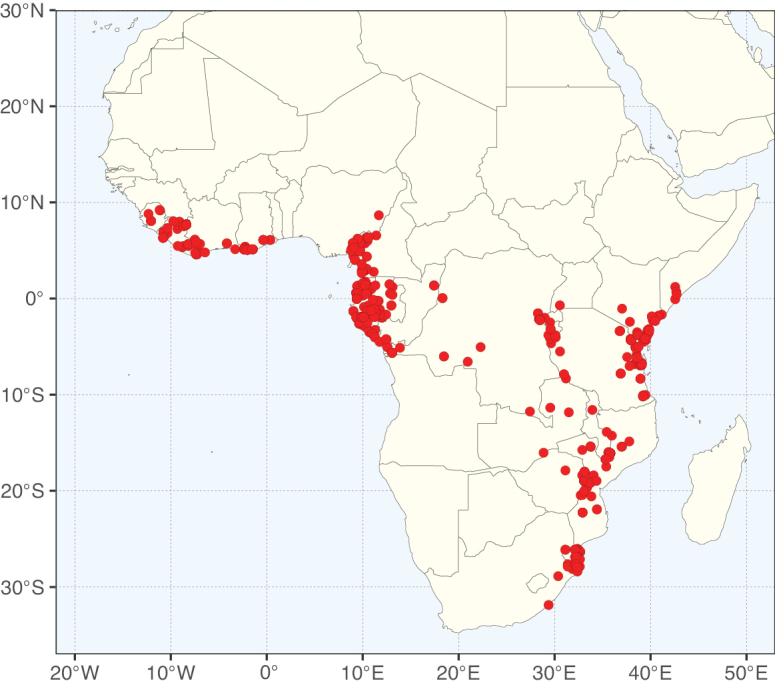
Distribution of *Fillaeopsis* based on quality-controlled digitised herbarium records. See Suppl. material 1 for the source of occurrence data.

Monospecific (*F.discophora*), restricted to central Africa in Cameroon, Central African Republic, Democratic Republic of Congo, Equatorial Guinea, Gabon, Nigeria, Republic of Congo (Fig. 130).

#### Ecology.

Large canopy-emergent trees growing in closed-canopy evergreen lowland tropical rainforests (Fig. 127C). Seeds wind-dispersed.

#### Etymology.

From Greek *Fillaea*- (a legume genus name, now a synonym of *Erythrophleum*), and -*opsis* (= appearance) and hence named for its resemblance to the genus *Fillaea*.

#### Human uses.

The wood is light and mainly used for veneer and plywood (Yvon 1975).

#### Notes.

*Fillaeopsis* forms its own monogeneric (and monospecific) lineage embedded in the grade that subtends the core mimosoid clade (Fig. 126). The fruits of *Fillaeopsis* and the winged seeds are amongst the largest known in Mimoseae, indeed across all Caesalpinioideae (Fig. 127L, M), underpinning wind dispersal of the seeds, which also characterises the genera *Plathymenia* and *Newtonia*.

The leaves of *Fillaeopsis* are very similar to those of *Cylicodiscus*, although flowers and fruits of the two genera are quite different. When sterile the two genera can be distinguished by the following characters: *Fillaeopsis* lacks a gland at the apex of the petiole, whereas *Cylicodiscus* has a small, sunken gland; leaflets of *Fillaeopsis* have a conspicuous marginal vein and are elliptical in shape, whereas leaflets of *Cylicodiscus* have an inconspicuous marginal vein and are ovate.

#### Taxonomic references.

Harms (1899); Villiers (1989), both with illustrations.

### ﻿ Newtonia

Taxon classificationPlantaeFabalesFabaceae

Baill., Bull. Mens. Soc. Linn. Paris 1(91): 721. 1888.

Figs 127 , 131

#### Type.

*Newtoniainsignis* Baill. [= *N.duparquetiana* (Baill.) Keay]

#### Description.

Unarmed large trees, often with buttresses (Fig. 127D) and aerial roots (one species a liana), tuberous storage roots absent; brachyblasts absent. **Stipules** small, caducous. **Leaves** bipinnate, extrafloral nectaries present between the proximal pair of pinnae and usually between all pairs, sessile or stipitate, crateriform to cylindrical; pinnae 1–27 pairs, opposite; leaflets 1–40 (67) pairs per pinna, opposite, sessile, pinnately veined, brochidodromous. **Inflorescences** paniculiform comprising aggregated spikes (Fig. 127H) (a raceme in one species), terminal or subterminal. **Flowers** sessile or pedicellate, functionally staminate or bisexual; hypanthium absent but the stamens and petals fused into a stemonozone, forming a nectary disk in several species; calyx 5 lobed, valvate; petals 5, valvate, basally connate or adnate to stamens; stamens 10, free above the stemonozone, anthers dorsifixed, anther glands present or absent; pollen in tricolporate monads, exine smooth (perforated), columellae present; ovary stipitate, stigma porate. **Fruits** strongly dorsiventrally flattened, dehiscing along ventral suture (Fig. 127N), valves coriaceous, exocarp brown, reticulately veined, endocarp lighter in colour, mesocarp absent. **Seeds** strongly flattened, winged (Fig. 127N), longitudinal, the testa thin, pleurogram absent, funicle attached apically.

#### Chromosome number.

2*n* = 26 (Goldblatt 1981b).

#### Included species and geographic distribution.

**Figure 131.**
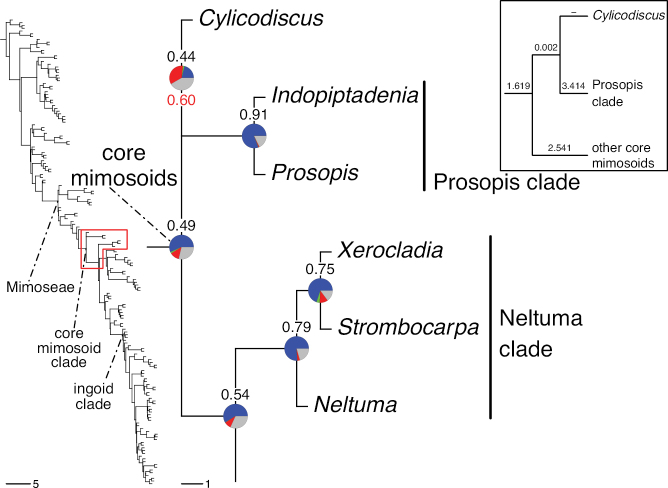
Distribution of *Newtonia* based on quality-controlled digitised herbarium records. See Suppl. material 1 for the source of occurrence data.

Eleven species, restricted to, but widely distributed in tropical Africa (Fig. 131).

#### Ecology.

*Newtonia* species are (with the exception of *N.scandens* Villiers) large trees, often with buttresses (Fig. 127D) and aerial roots, mostly in rainforests, although some species are found in more seasonal semi-deciduous forest in East Africa (Fig. 131). Seeds wind-dispersed (Fig. 127N).

#### Etymology.

Named by Baillon in honour of the English philosopher and mathematician Isaac Newton.

#### Human uses.

Various species are used for timber (Fern 2023).

#### Notes.

*Newtonia* is one of three genera of large, unarmed African trees with winged seeds that form part of the grade subtending the core mimosoid clade (Fig. 126). The follicular dehiscence of fruits of *Newtonia*, i.e., along one margin only, to release the winged, wind-dispersed seeds (Fig. 127N), is unusual in Mimoseae and found in only a few other genera (e.g., *Cylicodiscus*, *Piptadeniastrum* Brenan, *Pityrocarpa* (Benth.) Britton & Rose, and *Marlimorimia* L.P. Queiroz, L.M. Borges, Marc.F. Simon & P.G. Ribeiro). The spicate inflorescences are always aggregated into compound paniculiform terminal inflorescences (Fig. 127H).

In the past, several New World species were included in *Newtonia*. These are now placed in the genus *Marlimorimia*, a genus of the Stryphnodendron clade segregated from *Pseudopiptadenia* Rauschert by Borges et al. (2022), reflecting long-documented differences in pollen type [tricolporate monads in *Newtonia* vs polyads in *Pseudopiptadenia* (now *Marlimorimia*; Guinet 1969)] and other morphological differences (Lewis and Elias 1981; Lewis and Lima 1991).

#### Taxonomic references.

Brenan (1959); Hutchinson and Dalziel (1958); Mackinder and Cheek (2003); Villiers (1989, 1990), all with illustrations.

## ﻿﻿18. *Cylicodiscus*

Colin E. Hughes^3^, Melissa Luckow^29^, Gwilym P. Lewis^10^

Citation: Hughes CE, Luckow M, Lewis GP (2024) 18. Cylicodiscus. In: Bruneau A, Queiroz LP, Ringelberg JJ (Eds) Advances in Legume Systematics 14. Classification of Caesalpinioideae. Part 2: Higher-level classification. PhytoKeys 240: 250–253. https://doi.org/10.3897/phytokeys.240.101716

### ﻿ Cylicodiscus

Taxon classificationPlantaeFabalesFabaceae

Harms, Nat. Pflanzenfam. Nachtr. II–IV, 1: 192. 1897.

**Figure 132.**
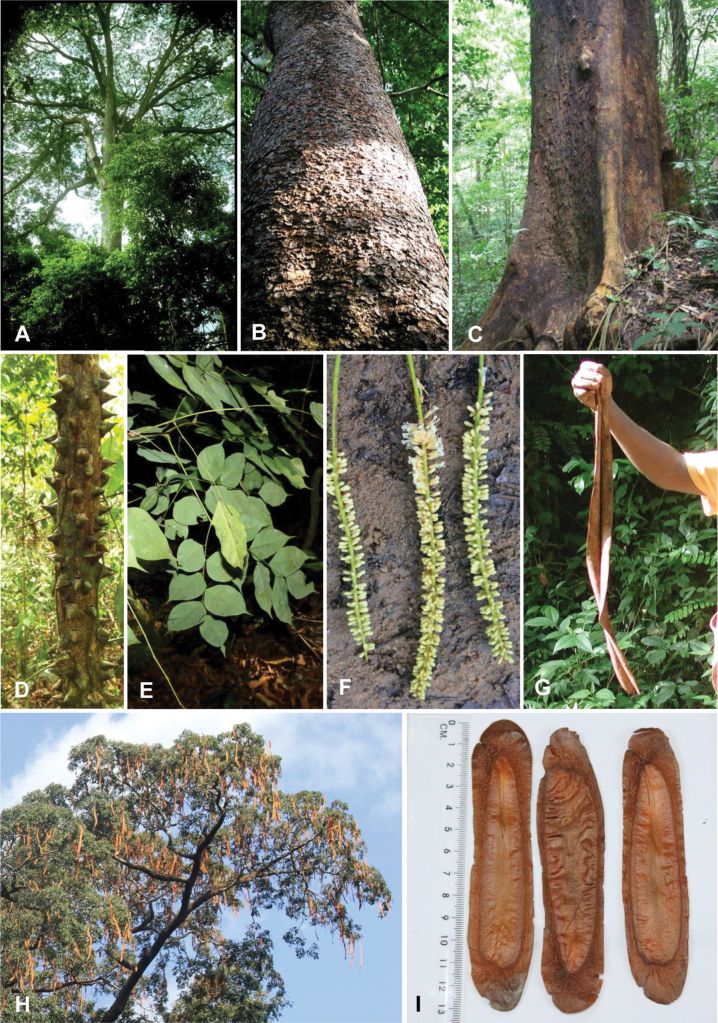
Phylogeny showing the relationships among *Cylicodiscus* and the genera of the Prosopis clade and the Neltuma clade, the first-branching lineages of the core mimosoid clade of tribe Mimoseae. Substantial gene tree conflict is associated with the relationships of *Cylicodiscus* which is separated from the Prosopis clade by a very short branch (see inset for exact branch lengths printed above branches). For description of phylogeny and support values, see Fig. 6 caption (page 63).

**Figure 133.**
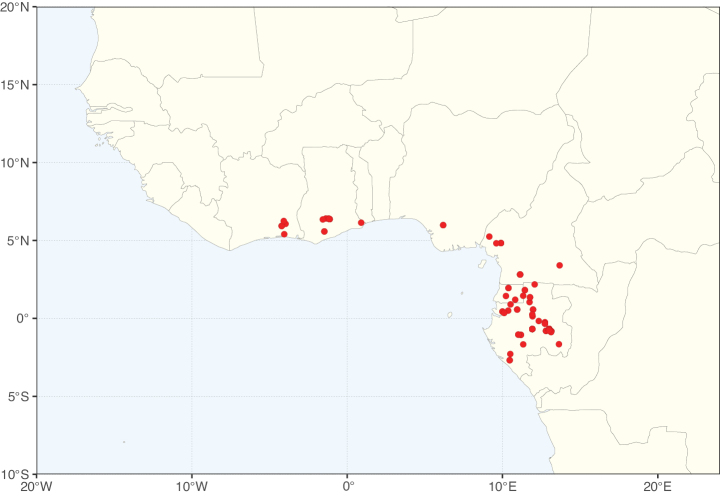
Habit, leaves, flowers and fruits of *Cylicodiscus* in wet tropical forest in west Africa **A, B***Cylicodiscusgabunensis* Harms, large canopy emergent tree **C** mature tree trunk with large knee-like buttresses **D** immature understory sapling showing sharp-pointed woody protuberances on stem **E** leaves showing leaflets with conspicuous drip tips **F** inflorescences **G** strap-like legumes (ca. 1 m long) showing follicular dehiscence **H** canopy-emergent tree crown with long strap-like pendulous fruits **I** 12 cm long winged seeds. Photo credits **A, B, D, E, G** W Hawthorne **C** X van der Burgt **F, H, I** R Ndonda Makemba.

**Figure 134.**
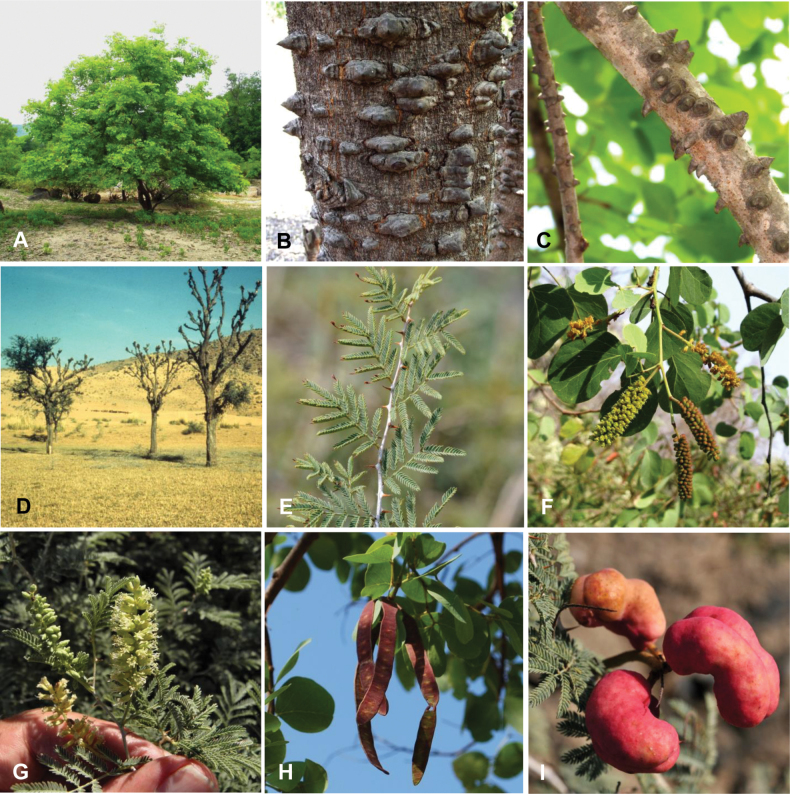
Distribution of *Cylicodiscus* based on quality-controlled digitised herbarium records. See Suppl. material 1 for the source of occurrence data.

Figs 132 , 133 , 134

Cyrtoxiphus Harms, Nat. Pflanzenfam. Nachtr. II–IV 1: 203. 1897. Type: Cyrtoxiphusstaudtii Harms [= Cylicodiscusgabunensis Harms]

#### Type.

*Cylicodiscusgabunensis* Harms

Although *Cylicodiscus* is clearly a member of the core mimosoid clade, its phylogenetic relationship to the Prosopis clade, from which it is separated by a very short branch, and to the Neltuma clade, are not well-resolved (Fig. 132). For this reason, *Cylicodiscus* is here presented as a separate monogeneric lineage rather than being included in another named clade of Mimoseae.

#### Description.

Very large trees 25–65 m, to 70+ cm trunk diameter, bole straight cylindrical, thick wandering buttresses with knee-like outgrowths (Fig. 133A–C) and small adventitious roots at base; trunks of young trees armed with sharply tipped pyramidal scattered woody protuberances (Fig. 133D); bark fibrous, rough, black-brown; slash brownish-yellow or reddish-orange, wood very hard, brachyblasts absent. **Stipules** caducous. **Leaves** bipinnate, petiole bearing a round, sunken nectary at the apex; pinnae 1–3 pairs, opposite; leaflets 3–7 pairs per pinna, alternate, venation brochidodromous. Inflorescences of solitary, axillary spiciform racemes; unfertilised flowers abscising post-anthesis to leave peg-like pedicels (Fig. 133E). **Flowers** white; hypanthium absent; calyx 5-lobed, valvate; petals 5, valvate, free; stamens 10, free, anthers dorsifixed, bearing an apical globose gland; intra-staminal disk present, well-developed; pollen in tricolporate monads, exine smooth (perforated), columellae present; ovary sessile/stipitate, stigma porate. **Fruits** pendulous, linear oblong, strap-like, up to 1 m long, dehiscent along the ventral suture (Fig. 133F), flattened, woody, ca. 10-seeded, exocarp cracking when mature, sutural ribs flattened, 5 mm wide; mesocarp of two layers, a black glassy layer underlying the endocarp and a reticulate, cardboard-like fibrous layer below that; endocarp smooth, fibrous. **Seeds** ellispoid, large, to 13 cm long and 2 cm wide (Fig. 133I) surrounded by a thin papery wing 6 mm wide, testa thin, papery, pleurogram absent, the funicle attached to the short end of the seed.

#### Chromosome number.

Unknown.

#### Included species and geographic distribution.

Monospecific (*C.gabunensis*), west and central Africa in Cameroon, Côte d’Ivoire, Equatorial Guinea, Gabon, Ghana, and Nigeria, widespread but apparently uncommon (or perhaps just difficult to collect!) (Fig. 134).

#### Ecology.

Very large canopy-emergent trees with massive crowns in well-drained evergreen lowland tropical Guineo-Congolan rainforest (Fig. 133A–C) and moist semi-deciduous forests. In common with several other genera of Mimoseae such as *Fillaeopsis* and some species of *Entada*, the flowers of *Cylicodiscus* are very small, yet give rise to very large fruits. Seeds winged and likely wind-dispersed (Fig. 133G–I).

#### Etymology.

From Greek *cylico* (= cup-shaped) and Latin *discus* (= disk), referring to the cup-shaped floral disk to which the stamens are attached.

#### Human uses.

The timber of *C.gabunensis* is very dense and used in heavy construction, for railway sleepers and flooring (Ndonda Makemba et al. 2019).

#### Notes.

**Figure 135.**
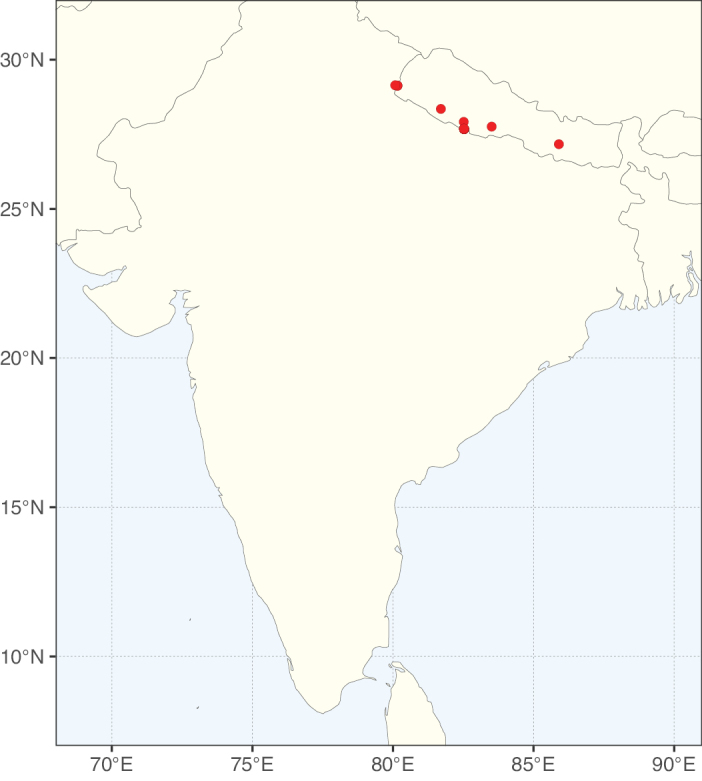
Habit, armature, inflorescences, and fruits of genera of the Prosopis clade **A** Small tree of *Indopiptadeniaoudhensis* (Brandis) Brenan in disturbed seasonal monsoon vegetation in Uttar Pradesh, northern India **B, C** scattered internodal sharply tipped woody protuberances on trunk and branches of young *Indopiptadeniaoudhensis* trees in Uttar Pradesh, northern India **D** trees of *Prosopiscineraria* (L.) Druce lopped for animal fodder on the arid fringes of the Thar desert, Rajasthan, India **E** internodal prickles on young shoot of *Prosopisfarcta* (Banks & Sol.) J.F. Macbride **F** inflorescences of *Indipiptadeniaoudhensis* in Uttar Pradesh, northern India **G** inflorescences of *Prosopisfarcta***H** unripe, plano-compressed fruits of *Indopiptadeniaoudhensis* in Uttar Pradesh, northern India **I** indehiscent fruits with a thickened mesocarp of *Prosopisfarcta*. Photo credits **A–C, F, H** O Bajpai and L Babu Chaudhary **D** CE Hughes **E** Zeynel Cebeci https://commons.wikimedia.org/wiki/File:Prosopis_farcta_-_Syrian_mesquite_01.JPG**G** Eitan Ferman https://de.wikipedia.org/wiki/Prosopis_farcta#/media/Datei:Prosopis_farcta,_flower.jpg**I** Zeynel Cebeci https://en.wikipedia.org/wiki/Prosopis_farcta#/media/File:Prosopis_farcta_01.JPG.

Phylogenetically, *Cylicodiscus* is separated by a very short branch from the Prosopis clade (Fig. 132). It is perhaps notable that both genera of the Prosopis clade, *Prosopis* L. and *Indopiptadenia* Brenan, share internodal prickles and/or sharp pyramidal scattered woody protuberances on the stem and shoots with *Cylicodiscus* (Figs 133D, 135B, C, E). Although the relationship of *Cylicodiscus* to the Prosopis and Neltuma clades remains uncertain, *Cylicodiscus* is clearly a member of the core mimosoid clade and shares presence of armature with the other two first-branching lineages of that clade.

#### Taxonomic references.

Aubréville (1959) with illustration; Burkill (1995); Ndonda Makemba et al. (2019); Villiers (1989) with illustrations.

## ﻿﻿19. Prosopis clade

Colin E. Hughes^3^, Melissa Luckow^29^, Gwilym P. Lewis^10^

Citation: Hughes CE, Luckow M, Lewis GP (2024) 19. Prosopis clade. In: Bruneau A, Queiroz LP, Ringelberg JJ (Eds) Advances in Legume Systematics 14. Classification of Caesalpinioideae. Part 2: Higher-level classification. PhytoKeys 240: 254–259. https://doi.org/10.3897/phytokeys.240.101716

**Prosopis clade**

Figs 132, 135–137

**Included genera (2).***Indopiptadenia* Brenan (1 species), *Prosopis* L. (3).

**Description.** Small trees, shrubs or subshrubs (Fig. 135A, D) or occasionally lianescent (*Prosopisfarcta* (Banks & Sol) J.F. Macbr.), (0.3) 3–12 m tall, armed with scattered internodal prickles on shoots (Fig. 135E) and in *Indopiptadenia* the trunk and branches with prominent scattered conical sharp-pointed protuberances (Fig. 135B, C), stipular spines or axillary thorns absent; brachyblasts absent. **Stipules** small, lanceolate, foliaceous, caducous. **Leaves** bipinnate, nectary between first pair of pinnae, often obscure, or at apex of petiole and often between leaflets, pinnae 1–6 (7) pairs, opposite or alternate; leaflets (1) 2–15 opposite pairs per pinna. **Inflorescences** spiciform racemes, solitary or in fascicles in leaf axils, or grouped into secondary terminal racemes (Fig. 135F, G). **Flowers** 5-merous, usually all hermaphrodite and appearing bisexual; hypanthium absent; sepals valvate in bud; petals valvate in bud, free or nearly so; stamens 10, free, anthers dorsifixed bearing minute caducous stipitate claviform apical glands arising from the connective; pollen in tricolporate monads; ovary sessile, stigma funnel-form. **Fruits** variable, either straight, plano-compressed, narrowly linear with coriaceous valves and dehiscent through both sutures (*Indopiptadenia*) (Fig. 135H), or indehiscent, cylindrical in cross-section, torluose with a thickened spongy mesocarp and thin endocarp segments (*Prosopis*) (Fig. 135I). **Seeds** compressed and unwinged (*Prosopis*) or flattened and winged (*Indopiptadenia*), pleurogram either absent or present.

**Distribution.** Restricted to the Old World in northern Africa, the Middle East, Pakistan, northern India and Nepal in dry and arid thorn scrub and seasonally dry deciduous or semi-evergreen monsoon forests.

**Clade-based definition.** The most inclusive crown clade containing *Indopiptadeniaoudhensis* (Brandis) Brenan and *Prosopiscineraria* (L.) Druce, but not *Cylicodiscusgabunensis* Harms, *Xerocladiaviridiramis* (Burch.) Taub. or *Newtoniahildebrandtii* (Vatke) Torre (Figs 126, 132).

**Notes.** The non-monophyly of the former *Prosopis* s.l. prompted its division into four segregate genera, *Anonychium* (Benth.) Schweinf., *Prosopis*, *Neltuma* Raf. and *Strombocarpa* Engelm. & A. Gray by Hughes et al. (2022b). This non-monophyly was first demonstrated by Catalano et al. (2008) and confirmed by phylogenomic analyses by Ringelberg et al. (2022) who showed that this is one of the most robustly supported parts of the Caesalpinioideae phylogeny, providing strong evidence for the non-monophyly of *Prosopis* s.l. and support for the division of *Prosopis* into four segregate genera (Hughes et al. 2022b). These four genera correspond to Burkart’s (1976) sections in his worldwide classification of *Prosopis* s.l. and coincide with variation in types of armature which distinguish between the genera and clades recognised here: *Anonychium*, along with *Plathymenia* Benth., *Fillaeopsis* Harms and *Newtonia* Baill., are unarmed; *Cylicodiscus* Harms, *Indopiptadenia* and *Prosopis* s.s. are armed with internodal prickles on the shoots and sometimes also on mature stems of young trees (Figs 133C, 135B, C, E); *Neltuma* has paired or solitary axillary nodal thorns or spinescent shoots (Fig. 138F, I); and *Xerocladia* Harv. and *Strombocarpa*, which are robustly supported as sister genera (Fig. 132), share spinescent stipules (Fig. 138G, H) (Hughes et al. 2022b). The reclassification of *Prosopis* s.l. by Hughes et al. (2022b) means that *Prosopis* is now reduced to three species, all of them native to the Old World.

The sister group relationship between *Prosopis* and the Indo-Nepalese monospecific genus *Indopiptadenia*, i.e., the Prosopis clade as circumscribed here, is robustly supported (Fig. 132). In addition to sharing internodal prickles on the shoots and/or prominent scattered conical sharp-pointed protuberances on branches and trunks, the two genera occupy a disjunct distribution across North Africa and west-central Asia that is unique within Caesalpinioideae.

### ﻿ Indopiptadenia

Taxon classificationPlantaeFabalesFabaceae

Brenan, Kew Bull. 10(2): 178. 1955.

Figs 135 , 136

#### Type.

*Indopiptadeniaoudhensis* (Brandis) Brenan [≡ *Piptadeniaoudhensis* Brandis]

#### Description.

Trees 3–12 m tall (Fig. 135A), variably unarmed or armed with scattered internodal prickles on shoots, and trunk with prominent scattered conical protuberances terminating in a sharp point (Fig. 135B, C); brachyblasts absent. **Stipules** small, lanceolate, caducous. **Leaves** bipinnate, extrafloral nectaries between first pair of pinnae or at apex of petiole and often between leaflets, shallow cupular or flat and pad-like, oval; pinnae 1–2 pairs, opposite; leaflets 1 (2) pairs per pinna, opposite, venation pinnate, brochidodromous. **Inflorescence** a spiciform raceme, not enveloped in fused bracts; solitary in leaf axils or grouped in secondary terminal racemes (Fig. 135F); pedicels flattened, pad-like, somewhat glandular, persistent on the receptacle after anthesis. **Flowers** greenish-yellow, lacking a hypanthium; sepals 5, valvate; petals 5, free, valvate; stamens 10, free, anthers dorsifixed, bearing a minute, deciduous claviform apical gland of the piptadenioid type (sensu Luckow and Grimes 1997); pollen in tricolporate monads, exine smooth (perforated), columellae present; ovary sessile, stigma funnel-shaped. **Fruits** straight, plano-compressed, narrowly linear (Fig. 135H), 15–20-seeded, valves coriaceous, dehiscent through both sutures. **Seeds** oblique, strongly flattened with a membranous wing, pleurogram absent, testa thin, funicle attached medially.

#### Chromosome number.

Unknown.

#### Included species and geographic distribution.

**Figure 136.**
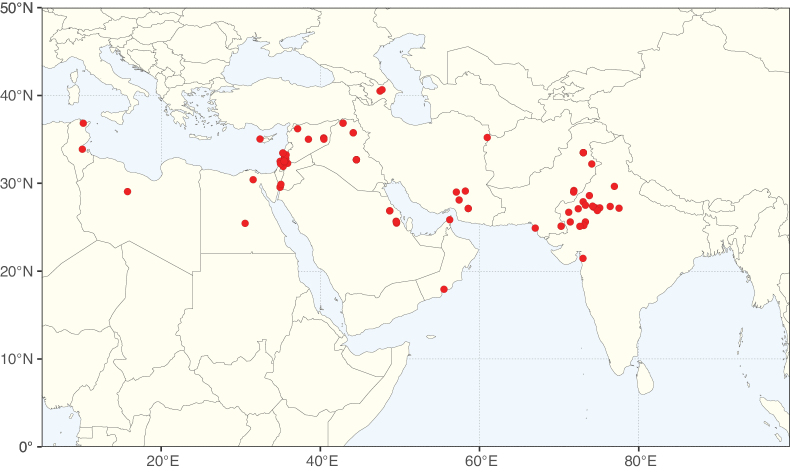
Distribution of *Indopiptadenia* based on quality-controlled digitised herbarium records. See Suppl. material 1 for the source of occurrence data.

Monospecific (*I.oudhensis*), occupying a narrow band restricted to the Terai region of the outlying lowest foothills of the Himalayas at scattered localities almost entirely within Nepal and just barely traversing the border into northern India in a few places (Fig. 136).

#### Ecology.

Seasonal deciduous or semi-evergreen monsoon forests and disturbed riverine vegetation (Fig. 135A) in the outlying foothills of the Himalayas, at 150–900 m elevation, mainly in gravelly and sandy soils. Deciduous. Seed dispersal passive, or possibly wind-dispersed.

#### Etymology.

*Indopiptadenia* refers to the superficial resemblance to the genus *Piptadenia* and Indo- (from India).

#### Human uses.

The leaves are used locally for fodder and the wood as fuel and timber. The wood is very hard, strong, and durable (Bajpai et al. 2014).

#### Notes.

*Indopiptadenia* remained poorly known, based on just a handful of collections, until Bajpai et al. (2014) published a detailed and amply illustrated account of the morphology and distribution of the genus. *Indopiptadenia* was segregated from *Piptadenia* by Brenan (1955) and later placed in the informal Newtonia group (Lewis and Elias 1981). *Indopiptadenia* is now known to be robustly supported as sister to the re-circumscribed *Prosopis* s.s. (Fig. 132; Hughes et al. 2022a; Ringelberg et al. 2022). Originally described as unarmed, *Indopiptadenia* often has small internodal prickles (shared with *Prosopis* s.s.) and characteristic conical sharply-tipped woody protuberances on the mature stems (Fig. 135B, C), strongly reminiscent of the stems of young trees of *Cylicodiscus* (Fig. 133C). The rupturing of the walls in immature fruits observed by Bajpai et al. (2014) is probably attributable to damage to the fruits, possibly by birds.

#### Taxonomic references.

Bajpai et al. (2014); Brenan (1955); Duthie (1906), with illustration.

### ﻿ Prosopis

Taxon classificationPlantaeFabalesFabaceae

L., Mantissa Pl. 68: 10. 1767.

Figs 135 , 137

Lagonychium M. Bieb., Fl. Taur.-Caucas. 3: 288. 1819. Type: Lagonychiumstephanianum M. Bieb. [= Prosopisfarcta (Banks & Sol.) J.F. Macbr.]
Prosopis sect. Adenopis DC., Prodr. [A.P. de Candolle] 2: 446. 1825. Type: Prosopisspicigera L. [= Prosopiscineraria (L.) Druce]
Pleuromenes Raf., Sylva Tellur.: 144. 1838. Type: Pleuromenesheterocarpa (Delile) Raf. [≡ Acaciaheterocarpa Delile (= Prosopisfarcta (Banks & Sol.) J.F. Macbr.)]

#### Type.

*Prosopisspicigera* L. [= *Prosopiscineraria* (L.) Druce]

#### Description.

Prickly subshrubs, shrubs, small trees (Fig. 135D) or occasionally lianescent (*P.farcta*), 0.3–6.5 (–10) m, deep-rooted and sometimes invading via root suckers, prickles internodal, scattered, straight, somewhat acroscopic, conical with broad bases (Fig. 135E), stipular spines or axillary thorns absent; brachyblasts absent. **Stipules** inconspicuous and caducous. **Leaves** bipinnate, an obscure gland between lower pair of pinnae; pinnae 1–6 (7) pairs, opposite/alternate; leaflets 7–15 pairs per pinna, opposite/alternate, mid-vein excentric. **Inflorescences** spiciform racemes, axillary, solitary or in fascicles (Fig. 135G), peduncle sometimes with an amplexicaul bract, this caducous and leaving an oblique scar. **Flowers** yellow, yellowish-white, green, or creamish-green; calyx truncate, sepals 5, valvate; petals 5, valvate, nearly free, reflexed; stamens 10, free, anthers with a minute caducous incurved claviform gland arising from the connective; pollen in tricolporate monads, pores without costae, exine irregularly areolate-verrucose, columellae present; ovary sessile or shortly stipitate, stigma porate. **Fruits** indehiscent, slender, cylindrical to sub-cylindrical in cross-section, torulose, mesocarp spongy, endocarp segments thin, little developed, seed chambers longitudinal or transverse (Fig. 135I). **Seeds** well separated, compressed, pleurogram present, not closed, testa hard.

#### Chromosome number.

2*n* = 28 (Goldblatt 1981b), polyploidy reported in *P.koelziana*: 2*n* = 28, 52 (Zaeifi et al. 2002).

#### Included species and geographic distribution.

**Figure 137.**
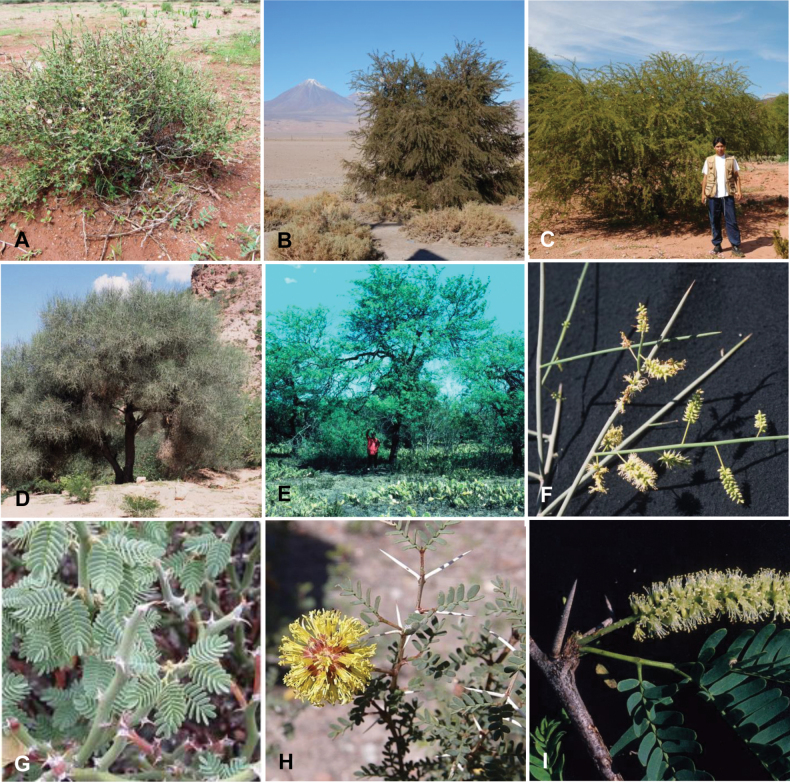
Distribution of *Prosopis* based on quality-controlled digitised herbarium records. See Suppl. material 1 for the source of occurrence data.

Three species distributed across arid parts of north Africa (but apparently the genus rare at its western limits in Libya and Tunisia), the Middle East, Pakistan and north-western India (especially Punjab and Rajasthan) and reaching its northern limits in Azerbaijan (Fig. 137).

#### Ecology.

Abundant in dry and arid parts of north-western India, where *P.cineraria* is sometimes the most common tree in parts of Punjab and Rajasthan (Fig. 135D) and abundant in arid thorn scrub in parts of the Middle East, where *P.farcta* (Banks & Sol.) J.F. Macbr. can spread via root suckers, and is sometimes considered weedy, tolerating saline soils. The fruits are eagerly consumed by herbivores, including livestock and this facilitates endozoochorous seed dispersal.

#### Etymology.

Pasiecznik et al. (2001) suggested the name to be derived from *pros*- (Greek = towards) and -*Opis* (wife of Saturn, the Greek goddess of abundance and agriculture), hence ‘towards agriculture’ referring to the widespread utility of the genus.

#### Human uses.

*Prosopiscineraria* is a highly valued tree, protected and cultivated in silvopastoral and other agroforestry systems as a source of high-quality durable wood, leaves for fodder, fruits for livestock feed and flowers as bee forage (Leakey and Last 1980) (Fig. 135D).

#### Notes.

Although *P.cineraria* and *P.farcta* are well-known, easily distinguished and widely distributed species, the third species of the genus, *P.koelziana* Burkart, is poorly-known from just a handful of collections from Iran. It has fruits similar to *P.farcta* and leaves similar to *P.cineraria*, and was suggested by Burkart (1976) to be of putative hybrid origin between these two species. In north-western India and Pakistan, introduced *Neltumajuliflora* (Sw.) Raf. is frequently found alongside native *P.cineraria* and the two are often confused having similar leaves and fruits, but can be distinguished based on armature.

#### Taxonomic references.

Burkart (1976); Hughes et al. (2022b).

## ﻿﻿20. Neltuma clade

Colin E. Hughes^3^, Melissa Luckow^29^, Gwilym P. Lewis^10^

Citation: Hughes CE, Luckow M, Lewis GP (2024) 20. Neltuma clade. In: Bruneau A, Queiroz LP, Ringelberg JJ (Eds) Advances in Legume Systematics 14. Classification of Caesalpinioideae. Part 2: Higher-level classification. PhytoKeys 240: 260–268. https://doi.org/10.3897/phytokeys.240.101716

**Neltuma clade**

Figs 132, 138–142

**Included genera (3).***Xerocladia* Harv. (1 species), *Strombocarpa* Engelm. & A. Gray (10), *Neltuma* Raf. (ca. 30).

**Figure 138.**
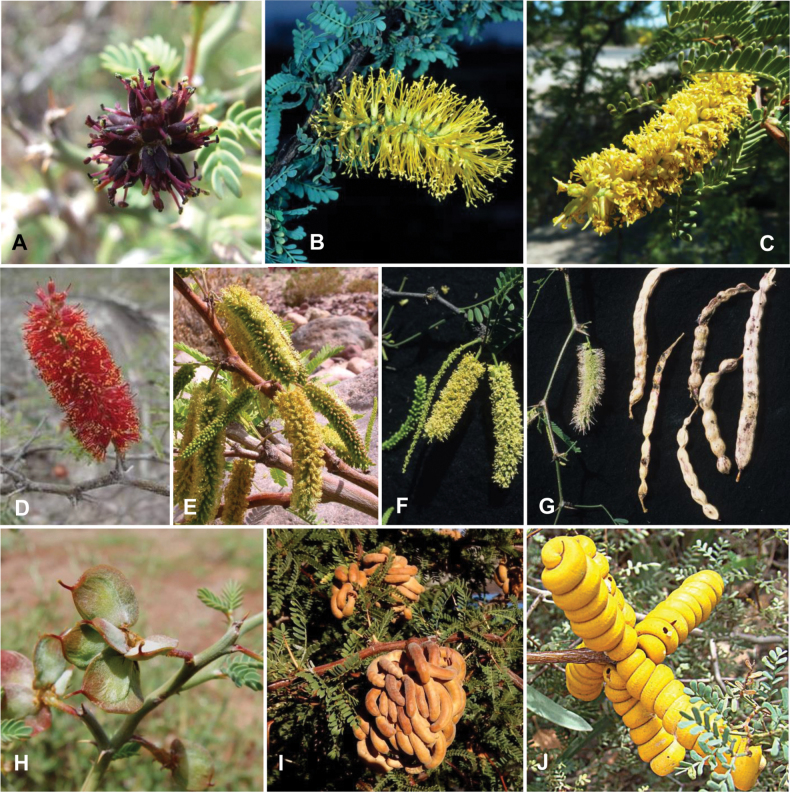
Habit and armature of the genera of the Neltuma clade **A** shrub of *Xerocladiaviridiramis* Taub. in the Namib desert, Namibia **B** small tree of *Strombocarpatamarugo* (Phil.) C.E Hughes & G.P. Lewis, on the Pampa del Tamarugal in the Atacama desert, northern Chile **C** shrubby treelet of *Strombocarpaferox* (Griseb.) C.E. Hughes & G.P. Lewis in seasonally dry tropical scrubland in southern Bolivia **D** small tree of *Neltumakuntzei* (Harms ex C.E.O. Kuntze) C.E. Hughes & G.P. Lewis in seasonally dry tropical forest in southern Bolivia **E** small tree of *Neltumajuliflora* (Sw.) Raf. in seasonally dry tropical scrubland on deep black vertisol soil in central Nicaragua **F** straight cylindrical spinescent shoots of the sub-aphyllous *Neltumakuntzei***G** short recurved stipular spines and unijugate leaves of *Xerocladiaviridiramis***H** slender pale whitish stipular spines, unijugate leaves, and globose to ovoid-elliptic capitula of *Strombocarpastrombulifera* (Lam.) A. Gray **I** axillary nodal spine of *Neltumajuliflora*, central Nicaragua. Photo credits **A** H Kolberg, Plants of Namibia https://herbaria.plants.ox.ac.uk/bol/Namibia**B** O Whaley **C–F, I** CE Hughes **G** GN Dreber https://www.southernafricanplants.net/plantdata_sub.php?Mspec_ID=6570**H** Guillermo Debandi, iNaturalist (https://guatemala.inaturalist.org/photos/1502506).

**Figure 139.**
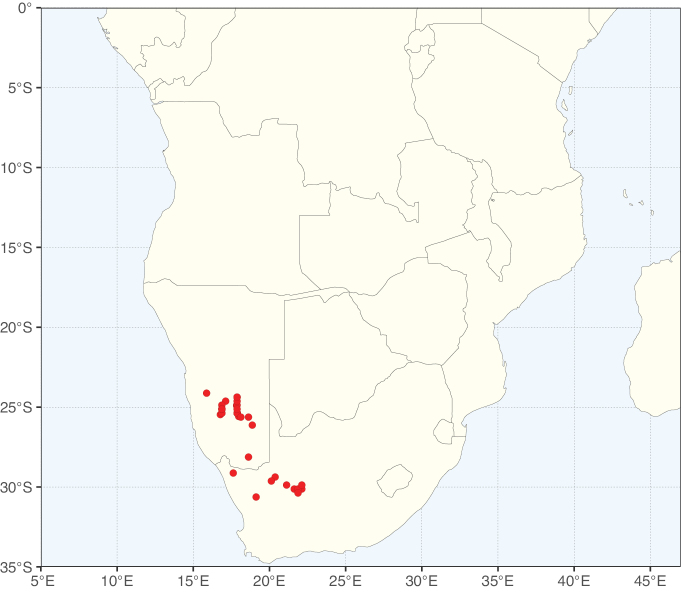
Inflorescences and fruits of the genera of the Neltuma clade **A** capitulum of *Xerocladiaviridiramis* Taub. **B** spike of *Strombocarpapalmeri* (S. Watson) C.E. Hughes & G.P. Lewis **C** spike of *Strombocarpatamarugo* (Phil.) C.E Hughes & G.P. Lewis **D** erect spike of *Neltumarubriflora* (Hassl.) C.E. Hughes & G.P. Lewis **E** pendulous spikes of *Neltumaflexuosa* (DC.) C.E. Hughes & G.P. Lewis, Antofagasta, Chile **F** spikes of *Neltumalaevigata* (Humb. & Bonpl. ex Willd.) Britton & Rose, south-central Mexico **G** indehiscent pods of *Neltumalaevigata*, south-central Mexico **H** unripe winged monospermous fruits of *Xerocladiaviridiramis* in the Namib desert, Namibia **I** ripe fruits of *Strombocarpatamarugo*, Atacama desert, northern Chile **J** spirally coiled indehiscent ‘screw-bean’ legumes of *Strombocarpastrombulifera* (Lam.) A. Gray. Photo credits **A, H** H Kolberg, Plants of Namibia https://herbaria.plants.ox.ac.uk/bol/namibia) **B, F, G** CE Hughes **C, I** O Whaley **D** X Enboc- Encontro de Botânicos do Centro Oeste **E** P Hechenleitner **J** Dick Culbert.

**Description.** Intricately much-branched erect to prostrate shrubs and small trees (Fig. 138A–E), (0.1) 1–15 (20)m tall, armed with either solitary or paired uninodal axillary thorns, spinescent shoots (*Neltuma*) (Fig. 138F, I), or spinescent stipules (*Xerocladia* and *Strombocarpa*) (Fig. 138G, H), internodal prickles absent; shoots often green; brachyblasts either absent or present (sometimes obscure), congested. **Stipules** spinescent, short, recurved or strongly decurrent and straight, or small, not spinescent and often obscure (*Neltuma*). **Leaves** bipinnate, petiole with a nectary immediately below or at insertion of pinnae, pinnae 1–3 (8) pairs, always unijugate in *Xerocladia* and *Strombocarpa* (Fig. 138G, H), opposite; leaflets (1) 2–30 (50) pairs per pinna, opposite, sub-opposite or alternate, or sometimes aphyllous or sub-aphyllous. **Inflorescence** either a globose or ovoid elliptic capitulum (*Xerocladia* and some *Strombocarpa*) or a spiciform raceme, solitary or in fascicles in leaf axils (Figs 138F, H, I, 139A–F). **Flowers** dark maroon, red, white, pale to bright lemon yellow or yellowish-green (Figs 138F, H, I, 139A–F), 5-merous, sometimes functionally staminate flowers basally, but usually all hermaphrodite and appearing bisexual; sepals 5, valvate in bud; petals 5, valvate in bud, free almost to the base or partially united; stamens 10, free, anthers dorsifixed bearing minute caducous, often incurved, stipitate claviform apical glands arising from the connective; pollen in tricolporate monads; ovary sessile or short-stipitate, stigma truncate or porate. **Fruits** variable, either pale orange-brown, 1 (2)-seeded, indehiscent, broadly falcate to semi-orbicular, compressed, the lower suture arched and winged, the valves coriaceous (Fig. 139H) (*Xerocladia*), or pale straw-yellow, streaked purple or black, many-seeded, indehiscent, moniliform or linear, straight, curved, annular or tightly coiled, with a thick, pulpy or mealy dry, and usually sweet mesocarp and a hard bony or coriaceous endocarp, segmented into closed seed chambers (*Strombocarpa* and *Neltuma*) (Fig. 139G, I, J). **Seeds** compressed sub-circular, ovate, reniform-ovoid, to elliptic, pleurogram present, U-shaped.

**Distribution.** Amphi-Atlantic, widespread in seasonally dry tropical and arid regions of the Americas, there with a somewhat bicentric predominantly amphitropical distribution of species diversity in the Mexican-Texan and the Argentinian-Chilean-Paraguayan regions, and in ecologically similar arid regions of Namibia and South Africa.

**Clade-based definition.** The most inclusive crown clade containing *Xerocladiaviridiramis* (Burch.) Taub. and *Neltumalaevigata* (Humb. & Bonpl. ex Willd.) Britton & Rose, but not *Cylicodiscusgabunensis* Harms, *Neptuniaoleracea* Lour. or *Newtoniahildebrandtii* (Vatke) Torre (Figs 126, 132).

**Notes.** The Neltuma clade and the relationships among its three genera are robustly supported (Ringelberg et al. 2022; Fig. 132). Within the clade, *Xerocladia* and *Strombocarpa* form a robust subclade supported by shared presence of stipular spines (Fig. 138G, H), which are never found in *Neltuma*, and by uniformly unijuguate leaves, which also occur in a few species of *Neltuma*. When Hughes et al. (2022a) split the former *Prosopis* s.l., resurrecting the genera *Anonychium* (Benth.) Schweinf., *Strombocarpa* and *Neltuma*, they justified recognition of two separate New World genera (i.e., *Strombocarpa* and *Neltuma*) to maintain the morphologically distinctive African *Xerocladia* (which is sister to *Strombocarpa*), with its unusual small, round, winged fruits which are unique within Mimoseae. Although the two New World genera have strongly overlapping distributions (see below), they can be immediately distinguished based on armature: *Strombocarpa* with spinescent stipules (Fig. 138G, H) and *Neltuma* with nodal solitary or paired axillary thorns or spinescent shoots (Fig. 138F, I). Furthermore, *Strombocarpa* and *Neltuma* correspond to Burkart’s (1976) sections *Strombocarpa* and *Algarobia* + *Monilicarpa* respectively and are placed in deeply divergent lineages; within each genus there is significant interspecific crossability and hybridisation but no hybrids between the two genera are known, and each genus has largely separate cohorts of bruchid seed predators, a set of differences that further justified recognition of *Strombocarpa* and *Neltuma* as separate genera (Hughes et al. 2022b).

Species of the Neltuma clade are almost entirely confined to arid and semi-arid succulent-rich, fire-free scrubland vegetation spanning three highly disjunct centres of species diversity in North America, South America, plus the outlying monospecific *Xerocladia* in southern Africa, providing another striking example of phylogenetic conservatism across the trans-continental succulent biome (Ringelberg et al. 2020).

### ﻿ Xerocladia

Taxon classificationPlantaeFabalesFabaceae

Harv. in W.H. Harvey & Sonder, Fl. Cap. 2: 278. 1862.

**Figure 140.**
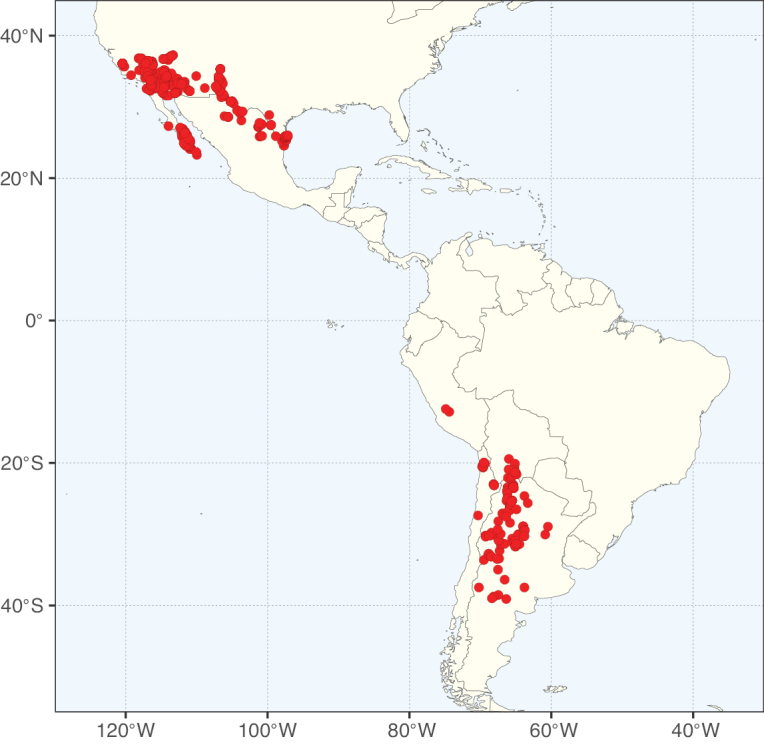
Distribution of *Xerocladia* based on quality-controlled digitised herbarium records. See Suppl. material 1 for the source of occurrence data.

Figs 138 , 139 , 140

#### Type.

*Xerocladiazeyheri* Harv. [= *Xerocladiaviridiramis* (Burch.) Taub.]

#### Description.

Small, rigid, densely and intricately much-branched shrub to 1 m (Fig. 138A), the branches often somewhat zig-zag, shoots mid- to olive-green, sub-striate; brachyblasts absent. **Stipules** spinescent, in pairs, recurved (Fig. 138G). **Leaves** small, bipinnate; petiole with a stipitate reddish-brown gland immediately below insertion of pinnae, unijugate (Fig. 138G); leaflets 6–12 pairs per pina, sub-opposite, usually with a small reddish-brown gland at the base of each leaflet. **Inflorescences** solitary capitula, in axils of leaves (Fig. 139A). **Flowers** sessile, dark maroon, young filaments reddish-maroon (Fig. 139A); sepals 5, valvate, free almost to the base; petals 5, valvate, free except at base; stamens 10, free, anthers with a minute, caducous, apical claviform gland arising from the connective; pollen in tricolporate monads, pores with costae, exine smooth, perforated, columellae present; ovary shortly stipitate, stigma truncate. **Fruits** sessile, indehiscent, broadly falcate-ovate to semi-orbicular, compressed, the lower suture arched and winged, 1 (2)-seeded, valves coriaceous, chestnut- to reddish- or purplish-brown when ripe (Fig. 139H). **Seeds** sub-circular to elliptical, smooth, pleurogram present, U-shaped, testa hard.

#### Chromosome number.

Unknown.

#### Included species and geographic distribution.

Monospecific (*X.viridiramis*), endemic to arid parts of Namibia and Namaqualand in southern Africa (Fig. 140).

#### Ecology.

Arid scrubland in sandy river soils, on riverbanks, alluvium and saline flats.

#### Etymology.

From Greek, *xero*- (= dry) and -*cladion* (= branch), in reference to the dense branching habit typical of small shrubs in arid climates.

#### Human uses.

Unknown.

#### Notes.

*Xerocladia* has long been thought to be closely related to the former *Prosopis* s.l. (Catalano et al. 2008) with which it shares arid ecology and characteristic green photosynthetic shoots. It is robustly supported as sister to *Strombocarpa* with which it shares similar armature in the form of spinescent stipules, and unijugate leaves. However, despite these obvious similarities, *Xerocladia* is a highly distinctive and easily recognised genus, and its small reniform, flattened, indehiscent, 1 (2)-seeded winged fruits are unique within Mimoseae (Fig. 139H).

Material referred to under the name *Xerocladiapampeana* Speg. from Argentina, shows clear affinities to the genus *Prosopidastrum* Burkart, as suggested by Palacios and Hoc (2005).

#### Taxonomic references.

Dyer (1975); Ross (1975a); illustration Fl. Southern Africa 16: 131.

### ﻿ Strombocarpa

Taxon classificationPlantaeFabalesFabaceae

Engelm. & A. Gray, Boston J. Nat. Hist. 5: 243. 1845.

**Figure 141.**
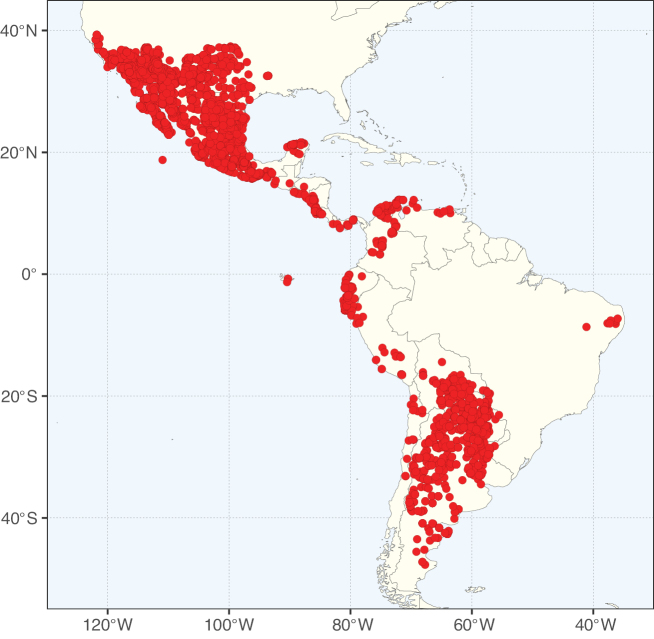
Distribution of *Strombocarpa* based on quality-controlled digitised herbarium records. See Suppl. material 1 for the source of occurrence data.

Figs 138 , 139 , 141

Spirolobium A.D. Orb., Voy. Amér. Mér. 8 (Atlas, Bot): t. 13. 1839, nom. rej. vs. Spirolobium Baill., Bull. Mens. Soc. Linn. Paris 1: 773. 1889 (Apocynaceae). Type: Spirolobiumaustrale A.D. Orb. [= Strombocarpastrombulifera (Lam.) A. Gray]
Sopropis Britton & Rose, N. Amer. Fl. 23: 182. 1928. Type: Sopropispalmeri (S. Watson) Britton & Rose [≡ Prosopispalmeri S. Watson (≡ Strombocarpapalmeri (S. Watson) C.E. Hughes & G.P. Lewis)]

#### Type.

*Strombocarpastrombulifera* (Lam.) A. Gray [≡ *Mimosastrombulifera* Lam.]

#### Description.

Low spiny, sometimes creeping, shrubs or small trees (Fig. 138B, C), 0.15–3 (18) m tall, multi-stemmed from base or sometimes with a short trunk, 10–30 (45) cm in diameter, usually densely and intricately much-branched, some species forming long underground, spreading, horizontal runners (gemmiferous roots or rhizomes), armed with strongly decurrent, straight, cinereous, white or pale-grey, stout, glabrous, 0.5–2 cm long spiny stipules (Fig. 138H); brachyblasts congested, blackish, prominent or obscure, or sometimes absent. **Stipules** spinescent. **Leaves** bipinnate, always unijugate; obscure gland between pinnae; leaflets 3–30 pairs per pinna, well separated, alternate to opposite, veins lacking or weakly 1–3-veined. **Inflorescences** axillary, solitary, globose to ovoid-elliptic capitula (Fig. 138H) or shortly cylindrical spikes (Fig. 139B, C). **Flowers** bright lemon yellow (Figs 138H, 139B, C), young filaments sometimes reddish; sepals 5, valvate; petals 5, valvate, partially united; stamens 10, free, anthers with a minute, caducous, incurved claviform gland on the connective; pollen in tricolporate monads, pores with costae, exine smooth, perforated, columellae present; ovary sessile or shortly stipitate, stigma porate. **Fruits** indehiscent, lemon-yellow, straw-yellow or reddish-brown when ripe, slender, elongate, sometimes almost straight or falcate, but usually more or less tightly spirally coiled (Fig. 139I, J) with (1) 8–19 (24) regular coils; exocarp crustaceous, mesocarp thin or more usually thick and pulpy, tannic, reddish, endocarp delicately segmented in longitudinal or transverse seed chambers which are easy to open or hard and closed. **Seeds** ovate or reniform ovoid, testa hard, pleurogram present, not closed.

#### Chromosome number.

2*n* = 28 (Bukhari 1997).

#### Included species and geographic distribution.

Ten species. Restricted to the New World and there occupying a markedly bicentric amphitropical distribution in arid and semi-arid regions of North America [southern USA, especially in the Sonoran Desert, Baja California, and northern Mexico (Coahuila)] and South America (south-central Peru to Argentina, Bolivia, and Chile) (Fig. 141).

#### Ecology.

Often abundant in cactus-rich, semi-desert Monte vegetation, deserts and arid mesetas, dry river-beds and washes (Fig. 138B, C) and in the hyper-arid Pampa del Tamarugal in northern Chile [*S.tamarugo* (Phil.) C.E. Hughes & G.P. Lewis] (Fig. 138B), where it is the only tree present and dependent on moisture absorbed from fog. The indehiscent fruits are consumed by herbivores facilitating endozoochorous seed dispersal.

#### Etymology.

*Strombo*- (Italian = conch) and -*carpa* (Greek = fruit), in reference to the resemblance of the fruits to the spiral shells of some tropical marine molluscs (Fig. 139J).

#### Human uses.

Fruits browsed by cattle and sheep and much valued in arid deserts for that purpose (Bell and Castetter 1937). Wood valued for fuel, and occasionally cultivated (*S.tamarugo*) (Pasiecznik et al. 2001).

#### Notes.

*Strombocarpa* is one of three genera segregated from *Prosopis* s.l. (Hughes et al. 2022a) and corresponds to Burkart’s section Strombocarpa of *Prosopis* s.l., characterised by armature in the form of spinescent stipules (Fig. 138H) which it shares with its sister genus *Xerocladia* (Fig. 132; Ringelberg et al. 2022), and which are not found in either *Prosopis* s.s. nor the genus *Neltuma*. A subset of species, the so-called ‘Screw-Beans’ (in the USA), like the ecologically important velvet mesquite, *S.pubescens* (Benth.) A. Gray in North America, have highly distinctive spirally coiled fruits. Across the genus as a whole fruits range from weakly falcate, to strongly curved, annular and tightly coiled (see Hughes et al. 2022b, Fig. 5).

#### Taxonomic references.

Benson (1941); Burkart (1976); Hughes et al. (2022b); Palacios (2006).

### ﻿ Neltuma

Taxon classificationPlantaeFabalesFabaceae

Raf., Sylva Tellur.: 119. 1838.

**Figure 142.**
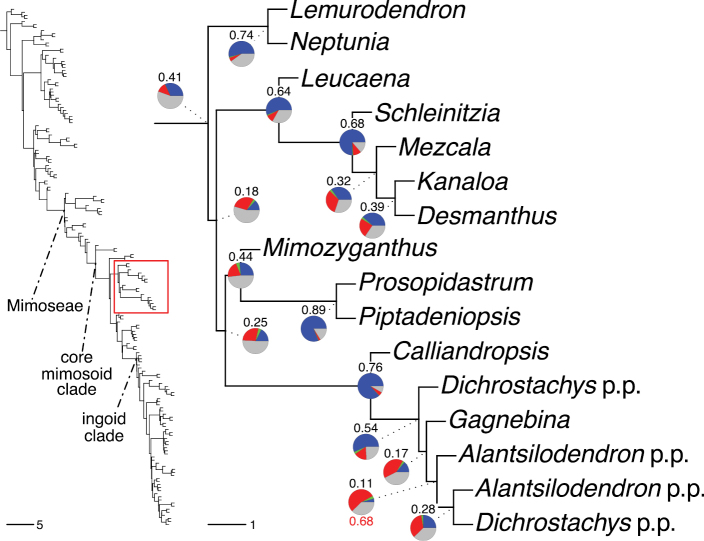
Distribution of *Neltuma* based on quality-controlled digitised herbarium records. See Suppl. material 1 for the source of occurrence data.

Figs 138 , 139 , 142

Prosopis sect. Algarobia DC., Prodr. [A.P. de Candolle] 2: 446. 1825. Type: Prosopisalgarobilla Griseb. [= Neltumaaffinis (Spreng.) C.E. Hughes & G.P. Lewis]
Mitostax Raf., Sylva Tellur.: 120. 1838. Type: Mitostaxpallida (Humb. & Bonpl. ex Willd.) Raf. [≡ Acaciapallida Humb. & Bonpl. ex Willd. (≡ Neltumapallida (Humb. & Bonpl. ex Willd.) C.E. Hughes & G.P. Lewis)]
Algarobia (DC.) Benth., Pl. Hartw.: 13. 1839. Type: Algarobiadulcis (Kunth) Benth. [= Neltumalaevigata (Humb. & Bonpl. ex Willd.) Britton & Rose]
Prosopis sect. Monilicarpa Ruiz Leal ex Burkart, J. Arnold Arbor. 57(3): 230. 1976. Type: Prosopisargentina Burkart [≡ Neltumaargentina (Burkart) C.E. Hughes & G.P. Lewis]

#### Type.

*Neltumajuliflora* (Sw.) Raf. [≡ *Mimosajuliflora* Sw.]

#### Description.

Spiny, erect to prostrate subshrubs, shrubs and small trees, (0.1) 4–10 (20) m tall, usually with a short trunk to 40–60 (>100) cm diameter (Fig. 138D, E), branching lax, crown spreading, rounded or flat-topped, twigs flexuous, often arched downwards, glabrous, green or reddish, armed with uninodal axillary, solitary or paired, straight, strong, cylindrical, subulate thorns (Fig. 138I), these not necessarily at all nodes, sometimes thicker than subtending twig, or with spinescent rigid straight cylindrical branchlets (Fig. 138F); brachyblasts present (sometimes obscure), congested, blackish, or absent. **Stipules** small, often obscure. **Leaves** bipinnate, an obscure gland between first pair of pinnae; pinnae 1–3 (8) pairs; leaflets (1) 2–30 (50) pairs, opposite, palmately pinnativeined or almost without veins, or sometimes aphyllous or subaphyllous. **Inflorescences** axillary, solitary or fascicled, spiciform racemes (Figs 138F, I, 139D–G). **Flowers** white, yellow, greenish-yellow or occasionally red (Figs 138F, I, 139D–F), often perfumed, sometimes functionally staminate flowers proximally; calyx short, valvate; petals 5, valvate, almost free; stamens 10, free, anthers with a minute caducous incurved claviform gland on the connective; pollen in tricolporate monads, exine smooth, perforated, columellae present; ovary sessile or stipitate, stigma porate. **Fruits** linear moniliform or compressed turgid (Fig. 139G), straw-yellow, sometimes tinged reddish-maroon or black, indehiscent, glabrous, mostly straight to sub-falcate, S- or C-shaped or annular with 1–3 very lax open spirals but never tightly coiled, margins often thickened and undulate, valves striate, corrugate or smooth, exocarp crustaceous, mesocarp thin or more usually thick and pulpy, mealy or spongy, dry, usually sweet, endocarp hard and bony or coriaceous, segmented in longitudinal or transverse subquadrate closed seed chambers. **Seeds** brown, compressed ovate, pleurogram present, not closed, testa hard.

#### Chromosome number.

2*n* = 26 or 28 (Goldblatt 1981b; Bukhari 1997); polyploidy reported in *N.juliflora*, 2*n* = 52 (Hunziker et al. 1975; Bukhari 1997).

#### Included species and geographic distribution.

Probably around 30 species. Widespread across seasonally dry tropical and arid regions of the Americas with a pseudo-amphitropical bicentric pattern of greatest species diversity in the Mexican-Texan and Argentinian-Chilean-Paraguayan regions, especially diverse and abundant in the Chaco, with an outlying disjunct occurrence of *Neltumaruscifolia* (Griseb.) C.E. Hughes & G.P. Lewis of questionable nativity in the Caatinga in north-east Brazil, also extending into warm and some colder temperate areas in Texas and Nevada in the north and Patagonia in the south, where *N.denudans* Benth. reaches 48°S (Fig. 142).

#### Ecology.

Dominant across large tracts of the Gran Chaco in mixed sub-xerophyllous woodland, also in Monte vegetation, open desert forests in dry quebradas (Fig. 138D) along seasonal rivers, in *Stipa*-dominated pampas and semi-desert shrub steppe with hot summers and cold winters in Patagonia as far as 48°S, some species capable of surviving extreme drought; spanning a wide range of substrates and edaphic conditions including stony and sandy mesas, coastal and inland sand dunes and deep black seasonally inundated, sometimes saline, clay vertisols (Fig. 138E) (Simpson 1977). The indehiscent cylindrical fruits with a somewhat sugary, thickened, fibrous mesocarp, and an endocarp that is segmented and either thin or, more frequently, somewhat hardened into one-seeded coriaceous or bony seed chambers, and seeds with a hard seed coat and a pleurogram, are eagerly consumed by herbivores, including all kinds of livestock, facilitating effective endozoochorous seed dispersal. This has promoted dispersal and establishment of a subset of species as troublesome invasive weeds, both within their native ranges and where introduced. *Neltumajuliflora* (Sw.) Raf. is one of the most pernicious plant invaders on the planet (Shackleton et al. 2014; Ayanu et al. 2015; Kleinjan et al. 2021), with many diverse impacts including enhanced malarial transmission due to habitat changes (Muller et al. 2017).

#### Etymology.

Possibly derived from the common name *Mulla Thumma* in the Dravidian language Teluga in the Indian states of Andhra Pradesh and Telangana, where *N.juliflora* is introduced.

#### Human uses.

The wood generally hard, dense, durable and flexible, and widely used for fence posts, parquet flooring, barrels, firewood and charcoal and the dependable provision of large quantities of protein- and sugar-rich, non-toxic, highly palatable and nutritious fruits (Pasiecznik et al. 2001).

#### Notes.

*Neltuma* is one of three genera segregated from *Prosopis* s.l. (Hughes et al. 2022b) and corresponds to Burkart’s sections *Monilicarpa* + *Algarobia* of *Prosopis* s.l., characterised by armature in the form of solitary or paired axillary thorns (Fig. 138I). Hybridisation is apparently common among the subset of species of *Neltuma* forming the so-called Mezquite subclade (Hunziker et al. 1986; Castillo et al. 2021).

Thirteen species of ‘*Prosopis*’ have been described since the publication of Burkart’s (1976) monograph, all of which can be confidently placed in *Neltuma* although some of these new species may be no more than regional variants of the widespread and taxonomically difficult *N.pallida*/*N.juliflora* species complex.

#### Taxonomic references.

Burkart (1976); Hughes et al. (2022b); Johnston (1962); Palacios (2006).

## ﻿﻿21. Dichrostachys clade

Colin E. Hughes^3^, Melissa Luckow^29^

Citation: Hughes CE, Luckow M (2024) 19. Dichrostachys clade. In: Bruneau A, Queiroz LP, Ringelberg JJ (Eds) Advances in Legume Systematics 14. Classification of Caesalpinioideae. Part 2: Higher-level classification. PhytoKeys 240: 269–298. https://doi.org/10.3897/phytokeys.240.101716

**Dichrostachys clade**

Figs 143–160

**Included genera (14).***Alantsilodendron* Villiers (11 species), *Calliandropsis* H.M. Hern. & Guinet (1), *Desmanthus* Willd. (23), *Dichrostachys* (DC.) Wight & Arn. (13–14), *Gagnebina* Neck. ex DC. (7), *Kanaloa* Lorence & K.R. Wood (1), *Lemurodendron* Villiers & Guinet (1), *Leucaena* Benth. (24), *Mezcala* C.E. Hughes & J.L. Contr. (1), *Mimozyganthus* Burkart (1), *Neptunia* Lour. (ca. 22), *Piptadeniopsis* Burkart (1), *Prosopidastrum* Burkart (ca. 6), *Schleinitzia* Warb. ex J.C. Willis (4).

**Description.** Predominantly small trees and shrubs 1–15 m, occasionally larger trees to 30 m and 1 m stem diameter (*Lemurodendron*), or functionally herbaceous subshrubs from a woody taproot (some *Desmanthus* and *Neptunia*) and, in two species of *Neptunia*, floating aquatic herbs; mostly unarmed, or in *Mimozyganthus*, *Piptadeniopsis* and *Prosopidastrum*, armed with spinescent stipules, and occasionally spinescent shoots (*Dichrostachys*). **Stipules** small and caducous, or more often persistent and foliaceous or setiform, spinose in three genera. **Leaves** bipinnate, a nectary almost always present on the petiole or at point of insertion of first, and sometimes multiple pinnae pairs, these variably sessile or stipitate, ellipsoid, round, and crateriform or raised; pinnae 1–40 (60) pairs, leaflets (1) 2–60 pairs per pinna. **Inflorescences** capitate or spicate, solitary or in fascicles in axils of coevally developing leaves or forming compound panicles exserted beyond the foliage. **Flowers** variably homomorphic to strongly heteromorphic, usually so to some degree, with variable proportions of sterile, functionally staminate and hermaphrodite flowers in most genera, or subsets of species within genera, sterile flowers often with showy white, pink or yellow filiform or petaloid staminodia, or staminodia absent; sepals always valvate in bud except in *Mimozyganthus*, where imbricate; petals valvate; spherical claviform anther glands present or absent, or sometimes the connective of the anther with a minute sub-cylindrical or hooded apiculum; pollen unit highly variable including tricolporate monads, tetrahedral tetrads and calymmate or acalymmate polyads; ovary sessile or stipitate, glabrous or pubescent; stigma porate to funnelform, broadly peltate in two genera. **Fruits** generally linear or linear-oblong and straight, occasionally coiled, in two genera obovate or suborbicular, usually plano-compressed, the sutures more or less raised and in one genus (*Gagnebina*) the fruits usually winged, generally inertly dehiscent along one, or more usually both sutures, or in four genera elastically dehiscent from the apex, the valves recurving, or indehiscent and lomentiform (*Prosopidastrum* and *Piptadeniopsis*), or in one genus (*Schleinitzia*) margins separating but the valves remaining attached. **Seeds** generally unwinged, but winged in *Lemurodendron* and narrowly so in *Mimozyganthus*, pleurogram generally present, but absent in *Lemurodendron*, *Mimozyganthus* and *Piptadeniopsis*.

**Distribution.** Pantropical including Africa, North and South America, Asia, Australia and the Pacific, with the core species and generic diversity concentrated in seasonally dry tropical vegetation in Madagascar, Mexico and the Chaco region of South America. Outside this core amphi-Atlantic dry tropical range are a number of notable outliers: in the Pacific with *Kanaloa* (endemic to Hawaii) and *Schleinitzia* (western Pacific), in wet forests of north-eastern Madagascar with the narrowly endemic monospecific *Lemurodendron*, pantropically in aquatic and seasonally flooded habitats with *Neptunia*, the only known truly aquatic member of subfamily Caesalpinioideae, and extending weakly into temperate North America (*Desmanthus*).

**Figure 143.**
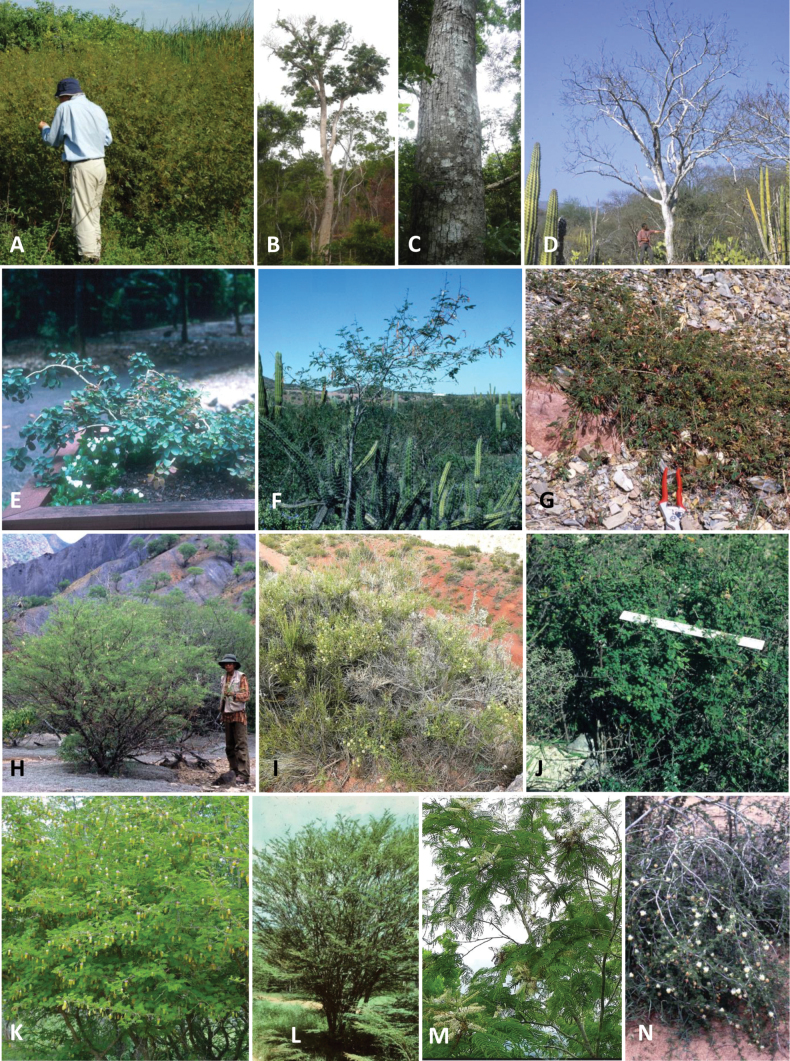
Generic relationships in the Dichrostachys clade (tribe Mimoseae). Note that the non-monophyly of *Dichrostachys* and *Alantsilodendron* has been simplified in this figure (see Suppl. material 2). For description of phylogeny and support values, see Fig. 6 caption (page 63).

**Notes.** The Dichrostachys clade sensu Koenen et al. (2020a) comprises 14 genera and ca. 116 species of generally deciduous small trees and shrubs (Fig. 144). It includes the informal Dichrostachys and Leucaena groups, originally delimited by Lewis and Elias (1981) and refined by Hughes et al. (2003) and Luckow (2005), both of which form robustly supported subclades (Fig. 143; Ringelberg et al. 2022), a largely South American subclade comprising *Mimozyganthus*, *Piptadeniopsis* and *Prosopidastrum* (Luckow et al. 2005), and a fourth subclade comprising *Neptunia* and *Lemurodendron*, which are together sister to the clade combining the rest of the genera of the Dichrostachys clade (Fig. 143; Koenen et al. 2020a; Ringelberg et al. 2022).

**Clade-based definition.** The most inclusive crown clade containing *Lemurodendroncapuronii* Villiers & Guinet and *Dichrostachysunijuga* Baker, but not *Prosopisspicigera* L., *Vachelliatortilis* (Forssk.) Galasso & Banfi or *Lachesiodendronviridiflorum* (Kunth) P.G. Ribeiro, L.P. Queiroz & Luckow (Fig. 143).

**Figure 144.**
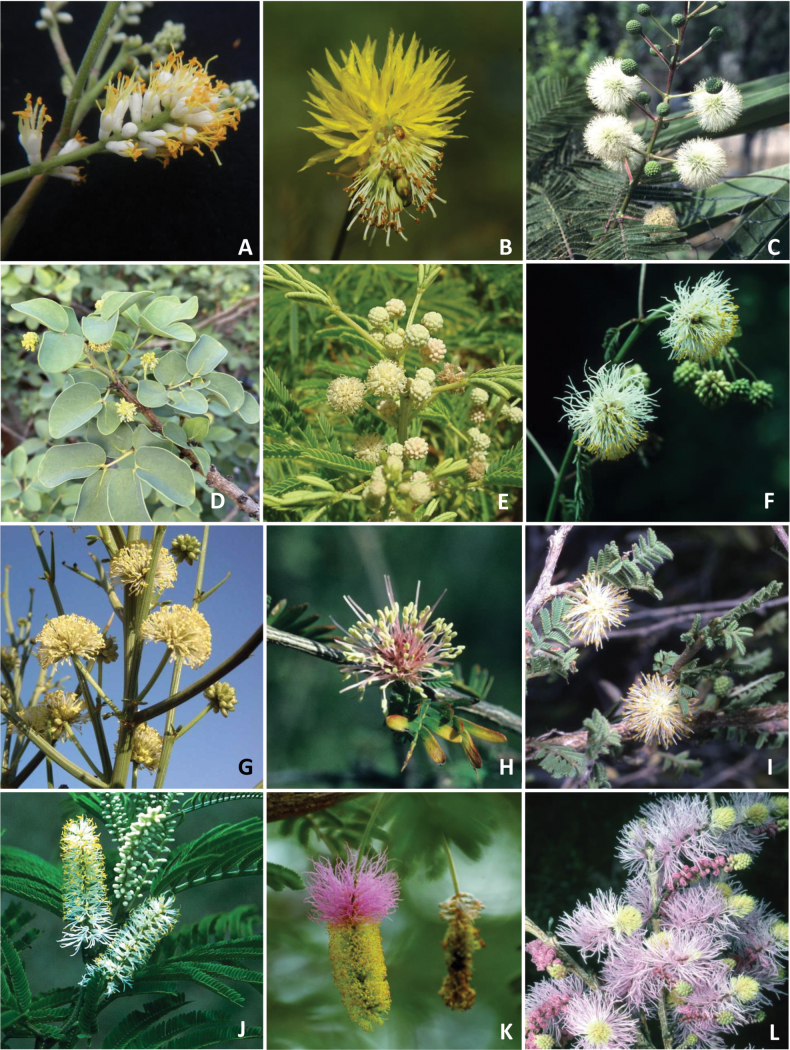
Habit and growth forms of genera of the Dichrostachys clade **A** herbaceous to weakly woody thicket of shrubby *Neptuniaplena* (L.) Benth. in seasonally inundated ruderal habitat, Bolivia (*Särkinen 2159*) **B, C** medium-sized to large trees of *Lemurodendroncapuronii* Villiers & P. Guinet, north-western Madagascar (*Koenen 429*) **D** small tree of *Leucaenamatudae* (Zárate) C.E. Hughes in seasonally dry tropical forest, Guerrero, Mexico (*Hughes 2153*) **E** shrub of *Kanaloakahoolawensis* Lorence & K.R. Wood cultivated, National Tropical Botanical Garden, Kaua’i, Hawaii **F** small treelet of *Desmanthusfruticosus* Rose, Baja California, Mexico (*Hughes 1532*) **G** prostrate woody suffrutex of *Desmanthusacuminatus* Benth., Potosi, Bolivia (*Hughes 2314*) **H** shrub of *Mimozyganthuscarinatus* (Griseb.) Burkart, Santa Cruz, Bolivia (*Hughes 2476*) **I** shrub of *Prosopidastrumglobosum* (Gillies ex Hook. & Arn.) Burkart, Mendoza, Argentina **J** shrublet of *Calliandropsisnervosa* (Britton & Rose) H.M. Hern. & P. Guinet, Puebla, Mexico (*Hughes 1784*) **K** small treelet of *Dichrostachyscinerea* (L.) Wight & Arn., Kruger National Park, South Africa **L** small treelet of *Alantsilodendronpilosum* Villiers, S. Madagascar (*Luckow 4162*) **M** treelet of *Gagnebinapterocarpa* (Lam.) Baill., Madagascar **N** shrub of *Dichrostachysvenosa* Villiers, in dry spiny forest, southern Madagascar. Photo credits **A, D–H, J** CE Hughes **B, C** E Koenen **I** Estepa Patagónica **K** B Dupont **L** M Luckow **M** C Commenge naturetropicale **N** D Du Puy.

**Figure 145.**
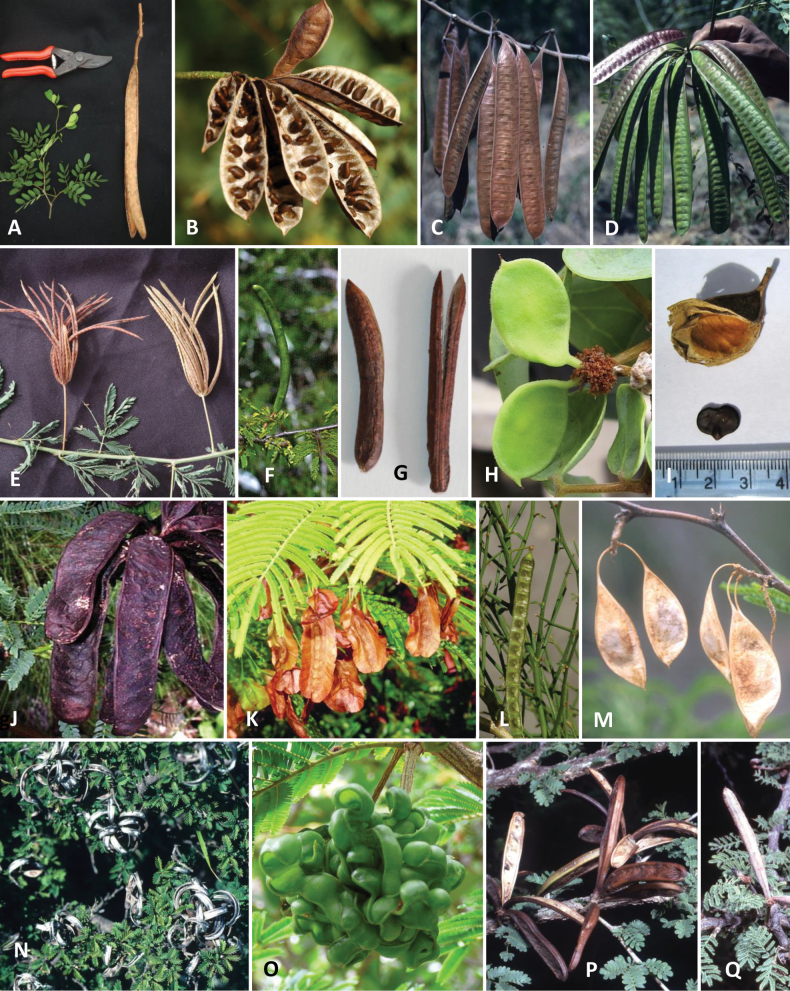
Inflorescences and flowers of genera of the Dichrostachys clade **A***Lemurodendroncapuronii* Villiers & P. Guinet (*Koenen 435*) **B** inflorescence of *Neptuniaplena* (L.) Benth., basal flowers sterile with showy yellow petaloid staminodia (*Wood 26650*) **C** inflorescence of *Leucaenaesculenta* (Sessé & Mociňo ex DC.) Benth. (*Hughes 1326*) **D** inflorescences and tergeminate leaves of *Kanaloakahoolawensis* Lorence & K.R. Wood **E** fascicles of capitate inflorescences in axils of coevally developing leaves, *Schleinitziafosbergii* Nevling & Niezgoda **F** inflorescences of *Desmanthusbicornutus* S. Watson showing basal sterile flowers with exserted white showy staminodia (*Hughes 1526*) **G** capitate inflorescences of *Prosopidastrumglobosum* (Gillies ex Hook. & Arn.) Burkart **H** semi-spherical capitate inflorescences of *Calliandropsisnervosa* (Britton & Rose) H.M. Hern. & P. Guinet (*Hughes 1784*) **I** inflorescence of *Alantsilodendronramosum* Villiers **J** spicate inflorescences of *Gagnebinapterocarpa* (Lam.) Baill., the basal flowers sterile with showy white staminodia **K** spicate inflorescence of *Dichrostachyscinerea* (L.) Wight & Arn., basal flowers sterile with showy pink staminodia **L** compact spikes of *Dichrostachysakataensis* Villiers the basal flowers sterile with showy pale pink staminodia. Photo credits **A** E Koenen **B, C, F, H, J, K** CE Hughes **D** A Palomino **E** Lauren Gutierrez **G** D Cabral, Flora Mondocina **I, L** D Du Puy.

**Figure 146.**
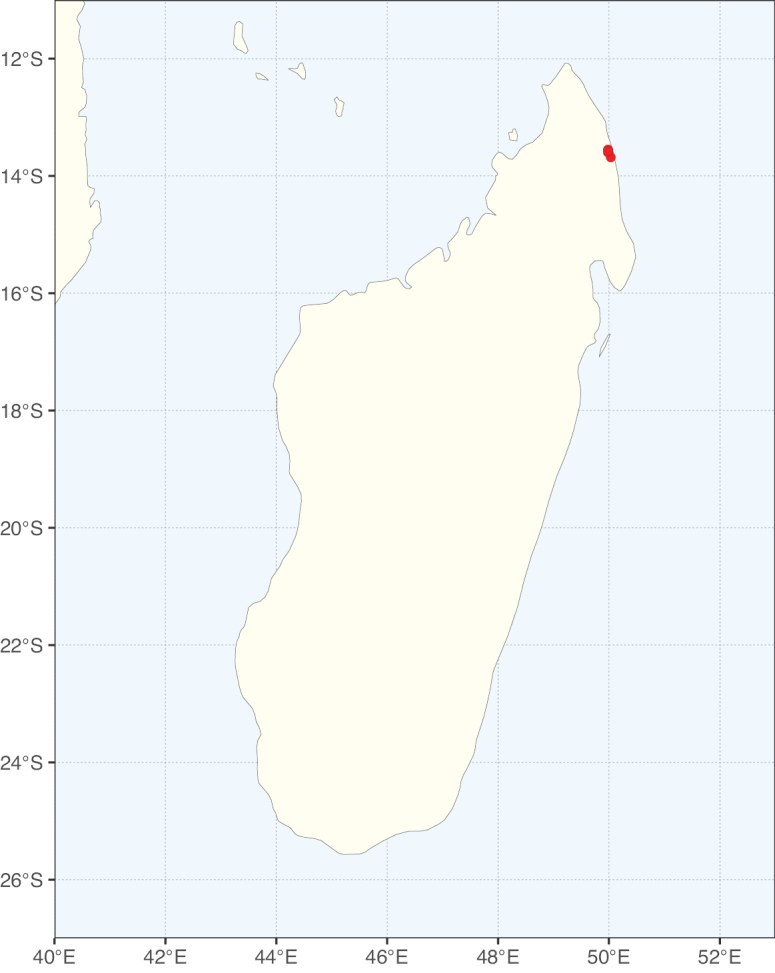
Fruits of genera of the Dichrostachys clade **A** leaves and pod of *Lemurodendroncapuronii* Villiers & P. Guinet, north-eastern Madagascar (*Koenen 429*) **B***Neptuniaplena* (L.) Benth. fruits dehiscing along the dorsal suture (*Wood 26650*) **C** ripe pods of *Leucaenacruziana* Britton & Rose, Oaxaca, Mexico (*Hughes 1300*) **D** cluster of unripe pods on a single inflorescence of Leucaenaleucocephala(Lam.)de Witsubsp.glabrata (Rose) S. Zárate, Honduras (*Hughes 1557*) **E** ripe pods of *Desmanthusleptophyllus* Kunth dehiscing along both sutures (*Hughes 3098*) **F** unripe fruit of *Mezcalabalsensis* (J.L. Contr.) C.E. Hughes & J.L. Contr., held erect, Guerrero, Mexico **G** ripe fruits of *Mezcalabalsensis* dehiscing from the apex along both sutures **H, I** unripe and ripe fruits of *Kanaloakahoolawensis* Lorence & K.R. Wood, cultivated at the Olinda rare plant propagation facility, Maui, Hawaii, USA **J** ripe fruits of *Schleinitziainsularum* (Guilemin) Burkart, the valves tardily splitting along both sutures but indehiscent **K** winged fruits of *Gagnebinapterocarpa* (Lam.) Baill., Madagascar **L** unripe, lomentiform fruits of *Prosopidastrumglobosum* (Gillies ex Hook. & Arn.) Burkart which break up into one- or few-seeded fragments when ripe **M** ripe fruits of *Mimozyganthuscarinatus* (Griseb.) Burkart, S Bolivia (*Hughes 2476*) **N** ripe fruits, elastically dehisced from the apex, *Calliandropsisnervosa* (Britton & Rose) H.M. Hern. & P. Guinet, Puebla, Mexico (*Hughes 1784*) **O** unripe coiled fruits of *Dichrostachyscinerea* (L.) Wight & Arn. **P** ripe fruits dehiscing elastically from the apex, *Dichrostachysvenosa* Villiers, southern Madagascar **Q** ripe fruits of *Alantsilodendronpilosum* Villiers, Madagascar. Photo credits **A** E Koenen **B–E, M, N** C Hughes **F, G** J Luis Contreras **H, I** A Palomino **J** G McCormack, Cook Islands Biodiversity **K, P, Q** D Du Puy **L** I Specogno, Flora Mendocina **O** Atamari (CC-BY-SA-3.0).

While a close relationship among the genera of the Dichrostachys and Leucaena subclades has long been recognised, the Mimozyganthus subclade was identified and placed with the Dichrostachys and Leucaena groups by Luckow et al. (2005), and brought together *Mimozyganthus*, *Piptadeniopsis* and *Prosopidastrum* for the first time. These three genera have divergent flower morphologies and pollen types, but are united by vegetative traits, geography and ecology, including shrubby habit (Fig. 144H, I), green photosynthetic shoots (Figs 145G, 146L), armature (stipular spines and, in some species, spinescent shoots), a distribution concentrated in the Argentinian–Paraguay–southern Bolivia Chaco and Monte vegetation, and a predilection for seasonally dry and arid ecologies.

The presence of heteromorphic inflorescences (Fig. 145), with variable proportions of sterile, functionally staminate and hermaphrodite flowers, and sometimes flowers with exserted showy staminodia at the base (*Desmanthus*, *Dichrostachys*, *Gagnebina*, *Neptunia*; Fig. 145B, F, J, K, L), are characteristic of this clade, albeit not confined to it in Mimoseae, nor universal within it. There is also considerable variation in pollen unit (monads and different types of polyads) which appears to be evolutionarily unconstrained across the Dichrostachys clade and variable even within genera (e.g., Hughes 1997), as well as the variable presence or absence of anther glands. All of these floral traits are evidently evolutionarily labile across the clade. Beyond a tendency to andromonoecy expressed as more functionally staminate flowers per inflorescence, functionally staminate inflorescences or occasionally functionally staminate individuals, this striking variability and evolutionary lability in pollen unit, proportions of sterile, functionally staminate and hermaphrodite flowers, showy staminodia and anther glands, remain poorly understood in terms of the reproductive biology of the species in this clade. Several species of several genera also show a propensity for high fruit set on a single inflorescence (Fig. 146B, D, E, O; some *Desmanthus*, *Dichrostachys*, *Leucaena*, and *Neptunia*), which may be attributed to selfing.

Fruits and seed dispersal mechanisms are diverse and apparently also evolutionarily labile, including pods inertly dehiscent along one or both sutures, sometimes tardily so and the valves remaining attached (in *Schleinitzia*; Fig. 146J), elastically dehiscent from the apex, the valves recurving (Fig. 146G, N, P; *Mezcala*, *Calliandropsis*, *Alantsilodendron* and some *Dichrostachys*), or lomentiform and breaking up into 1-seeded articles (Fig. 146L; *Prosopidastrum* and *Piptadeniopsis*), and fruits and seeds likely wind or water dispersed (*Gagnebina*, *Lemurodendron*, *Mimozyganthus* and *Piptadeniopsis*), the seeds of the latter three genera are thin and lack pleurograms.

These apparently complex and highly labile trajectories of fruit and flower trait evolution are likely attributable, at least in part, to reticulation given that polyploidy appears to be prevalent across the Dichrostachys clade. At least two ancient whole genome duplications are likely associated with this clade, one subtending the genus *Leucaena* (Govindarajulu et al. 2011a), the other probably an allopolyploid event subtending the genus *Schleinitzia* (Ringelberg et al. unpubl. data). In addition, high numbers of gene duplications associated with the branch subtending the genus *Lemurodendron* are indicative of possible polyploidy (Ringelberg et al. unpubl. data). Finally, there is extensive variation in chromosome numbers and molecular evidence for multiple instances of neopolyploidy in several genera including *Dichrostachys*, *Leucaena* and *Neptunia* (Goldblatt 1981b; Govindarajulu et al. 2011a). This apparent propensity for whole genome duplication and the full extent and details of polyploidy across the clade as a whole, remain poorly understood.

Recent re-delimitation to ensure generic monophyly has led to segregation of the monospecific *Mezcala* to deal with the non-monophyly of *Desmanthus* (Hughes et al. 2022c). In addition, segregation of a new Madagascan genus, and consequent re-circumscription of *Gagnebina*, *Dichrostachys* and *Alantsilodendron* will deal with the current non-monophyly of *Dichrostachys* and *Alantsilodendron* (Fig. 143) in a forthcoming monograph of this clade (Luckow, in prep.). Maintenance of *Piptadeniopsis* and *Prosopidastrum* (which are robustly supported as sister genera subtended by a long phylogenetic branch – Fig. 143) as separate genera was questioned by Luckow et al. (2005) given the many similarities in fruits and flowers shared by these two genera, but they are retained here based on differences in pollen and habit.

None of the genera of the Dichrostachys clade are species-rich; only *Leucaena* and *Desmanthus* have more than 20 species; six genera are monospecific. The majority of genera and species are concentrated in seasonally dry tropical forests of Madagascar and the Neotropics: *Alantsilodendron*, *Gagnebina* and *Dichrostachys* mainly in drier parts of Madagascar, and *Calliandropsis*, *Desmanthus*, *Leucaena*, *Mezcala*, *Mimozyganthus*, *Piptadeniopsis* and *Prosopidastrum* mainly in the seasonally dry Neotropics. This amphi-Atlantic distribution largely confined to highly disjunct areas of seasonally dry tropical forests and scrublands has been interpreted as the result of trans-continental phylogenetic biome conservatism across the succulent biome sensu Schrire et al. (2005a) indicative of a long history in seasonally dry tropical regions (Lavin and Luckow 1993; Lavin et al. 2004; Ringelberg et al. 2020). The genera of the mainly Madagascan Dichrostachys subclade are separated by very short branches and show high levels of gene tree conflict in phylogenomic analyses (Fig. 143; Ringelberg et al. 2022) likely indicative of a rapid evolutionary radiation.

### ﻿ Lemurodendron

Taxon classificationPlantaeFabalesFabaceae

Villiers & P. Guinet, Bull. Mus. Natl. Hist. Nat., B, Adansonia 11(1): 3. 1989.

**Figure 147.**
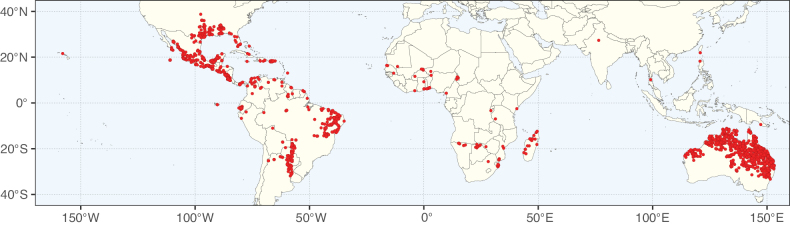
Distribution of *Lemurodendron* based on quality-controlled digitised herbarium records. See Suppl. material 1 for the source of occurrence data.

Figs 144 , 145 , 146 , 147

#### Type.

*Lemurodendroncapuronii* Villiers & P. Guinet

#### Description.

Unarmed trees to 30 m and 1 m stem diameter (Fig. 144B, C); brachyblasts absent. **Stipules** caducous. **Leaves** bipinnate, petiole with discoid glands between pinnae; pinnae 1–2 pairs; leaflets 2–4 pairs per pinna, obovate, slightly asymmetrical, the apex rounded and notched to subtruncate, mucronate, glabrous, midvein more or less central, with 3–5 pairs of lateral veins, the basal pair very long. **Inflorescences** loose-flowered, short spikes (Fig. 145A), combined into a terminal panicle. **Flowers** with the sepals valvate in bud, petals valvate, basal flowers often sterile, apical flowers hermaphrodite; calyx, scarcely lobed, porcelain white, petals connate to near tips, obovate, the free tips subacute, pure white; basal flowers with staminodes 10 or fewer, filiform, or long (ca. 7 mm) and flattened within the same flower, gynoecium reduced; apical flowers hermaphrodite with 10 free stamens, anthers ovoid with a caducous apical gland, ovary subsessile, narrow ellipsoid, ca. 2 mm long, style slender, 3–4 mm long, stigma tubular and flared; pollen in acalymmate tetrahedral tetrads. **Fruits** narrowly linear-oblong, strap-like, straight (Fig. 146A), held erect above shoots, (8) 15–35 × 2–2.5 cm, ca. 10-seeded, the base cuneate, the apex obtuse to acute, valves glabrous, the blackish exocarp breaking away, dehiscent along both sutures. **Seeds** broadly winged, oblong, 30–50 × 16–24 mm including the 5–10 mm broad wing, testa lacking a pleurogram.

#### Chromosome number.

Unknown, but many gene duplications suggestive of a polyploid (Ringelberg et al. unpubl. data).

#### Included species and geographic distribution.

Monospecific (*L.capuronii*), a very narrowly restricted endemic in north-eastern Madagascar, known from just a handful of collections from the 1950s and 1960s, but recollected in 2014 from south of Vohemar, Antsiranana (Fig. 147).

#### Ecology.

Mixed lowland evergreen and deciduous forest on igneous rock (gabbro), to 200 m elevation. Seeds likely wind-dispersed.

#### Etymology.

From Greek, *lemur* and -*dendron* (= tree), in reference to the endemic distribution of the genus in Madagascar where extant lemurs are also endemic.

#### Human uses.

The wood is locally harvested for making charcoal, potentially threatening the survival of this globally very rare genus (Erik Koenen, field observation).

#### Notes.

*Lemurodendron* is sister to *Neptunia* in recent phylogenomic analyses (Koenen et al. 2020a), a perhaps surprising result given the disparate morphologies of these two genera, but support for this relationship is robust (Fig. 143; Ringelberg et al. 2022). The glossy pure white sepals and petals of *Lemurodendron* flowers are unique within Caesalpinioideae and the erect disposition of the long strap-like pods is unusual. Winged seeds are rare within the Dichrostachys clade, found only in *Lemurodendron* (broadly-winged seeds) and *Mimozyganthus* (very narrowly-winged seeds).

#### Taxonomic references.

Villiers (2002); Villiers and Guinet (1989), including illustration.

### ﻿ Neptunia

Taxon classificationPlantaeFabalesFabaceae

Lour., Fl. Cochinch 2: 653. 1790.

**Figure 148.**
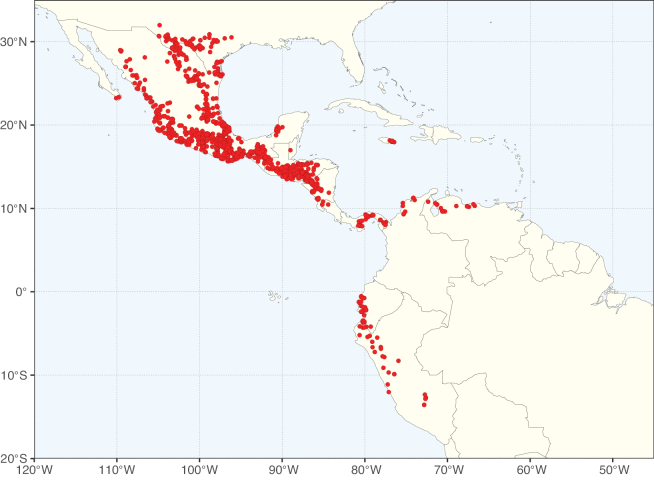
Distribution of *Neptunia* based on quality-controlled digitised herbarium records. See Suppl. material 1 for the source of occurrence data.

Figs 144 , 145 , 146 , 148

Hemidesmas Raf., Sylva Tellur.: 119. 1838. Type: Hemidesmasplenus (L.) Raf. [≡ Neptuniaplena (L.) Benth. (≡ Mimosaplena L.)]

#### Type.

*Neptuniaoleracea* Lour.

#### Description.

Unarmed herbaceous perennials from a woody taproot (Fig. 144A), or an annual (*N.major* (Benth.) Windler), sometimes a floating aquatic with enlarged, spongy, aerenchymatous stems, prostrate to erect to 1–2 (3) m. **Stipules** broadly ovate, occasionally narrow, striate with raised nervation when dried, usually persistent, occasionally caducous; brachyblasts absent. **Leaves** bipinnate, petiole sulcate, petiolar glands present or absent, crateriform; pinnae 1–11 pairs, opposite, pseudostipels sometimes present at base of pinnae; leaflets opposite, often sensitive, 6–43 pairs per pinna, linear to narrowly oblong, nearly sessile, venation brochidodromous, obscure other than central midvein, secondary venation reticulate, sometimes raised abaxially. **Inflorescences** of condensed cylindrical to obovate or ellipsoid spikes or capitula, 1–2 in leaf axils, pedunculate, with 2–3 lanceolate-linear to ovate-cordate bracts scattered on the peduncle, or these sometimes lacking, composed of sterile staminodial flowers proximally, hermaphrodite flowers apically, and functionally staminate flowers in between (Fig. 145B) (sterile flowers lacking in *N.lutea* (Leavenw.) Benth.), bracteoles subtending each flower linear, carinate, persistent. **Flowers** sessile; hypanthium absent; sepals valvate in bud; petals valvate; sterile flowers similar to fertile ones but smaller, lacking anthers and gynoecium, staminodia petaloid, yellow; hermaphrodite flowers with the calyx cupulate, petals 5, free, yellow, white, or pale green; stamens 5 or 10, filaments not flattened, free, anthers dorsifixed, usually bearing a semi-persistent apical claviform gland, absent in some species; pollen of tricolporate monads, exine striate; ovary sessile, usually glabrous, stigma funnelform. **Fruits** oblong, or when monospermous circular to broadly elliptical, dorsiventrally flattened, stipitate, often in clusters of 6 or more per inflorescence, 1–7 (20)-seeded, valves chartaceous, inertly dehiscent through both sutures, rarely through only 1 suture (Fig. 146B) or indehiscent (notably when monospermous), not internally septate, epicarp papery, dark brown, endocarp papery, buff-coloured, mesocarp absent. **Seeds** transverse or rarely oblique, ovate, olivaceous, smooth, unwinged, testa hard, pleurogram present.

#### Chromosome number.

*Neptunia* species comprise a polyploid series with diploids, tetraploids and hexaploids: 2*n* = 26, 28, 52, 56, 72, 78 (Turner and Fearing 1960b; Poggio et al. 2008; Santos-Silva et al. 2020).

#### Included species and geographic distribution.

Ca. 22 species (10 new Australian species added by Bean 2022), pantropically distributed (Fig. 148). The genus is most speciose in Australia (14 endemics); two species in south-west USA, two in Asia, and one endemic to Brazil. The aquatic *N.oleracea* Lour. is widespread throughout the tropics while *N.plena* is most common in the Americas, but is also found in Australia and South East Asia, where it is almost certainly introduced.

#### Ecology.

*Neptunia* is unique among Caesalpinioideae in having two species with an aquatic habit, occurring by dams, or in lagoons and swampy areas with little water movement. The remaining species are terrestrial but sometimes grow in seasonally inundated riverine or other ruderal habitats. It is also one of relatively few functionally herbaceous perennial Mimoseae (a habit found elsewhere in *Desmanthus*, *Mimosa*, and *Entada*). Although the majority of species are tropical, the genus also extends to warm temperate regions in the southern USA. In Australia, where the genus is most diverse, *Neptunia* species are generally found on heavy clay vertisols in grassland and open eucalypt woodland (Bean 2022). *Neptuniaamplexicaulis* Domin is one of the strongest hyperaccumulators of the metalloid element selenium (Se) in Queensland, Australia (Bean 2022).

#### Etymology.

Named after the Roman god of fresh water and the sea, Neptune, in reference to the unusual aquatic habit of two species, including the type, in the genus.

#### Human uses.

*Neptuniaoleracea* is a nutritious vegetable rich in vitamins and is cultivated as a food plant in Asia and Africa, where young shoots and fruits are eaten raw or cooked as a green vegetable (Fern 2023); this species is also classified as an invasive pest in Australia (Queensland Government 2009).

#### Notes.

*Neptunia* has long been considered closely allied to members of the Dichrostachys Group (Luckow 1993) and is robustly supported as sister to the endemic Madagascan *Lemurodendron* (Fig. 143; Koenen et al. 2020a; Ringelberg et al. 2022). This sister-group relationship of *Neptunia* and *Lemurodendron* is perhaps unexpected given their divergent morphologies, but arguably *Neptunia* is unlike any other mimosoid because of its herbaceous (semi-) aquatic lifestyle. *Neptunia* can be distinguished from other genera of the Dichrostachys clade by its herbaceous habit and by its yellow petaloid staminodia on the basal sterile flowers. Some (and possibly all) Australian species of *Neptunia* have sensitive leaves, but, unlike in the sensitive-leaved species of *Mimosa*, the movement is rather slow and the leaflets often do not fold completely (Bean 2022).

The ten new species described by Bean (2022) remain to be included in a phylogenetic analysis or to be studied cytologically. Phylogenomic analyses on *Neptunia* are in progress and will likely shed light on species relationships and the origins of polyploidy in the genus (Ellie Becklund, University of Ohio, USA, pers. comm.).

#### Taxonomic references.

Bean (2022); Cowan (1998); Nielsen (1992); Santos-Silva et al. (2020), illustrations; Windler (1966); Windler and Windler (1974).

### ﻿ Leucaena

Taxon classificationPlantaeFabalesFabaceae

Benth., J. Bot. (Hooker) 4: 416. 1842.

**Figure 149.**
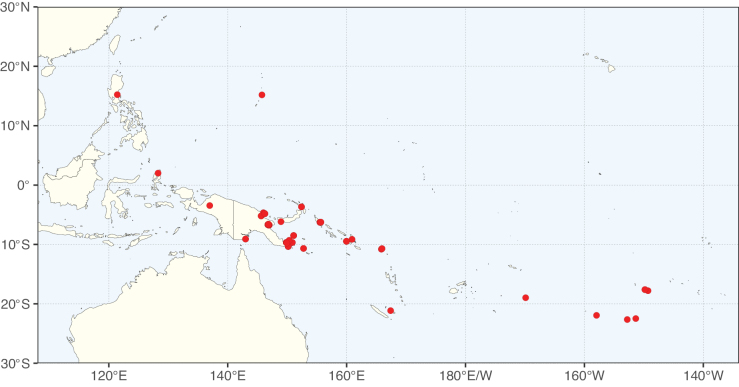
Distribution of *Leucaena* based on quality-controlled digitised herbarium records. Note that the pantropically cultivated and widely introduced species *Leucaenaleucocephala* (Lam.) de Wit, whose true native distribution remains unknown (Hughes 1998a), is not included in the map. See Suppl. material 1 for the source of occurrence data.

Figs 144 , 145 , 146 , 149

Ryncholeucaena Britton & Rose, Fl. N. Amer. 23: 130. 1928. Type: Ryncholeucaenagreggii (S. Watson) Britton & Rose [≡ Leucaenagreggii S. Watson]
Caudoleucaena Britton & Rose, Fl. N. Amer. 23: 130. 1928. Type: Caudoleucaenaretusa (Benth.) Britton & Rose [≡ Leucaenaretusa Benth.]

#### Type.

*Leucaenadiversifolia* (Schlecht.) Benth. [≡ *Acaciadiversifolia* Schlecht.]

#### Description.

Unarmed small or medium-sized trees, 2–15 (20) m (Fig. 144D), brachyblasts absent. **Stipules** persistent or caducous, ovate or subulate. **Leaves** bipinnate, extrafloral nectaries between proximal pair of pinnae, sometimes between other pinnae pairs and on pinnular rachis, to 75 on a single leaf (*L.trichandra* (Zucc.) Urban); pinnae 1–20 (60) pairs, opposite; leaflets 3–65 (85) pairs per pinna, variable in size and shape (linear, oblong, elliptic). **Inflorescences** capitate, peduncle with a distal, or sub-distal involucel of united bracts, 1 (4) in leaf axils or on 1–2-branched terminal panicles exserted beyond the leaves (Fig. 145C), composed mainly of hermaphrodite flowers, sterile flowers with staminodia absent; bracteoles subtending each flower bud persistent, peltate, sometimes exserted in bud, round, lanceolate or caudate. **Flowers** with 5 sepals, valvate in bud, calyx obconic, tubular or campanulate or united along mid-portion; petals 5, valvate, usually free, white or pale green; stamens 10, filaments usually white, but occasionally pink, yellow or reddish, anthers usually pilose, occasionally glabrous, usually eglandular, but sometimes the connective with a small rounded or hooded apiculum; pollen highly variable among species, occurring in monads, acalymmate polyads, or calymmate tetrahedral tetrads, tricolporate or pantoporate, exine smooth, psilate or punctate; ovary sessile or sub-sessile, glabrous or hairy, stigma narrowly funnelform. **Fruits** linear, flattened, (5) 10–20 (26)-seeded, valves chartaceous or coriaceous, inertly dehiscent along one or usually both sutures (Fig. 146C, D). **Seeds** transverse or occasionally oblique in pod, ovate to rhomboidal, pleurogram U-shaped.

#### Chromosome number.

2*n* = 52, 56, 104, 112 (Cardoso et al. 2000); whole genus paleopolyploid; five documented neo-allopolyploid species (Govindarajulu et al. 2011a); one relatively common named sterile triploid, L.×mixtec C.E. Hughes & S.A. Harris (Hughes and Harris 1994) and one named homoploid hybrid, L.×spontanea C.E. Hughes & S.A. Harris, at the tetraploid level (Hughes and Harris 1998).

#### Included species and geographic distribution.

Twenty-four species plus two named hybrids (Hughes 1998a; Govindarajulu et al. 2011b). Mexico 11 endemic species with two extending to the USA (Texas and New Mexico) and four species to Central America; Central America five endemic species (Guatemala to Panama); one species in South America; one species pantropically introduced (Fig. 149).

#### Ecology.

Seasonally dry tropical forest and semi-arid thorn scrub; two species extending to warm temperate scrublands and one species to rainforests. Seed dispersal passive. All species deciduous. Nodulating, symbiosome present. Bee-pollinated (*Xylocopa* and other smaller bees).

#### Etymology.

From *Leucaino* (Latin = becoming white), probably referring to the predominantly white stamen filaments.

#### Human uses.

Unripe seeds of 13 species are used as minor foods in Mexico and are widely cultivated and incipiently domesticated in backyards in south-central Mexico (Hughes 1998b), spawning spontaneous interspecific hybrids (Hughes et al. 2007). One species, *L.leucocephala* (Lam.) de Wit, cultivated pantropically, naturalised and weedy in many places. Used in tropical agroforestry especially for livestock fodder (dubbed the alfalfa of the tropics), green manure, poles, firewood and soil conservation (Hughes 1998b).

#### Notes.

The Leucaena subclade (*Leucaena*, *Schleinitzia*, *Mezcala*, *Kanaloa* plus *Desmanthus*) is equivalent to the informal Leucaena group of Hughes et al. (2003). *Leucaena* can be distinguished from the other genera within this subclade by its hairy anthers which are present in all but three species and which are unique within Mimoseae. The genus is the focus of various genome sequencing initiatives (Dugas et al. 2015; Kovar et al. 2018).

#### Taxonomic references.

Govindarajulu et al. (2011b); Hughes (1998a, b), with illustrations; Hughes et al. (2007); Zárate (1994).

### ﻿ Schleinitzia

Taxon classificationPlantaeFabalesFabaceae

Warb. ex J.C. Willis, Dict. Fl. Pl., ed. 4: 594. 1919.

**Figure 150.**
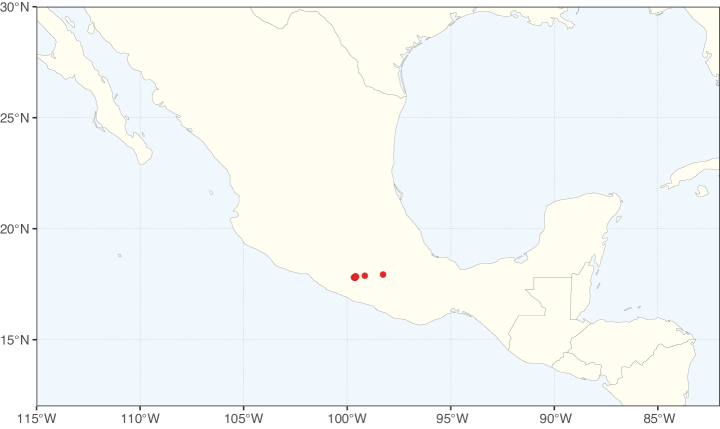
Distribution of *Schleinitzia* based on quality-controlled digitised herbarium records. See Suppl. material 1 for the source of occurrence data.

Figs 145 , 146 , 150

#### Type.

*Schleinitzianovoguineensis* (Warb.) L.I. Nevling & C.J. Niezgoda [≡ *Piptadenianovoguineensis* Warb.]

#### Description.

Trees or shrubs, 2–20 (–25) m, unarmed, young stems angled with corky ridges, brachyblasts absent. **Stipules** acicular to triangular, persistent. **Leaves** bipinnate; petiole ventrally sulcate, nectary at apex of petiole or mid-petiole, additional nectaries between distal-most and sometimes all pinnae, crateriform to urceolate; pinnae 4–30 pairs, opposite to subopposite; leaflets 20–60 pairs per pinna, opposite, linear to oblong; venation obscure except central midvein. **Inflorescence** capitate, globose, clustered 2–7 per node in terminal, largely efoliate panicles (Fig. 145E), peduncle bearing an involucel of fused bracts below the inflorescence; bracteoles subtending each flower peltate, carinate, semi-persistent. **Flowers** either all hermaphrodite, all functionally staminate, or functionally staminate proximally and hermaphrodite distally, sterile flowers with staminodia absent, sessile, base of calyx prolonged into a pseudopedicel; hypanthium absent; sepals valvate in bud, calyx obconic, ¾ length of petals; petals valvate, 5, free, membranous, 1-nerved; stamens 10; anthers dorsifixed, bearing a caducous apical claviform gland; pollen in tetrahedral tetrads of tricolporate grains, or [*Schleinitziamegaladenia* (Merr.) P. Guinet & I.C. Nielsen] loose polyads of 5 associated tetrads of porate grains; ovary sessile, oblong, glabrous, stigma funnelform. **Fruits** somewhat upright on stout peduncles, often 5–8 per infructescence, straight, strongly compressed, linear-oblong (Fig. 146J), 8–20-seeded, seeds transversely inserted; valves coriaceous, indehiscent, sutural ribs separating but valves remaining attached; epicarp chartaceous, dark brown; endocarp fibrous, smooth, straw-coloured forming partitions between seeds, mesocarp spongy. **Seeds** narrow ovate to oblong, smooth, testa dark brown to black, pleurogram deeply U-shaped.

#### Chromosome number.

2*n* = 52 or 54 (Nevling and Niezgoda 1978; Goldblatt 1981b); whole genus apparently paleopolyploid.

#### Included species and geographic distribution.

Four species disjunctly distributed across the western Pacific basin in New Guinea, the Philippines, Melanesia, Micronesia and Polynesia (Fig. 150).

#### Ecology.

In lowland rainforests, especially common in secondary vegetation near the coast, and in littoral habitats above high-tide limits on calcareous substrates, especially coral limestone and coral sand, and often forming thickets on coastal beach strands (Fosberg and Stone 1965).

#### Etymology.

Named in honour of Vice Admiral George Schleinitz (1834–1910), governor of German New Guinea (now part of Papua New Guinea).

#### Human uses.

The wood is favoured for cremations, handicrafts and frames for fish nets (Nevling and Niezgoda 1978).

#### Notes.

*Schleinitzia* is characterised by a fused whorl of bracts subtending the capitate inflorescence, similar to those of *Kanaloa* and *Leucaena*. Pollen in calymmate tetrahedral tetrads is also seen in *Lemurodendron*, *Leucaena* and *Mezcala*. *Schleinitzia* differs from related genera in having the flowers borne on pseudopedicels instead of being sessile.

#### Taxonomic references.

Nevling and Niezgoda (1978), including illustrations.

### ﻿ Mezcala

Taxon classificationPlantaeFabalesFabaceae

C.E. Hughes & J.L. Contr., PhytoKeys 205: 197. 2022.

Figs 146 , 151

#### Type.

*Mezcalabalsensis* (J.L. Contr.) C.E. Hughes & J.L. Contr. [≡ *Desmanthusbalsensis* J.L. Contr.]

#### Description.

Unarmed multi-stemmed treelet or large shrub to 3.5 m, brachyblasts present, sheathed in persistent stipules. **Stipules** setiform with striate membranous wings. **Leaves** bipinnate, a stipitate nectary between basal pinnae pair; pinnae 2–4 (5) pairs, opposite; leaflets 8–14 pairs per pinna, oblong, opposite. **Inflorescences** capitate, 1–2 per leaf axil, composed of 30–50 functionally sterile, functionally staminate and hermaphrodite flowers, proportions of each flower type variable; bracteoles subtending each flower peltate or deltate setiform. **Flowers** with sepals and petals valvate in bud; each inflorescence with 0–5 sterile flowers proximally, staminodia filamentous, white; similar to the hermaphrodite flowers but smaller and lacking anthers and ovary; functionally staminate flowers 12–30 per inflorescence, lacking an ovary, borne above the sterile flowers; hermaphrodite flowers apical, 5–25 per inflorescence, the calyx obconic, petals 5, free, white or pale green, stamens 10, cream-white, anthers with a minute orbicular gland on a filiform stalk; pollen in tetrahedral tetrads with striate exine ornamentation; ovary sessile, glabrous, stigma narrow funnelform. **Fruits** held erect above shoots, linear-oblong, straight or weakly arcuate, terete or sub-terete, 3–10 cm long, 5–13-seeded, seeds longitudinally inserted, valves initially fleshy becoming woody or sub-woody when ripe, elastically but tardily dehiscent from the apex along both sutures (Fig. 146F, G). **Seeds** square to rhomboidal, pleurogram deeply U-shaped, often asymmetric with unequal arms.

#### Chromosome number.

Unknown.

#### Included species and geographic distribution.

**Figure 151.**
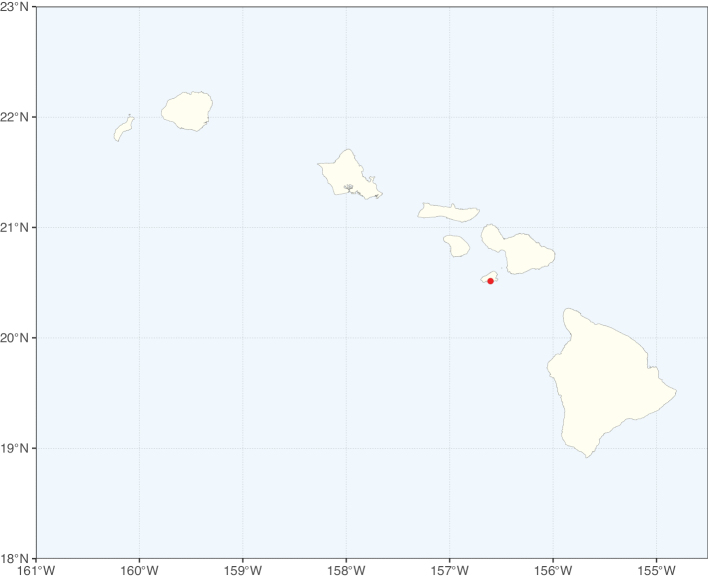
Distribution of *Mezcala* based on quality-controlled digitised herbarium records. See Suppl. material 1 for the source of occurrence data.

Monospecific (*M.balsensis*), narrowly restricted, endemic in the Balsas Depression of Mexico (Fig. 151).

#### Ecology.

Seasonally dry tropical forest and semi-arid thorn scrub on shallow calcareous rocky soils. Seed dispersal passive. Strongly deciduous. Nodulation status unknown. Bee-pollinated (*Xylocopa* and other generalist bee species).

#### Etymology.

Named for the Mezcala culture which blossomed from 700 to 200 BC in the central Balsas Depression in Guerrero Mexico where the genus is endemic.

#### Human uses.

Unknown.

#### Notes.

*Mezcala* was established as a monospecific genus to account for the non-monophyly of *Desmanthus* (Hughes et al. 2022c) by segregating the single species *D.balsensis* which differs from the remaining species of *Desmanthus* in possessing anther glands, pollen in tetrads as opposed to tricolporate monads, and erect woody, as opposed to chartaceous, fruits.

#### Taxonomic references.

Contreras-Jiménez (1986) including an illustration; Hughes et al. (2022b); Luckow (1993).

### ﻿ Kanaloa

Taxon classificationPlantaeFabalesFabaceae

Lorence & K.R. Wood, Novon 4(2): 137. 1994.

**Figure 152.**
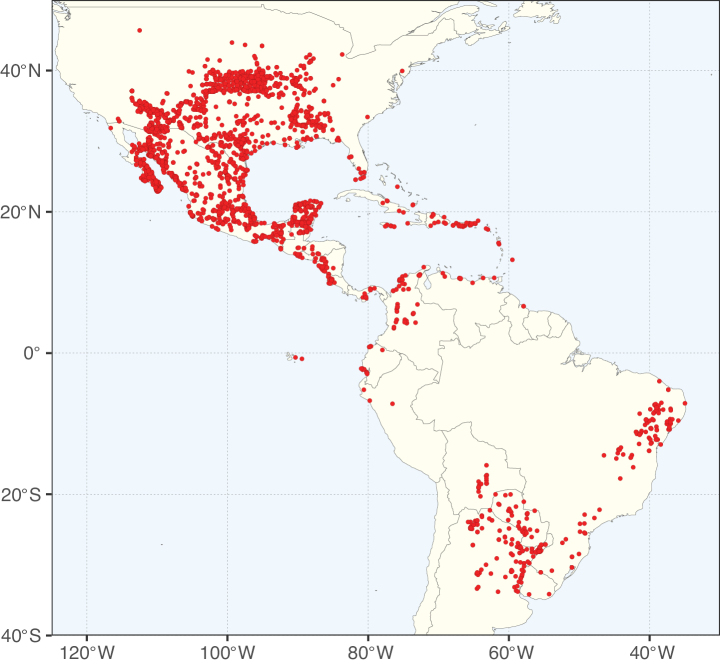
Distribution of *Kanaloa* based on quality-controlled digitised herbarium records. See Suppl. material 1 for the source of occurrence data.

Figs 144 , 145 , 146 , 152

#### Type.

*Kanaloakahoolawensis* Lorence & K.R. Wood

#### Description.

Unarmed shrub, 0.75–1 m, branches dense, decumbent (Fig. 144E), 0.75–1.5 m long, new growth densely brown hirtellous-villosulous; brachyblasts absent. **Stipules** free, ovate, villosulous. **Leaves** bipinnate, a sessile elliptical gland at point of insertion of pinnae (Fig. 145D), pinnae one pair per leaf, leaflets 3 per pinna, a terminal pair and a single proximal leaflet on the abaxial side, leaflets nearly sessile, ovate to elliptic, asymmetrical, venation reticulate, margin entire. **Inflorescence** a globose capitulum (Fig. 145D), 7.0–8.5 mm in diameter, lacking an involucel of united bracts on peduncle, composed of variable numbers of functionally staminate and hermaphrodite flowers, some inflorescences mostly unisexual; sterile flowers bearing showy staminodia absent; bracteoles subtending each flower persistent, deltate or peltate. **Flowers** 20–54 per inflorescence; sepals valvate in bud, connate, the calyx obconic, 5-lobed, pale green, pubescent; petals valvate in bud, 5, free, oblanceolate, inflexed, extremely hirtellous apically, pale green; stamens 10, filaments white, free, anthers dorsifixed, glabrous and eglandular; pollen in spheroidal tricolporate monads, exine rugulate; ovary short, squat, flask-shaped, the stigma in hermaphrodite flowers wide funnelform, anvil-shaped, flanged, peltate. **Fruits** stipitate, up to 4 per inflorescence, monospermous, inertly dehiscent along both margins, obovate or subcircular, plano-compressed, valves coriaceous, 2.4–3.2 × 2–2.3 cm (Fig. 146H, I). **Seed** cordiform (Fig. 146I), pleurogram present.

#### Chromosome number.

2*n* = 28 (Lorence and Wood 1994).

#### Included species and geographic distribution.

Monospecific (*K.kahoolawensis*), narrowly restricted to the island of Kaho’olawe, Hawaii (Fig. 152). When discovered, known from just two plants on a sea stack, now thought to be extinct in the wild and the focus of ex-situ conservation efforts. It is possible that the range previously included other Hawaiian islands in that fossilised pollen from plants likely to be of *Kanaloa* has been found in core samples taken from sinkholes in O‘ahu’s ‘Ewa Plain, Maui, and Kaua‘i’s Makauwahi Cave.

#### Ecology.

Steep rocky cliffs and screes derived from basaltic lava flows. Seed dispersal passive.

#### Etymology.

*Kanaloa* is the name of a Hawaiian deity who according to legend used the island of Kaho’olawe to rest and recoup his energies. According to Lorence and Wood (1994), *Kanaloa* means, “secure, firm, immovable, established, unconquerable..... attributes [which] are certainly essential for this plant to have survived in spite of the severe degradation of the island”, and which will be required more than ever now, given that the genus is likely extinct in the wild and the focus of ex-situ conservation.

#### Human uses.

Unknown.

#### Notes.

The affinities of *Kanaloa* have been in doubt due to lack of definitive support in previous phylogenetic analyses (Hughes et al. 2003; Luckow et al. 2003, 2005), but recent phylogenomic work (Ringelberg et al. 2022) has demonstrated that *Kanaloa* is robustly supported as sister to the re-circumscribed *Desmanthus* (Hughes et al. 2022c). The tergeminate leaves and one-seeded fruits easily distinguish it from its nearest relatives, as does the large peltate stigma, which is reminiscent of the stigma of *Mimozyganthus*.

#### Taxonomic references.

Lorence and Wood (1994), including illustration.

### ﻿ Desmanthus

Taxon classificationPlantaeFabalesFabaceae

Willd., Sp. Pl. 4: 1044. 1806.

**Figure 153.**
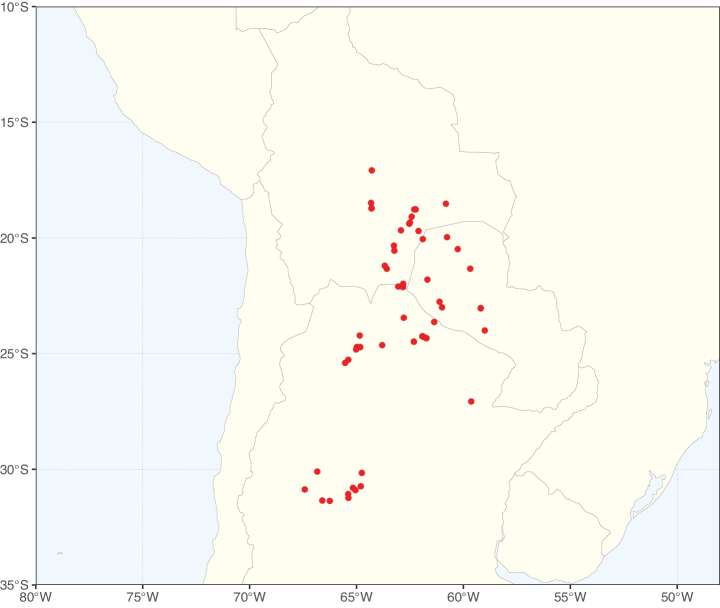
Distribution of *Desmanthus* based on quality-controlled digitised herbarium records. See Suppl. material 1 for the source of occurrence data.

Figs 144 , 145 , 146 , 153

Acuan Medik., Theodora: 63. 1786, nom. rej. vs. Desmanthus Willd. Type: Acuanvirgatum (L.) Medikus [(≡ Desmanthusvirgatus (L.) Willd.)]
Darlingtonia DC., Ann. Sci. Nat. (Paris) 4: 97. 1825. Type: Darlingtoniabrachyloba (Willd.) DC. [≡ Acaciabrachyloba Willd. (≡ Desmanthusillinoensis (Michx.) MacMill. ex B.L. Rob. & Fernald)]

#### Type.

*Desmanthusvirgatus* (L.) Willd. [≡ *Mimosavirgata* L.]

#### Description.

Unarmed, mostly woody shrubs or small trees to 4 m, or functionally herbaceous perennials much-branched from a woody base (Fig. 144F, G), arising from a stout cylindrical or napiform tap root and dying back annually to the base in temperate regions, brachyblasts present in woody species. **Stipules** usually persistent, setiform with a dilated, auriculate base. **Leaves** bipinnate, extrafloral nectaries between proximal pair of pinnae and sometimes other pinnae pairs; pinnae 1–9 (17) pairs, opposite; leaflets 5–45 (55) pairs per pinna, opposite, variable in size and shape (linear, oblong, elliptic). **Inflorescences** capitate, peduncle sometimes with a distal involucel of united bracts, 1 (2) in leaf axils, composed of variable proportions of sterile, functionally staminate, and hermaphrodite flowers, rarely all of one type (Fig. 145F); bracteoles subtending each flower deltate, peltate. **Flowers** with sepals valvate in bud, petals valvate; sterile flowers borne proximally, with elongated showy white, or rarely pink, filamentous staminodia (Fig. 145F), ovary vestigial but like the fertile flowers in other respects; fertile flowers with the calyx cupulate, 5-lobed; petals 5, free, white or pale green; stamens 10 (5 in one species), filaments white or pink, anthers glabrous, lacking an apical gland; pollen in symmetric tricolporate monads, the exine striate, fossulate or foveolate; ovary sessile, glabrous or rarely pubescent, stigma funnelform. **Fruits** linear, flattened, valves chartaceous or coriaceous, seeds oblique or longitudinal, inertly dehiscent along both sutures (Fig. 146E) (indehiscent in one species). **Seeds** ovate to rhomboidal, pleurogram U-shaped.

#### Chromosome number.

2*n* = 28 (Turner and Beaman 1953).

#### Included species and geographic distribution.

23 species. Three endemic species in the USA, with six extending to Mexico, six endemic species in Mexico, with three species extending to South and Central America, one endemic to Argentina, two bicentric in the USA and Argentina, one in the Antilles and north-western South America, one widespread USA to Argentina (Fig. 153). One species, *Desmanthuspernambucanus* (L.) Thell. (often mis-identified as *D.virgatus*), is weedy and widely introduced throughout the tropics.

#### Ecology.

Seasonally dry tropical forest and semi-arid thorn scrub (ca. 10 species); warm temperate scrubland and oak woodland (seven species), three species extending to cool temperate grasslands and woodlands, two species in wet tropics (but absent from the Amazon basin), very often in disturbed open habitats, abandoned pastures and coastal thickets. Seed dispersal passive. Usually deciduous. Nodulating, symbiosome present. Bee-pollinated (*Xylocopa* and generalist bee species).

#### Etymology.

From Greek, *desme* (= bundle) and *anthos* (= flower), in reference to dense capitate inflorescences.

#### Human uses.

Used as ornamentals, livestock fodder and in erosion control; *Desmanthusillinoensis* (Michx.) MacMill. ex B.L. Rob. & Fernald is used for food (leaves, cooked seeds), medicine and is a potential pulse crop (Luckow 1993).

#### Notes.

The delimitation of *Desmanthus* was altered by Hughes et al. (2022b) to account for the non-monophyly of the genus and exclude the morphologically and phylogenetically distinct species *Desmanthusbalsensis*, which was placed in the new genus *Mezcala*.

#### Taxonomic references.

Luckow (1993), including illustrations; Luckow et al. (2003, 2005).

### ﻿ Mimozyganthus

Taxon classificationPlantaeFabalesFabaceae

Burkart, Darwiniana 3: 448. 1939.

**Figure 154.**
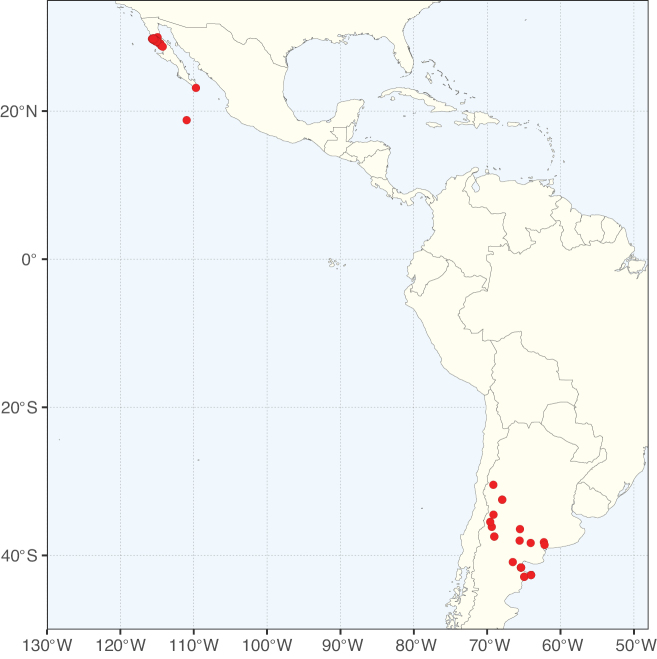
Distribution of *Mimozyganthus* based on quality-controlled digitised herbarium records. See Suppl. material 1 for the source of occurrence data.

Figs 144 , 146 , 154

#### Type.

*Mimozyganthuscarinatus* (Griseb.) Burkart [≡ *Mimosacarinata* Griseb.]

#### Description.

Shrub or small treelet, 3–5 m (Fig. 144H), armed with stipular spines at nodes; young branches zigzagging, glabrate to finely puberulent, brachyblasts present, clothed with persistent stipules and bearing leaves and inflorescences. **Stipules** linear and firm on young growth, forming spines on older branches. **Leaves** bipinnate, borne on brachyblasts or alternate on new growth, petiole shallowly canaliculate, terminating in a caducous spicule; rachis (if present) to 8 mm long; nectaries sessile, orbicular, crateriform, between all pairs of pinnae; pinnae 1–2 (3) pairs, opposite, articulate to the rachis, elongate red glands present at their insertion; leaflets 15–35 pairs per pinna, alternate to subopposite proximally, opposite distally, oblong. **Inflorescences** globose capitula borne in pairs or fours congested on the brachyblasts or axillary on new growth; bracteoles subtending each flower subulate, spiny and greatly exceeding the flowers in bud. **Flowers** all hermaphrodite, 25–35 per inflorescence, sessile; hypanthium absent; sepals 5, imbricate in bud, free to base, indurate and incurved at apex; petals 5, irregularly valvate in bud, sometimes valvate apically and imbricate basally, free, glabrous, greenish; stamens 10, free, anthers dorsifixed, lacking a terminal gland; intrastaminal disc absent; pollen in columellate tricolporate monads, exine verrucate, colpi granular; ovary ovoid, stipitate, glabrous, stigma large, peltate, sub-pentagonal. **Fruits** several per axis, indehiscent, elliptical, acute apically and basally, 3.5–4 × 1–1.5 cm, 1 (2)-seeded, dorsiventrally flattened, pericarp thin, papery, translucent when young, pale brown at maturity, ventral sutural rib flattened and forming a narrow wing (Fig. 146M). **Seeds** dorsiventrally compressed, orbicular in outline, narrowly winged, testa thin, pleurogram absent, chestnut brown.

#### Chromosome number.

2*n* = 28 (Luckow et al. 2005).

#### Included species and geographic distribution.

Monospecific (*M.carinatus*), centred on the Chaco phytogeographic region in north-west and west-central Argentina, north-west Paraguay and south-east Bolivia (Fig. 154).

#### Ecology.

Tropical and subtropical arid and semi-arid Chaco woodlands and adjacent Piedmont seasonally dry scrub and forest, 150–1200 m elevation. The papery marginally-winged fruits suggest that the fruit is likely wind-dispersed.

#### Etymology.

From Greek, *zygo*- (= paired) and -*anthos* (= flower), referring to the inflorescences which arise in the yoke between a pair of spinescent stipules, plus the Greek prefix *mimo*- (= mime or mimic) indicating placement of the genus in what was then subfamily Mimosoideae.

#### Human uses.

Used locally for firewood and lumber; apparently a dye can be extracted from the stems (Fortunato 2005).

#### Notes.

Until recently, *Mimozyganthus* was sometimes considered to be ‘transitional’ between subfamily Caesalpinioideae and the former Mimosoideae and was placed in its own tribe Mimozygantheae (Burkart 1939; Fortunato 1997, 2005). This placement of *Mimozyganthus* was based on three characters more typical of non-mimosoid Caesalpinioideae than of genera of the mimosoid clade: imbricate, as opposed to valvate, aestivation of the sepals, a peltate stigma, and lack of a pleurogram on the seed. However, molecular phylogenetic analyses (Luckow et al. 2005; Koenen et al. 2020a; Ringelberg et al. 2022) have shown that this monospecific genus is indeed a mimosoid, placed in a robustly supported subclade with *Prosopidastrum* and *Piptadeniopsis* within the Dichrostachys clade sensu Koenen et al. (2020a). This placement requires an evolutionary reversal in sepal aestivation. Lack of a pleurogram is best understood as an adaptation often seen in phylogenetically scattered hydrochorous and anemochorous Caesalpinioideae. Although this subclade is geographically coherent in being almost entirely distributed in the Argentinian–Paraguayan–Bolivian Chaco domain and shares similar armature (spinescent stipules) across its three constituent genera, there is remarkable diversity in floral and pollen morphology suggesting strong selection for floral diversity across this subclade. The peltate stigma in *Mimozyganthus* is apparently similar to the anvil-shaped, flanged stigma of *Kanaloa*, although very limited material of *Kanaloa* with fertile flowers is available.

#### Taxonomic references.

Burkart (1939); Luckow et al. (2005), both with illustrations.

### ﻿ Prosopidastrum

Taxon classificationPlantaeFabalesFabaceae

Burkart, Darwiniana 13(2–4): 436. 1964.

**Figure 155.**
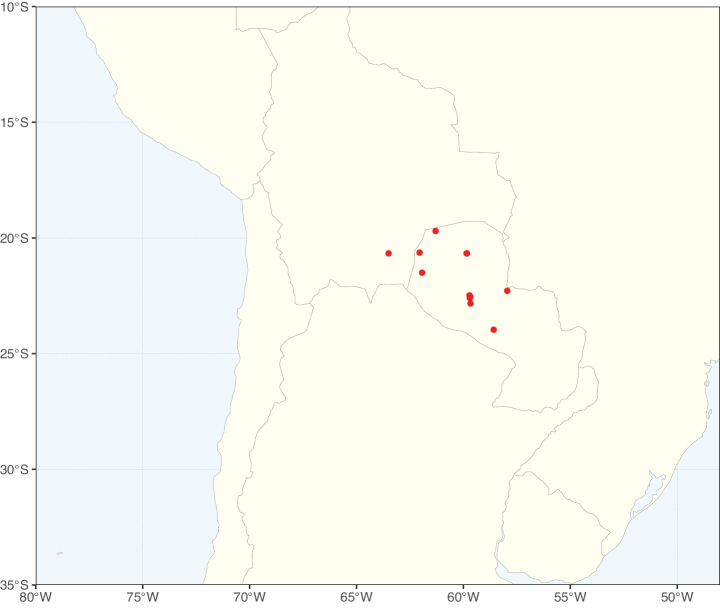
Distribution of *Prosopidastrum* based on quality-controlled digitised herbarium records. See Suppl. material 1 for the source of occurrence data.

Figs 144 , 145 , 146 , 155

#### Type.

*Prosopidastrummexicanum* (Dressler) Burkart [≡ Prosopisglobosavar.mexicana Dressler]

#### Description.

Small often aphyllous shrubs, 0.5–1.5 (3) m (Fig. 144I), usually armed with spinescent stipules, the shoots also often spinescent, tapered to a hard point, the whole shrub prickly; stems green, photosynthetic, striate with longitudinal, golden corky ridges (Fig. 145G); plants usually glabrous, sometimes sparsely pubescent with simple hairs. **Stipules** spinescent, straight or recurved, persistent or sometimes caducous, each bearing a rounded, adaxial nectary. **Leaves** bipinnate, small, to 2 cm long; nectaries cylindrical to cupulate, pitted, borne between the pinnae; pinnae 1 pair, opposite; leaflets 1–4 pairs per pinna, opposite, sessile, venation obscure except for central midvein. **Inflorescence** a capitulum, axillary, one head per axil (Fig. 145G); bracteoles subtending each flower peltate to lanceolate, hooded. **Flowers** 5-merous, sessile or pedicellate, functionally staminate or bisexual; sepals valvate in bud, connate, calyx campanulate, shallowly lobed; petals valvate in bud, free or basally adnate to the stamens forming a stemonozone, 1-veined, variously reflexed or incurved; stamens 10, fertile, filaments not flattened, white, anthers ovate, dorsifixed, pale yellow, equipped with a stipitate claviform apical gland; intrastaminal nectaries present; pollen in columellate tricolporate monads; ovary short stipitate to sessile, glabrous to pilose, stigma punctate with a deep cup. **Fruit** dorsiventrally flattened, linear-oblong loments (Fig. 146L) or craspedia, rarely dehiscent along the ventral suture, 3–10-seeded; valves coriaceous, exocarp light brown, reticulately veined, forming an umbo over each seed, endocarp smooth, fibrous, mesocarp absent, layers of fruits not separating from one another at maturity. **Seeds** not winged, obliquely inserted, testa hard, pleurogram present.

#### Chromosome number.

2*n* = 14, 28 (Luckow et al. 2005).

#### Included species and geographic distribution.

Six to eight species occupying a highly disjunct amphitropical distribution in Baja California and Isla Socorro, Mexico and central Argentina (Fig. 155).

#### Ecology.

In arid subtropical scrub and semi-desert vegetation. Often aphyllous. Fruits breaking up into one- or few-seeded segments.

#### Etymology.

From -*astrum* (Latin = partial resemblance to) and *Prosopis*, another mimosoid genus which it resembles.

#### Human uses.

Unknown.

#### Notes.

*Prosopidastrum* shares with *Piptadeniopsis* and *Mimozyganthus*, the two genera with which it is resolved in a robustly supported subclade of the Dichrostachys clade (Fig. 143; Luckow et al. 2005; Koenen et al. 2020a; Ringelberg et al. 2022), spinescent stipules and a mainly Argentina – Paraguay – Bolivia Chaco – Monte distribution in South America. *Prosopidastrum* is of particular biogeographic interest, being disjunct between Baja California and Argentina.

In the past, only 1–2 species were recognised until Palacios and Hoc (2001, 2005) worked on the Argentinian members when they recognised six species in Argentina alone. The main characters they used to separate species include sessile vs. pedicellate flowers, fruit dehiscent or indehiscent, and texture of the leaflets, as well as branching pattern and persistence of the stipules. A secondary character was inflorescence size.

It is difficult to reliably distinguish *P.angusticarpum* R.A. Palacios & Hoc from *P.striatum* (Benth.) R.A. Palacios & Hoc as the diameter of the inflorescence does not correlate with other characters, all of which overlap except petal colour and pubescence. The status of the segregates *P.gracile* R.A. Palacios & Hoc and *P.benthami* (Chodat & Wilczlek) R.A. Palacios & Hoc is also doubtful as they appear to differ from one another only in the colour of the petals (white vs. green-tipped). Thus, pending more detailed taxonomic revisionary work, there is uncertainty about whether six or eight species should be recognised in the genus.

#### Taxonomic references.

Palacios and Hoc (2001, 2005), with illustrations.

### ﻿ Piptadeniopsis

Taxon classificationPlantaeFabalesFabaceae

Burkart, Darwiniana 6: 478. 1944.

Fig. 156

#### Type.

*Piptadeniopsislomentifera* Burkart

#### Description.

Shrub or small branched treelet 3–7 m, shoots striate, green for first two years, terminal shoots often slender and somewhat pendulous, armed with 1–3 mm long stipular spines at nodes. **Leaves** bipinnate, small, borne on short axillary tuberculous brachyblasts or alternate on older growth; petiole 3–10 mm long, puberulent, terminating in a caducous spicule; sessile, circular nectaries at point of insertion of pinnae and 1–2 minute sessile nectaries at insertion of distal leaflet pairs; pinnae 1 pair, leaflets (2) 3 pairs, elliptic-obovate, obtuse and emarginate at apex, weakly asymmetric at base, glabrous or sparsely pubescent, 3–4-veined. **Inflorescence** capitate, solitary, axillary on 1 cm peduncles; bracteoles subtending each flower rhomboid, acuminate, incurved, caducous. **Flowers** uniformly hermaphrodite; sepals valvate, fused to form a cupular calyx, villous; petals valvate, almost free at anthesis, villous; stamens 10, free, anthers with a minute stipitate spherical gland apical on the connective; pollen in columellate 8–12 (16)-grained polyads, the individual grains with porate apertures; ovary stipitate, pubescent, stigma punctate, inconspicuous. **Fruits** stipitate, true loments, breaking up into 3–9 one-seeded articles with no persistent replum, linear, straight, compressed, margins raised, to 8 cm long × 0.8–1 cm wide, valves sub-coriaceous, glabrous. **Seeds** very compressed, ovate, 7 × 7 × 1 mm, wingless, testa thin, pleurogram absent.

#### Chromosome number.

2*n* = 28 (Luckow et al. 2005).

#### Included species and geographic distribution.

**Figure 156.**
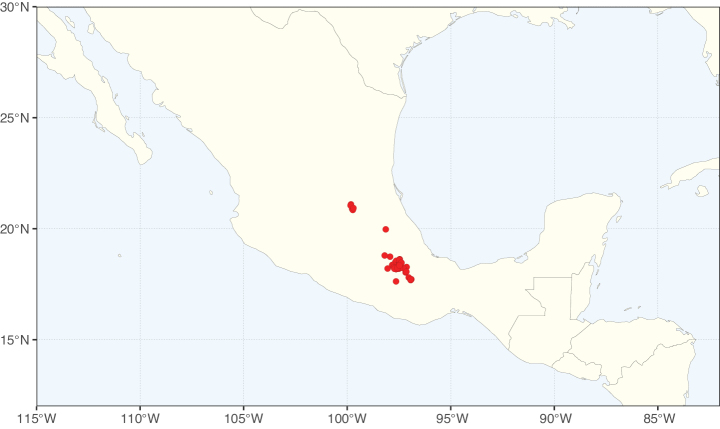
Distribution of *Piptadeniopsis* based on quality-controlled digitised herbarium records. See Suppl. material 1 for the source of occurrence data.

Monospecific (*P.lomentifera*), endemic to the Chaco domain in Paraguay and south-eastern Bolivia (Fig. 156).

#### Ecology.

Tropical and subtropical seasonally dry thorn forest and Chaco woodland. Usually deciduous.

#### Etymology.

From -*opsis* (Greek = appearance) and *Piptadenia*, another genus of mimosoid legume.

#### Human uses.

Unknown.

#### Notes.

*Piptadeniopsis* is robustly supported as sister to *Prosopidastrum* and these two genera together with *Mimozyganthus* form a subclade (Fig. 143; Luckow et al. 2005; Koenen et al. 2020a; Ringelberg et al. 2022). *Piptadeniopsis* and *Propidastrum* both have photosynthetic stems with longitudinal striations, whereas the stems of *Mimozyganthus* are terete. The two former genera also share lomentiform fruits while those of *Mimozyganthus* are plano-compressed, marginally winged and single-seeded. *Piptadeniopsis* has pollen in polyads whereas *Mimozyganthus* and *Prosopidastrum* have monads.

#### Taxonomic references.

Burkart (1944), including illustration; Luckow et al. (2005).

### ﻿ Calliandropsis

Taxon classificationPlantaeFabalesFabaceae

H.M. Hern. & P. Guinet, Kew Bull. 45(4): 609. 1990.

**Figure 157.**
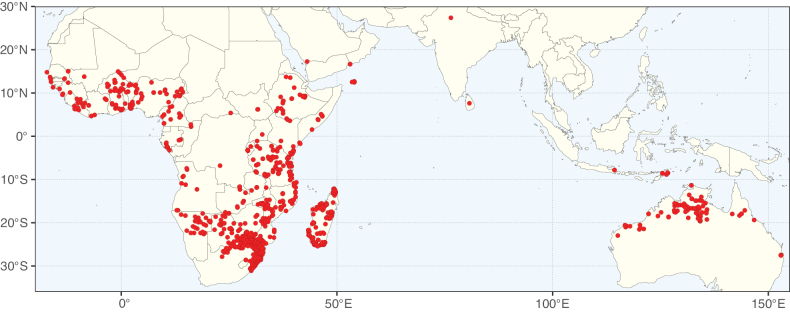
Distribution of *Calliandropsis* based on quality-controlled digitised herbarium records. See Suppl. material 1 for the source of occurrence data.

Figs 144 , 145 , 146 , 157

#### Type.

*Calliandropsisnervosa* (Britton & Rose) H.M. Hern. & P. Guinet [≡ *Anneslianervosa* Britton & Rose]

#### Description.

Small, unarmed shrubs to 1 m, profusely and intricately branched from the base (Fig. 144J); brachyblasts present, densely covered with persistent imbricate stipules, bearing leaves and inflorescences. **Stipules** leafy, triangular, to 2 mm. **Leaves** bipinnate; a cupular nectary at the point of insertion of the pinnae; pinnae 1 pair; leaflets (5) 6–9 (11) pairs per pinna, opposite, oblong, oblique at the base, acute at the apex, glabrous or sparsely villous with prominent primary and secondary veins abaxially. **Inflorescences** compact semi-spherical capitula, solitary in the axils (Fig. 145H), on a short peduncle, subtended by triangular bracts. **Flowers** usually all hermaphrodite but sometimes with a few functionally staminate flowers proximally; sepals valvate in bud, connate, calyx campanulate, 5-lobed; petals 5, valvate in bud, free or fused, lobes oblanceolate; stamens 5 per flower, all fertile, white or pale pink, anthers mostly eglandular, rarely with a minute sub-cylindrical apical gland; pollen in tricolporate monads; ovary sericeous, sessile, 5–6-ovulate, stigma tubular. **Fruits** dry, non-septate, straight, plano-compressed, oblanceolate, 1–5-seeded, the margins thickened, apex acute, valves rigidly coriaceous, dehiscing elastically from the apex to the base without twisting, the valves strongly recurving and persistent after dehiscence (Fig. 146N). **Seeds** ovoid, dark brown, pleurogram U-shaped.

#### Chromosome number.

Unknown.

#### Included species and geographic distribution.

Monospecific (*C.nervosa*), endemic to central Mexico from Durango south to Oaxaca (Fig. 157).

#### Ecology.

Seasonally dry thorn scrub on dry rocky calcareous soils, 1450–2000 m elevation. Seed dispersal mechanical via elastically dehiscent pods. Deciduous.

#### Etymology.

From Greek, -*opsis* (= appearance), referring to the resemblance to (the fruits of) the genus *Calliandra*.

#### Human uses.

Unknown.

#### Notes.

*Calliandropsis* is robustly supported as sister to the rest of the Dichrostachys subclade (Fig. 143), presenting a striking amphi-Atlantic disjunction between dry central Mexico and the Old World, mainly Madagascan, *Dichrostachys*, *Gagnebina* and *Alantsilodendron* clade. Fruiting specimens of *Calliandropsis* are often mis-identified as *Calliandraeriophylla* Benth., which co-occurs with *Calliandropsis* in south-central Mexico, because of the strong similarity of its elastically dehiscent fruits to those of *Calliandra*, but the flowers of *Calliandropsis*, with few free stamens, immediately distinguish it from *Calliandra* which has flowers with numerous stamens fused into a tube.

#### Taxonomic references.

Hernández and Guinet (1990), including an illustration.

### ﻿ Dichrostachys

Taxon classificationPlantaeFabalesFabaceae

Wight & Arn., Prod. Fl. Ind. Orient.: 271. 1834.

**Figure 158.**
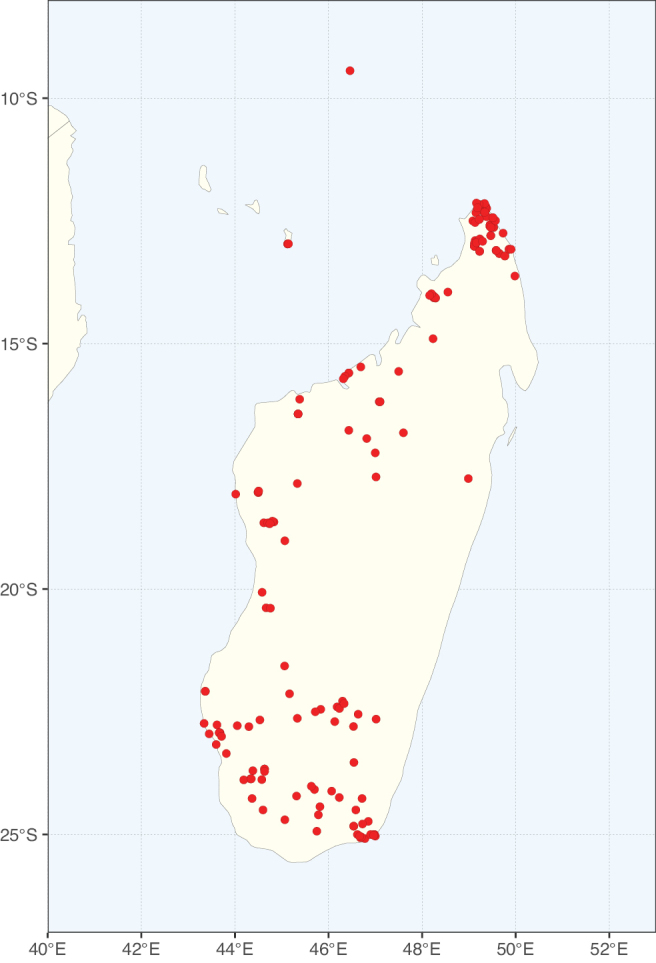
Distribution of *Dichrostachys* based on quality-controlled digitised herbarium records. See Suppl. material 1 for the source of occurrence data.

Figs 144 , 145 , 146 , 158

Cailliea Guill. & Perr., Fl. Seneg. Tent.: 239. 1832, nom. rej. vs. Dichrostachys Wight & Arn. Type: Caillieadichrostachys Guill. & Perr., nom. illeg. [≡ Mimosanutans Pers. (= Dichrostachyscinereasubsp.africana Brenan & Brummitt)]

#### Type.

*Dichrostachyscinerea* (L.) Wight & Arnott [≡ *Mimosacinerea* L.]

#### Description.

Shrubs or small trees to 7 m (Fig. 144K), branching from base, unarmed or the branches modified into thorns, branches plagiotropic, young stems terete, rarely angled with corky ridges; older stems terete, brachyblasts present, clothed in distichous fused stipule bases. **Stipules** monomorphic, ovate-triangular, striate, stramineous, persistent. **Leaves** bipinnate; extrafloral nectaries usually between the proximal pair of pinnae but sometimes at mid-petiole and sometimes additional glands between more distal pinnae, usually cylindrical, raised; pinnae 1–15 (20) pairs; leaflets 1–40 pairs per pinna, linear to ovate, venation brochidodromous to fused eucamptodromous, obscure to abaxially raised, pubescent to glabrous. **Inflorescences** pedunculate condensed spikes (Fig. 145K, L), the peduncle often bearing a few lanceolate bracts, 1–3 in leaf axils of new growth or more frequently on the brachyblasts, not forming paniculiform secondary inflorescences; bracteoles subtending each flower carinate, 1-nerved; spikes composed of sterile flowers proximally, rarely sterile flowers absent (D.kirkiif.puccioniana), fertile flowers distally, and often a few functionally staminate flowers in between. **Flowers** sessile, pseudopedicels absent; sepals and petals valvate in bud; sterile flowers smaller than the hermaphrodite ones with 10, showy, filamentous or flattened and ribbon-like staminodia, bright rose-pink fading white; functionally staminate flowers similar to bisexual ones but either lacking or having only a rudimentary ovary; hermaphroditic flowers with a 5-lobed calyx, petals 5, free or sometimes centrally or basally connate, usually linear or lanceolate, 1-nerved; stamens 10, the filaments either exserted or included at anthesis, anthers with a spherical, long-stipitate, apical gland or gland absent (Madagascar); pollen in 16-grained, acalymmate polyads shed as single grains or calymmate polyads of 8–16 grains, exine verrucate to reticulate; ovary oblong to ovate, sessile, densely strigose with silky white hairs, style exserted at anthesis, white, stigma punctate. **Fruits** sessile, dorsiventrally compressed, elastically dehiscent from the apex (Fig. 146P) and coiling after dehiscence, or indehiscent (Fig. 146O); pericarp coriaceous in elastically dehiscent species, woody in the indehiscent ones, the sutural ribs well defined but not greatly enlarged and overtopping the valves, the interior of the fruit usually invaginated between the seeds. **Seeds** obliquely oriented, ovate to rhomboidal, pleurogram either U-shaped or forming nearly a complete oval on the face of the seed.

#### Chromosome number.

2*n* = 28 (36, 54, 56, 78) in *D.cinerea* (Atchison 1951) which forms a poorly understood polyploid series (Brenan and Brummitt 1965).

#### Included species and geographic distribution.

ca. 13–14 species, *D.cinerea* with many varieties. One species native and widespread in Africa, India, and Australia, and widely introduced and weedy elsewhere; one species restricted to the Horn of Africa; one endemic to Socotra; one endemic to Australia; 10 species in Madagascar (Fig. 158).

#### Ecology.

Many diverse habitats, from open savannas and seasonally dry forests to arid tropical scrub. Mostly deciduous. *Dichrostachyscinerea* is a problematic weed in many parts of the tropics, where it can form pure stands and outcompete native vegetation.

#### Etymology.

From Greek, *di*- (= two), *chroma* (= colour) and -*stachys* (= spike), in reference to the bi-coloured spicate inflorescence found in the genus (Fig. 145K, L).

#### Human uses.

Wood used for fuel and for small tools, leaves and fruits are important sources of livestock forage. *Dichrostachyscinerea* has been used in soil reclamation projects and as an ornamental (Pedroso and Kaltschmitt 2012).

#### Notes.

As currently circumscribed, *Dichrostachys* is non-monophyletic (Fig. 143; Hughes et al. 2003; Luckow et al. 2005; Ringelberg et al. 2022). This non-monophyly will be dealt with via erection of a new genus, *Famoha* ined. (Luckow in prep.) in a forthcoming monograph that includes a species-level taxonomic account of the four closely related genera: *Dichrostachys*, *Gagnebina*, *Alantislodendron*, and the upcoming new genus.

#### Taxonomic references.

Brenan and Brummitt (1965); Thulin (1989).

### ﻿ Gagnebina

Taxon classificationPlantaeFabalesFabaceae

Neck. ex DC., Prodr. [A.P. de Candolle] 2: 431. 1825.

**Figure 159.**
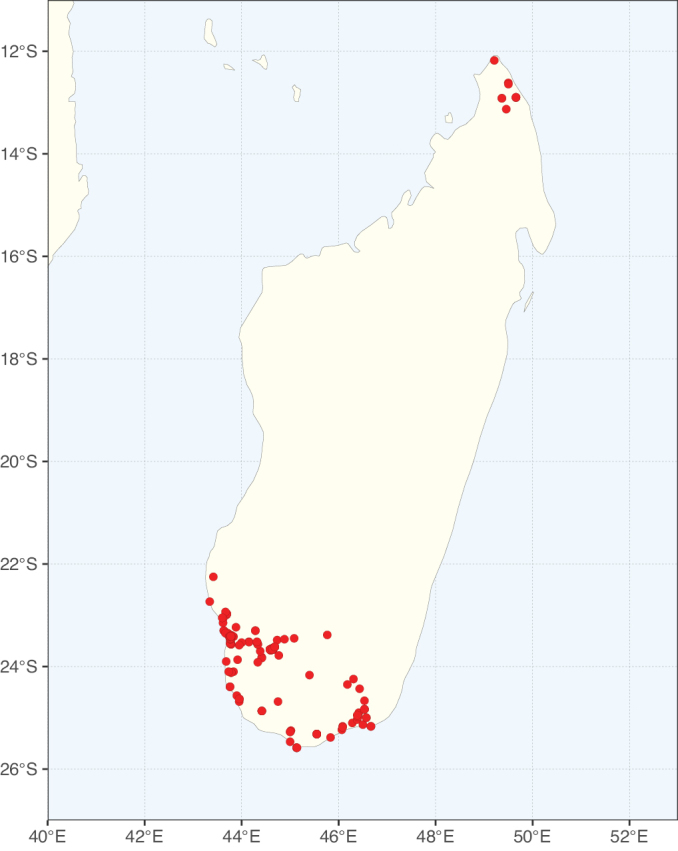
Distribution of *Gagnebina* based on quality-controlled digitised herbarium records. See Suppl. material 1 for the source of occurrence data.

Figs 144 , 145 , 146 , 159

#### Type.

*Gagnebinatamarascina* (Lam.) DC. [≡ *Mimosatamariscina* Lam. (= *Gagnebinapterocarpa* (Lam.) Baill.)]

#### Description.

Unarmed shrubs or small trees, branched from the base, 1–6 (10) m (Fig. 144M); brachyblasts absent, perulate resting buds present, the scales striate with raised nerves. **Stipules** dimorphic, those on leaves formed just after the resting bud breaks striate, stramineous, similar in colour and texture to the resting bud scales, caducous, becoming subulate or setose distally on shoots, the stipule type most commonly seen on flowering and fruiting branches. **Leaves** bipinnate, nectaries present between the lower pair of pinnae or mid-petiole, sometimes between additional pinnae; pinnae (3) 5–40 pairs per leaf, opposite or sub-opposite, leaflets numerous, linear to oblong with truncate bases, pinnately veined, usually glabrous or ciliate. **Inflorescence** usually a condensed spike (a true spike in one species, Fig. 145J), pedunculate, the peduncle often bearing a few lanceolate bracts, 1–8 in the leaf axils, the leaves sometimes suppressed and forming a paniculiform secondary inflorescence, this immersed in (or rarely exserted above) the foliage, calyx often constricted into a pseudopedicel and inflorescence appearing racemose; bracteoles subtending each flower carinate, 1-nerved; inflorescences composed of sterile flowers proximally, fertile flowers distally, and often a few functionally staminate flowers in between. **Flowers** with sepals and petals valvate in bud; sterile flowers with a 5-lobed calyx, fused ½–¾ its length, lobes with a central raised nerve; petals 5, free or connate, 1-nerved, staminodia 10, showy, filamentous or flattened and ribbon-like, white or rarely rose-coloured; functionally staminate flowers similar to the fertile ones but either lacking or having only a rudimentary ovary; hermaphrodite flowers similar to the sterile ones but larger, with a 5-lobed calyx and 5 free (rarely connate), usually linear or lanceolate, 1-nerved petals; stamens 10, ovate to linear, either with a terminal apiculum or lacking a gland, sagittate at the base; pollen of acalymmate 8-grained porate polyads, exine verrucate; ovary ovate, stipitate or sessile, densely strigose apically with silky white hairs, stigma punctate. **Fruits** either sessile or stipitate, compressed, either elastically dehiscent from the apex and recurved but not coiled after dehiscence, or indehiscent or tardily inertly dehiscent; pericarp woody in elastically dehiscent species, coriaceous to chartaceous in the indehiscent ones, the sutural ribs often greatly thickened or modified into flattened wings (Fig. 146K), the interior of the fruit usually invaginated between the seeds. **Seeds** obliquely or laterally positioned, ovate to rhomboidal, with the pleurogram either U-shaped or forming nearly a complete oval on the face of the seed.

#### Chromosome number.

2*n* = 26 (Goldblatt 1981b).

#### Included species and geographic distribution.

Seven species. Madagascar, Mauritius, the Mascarenes and Comoros Islands, Aldabra (Fig. 159).

#### Ecology.

Usually along rivers in evergreen or deciduous woodlands; one species [*G.commersoniana* (Baill.) R. Vig.] weedy and found in a wide array of habitats. The winged pods of *G.pterocarpa* have allowed it to colonise islands in the Indian Ocean; *G.commersonia* also has lightweight pods and in addition to being a widespread weed in Madagascar, it is found on Aldabra.

#### Etymology.

Named in honour of Françoise Gagnepain, French botanist (1866–1952).

#### Human uses.

Wood used locally for building houses, reportedly used in making Maintirano paper (Bosch et al. 2011).

#### Notes.

Closely related to *Dichrostachys* and *Alantsilodendron* (Fig. 143) and differing from these genera in having resting buds with perulate scales instead of brachyblasts, as well as having dimorphic stipules.

#### Taxonomic references.

Lewis and Guinet (1986); Luckow (1995); Luckow and DuPuy (2000), all with illustrations.

### ﻿ Alantsilodendron

Taxon classificationPlantaeFabalesFabaceae

Villiers, Bull. Mus. Natl. Hist. Nat., B, Adansonia 16: 65. 1994.

**Figure 160.**
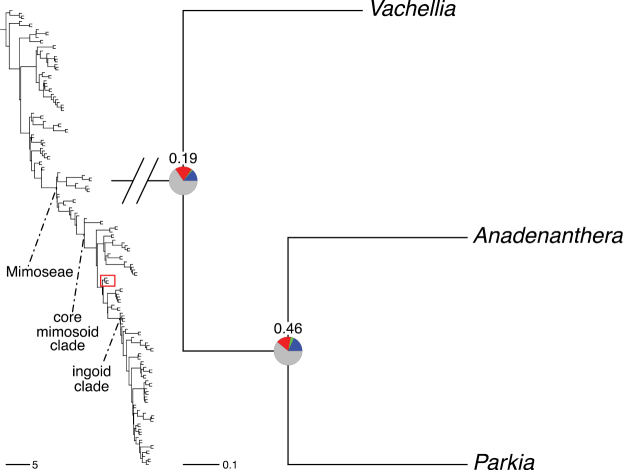
Distribution of *Alantsilodendron* based on quality-controlled digitised herbarium records. See Suppl. material 1 for the source of occurrence data.

Figs 144 , 145 , 146 , 160

#### Type.

*Alantsilodendronvillosum* (R. Vig.) Villiers [≡ *Dichrostachysvillosa* R. Vig.]

#### Description.

Unarmed (weak thorns in one species) shrubs or small treelets (Fig. 144L), 1–5 m, much-branched from base, branches often plagiotropic; brachyblasts present, usually clothed in distichous or spirally arranged rows of persistent or caducous stipule bases. **Stipules** monomorphic, striate, ovate. **Leaves** bipinnate, extrafloral nectary between lower pair, and sometimes more distal pinnae; pinnae 1–7 pairs (10–50 in one species); leaflets 2–20 pairs per pinna (30–50 in one species), linear to ovate, base truncate. **Inflorescence** usually a capitulum (Fig. 145I), occasionally a condensed spike, usually solitary in leaf axils of new growth or more often on short shoots, flowers usually lacking a pseudopedicel; bracteoles subtending individual flowers carinate, 1-nerved; inflorescence composed usually entirely of hermaphrodite flowers but some species with sterile flowers proximally, fertile flowers distally, and often a few functionally staminate flowers in between. **Flowers** with sepals and petals valvate in bud; sterile flowers with a 5-lobed or entire calyx, fused ½–¾ its length, each lobe with a central raised nerve; petals 5, connate, each petal 1–3 nerved, staminodia 10, filamentous, white; functionally staminate flowers similar to fertile ones but either lacking an ovary or having only a rudimentary one; bisexual flowers similar to sterile ones but larger, with a 5-lobed calyx and 5 connate petals (free in one species), usually linear or lanceolate, 1–3 nerved; stamens 8–10, filaments exserted at anthesis, anthers either with a terminal apiculum or lacking an apical gland; pollen of 8- or 16-grained calymmate or acalymmate polyads, grains shed as tetrads if acalymmate, exine verrucate or psilate; ovary ovate, sessile, densely pilose or pubescent, style exserted (rarely included) at anthesis, stigma porate to broad funnelform. **Fruits** sessile, terete when immature, dorsiventrally compressed at maturity, either inertly dehiscent or more often elastically dehiscent from the apex, the valves recurved but not coiling after dehiscence (Fig. 146Q), pericarp woody in elastically dehiscent species, coriaceous in inertly dehiscent ones, sutural ribs often greatly thickened, interior of pod usually invaginated between seeds. **Seeds** obliquely or laterally positioned in the fruit, ovate to rhomboidal, pleurogram either U-shaped or forming nearly a complete oval.

#### Chromosome number.

Unknown.

#### Included species and geographic distribution.

Eleven species, in southern, western, and far northern Madagascar (Fig. 160).

#### Ecology.

Restricted to seasonal xerophytic deciduous woodlands and scrub, especially in the spiny forests of south-western Madagascar, deciduous in long dry season.

#### Etymology.

From *alantsili*-, the Madagascan name for dry forest, and -*dendron* (Greek = tree) in reference to the distribution of the genus restricted to dry forest.

#### Human uses.

Unknown.

#### Notes.

*Alantsilodendron* is closely related to *Dichrostachys* and *Gagnebina* (Fig. 143) but differs in having fused petals, and typically a capitate inflorescence lacking staminodial flowers. The current non-monophyly of *Alantsilodendron* (Fig. 143) will be dealt with in a forthcoming monograph (see *Dichrostachys* and Dichrostachys clade notes for more details).

#### Taxonomic references.

Villiers (1994, 2002), both including illustrations.

## ﻿﻿22. Parkia clade

Helen C. F. Hopkins^10^, David S. Seigler^38^, John E. Ebinger^14^, Vanessa Terra^41^

Citation: Hopkins HCF, Seigler DS, Ebinger JE, Terra V (2024) 22. Parkia clade. In: Bruneau A, Queiroz LP, Ringelberg JJ (Eds) Advances in Legume Systematics 14. Classification of Caesalpinioideae. Part 2: Higher-level classification. PhytoKeys 240: 299–315. https://doi.org/10.3897/phytokeys.240.101716

**Parkia clade**

Figs 161–167

**Included genera (3).***Anadenanthera* Speg. (2–ca. 4 species), *Parkia* R. Br. (ca. 35), *Vachellia* Wight & Arn. (164).

**Description.** Trees or shrubs. **Stipules** small and caducous (*Anadenanthera*, *Parkia*) or persistent and spinose (*Vachellia*). **Leaves** bipinnate, usually alternate (some exceptions in *Parkia*); extrafloral nectaries present on the petiole, and commonly also distally on the rachis (*Vachellia*, *Parkia*); leaflets opposite (rarely alternate in *Parkia*), oblong, narrowly oblong, cultrate, elliptic, linear or rarely otherwise, main-vein typically centric and parallel to the margins in oblong and narrowly oblong leaflets, petiolule or point of attachment typically centric or subcentric (a few exceptions). **Inflorescences** globose (all 3 genera), or clavate, oblate, subglobose or biglobose (*Parkia* p.p.), or spicate or oblongoid (*Vachellia* p.p.). **Flowers** homomorphic (all 3 genera) or heteromorphic (most species of *Parkia*); perianth 5-merous; stamens 10 (*Anadenanthera*, *Parkia*) or many (*Vachellia*), anther glands present or absent; pollen in polyads; styles elongate-filiform, stigmas cup-shaped to poriform. **Fruits** dehiscent or not, containing pulp, or gum, or neither. **Seeds** in 1 or occasionally in 2 series, with a pleurogram (possible exceptions in *Parkia*).

**Geographic distribution.** Pantropical; *Vachellia* and *Parkia* are both pantropical whereas *Anadenanthera* is confined to the Americas.

**Figure 161.**
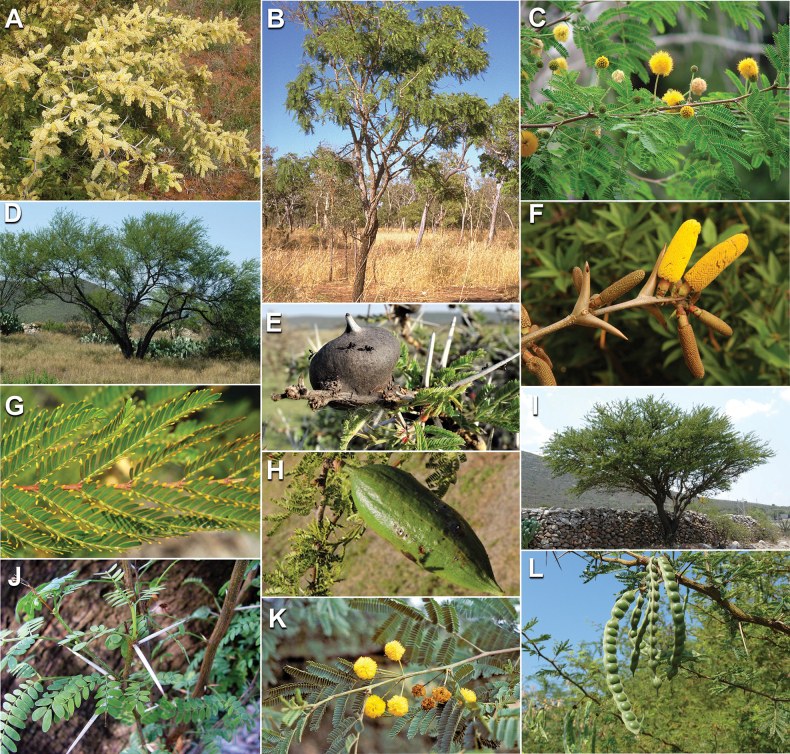
Generic relationships in the Parkia clade (tribe Mimoseae). For description of phylogeny and support values, see Fig. 6 caption (page 63).

**Notes.** The Parkia clade sensu Koenen et al. (2020a) comprises *Anadenanthera*, *Parkia* and *Vachellia* where *Anadenanthera* and *Parkia* are sister taxa and *Vachellia* is sister to both (Fig. 161). The relationship between *Parkia* and *Anadenanthera* had already been established, for instance by Simon et al. (2016) and Ribeiro et al. (2018), but these three disparate genera have never been considered closely related in classifications based on morphology. The clade is heterogeneous morphologically and ecologically, and we have found no characters that easily define it. This is reflected in the long branches that characterise each of the three genera, particularly *Parkia* and *Vachellia* (Koenen et al. 2020a; Ringelberg et al. 2022). Most features that the three genera share are common to others in closely related clades, such as the type of extrafloral nectaries (parenchymatic, subcategory elevated; Marazzi et al. 2019) and anther glands (Piptadenia type and subtype; Barros and Teixeira 2016).

**Clade-based definition.** The most inclusive crown clade containing *Vachelliatortilis* (Forssk.) Galasso & Banfi and *Parkiabicolor* A. Chev., but not *Dichrostachysunijuga* Baker, *Lachesiodendronviridiflorum* (Kunth) P.G. Ribeiro, L.P. Queiroz & Luckow, or *Stryphnodendronadstringens* (Mart.) Coville (Fig. 161).

Many characters show a patchwork distribution within the clade. These include: stipular spines (present in *Vachellia*, sometimes enlarged in Africa and the Americas and inhabited by ants; absent in *Anadenanthera* and *Parkia*); brachyblasts (present in *Vachellia*; absent in *Anadenanthera* and *Parkia*); involucre of bracts on the peduncle (present in *Vachellia* and *Anadenanthera*, absent in *Parkia*); calyx lobes (equal and valvate in *Vachellia* and *Anadenanthera*; unequal and imbricate in *Parkia*); stamen number (10 in *Parkia* and *Anadenanthera*, 20–100 in *Vachellia*); stemonozone (absent in *Vachellia*, present in *Anadenanthera* and often in *Parkia*); and pollen grains (colporate in *Vachellia*, porate in *Parkia* and *Anadenanthera*). Evidence of polyploidy is seen in *Vachellia* but not the other two genera. Root nodulation is apparently common in *Vachellia* and *Anadenanthera* and absent in *Parkia* (Faria et al. 2022). In terms of habitat, *Parkia* is primarily found in tropical rainforests whereas *Vachellia* generally prefers drier environments, including seasonally dry forest and scrub, as does *Anadenanthera*. Taken together, these characters reflect the closer relationship between *Anadenanthera* and *Parkia*, and confirm the greater distinctiveness of *Vachellia*.

Most, though not all, species of *Parkia* are bat-pollinated and many of their unusual morphological characters can be related to chiropterophily (see *Parkia* Notes). In contrast, the smaller inflorescences in *Anadenanthera* and *Vachellia* commonly exhibit characters typical of melittophily or generalised pollination by diurnal insects (short, slender peduncles, fragrant floral odours, diurnal anthesis).

### ﻿ Vachellia

Taxon classificationPlantaeFabalesFabaceae

Wight & Arn., Prodr. Fl. Ind. Orient. 1: 272. 1834.

Figs 162 , 163

Aldina E. Mey., Comment. Pl. Africae Austr. 171. 1836, nom. illeg., non Aldina Adans. (1763) nom. rej., nec Aldina Endl. 1840. nom. cons. Type not designated.
Farnesia Gasp., Deser. Nuov. Gen. 1838, non Farnesia Heist. ex Fabr., Enum. Pl. Hort. Helmstad. ed. 2, 400. 1763. Type: Farnesiaodora Gasp., nom. illeg. [= Mimosafarnesiana L. (≡ Vachelliafarnesiana (L.) Wight & Arn.)]
Gumifera Raf., Sylva Tellur.: 118. 1838. Lectotype: Gumiferanilotica (L.) Raf. [≡ Mimosanilotica L. (≡ Vachellianilotica (L.) P.J.H. Hurter & Mabb.)]
Poponax Raf., Sylva Tellur.: 118. 1838. Type: Poponaxtortuosa (L.) Raf. [≡ Mimosatortuosa L. (≡ Vachelliatortuosa (L.) Seigler & Ebinger)]
Delaportea Thorel ex Gagnep., Notul. Syst. (Paris) 2: 118. 1911. Type: Delaporteaarmata Thorel ex Gagnep. [≡ Pithecellobiumharmandianum Pierre (≡ Vachelliaharmandiana (Pierre) Maslin, Seigler & Ebinger)]
Pithecodendron Speg., Physis (Buenos Aires) 6: 313. 1923. Type: Pithecodendronargentinense Speg. [= Mimosahorrida L. (≡ Vachelliahorrida (L.) Kyal. & Boatwr.)]
Nimiria Prain ex Craib, Bull. Misc. Inform. Kew 1927: 393. 1927. Type: Nimiriasiamensis Craib [≡ Vachelliasiamensis (Craib) Maslin, Seigler & Ebinger]
Acaciopsis Britton & Rose, N. Amer. Fl. 23: 93. 1928. Type: Acaciopsispringlei (Rose) Britton & Rose [≡ Acaciapringlei Rose (≡ Vachelliapringlei (Rose) Seigler & Ebinger)]
Bahamia Britton & Rose, N. Amer. Fl. 23: 86. 1928. Type: Bahamiaacuifera (Benth.) Britton & Rose [≡ Acaciaacuifera Benth. (≡ Vachelliaacuifera (Benth.) Seigler & Ebinger)]
Feracacia Britton & Rose, N. Amer. Fl. 23: 86. 1928. Type: Feracaciadaemon (Ekman & Urb.) Britton & Léon [≡ Acaciadaemon Ekman & Urb. (≡ Vachelliadaemon (Ekman & Urb.) Seigler & Ebinger)]
Fishlockia Britton & Rose, N. Amer. Fl. 23: 91. 1928. Type: Fishlockiaanegadensis (Britton) Britton & Rose [≡ Acaciaanegadensis Britton (≡ Vachelliaanegadensis (Britton) Seigler & Ebinger)]
Lucaya Britton & Rose, N. Amer. Fl. 23: 87. 1928. Type: Lucayachoriophylla (Benth.) Britton & Rose [≡ Acaciachoriophylla Benth. (≡ Vachelliachoriophylla (Benth.) Seigler & Ebinger)]
Myrmecodendron Britton & Rose, N. Amer. Fl. 23: 91. 1928. Type: Myrmecodendronhindsii (Rose) Britton & Rose [≡ Acaciahindsii Benth. (≡ Vachelliahindsii (Benth.) Seigler & Ebinger)]
Tauroceras Britton & Rose, N. Amer. Fl. 23: 85. 1928. Type: Taurocerasspadicigerum (Schltdl. & Cham.) Britton & Rose [≡ Acaciaspadicigera Schltdl. & Cham. (= Vachelliacornigera (L.) Seigler & Ebinger)]
Acacia Mill. subg. Acacia sensu Vassal, Bull. Soc. Hist. Nat. Toulouse 108: 139. 1972.

#### Lectotype

**(designated by Ross 1975b: 465, 471).***Vachelliafarnesiana* (L.) Wight & Arn. [≡ *Mimosafarnesiana* L.] [Linnaean Plant Name Typification Project (2022); illustration at S, IDC 214.5]

#### Description.

**Figure 162.**
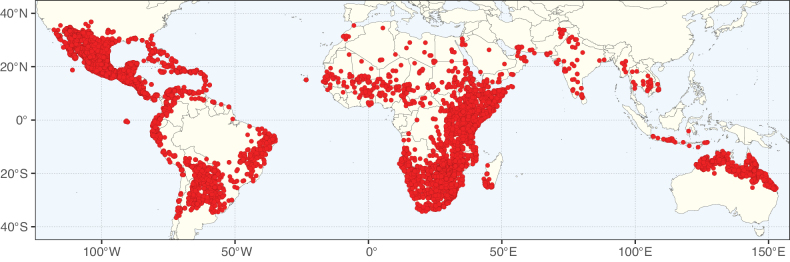
Morphological features of *Vachellia***A** branches with spicate inflorescences of *V.rigidula* (Benth.) Seigler & Ebinger **B** habit of *V.bidwillii* (Benth.) Kodela (*M. Simmons 3191*) **C** inflorescences and leaves of *V.farnesiana* (L.) Wight & Arn. **D** habit of *V.farnesiana***E** gall and spines of *V.drepanolobium* (Harms ex Y. Sjöstedt) P.J.H. Hurter **F** spines and inflorescences of *V.cornigera* (L.) Seigler & Ebinger (*Seigler 16051*) **G** partial leaf of *V.cornigera* with Beltian Bodies (Tamaulipas, Mexico) (*Seigler 15962*) **H** fruit of *V.caven* (Molina) Seigler & Ebinger **I** habit of *V.schaffneri* (S. Watson) Seigler & Ebinger **J** leaves and stems of *V.karroo* (Hayne) Banfi & Galasso **K** inflorescences and leaves of V.niloticasubsp.indica (Benth.) Kyal. & Boatwr. (*Simmons 1032*) **L** fruits of V.niloticasubsp.kraussiana (Benth.) Kyal. & Boatwr. Photo credits **A** D Seigler **B, K** J Simmons **C, J** S Navie **D, F, G, I** B Maslin **E** T Nicholls, Nature Education Center **H** RT Queiroz http://rubens-plantasdobrasil.blogspot.com/**L** D Kunjithapatham.

Shrubs or trees, 0.5–30 m, rarely a prostrate shrub; bark mostly dark to light brown to black or grey, rarely whitish-reddish to yellowish, and papery, peeling or corky, with most species commonly rough to furrowed; brachyblasts usually present; prickles absent. **Stipules** spinescent, woody, paired at the nodes, straight to curved, sometimes asymmetrical, in some species enlarged and inhabited by ants, occasionally exceeding 200 mm long; a number of African species with galls subtending the spines. **Leaves** caducous (sometimes evergreen), alternate and also commonly clustered (2–8) on short shoots, these brachyblast leaves usually smaller and with fewer pinna pairs and leaflets than the alternately arranged leaves on fast growing branches; petioles with one or more extrafloral nectaries, often with one to several on the rachis, most commonly between the pinna pairs; pinnae 1–many (60+) pairs, mostly opposite; leaflets 1-many (70+) pairs per pinna, mostly opposite, sessile to subsessile, the apex in most American myrmecophytes bearing a small yellow detachable Beltian body. **Inflorescences** capitula to cylindrical spikes, solitary or clustered in leaf axils or on short shoots, rarely in pseudo-racemes or pseudo-panicles, sometimes andromonoecious; peduncles with a small involucel, usually medial or basal, rarely at the base of the inflorescence. **Flowers** bracteate, sessile to subsessile, actinomorphic; calyx 4–5 (6)-lobed; corolla 4–5 (6)-lobed; stamens 20–100, yellow to gold or creamy white, filaments usually separate to the base or occasionally shortly fused (or rarely and irregularly a greater degree of fusion), exserted, anthers small, dorsifixed, apical glands often present; pollen in polyads of 16 (8, 12, 24, 32, 48) grains, colporate with the exine surface psilate and nexine with columellae (Guinet and Vassal 1978; Guinet 1981b; Caccavari and Dome 2000; Maslin et al. 2003; Miller and Bayer 2003; Duarte et al. 2021); nectary disc absent; ovary sessile to subsessile. **Fruits** mostly dehiscing along both sutures, occasionally indehiscent, linear to oblong, straight to falcate, flattened to terete, a pericarpic strip (papyraceous mesocarp) sometimes present inside each valve in some species. **Seeds** uniseriate or biseriate to irregularly arranged, (4) 6–12 (14) per fruit if uniseriate, to 22 (40) if biseriate, sometimes surrounded by pulp, ovoid to ellipsoid, often flattened, testa hard, pleurogram large to small; funicle filiform, not enlarged arillate (Fig. 162).

#### Chromosome number.

*x* = 13. Many American species of *Vachellia* are diploids with 2*n* = 26 (Atchison 1948; Darlington and Wylie 1956; Turner 1959; Turner and Fearing 1960a; Hamant et al. 1975; Goldblatt and Johnson 1979–; Rico-Arce 2007; Rice et al. 2015). A smaller number of African species are diploids and several are tetraploid, whereas others have higher ploidy levels (Ross 1979). Among Asian species, a number in India have 2*n* = 26, 52 and/or 78 (Chakrabarty and Gandopadhyay 1996; Gómez-Acevedo and Tapia-Pastrana 2003).

#### Included species and geographic distribution.

**Figure 163.**
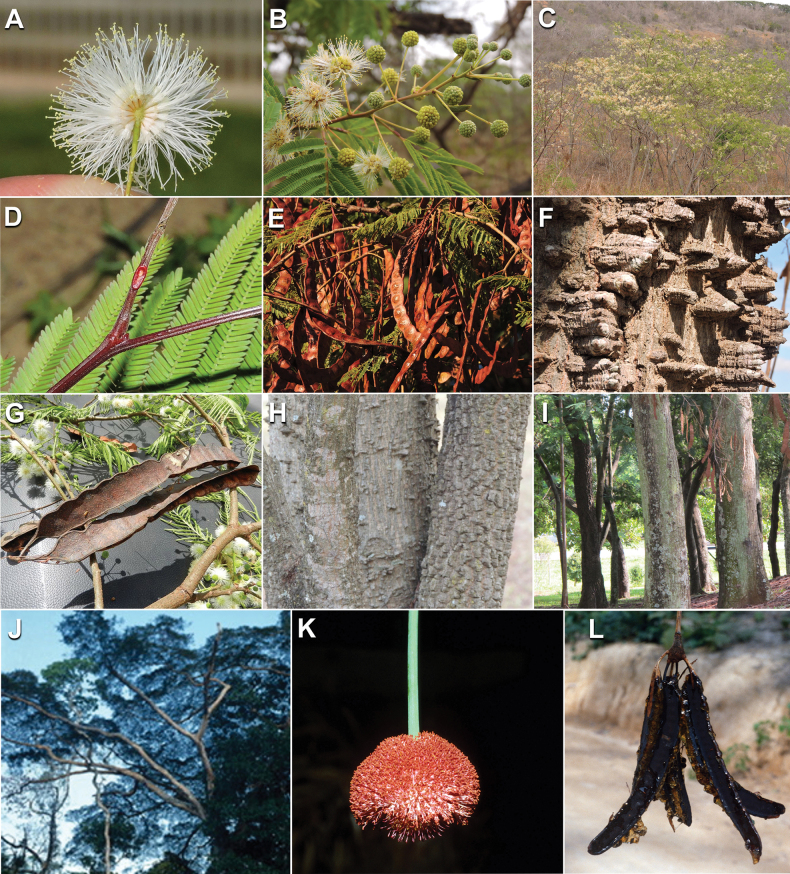
Distribution of *Vachellia* based on quality-controlled digitised herbarium records. The map includes records for introduced/naturalised taxa as well as native ones. For instance, most records in eastern Brazil are due to *V.farnesiana*, which is naturalised there. The Indian subcontinent may be under-sampled (see Deshpande et al. 2019). See Suppl. material 1 for the source of occurrence data.

*Vachellia* (164 species in total) is represented by 61 species (some with forms and varieties) in the Americas, 75 in Africa and Madagascar, nine in Australia and the Pacific, and 33 in Asia (including about 15 also found in Africa) (Maslin et al. 2003; Maslin 2015; WorldWideWattle 2022). A strong geographical element (America vs Africa + Asia + Australia) is evident in the relationships amongst *Vachellia* species worldwide (Miller and Seigler 2012; Kyalangalilwa et al. 2013; Boatwright et al. 2015; Comben et al. 2020) (Fig. 163).

*Vachellia* is widespread in most tropical and many subtropical areas of the world. A few species enter warm temperate regions but their distribution is generally limited to areas that lack killing frosts. *Vachellianilotica* and *V.farnesiana* have been widely introduced into the Old and New World, respectively. The latter has a pantropical distribution and, including probable introductions, is the most widely distributed of all members of the genus.

#### Ecology.

Many members of *Vachellia* are tenacious, spiny and invasive. Although most species favour disturbed, arid sites from sea level to ca. 2800 m, others (e.g., *V.mayana* (Lundell) Seigler & Ebinger) are limited to relatively undisturbed rainforests. Species are known from many forest types including primary and secondary tropical evergreen forests, rainforests, semi-deciduous forests, dry and wet secondary forests, montane, gallery, riverine forests, and are frequently found in *Quercus-Juniperus* woodlands, but uncommonly in secondary vegetation of cloud forests and ground water forests in Africa (Ross 1979). *Vachellia* species are also frequent in dry, deciduous scrub vegetation, xerophytic scrub, river valley scrub, thornveld, bushveld, semi-desert scrub, caatinga, chaco scrub, bushlands, grasslands, savanna grasslands, dwarf-tree grasslands, and wooded grasslands.

They are associated with many soil types including sands, clay, serpentine soils, volcanic soils, limestone and rocky limestone soils, karst limestone, coastal dunes, granitic washes, loam, saline soils, heavy black soils, gravelly soils, Kalahari sands, seasonally flooded alluvium, black cotton soils, hard-pan grey soils, dry water courses, margins of seasonal swamps, and they sometimes grow on termite mounds (Ross 1979).

#### Etymology.

*Vachellia* was named in honour of the Reverend George Harvey Vachell (1799–1839), a collector of the flora of China.

#### Human uses.

*Vachellia* species are often used for firewood and for making charcoal and are the major source of fuel in many areas of the world. They often serve as forage for livestock especially in times of drought (Cheatham et al. 1995; Chakrabarty and Gandopadhyay 1996; Smit 1999; Rico-Arce 2007). The wood has been used for construction of houses, for making furniture, tool handles, digging sticks, ploughs, cart wheels, fence posts, bows and arrow shafts and various objects. Only a few species such as *V.leucophloea* (Roxb.) Maslin, Seigler & Ebinger are important for lumber. The inner bark of this species and others is used for making twine and fiber for fishnets and cordage (Chakrabarty and Gandopadhyay 1996). Tannins from bark and pods of *Vachellia* species have been used to tan leather and extracts of the bark have also been used to prepare permanent black ink (Smit 1999).

A number of especially spiny species are grown for living fences. In Africa these are used to surround kraals. Others, such as *Vachellialeucophloea* and *V.nilotica*, are planted along roadsides and in gardens. Some are grown as shade trees for coffee, cacao and other crops (Chakrabarty and Gandopadhyay 1996). In the Americas species such as *V.constricta* (Benth.) Seigler & Ebinger are used as ground covers and ornamentals in landscaping (Cheatham et al. 1995).

*Vachelliafarnesiana* and *V.caven* (Molina) Seigler & Ebinger were early introduced into Spain, France and Italy (Bell et al. 2017). The fragrances from the flowers are commonly used in perfumes. Egypt is presently the most important producing country. Although the foliage, fruits and seeds of many species are toxic to humans as well as to other animals, they often serve as fodder for livestock and in some instances are eaten by humans. Others are used as components of traditional medicine (Cheatham et al. 1995).

#### Notes.

The genus *Vachellia* was once part of a widely circumscribed *Acacia* Mill., and as first described by Miller (1754), *Acacia* had a type that now belongs to *Vachellia*. Many of the species later placed in *Acacia* were included in a larger genus *Mimosa* but Bentham (1841a, 1842a, b) narrowed the concept of *Acacia* and later he restricted his tribe Acacieae and divided the genus *Acacia* into six series (Bentham 1875). Vassal (1972), in turn, described three subgenera, viz *Acacia*, *Aculeiferum*, and Heterophyllum (Phyllodineae), from these series. Pedley (1986) concluded that *Acacia* was too broadly conceived and suggested that it be subdivided into three genera, *Acacia*, *Senegalia* Raf., and *Racosperma* Mart. Around the turn of the century, the results of molecular studies began to support such a division. At approximately the same time, a proposal was put forth to recognise an Australian species, *A.penninervis* Sieber ex DC., as the new type for a more narrowly circumscribed genus *Acacia* (Orchard and Maslin 2003). After much controversy, the name *Acacia* supplanted *Racosperma.* A consequence of these decisions was that species formerly recognised as *Acacia* sensu Pedley (1986) then became *Vachellia*.

No intrageneric classification has yet been published for *Vachellia*. Major works that present descriptions and/or keys include: Ebinger et al. (2000) and Rico-Arce (2007) for the Americas, Ross (1979) for Africa and Du Puy and Villiers (2002) for Madagascar, Nielsen (1981b, 1985a, 1992) for South East Asia and Malesia, and Orchard and Wilson (2001) for Australia, all under the name *Acacia*.

*Vachellia* species can readily be distinguished from other members of *Acacia* s.l., and especially the large segregate genus *Senegalia* that is also common in both Old and New World tropics, by the presence of stipular spines, the absence of prickles, the presence of an involucre on the peduncle, the absence of a torus-shaped nectary at the base of the ovary or ovary stalk, pollen polyads with colporate apertures and nexine possessing columellae, and different seedling development (Vassal 1972; Maslin et al. 2003; Seigler and Ebinger 2005). Characters that *Vachellia* shares with *Senegalia* include capitate, oblongoid or spicate inflorescences, and flowers that have numerous stamens (20–100).

Most *Vachellia* species have sessile petiolar glands, usually near the lowermost pair of pinnae, and smaller rachis glands are commonly found between the upper one or more pairs of pinnae. These glands, at least during the rapid growth associated with flower development, appear to produce a food reward for pollinators and various insects, especially ants.

The flower-heads of *Vachellia* species are visited by large numbers of different insects, especially bees, occasionally birds, and rarely by bats; however, pollination biology has only been examined in detail for a few species (Stone et al. 2003). Pollen is the principal floral reward but although a nectary disc is absent, nectar is produced in a minority of species (Stone et al. 2003). The inflorescences are commonly sweetly scented although the odour in *V.rigidula* (Benth.) Seigler & Ebinger is foetid.

Seeds of the swollen-thorn American *Vachellia* species are dispersed by birds (Janzen 1974). Some African species are dispersed by a range of ungulates, including giraffes and several species of antelopes, and by ostriches (Miller 1996).

Twelve Neotropical myrmecophytes share many adaptive ecological and morphological traits, most of which appear to be related to their mutualistic association with acacia-ants of the genus *Pseudomyrmex* Lund, 1831 (Janzen 1967a, 1967b, 1974). These species of *Vachellia* have inflated stipular spines usually inhabited by ants. Near the tip of many of these inflated spines, a small entrance hole is made by the ant queen when the spines are young and soft. Detachable tips, known as Beltian bodies after Thomas Belt, are found on the leaflets of most of the ant-acacias and they are rich in lipids, sugars and proteins. They are small, yellowish, ovoid to ellipsoid structures, about 3 mm long, and used as a food source by the ant-larvae. They are thought to have evolved in a symbiotic relationship with ants.

In approximately 11 species of African *Vachellia*, the stipular spines are distinctly and characteristically swollen into structures commonly known as ant-galls. These species are sometimes called Whistle Thorn acacias because of the noise caused by wind blowing over the ant entry holes. These galls are often inhabited by ants, commonly those of the genus *Crematogaster* Lund, 1831.

A number of hybrids have been identified in both Africa (Ross 1979, as *Acacia*) and the Americas where most *Vachellia* hybrids appear to be rare or uncommon, although swarms involving *V.macracantha* (Humb. & Bonpl. ex Willd.) Seigler & Ebinger, *V.pennatula* (Schltdl. & Cham.) Seigler & Ebinger, and *V.campeachiana* (Mill.) Seigler & Ebinger are common in central Mexico (Ebinger and Seigler 1987, 1992; Clarke et al. 2005).

#### Taxonomic references.

Ali (1973); Chakrabarty and Gandopadhyay (1996); Comben et al. (2020); Deshpande et al. (2019); Du Puy and Villiers (2002); Ebinger et al. (2000); Kodela and Wilson (2006); Kyalangalilwa et al. (2013); Lewis (1987); Lock (1989); Lock and Simpson (1991); Maslin (2015); Maslin et al. (2003, 2019a); Miller and Seigler (2012); Nielsen (1981b, 1985a, 1992); Orchard and Wilson (2001); Queiroz (2009); Ragupathy et al. (2014); Rico-Arce (2007); Ross (1975b, 1979, 1981); Smit (1999).

### ﻿ Anadenanthera

Taxon classificationPlantaeFabalesFabaceae

Speg., Physis (Buenos Aires) 6: 313. 1923.

Figs 164 , 165

Piptadenia sect. Niopa Benth., J. Bot. (Hooker) 4: 340. 1841. Type: Piptadeniaperegrina (L.) Benth. [≡ Mimosaperegrina L. (≡ Anadenantheraperegrina (L.) Speg.)]
Niopa (Benth.) Britton & Rose, Addisonia 12: 37, t. 403. 1927. Type: Niopaperegrina (L.) Britton & Rose [≡ Mimosaperegrina L. (≡ Anadenantheraperegrina (L.) Speg.)]

#### Lectotype

**(designated by Altschul 1964).***Anadenantheraperegrina* (L.) Speg. [≡ *Mimosaperegrina* L.]

#### Description.

**Figure 164.**
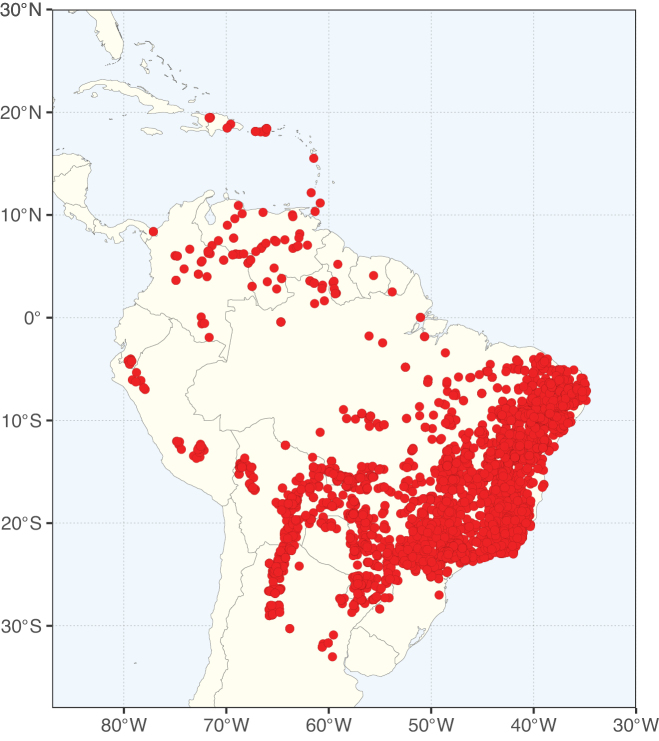
Morphological features of *Anadenanthera* (**A–I**) and Parkiasect.Platyparkia (**J–L**) **A***A.colubrina* (Vell.) Brenan capitulum cut in half, at anthesis **B** capitula at anthesis and in bud **C** shrub **D***Anadenanthera* sp. foliage and petiolar nectary **E***A.colubrina* mature pods **F** mammillate projections on trunk **G** dehisced pods **H**A.peregrinavar.falcata (Benth.) Altschul mammillate trunks **I**A.peregrina(L.)Speg.var.peregrina trees with smooth trunks **J***Parkiapendula* (Willd.) Benth. ex Walp. (*Hopkins & Hopkins 273*), Neotropics, tree crown **K** capitulum at anthesis, nectar exuding from apical flowers **L** pods with enlarged adaxial suture secreting gum into which the seeds have fallen. Photo credits **A–I** RT Queiroz https://rubens-plantasdobrasil.blogspot.com/**J–L** MJG Hopkins and HCF Hopkins.

Unarmed trees or shrubs, 3–30 m high; trunk ± smooth or with mammillate projections; bark often suberose, sometimes thick. **Stipules** small, bristly, caducous; bracts enclosing new shoots broad, commonly persistent. **Leaves** feathery; extrafloral nectaries on petiole above base, sometimes on rachis between ultimate pairs of pinnae, small, round; pinnae 7–35 pairs, opposite or almost so; leaflets 20–80 pairs per pinna, opposite or almost so, ± narrowly oblong, lanceolate, cultrate to slightly falcate, sometimes imbricate, main-vein central and straight. **Compound inflorescences** of pedunculate capitula, in fascicles of up to 7 peduncles inserted in series in successive leaf axils or forming terminal paniculate groups by suppression of leaves; peduncles each with 2 membranous bracts united to form an annular involucre; capitula spherical, 1–2 cm diameter including stamens, each with 35–60 flowers, greenish white to creamy yellow or rarely orange, fragrant. **Flowers** hermaphroditic or sometimes staminate and hermaphroditic [at least in *A.colubrina* (Vell.) Brenan], small, sessile; calyx campanulate, loosely gamosepalous, to 3 mm long; corolla tubular-campanulate, to 4 mm long, lobes loosely cohering to the level of the calyx mouth; stamens 10, far-exserted, free distally, adnate to corolla proximally, anther gland present (at least in bud, *A.colubrina*) or absent (*A.peregrina*); pollen in polyads of 8, 12 or commonly 16 grains, porate, exine granular, columellae absent (Altschul 1964; Guinet 1969, 1981b; Caccavari 2002; Soares EL et al. 2022); nectary disc absent; ovary sessile to subsessile. **Fruits** shortly stipitate, narrowly oblong, ± flattened, sometimes slightly falcate, dry-coriaceous, sometimes falsely septate internally, pulp and gum absent, dehiscent along 1 suture, sutures slightly to ± prominently thickened and sometimes ± contracted between the seeds, surface of valves glabrous, reticulately veined, shiny or scurfy to verrucose. **Seeds** 8–16 in 1 series, circular, thin-discoid with a thin, sharp rim, dark brown to black, shiny, pleurogram present (Fig. 164).

#### Chromosome number.

2*n* = 26 (24) (Santos et al. 2012).

#### Included species and geographic distribution.

**Figure 165.**
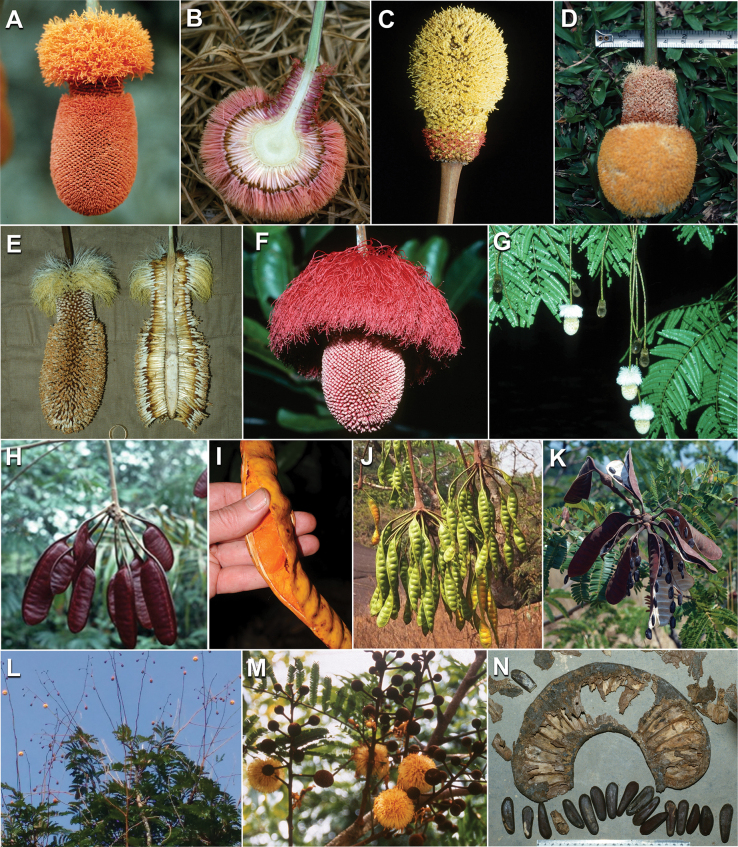
Distribution of *Anadenanthera* based on quality-controlled digitised herbarium records. Occurrences in the West Indies may be due to pre-Colombian naturalisation. See Suppl. material 1 for the source of occurrence data.

2–4 species, possibly more (Altschul 1964; Cacharani et al. 2020; Mangaravite et al. 2023). Endemic to South America (Fig. 165), from northern Argentina and Paraguay northwards into Bolivia and Peru, southern and eastern Brazil, southern Guyana and Venezuela, with a few occurrences in Amazonia; scattered in much of the northern Andes; although present in the West Indies (Hispaniola, Puerto Rico, Lesser Antilles, Trinidad-Tobago), it was probably introduced there in the pre-Columbian era (Altschul 1964).

#### Ecology.

Tropical and subtropical seasonally dry forests, along rivers and at forest margins, in woodland, thickets and wooded grassland (cerrado, savanna) and caatinga, often planted near villages, sometimes weedy and found along roadsides and on wasteland; growing on a range of substrates, sometimes dominant; to 2000 (–2700) m. Trees are partly deciduous.

#### Etymology.

*an*- (Gr., lacking), *aden*- (Gr., gland), *anthera* (L., anther), indicating a lack of anther glands, although *A.colubrina* has them in bud.

#### Human uses.

In pre-Columbian times, ground seeds of both species were used by Amerindians as a source of hallucinogenic snuff (*A.peregrina* – cohoba, niopo, yopo; A.colubrinavar.cebil – cebil, curupay, vilca), probably for 3000+ years. The northern species, *A.peregrina*, is still used for magical, medicinal, religious and stimulative purposes (Altschul 1972; Torres and Repke 2006). *Anadenantheracolubrina* (angico) is used for timber, paper, and leather-tanning.

#### Notes.

The long-established treatment by Altschul (1964) recognised only two species (*Anadenantheraperegrina*, *A.colubrina*) with overlapping distributions, each containing two varieties with a third subsequently added to *A.colubrina* in Argentina (Cacharani et al. 2020). A recent genetic study by Mangaravite et al. (2023) elevated Altschul’s varieties to specific level, reinstating Brenan’s (1955) species concepts by recognising *A.falcata* (Benth.) Speg. and *A.macrocarpa* (Benth.) Brenan to give four species in total. However, this study, based on material from southern and eastern Brazil, does not cover the entire distribution area of the genus in South America and the Caribbean and may still not reflect the full genetic diversity within the genus.

Bentham (1841b) placed the species he recognised in Piptadeniasect.Niopa Benth. under five names, all based on South American material. One of them, *P.peregrina* (L.) Benth., was subsequently treated as a distinct but related genus *Anadenanthera* by Spegazzini (1923), who excluded several un-named Australian taxa that Taubert (1894) had suggested also belonged to sect. Niopa, as well as another that is now a synonym of *Schleinitzianovoguineensis* (Warb.) Verdc. Britton and Rose (1927) created the genus *Niopa* to contain *N.peregrina*, but Brenan (1955) resurrected the name *Anadenanthera* as it had priority and this was then used by Altschul (1964) in her monograph. This work gave detailed accounts of the taxa and their morphology. The species of *Anadenanthera* had been atypical within *Piptadenia* because of their small, globose, rather than spicate, inflorescences (Bentham 1875) and pods that dehisce along only one suture, rather than more or less along both (Lewis and Elias 1981). Lewis and Elias (1981) assigned *Anadenanthera* to the *Piptadenia* group of tribe Mimoseae, as did Luckow et al. (2003) and Luckow (2005).

In addition to its capitula and dehiscent pods, distinctive characters of *Anadenanthera* include the thin, rimmed or very narrowly winged seeds that are circular in outline and lack endosperm. The species recognised by Altschul (1964) differ from one another in the texture of the pods, the presence/absence of anther glands and the position of the involucre on the peduncle.

The use of *Anadenanthera* as an hallucinogen is dependent on the presence in the seeds, fruits and bark of many populations of a range of indole alkaloids derived from tryptamine and related to serotonin; among these, bufotenin is often especially abundant and psychoactive (Torres and Repke 2006). Some of these compounds are present in other Mimoseae but at much lower concentrations.

Plants of *Anadenantheracolubrina* are partially self-incompatible and its main pollinators are diurnal eusocial bees; eusocial honey wasps are nectar thieves or sometimes pollinators, and other insects are largely thieves of nectar and/or pollen (Kiill and da Silva 2016; Borges et al. 2017). Unusually, nectar is produced by the corolla lobes in this species (Borges et al. 2017).

Trees are possibly mast fruiting and seed dispersal is by gravity and probably wind and leaf-cutter ants (Fredericksen et al. 2000). Insect seed-predators include Curculionidae (Justiniano and Fredericksen 1998) and bark exudates are consumed by marmosets (Callitrichidae) (Fransisco et al. 2017). The nodulated roots form a large tuber in *Anadenantheraperegrina* (Gross et al. 2002).

#### Taxonomic references.

Altschul (1964); Cacharani et al. (2020); Mangaravite et al. (2023); Martinez et al. (2013).

### ﻿ Parkia

Taxon classificationPlantaeFabalesFabaceae

R. Br. in Denham & Clapperton, Narr. Travels Africa, App.: 234. 1826.

**Figure 166.**
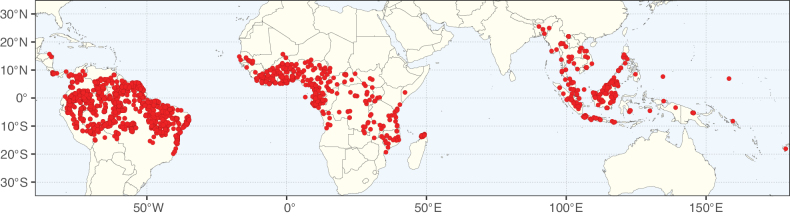
Morphological features of Parkiasect.Parkia (**A–L**) and sect. Sphaeroparkia (**M, N**) **A***P.bicolor* A. Chev. pendent capitulum approaching anthesis, Korup National Park, Cameroon **B***P.biglobosa* (Jacq.) R. Br. ex G. Don pendent capitulum cut in half, Ibadan, Nigeria **C***P.decussata* Ducke erect capitulum at anthesis, Neotropics (*Hopkins & Hopkins 237*) **D***P.timoriana* (DC.) Merr. pendent capitulum, South East Asia (*H.C.F. Hopkins 634*) **E***P.gigantocarpa* Ducke pendent capitulum and another cut in half, shortly post-anthesis, finger ring gives scale, Neotropics (*Hopkins & Hopkins 298*) **F***P.igneiflora* Ducke pendent capitulum near anthesis, Neotropics (*Hopkins & Hopkins 230*) **G***P.speciosa* Hassk., capitula at anthesis, Temburong, Brunei **H***P.discolor* Spruce ex Benth. indehiscent pods nearing maturity, Neotropics (*Hopkins & Hopkins 264*) **I, J***P.bicolor***I** ripe indehiscent pod with yellow valves containing orange pulp, Bero Mts, Guinea-Conakry **J** immature pods, Korup National Park, Cameroon **K***P.cachimboensis* H.C. Hopkins dehiscent pods lacking gum, the seeds attached by their funicles, Serra do Cachimbo, Brazil **L**P.igneifloravar.aurea Ducke vel aff. erect compound inflorescence axes projecting above the tree crown bearing pendent yellow capitula on short pendent peduncles, Cachoeira Berro d’Agua, AM, Brazil **M, N***P.multijuga* Benth. **M** capitula at anthesis and in bud, Trombetas, Brazil **N** old pod from ground plus seeds, INPA, Manaus, Brazil. Photo credits **A** R Grünmeier **B** HCF Hopkins **C–F, H, K, N** MJG Hopkins and HCF Hopkins **G** I Nielsen **I** M Cheek **J** X van der Burgt **L** L Mello **M** unknown.

**Figure 167.**
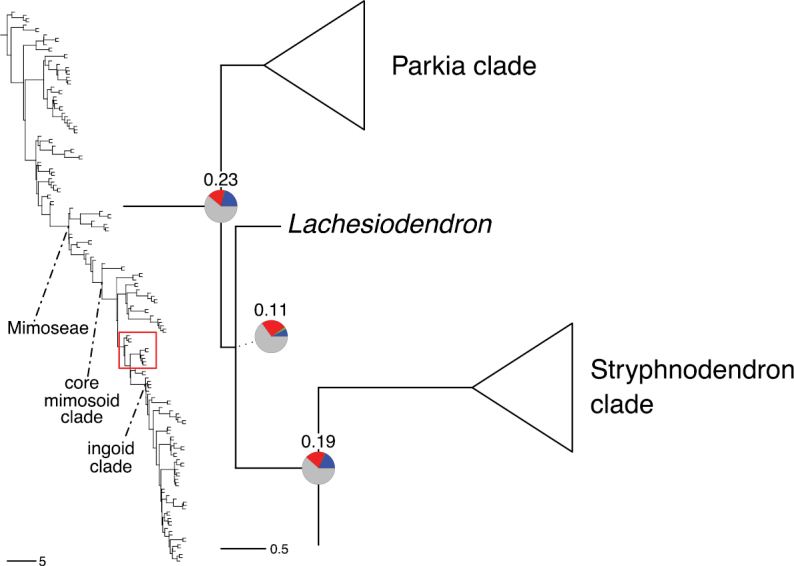
Distribution of *Parkia* based on quality-controlled digitised herbarium records. The presence of the West African *P.biglobosa* (Jacq.) R. Br. ex G. Don in Haiti, where it is naturalised, is not shown. See Suppl. material 1 for the source of occurrence data.

Figs 164 , 166 , 167

Paryphosphaera H. Karst., Fl. Columb. 2: 7, tab. 104. 1862. Type: Paryphospheraarborea H. Karst. [= Parkianitida Miq.]

#### Type.

*Parkiaafricana* R. Br., nom. superfl. [≡ *Parkiabiglobosa* (Jacq.) R. Br. ex G. Don (≡ *Mimosabiglobosa* Jacq.)]

#### Description.

Unarmed trees or rarely shrubs, 3–40 m, evergreen or rarely deciduous; trunk sometimes buttressed, bark variable. **Stipules** small, caducous. **Leaves** alternate, (sub)opposite or clustered at the ends of twigs; pinnae 1–55 pairs, opposite, subopposite or rarely alternate; leaflets 3–110 pairs per pinna, opposite or rarely alternate (*P.biglobosa*), linear to oblong or slightly sigmoid or rarely elliptic and 3–45 × 1–13 mm, or rarely ovate (*P.singularis* Miq.) and then to 120 × 75 mm; main-vein central, straight or slightly sigmoid; extrafloral nectaries often present on petiole near the base, elliptic, single or double (or heart-shaped), and sometimes on the rachis between the pinnae, especially in seedlings, small, round. **Compound inflorescences** of pedunculate capitula arranged in axillary or terminal, short to very long racemes or panicles; principal axis 0.15–5 m long, erect, horizontal, pendent or projecting at all angles, within or beneath the crown to far-extending beyond it; peduncles alternate or (sub)opposite, 1–115 cm long, pendent, erect or projecting at all angles, tough, sometimes thick and robust; 4 caducous bracts enclosing the capitulum in young bud stage. **Capitula** of 3 types: in sect. Sphaeroparkia: globose, 1–5 cm diameter, with 120–650 flowers, all fertile, lacking specialised nectar-secreting flowers, red or yellow at anthesis; in sect. Platyparkia: oblate, 2.7–3.5 × 4–5 cm, with 1060–1325 flowers, these of 2 sorts, those in the middle and at the base fertile, those at the apex modified and nectar-secreting, capitula red; in sect. Parkia: clavate, subglobose or biglobose, 4–21.5 × 3–8 cm, with 1090–3240 flowers, these of 3 main sorts: fertile ones forming an apical ball, below this a constricted cylinder or depressed ring of nectar-secreting flowers, at the base a zone of staminodial flowers in which the filaments are short to far-projecting and then forming a wide fringe, capitula yellow (sometimes the fringe white), reddish (bright to dull red, pink, orange or purplish), or occasionally bicoloured (red at the base, apical ball yellow). **Flowers** tubular, each subtended by an obdeltate-spathulate bract, slightly longer than the calyx. **Fertile flowers** hermaphroditic, functionally staminate, or a mixture; calyx almost bilabiate with 2 large lobes and 3 smaller ones, lobes imbricate in bud (or sub-equal and sub-imbricate in *P.ulei* (Harms) Kuhlm.); corolla lobes with lower parts variously connate and often adnate to the filament-tube; stamens 10, shortly exserted, filaments usually connate proximally and free distally, anthers basifixed (most species) or dorsifixed (sect. Sphaeroparkia), with or without an apical gland; pollen in polyads of 16, 28 or 32 grains, porate, exine granular or with columellae, variously ornamented (Guinet 1981b; Feuer et al. 1985; Feuer 1986; Luckow and Hopkins 1995), polyads sometimes with a central cavity (Capucho and Teixeira 2014); nectary disc absent; ovary stipitate, gynoecium reduced in functionally staminate fertile flowers. **Nectar-secreting flowers** sterile, the basal parts of the calyx, corolla and androecium adnate, much thickened and nectariferous, gynoecium absent (sect. Parkia) or modified with the style exserted (sect. Platyparkia). **Staminodial flowers** sterile, the filaments often bearing minute, non-functional anthers, gynoecium absent. **Fruits** borne on a large, woody, claviform to ellipsoid receptacle with a narrowly terete base (receptacle smaller in sect. Sphaeroparkia), stipitate, coriaceous to thick-woody, rarely tough-fleshy (*P.platycephala* Benth.), to 60 cm long, indehiscent or dehiscent along the adaxial suture, strap-shaped, narrowly oblong, oblong or rarely terete (e.g., *P.biglobosa*), sub-moniliform (e.g., *P.filicoidea* Welw. ex Oliv. p.p.) or broadly crescent-shaped (*P.multijuga* Benth.), sometimes twisted or rarely ± curled, sometimes containing pulp (Paleotropics) or gum (Neotropics), or gum secreted along a laterally enlarged dehiscent adaxial suture (sect. Platyparkia p.p.). **Seeds** 6–34 per pod in 1 or rarely 2 rows (sect. Platyparkia p.p.), flattened-ellipsoid or otherwise, 7–60 mm long; testa hard, thick, dark (rarely soft, green, *P.speciosa* Hassk.) with a pleurogram, or rarely thin and pleurogram lacking (Figs 164, 166).

#### Chromosome number.

2*n* = 26 (22, 24) (Santos et al. 2012).

#### Included species and geographic distribution.

Currently ca. 35 species but more are likely to be recognised as a result of genetic studies (e.g., Ahossou et al. 2020). Species are arranged in three sections: sect. Parkia (ca. 30 species), pantropical; sect. Platyparkia [three species: *P.paraensis* Ducke, *P.pendula* (Willd.) Benth. ex Walp., *P.platycephala*], South and Central America; sect. Sphaeroparkia (three species: *P.multijuga*, *P.ulei*, *P.velutina* Benoist), South America.

The genus is pantropical (Fig. 167) and includes ca. 20 species in the Neotropics, all endemic, from Bolivia and coastal Brazil north to Honduras, plus one African species (*Parkiabiglobosa*) naturalised in Haiti (omitted from map), introduced to this and other islands in the West Indies in the 17–18^th^ century; most species are Amazonian and the genus is only rarely found west of the Andes. In mainland Africa: at least three species species, all endemic. In Madagascar: one species, endemic. In the Indo-Pacific: 11 species, including two probably extinct, all endemic, from north-east India eastwards to south-east China, and through South East Asia and Malesia into the Pacific as far east as Ponape and Fiji.

#### Ecology.

Tropical, predominantly occurring in moist habitats; most species are found in lowland rainforest (occasionally to 1500 m elevation), others grow in riparian forest, fresh-water flooded forest (várzea and igapó), woodland and wooded grassland (cerrado, savanna), and campinarana. Less common habitats in South America include coastal restinga (*P.bahiae* H.C. Hopkins), rocky savanna (cerrado rupestre; *P.cachimboensis* H.C. Hopkins) and sub-Andean dwarf forest (*P.nana* D.A. Neill), and in South East Asia and Malesia, peat swamp forest (*P.paya* H.C. Hopkins), tidal streams and *Nypa* swamp (*P.sherfeseei* Merr.), and dry evergreen and/or deciduous forest (*P.leiophylla* Kurz, *P.sumatrana* Miq.).

#### Etymology.

Named for the Scottish explorer Mungo Park (1771–1806), who investigated the course of the Niger River in West Africa and mentioned what became *Parkiabiglobosa* as the nitta tree in the account of his first expedition to the region (Park 1799).

#### Human uses.

In West Africa, the seeds of *Parkiabiglobosa* (African locust bean, néré, nété) are fermented into a widely used pungent condiment (dawadawa, soumbala, iru); the sweet mealy pulp around the seeds is also consumed (Campbell‐Platt 1980; Hall et al. 1997; Termote et al. 2022). In South East Asia, the sulphurous smelling seeds of *P.speciosa* are eaten fresh or tinned as a vegetable (petai, pete, sator, stinkbean) (e.g., Wiriadinata and Bamroongrugsa 1993; Woon 1995), and the seeds of *P.timoriana* (DC.) Merr. are consumed in a similar manner in north-east India (Singh 2022). The pods of *P.platycephala* are used to feed cattle and goats in north-east Brazil (Hopkins 1986; Sousa et al. 2015). Numerous traditional medicinal uses have been reported, especially in Africa and Asia, and chemical characteristics suggest much wider medicinal potential (e.g., Saleh et al. 2021).

#### Notes.

Following Bentham (1841a, 1875), *Parkia* was traditionally placed with *Pentaclethra* Benth. in the tribe Parkieae (Wight & Arn.) Endl. because both genera have a calyx with imbricate lobes. Bentham (1875) listed this tribe first (e.g., page 358), presumably to reflect a basal position in his suborder Mimoseae, linking it to the caesalpinioids, but he was equivocal about the affinity of the two genera and sometimes elsewhere in this work he treated them separately. Although it was clear that *Parkia* and *Pentaclethra* were unlikely to be closely related (Bentham 1875; Guinet 1969; Elias 1981b), they were not formally separated until Luckow et al. (2003) and Luckow (2005) placed them in different parts of the Mimoseae, although neither were assigned to a formal group within the tribe. The zygomorphic calyx with imbricate lobes that is so distinctive in *Parkia* is clearly a derived character, probably related to floral packing in the large bud capitula.

The generic limits of *Parkia* are unchanged from those of Bentham (1875) and Ducke (1932b, 1949), who devised the sectional classification, slightly modified by Hopkins (1986). Diagnostic characters for the genus include the form of the calyx, the large size of the usually densely-flowered capitula (except *P.ulei*), and marked floral differentiation in sections *Parkia* and *Platyparkia*. The capitulum in sect. Platyparkia, with nectar-secreting flowers at the apex (Fig. 164K), is unique in Mimoseae. The fruits of two of its species (*P.paraensis*, *P.pendula*), in which copious sticky gum is secreted along a laterally enlarged dehiscent adaxial suture, may also be unique (Fig. 164L). The capitula of the species in sect. Parkia that have long staminodes projecting from their basal flowers (Fig. 166A, E, F, G) are superficially similar to those of *Dichrostachyscinerea* (L.) Wight & Arn., and the pale yellow capitula of *P.ulei* resemble those of some other Mimoseae (e.g., *Leucaena* Benth.) in size, colour and arrangement.

*Parkia* is one of the most variable genera in the Mimoseae. However, despite variation in the structure of the capitula, in the morphology of the flowers, fruits and seeds, in the type of germination (phaneroepigeal, phanerogeal or cryptohypogeal) and in pollen sculpturing (Hopkins 1986; Luckow and Hopkins 1995), it has been shown to be monophyletic (Luckow and Hopkins 1995; Koenen et al. 2020a; Oliveira et al. 2021; Ringelberg et al. 2022). The cradle of the genus is the Americas (Oliveira et al. 2021), and the greatest morphological diversity and species richness also occur here. Inter-continental trans-oceanic dispersal has most likely been facilitated by an ability in some species (e.g., *P.discolor* Spruce ex Benth.) of the pods to float and the seeds to withstand prolonged immersion in salt water (Hopkins 1986).

This genus has a number of unusual characters compared with others in this and closely related clades. Some appear idiosyncratic, such as the opposite leaves in a few Neotropical and one Asian species, and the lack of root nodulation. However, many of its distinctive features can be related to reproductive biology, including the sometimes very elongated compound inflorescence axes, tough and sometimes long, pendent or erect peduncles, capitula commonly composed of very numerous, relatively large flowers, foetid floral odours, and crepuscular/nocturnal anthesis (diurnal only in *P.ulei*). Sections *Parkia* and *Platyparkia* are pollinated by bats that typically land on the capitula to lap nectar (rather than by hovering to feed), belonging to the Phyllostomidae in the Americas and the Pteropodidae in Africa, Asia and the Pacific; various non-volant mammals, insects including bees, and birds, are occasional pollen vectors and nectar and/or pollen thieves (e.g., Pettet 1977; Grünmeier 1990; Birkinshaw and Colquhoun 1998; Hopkins 1998; Piechowski et al. 2010; Lassen et al. 2012; Kobayashi et al. 2021). The smaller, less specialised capitula of sect. Sphaeroparkia are insect-pollinated (*P.ulei*: diurnal bees; *P.velutina*: nocturnal bees; *P.multijuga*: diverse small insects including beetles and thrips) (Hopkins et al. 2000; Chaves 2015). Partial self-incompatibility has been demonstrated in *P.biglobosa* (Lassen et al. 2012).

The wide range in fruit characters is reflected in a variety of dispersal mechanisms. Seed-dispersers include chimpanzees, various Neotropical and Paleotropical monkeys and perhaps birds, and for fruits that fall to the ground readily at maturity, large rodents (*Parkiamultijuga*, Fig. 166N), ruminants, and water (*P.discolor*) (Hopkins 1983; Hopkins and Hopkins 1983; Bertolani and Pruetz 2011). In Africa, the sweet pulp around the seeds (Fig. 166I, *P.bicolor*) and to a lesser extent the seeds themselves are attractive to primates. Amongst Neotropical monkeys, marmosets and tamarins (Callitrichidae) in particular consume the gum that is found inside the indehiscent fruits of many species in sect. Parkia or exuded from the dehiscent ones of *P.pendula* (Peres 2000). Some Callitrichidae also consume exudates from gouging the bark (e.g., Ramírez et al. 1977). Insect seed-predators include moths and, particularly in the Americas, bruchid beetles (Chrysomelidae: Bruchinae) (Hopkins 1984).

#### Taxonomic references.

Ducke (1949); Hagos (1962); Hopkins (1983, 1986, 1994); Nielsen (1981b, 1985a, 1992); Villiers (2002).

## ﻿﻿23. *Lachesiodendron*

Pétala Gomes Ribeiro^2^, Leonardo M. Borges^5^

Citation: Ribeiro PG, Borges LM (2024) 23. Lachesiodendron. In: Bruneau A, Queiroz LP, Ringelberg JJ (Eds) Advances in Legume Systematics 14. Classification of Caesalpinioideae. Part 2: Higher-level classification. PhytoKeys 240: 316–318. https://doi.org/10.3897/phytokeys.240.101716

### ﻿ Lachesiodendron

Taxon classificationPlantaeFabalesFabaceae

P.G. Ribeiro, L.P. Queiroz & Luckow, Taxon 67(1): 45. 2018.

**Figure 168.**
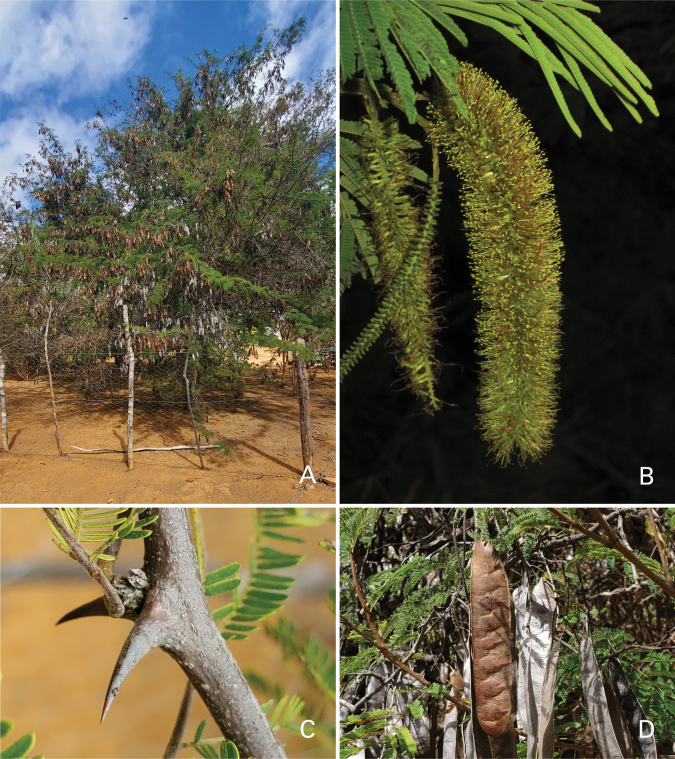
Phylogenetic relationships of *Lachesiodendron* in tribe Mimoseae. For description of phylogeny and support values, see Fig. 6 caption (page 63).

**Figure 169.**
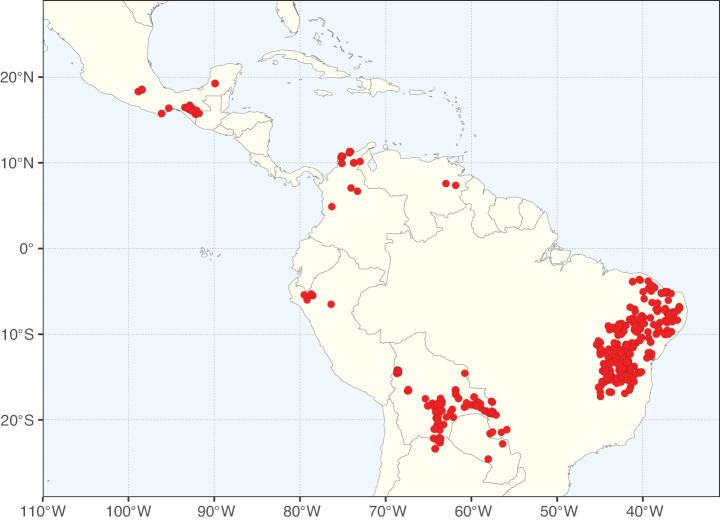
*Lachesiodendronviridiflorum* (Kunth) P.G. Ribeiro, L.P. Queiroz & Luckow **A** habit **B** inflorescence **C** pair of persistent spinescent stipules **D** fruits. Photo credits **A, C, D** PG Ribeiro **B** LP Queiroz.

**Figure 170.**
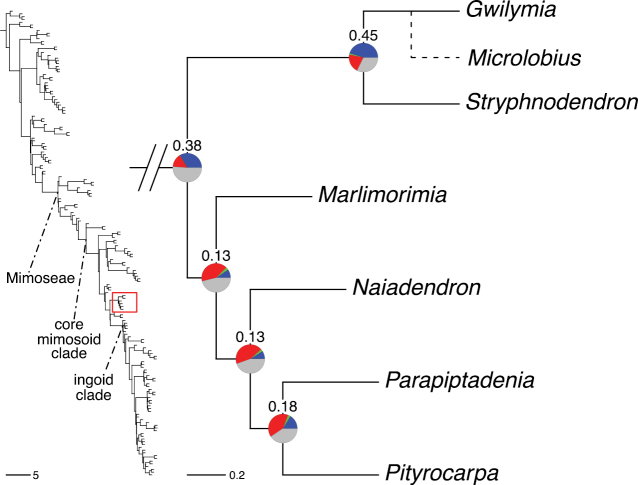
Distribution of *Lachesiodendron* based on quality-controlled digitised herbarium records. See Suppl. material 1 for the source of occurrence data.

Figs 168 , 169 , 170

#### Type.

*Lachesiodendronviridiflorum* (Kunth) P.G. Ribeiro, L.P. Queiroz & Luckow [≡ *Acaciaviridiflora* Kunth]

*Lachesiodendron* has an isolated position in the Mimoseae phylogeny, between the Parkia clade and the node including the Mimosa, Stryphnodendron and Ingoid clades (Koenen et al. 2020a; Ringelberg et al. 2022; Fig. 168). Because *Lachesiodendron* is not resolved with any of these clades, it is here treated as a distinct, monospecific lineage within Mimoseae.

#### Description.

Trees (2) 3–20 m; indumentum puberulent to rarely glabrous; brachyblasts absent; branches armed with stipules modified into lignified spines, down-curved, paired at branch nodes, prickles absent, lenticels present. **Stipules** spinescent. **Leaves** bipinnate, unarmed; extrafloral nectaries on the petiole, on the leaf rachis between distal pairs of pinnae, and on the pinnae between distal pairs of leaflets; pinnae 5–15 pairs, opposite or sub-opposite; leaflets 20–50 pairs, opposite. **Inflorescences** 1–2 (3) axillary spikes. **Flowers** 5-merous, yellowish green; calyx gamosepalous, campanulate; corolla gamopetalous, cylindrical; stamens 10, anthers with a short-stipitate caducous apical gland; pollen in 8-grained polyads; ovary glabrous, long stipitate, exserted from the corolla, stigma in a terminal pore. **Fruit** a flat-compressed legume with thick margins, straight, not constricted between the 8–10 seeds, valves thin, coriaceous. **Seeds** ovate to obovate with a U-shaped pleurogram on both faces (Fig. 169).

#### Chromosome number.

Unknown.

#### Included species and geographic distribution.

Monospecific (*L.viridiflorum*), in tropical America, disjunctly from southern and western Mexico to northern Argentina (Fig. 170).

#### Ecology.

Confined to seasonally dry tropical forests and woodlands.

#### Etymology.

From ‘Lachesis’, in reference to the bushmaster viper [*Lachesismuta* (Linnaeus, 1766)] whose vernacular name (surucucu) is also applied to the tree in Brazil, probably because the pair of nodal spines resembles the viper’s fangs (Fig. 169), and -*dendron* (Greek = tree).

#### Human uses.

*Lachesiodendronviridiflorum* is used as fodder, for the timber, for environmental restoration, and provides good quality firewood and charcoal (Carvalho 2014). The species could be used for beekeeping, as it produces abundant nectar, and is potentially medicinal due to the presence of tannins in the bark (Carvalho 2014).

#### Notes.

*Lachesiodendron* was recently described by Ribeiro et al. (2018) to accommodate a single species of *Piptadenia* that did not group with the remainder of the genus, or with any other mimosoid genera in phylogenetic analyses (Jobson and Luckow 2007; Simon et al. 2011, 2016; Ribeiro et al. 2018). This circumscription is supported by an unusual combination of morphological characters: stipules modified into lignified spines; absence of prickles; 1–2 (3) spikes 20–22 mm in diameter at anthesis, in fascicles in the axils of coevally developing leaves; cylindrical corolla, much longer than the calyx; unusual greenish flowers which gave rise to the species epithet *viridiflorum*; polyads with 8 grains arranged in two opposing tetrads (Ribeiro et al. 2018).

#### Taxonomic references.

Ribeiro et al. (2018).

## ﻿﻿24. Stryphnodendron clade

Leonardo M. Borges^5^, Marcelo F. Simon^40^, Pétala Gomes Ribeiro^2^, Melissa Luckow^29^, Alexandre Gibau de Lima^8,28^

Citation: Borges LM, Simon MF, Ribeiro PG, Luckow M, Lima AG (2024) 24. Stryphnodendron clade. In: Bruneau A, Queiroz LP, Ringelberg JJ (Eds) Advances in Legume Systematics 14. Classification of Caesalpinioideae. Part 2: Higher-level classification. PhytoKeys 240: 319–331. https://doi.org/10.3897/phytokeys.240.101716

**Stryphnodendron clade**

Figs 171–180

**Included genera (7).***Gwilymia* A.G. Lima, Paula-Souza & Scalon (7 species), *Marlimorimia* L.P. Queiroz, L.M. Borges, Marc.F. Simon & P.G. Ribeiro (6), *Microlobius* C. Presl (1), *Naiadendron* A.G. Lima, Paula-Souza & Scalon (1), *Parapiptadenia* Brenan (6), *Pityrocarpa* (Benth.) Britton & Rose (7), *Stryphnodendron* Mart. (28).

**Description.** Trees, rarely shrubs or subshrubs; indumentum composed of simple trichomes and sometimes also reddish granular trichomes; brachyblasts absent or present; branches and leaves unarmed, with a garlic smell in one genus. **Stipules** absent or present, usually caducous. **Leaves** bipinnate, extrafloral nectaries present on the petiole, rachis and pinnae; pinnae 2–many pairs, opposite or subopposite, variable in size and shape; leaflets 1–many pairs, opposite or alternate, variable in size and shape. **Inflorescences** cylindrical spikes, solitary or in groups, in the axils or supra-axillary to coevally developing leaves, or in efoliate nodes, sometimes further arranged in complex synflorescences. **Flowers** 5-merous; calyx gamosepalous; corolla gamopetalous; stamens 10, anthers with an apical gland; pollen usually in 16-grained polyads, but sometimes in groups of 4–32 grains. **Fruit** a dehiscent or indehiscent legume or a follicle, variable in shape and size. **Seeds** ellipsoid or flat-compressed, winged or not, brown, dark or white, pleurogram absent or present.

**Geographic distribution.** Tropical America, from Mexico to Argentina and Uruguay.

**Figure 171.**
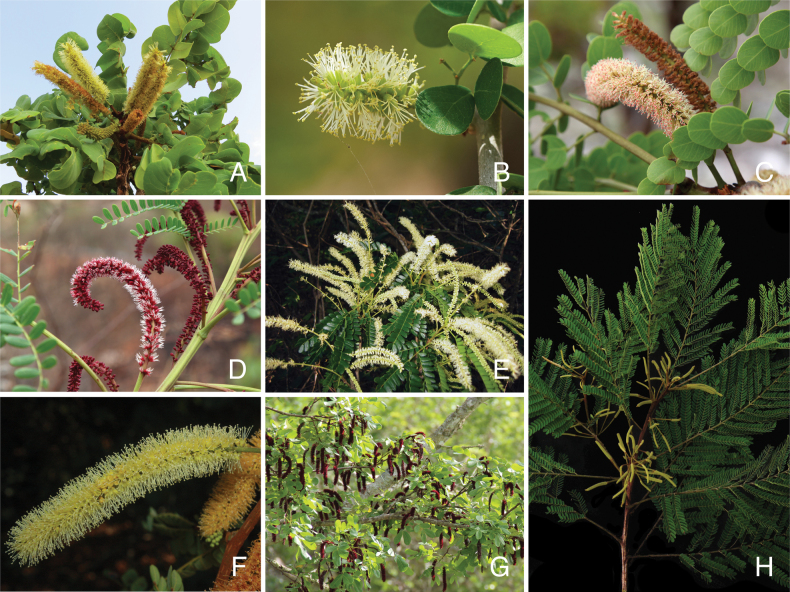
Generic relationships in the Stryphnodendron clade (tribe Mimoseae). The most likely position of unsampled genus *Microlobius* is indicated with a dashed line [following Simon et al. (2016) and Lima et al. (2022)]. For description of phylogeny and support values, see Fig. 6 caption (page 63).

**Notes.** Members of the Stryphnodendron clade are mostly trees, always unarmed, with spicate inflorescences, and pentamerous, diplostemonous flowers with glands at the apex of the anthers (Fig. 172). Although sharing this general floral and inflorescence morphology, the genera differ in synflorescence organisation, as well as in leaf and fruit traits (Jobson and Luckow 2007; Simon et al. 2016; Borges et al. 2022; Lima et al. 2022) (Figs 172, 173). Nonetheless, circumscription of the clade’s genera has been in a state of flux, particularly as phylogenetic analyses showed that many were not monophyletic and that homoplasy was pervasive across the group, including in traits previously thought to be taxonomically important (Simon et al. 2016; Ribeiro et al. 2018; Ringelberg et al. 2022). As a consequence, the Stryphnodendron clade includes a number of small, recently described genera (Borges et al. 2022; Lima et al. 2022), some of which mirror previous infrageneric subdivisions of *Piptadenia* (Bentham 1875; Simon et al. 2016).

**Clade-based definition.** The most inclusive crown clade including *Stryphnodendronadstringens* (Mart.) Coville and *Pityrocarpamoniliformis* (Benth.) Luckow & R.W. Jobson, but not *Vachelliatortilis* (Forssk.) Galasso & Banfi, *Piptadeniaadiantoides* (Spreng.) J.F. Macbr. or *Lachesiodendronviridiflorum* (Kunth) P.G. Ribeiro, L.P. Queiroz & Luckow (Fig. 171).

**Figure 172.**
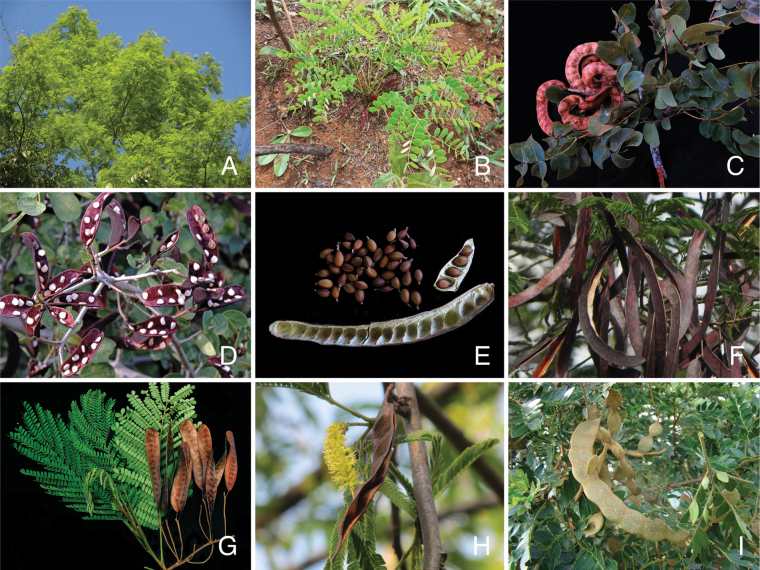
Flowering branches in the Stryphnodendron clade **A***Gwilymiacoriacea* (Benth.) A.G. Lima, Paula-Souza & Scalon (*Simon 3482*) **B***Microlobiusfoetidus* (Jacq.) M. Sousa & G. Andrade **C***Stryphnodendronadstringens* (Mart.) Coville **D***S.gracile* Rizzini & Heringer **E***Marlimorimiabahiana* (G.P. Lewis & M.P. Lima) L.P. Queiroz & L.M. Borges **F***Pityrocarpamoniliformis* (Benth.) Luckow & R.W. Jobson **G***Parapiptadeniablanchetii* (Benth.) Vaz & M.P. Lima **H***Naiadendronduckeanum* (Occhioni) A.G. Lima, Paula-Souza & Scalon (*Simon 1457*). Photo credits **A, H** MF Simon **B** T Iwane **C, D** H Moreira **E** LP Queiroz **F** D Cardoso **G** PG Ribeiro.

**Figure 173.**
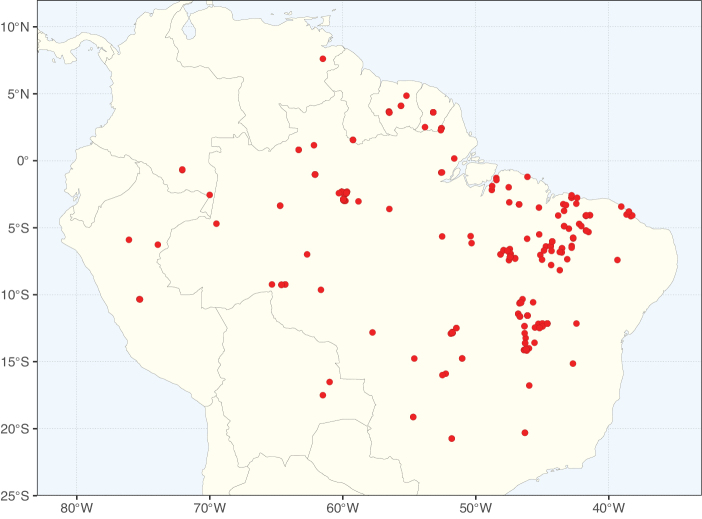
Habit and fruits in the Stryphnodendron clade **A***Marlimorimiapsilostachya* (DC.) L.P. Queiroz & Marc.F. Simon showing the tree habit **B***Stryphnodendronplatyspicum* Rizzini & Heringer (*Simon 2017*) showing the geoxyle subshrub habit **C***Gwilymiacoriacea* (Benth.) A.G. Lima, Paula-Souza & Scalon (*Simon 3730*) **D***Microlobiusfoetidus* (Jacq.) M. Sousa & G. Andrade **E***Stryphnodendronrotundifolium* Mart. fruit, manually opened revealing internal septa and seeds **F***Marlimorimiacontorta* (DC.) L.P. Queiroz & P.G. Ribeiro **G***Naiadendronduckeanum* (Occhioni) A.G. Lima, Paula-Souza & Scalon (*Pereira-Silva 15692*) **H***Parapiptadeniarigida* (Benth.) Brenan showing a yellow inflorescence and dehiscing fruit **I***Pityrocarpaleptostachya* (Benth.) L.P. Queiroz & P.G. Ribeiro. Photo credits **A** R Aguilar **B, C, E** MF Simon **D** H Hulsberg **F** G Carvalho-Sobrinho **G** G Pereira-Silva **H** RT Queiroz https://rubens-plantasdobrasil.blogspot.com/**I** LM Borges.

Similarly to the Mimosa clade (page 332), genera of the Stryphnodendron clade have diplostemonous flowers, pollen in tetrads or polyads and a narrowing style with a small porate stigma at the tip and, thus, were included in the informal Piptadenia group of Mimoseae (Lewis and Elias 1981). Although the Piptadenia group has not been supported as monophyletic, a subgroup of the genera were shown to form a monophyletic group, referred to as the Stryphnodendron clade (Jobson and Luckow 2007; Simon et al. 2016) and supported in recent phylogenomic analyses (Koenen et al. 2020a; Ringelberg et al. 2022).

### ﻿ Gwilymia

Taxon classificationPlantaeFabalesFabaceae

A.G. Lima, Paula-Souza & Scalon, PhytoKeys 205: 2019. 2022.

**Figure 174.**
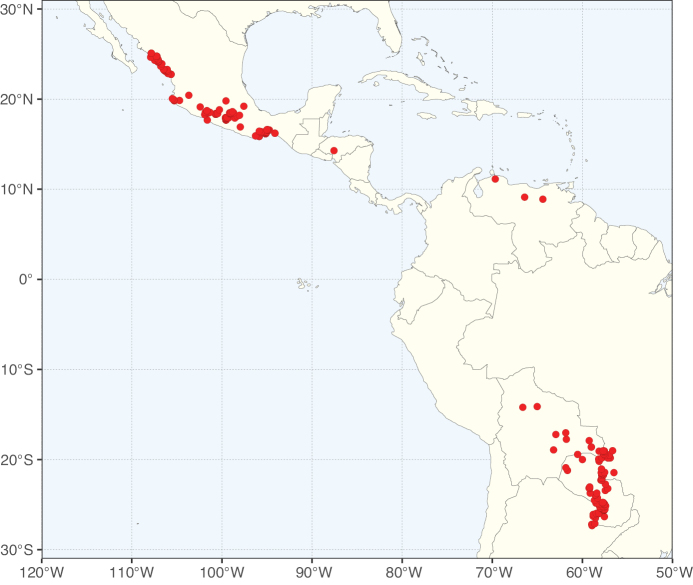
Distribution of *Gwilymia* based on quality-controlled digitised herbarium records. See Suppl. material 1 for the source of occurrence data.

Figs 172 , 173 , 174

#### Type.

*Gwilymiapaniculata* (Poepp. & Endl.) A.G. Lima, Paula-Souza & Scalon [≡ *Stryphnodendronpaniculatum* Poepp. & Endl.]

#### Description.

Trees; indumentum composed of simple and granular trichomes; brachyblasts absent; branches unarmed, young shoots and leaves covered with reddish granular trichomes. **Stipules** caducous. **Leaves** bipinnate; extrafloral nectaries on the petiole, between pinnae and between apical leaflets; pinnae 2–4 (6) pairs, opposite or subopposite; leaflets 3–5 pairs, opposite, variable in shape. **Inflorescence units** cylindrical spikes, arranged in fascicles of 2–5 spikes in pseudoracemes or panicles. **Flowers** 5-merous, whitish, yellowish or reddish; calyx gamosepalous, cupulate; corolla gamopetalous, cupulate to tubular; stamens 10, anthers with an apical gland; pollen in 16-grained polyads, but also in groups of 4–28 grains; ovary included in the corolla. **Fruit** an indehiscent nucoid legume, curved, falcate or spiralled; valves coriaceous or woody. **Seeds** lenticular, wingless, pleurogram present.

#### Chromosome number.

Unknown.

#### Included species and geographic distribution.

Seven species of which six occur in Brazil, with one extending to Bolivia and another to French Guiana, Guyana, Suriname and Venezuela. One species is narrowly endemic in French Guiana (Fig. 174).

#### Ecology.

Five species occur in the Amazon rainforest, while two are found in Cerrado savannas, one of which also extends to the seasonally dry tropical forests and woodlands of north-eastern Brazil.

#### Etymology.

*Gwilymia* is in homage to the Royal Botanic Gardens, Kew botanist, Dr. Gwilym P. Lewis.

#### Human uses.

Unknown.

#### Notes.

The genus was recently described to accommodate species previously assigned to *Stryphnodendron*, but that were both morphologically and phylogenetically distinct (Lima et al. 2022; Scalon et al. 2022). *Gwilymia* differs from other genera of the Stryphnodendron clade by the following set of traits: leaves with 2–4 (6) pairs of pinnae (vs. 1–2 (–3) or ﻿(3–) 5–32), leaflets relatively large (2.5–16 × 1.5–8 cm vs. 0.6–5 × 0.3–2.5), spikes clustered in panicles [except in *G.coriacea* (Benth.) A.G. Lima, Paula-Souza & Scalon and *G.fissurata* (E.M.O. Martins) A.G. Lima, Paula-Souza & Scalon; vs. simple thyrses], and fruit an indehiscent nucoid legume (vs. follicles or legumes). Indehiscent fruits are also common in *Stryphnodendron*, but species of that genus have alternate leaflets.

#### Taxonomic references.

Caccavari (2002); Lima et al. (2022); Simon et al. (2016); Scalon et al. (2022).

### ﻿ Microlobius

Taxon classificationPlantaeFabalesFabaceae

C. Presl, Abh. Königl. Böhm. Ges. Wiss. ser. 5, 3: 496. 1845.

**Figure 175.**
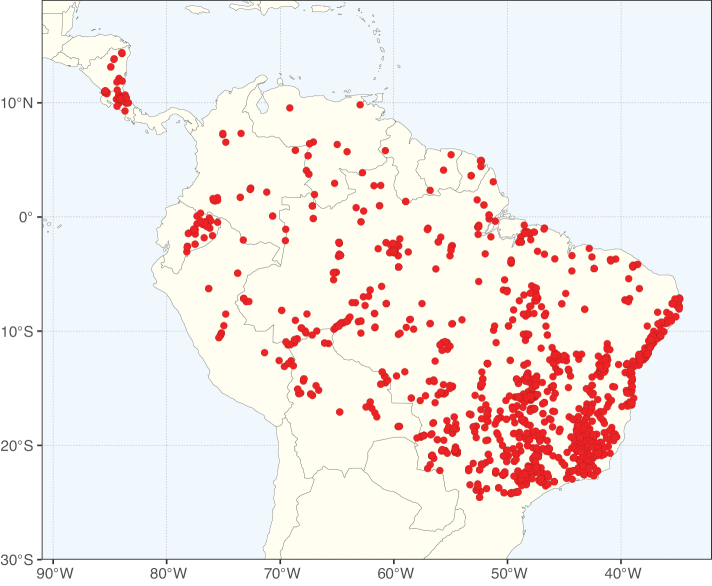
Distribution of *Microlobius* based on quality-controlled digitised herbarium records. See Suppl. material 1 for the source of occurrence data.

Figs 172 , 173 , 175

Goldmania Rose ex Micheli, Mém. Soc. Phys. Genève 34: 274. 1903. Type: Goldmaniaplatycarpa Rose [= Microlobiusfoetidus (Jacq.) M. Sousa & G. Andrade]

#### Type.

*Microlobiusmimosoides* C. Presl [= *Microlobiusfoetidus* (Jacq.) M. Sousa & G. Andrade]

#### Description.

Trees or shrubs, indumentum composed of simple and granular trichomes, brachyblasts present; branches and leaves unarmed, with a strong garlic odour. **Stipules** caducous. **Leaves** bipinnate, extrafloral nectaries between pinnae pairs, and sometimes also between leaflets, but never on the petiole; pinnae 1–2 (3) pairs, opposite; leaflets 1–2 pairs, opposite, obovate or elliptic, with or without a tuft of trichomes at the base of the abaxial surface. **Inflorescence units** cylindrical spikes, arranged in fascicles of 2–5 spikes in pseudoracemes. **Flowers** 5-merous, white to yellow; calyx gamosepalous, campanulate; corolla gamopetalous, narrowly campanulate; stamens 10, anthers with an apical gland; pollen in 8-grained polyads; ovary included in the corolla. **Fruit** a follicle, subfalcate; valves coriaceous. **Seeds** obovate, wingless, white, pleurogram present.

#### Chromosome number.

2*n* = 26 (Goldblatt 1981a).

#### Included species and geographic distribution.

Monospecific (*M.foetidus*), with two varieties. Microlobiusfoetidusvar.foetidus occurs in Mexico, Honduras and Venezuela, and M.foetidusvar.paraguensis (Benth.) M. Sousa & G. Andrade is restricted to Bolivia, Paraguay, Brazil and Argentina (Fig. 175).

#### Ecology.

Seasonally dry tropical forests and woodlands.

#### Etymology.

From Greek, *micro* (= small) and *lobos* (= pods), in reference to the small lignified ovaries present in the original material, which were thought to be mature fruits (Sousa and Andrade 1992).

#### Human uses.

Unknown.

#### Notes.

The status of *Microlobius* as a distinct genus has been questioned since its description (as *Goldmania*), particularly in comparison to the original, more ample circumscription of *Piptadenia* (Sousa and Andrade 1992; Mimosa clade, page 332). Phylogenetic evidence reinforced such doubts, indicating that *Microlobius* could be synonymised under *Stryphnodendron* (Simon et al. 2016; Lima et al. 2022). Notwithstanding, morphological and ecological evidence, as well as diagnosability, supported the dismembering of *Stryphnodendron* into distinct genera and the maintenance of *Microlobius* as a monospecific genus (Lima et al. 2022).

Among members of the Stryphnodendron clade, *Microlobius* is diagnosed by the following combination of characters: presence of garlic odour in the wood and leaves, petioles without extrafloral nectaries, leaves with a single pair of pinnae, opposite leaflets, and fruits follicles with white seeds (Lima et al. 2022).

#### Taxonomic references.

Burkart (1969); Sousa and Andrade (1992); Lima et al. (2022).

### ﻿ Stryphnodendron

Taxon classificationPlantaeFabalesFabaceae

Mart., Flora 20 (2 Beibl): 117. 1837.

**Figure 176.**
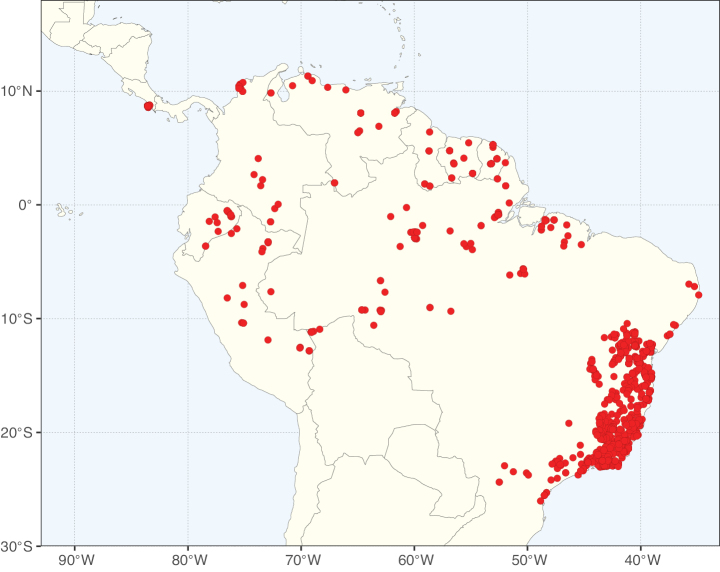
Distribution of *Stryphnodendron* based on quality-controlled digitised herbarium records. See Suppl. material 1 for the source of occurrence data.

Figs 172 , 173 , 176

Folianthera Raf., Sylva Tellur.: 120. 1838. Type: Foliantheraguianensis (Aubl.) Raf. [≡ Mimosaguianensis Aubl. (≡ Stryphnodendronguianense (Aubl.) Benth.)]

#### Type.

*Stryphnodendronbarbadetiman* (Vell.) Mart. [= *Stryphnodendronadstringens* (Mart.) Coville]

#### Description.

Trees, shrubs, or geoxyle subshrubs; indumentum composed of simple and granular trichomes; brachyblasts absent; branches unarmed, young shoots and leaves ferruginous, covered with reddish granular trichomes, not odoriferous. **Stipules** usually caducous. **Leaves** bipinnate; extrafloral nectaries present on the petiole, between or just below pinnae pairs and between or just below the distal pairs of leaflets; pinnae (3) 5–32 pairs, subopposite, opposite or rarely alternate; leaflets 8–20 pairs, alternate. **Inflorescence units** cylindrical spikes, arranged in fascicles of 2–6 spikes in pseudoracemes. **Flowers** 5-merous, whitish, yellowish or reddish; calyx gamosepalous, campanulate; corolla gamopetalous, narrowly campanulate; stamens 10, anthers with an apical gland; pollen usually in 16-grained polyads, but also in groups of 4–32 grains; ovary included in the corolla. **Fruit** an indehiscent legume or a follicle, oblong, linear or slightly curved; valves woody or coriaceous. **Seeds** obovoid or ellipsoid, wingless, black, brown, or ochre, pleurogram present.

#### Chromosome number.

2*n* = 26 (Bandel 1974; Santos et al. 2012).

#### Included species and geographic distribution.

Twenty-eight species in tropical Central and South America, from Nicaragua to southern Brazil (Fig. 176).

#### Ecology.

The majority of species occur either in rainforests (most in the Brazilian Amazon), or in savannas (Brazilian Cerrado), and four extend into seasonally dry tropical forests and woodlands (Lima et al. 2020b, 2021; Scalon et al. 2022).

#### Etymology.

From Greek, *stryphno* (= sour, adstringent) and *dendron* (= tree), in reference to the astringent properties of its bark.

#### Human uses.

*Stryphnodendronadstringens* is widely used due to its tannin-rich bark with astringent properties (Primo 1945) in leather tanning and, most importantly, as a medicine (Martius 1843; Rodrigues 1893; Santos et al. 2002; Brandão et al. 2008). The species has healing, antiseptic and anti-inflammatory properties (Panizza et al. 1988; Nascimento et al. 2013; Souza-Moreira et al. 2018).

#### Notes.

Phylogenetic and morphological evidence together with diagnosability supported the segregation of part of *Stryphnodendron* species into two new genera (*Gwilymia* and *Naiadendron*; Simon et al. 2016; Lima et al. 2022). In this context, *Stryphnodendron* became more coherent morphologically and includes only species bearing alternate leaflets with a tuft of trichomes at the base of the midrib on the abaxial surface, traits commonly used to diagnose the genus, but that do not occur in species now assigned to *Gwilimya* and *Naiadendron* (Simon et al. 2016; Lima et al. 2022; Scalon et al. 2022).

Among other members of the Stryphnodendron clade, the genus *Stryphnodendron* can be recognised by joint occurrence of ferruginous indumentum (densely covered with reddish granular trichomes) on young branches and leaves, relatively small (0.6–1.2 × 0.3–0.6 cm) and alternate leaflets, spikes arranged in pseudoracemes, and the fruit an indehiscent nucoid legume or a follicle.

#### Taxonomic references.

Lima et al. (2020b, 2021, 2022); Occhioni (1990); Occhioni-Martins (1974, 1975, 1981); Scalon et al. (2022).

### ﻿ Marlimorimia

Taxon classificationPlantaeFabalesFabaceae

L.P. Queiroz, L.M. Borges, Marc.F. Simon & P.G. Ribeiro, PhytoKeys 205: 252–253. 2022.

**Figure 177.**
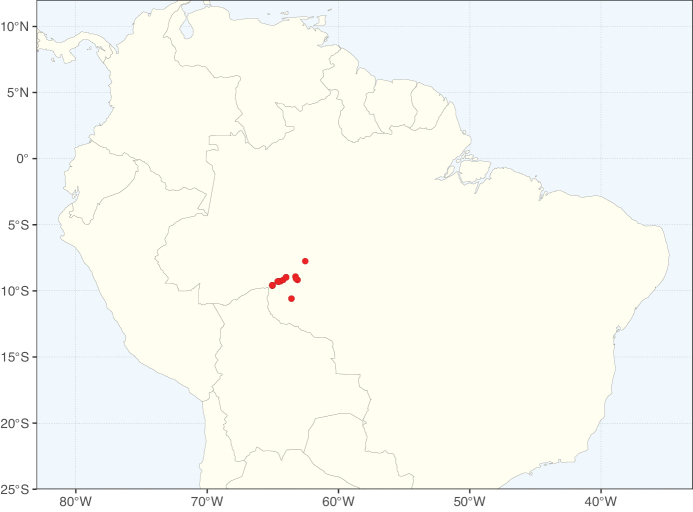
Distribution of *Marlimorimia* based on quality-controlled digitised herbarium records. See Suppl. material 1 for the source of occurrence data.

Figs 172 , 173 , 177

Newtonia sect. Neonewtonia Burkart, Fl. Il. Catarin. fasc. LEGU: 285. 1979. Type: Newtonianitida (Benth.) Brenan [≡ Piptadenianitida Benth. (= Marlimorimiacontorta (DC.) L.P. Queiroz & P.G. Ribeiro)]

#### Type.

*Marlimorimiacontorta* (DC.) L.P. Queiroz & P.G. Ribeiro [≡ *Acaciacontorta* DC.]

#### Description.

Trees; indumentum composed of simple trichomes; brachyblasts absent; branches and leaves unarmed, not odoriferous. **Stipules** caducous. **Leaves** bipinnate; extrafloral nectaries on the lower half of the petiole; pinnae (2) 5–many pairs, opposite; leaflets (6) 10–many pairs, opposite, mostly oblong to linear, rarely rhomboid. **Inflorescence units** cylindrical spikes, arranged in terminal pseudoracemes or clustered in efoliate nodes below the leaves. **Flowers** 5-merous, white to yellowish or greenish; calyx gamosepalous; corolla gamopetalous; stamens 10, anthers with an apical gland; pollen in polyads with 8, 12 or 16 grains; ovary included or exserted from the corolla. **Fruit** a follicle; margins straight, rarely sinuous and constricted where the seeds abort. **Seeds** flat compressed, dark, narrowly winged, pleurogram absent.

#### Chromosome number.

Unknown.

#### Included species and geographic distribution.

Six species, three in eastern Brazil, one in northern South America and Costa Rica, one endemic to Colombia, and one endemic to Venezuela (Fig. 177).

#### Ecology.

All species of *Marlimorimia* occur in rainforests.

#### Etymology.

*Marlimorimia* honours the Rio de Janeiro Botanical Garden botanist, Dr. Marli Pires Morim.

#### Human uses.

*Marlimorimiawarmingii* (Benth.) L.P. Queiroz & P.G. Ribeiro is used as firewood, timber, to make agricultural tools, and could be used as an ornamental and for ecological restoration (Burkart 1979; Carvalho 2008). Bark extracts of *M.contorta* have healing properties (Vieira et al. 2015).

#### Notes.

The genus was described to accommodate species that could not retain the name *Pseudopiptadenia* Rauschert, as it had to be synonymised under *Pityrocarpa* based on phylogenetic evidence (see below and Borges et al. 2022). Recognition of *Marlimorimia* is also supported by a suite of morphological features, such as extrafloral nectaries on the lower half of the petiole and spikes in fascicles arranged in a terminal pseudoraceme, or distributed in efoliate nodes below mature leaves.

Two species of the genus, *Marlimorimiacolombiana* (Britton & Killip) L.P. Queiroz & Marc.F. Simon, from Colombia, and *M.pittieri* (Harms) L.P. Queiroz & L.M. Borges, from Venezuela, are poorly known and have been assigned to the genus based on the morphology of type specimens only (Borges et al. 2022). Considering that morphological homoplasy is pervasive across mimosoid legumes, efforts should be made to expand knowledge on the distribution and morphological variation of these species, and to include them in molecular phylogenetic analyses.

#### Taxonomic references.

Barneby and Grimes (1984); Borges et al. (2022); Brenan (1955, 1963b); Lewis and Lima (1991); Lima (1985); Rauschert (1982).

### ﻿ Naiadendron

Taxon classificationPlantaeFabalesFabaceae

A.G. Lima, Paula-Souza & Scalon, PhytoKeys 205: 222. 2022.

**Figure 178.**
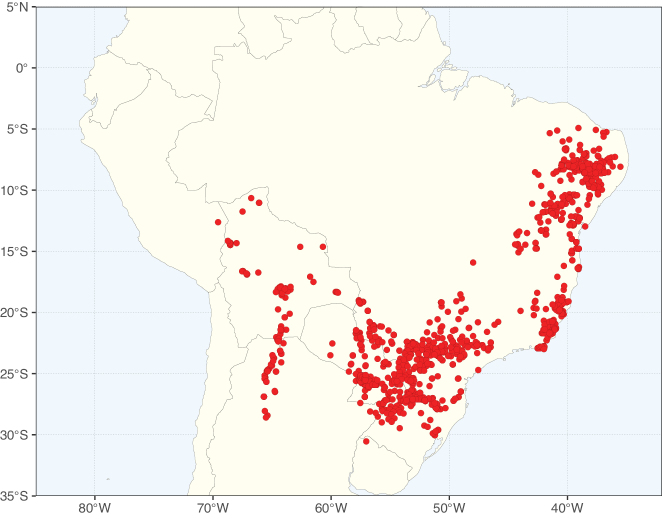
Distribution of *Naiadendron* based on quality-controlled digitised herbarium records. See Suppl. material 1 for the source of occurrence data.

Figs 172 , 173 , 178

#### Type.

*Naiadendronduckeanum* (Occhioni) A.G. Lima, Paula-Souza & Scalon [≡ *Stryphnodendronduckeanum* Occhioni]

#### Description.

Trees; indumentum composed of simple and granular trichomes; brachyblasts absent; branches unarmed, strongly striate, young shoots and leaves ferruginous with reddish granular trichomes, not odoriferous. **Stipules** caducous. **Leaves** bipinnate, not odoriferous; extrafloral nectaries on the petiole, rachis and pinnae; pinnae 10–22 pairs, subopposite to opposite; leaflets 15–23 pairs, opposite. **Inflorescence units** cylindrical spikes grouped in fascicles of 3–5 in pseudoracemes. **Flowers** 5-merous, white to yellowish; calyx gamosepalous; corolla gamopetalous; stamens 10, anthers with an apical gland; pollen in (12) 16-grained polyads; ovary included. **Fruit** a legume, dehiscent along both margins, linear to narrow-oblong, laterally-compressed; valves chartaceous. **Seeds** obovate or elliptic, wingless, ochre, pleurogram present.

#### Chromosome number.

Unknown.

#### Included species and geographic distribution.

Monospecific (*N.duckeanum*), from the northern Brazilian states of Acre, Amazonas and Rondônia (Fig. 178).

#### Ecology.

Rainforests (terra firme, often disturbed), on clay or sandy soil.

#### Etymology.

From *naiades*, Greek mythology’s nymphs of freshwater, and *dendron* (Greek = tree) in reference to the name given to the Brazilian Amazon (Naiades) by Carl Friedrich Philipp von Martius, where the single species of the genus occurs.

#### Human uses.

Unknown.

#### Notes.

Analysis of the extrafloral nectaries and fruits of *Stryphnodendronduckeanum*, initially described based on flowering material only, showed the species did not fit the limits of *Stryphnodendron* and that it could belong to *Piptadenia* (Rupert Barneby unpublished note; Scalon et al. 2022). Phylogenetic evidence reinforced segregation of the species from *Stryphnodendron* and showed it to be more closely related to *Parapiptadenia* and *Pityrocarpa*, thus supporting recognition of a distinct monospecific *Naiadendron* (Simon et al. 2016; Borges et al. 2022; Lima et al. 2022).

*Naiadendron* and *Parapiptadenia* are the only members of the Stryphnodendron clade with typical legume fruits, i.e., dehiscing along both margins. However, *Naiadendron* is readily set apart by the ferruginous indumentum covering both its young branches and leaves. Strongly striate branches and petiolar nectaries 8–12 mm long also differentiate *Naiadendron* from all other members of the clade.

#### Taxonomic references.

Lima et al. 2022; Scalon et al. 2022; Simon et al. 2016.

### ﻿ Parapiptadenia

Taxon classificationPlantaeFabalesFabaceae

Brenan, Kew Bull. 17(2): 228. 1963.

**Figure 179.**
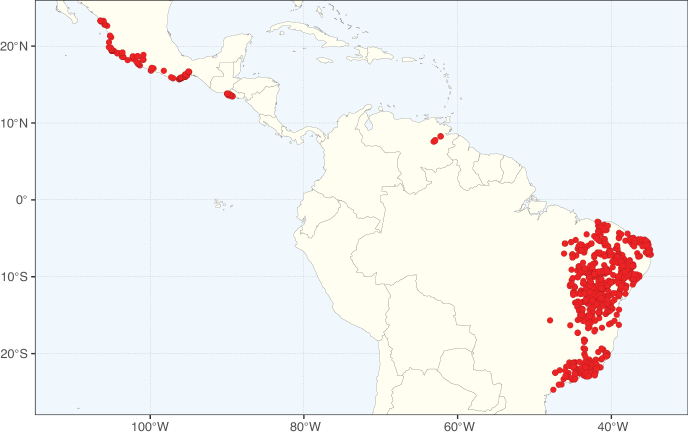
Distribution of *Parapiptadenia* based on quality-controlled digitised herbarium records. See Suppl. material 1 for the source of occurrence data.

Figs 172 , 173 , 179

#### Type.

*Parapiptadeniarigida* (Benth.) Brenan [≡ *Piptadeniarigida* Benth.]

#### Description.

Trees; indumentum composed of simple trichomes; brachyblasts absent; branches and leaves unarmed, not odoriferous. **Stipules** present or absent. **Leaves** bipinnate; extrafloral nectaries on the petiole, and usually between the distal pair of pinnae and leaflets; pinnae 1–8 pairs, opposite; leaflets (1) 2–26 pairs, opposite, elliptic to oblong. **Inflorescence units** cylindrical spikes, solitary, axillary or supra-axillary to coevally developing leaves. **Flowers** 5-merous, reddish, rarely yellowish; calyx gamosepalous, campanulate; corolla gamopetalous, campanulate to tubular; stamens 10, anthers with an apical gland; pollen in 8, 12 or 16-grained polyads; ovary included in the corolla. **Fruit** a legume, flat-compressed, valves undulate above the seeds (rarely plane). **Seeds** flat-compressed, dark, winged, pleurogram absent.

#### Chromosome number.

2*n* = 26 (Goldblatt 1981a).

#### Included species and geographic distribution.

Six species, four from north-eastern Brazil, one extending also to south-eastern Brazil; and two occurring in southern Brazil, Argentina, Paraguay, and Uruguay, one of which also reaches Bolivia, western Brazil and Peru (Fig. 179).

#### Ecology.

Sub-tropical forests, rainforests, seasonally dry tropical forests and woodlands.

#### Etymology.

From *para* (Greek = next to) and *piptadenia*, in reference to the close taxonomic relationship to the genus *Piptadenia* (Brenan 1963b).

#### Human uses.

*Parapiptadeniarigida* has medicinal properties, is used as fodder, timber, firewood, an ornamental, as a source of saponins, gum, tannins, cellulose, for paper production, and for ecological restoration (Burkart 1979; Carvalho 2003).

#### Notes.

In addition to *Anadenanthera* Speg., *Parapiptadenia* is the only segregate from *Piptadenia* that was confirmed as monophyletic (Jobson and Luckow 2007; Simon et al. 2016; Ribeiro et al. 2018; Borges et al. 2022). The genus commonly includes plants with reddish flowers, although these are yellow or cream in a few species [e.g., *Pa.rigida* (Benth.) Brenan], and legumes with undulate valves above the seeds. Most genera in the Stryphnodendron clade have yellow or cream inflorescences and either follicles or indehiscent fruits, except for *Stryphnodendron* and *Naiadendron*, which include some species with reddish inflorescences and legumes, respectively.

#### Taxonomic references.

Brenan (1963b); Caccavari (2002); Lewis (1993); Lima and Lima (1984); Vaz and Lima (1980).

### ﻿ Pityrocarpa

Taxon classificationPlantaeFabalesFabaceae

(Benth.) Britton & Rose, N. Amer. Fl. 23(3): 190. 1928.

**Figure 180.**
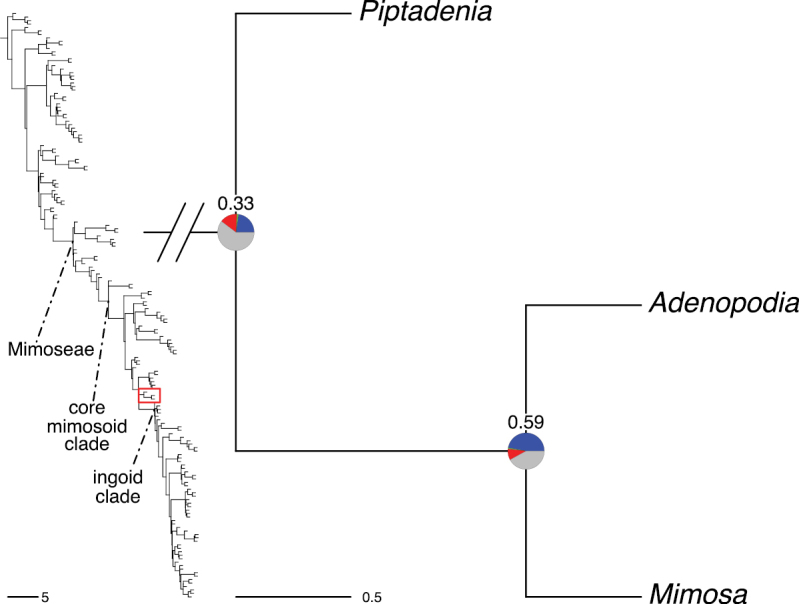
Distribution of *Pityrocarpa* based on quality-controlled digitised herbarium records. See Suppl. material 1 for the source of occurrence data.

Figs 172 , 173 , 180

Piptadenia sect. Pityrocarpa Benth., J. Bot. (Hooker) 4: 339. 1841. Type: Piptadeniamoniliformis Benth. [≡ Pityrocarpamoniliformis (Benth.) Luckow & R.W. Jobson]
Monoschisma Brenan, Kew Bull. 10(2): 179. 1955, nom. inval., non Monoschisma Duby, Mém. Soc. Phys. Genève 19: 294. 1868 (Musci, Meteoriaceae). Type: Monoschismaleptostachyum (Benth.) Brenan [≡ Piptadenialeptostachya Benth. (≡ Pityrocarpaleptostachya (Benth.) L.P. Queiroz & P.G. Ribeiro)]
Pseudopiptadenia Rauschert, Taxon 31(3): 559. 1982. Type: Pseudopiptadenialeptostachya (Benth.) Rauschert [≡ Piptadenialeptostachya Benth. (≡ Pityrocarpaleptostachya (Benth.) L.P. Queiroz & P.G. Ribeiro)]

#### Lectotype

**(designated by Britton and Rose 1928).***Pityrocarpamoniliformis* (Benth.) Luckow & R.W. Jobson [≡ *Piptadeniamoniliformis* Benth.]

#### Description.

Trees or shrubs; indumentum composed of simple trichomes; brachyblasts absent; branches and leaves unarmed, not odoriferous. **Stipules** present, sometimes caducous. **Leaves** bipinnate; extrafloral nectaries between or just below the first pair of pinnae; pinnae 1–4 (10) pairs, opposite; leaflets 1–10 (20) pairs per pinna, opposite, rhomboid, sometimes asymmetrically elliptical or lanceolate. **Inflorescences** cylindrical spikes, white to yellowish or greenish, solitary (rarely 2) in the axils of coevally developing leaves. **Flowers** 5-merous; calyx cupulate; petals free (sometimes joined in one species); stamens 10, anthers with an apical gland; pollen in polyads with 8 or 16 grains; ovary included or exserted from the corolla; stigma in a terminal pore. **Fruit** a follicle, flat compressed, margins deeply and regularly constricted, rarely sinuous and shallowly constricted. **Seeds** flat compressed, dark, narrowly winged, or rarely ovoid or discoid, white and wingless, pleurogram absent from dark seeds, but present in the white ones.

#### Chromosome number.

2*n* = 26 (Alves and Custódio 1989).

#### Included species and geographic distribution.

Seven species occurring in three major areas in tropical America: Mexico and Central America, northern South America (Guyana, Venezuela), and eastern Brazil (Fig. 180).

#### Ecology.

Rainforests (Brazil), seasonally dry tropical forests and woodlands (Brazil and Mexico), savannas (Venezuela) and Chaco (Paraguay).

#### Etymology.

From Greek, *pityron* (= scurf; husks of bran) and *carpus* (= fruit), in reference to the leprose fruits of some species (e.g., *Pityrocarpamoniliformis*).

#### Human uses.

*Pityrocarpamoniliformis* is used as fodder, timber for light constructions, firewood, and for soil enrichment, ecological restoration and honey production; it is also rich in tannins (Carvalho 2010; Carvalho 2014).

#### Notes.

Originally described as a section of *Piptadenia* (Bentham 1841b), *Pityrocarpa* included three species with leprose fruits (Britton and Rose 1928). The limits of the genus were briefly expanded due to nomenclatural mistakes (Brenan 1955), which, after being corrected, culminated in a return to its original circumscription and rank (Brenan 1963b). Although phylogenetic evidence provided renewed support for recognition of *Pityrocarpa* at the generic level (Jobson and Luckow 2007), more recent analyses recovered the genus as paraphyletic in relation to part of *Pseudopiptadenia*, including its type species (Simon et al. 2016; Borges et al. 2022). As *Pityrocarpa* has publication priority, *Pseudopiptadenia* was subsumed under it.

The recent updates to the circumscription of *Pityrocarpa* rendered the genus more variable with respect to seed morphology —a character previously used to distinguish it from *Parapiptadenia* and *Pseudopiptadenia*, for example— but also highlighted the taxonomic relevance of particular traits for recognition of the genus, such as extrafloral nectaries between or just below the first pair of pinnae and inflorescences in general solitary and axillary to coevally developing leaves (Borges et al. 2022). Another distinctive, although not exclusive, feature of *Pityrocarpa* is the deeply constricted, moniliform fruits (Bentham 1875; Simon et al. 2016; Borges et al. 2022).

#### Taxonomic references.

Borges et al. (2022); Jobson and Luckow (2007); Lewis and Lima (1991); Simon et al. (2016).

## ﻿﻿25. Mimosa clade

Leonardo M. Borges^5^, Marcelo F. Simon^40^, Matías Morales^33,34^, Melissa Luckow^29^, Pétala Gomes Ribeiro^2^, Rosaura Grether^19^

Citation: Borges LM, Simon MF, Morales M, Luckow M, Ribeiro PG, Grether R (2024) 25. Mimosa clade. In: Bruneau A, Queiroz LP, Ringelberg JJ (Eds) Advances in Legume Systematics 14. Classification of Caesalpinioideae. Part 2: Higher-level classification. PhytoKeys 240: 332–342. https://doi.org/10.3897/phytokeys.240.101716

**Mimosa clade**

Figs 181–187

**Included genera (3).***Adenopodia* C. Presl (7 species), *Mimosa* L. (615), *Piptadenia* Benth. (28).

**Description.** Trees, shrubs, herbs, geoxyles, and lianas; brachyblasts absent or sometimes present; indumentum composed of either simple, glandular, or complex multicellular trichomes, or a combination of them; branches armed or not with scattered, serial, or nodal prickles. **Stipules** present, in general caducous. **Leaves** bipinnate (rarely once pinnate or phyllodinous with transversely dilated leafstalks), rachis armed or not; extrafloral nectaries absent (most *Mimosa*) or present on the petiole, sometimes also on the rachis and pinnae; pinnae 1–many pairs, opposite, armed or not; leaflets 1–many pairs per pinna, sessile, mostly opposite, variable in size and shape. **Inflorescence** globose or ellipsoid capitula, or cylindrical racemes or spikes, solitary, fasciculate or organised in complex synflorescences, sometimes developing from short shoots on efoliate branches. **Flowers** 3–5 (6)-merous, haplo- or diplostemonous; anther glands absent (*Mimosa*) or present; pollen in tetrads or in polyads with 8, 12 or 16 grains; ovary linear to oblong or elliptic, indumentum variable, stigma generally in a terminal pore. **Fruit** a legume with entire valves, a craspedium (valves breaking up in monospermic articles leaving persistent margins), or an unjointed craspedium (valves remain entire after separating from the margins), variable in size, shape, indumentum and seed number. **Seeds** lenticular, elliptic to subspherical or rhomboid, wingless; pleurogram present.

**Geographic distribution.** Species of the Mimosa clade occur in almost all tropical and subtropical vegetation formations from the USA to Argentina, but are most diverse in the dry areas of the American tropics. Approximately 40 species of *Mimosa* are native to continental Africa, Madagascar and Asia, while four species of *Adenopodia* occur in Africa.

**Figure 181.**
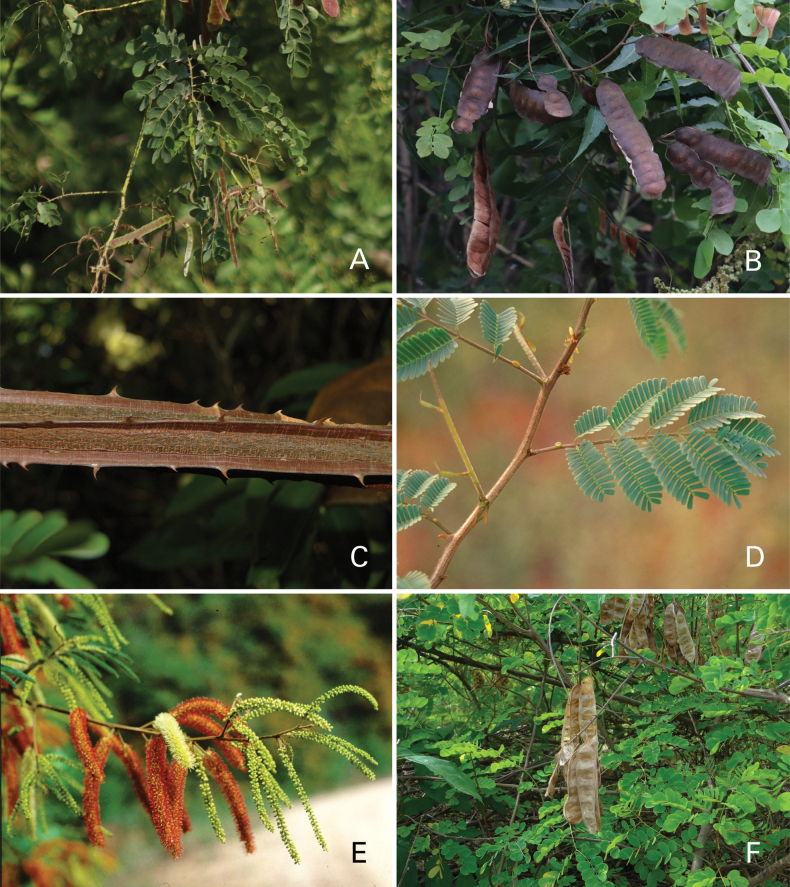
Generic relationships in the Mimosa clade (tribe Mimoseae). For description of phylogeny and support values, see Fig. 6 caption (page 63).

**Notes.** Members of the Mimosa clade were previously treated as part of the Piptadenia group, an informal assemblage of genera with haplo- or diplostemonous flowers, pollen in tetrads or polyads, and a narrowing style with a small porate stigma at the tip (Lewis and Elias 1981). Genera of this informal Piptadenia group have subsequently been shown to be phylogenetically dispersed along the backbone of the Mimoseae phylogeny spanning the Mimosa, Parkia and Stryphnodendron clades, as recognised here (Jobson and Luckow 2007; Simon et al. 2016; Koenen et al. 2020a; Borges et al. 2022; Lima et al. 2022; Ringelberg et al. 2022 ; Styphnodendron clade, page 319).

**Clade-based definition.** The most inclusive crown clade containing *Piptadeniaadiantoides* (Spreng.) J.F. Macbr. and *Mimosapudica* L., but not *Senegalianigrescens* (Oliv.) P.J.H. Hurter, *Stryphnodendronadstringens* (Mart.) Coville or *Lachesiodendronviridiflorum* (Kunth) P.G. Ribeiro, L.P. Queiroz & Luckow (Fig. 181).

All three genera of the Mimosa clade include plants armed with internodal prickles (although unarmed plants are common in *Mimosa*) with leaflets varying greatly in size and number and flowers arranged in spiciform racemes or spikes, even though globose capitula are common in *Mimosa*. *Mimosa* stands out from the other two genera by its anthers lacking apical glands; *Adenopodia* and *Mimosa* share fruits with valves separating from the persistent margins and breaking up into monospermic segments (a craspedium; in a few species of *Mimosa* the valves remain entire forming an unjointed craspedium, and in one species the fruits are lomentiform), while those of *Piptadenia* are typical legumes dehiscing along both sutures; some species also have non-pentamerous flowers (3-, 4- or more rarely 6-merous in *Mimosa*), and extrafloral nectaries are present across all three genera of the clade including in MimosasectionMimadenia Barneby, but subsequently lost across the vast majority of species of *Mimosa* (Barneby 1991; Simon et al. 2011).

Phylogenomic analyses show that *Adenopodia* is robustly supported as sister to *Mimosa* (Fig. 181), with which it is morphologically similar. In particular, the two genera share the craspedial fruit type, which is a diagnostic morphological synapomorphy for that sub-clade. *Adenopodia* differs from *Mimosa* by the presence of the apical anther glands (also found in *Piptadenia* but absent in *Mimosa*).

### ﻿ Piptadenia

Taxon classificationPlantaeFabalesFabaceae

Benth., J. Bot. (Hooker) 4: 334. 1841.

Figs 182 , 183

#### Lectotype.

*Piptadenialatifolia* Benth. [= *Piptadeniaadiantoides* (Spreng.) J.F. Macbr.]

#### Description.

Trees 3–30 m, shrubs or lianas; indumentum composed of simple trichomes, commonly absent; brachyblasts absent; branches armed with recurved or straight prickles, these scattered or aligned in raised ribs, rarely in groups of three at the leaf nodes. **Stipules** present, caducous. **Leaves** bipinnate, commonly with prickles on petioles and pinnae; extrafloral nectaries on the petiole, on the rachis between distal pairs of pinnae, and on the pinnae between distal pairs of leaflets; pinnae 1–14 pairs, opposite; leaflets 1–many pairs, opposite. **Inflorescences** cylindrical spikes, white, cream, yellow or red, isolated, in fascicles, or arranged in complex racemose or paniculate synflorescences. **Flowers** 5-merous, diplostemonous; calyx gamosepalous; corolla gamopetalous; stamens 10, anthers with a caducous apical claviform gland; pollen in tetrads or in polyads with 8, 12 or 16 grains; ovary glabrous to pubescent; stigma in a terminal pore. **Fruit** a flat compressed legume with thick margins, straight, usually papery. **Seeds** compressed, obovate to orbicular in outline, pleurogram present.

#### Chromosome number.

2*n* = 26 [*Piptadeniaretusa* (Jacq.) P.G. Ribeiro, Seigler & Ebinger; Alves and Custódio (1989), as *P.stipulacea* (Benth.) Ducke].

#### Included species and geographic distribution.

Twenty-eight species in tropical America, from western and southern Mexico to southern Brazil and perhaps also at the northern limits of Argentina (Fig. 183).

**Figure 182.**
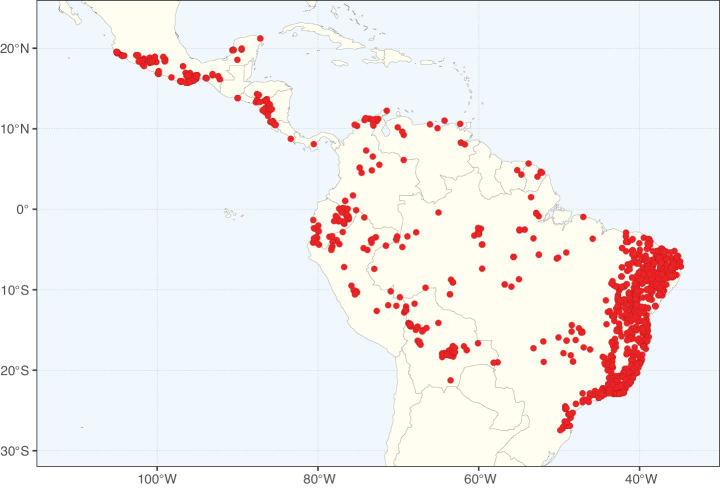
*Adenopodia* and *Piptadenia* diversity **A***Adenopodiagymnantha* Brenan fruiting branch **B** fruits **C***Piptadeniaramosissima* Benth., branch with prickles aligned along raised ribs **D***P.retusa* (Jacq.) P.G. Ribeiro, Seigler & Ebinger, vegetative branch **E, F***P.micracantha* Benth. **E** flowering branch **F** fruits. Photo credits **A, B** EOA Pérez **C–F** LP Queiroz.

**Figure 183.**
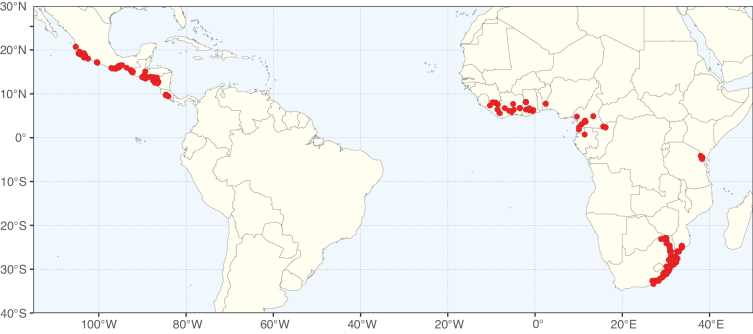
Distribution of *Piptadenia* based on quality-controlled digitised herbarium records. See Suppl. material 1 for the source of occurrence data.

#### Ecology.

Rainforests, seasonally dry tropical forests and woodlands, and riparian forests within Neotropical savannas.

#### Etymology.

From Greek, *pipto* (= to fall) and *aden* (= gland), in reference to the caducous anther glands.

#### Human uses.

Some species are used as a fodder, a source of tannins, as ornamentals, for timber, woodwork, firewood, paper and honey production, soil enrichment and ecological restoration (Carvalho 2014).

#### Notes.

*Piptadenia* was originally broadly circumscribed to accommodate mimosoid legumes with glandular anthers, and flat, dehiscent fruits with thin valves (Bentham 1841a; 1875). However, subsequent studies showed that this circumscription was both morphologically inaccurate and non-monophyletic. The fruits vary considerably in shape, type of dehiscence, and texture, and species once assigned to *Piptadenia* s.l. are placed in different groups scattered across the Mimoseae phylogeny (Brenan 1963b; Lima and Lima 1984; Lewis and Lima 1991; Jobson and Luckow 2007; Ribeiro et al. 2018). In this context, the circumscription of *Piptadenia* has been narrowed by transferring species to *Anadenanthera* Speg., *Indopiptadenia* Brenan, *Lachesiodendron* P.G. Ribeiro, L.P. Queiroz & Luckow, *Microlobius* C. Presl, *Newtonia* Baill., *Parapiptadenia* Brenan, *Piptadeniastrum* Brenan, *Pityrocarpa* (Benth.) Britton & Rose, and *Marlimorimia* L.P. Queiroz, L.M. Borges, Marc.F. Simon & P.G. Ribeiro (Brenan 1963b; Lima and Lima 1984; Lewis and Lima 1991; Jobson and Luckow 2007; Ribeiro et al. 2018; Borges et al. 2022), and thereby restricting *Piptadenia* to 28 species in the New World.

Although a densely sampled phylogeny of *Piptadenia* is still pending, the genus can be diagnosed by the stems armed with recurved prickles, extrafloral nectaries on the petioles, cylindrical spikes, presence of anther glands, and flat, papery, straight legumes. Three species in the genus are known to nodulate (Faria et al. 2022).

#### Taxonomic references.

Brenan (1955, 1963b); Jobson and Luckow (2007); Ribeiro et al. (2018).

### ﻿ Adenopodia

Taxon classificationPlantaeFabalesFabaceae

C. Presl, Epimel. Bot.: 206. 1851.

Figs 182 , 184

Pseudoentada Britton & Rose, N. Amer. Fl. 23: 191. 1928. Type: Pseudoentadapatens (Hook. & Arn.) Britton & Rose [≡ Ingapatens Hook. & Arn. (≡ Adenopodiapatens (Hook. & Arn.) J.R. Dixon ex Brenan)]
Entada subg. Acanthentada Brenan, Kew Bull. 20: 366. 1966. Type: Entadaspicata (E. Mey.) Druce [≡ Mimosaspicata E. Mey. (≡ Adenopodiaspicata (E. Mey.) C. Presl)]

#### Type.

*Adenopodiaspicata* (E. Mey.) C. Presl [≡ *Mimosaspicata* E. Mey.]

#### Description.

Lianas, rarely shrubs or treelets; indumentum composed of simple trichomes; brachyblasts absent; branches armed with scattered, sometimes paired, recurved prickles. **Stipules** present, caducous or persistent. **Leaves** bipinnate, often with prickles on petioles and pinnae; extrafloral nectaries on the petiole and sometimes also between each pinnae pair; pinnae 1–many pairs, opposite; leaflets 1–many pairs, opposite. **Inflorescences** cylindrical spikes, white, yellow, pink or purple, often organised in panicles. **Flowers** 5-merous, diplostemonous; calyx gamosepalous; corolla polypetalous or gamopetalous; stamens 10, anthers with an apical gland; pollen in 16-grained polyads; ovary pubescent. **Fruit** a craspedium, straight or curved, sometimes armed along the margins. **Seeds** ellipsoid to spheroid; pleurogram present.

#### Chromosome number.

Unknown, indicated as possibly 2*n* = 28 by Lewis and Elias (1981) but more likely to be 2*n* = 26, as in the closely related *Mimosa* and *Piptadenia*.

#### Included species and geographic distribution.

**Figure 184.**
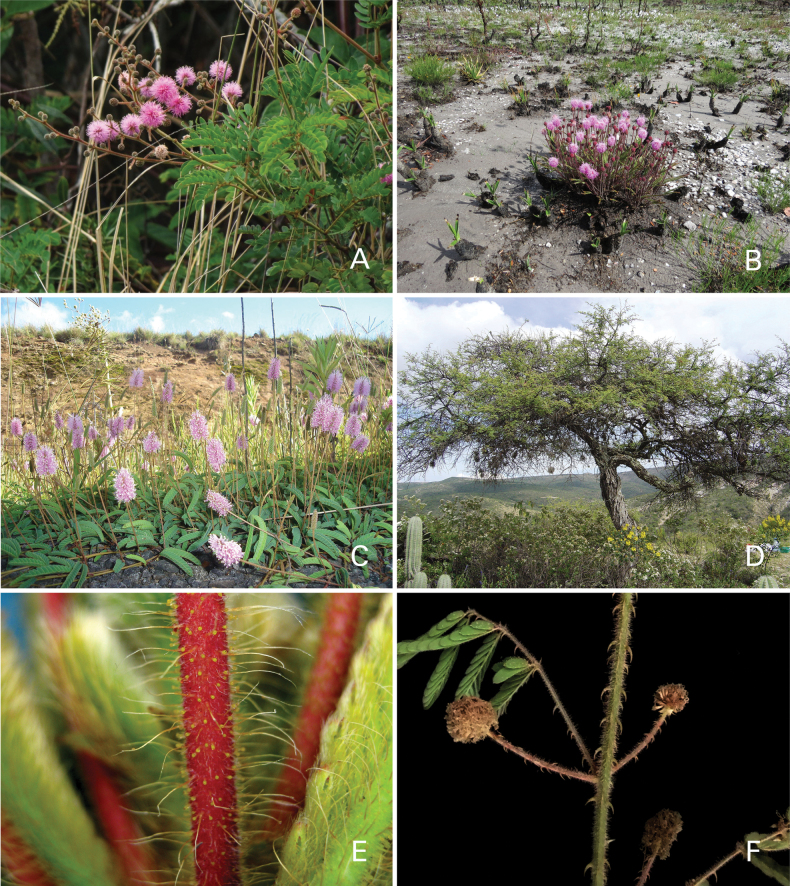
Distribution of *Adenopodia* based on quality-controlled digitised herbarium records. See Suppl. material 1 for the source of occurrence data.

Seven species (see notes below). Three species are restricted to Mexico and Central America, and four occur disjunctly across sub-Saharan Africa (Fig. 184).

#### Ecology.

In the New World confined to seasonally dry tropical forest, in Africa, on the margins of rain and deciduous forests, thickets and disturbed habitats.

#### Etymology.

From Greek, *adeno*- (= gland) and -*podia* (= foot), likely a reference to the shortly pedicellate anther glands.

#### Human uses.

*Adenopodiaspicata* has anti-hypertensive properties (Duncan et al. 1999) and in South Africa is used in spiritual rituals, and to treat lice infestation and respiratory illness (Sobiecki 2002; Mhlongo and Van Wyk 2019; Cock and Van Vuuren 2020).

#### Notes.

Brenan (1986) included 10 species in *Adenopodia*, of which three have valvately dehiscent legumes (not craspedia), and, thus, belong to *Piptadenia* as noted by Barneby (1986): *P.floribunda* Kleinhoonte, *P.minutiflora* Ducke, and *P.uaupensis* Spruce ex Benth. Only a few species of *Adenopodia* have been examined for nodulation and reports are mixed, with one species nodulating [*A.patens* (Hook. & Arn.) J.R. Dixon ex Brenan; Faria et al. 2022] and another not [*A.scelerata* (A. Chev.) Brenan; Diabate et al. 2005].

#### Taxonomic references.

Barneby (1986); Brenan (1986).

### ﻿ Mimosa

Taxon classificationPlantaeFabalesFabaceae

L., Sp. Pl. 1: 516. 1753.

**Figure 185.**
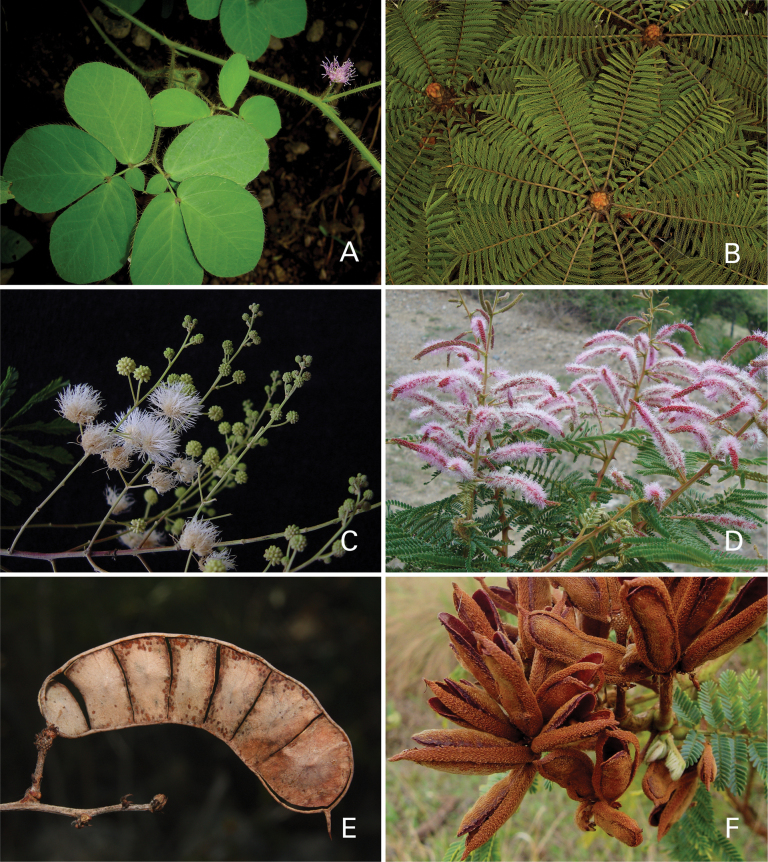
*Mimosa* vegetative diversity **A***M.dasilvae* A.S.L. Silva & Secco, a shrub **B***M.pumilio* Barneby, a geoxyle subshrub (*Simon 3187*) **C***M.dutrae* Malme, a trailing subshrub **D**M.texanavar.filipes (Britton & Rose) Barneby, a tree (*Simon 845*) **E***M.oedoclada* Barneby showing the indumentum composed by simple, glandular and setiform trichomes (*Simon 2588*) **F***M.supravisa* Barneby showing a branch armed with prickles (*Jordão 442*). Photo credits **A** CO Andrino **B, D–F** MF Simon **C** N Dahmer.

**Figure 186.**
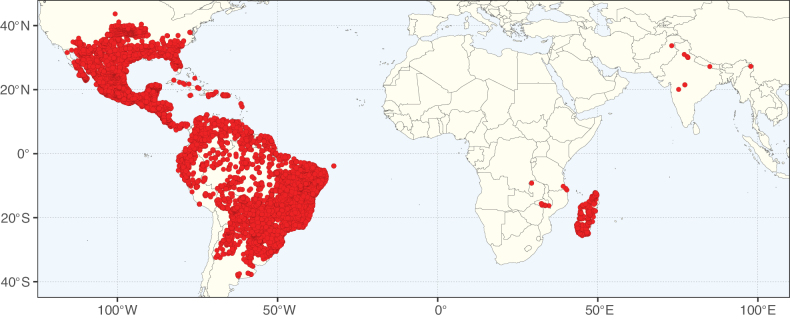
*Mimosa* leaf, inflorescence and fruit diversity **A***M.tequilana* S. Watson showing bipinnate leaves with one pair of pinnae and two pairs of broad leaflets per pinna (*Simon 813*) **B***M.splendida* Barneby showing multi-pinnate leaves with many small leaflets (*Simon 739*) **C***M.bimucronata* (DC.) Kuntze showing capitate inflorescences (*Simon 872*) **D***M.benthamii* J.F. Macbr. showing cylindrical inflorescences **E***M.irrigua* Barneby showing a craspedium **F***M.foliolosa* Benth. showing many unjointed craspedia. Photo credits **A–C, F** MF Simon **D** R Grether **E** D Cardoso.

**Figure 187.**
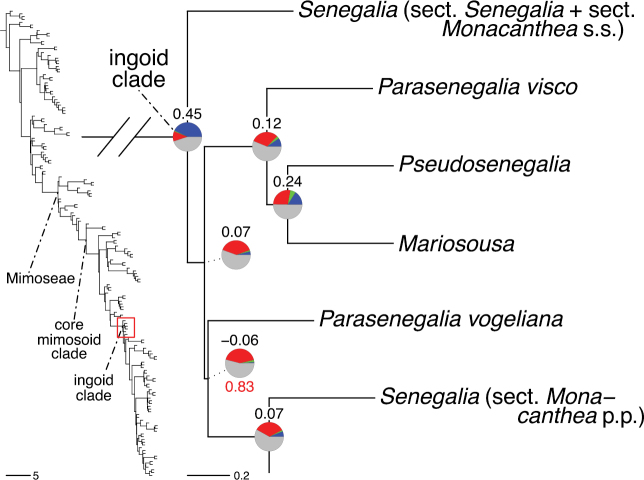
Distribution of *Mimosa* based on quality-controlled digitised herbarium records. See Suppl. material 1 for the source of occurrence data.

Figs 185 , 186 , 187

Schrankia Willd., Sp. Pl., ed. 4(2): 888, 1041. 1806, nom. cons. Lectotype: Schrankiaaculeata Willd. [= Mimosaquadrivalvis L.]
Eburnax Raf., New Fl. [Rafinesque] 1: 42. 1836. Type: Eburnaxpudica (L.) Raf. [≡ Mimosapudica L.]
Sensitiva Raf., Sylva Tellur.: 119. 1838. Type: Sensitivapudica Raf. [≡ Mimosapudica L. ?]
Lomoplis Raf., Sylva Tellur.: 118. 1838. Lectotype (designated by Britton and Rose 1928): Lomoplisceratonia (L.) Raf. [≡ Mimosaceratonia L.]
Leptoglottis DC. ex Torr. & A. Gray, Fl. N. Amer. (Torr. & A. Gray) 1: 695. 1840. Type: Leptoglottisnuttallii DC. ex Torr. & A. Gray [≡ Mimosaquadrivalvisvar.nuttallii (DC. ex Torr. & A. Gray) Beard ex Barneby]
Morongia Britton, Mem. Torrey Bot. Club 5: 191. 1894. Lectotype (designated here): Morongiaangustata (Torr. & A. Gray) Britton [≡ Mimosaquadrivalvisvar.angustata (Torr. & A. Gray) Barneby]
Schranckiastrum Hassl., Repert. Spec. Nov. Regni Veg. 16: 151. 1919. Type: Schranckiastruminsigne Hassl. [≡ Mimosainsignis (Hassl.) Barneby]
Acanthopteron Britton, N. Amer. Fl. 23: 179. 1928. Type: Acanthopteronlaceratum (Rose) Britton [≡ Mimosalacerata Rose]
Haitimimosa Britton, N. Amer. Fl. 23: 179. 1928. Type: Haitimimosaextranea (Benth.) Britton [≡ Mimosaextranea Benth.]
Mimosopsis Britton & Rose, N. Amer. Fl. 23: 174. 1928. Type: Mimosopsisprolifica (S. Watson) Britton & Rose [≡ Mimosaprolifica S. Watson]
Neomimosa Britton & Rose, N. Amer. Fl. 23: 172. 1928. Type: Neomimosaeurycarpa (B.L. Rob.) Britton & Rose [≡ Mimosaeurycarpa B.L. Rob.]
Pteromimosa Britton, N. Amer. Fl. 23: 171. 1928. Type: Pteromimosabahamensis (Benth.) Britton [≡ Mimosabahamensis Benth.]

#### Lectotype.

*Mimosasensitiva* L.

#### Description.

Perennial shrubs, herbs, geoxyle subshrubs, lianas, treelets and trees, occasionally monocarpic; indumentum composed of numerous different combinations of simple, glandular, and complex multicellular trichomes and sharp-pointed setae; brachyblasts sometimes present; branches armed or not with scattered, serial, or nodal prickles. **Stipules** present, caducous or persistent. **Leaves** bipinnate, rarely once-pinnate (*M.unipinnata* B.D. Parfitt & Pinkava), sometimes reduced to phyllodes bearing ephemeral pinnae (*M.ephedroides* (Gillies ex Hook. & Arn.) Benth., *M.extranea* Benth., *M.phyllodinea* Benth.), rachis armed or not, sometimes with a spicular projection between pinnae pairs; extrafloral nectaries absent in the vast majority of species, if present, on the petiole and sometimes also on the rachis and pinnae; pinnae 1–many pairs, opposite, armed or not; leaflets 1–many pairs per pinna, sessile, mostly opposite, variable in size and shape; paraphyllidia mostly present. **Inflorescences** globose or ellipsoid capitula, cylindrical spikes or racemes, white, pink, yellow or red, solitary or fasciculate in the axils of coevally developing leaves, or organised in complex efoliate synflorescences, sometimes developing from short shoots on efoliate branches. **Flowers** 3–5 (6)-merous, haplo- or diplostemonous; bisexual or staminate, the latter located at the lower parts of the inflorescence, rarely all flowers bisexual; calyx gamosepalous; corolla gamopetalous; anther glands absent; pollen in tetrads or polyads with 8, 12, or rarely 16 grains; ovary glabrous to lanose. **Fruit** a typical craspedium or an unjointed craspedium, rarely lomentiform or follicular, variable in size, shape, indumentum, armature and seed number. **Seeds** lenticular, ellipsoid to spheroidal or rhombic.

#### Chromosome number.

The basic chromosome number in *Mimosa* is reported to be *x* = 13 (Isely 1971; Goldblatt 1981b), with varying sporophytic chromosome counts recorded as 2*n* = 26, 2*n* = 39, 2*n* = 52, 2*n* = 104 (Seijo 1993, 1999, 2000; Seijo and Fernández 2001; Morales et al. 2010, 2011, 2014a, 2014b, 2015, 2018; Dahmer et al. 2011), and approximately 20% of species potentially polyploid (Dahmer et al. 2011).

#### Included species and geographic distribution.

615 species. Most are local endemics distributed right across the Americas from the USA to Argentina, with ca. 40 occurring in Africa, Madagascar (32 endemics) and Asia (Fig. 187). A few species of American origin (e.g., *Mimosapigra* L.) are introduced pantropically and highly invasive around the globe.

#### Ecology.

*Mimosa* is one of the most ecologically adaptable, widespread and ubiquitous genera of Mimoseae, occurring across the full tropical rainfall gradient and in almost all tropical and subtropical vegetation types in the Americas, including seasonally dry tropical and subtropical forests and semi-arid thorn scrub, desert, tropical and subtropical savannas and grasslands, lowland tropical rainforest, mid-elevation moist subtropical forest, wetlands, temperate forests, rarely on sand dunes, and often in disturbed open habitats. Some species become weedy outside their native range. This ecological adaptability is closely correlated with the diversity of growth forms - lianas in wet forests, stiffly-branched xerophytic shrubs in arid and semi-arid ecologies, and geoxyles in savannas (Barneby 1991). *Mimosa* is especially abundant, diverse and ecologically important in the Cerrado (with 170 endemics), this facilitated by multiple independent evolutionary adaptations to fire, including numerous functionally herbaceous geoxyles with underground lignotubers, and pachycaul rosette trees with naked trunks (Simon et al. 2009; Simon and Pennington 2012), and rapid recent species diversification within some of these fire-adapted clades (Koenen et al. 2013).

#### Etymology.

Either from Spanish, mimoso (= sensitive; Barneby 1991), or from Greek, mimos (= actor, mimicking; Grether 2023), in reference to the responsiveness of the leaflets and pinnae to touch (seismonasty) in some species.

#### Human uses.

*Mimosa* species are used as living fences (e.g., *M.caesalpiniifolia* Benth. in Brazil), timber (e.g., *M.scabrella* Benth. in southern Brazil and Argentina), fodder (e.g., *M.strigillosa* Torr. & A. Gray, in Argentina and the USA; Fernández et al. 1988; Alison et al. 2022), ornamentals, medicinal (Burkart 1948), firewood, and for soil enrichment and ecological restoration. Many species are a source of nectar for honey production, while some species with sensitive leaves are sold as “pet” plants (e.g., *M.pudica*).

#### Notes.

*Mimosa* is classified into a complex infrageneric and infraspecific hierarchy (five sections, 41 series, 39 subseries, 18 subspecies and 268 varieties; Barneby 1991). However, almost all of Barneby’s traditional sections and series are not monophyletic (Bessega et al. 2008; Simon et al. 2011) and a new phylogenetically-based classification is needed. Furthermore, the often complex patterns of morphological variation associated with some clades and widespread species alliances, likely further complicated by extensive polyploidy, which prompted Barneby’s extensive reliance on infraspecific taxa, are the focus of detailed studies of particular species complexes (e.g., Grether 2000; Morales and Fortunato 2010; Morales et al. 2010, 2018; Léon de la Luz et al. 2015; Borges et al. 2017, 2023; Jordão et al. 2018). The re-assessment of taxon delimitations within species complexes in this group has often resulted in the recognition at the specific rank of taxa that were treated by Barneby as subspecies and varieties.

Most species nodulate, particularly with beta-rhizobia (Reis Junior et al. 2010; Bontemps et al. 2016; Platero et al. 2016). Pollinators are unknown for the majority of species but include bees, bats, butterflies, dipterans, hemipterans and wasps (Vogel et al. 2005). Although most species do not show specialised dispersal mechanisms, species such as *M.pigra*, a globally widespread and wetland weed, have floating one-seeded articles highly adapted to water dispersal. The presence of setae and prickles on articles of *M.diplotricha* C. Wright, *M.sensitiva*, *M.skinnerii* Benth., *M.velloziana* Mart. and *M.ursina* Mart. indicates adaptation for animal dispersal (epizoochory).

Rapid movement of leaves and leaf parts in response to touch is a trademark of *Mimosa*. Although leaf movement evolved in many lineages within the genus, it is restricted to less than 5% of the species (Simon et al. 2011). The mechanism of leaf movement in the sensitive plant (*M.pudica*) has been studied in detail and seems to be associated with protection from insect attacks (Hagihara et al. 2022).

The highly disjunct amphi-Atlantic distribution of *Mimosa* (Fig. 187), and extremely uneven distribution of species diversity across this range are striking. This diversity anomaly with high species richness in the Neotropics and Madagascar, where the genus has diversified extensively, and Africa / Asia with just a handful of species, is as yet largely unexplained. However, it is clear that the Old World species form a relatively young clade (late Miocene) that is deeply nested within New World *Mimosa* (Simon et al. 2011), and that the Madagascan species also form a clade within the Old World clade (Simon et al. 2011) that shows a signature of a rapid evolutionary radiation (Ringelberg et al. 2023).

We could not confirm the type species of *Sensitiva*, one of the many generic synonyms of *Mimosa*. Although *Sensitiva* was validly published and circumscribed to include species close to *M.pudica* and *M.sensitiva*, Rafinesque (1838) did not make any new combinations under it.

#### Taxonomic references.

Barneby (1991); Bessega and Fortunato (2011); Bessega et al. (2008); Borges et al. (2023); Burkart (1948); Dutra et al. (2020); Grether (1987; 2000; 2023); Grether et al. (1996); Jordão et al. (2020); Medina-Acosta et al. (2019); Morales and Fortunato (2010); Morales et al. (2010, 2018); Santos-Silva et al. (2015); Simon et al. (2011); Villiers (2002).

## ﻿﻿26. Senegalia grade

Bruce R. Maslin^31,32^, Vanessa Terra^41^, David S. Seigler^38^, John E. Ebinger^14^, Colin E. Hughes^3^

Citation: Maslin BR, Terra V, Seigler DS, Ebinger JE, Hughes CE (2024) 26. Senegalia grade. In: Bruneau A, Queiroz LP, Ringelberg JJ (Eds) Advances in Legume Systematics 14. Classification of Caesalpinioideae. Part 2: Higher-level classification. PhytoKeys 240: 343–357. https://doi.org/10.3897/phytokeys.240.101716

**Senegalia grade**

Figs 188–195

**Included genera (4).***Mariosousa* Seigler & Ebinger (14 species), *Parasenegalia* Seigler & Ebinger (11), *Pseudosenegalia* Seigler & Ebinger (2), *Senegalia* Raf. (219).

**Distribution.** Pantropical; all four genera occur in the New World, but *Senegalia* extends to Africa, Asia and Australia.

**Figure 188.**
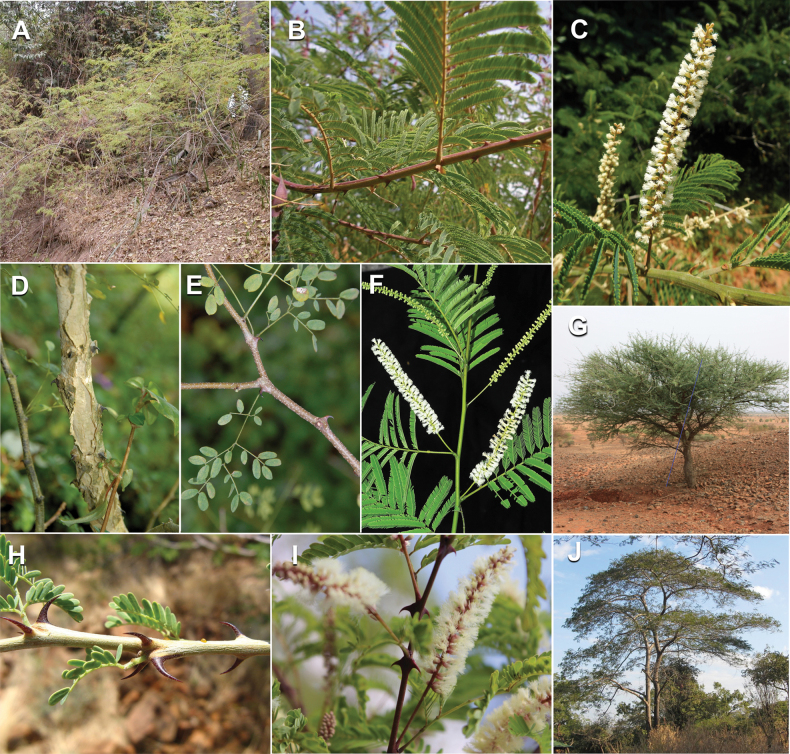
Generic relationships in the Senegalia grade (tribe Mimoseae). For description of phylogeny and support values, see Fig. 6 caption (page 63).

**Notes.** Species of the four Senegalia grade genera, together with species of *Acacia* Mill., *Acaciella* Britton & Rose and *Vachellia* Wight & Arn., were formerly included in what was previously called *Acacia* s.l. Recent phylogenomic analyses have confirmed that, as broadly circumscribed, *Acacia* s.l. is polyphyletic (Koenen et al. 2020a) as had been found in earlier studies (e.g., Luckow et al. 2003; Miller and Seigler 2012). *Acacia* is now assigned to the Archidendron clade (Brown et al. 2022; page 404), *Acaciella* to the Calliandra clade (page 358), *Vachellia* to the Parkia clade (page 299); and the remaining four genera, which form a grade subtending the ingoid clade, are here referred to as the Senegalia grade (Fig. 188).

A number of morphological characters, typical of all (or most) species of the four Senegalia grade genera, help distinguish these genera from the other three that were originally included in *Acacia* s.l. These include the absence of stipular spines, the bipinnate leaves having glands on the petiole and/or rachis (although occasionally absent from some individual plants), peduncles lacking a multi-bracteate involucre, flowers commonly sessile or subsessile (but if pedicellate, the pedicel not persistent on receptacle after flowers have dropped), and pollen (unknown for *Pseudosenegalia*) comprising 16-grained polyads, the grains porate, and lacking pseudocolpi and having a granular exine (i.e., lacking columellae). For further discussion on the morphological characters of *Acacia*, *Acaciella*, and *Vachellia* see their respective treatments in the Archidendron clade (page 404), Calliandra clade (page 358), and Parkia clade (page 299).

For several years prior to fragmentation *Acacia* s.l., many species that are now included in genera of the Senegalia grade had been assigned to Acaciasubg.Aculeiferum Vassal by various authors (e.g., Vassal 1972; Ross 1979; Nielsen 1981b, 1985a, 1985b, 1992). Sixteen species of *Senegalia* were included in Vassal’s (1972) study, but none from *Mariosousa*, *Parasenegalia*, or *Pseudosenegalia*. Further discussion of these matters is provided under the *Acacia* treatment in the Archidendron clade (page 404).

Over the following two decades, compelling evidence, especially from phylogenetic studies, confirmed that *Acacia* s.l. was polyphyletic and could not be sustained as a single genus (e.g., Luckow et al. 2003; Miller and Seigler 2012). *Senegalia* was subsequently reinstated and, based on a combination of molecular phylogenetic and morphological evidence, four small New World genera were segregated from it (or from Acaciasubg.Aculeiferum). These four genera are most obviously distinguished from *Senegalia* by their lack of armature, although prickles are also occasionally absent from some individual specimens of *Senegalia*. Firstly, Rico Arce and Bachman (2006) resurrected *Acaciella* (= Senegaliasect.Filicinae), a small but distinctive genus of 15 species (now included in the Calliandra clade). In that same year Seigler et al. (2006b) described *Mariousousa*, a similarly small genus that had previously been recognised as the informal ‘*Acaciacoulteri* group’ (e.g., Seigler et al. 2006b). About a decade later, two additional small New World genera, *Pseudosenegalia* and *Parasenegalia* (previously recognised by Miller and Seigler (2012) as the informal ‘*Acaciaskleroxyla* group’), were segregated from *Senegalia* in Seigler et al. (2017).

*Pseudosenegalia* was shown to be monophyletic and sister to a subclade containing *Mariosousa* and *Parasenegalia* by Miller et al. (2017) in a study based on plastid and nuclear sequence data. *Parasenegalia* was also shown to be monophyletic by Miller et al. (2017), but support for the genus was low. However, the phylogenomic study by Ringelberg et al. (2022) shows *Parasenegalia* to be non-monophyletic with *Albizialeonardii* Britton & Rose ex Barneby & J.W. Grimes [a synonym of *Parasenegaliavogeliana* (Steud.) Seigler & Ebinger - see Terra et al. 2022] placed separately from *P.visco* (Lorentz ex Griseb.) Seigler & Ebinger (see *Parasenegalia* treatment below for further discussion) (Fig. 188).

Despite the segregation of these four genera, the large, pantropical genus *Senegalia* is not always supported as monophyletic in recent phylogenomic studies (Koenen et al. 2020a; Ringelberg et al. 2022; Terra et al. 2022). The Senegalia grade comprising four genera represents one of the most perplexing and complex parts of the whole Caesalpinioideae phylogeny (Ringelberg et al. 2022; Terra et al. 2022). This complexity is caused by very short branches and high levels of gene tree conflict across the backbone of the grade, plus stark cytonuclear discordance which is relevant to the monophyly of *Senegalia*. This means that delimitation of these four genera and the relationships among them remain poorly resolved. Despite this lack of resolution, it is clear that *Senegalia* forms two separate robustly supported subclades in phylogenies derived from many nuclear genes (Suppl. materials 2, 3; Koenen et al. 2020a; Ringelberg et al. 2022), which contrasts with the plastid gene tree where *Senegalia* is resolved as monophyletic (Terra et al. 2022). Although it seems likely that *Senegalia* will need to be split into two (or perhaps three) genera, further phylogenomic work with denser taxon sampling is needed before this can proceed (Terra et al. 2022).

A key to the four genera of the Senegalia grade, plus *Acaciella*, is provided in Miller et al. (2017).

### ﻿ Senegalia

Taxon classificationPlantaeFabalesFabaceae

Raf., Sylva Tellur.: 119. 1838.

**Figure 189.**
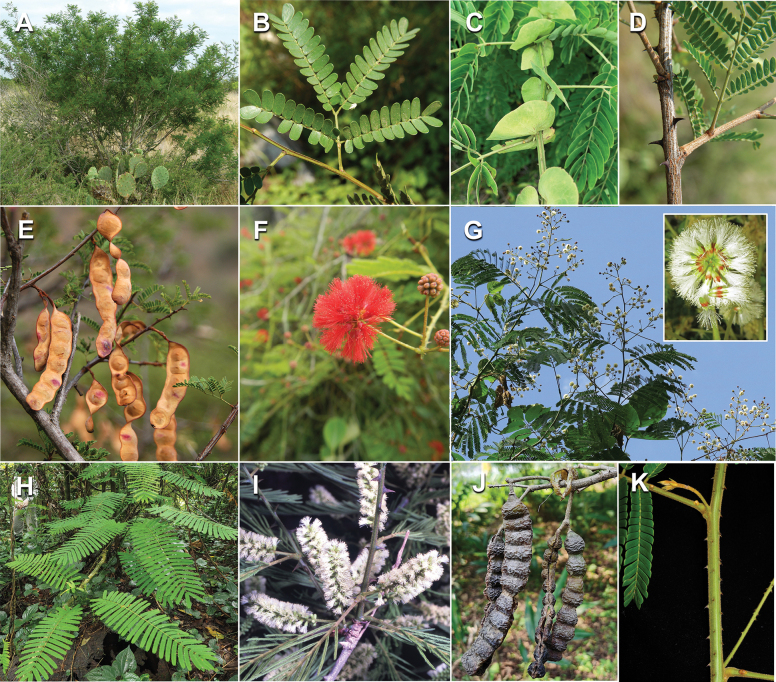
Morphological features of **A–C**Senegaliasect.Monacanthea s.s. and **D–J**Senegaliasect.Senegalia**A–C***Senegaliaataxacantha* (DC.) Kyal. & Boatwr. **A** lianescent shrub habit **B** branch showing internodal prickles **C** spicate inflorescence, Pretoria National Botanical Garden, South Africa **D, E***S.modesta* (Wall.) P.J.H. Hurter **D** papery peeling bark on branch, living collection at Singapore Botanic Gardens **E** branch showing two prickles at nodes and leaves with few pinnae, Asia **F***S.catechu* (L.f.) P.J.H. Hurter & Mabb. spicate inflorescence, South China Botanic Garden, Guangzhou **G***S.laeta* (R. Br. ex Benth.) Seigler & Ebinger habit **H***S.senegal* (L.) Britton three cauline prickles at nodes **I***S.goetzei* (Harms) Kyal. & Boatwr. two cauline prickles at nodes and spicate inflorescence **J**S.polyacanthasubsp.campylacantha (Hochst. ex A. Rich.) Kyal. and Boatwr. habit. Photo credits **A** P Birnbaum **B** S Piry **C** E Koenen **D, E** B Maslin **F** Y Chen **G** M Schmidt **H** A Dreyer **I** C Boucher Chisale **J** E Faust. **A, B, G–J** from African plants – A Photo Guide (www.africanplants.senckenberg.de).

**Figure 190.**
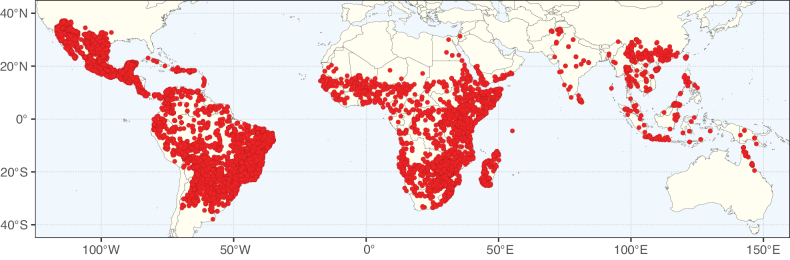
Morphological features of Senegaliasect.Monacanthea p.p. **A**Senegalia×emoryana (Benth.) Britton & Rose habit, New World **B***S.micrantha* (Benth.) Britton & Rose leaf, New World **C***S.grandistipula* (Benth.) Seigler & Ebinger large foliaceous stipules (such stipules not especially common in *Senegalia*) (*Terra 715*), New World **D**Senegalia×emoryana internodal prickles, New World **E***S.subsessilis* Britton & Rose thin-textured fruits, New World **F***S.sakalava* (Drake) Boatwr. globose scarlet inflorescence (scarlet flowers are rare in *Senegalia* but are found in several species from Madagascar) (*Koenen 215*), Madagascar **G***S.pruinescens* (Kurz) Maslin, Seigler & Ebinger lianescent shrub habit and terminal paniculate inflorescences (insert of head showing calyx red in upper part) (*Maslin 11023*), Asia **H**S.pennatasubsp.insuavis (Lace) Maslin, Seigler & Ebinger liana habit (*Maslin 11016*), Asia **I***S.menabeensis* (Villiers & Du Puy) Boatwr. spicate inflorescences (*Du Puy M359*), Madagascar **J***S.rugata* (Lam.) Britton & Rose hard-textured pods, Macau, China **K***S.tonkinensis* (I.C. Nielsen) Maslin, Seigler & Ebinger branch showing internodal prickles and two glands on petiole (uncommon in *Senegalia*) (*Maslin 11041*), Asia. Photo credits **A, B, D, E, H** B Maslin **C** V Terra **F** E Koenen **G, K** L Bai **I** D Du Puy **J** L-x Yuan.

**Figure 191.**
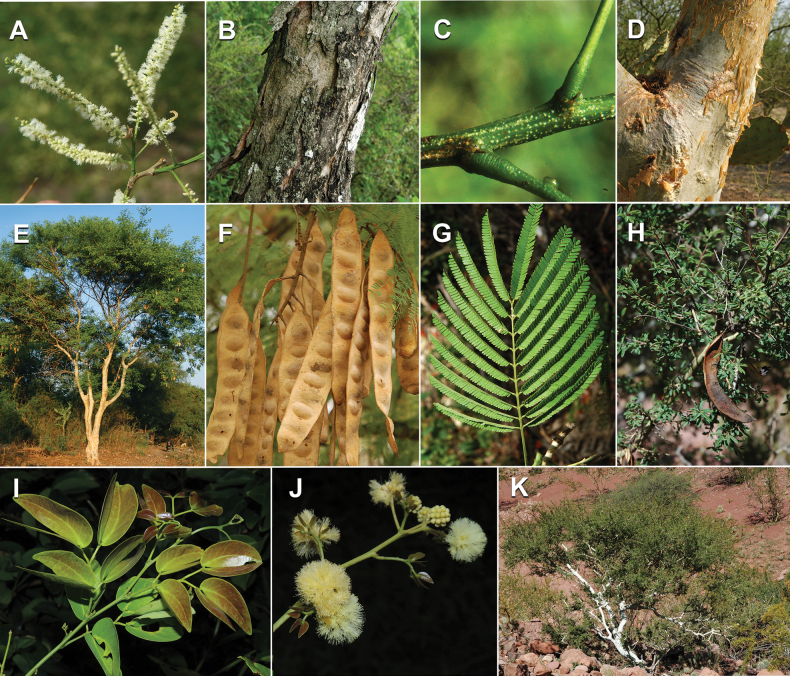
Distribution of *Senegalia* based on quality-controlled digitised herbarium records. Note that the Indian subcontinent was only sparsely sampled for this map. Details of species distribution, based on states of India and other countries of the subcontinent, are provided in Deshpande et al. (2019). See Suppl. material 1 for the source of occurrence data.

Figs 189 , 190 , 191

Manganaroa Speg., Bol. Acad. Nac. Ci. [Córdoba] 26(2): 227. [12 Oct.] 1922. Lectotype (designated by Pedley 1986): Manganaroamonacantha (Willd.) Speg. [≡ Acaciamonacantha Willd. (≡ Senegaliamonacantha (Willd.) Seigler & Ebinger)]
Dugandia Britton & Killip, Ann. New York Acad. Sci. 35(3): 137. 1936. Type: Dugandiarostrata (Humb. & Bonpl. ex Willd.) Britton & Killip [≡ Acaciarostrata Humb. & Bonpl. ex Willd. (≡ Senegaliarostrata (Humb. & Bonpl. ex Willd.) Seigler & Ebinger)]

#### Lectotype

**(designated by Britton and Rose, N. Amer. Fl. 23: 106. 1928).***Senegaliatriacantha* Raf., nom. illeg. [≡ *Mimosasenegal* L. (≡ *Senegaliasenegal* (L.) Britton)]. Note: Ross (1975c) did not typify *Senegalia* as stated by Pedley (1986), but he did select a neotype for *Mimosasenegal* L.

Two sections are currently recognised:

**SenegaliaRaf.sect.Senegalia**

Acaciasubg.Aculeiferum Vassal, Bull. Soc. Nat. Hist. Toulouse 108: 138. 1972; Vassal, Trav. Lab. Forest. Toulouse Tome 1, Vol. 8, Art. 17: 15. 1972. **Type**: *Acaciasenegal* (L.) Willd. [≡ *Mimosasenegal* L. (≡ *Senegaliasenegal* (L.) Britton]. Note: The subgenus was based on Acaciaser.Vulgares Benth., London J. Bot. 1: 322. 1842, not on Acaciaser.Vulgares and ser. Filicinae Benth. as given by Vassal (1981) in Polhill and Raven (1981: 170).

**Senegaliasect.Monacanthea (Vassal) Maslin, Pl. Diversity 41: 371. 2019.**

Acaciasubg.Aculeiferumsect.Monacanthea Vassal, Bull. Soc. Nat. Hist. Toulouse 108: 139. 1972; Vassal, Trav. Lab. Forest. Toulouse Tome 1, Vol. 8, Art. 17: 15. 1972. **Type**: *Acaciaataxacantha* DC. [≡ *Senegaliaataxacantha* (DC.) Kyal. & Boatwr.]

#### Description.

Armed (with prickles) lianas, shrubs or trees (rarely taller than ca. 12 m); bark usually grey to brown and hard, sometimes yellowish and papery or corky when young; brachyblasts sometimes present; prickles present on branchlets (1, 2 or 3 at leaf nodes, or scattered irregularly or in rows along internodes; sometimes absent from some individuals and/or herbarium specimens) and often on underside of petiole and/or rachis. **Stipules** usually small and caducous, infrequently large and ± persistent, not spinescent. **Leaves** bipinnate, not sensitive; extrafloral nectaries present on petiole and/or rachis (sessile or sometimes stipitate) and normally on the pinnae (sessile); pinnae 1–40 (60) pairs; paraphyllidia present or absent; leaflets opposite or rarely alternate [e.g., *S.comosa* (Gagnep.) Maslin, Seigler & Ebinger, sect. Monacanthea s.s.], petiolulate or sometimes sessile, (1) 3–80 (100) pairs per pinna. **Inflorescences** comprising pedunculate heads, spikes or rarely spiciform racemes that are solitary or in fascicles in leaf axils or arranged in compound racemes or panicles; peduncles lacking a multi-bracteate involucre. **Flowers** all hermaphrodite or sometimes staminate and hermaphrodite within the one inflorescence, uniform, 5-merous, with a basal nectariferous disk, sessile to sub-sessile or sometimes pedicellate, commonly white to cream, sometimes yellow or red; perianth connate, valvate, not scarious; stamens numerous (ca. 30+), free; anther glands present or absent; pollen normally comprising 16-grained polyads, normally porate and lacking pseudocolpi, exine surface psilate (often with circular depressions) or variously rugulate, exine lacking columellae (or rarely columellae very short); ovary sessile to stipitate. **Fruits** normally dehiscent, rarely indehiscent [e.g., *S.pentagona* (Schumach. & Thonn.) Kyal. & Boatwr., sect. Monacanthea p.p.] or breaking into 1-seeded articles (e.g., *S.rostrata*, *S.monacantha*, sect. Monacanthea p.p.), clearly flattened or rarely elliptic in cross-section, valves normally chartaceous to coriaceous, infrequently crustaceous to sub-woody [e.g., *S.rugata* (Lam.) Britton & Rose, sect. Monacanthea p.p.]. **Seeds** not winged, exarillate; pleurogram U-shaped (open at hilar end), occasionally circular or elliptic, rarely absent [e.g., *S.pedicellata* (Benth.) Seigler & Ebinger, sect. Monacanthea p.p.].

#### Chromosome number.

2*n* = 26 (known for 25 species, including those of sect. Senegalia and sect. Monacanthea p.p.), 2*n* = 39, 52 [*S.laeta* (R. Br. ex Benth.) Seigler & Ebinger, sect. Senegalia], 2*n* = 40 [*S.galpinii* (Burt Davy) Seigler & Ebinger, sect. Senegalia] and 2*n* = 26, 52 and 104 (*S.ataxacantha*, sect. Monacanthea s.s.) (Hamant et al. 1975; Goldblatt and Johnson 1979–; Ross 1979; Rice et al. 2015; all as *Acacia*).

#### Included species and geographic distribution.

A total of 219 species accommodated in three infrageneric groups (see Notes below): sect. Senegalia (51 species), sect. Monacanthea s.s. (four species) and sect. Monacanthea p.p. (164 species). The genus is distributed pantropically in the Americas (99 species), the African region (Africa and Madagascar, 68 species), Asia (Arabian Peninsula to East and South East Asia, 57 species) and north-east Australia (two species) (Fig. 191). Centres of species richness include Brazil (63 species), Mexico (30 species), East Asia (China, 22 species) and East Africa (e.g., Somalia, 21 species; Mozambique, 20 species). At the infrageneric level: sect. Senegalia occurs in Africa and Asia, sect. Monacanthea s.s. in Africa and sect. Monacanthea p.p. is pantropical, occurring in the Americas, Africa and Madagascar, Asia and north-east Australia. Distribution maps showing areas of species richness, and further details of species numbers are provided in Terra et al. (2022).

#### Ecology.

The majority of *Senegalia* species occur in seasonally dry tropical habitats, especially seasonally dry tropical forests, scrublands and savannas. Despite this predilection for seasonally dry biomes, the genus shows wide adaptability, with some species occurring in wetter lowland tropical vegetation types, which accounts for its almost cosmopolitan distribution across the tropics. Details of habitats for many species of *Senegalia* are provided in Maslin et al. (2019a) and Bai et al. (2021) for East Asian species, Nielsen (1981b, 1985a, 1985b, 1992) for South East Asian species, Ross (1979) for African species, Du Puy and Villiers (2002) for Madagascan species, and Barros and Morim (2014) and Rico-Arce (2007) for American species.

#### Etymology.

The genus name refers to Senegal, a country in West Africa where the lectotype *S.senegal*, was collected.

#### Human uses.

The most valuable commercial species of *Senegalia* is the widespread African *S.senegal* (sect. Senegalia), which is the classical source of Gum Arabic. This water-soluble gum is also derived from a variety of other sources, including *Vachelliaseyal* (Delile) P.J.H. Hurter, where it is harvested from trees both in the wild and in plantations. Gum Arabic is used primarily in the food and soft-drink industries as a stabilizer but also has applications in the production of paint, glue and cosmetics, in textile industries and for viscosity control in inks. Also in Africa, the two sect. Senegalia species *S.nigrescens* and *S.caffra* (Thunb.) P.J.H. Hurter & Mabb. are sources of tannin (Mabberley 2017).

In Asia, the most commonly utilised species is *S.catechu* (L. f.) P.J.H. Hurter & Mabb. (sect. Senegalia). In Pakistan for example, its very durable and termite-resistant wood is used for house construction and for making agricultural implements; it is also an excellent source of firewood and is regarded as one of the best woods for charcoal production; tannin extracted from the wood is used for tanning (Ali 1973). The soft new shoots of S.pennatasubsp.insuavis (Lace) Maslin, Seigler & Ebinger (sect. Monacanthea p.p.) are commonly used in Asian cooking and are sold in some local markets in countries including China and Thailand; plants are sometimes grown as live fences in Thailand and northern Australia (Maslin et al. 2019a) and extracts from its roots are used in traditional medicine in Laos to help combat anaemia (Nielsen 1981b). The fruits of *S.rugata* (sect. Monacanthea p.p.) are also used for culinary purposes and as a traditional shampoo in Nepal (Singh 2016). *Senegaliamodesta* (Wall.) P.J.H. Hurter (sect. Senegalia) from Afghanistan, northern India, Pakistan and Myanmar is a source of Amritsar Gum (Mabberley 2017).

In the Americas many species of *Senegalia* are used locally for firewood; the wood is often converted to charcoal, but this is not usually economically important. Furthermore, almost all species of *Senegalia* that are large enough are used for making small tools, small instruments, furniture, and fence posts. The larger species are sometimes used for lumber [e.g., *Senegaliaaristeguietana* (L. Cárdenas) Seigler & Ebinger], but most do not have significant commercial value. Rico-Arce (2007) noted that American *Senegalia* are sometimes grown as live fences [e.g., *S.bonariensis* (Gillies ex Hook. & Arn.) Seigler & Ebinger] or ornamentals [e.g., *S.occidentalis* (Rose) Britton & Rose] and used in traditional medicines [e.g., *S.greggii* (A. Gray) Britton & Rose, this species is also sometimes used as a coffee substitute]. *Senegaliaberlandieri* (Benth.) Britton & Rose is an important honey source in Texas (Mabberley 2017), even though the foliage is toxic to livestock, especially cattle, on account of the alkaloids it possesses.

#### Notes.

Prior to the recent fragmentation of the former, broadly defined genus *Acacia*, species now regarded as *Senegalia* had been referred to Acaciasubg.Aculeiferum as defined by Vassal (1972). This subgenus corresponded to series *Vulgares* that Bentham (1842b) had erected, and which was maintained in his *magnum opus* (Bentham 1875) and was in use at the time of Vassal’s (1972) publication. *Senegalia* had been described by Rafinesque (1838) but had been overlooked or ignored until it was adopted for a short period by Britton and Rose (1928) in their Flora of North America. Within the context of a reassessment of the classification of *Acacia* s.l., Pedley (1986) resurrected *Senegalia* again. Although this classification was not widely adopted at that time, from 2000 onwards after phylogenetic evidence clearly showed that *Acacia* s.l. was polyphyletic, *Senegalia* was once again resurrected and four small New World genera were segregated from it. Regardless, *Senegalia* as currently defined is most likely not monophyletic (fide Terra et al. 2022).

Vassal’s (1972)AcaciasubgenusAculeiferum included two major infrageneric groups, section Aculeiferum which comprised species having cauline prickles located at the nodes, and section Monacanthea comprising species with cauline prickles scattered between the nodes; the type of this latter section was nominated as Acacia (Senegalia) ataxacantha. Over the past decade several phylogenetic analyses have included species of AcaciasubgenusAculeiferum (now *Senegalia*). Whether based on plastid DNA sequences (Bouchenak-Khelladi et al. 2010; Kyalangalilwa et al. 2013; Boatwright et al. 2015) or plastid loci combined with nrDNA ITS sequences (Terra et al. 2017), all recovered two well supported clades. One contains species with nodal prickles that are now included in the Afro-Asian SenegaliasectionSenegalia (= section Aculeiferum as recognised by Vassal 1972) plus species of the small African *S.ataxacantha* group that possessed internodal prickles (because this group included the type of section Monacanthea, it was designated as section Monacanthea sensu stricto by Terra et al. 2022). The second clade contains a pantropical group of species with internodal prickles, but as this group did not include *S.ataxacantha* it was designated section Monacanthea pro parte by Terra et al. (2022). This same three-group topology was also revealed by phylogenomic analyses of large numbers of nuclear genes (Ringelberg et al. 2022), albeit with very sparse taxon sampling.

In summary, the present concept of *Senegalia* is based on a combination of genetic and morphological evidence as detailed by Terra et al. (2022), and synoptically, the genus comprises the following three major groups:

**Senegaliasect.Senegalia**: cauline prickles 1, 2 or 3 at the nodes, flowers almost always in spikes; 51 species in Africa and Asia (Fig. 189D–J).

**Senegaliasect.Monacanthea s.s.**: cauline prickles internodal, flowers in spikes; four species in Africa (Fig. 189A–C).

**Senegaliasect.Monacanthea p.p.**: cauline prickles internodal but sometimes also at the nodes in some American species; flowers in heads or spikes; 164 species in Australia, Asia, Africa and the Americas. The name *Manganaroa* is available if this group is ever treated as a distinct genus (Fig. 190).

The pollen description above is based on Guinet (1969), Guinet and Vassal (1978), Nielsen (1992), Caccavari and Dome (2000) and Duarte et al. (2021). Where species of *Senegalia* are included in those works, they are described as having 16-grained polyads. However, in Guinet (1981b, Table 1) *Senegalia* was included in *Acacia* group one which was described as having (24-), 16-, 12-grained polyads. Because it is not known what species were included within that group, it is not possible to verify this range of variation for pollen grain number in *Senegalia*.

#### Taxonomic references.

Ali (1973); Bai et al. (2021); Barros and Morim (2014); Bentham (1842b, 1875); Britton and Rose (1928); Kyalangalilwa et al. (2013); Maslin et al. (2019a); Nielsen (1981b, 1985a, 1985b, 1992); Pedley (1986); Rafinesque (1838); Ross (1979); Terra et al. (2017, 2022); Vassal (1972).

### ﻿ Parasenegalia

Taxon classificationPlantaeFabalesFabaceae

Seigler & Ebinger, Novon 25(2): 181. 2017.

Figs 192 , 193

#### Type.

*Parasenegaliaskleroxyla* (Tussac) Seigler & Ebinger [≡ *Acaciaskleroxyla* Tussac]

#### Description.

**Figure 192.**
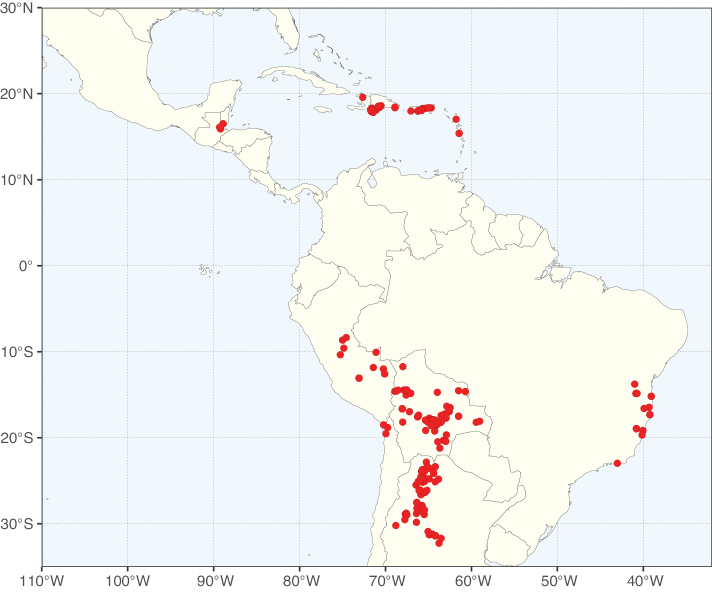
Morphology of *Mariosousa*, *Parasenegalia* and *Pseudosenegalia***A***Mariosousaacatlensis* (Benth.) Seigler & Ebinger spicate inflorescences (*Seigler 16002*) **B***M.coulteri* (Benth.) Seigler & Ebinger bark not papery **C***M.dolichostachya* (S.F. Blake) Seigler & Ebinger branchlet unarmed (*Seigler 16035*) **D–F***M.salazari* (Britton & Rose) Seigler & Ebinger **D** exfoliating papery bark (*Seigler 16057*) **E** habit **F** fruits (*Seigler 15978*) **G***M.usumacintensis* (Lundell) Seigler & Ebinger leaf (*Seigler 16027*) **H***Pseudosenegaliafeddeana* (Harms) Seigler & Ebinger fruits (*Atahuachi 1146*) **I, J***Parasenegaliamiersii* (Benth.) Seigler & Ebinger (*Terra, Coutinho & Dalvi 667*) **I** branch showing unusual leaves (i.e., pinnae few and leaflets large) **J** inflorescences **K***Pseudosenegaliafeddeana* contorted tree habit. Photo credits **A–G** B Maslin **H, K** CE Hughes **I, J** V Terra.

Unarmed trees (some to 25 m), shrubs (often lianescent) or lianas; bark (known for only few species) grey to brown, smooth to shallowly flat-ridged or shallowly fissured; brachyblasts absent. **Stipules** small, often falling tardily, rarely absent (*P.lundellii* Seigler & Ebinger), not spinescent. **Leaves** bipinnate, not sensitive; extrafloral nectaries present on petiole (variably positioned) and rachis (below uppermost 1–7 pairs or sometimes all pairs of pinnae), sessile or occasionally stipitate; pinnae normally 3–16 (–20) pairs [except 1–2 pairs in *P.miersii* (Benth.) Seigler & Ebinger]; paraphyllidia present or absent; leaflets opposite, normally 6–46 (65) pairs per pinna (except 1 or rarely 2 pairs in *P.miersii*). **Inflorescences** comprising pedunculate, loosely flowered spikes (1–4 in leaf axils) or densely flowered heads (1–4 in leaf axils or arranged in large, axillary and/or terminal racemes or panicles); peduncles lacking a multi-bracteate involucre**. Flowers** hermaphrodite, uniform, 5-merous, with a basal nectariferous disk, sessile, white to cream (sometimes aging yellowish); perianth connate, valvate, not scarious; stamens numerous (40–140), free; anther gland normally present; pollen comprising 16-grained polyads, the grains porate and lacking pseudocolpi, exine surface psilate with circular depressions or rugulate-perforate, exine lacking columellae; ovary sessile to subsessile (except stipitate with stipe to 1.6 mm long in *P.vogeliana*). **Fruits** dehiscent along both sutures (sometimes tardily so), flattened, valves chartaceous or coriaceous. **Seeds** sometimes with a narrow marginal wing, exarillate; pleurogram U-shaped (open at hilar end) or sometimes circular, rarely absent (e.g., *P.miersii*) (Fig. 192I, J).

#### Chromosome number.

2*n* = 26 (known only for *P.visco*), fide Goldblatt and Johnson (1979–), as *Acacia*.

#### Included species and geographic distribution.

**Figure 193.**
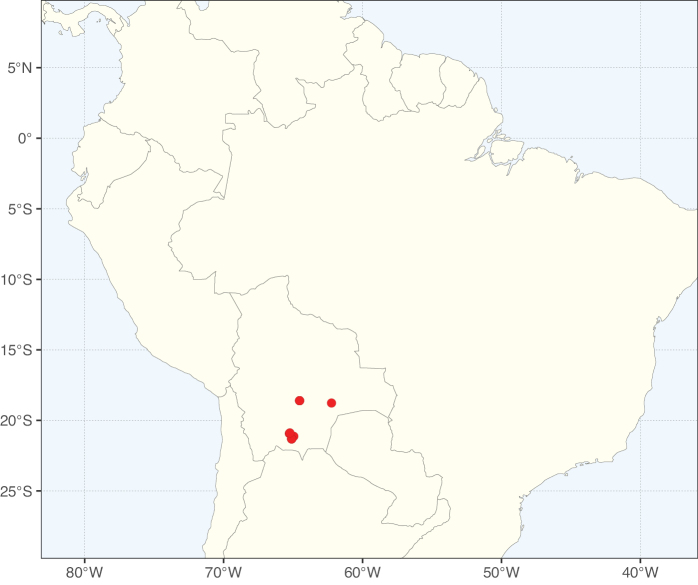
Distribution of *Parasenegalia* based on quality-controlled digitised herbarium records. See Suppl. material 1 for the source of occurrence data.

Eleven species, widely but discontinuously distributed in the Neotropics from the Caribbean (three endemic species) through Central America (one species in Belize and Guatemala) to South America (two species in Argentina, Bolivia, Chile and Peru, and five endemic to Brazil) (Fig. 193).

#### Ecology.

Commonly found in evergreen or semi-evergreen tropical, often riparian, forests or disturbed second growth forests and thickets, 0–800 m. (except *P.visco* from northern Argentina, Bolivia, northern Chile and Peru, which grows in seasonally wet montane regions between 1000–3000 m), as well as seasonally dry deciduous or semi-deciduous forests, savannas and desert scrub.

#### Etymology.

The genus name *Parasenegalia* is from the Greek *para* (= beside, near) + *Senegalia*, in reference to the close relationship to that genus.

#### Human uses.

The wood of *P.muricata* (L.) Seigler & Ebinger is sometimes used for construction and *P.visco* is commonly cultivated in Peru where it is economically important as a source of cabinet wood (Rico-Arce 2007).

#### Notes.

Prior to the recognition of *Parasenegalia*, most species now assigned to this genus had been included in a broadly circumscribed *Senegalia* (e.g., Seigler et al. 2006a; Barros and Morim 2014) and before that, regarded as *Acacia* (s.l.). Bentham (1875) included two of the species, *P.muricata* (= *A.nudiflora* Rich. ex Willd.) and *P.skleroxyla* in his treatment of *Acacia* under the *Nudiflorae* group of subseries *Americanae Spiciflorae*; this group also included species now assigned to *Mariosousa* and *Senegalia*. For a short period, some species of *Parasenegalia* were referred to the informal ‘*skleroxyla*’ group of Acaciasubg.Aculeiferum (fide Seigler et al. 2017; Miller et al. 2017).

When *Parasenegalia* was first recognised it included seven species that were comprehensively described and illustrated (see Seigler and Ebinger in Seigler et al. 2017). Seigler and Ebinger (2018) subsequently recognised an additional four species and provided an identification key to all 11 species as well as a key to the four genera of the Senegalia grade, together with *Acaciella*.

*Parasenegalia* and *Pseudosenegalia* were simultaneously described by Seigler et al. (2017) as New World segregates of *Senegalia*, differing from that genus notably by their lack of prickles. In a companion paper, Miller et al. (2017) found *Parasenegalia* to be strongly supported as monophyletic so long as *P.visco* was excluded from the group. These authors suggested that future studies may show that this species may warrant recognition as a distinct, monotypic genus. Indeed, as noted above, in a recent sparsely sampled phylogenetic study where *Parasenegalia* was represented by *P.visco* and *P.vogeliana* (= *Albizialeonardii*) (Terra et al. 2022), these two species did not form a monophyletic group, supporting doubts about the monophyly of *Parasenegalia* (Fig. 188). Apart from its distinctive habitat (see above), *P.visco* differs from other members of *Parasenegalia* in having larger flower heads (16–23 mm diam. vs 5–14 mm) and leaflets with dark bluish purple midveins; these leaflets are at the lower end of the size range for the genus in being 3–7 × 0.8–2.1 mm. Notwithstanding these morphological differences, *P.visco* was included in *Parasenegalia* on account of its leaflet size (3–7 × 0.8–2.1 mm) and its possession of anther glands (fide Miller et al. 2017).

The principal morphological characters that distinguish *Parasenegalia* from *Pseudosenegalia* are noted below under the latter genus. Note that pollen data are only available for three species of *Parasenegalia*, namely, *P.miersii*, *P.santosii* (G.P. Lewis) Seigler & Ebinger and *P.visco* (Caccavari and Dome 2000; Duarte et al. 2021).

#### Taxonomic references.

Barros and Morim (2014); Bentham (1875); Miller et al. (2017); Nielsen (1992); Seigler and Ebinger (2018); Seigler et al. (2006a, 2017); Terra et al. (2022).

### ﻿ Pseudosenegalia

Taxon classificationPlantaeFabalesFabaceae

Seigler & Ebinger, Novon 25(2): 199. 2017.

Figs 192 , 194

#### Type.

*Pseudosenegaliafeddeana* (Harms) Seigler & Ebinger [≡ *Acaciafeddeana* Harms]

#### Description.

Unarmed trees (some to 12 m) or shrubs; bark white to grey-white and smooth; brachyblasts present at some or all nodes. **Stipules** small, persistent or falling late, not spinescent. **Leaves** bipinnate, not sensitive; pinnae 1–7 pairs; paraphyllidia absent; leaflets opposite, 11–26 pairs per pinna; gland present at apex of petiole and (when leaves multijugate) on rachis below the uppermost 1–2 pinna pairs, sessile. **Inflorescences** comprising pedunculate, loosely flowered spikes, 1 or 2 in leaf axils; peduncles lacking a multi-bracteate involucre. **Flowers** hermaphrodite, uniform, 5-merous, with a basal nectariferous disk, sessile, white or pale creamy white; perianth connate, valvate, scarious; stamens numerous (100–150), free; anther glands absent; pollen not studied; ovary stipitate. **Fruits** dehiscent along both sutures, flattened or elliptic in cross-section, valves coriaceous, twisting after dehiscence. **Seeds** not winged, exarillate; pleurogram U-shaped (open at hilar end) or absent in *P.feddeana* (Fig. 192H, K).

#### Chromosome number.

Unknown.

#### Included species and geographic distribution.

**Figure 194.**
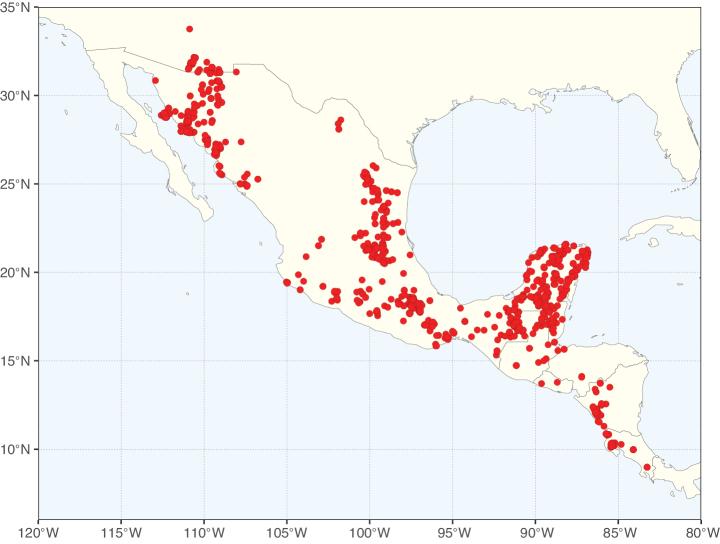
Distribution of *Pseudosenegalia* based on quality-controlled digitised herbarium records. See Suppl. material 1 for the source of occurrence data.

Two species, endemic to Bolivia (Fig. 194).

#### Ecology.

In seasonally dry tropical forest, dry scrub and thorn-scrub between 1300 and 3300 m elevation.

#### Etymology.

The generic name *Pseudosenegalia* is from Greek *pseudo* (= false) + *Senegalia*, in reference to a superficial resemblance to that genus.

#### Human uses.

*Pseudosenegaliafeddeana* is important locally as a forage plant and its wood is used as a fuel; also, its seeds are edible and used as a coffee substitute (Rico-Arce 2007, as *Acacia*).

#### Notes.

Seigler et al. (2017) described *Pseudosenegalia* as a segregate of *Senegalia* for two New World species which lack prickles, namely, *P.feddeana* and *P.riograndensis* (Atahuachi & L. Rico) Seigler & Ebinger. In that work the two species were comprehensively described and illustrated, and an identification key provided. A phylogenetic study based on plastid and nuclear sequence data supported the monophyly of *Pseudosenegalia* which was resolved as sister to a clade containing *Parasenegalia* and *Mariosousa* (Seigler et al. 2017). A revised key that included all species of both genera is given in Seigler and Ebinger (2018).

Both species of *Pseudosenegalia* were originally described under *Acacia* and subsequently transferred to *Senegalia* by Seigler et al. (2006a) and Seigler and Ebinger (2009). Prior to the recognition of *Pseudosenegalia* as a distinct genus, these two species had never been treated together as a distinct taxonomic unit, nor had they been included in former major classifications such as those of Bentham (1875) or Britton and Rose (1928) or assigned to an informal species group.

Species of *Pseudosenegalia* are readily distinguished from those of the more speciose and widespread *Parasenegalia* by their extremely small leaflets (1–3 × 0.5–1 mm compared with normally (3) 5–18 × 0.5–11 mm in *Parasenegalia*), the presence of brachyblasts on branchlets, the absence of anther glands and by a scarious-textured perianth. The latter trait is otherwise unknown in the Senegalia grade. Even in the very large genus *Acacia* (1082 species), only one species, *A.unifissilis* Court, is described as having a scarious calyx (Court 1978).

#### Taxonomic references.

Seigler and Ebinger (2009, 2018); Seigler et al. (2006a, 2017).

### ﻿ Mariosousa

Taxon classificationPlantaeFabalesFabaceae

Seigler & Ebinger, Novon 16(3): 415. 2006.

Figs 192 , 195

#### Type.

*Mariosousacoulteri* (Benth.) Seigler & Ebinger [≡ *Acaciacoulteri* Benth.]

#### Description.

Unarmed shrubs and trees; bark hard and fissured or scaly, sometimes papery and exfoliating; brachyblasts normally absent (except commonly present in *M.compacta* (Rose) Seigler & Ebinger). **Stipules** small, mostly persistent, not spinescent. **Leaves** bipinnate, not sensitive; extrafloral nectaries present on petiole and/or rachis (petiole glands commonly absent in *M.gentryi* Seigler & Ebinger, *M.millefolia* (S. Watson) Seigler & Ebinger and *M.salazarii* (Britton & Rose) Seigler & Ebinger), sessile or occasionally stipitate; pinnae 1–20 (30) pairs; paraphyllidia present or absent; leaflets opposite, (4) 8–65 pairs per pinna. **Inflorescences** comprising pedunculate, loosely flowered spikes, 1–4 in leaf axils or arranged in short, normally terminal, racemose clusters; peduncles lacking a multi-bracteate involucre. **Flowers** hermaphrodite, uniform, 5-merous, with a basal nectariferous disk, sessile, creamy white; perianth connate, valvate, not scarious; stamens numerous (50+), free; anther glands normally present; pollen comprising 16-grained polyads, porate and lacking pseudocolpi, exine surface rugulate, exine lacking columellae; ovary stipitate. **Fruits** dehiscent along both sutures, strongly flattened, valves chartaceous or coriaceous. **Seeds** exarillate, not winged, pleurogram U-shaped (open at hilar end) and areole usually large (covering 50%–70% of the seed) (Fig. 192A–G).

#### Chromosome number.

Unknown.

#### Included species and geographic distribution.

**Figure 195.**
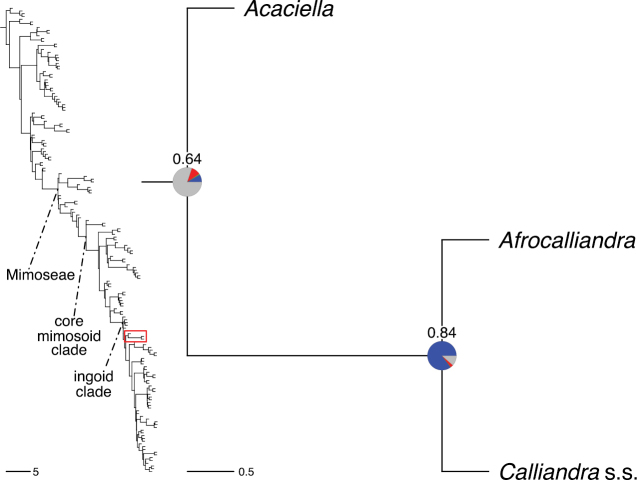
Distribution of *Mariosousa* based on quality-controlled digitised herbarium records. See Suppl. material 1 for the source of occurrence data.

Fourteen species distributed from Arizona and New Mexico (one species), south through Mexico (where all species of the genus occur) to Belize, Guatemala, El Salvador, Honduras, Nicaragua and Costa Rica (where three species occur, the most widespread being *M.centralis* (Britton & Rose) Seigler & Ebinger) (Fig. 195).

#### Ecology.

Mainly in seasonally dry tropical forests, thorn-scrub and thickets, weakly extending into moister semi-deciduous tropical forests; on arid hills or sometimes rocky deserts or desert grasslands, 0–2200 m elevation.

#### Etymology.

The genus *Mariosousa* honours Mario Sousa (1940–2017), legume specialist and former Director of the Herbarium of the Instituto de Biología (MEXU), Universidad Nacional Autónoma de México.

#### Human uses.

*Mariosousacoulteri* is one of the few trees of this genus with enough size and quality of wood to be of some local importance and useful for lumber (Seigler and Jones 2005). The wood is extremely durable and is sought after for building houses.

#### Notes.

Immediately prior to the description of *Mariosousa*, the 13 species that were subsequently assigned to this genus were included in the informal *Acaciacoulteri* species-group (Jawad et al. 2000). Before that, many species had been treated as *Senegalia* by Britton and Rose (1928) or as *Acacia* by Bentham (1875) under the *Nudiflorae* group of subseries *Americanae Spiciflorae*; this group also included some species now assigned to both *Parasenegalia* and *Senegalia. Mariosousa* together with *Parasenegalia* (and *Pseudosenegalia*) are distinguished from *Senegalia* by their lack of prickles. *Mariosousa* is further distinguished from *Senegalia* by the order of development of seedling leaves: those of *Mariosousa* having the first two leaves pinnate followed by a bipinnate leaf, whereas in *Senegalia* the first three leaves are bipinnate or a singly pinnate leaf followed by two bipinnate leaves (Vassal 1972). A key that includes these four genera is provided in Miller et al. (2017).

Phylogenetic analyses of plastid and/or nuclear sequence data have supported the genus as monophyletic (Seigler et al. 2006a, 2006b; Miller et al. 2017). Jawad et al. (2000) had previously used morphometric studies to revise the group and included a key to species and formal descriptions. Seigler et al. (2006a, 2006b) also provided a key to the then recognised 13 species of *Mariosousa*, but an additional species, *M.gentryi*, was described by Seigler and Ebinger (2021).

*Mariosousaheterophylla* (Benth.) Seigler & Ebinger [= *Acaciawillardiana* Rose, ≡ *Mariosousawillardiana* (Rose) Seigler & Ebinger], which is endemic to the state of Sonora, Mexico, is morphologically somewhat unusual, but is clearly nested within *Mariosousa* (fide Seigler et al. 2006a, 2006b). Apart from its exfoliating, papery bark (which also occurs in *M.gentryi* and *M.salazari*) the exceptionally long flattened petioles (2–40 cm) that normally support a single pair of pinnae are highly distinctive within the genus. Bentham (1846) noted these characters and commented that the petiole was ‘almost phyllodinous’ when he tentatively assigned this taxon to *Prosopisheterophylla* Benth. Vasey and Rose (1890) subsequently referred this species to *Acacia*, as *A.willardiana*; a proposal by Seigler and Ebinger (2008) to conserve this name against *P.heterophylla* was declined (Brummitt 2011; Barrie 2011). Vassal and Guinet (1972) conducted a detailed study of the phyllodinous petioles of *A.willardiana* which they described as being horizontally flattened, and as such applied the term ‘diaphyllode’ to them. Bentham (1846) had erroneously described the phyllodes as vertically flattened (‘orthophyllodes’), which is the normal condition found in species of *Acacia*. However, diaphyllodes do occur in few disparate groups of Australian *Acacia* (Vassal and Maslin 1979). Note that pollen data are only available for two species of *Mariosousa*, namely, *M.centralis* and *M.coulteri* (Guinet 1969; Caccavari and Dome 2000; Rico Arce and Banks 2001; Duarte et al. 2021).

#### Taxonomic references.

Bentham (1846, 1875); Britton and Rose (1928); Jawad et al. (2000); Miller et al. (2017); Nielsen (1992); Seigler et al. (2006a, 2006b, 2023); Seigler and Ebinger (2008, 2021); Vasey and Rose (1890); Vassal (1972).

## ﻿﻿27. Calliandra clade

Héctor M. Hernández^22^

Citation: Hernández HM (2024) 27. Calliandra clade. In: Bruneau A, Queiroz LP, Ringelberg JJ (Eds) Advances in Legume Systematics 14. Classification of Caesalpinioideae. Part 2: Higher-level classification. PhytoKeys 240: 358–370. https://doi.org/10.3897/phytokeys.240.101716

**Calliandra clade**

Figs 196–202

**Included genera (3).***Acaciella* Britton & Rose (15 species), *Afrocalliandra* E.R. Souza & L.P. Queiroz (2), *Calliandra* Benth. (140).

**Description.** Shrubs or small trees, rarely perennial herbs or geoxylic subshrubs, the branches often provided with brachyblasts from which leaves and inflorescences arise, usually unarmed but in a few cases armed with long shoots tapering at apex into a stout thorn or with spinescent stipules. **Stipules** persistent or caducous, rarely spinescent. **Leaves** bipinnate, once pinnate in a single species; petioles and rachis without extrafloral nectaries; pinnae 1–many pairs; leaflets (1) 2–many pairs per pinna, small (ca. 5 mm) to up to 12 cm long. **Inflorescences** capitate, umbelliform or sometimes elongated into short racemes, usually pedunculate, solitary or fasciculate, arising from leaf axils or from brachyblasts, sometimes with the capitula or umbels forming long, terminal pseudoracemes through suppression of distal leaves; capitula either homomorphic or heteromorphic. **Flowers** sessile, subsessile or pedicellate; calyx often cup-shaped, less frequently turbinate, hemispherical or inflated, (3) 5 (6)-merous; corolla cup-shaped to tubular, (3) 5 (6)-merous; stamens (8) 10 to moderately or extremely numerous (up to 300 in *Acaciella*) in a single flower, exserted or long exserted from the corolla, free from the base or basally fused to form a conspicuous tube, this disproportionately long in the central flowers of heteromorphic capitula, red, pink or white, or white in the basal half and pink or red in the distal half, rarely bright yellow; anthers eglandular; pollen in 8-grained polyads, rarely with 7 or up to 10 grains in *Afrocalliandra*, the polyads calymmate or acalymmate, with or without a basal grain acutely narrowed and bearing a sticky appendage. **Fruits** linear, oblanceolate, narrowly oblong or linear-oblanceolate, flattened, straight or slightly curved, with thickened margins, the valves membranous, coriaceous, chartaceous or ligneous, dehiscing passively or elastically along both margins, recurving from apex downwards. **Seeds** without an aril, discoid, ovoid or rhomboid, usually compressed, with or without U-shaped pleurogram.

**Geographic distribution.** The Calliandra clade is primarily a Neotropical group, with the exception of *Afrocalliandra* whose two species are allopatrically distributed in restricted arid areas of Kenya and adjacent Somalia, and in the northern Cape Province of South Africa.

**Figure 196.**
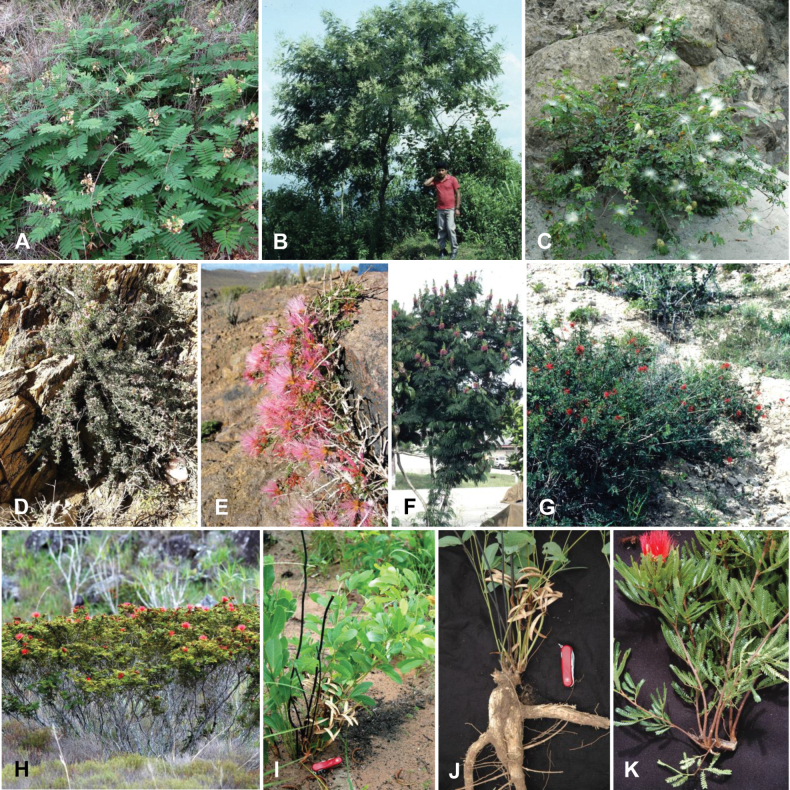
Generic relationships in the Calliandra clade (tribe Mimoseae). For description of phylogeny and support values, see Fig. 6 caption (page 63).

**Notes.** In the phylogenomic analyses of Ringelberg et al. (2022), the three genera here circumscribed as the Calliandra clade are resolved for the first time as a monophyletic group with *Acaciella* sister to the other two. In a well-sampled phylogenetic analysis focused on *Calliandra* and close relatives, and using plastid and nuclear ribosomal sequences, Souza et al. (2013) had resolved a strongly supported New World *Calliandra* clade sister to which were two African *Calliandra* species, which they transferred to *Afrocalliandra* based on molecular and morphological evidence. Although *Acaciella* was not sampled in Souza et al. (2013), a previous study on *Acacia* s.l. and ant mutualisms had found most *Acaciella* species sampled to form a clade with one species of *Calliandra* (Gomez-Acevedo et al. 2010; see also Koenen et al. 2020a).

**Clade-based definition.** The most inclusive crown clade containing *Calliandrahoustoniana* (Mill.) Standl. and *Acaciellaangustissima* (Mill.) Britton, but not *Zapotecacaracasana* (Jacq.) H.M. Hern., *Senegaliabahiensis* (Benth.) Seigler & Ebinger or *Pithecellobiumdulce* (Roxb.) Benth. (Fig. 196).

The phylogenetic affinity of the three genera (ca. 157 species) of the Calliandra clade is reflected by the presence of several shared morphological characters: predominantly unarmed shrubs or small trees, bipinnate leaves [except *C.hymenaeoides* (Rich.) Benth.], lack of extrafloral nectaries, 8-grained polyads, dehiscent, flat pods with thickened margins, and exarillate, usually compressed seeds. *Acaciella* has the most disparate morphological characters compared with the other two genera, especially with respect to flower traits (e.g., lack of staminal tube, short and extremely numerous stamens), and pods with passive, rather than active (elastic) dehiscence.

All species of the clade are unarmed and lack spinescent stipules, except two species of *Calliandra* [*C.haematomma* (DC.) Benth. and *C.pauciflora* (A. Rich.) Griseb.] and *Afrocalliandraredacta* (J.H. Ross) E.R. Souza & L.P. Queiroz. In addition, *Afrocalliandragilbertii* (Thulin & Hunde) E.R. Souza & L.P. Queiroz and some Brazilian species of *Calliandra* (e.g., *C.spinosa* Ducke) are armed with tapering branches becoming spinescent at the tips.

Eight-grained polyads are common to all members of the clade, although those of *Calliandra* are characteristically calymmate, all grains being covered by a common exine. In contrast, the polyads of all species of *Acaciella* and *Afrocalliandra* are acalymmate; in *Afrocalliandraredacta* the number of grains per polyad has been reported to vary from 7 to 10, but 8-grained polyads are the most common (Robbertse and von Teichman 1979). However, it is important to emphasise that *Calliandra* and *Afrocalliandra* share a unique combination of polyad characters within Mimoseae. The polyads in these two genera have an ellipsoid, tear-shaped outline displaying a highly modified “basal” grain with an acute apex, this provided with a mucilaginous appendage. This sticky appendage, which is associated with the transfer of polyads by pollinators, is extremely large and conspicuous in *Calliandra* (see fig. 2 in Hernández 1986), and appears to be rather rudimentary in *A.gilbertii* (see fig. 3G in Thulin et al. 1981). The unique polyad characteristics of *Calliandra* in particular allowed the identification of fossil *Calliandra* polyads from the middle Miocene of north-west Argentina (Caccavari and Barreda 2000), and because they are so distinctive and diagnostic of the genus, they have been used to constrain molecular phylogenetic divergence time estimations.

All species, with the exception of the two *Afrocalliandra* species, occur in tropical and subtropical regions of the Americas. Mexico appears to have been an important region for the evolution of the clade with almost all species of *Acaciella* occurring in this country (14 of 15 species), and 39 species of *Calliandra*, 19 of which are endemic. However, Brazil is by far the main centre of distribution of *Calliandra*, with ca. 71 species, most of them endemic. The group as a whole is absent (*Acaciella*) or poorly represented (*Calliandra*) in the Amazon basin. The vast majority of species occur in areas dominated by seasonally dry tropical climates and even in semi-desert regions, and less frequently in more mesic *Quercus* or *Pinus-Quercus* forests, grasslands or savannas. Although not known for *Afrocalliandra*, the two other genera of the Calliandra clade are reported to fix nitrogen with a symbiosome-type nodule anatomy (Faria et al. 2022).

### ﻿ Acaciella

Taxon classificationPlantaeFabalesFabaceae

Britton & Rose, N. Amer. Fl. 23(2): 96. 1928.

**Figure 197.**
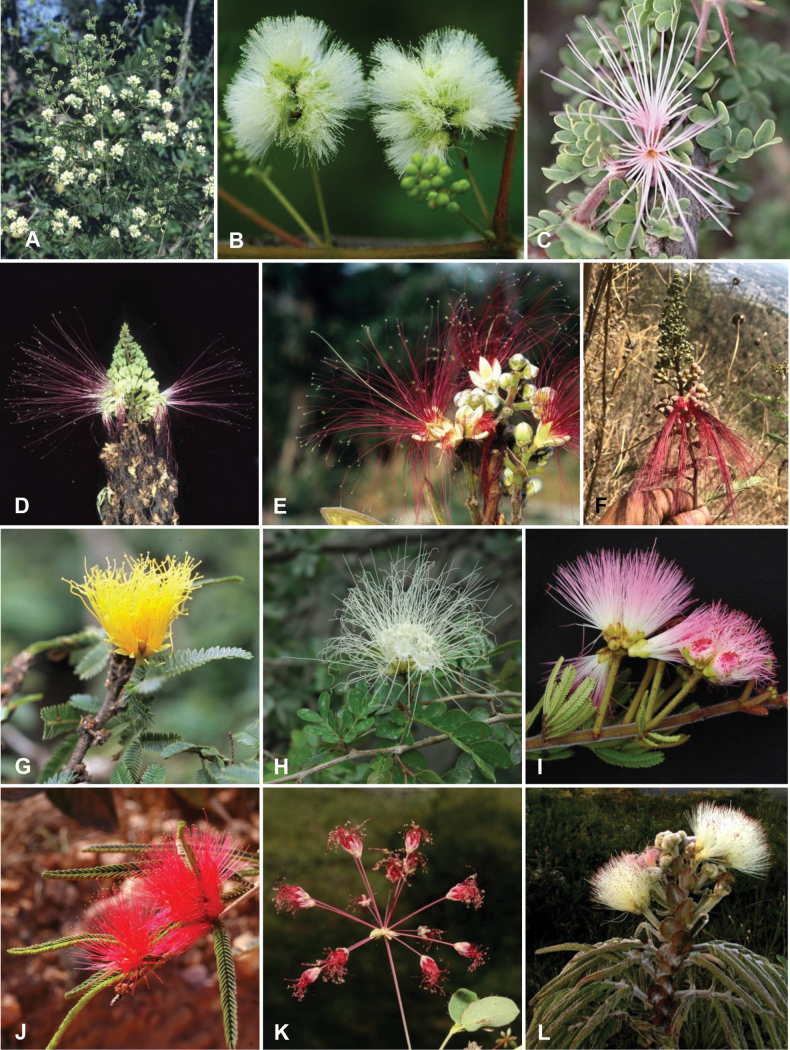
Diversity of plant growth forms of the genera of the Calliandra clade **A** shrubby habit of Acaciellaangustissima(Mill.)Britton & Rosevar.angustissima, Texas, USA **B** treelet of Acaciellaangustissimavar.angustissima, Chiquimula, Guatemala (*Hughes 1487*) **C** shrub of *Calliandramollissima* (Humb. & Bonpl. ex Willd.) Benth., Marañón Valley, Peru (*Särkinen 2198*) **D** stunted xerophytic shrublet of *Afrocalliandraredacta* (J.H. Ross) E.R. Souza & L.P. Queiroz, Kuboes, South Africa **E** stunted xerophytic shrublet of *Calliandrachilensis* Benth., northern Chile **F** small treelet of *Calliandracalothyrsus* Meisn., Siguatepeque, Honduras **G** shrub of *Calliandracalifornica* Benth., Baja California Sur, Mexico (*Hughes 1546*) **H** treelet of *Calliandrafuscipila* Harms, Serra do Espinhaço, Bahia, Brazil (*Queiroz 15626*) **I, J** functionally herbaceous geoxyle of *Calliandralongipes* Benth. arising from a stout lignotuber and resprouting and fruiting after fire in savanna woodland, Santa Cruz, Bolivia (*Wood 26548*) **K** geoxylic shrublet of *Calliandramucugeana* Renvoize, arising from a stout woody underground stem and forming carpet-like thickets, Serra do Espinhaço, Bahia, Brazil (*Queiroz 15540*). Photo credits **A** Ron Stephens, iNaturalist (https://www.inaturalist.org/photos/199321699) **B, C, F–K** CE Hughes **D** Pietermier, iNaturalist (https://www.inaturalist.org/photos/152398478) **E** J Jiménez Castillo.

**Figure 198.**
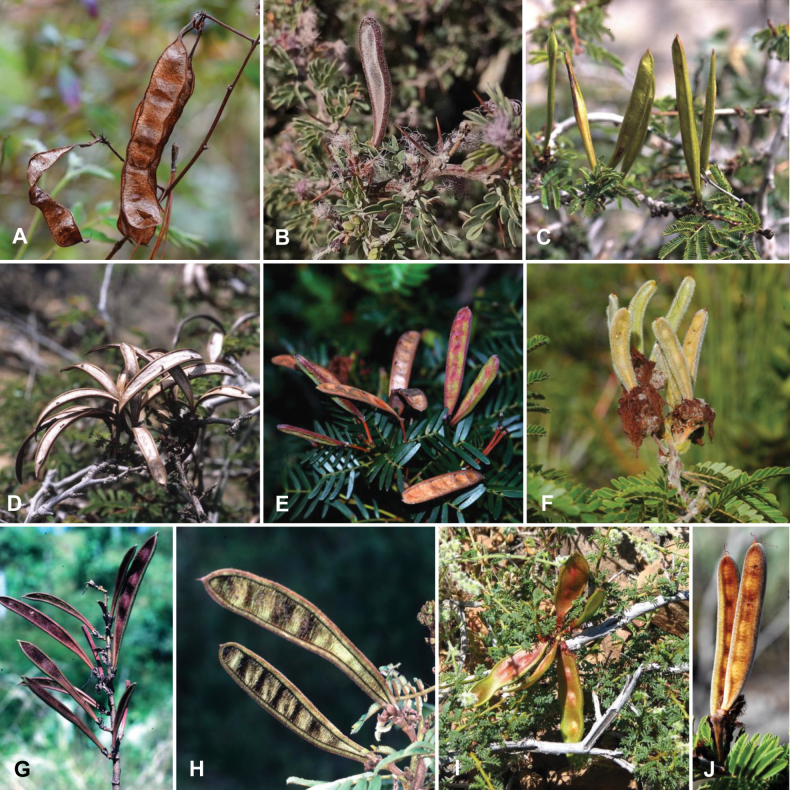
Variation in inflorescences of the genera of the Calliandra clade. **A** compound terminal panicle of capitula of *Acaciellavillosa* (Sw.) Britton & Rose, Oaxaca, Mexico (*Hughes 1333*) **B** capitula of *Acaciellavillosa*, Piura, Peru (*Hughes 2635*) **C** flowers of *Afrocalliandraredacta* (J.H. Ross) E.R. Souza & L.P. Queiroz, Kuboes, South Africa **D** erect terminal racemose inflorescence of *Calliandracalothyrsus* Meisn. flowering at night, Siguatepeque, Honduras (*Macqueen 3*) **E** erect terminal racemose inflorescence of *Calliandrajuzepczukii* Standl., Oaxaca, Mexico (*Hughes 1675*) **F** erect terminal inflorescence with buds and flowers opening acropetally, *Calliandragrandiflora* (L’Her.) Benth., Mexico City, Mexico **G** inflorescence of *Calliandrataxifolia* (Kunth) Benth., Mollendo, Arequipa, Peru (*Hughes 2357*) **H** heteromorphic inflorescence of *Calliandramollissima* (Humb. & Bonpl. ex Willd) Benth., Marañón Valley, Peru, showing central flowers with enlarged staminal tubes (*Särkinen 2198*) **I***Calliandranebulosa* Barneby, Serra do Espinhaço, Bahia, Brazil (*Queiroz 15624*) **J** flowers of *Calliandralongipinna* Benth., Serra do Espinhaço, Bahia, Brazil (*Queiroz 15603*) **K** long pedicellate flowers of *Calliandraleptopoda* Benth., Serra do Espinhaço, Bahia, Brazil **L** leaves, foliaceous stipules and terminal inflorescences of *Calliandralanata* Benth., Serra do Espinhaço, Bahia, Brazil. Photo credits **A, B, D, E, G–J** CE Hughes **C** Pietermier, iNaturalist (https://www.inaturalist.org/photos/15308160) **F** Mvz-juangonzalezromero, iNaturalist (https://www.inaturalist.org/photos/195966667) **K, L** E de Souza.

**Figure 199.**
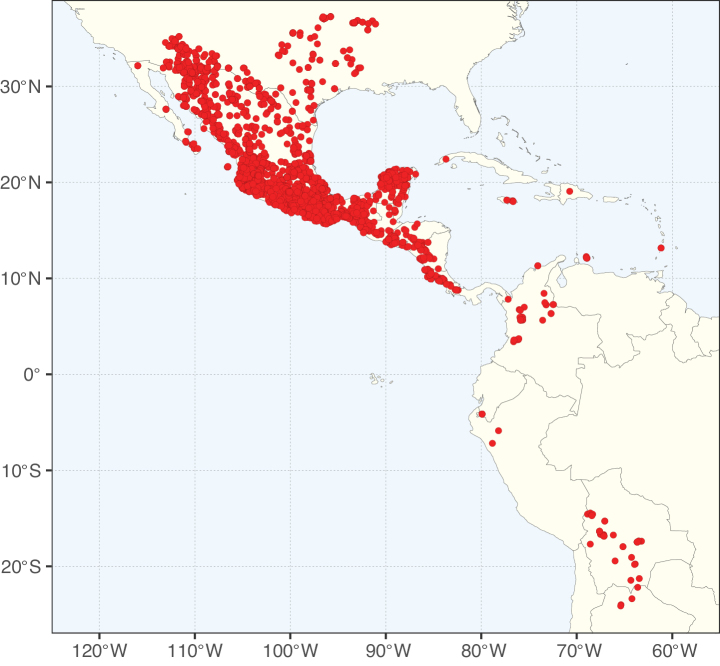
Variation in fruits across genera of the Calliandra clade **A** pendulous fruit of Acaciellaangustissima(Mill.)Britton & Rosevar.angustissima, Chiapas, Mexico **B** erect fruit and spinescent stipules of *Afrocalliandraredacta* (J.H. Ross) E.R. Souza & L.P. Queiroz, Kuboes, South Africa **C, D** fruits of *Calliandrataxifolia* (Kunth) Benth., Mollendo, Arequipa, Peru (*Hughes 2357*) **C** unripe green erect fruits **D** ripe fruits elastically dehiscent from the apex, the valves recurved backwards **E** ripe and unripe fruits of *Calliandraluetzelburgii* Harms, Serra do Espinhaço, Bahia, Brazil (*Queiroz 15618*) **F** unripe, green, erect fruits of *Calliandraviscidula* Benth., Serra do Espinhaço, Bahia, Brazil (*Queiroz 15541*) **G, H** fruits of *Calliandrahoustoniana* (Mill.) Standl., Chiapas, Mexico (*Hughes 1271* & *1287*) **I** unripe fruits of *Calliandrachilensis* Benth., northern Chile **J** unripe, erect fruits of CalliandrabahianaRenvoizevar.erythematosa Barneby, Serra do Espinhaço, Bahia, Brazil (*Queiroz 15622*). Photo credits **A** Neptalí Ramírez Marcial, iNaturalist (https://www.inaturalist.org/photos/199788369) **B** Pietermier, iNaturalist (https://www.inaturalist.org/photos/152398411) **C–H, J** CE Hughes **I** J Jiménez Castillo.

**Figure 200.**
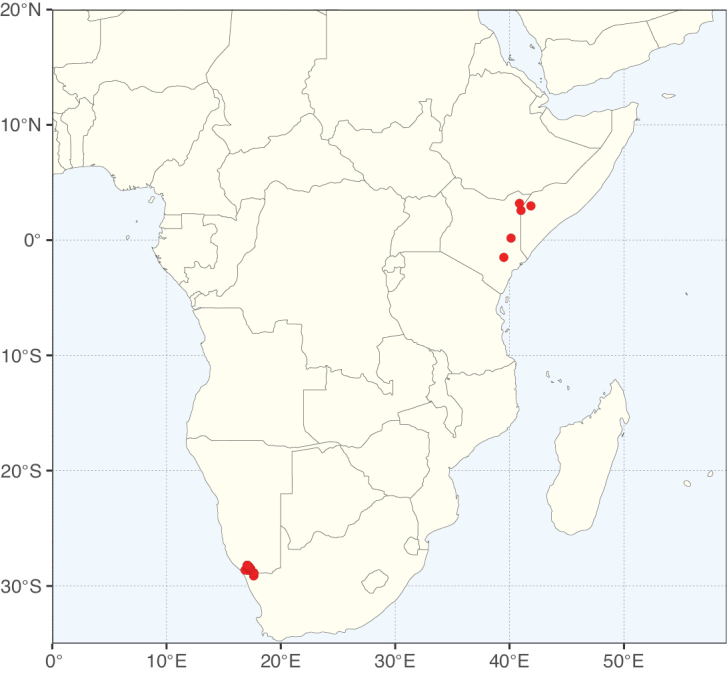
Distribution of *Acaciella* based on quality-controlled digitised herbarium records. See Suppl. material 1 for the source of occurrence data.

Figs 197 , 198 , 199 , 200

Acacia ser. Filicinae Benth., London J. Bot. 1: 322. 1842. Type not designated.
Acacia sect. Filicinae (Benth.) Taub., Nat. Pflanzenfam. 3(3): 113. 1894. Type not designated.
Senegalia sect. Filicinae (Benth.) Pedley, Bot. J. Linn. Soc. 92(3): 238. 1986. Type: Senegaliaangustissima (Mill.) Pedley [≡ Mimosaangustissima Mill. (≡ Acaciellaangustissima (Mill.) Britton & Rose)]

#### Type.

*Acaciellavillosa* (Sw.) Britton & Rose [≡ *Mimosavillosa* Sw.]

#### Description.

Shrubs or small trees to 12 m (Fig. 197A, B), rarely perennial herbs, always unarmed. **Stipules** leafy, ephemeral or rarely persistent, to 5 (10) mm. **Leaves** bipinnate; petioles and rachis without extrafloral nectaries; pinnae 2–25 pairs; leaflets 1–30 pairs per pinnae, predominantly small (to 12 mm long) but very large (to 3–6.5 cm) in some species. **Inflorescences** capituliform, sometimes elongated into short racemes, pedunculate, often organised in long efoliate terminal panicles (Fig. 198A, B); inflorescence units homomorphic. **Flowers** bracteate, the bracts caducous, pedicellate, the pedicels persistent after the flowers have fallen; calyx cup-shaped, 5-merous, the lobes less than ¼ the length of the calyx tube to almost truncate; corolla 5-merous, the lobes more than ½ the whole corolla length; stamens always numerous (Fig. 198B), sometimes to 300 in a single flower, exserted from the corolla, always free and white, drying yellow, orange or pink; anthers eglandular; pollen in 8-grained polyads; ovary stipitate, the stipe sometimes ¼ the length of the ovary. **Fruits** linear to narrowly oblong, flattened, straight, mucronate and stipitate, with thickened margins, the valves membranous or chartaceous (Fig. 199A), dehiscing along both margins from apex downwards, seeds up to 8 (12) per pod. **Seeds** lenticular or spherical, dark brown, with a prominent U-shaped pleurogram.

#### Chromosome number.

*2n* = 26 [*A.texensis* (Torr. & A.Gray) Britton & Rose] (Rico and Bachman 2006).

#### Included species and geographic distribution.

Fifteen species are currently recognised (Rico Arce and Bachman 2006), all restricted to the Neotropics. With the exception of *A.glauca* (L.) L. Rico, all species of *Acaciella* occur in Mexico, and 11 of them are endemic to this country. Species have been recorded from south-eastern United States, throughout Mexico and south to Costa Rica and western Panama in Central America, with scattered records in the Antilles (Cuba, Jamaica and Dominican Republic), and South America (Venezuela, Colombia, Ecuador, Peru, Bolivia and Argentina). The genus is absent in the Amazonian basin (Fig. 200).

#### Ecology.

Sea level to 2500 m elevation, primarily in areas covered by seasonally dry tropical deciduous forests, thorn scrub, semi-desert vegetation, and mixed *Quercus-Pinus* forest.

#### Etymology.

Small *Acacia*, from -*ellus* (suffix) used to form diminutives.

#### Human uses.

In its native range, the leaves of *A.angustissima* are reported to be used as forage for livestock, the roots for tanning, and different parts of the plants are utilised in traditional medicine and for the production of fermented beverages (Rico Arce and Bachman 2006). In addition, due to its high growth-rate and its ability to fix atmospheric nitrogen and accumulate tannins, *A.angustissima* is being tested as a multi-use, agroforestry species (Rico Arce and Bachman 2006; Rincón-Rosales and Gutiérrez-Miceli 2008; Musara and Aladejana 2020). However, in Asia and Australia, due to its high capacity to naturalise, it has been perceived as a potential weed.

#### Notes.

*Acaciella* has traditionally been related to *Acacia* Mill. s.l., and species were grouped into Acaciaser.Filicinae Benth. (Bentham 1842b), regarded by Pedley (1978) as sect. Filicinae. A few years later, Acaciaser.Filicinae was transferred to genus SenegaliaBritton & Rose, assect.Filicinae (Benth.) Pedley (Pedley 1986).

Despite the resemblance of the flowers of *Acaciella* to those of *Acacia*, Rico Arce and Bachman (2006) resurrected *Acaciella* as a distinct genus, based on a combination of morphological traits, essentially the lack of spines and extrafloral nectaries, and 8-grained polyads (Rico Arce and Bachman 2006). Gómez-Acevedo et al. (2010), using morphological and molecular evidence, established *Acaciella* [excl. *A.chamelensis* (L. Rico) L. Rico] as monophyletic and phylogenetically related to *Calliandra* rather than to *Acacia*.

#### Taxonomic references.

Britton and Rose (1928); Rico Arce and Bachman (2006).

### ﻿ Afrocalliandra

Taxon classificationPlantaeFabalesFabaceae

E.R. Souza & L.P. Queiroz, Taxon 62(6): 1213. 2013.

**Figure 201.**
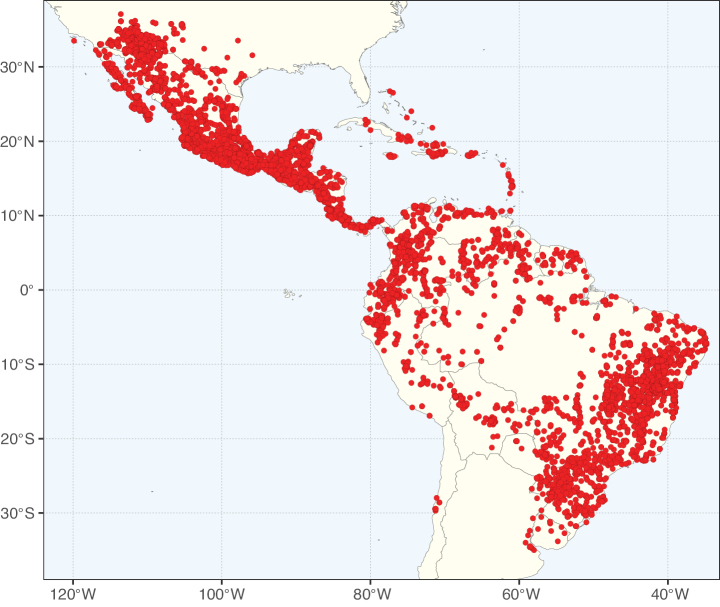
Distribution of *Afrocalliandra* based on quality-controlled digitised herbarium records. See Suppl. material 1 for the source of occurrence data.

Figs 197 , 198 , 199 , 201

#### Type.

*Afrocalliandraredacta* (J.H. Ross) E.R. Souza & L.P. Queiroz [≡ *Acaciaredacta* J.H. Ross]

#### Description.

Shrubs to 2.5 m, densely branched (Fig. 197D), the branches provided with brachyblasts from which leaves and inflorescences emerge, armed with tapering branches becoming spinescent at the tips (*A.gilbertii*) or with spinescent stipules (*A.redacta*). **Stipules** either leafy or modified into long, paired, straight or slightly curved, rigid spines (Fig. 199B), persistent. **Leaves** bipinnate; petioles without extrafloral nectaries; pinnae 1 pair; leaflets 3–9 pairs per pinna, small. **Inflorescences** capitate, pedunculate, homomorphic, few-flowered (Fig. 198C). **Flowers** sessile or subsessile; calyx cup-shaped, 5-merous; corolla 5-merous; stamens 10–34, long exserted from the corolla, with their bases fused forming a conspicuous tube, this slightly exserted above the corolla, creamy white or pink, the anthers eglandular; pollen in (7) 8 (10)-grained acalymmate polyads, bilateral, flattened-ovoid, with the basal grain acutely narrowed, with a rather inconspicuous sticky appendage; stigma capitate (*A.gilbertii*). **Fruits** narrowly oblanceolate, erect, flattened, slightly curved, with thickened margins, the valves thick coriaceous (Fig. 199B), dehiscing elastically along both margins, recurving from apex downwards, 2–4 seeds per fruit. **Seeds** rounded, ca. 5 × 3 mm, compressed, flattened, pointed towards the hilar end, areolate.

#### Chromosome number.

Unknown.

#### Included species and geographic distribution.

Two species distributed allopatrically in distant arid areas of Africa. *Afrocalliandraredacta* is endemic to a restricted area of the Northern Cape Province, across the mountains of the Richtersveld, South Africa, and *A.gilbertii* is found in the Mandera District, Kenya and in adjacent Somalia (Fig. 201).

#### Ecology.

Both species of *Afrocalliandra* occur in arid zones, in areas surrounded by open, shrubby vegetation. *Afrocalliandraredacta* has been reported to grow on rocky slopes of schistoid granite, at 700 m elevation, whereas *A.gilbertii* has been found on sandstone ridges at 450–500 m.

#### Etymology.

African *Calliandra*, from Greek, *calli*- (= beautiful) and -*andrus* (= male), pertaining to the highly attractive stamens.

#### Human uses.

Unknown.

#### Notes.

Based on a nuclear and plastid DNA sequence phylogeny, Souza et al. (2013) transferred to *Afrocalliandra* the two African species originally placed in *Calliandra*. Although the morphology of the African species is similar to some *Calliandra* species inhabiting arid areas of the New World, they differ by some fundamental features, such as the (7) 8 (10)-celled, acalymmate polyads, and the presence of axillary branches or stipules modified into thorns, although both types of armature also occur sporadically within *Calliandra* [spinescent stipules in *C.pauciflora* and *C.haematomma* (Bertero ex DC.) Benth., and spinescent shoots in *C.spinosa*].

Contrasting with the current view to maintain *Calliandra* and *Afrocalliandra* as separate genera, Thulin (2023) recently proposed a return to the circumscription of *Calliandra* as an amphi-Atlantic genus that includes the two African species, here regarded as *Afrocalliandra*. As emphasised by Thulin (2023), *Calliandra* and *Afrocalliandra* are two closely related monophyletic sister taxa that are morphologically not easily separable. Thulin (2023) noted that the two *Afrocalliandra* species may be distinguished morphologically from all of the Neotropical *Calliandra* only by a single microscopic character: the acalymmate polyads, a plesiomorphic character state among mimosoids. However, the calymmate condition in *Calliandra* is rarely found in other Mimoseae and clearly distinguishes this genus from *Afrocalliandra*. An associated structure present in all *Calliandra* species, as well as in *Afrocalliandra*, is the sticky appendage attached to the basal polyad cell. This appendage, which is destroyed in acetolysed material, seems to be rudimentary in *Afrocalliandra* (Thulin et al. 1981, fig. 3G), whereas in the observed *Calliandra* species it is extremely large and evident (see fig. 2 in Hernández 1986). In addition to the differential polyad characters, all available phylogenies show that the *Calliandra* and *Afrocalliandra* clades are supported by long branches reflecting ancient cladogenesis (Suppl. material 2), even longer than the morphologically dissimilar *Acaciella*. Consequently, considering the important differences in polyad structure, the separation of the two genera by long branches in the existing phylogenies, and their geographic separation in Africa and America, we have opted here to keep the two genera separate.

#### Taxonomic references.

Souza et al. (2013); Thulin (1993, 2023); Thulin et al. (1981).

### ﻿ Calliandra

Taxon classificationPlantaeFabalesFabaceae

Benth., J. Bot. (Hooker) 2(11): 138. 1840 nom. cons.

**Figure 202.**
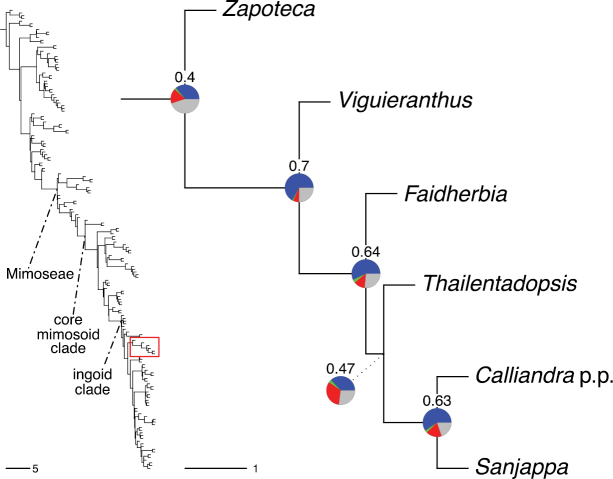
Distribution of *Calliandra* based on quality-controlled digitised herbarium records. See Suppl. material 1 for the source of occurrence data.

Figs 197 , 198 , 199 , 202

Anneslia Salisb., Parad. Lond.: pl. 64. 1807, nom. ut. rej. vs. Calliandra Benth. Type: Annesliafalcifolia Salisb. [≡ Calliandrahoustoniana (Mill.) Standl.]
Clelia Casar., Nov. Stirp. Brasil. Dec. 10: 83. 1845. Type: Cleliaornata Casar. [≡ Calliandraharrisii (Lindl.) Benth.]
Codonandra H. Karst., Fl. Columb. 2: 43, t. 122. 1863. Type: Codonandrapurpurea H. Karst. [≡ Calliandramagdalenae(DC.)Benth.var.magdalenae]
Guinetia L. Rico & M. Sousa, Kew Bull. 54(4): 977. 1999 (publ. 2000). Type: Guinetiatehuantepecensis L. Rico & M. Sousa [≡ Calliandratehuantepecensis (L. Rico & M. Sousa) E.R. Souza & L.P. Queiroz]

#### Type.

*Calliandrahoustoniana* (Mill.) Standl. [≡ *Mimosahoustoniana* Mill.]

#### Description.

Shrubs or small trees (Fig. 197C, E–H), rarely functionally herbaceous or geoxylic subshrubs with an enlarged xylopodium seasonally arising from a perennial root (Fig. 197I–K), usually unarmed, very rarely armed with long shoots tapering at apex into a stout thorn or with spinescent stipules. **Stipules** leafy or rarely spinescent, persistent or caducous. **Leaves** bipinnate, pinnate only in *C.hymenaeoides*; petioles and rachis without extrafloral nectaries; pinnae 1–many pairs; leaflets (1) 2–many pairs per pinnae, small (ca. 5 mm) to up to 12 cm long, size usually being inversely proportional to number of leaflet pairs per pinna. **Inflorescences** capitate or umbelliform (Fig. 198G–L), usually pedunculate, solitary or fasciculate, arising from leaf axils or from brachyblasts, sometimes with the umbels forming a long, terminal pseudoraceme through suppression of distal leaves (Fig. 198D–F); capitula either homomorphic or heteromorphic, the latter bearing 1 to several large, central flowers and numerous small, lateral flowers (Fig. 198H). **Flowers** bracteate, hermaphroditic or functionally staminate; calyx usually cup-shaped to turbinate, rarely hemispherical or inflated, (3) 5 (6)-merous; corolla cup-shaped to tubular, (3) 5 (6)-merous; stamens (8) 10–numerous, long exserted from the corolla, always with the base fused forming a conspicuous tube, this disproportionately long and exserted in the central flowers of heteromorphic inflorescences, red, pink or white, or white in the basal half and pink or red in the distal half, rarely bright yellow (Fig. 198D–L); anthers eglandular; pollen in 8-grained polyads, these calymmate, bilateral, flattened-ovoid, with the basal grain acutely narrowed and bearing a sticky appendage; ovary sessile or short pedicellate, usually 8–12-ovulate; stigma discoid or capitate with a wide area of polyad receptivity. **Fruits** narrowly oblanceolate to linear-oblanceolate, flattened, straight or slightly curved, with thickened margins, the valves usually coriaceous to ligneous, often erect (Fig. 199C–J), dehiscing elastically along both margins, recurving from apex downwards (Fig. 199D). **Seeds** discoid, ovoid or rhomboid, usually compressed, with or without U-shaped pleurogram.

#### Chromosome number.

*n* = 8, 11 (Goldblatt 1981b; Hernández 1986, 1989; Tapia-Pastrana and Uribe-Hernández 2019).

#### Included species and geographic distribution.

Approximately 140 species restricted to the Neotropics, occurring from southern United States (southern Arizona, New Mexico and Texas), throughout Mexico, Central America and South America, south to Uruguay, and northern Argentina and Chile (Fig. 202). Areas of high species concentration and endemism exist in north-eastern Brazil and in western and southern Mexico. A particularly important area of species richness corresponds to the campos rupestres of Serra do Espinhaço in north-eastern Brazil, where ca. 37 endemic species occur, these forming a clade that corresponds to section Monticola E.R. Souza & L.P. Queiroz. Koenen et al. (2013) showed that this clade is recent, dating to the Pliocene and is explained by an acceleration in the rate of species diversification, showing that this campos rupestres clade represents a remarkable example of a recent, rapid evolutionary radiation.

#### Ecology.

Most species occur at low or moderate elevations, in seasonally dry tropical deciduous forests, and less frequently in wet forests, riparian vegetation, tropical savannas, campos rupestres (rupestrian grasslands) from Espinhaço range in eastern Brazil, temperate grasslands, and even in semi-desert or desert vegetation. Seeds disperse over short distances through the explosive dehiscing mechanism. Most Mesoamerican and Mexican species have nocturnal anthesis and are legitimately pollinated by sphingid moths or bats; however, hummingbird pollination in day-flowering species appears to be also common in the genus.

#### Etymology.

From Greek, *calli*- (= beautiful) and -*andrus* (= male), pertaining to the highly attractive stamens.

#### Human uses.

*Calliandracalothyrsus* Meissn. has proved to be an excellent fast-growing, multi-use shrub or small tree as a source of fuelwood and fodder, as well as for soil improvement (Macqueen and Hernández 1997). Some species, most notably *C.haematocephala* (Bertero ex DC.) Benth., *C.californica* Benth. and *C.riparia* Pittier, are planted as ornamentals in urban areas.

#### Notes.

For over a century, after publication of Bentham’s (1875) influential treatment of suborder Mimoseae, *Calliandra* was understood to be a genus from both the New and Old Worlds. However, this concept has been modified over the last three decades. Species of Bentham’s ser. Laetevirentes, together with other species, were segregated into the Neotropical genus *Zapoteca* (Hernández 1986, 1989). Barneby (1998) explicitly restricted *Calliandra* to the New World species in his monograph of the genus, even though he did not assign the Old World species to new segregate genera. His work anticipated the subsequent description of segregate genera that were confirmed by molecular phylogenies. Species from Madagascar were placed in *Viguieranthus* (Villiers 2002). In addition, the continental African species (*C.redacta* and *C.gilbertii*), were transferred to *Afrocalliandra* (Souza et al. 2013), and *C.cynometroides* Beddome, from India, to *Sanjappa* (Souza et al. 2016).

Phylogenetic analyses using morphological and molecular sequence data have shown that all the American species of *Calliandra*, plus the monotypic *Guinetia* L. Rico & M. Sousa, later transferred to *Calliandra* [*C.tehuantepecensis* (L. Rico & M. Sousa) E.R. Souza & L.P. Queiroz], constitute a monophyletic group (Souza et al. 2013). Therefore, *Calliandra*, in this more restricted concept, is a Neotropical genus defined by the following combination of characters: elastically dehiscent pods; 8-grained, bisymmetric, calymmate polyads with a large mucilaginous basal appendage; and atypical (*n* = 8 and 11) chromosome numbers (Guinet and Hernández 1989; Hernández 1989; Barneby 1998).

**Figure 203.**
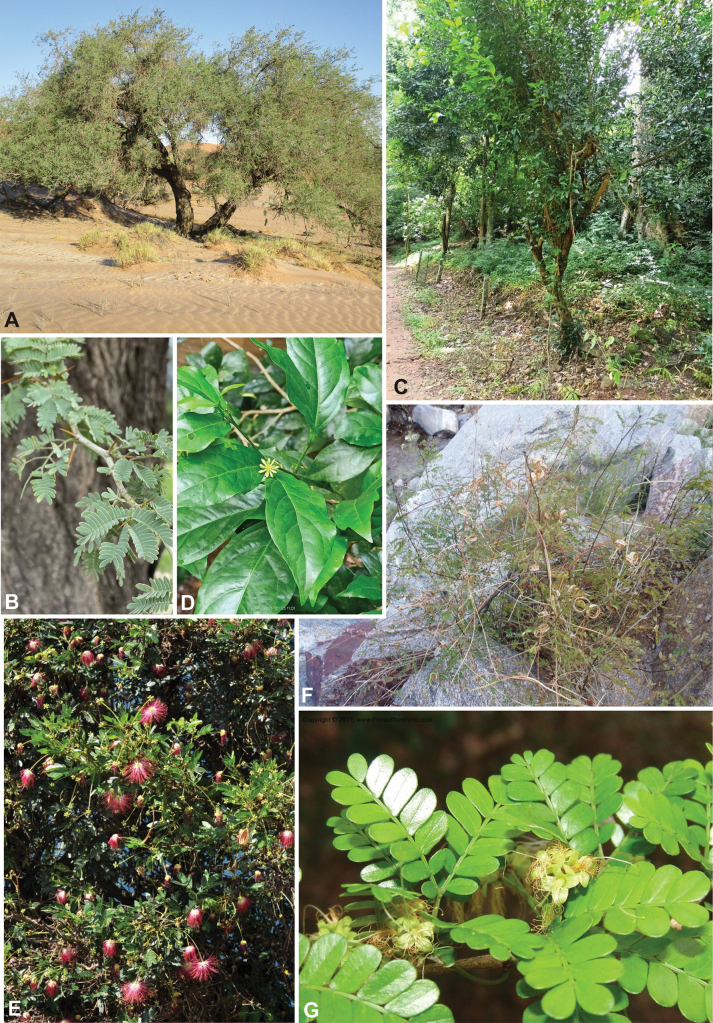
Generic relationships in the Zapoteca clade (tribe Mimoseae). Note that *Calliandra* p.p. represents a single taxon, which is currently unplaced [and is not the type species of *Calliandra* (see tribe notes and footnote on page 382)]. For description of phylogeny and support values, see Fig. 6 caption (page 63).

The generic placement of one Asian species, *C.umbrosa* (Wall.) Benth. from India, Bangladesh and Myanmar, which is still included in *Calliandra*, remains to be evaluated^2^. It is characterised by having spinescent stipules, 3-foliolate pinnae, petioles with extrafloral nectaries, and discoid, 16-grained, acalymmate polyads, and certainly does not belong to *Calliandra* as currently understood. Likewise, an undescribed species of *Calliandra*, excluded from the genus by Barneby (1998), also does not group with the rest of the genus (Fig. 203; Ringelberg et al. 2022), occurring instead as sister to *Sanjappa* E.R. Souza & Krishnaraj in the Zapoteca clade (page 371).

#### Taxonomic references.

Barneby (1998); Macqueen and Hernández (1997); Souza et al. (2013).

## ﻿﻿28. Zapoteca clade

Héctor M. Hernández^22^

Citation: Hernández HM (2024) 28. Zapoteca clade. In: Bruneau A, Queiroz LP, Ringelberg JJ (Eds) Advances in Legume Systematics 14. Classification of Caesalpinioideae. Part 2: Higher-level classification. PhytoKeys 240: 371–383. https://doi.org/10.3897/phytokeys.240.101716

**Zapoteca clade**

Figs 203–210

**Included genera (5).***Faidherbia* A. Chev. (1 species), *Sanjappa* E.R. Souza & M.V. Krishnaraj (1), *Thailentadopsis* Kosterm. (3), *Viguieranthus* Villiers (18), *Zapoteca* H.M. Hern. (22).

**Description.** Shrubs or trees, armed with spinescent stipules or unarmed. **Stipules** leafy or spinescent, persistent or caducous. **Leaves** bipinnate, rarely pinnate, with or without extrafloral nectaries on the petiole, leaf rachis or pinnae; pinnae 1–numerous pairs; leaflets 1–numerous pairs per pinna, opposite or rarely alternate, small (ca. 5 mm) to up to 22 cm long. **Inflorescences** spherical heads, umbels, or elongated spikes or racemes, pedunculate, usually densely-flowered, solitary or fasciculate, arising from leaf axils, sometimes forming long, terminal pseudopanicles, the capitula always homomorphic. **Flowers** hermaphroditic or functionally staminate; calyx cup-shaped, less frequently campanulate, obovoid, obconical or hemispherical, (3–4) 5-lobed; corolla campanulate or infundibuliform, (3–4) 5-lobed; stamens numerous, long exserted from the corolla, to 4.3 cm long, always with the base fused forming a conspicuous tube, this rarely as short as 1 mm, the staminal tube always included in the corolla, usually white, rarely pink or red-purple, or white in the basal half and pink or red in the distal half, anthers eglandular; pollen in 16-grained polyads (32-grained in *Faidherbia*), always acalymmate, discoid, heteromorphic; ovary sessile or shortly stipitate, style filiform, the stigma cup-shaped, capitate or discoid. **Fruits** linear to elliptic, oblanceolate or obovate, flattened or thickened, straight, slightly curved or curled and twisted, with or without thickened margins, the valves membranous, chartaceous, coriaceous or ligneous, brownish or orange when mature, usually but not always dehiscent elastically along both margins, recurving from apex downwards, or indehiscent. **Seeds** ovoid, oblong or rhomboid, with or without a pleurogram.

**Geographic distribution.** The Zapoteca clade displays a pantropical distribution, with *Zapoteca* restricted to tropical and subtropical America, *Viguieranthus* endemic to Madagascar and the Comoro Islands, *Faidherbia* widespread throughout Africa, *Thailentadopsis* restricted to parts of Indochina (Thailand and Vietnam) and Sri Lanka, and *Sanjappa* limited to south-west India.

**Clade-based definition.** The most inclusive crown clade containing *Zapotecacaracasana* (Jacq.) H.M. Hern. and *Sanjappa cynometroides* (Bedd.) E.R. Souza & Krishnaraj, but not *Calliandrahoustoniana* (Mill.) Standl., *Hesperalbiziaoccidentalis* (Brandegee) Barneby & J.W. Grimes or *Pithecellobiumdulce* (Roxb.) Benth. (Fig. 203).

**Notes.** The Zapoteca clade comprises five genera with 45 known species. *Faidherbia*, *Thailentadopsis*, *Sanjappa* and *Viguieranthus* were shown to form a monophyletic group in phylogenetic analyses of Ingeae (Souza et al. 2013, 2016), but resolved as sister to *Zapoteca* only in recent phylogenomic analyses (Koenen et al. 2020a; Ringelberg et al. 2022). The clade is difficult to define morphologically, but in contrast to some other close relatives in Mimoseae, such as some species of *Calliandra* Benth. and *Albizia* Durazz., which have heteromorphic inflorescences, the inflorescences in the Zapoteca clade are always homomorphic. Extrafloral nectaries occur on the leaves of all genera, although these are present in only four species of *Zapoteca*. The stamens are always basally fused to form a tube, this being rather short (± 1 mm) in *Faidherbia*, and anthers are always eglandular. Polyads are universally acalymmate, discoid and heteromorphic, and are 16-grained, with the exception of *Faidherbia* that has 32-grained polyads. Finally, most species appear to occur in seasonally dry deciduous or evergreen forests, and less frequently in savannas and xerophytic plant formations, at low to moderate elevations.

The individual genera may be easily distinguished. Three genera (*Faidherbia*, *Thailentadopsis* and *Sanjappa*) are armed with paired, persistent or caducous, straight, lignescent stipules, whereas *Zapoteca* (excl. *Z.aculeata* Spencer ex Benth.) and *Viguieranthus* are unarmed. The leaves are usually bipinnate, and have a single pair of pinnae in most species of *Viguieranthus*, 1–2 pairs in *Thailentadopsis*, and 1–10 pairs in *Zapoteca* and *Faidherbia*. In contrast, the leaves of *Sanjappa* and two species of *Viguieranthus* (*V.brevipennatus* Villiers and *V.unifoliolatus* Villiers) may be interpreted as pinnate, and are reduced to a single pair of leaflets per leaf; the highly reduced pinnate leaves of these two genera may have been derived from bipinnate-leaved ancestors. The number of leaflets per pinna is also highly variable, and leaflet size is usually inversely proportional to the number of leaflet pairs per pinna.

Fruits of *Zapoteca* and *Sanjappa* are essentially flat and straight, have thickened margins, and are elastically dehiscent, a dehiscing mechanism typical of *Calliandra* species, for example. The fruits of *Viguieranthus* are morphologically similar to those of the two previous genera, but the dehiscing mechanism appears to be passive, rather than active (elastically dehiscent). The submoniliform fruits of *Thailentadopsis* are dehiscent, but also lack the explosive mechanism of *Zapoteca* and *Sanjappa*. Contrasting with the previous four genera, the fruits of *Faidherbia* are indehiscent and markedly different morphologically. In fact, *Faidherbia*, when compared with the other genera in the clade, has the most divergent morphological features (i.e., large trees, spicate inflorescences, short staminal tube, 32-grained polyads, thick, twisted, indehiscent fruits), although sharing the straight stipular spines with *Thailentadopsis* and *Sanjappa*.

As noted in the Calliandra clade (page 358), two Asian species of *Calliandra*, *C.umbrosa* (Wall.) Benth. and a yet undescribed species, are the last remaining of the species excluded from *Calliandra* by Barneby (1998) that have not yet been placed in a segregate genus. Although *C.umbrosa* has yet to be included in a phylogenetic analysis, in the analyses of Ringelberg et al. (2022) the undescribed new species is placed as sister to the monospecific Indian genus *Sanjappa* in the Zapoteca clade (Fig. 203).^3^

### ﻿ Zapoteca

Taxon classificationPlantaeFabalesFabaceae

H.M. Hern., Ann. Missouri Bot. Gard. 73(4): 757. 1986 (publ. 1987).

**Figure 204.**
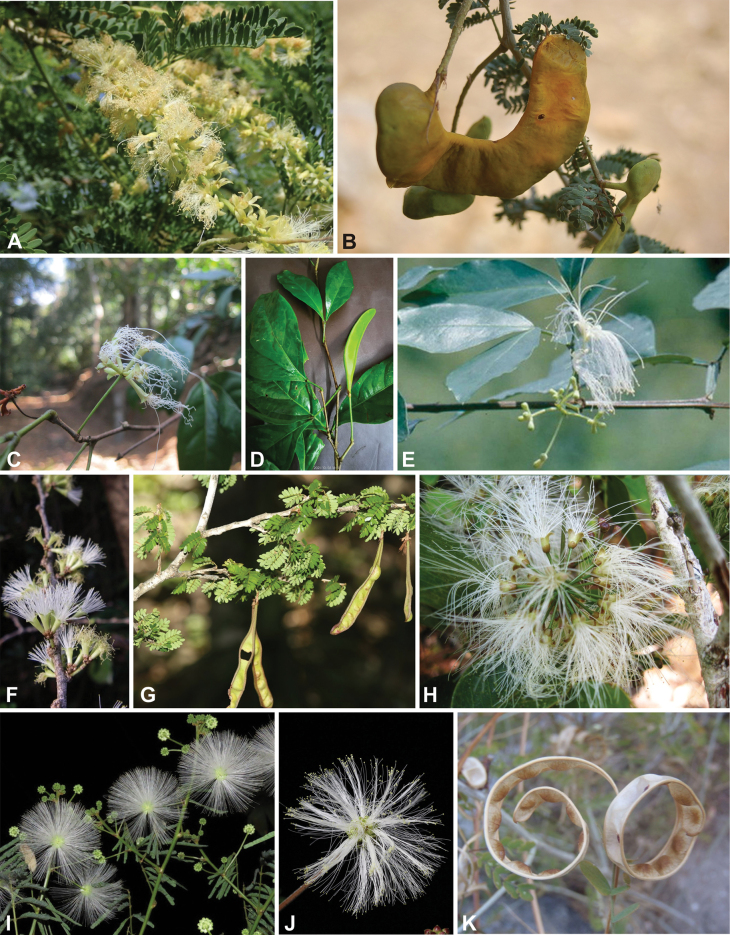
Morphological features of the Zapoteca clade **A, B***Faidherbiaalbida* (Delile) A. Chev. **A** habit **B** leaves **C, D***Sanjappa cynometroides* (Bedd.) E.R. Souza & M.V. Krishnaraj **C** habit **D** pinnate leaves and inflorescence in bud **E***Zapotecaaculeata* (Spruce ex Benth.) H.M. Hern. foliage and inflorescences at anthesis (*Neill 18437*) **F**Zapotecaformosasubsp.schottii (Torr. ex S. Watson) H.M. Hern. habit and dehisced fruits **G***Viguieranthusglaber* Villiers leaves and inflorescences post-anthesis. Photo credits **A** Gobabeb - Namib Research Institute **B** Dave U **C, D** G Suresh **E** D Neill **F** D Beckman **G** C Davidson.

**Figure 205.**
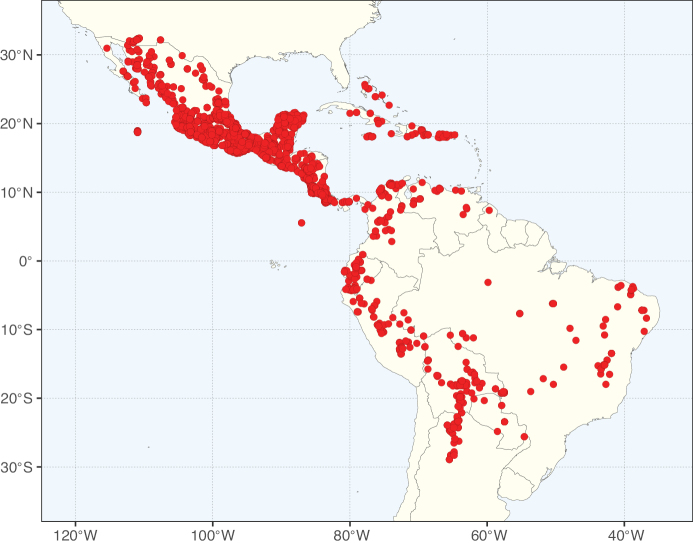
Inflorescence and fruit morphology in the Zapoteca clade **A, B***Faidherbiaalbida* (Delile) A. Chev. **A** inflorescences at anthesis **B** indehiscent fruit **C, D***Sanjappa cynometroides* (Bedd.) E.R. Souza & M.V. Krishnaraj **C** inflorescence post-anthesis **D** pinnate leaves and immature fruit **E***Thailentadopsistenuis* (Craib) Kosterm. inflorescence **F***Viguieranthusperrieri* (R. Vig.) Villiers inflorescence at anthesis **G***Viguieranthusalternans* (Benth.) Villiers foliage and fruits before dehiscence **H***Viguieranthusbrevipennatus* Villiers inflorescence at anthesis (*Randrianarivony 175*) **I***Zapotecatetragona* (Willd.) H.M. Hern. inflorescence at anthesis **J**Zapotecaportoricencis(Jacq.)H.M. Hern.subsp.portoricensis inflorescence at anthesis (*Amith 74438*) **K**Zapotecaformosasubsp.schottii (Torr. ex S. Watson) H.M. Hern. dehisced fruit. Photo credits **A** S Piry **B** B Adkins **C, D** G Suresh **E**https://marketingoemoffice.com/Data_Pithecellobium.html**F** F Ratovoson **G** F Rakotoarivony **H** T Randrianarivony **I** FJ Gómez Marín **J** J Amith **K** D Beckman.

**Figure 206.**
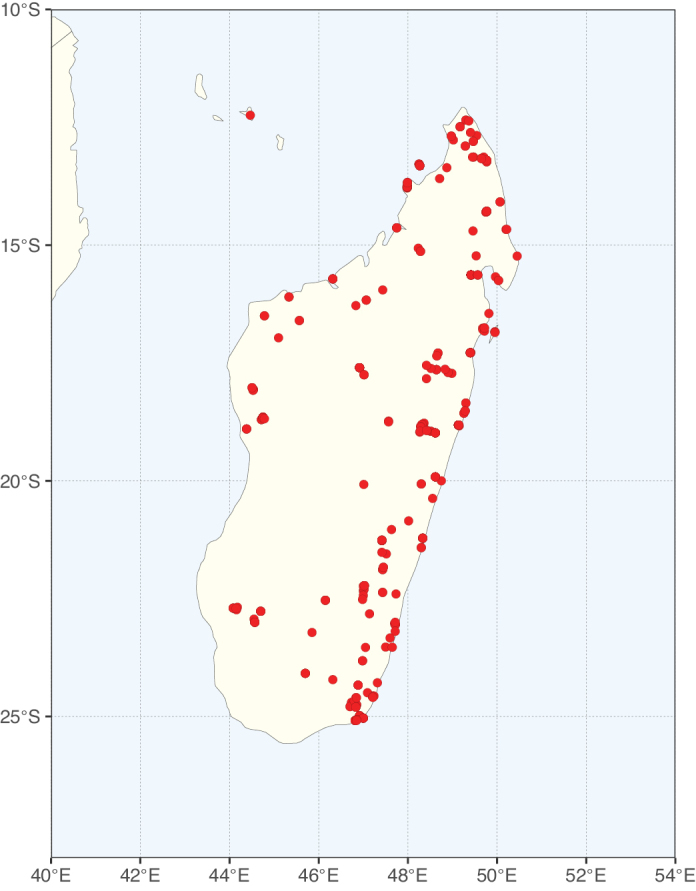
Distribution of *Zapoteca* based on quality-controlled digitised herbarium records. See Suppl. material 1 for the source of occurrence data.

Figs 204 , 205 , 206

Calliandra ser. Laetevirentes Benth., London J. Bot. 3: 97. 1844. Type not designated.

#### Type.

*Zapotecatetragona* (Willd.) H.M. Hern. [≡ *Acaciatetragona* Willd.]

#### Description.

Erect, suberect or scandent shrubs, rarely small trees, usually unarmed, very rarely armed with spinescent stipules. **Stipules** leafy or rarely spinescent, usually persistent. **Leaves** bipinnate, usually without extrafloral nectaries, but 3 (4) species with cup-shaped or cylindrical glands on the rachis or pinnae or near the base of the petiole; pinnae 1–numerous pairs; leaflets (1) 2–numerous pairs per pinna, opposite, small (ca. 5 mm) to up to 22 cm long. **Inflorescences** capitate, spherical in bud and at anthesis, homomorphic, pedunculate, densely-flowered, solitary or fasciculate, arising from leaf axils, sometimes forming long, terminal pseudopanicles. **Flowers** bracteate, hermaphroditic or functionally staminate; calyx cup-shaped, dentate or denticulate; corolla campanulate or infundibuliform, the petals valvate in bud, usually revolute at anthesis; stamens ca. 30–60, long exserted from the corolla, 1.9–4.3 cm, always with the base fused forming a conspicuous tube, the staminal tube always included in the corolla, white, pink or red-purple, or white in the basal half and pink or red in the distal half, anthers eglandular; pollen in 16-grained polyads, acalymmate, discoid, heteromorphic, nearly always with eccentric lens-shaped thickenings on the central cells on one side of the polyad; ovary sessile or shortly stipitate, usually 10–15-ovulate, style filiform, with the stigma cup-shaped. **Fruits** usually pendent, linear, flattened, straight or rarely slightly curved, with thickened margins, the valves membranous or rarely coriaceous, dehiscing elastically along both margins, recurving from apex downwards. **Seeds** ovoid or rhomboid, usually with irregular or U-shaped pleurogram.

#### Chromosome number.

*n* = 13 (Hernández 1986, 1989).

#### Included species and geographic distribution.

Twenty-two described species, all of which are restricted to the Neotropics. Its distribution extends from southern United States (southern Arizona and Texas), throughout Mexico, Central America and South America, south to Paraguay and northern Argentina, including the West Indies and the Revillagigedo Islands (Fig. 206). Mexico, especially the southern portion of the country, includes the richest areas in terms of species number and endemism, with 12 species, eight of which are endemic. Six species (two endemic) are distributed in Central America, and four species (one endemic) occur in the West Indies. South America harbours ten species, three of which are endemic to the Andean basin, and a further three are restricted to isolated areas in the northern Andes (Colombia, Ecuador and Peru).

#### Ecology.

Populations occur from sea-level to 2850 m, although most species are found at low or moderate elevations, in areas covered by seasonally dry tropical deciduous forests, and less frequently in wet forests, and even in desert or semi-desert vegetation. Seed dispersal covers short distances through the explosive dehiscing mechanism. Settling moths of the families Noctuidae, Pyralidae and Geometridae are confirmed to be the primary pollinators (Hernández 1989).

#### Etymology.

Named after the Zapotec ethnic group of Oaxaca, Mexico, where a significant number of taxa occur.

#### Human uses.

No significant uses are known, although Z.formosa(Kunth)H.M. Hern.subsp.formosa is reported to be potentially important as a forage resource for sheep (Newman et al. 1999).

#### Notes.

Detailed morphological and cytological observations supported the recognition of *Zapoteca* as a genus distinct from *Calliandra* by consistently having spherical, homomorphic, capituliform inflorescences; 16-grained, acalymmate, discoid polyads, with lens-shaped thickenings in the central grains [excluding *Z.nervosa* (Urban) H.M. Hern.]; cup-shaped stigmas; and a basic chromosome number of *x* = 13. In contrast, the Neotropical species of *Calliandra* are characterised by having spherical, obconic, capituliform, umbelliform or racemose, homomorphic or heteromorphic inflorescences; capitate or fungiform stigmas; 8-grained, bisymmetric, calymmate polyads with a mucilaginous basal appendage; and atypical (*n* = 8 and 11, rather than the *x* = 13 of closely related genera) chromosome numbers (Hernández 1986, 1989; Guinet and Hernández 1989; Barneby 1998).

The pattern of differentiation within *Calliandra* s.l. sensu Bentam (1875) provided the basis for the segregation of the species in ser. Laetevirentes, together with two other species placed by Bentham in his ser. Macrophyllae (*C.aculeata* and *C.amazonica* Benth.), into the new genus *Zapoteca* (Hernández 1986, 1989). More than two decades later, in a morphological and molecular based phylogenetic analysis of *Calliandra*, Souza et al. (2013) supported the validity of *Zapoteca* as a separate genus and confirmed its monophyletic status. More recently, in a phylogenetic analysis of *Zapoteca*, based on nuclear and plastid DNA sequence data, Ferm (2019) and Ferm and Ståhl (2022), ratified the monophyly of *Zapoteca* and suggested some taxonomic rearrangements to the Hernández (1989) subgeneric classification.

#### Taxonomic references.

Ferm (2019); Ferm and Ståhl (2022); Hernández (1986, 1989).

### ﻿ Viguieranthus

Taxon classificationPlantaeFabalesFabaceae

Villiers, Legum. Madagascar: 271. 2002.

**Figure 207.**
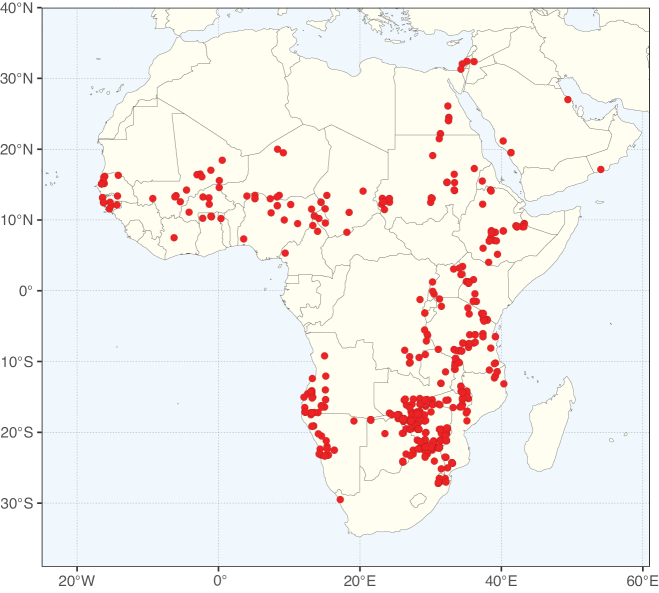
Distribution of *Viguieranthus* based on quality-controlled digitised herbarium records. See Suppl. material 1 for the source of occurrence data.

Figs 204 , 205 , 207

#### Type.

*Viguieranthusalternans* (Benth.) Villiers [≡ *Calliandraalternans* Benth.]

#### Description.

Unarmed shrubs or trees to 25 m, with trunks up to 80 cm diameter. **Stipules** coriaceous, not spinescent, more or less persistent. **Leaves** bipinnate, rarely pinnate; petioles with an extrafloral nectary at the apex, sometimes also present on the rachis tips, sometimes the petioles and rachis with narrowly winged margins; pinnae of bipinnate leaves 1 pair; leaflets alternate to opposite, sometimes 1 or 3 per pinna, 1 pair per leaf in pinnate leaves, up to 7.7 × 4.6 cm, but frequently more numerous (to 34 per pinna), and smaller (9–35 × 2–15 mm). **Inflorescences** spherical heads, elongate spikes or racemes, pedunculate, solitary or fasciculate, arising from leaf axils, sometimes organised in pseudopanicles, homomorphic. **Flowers** hermaphrodite; calyx usually cup-shaped, less frequently broadly obovoid, obconical or hemispherical, 4–5-lobed; corolla obconical, with the petals connate into a tube, 4–5-lobed; stamens numerous, long exserted from the corolla, white, always with the base fused forming a tube inserted in the corolla, anthers eglandular; pollen in 16-grained polyads, acalymmate, discoid, heteromorphic, without eccentric lens-shaped thickenings on the central cells; ovary sessile or short-pedicellate; stigma funnel-shaped or capitate. **Fruits** linear-oblanceolate, linear-elliptic, or linear-obovate to narrowly obovate, flattened, straight or slightly curved, with thickened margins, the valves chartaceous, coriaceous or ligneous, dehiscing along both margins. **Seeds** ovoid, oblong or rhomboid, without a pleurogram.

#### Chromosome number.

Unknown.

#### Included species and geographic distribution.

Eighteen species occurring in Madagascar, all endemic (except *V.subauriculatus* Villiers also recorded from Comoro Islands) (Fig. 207).

#### Ecology.

Most species are reported from the eastern, humid, evergreen forests in Madagascar. However, some species grow in areas covered by dry deciduous woodland or xerophytic scrubland. Populations are reported from sea level to 1600 m (Villiers 2002).

#### Etymology.

Named after René Viguier (1880–1931), French botanist.

#### Human uses.

The wood of some species is reported to be used as firewood, and for house construction and joinery (Villiers 2002).

#### Notes.

In the original description of *Viguieranthus*, published in The Leguminosae of Madagascar one year after J.F. Villiers’ death (Villiers 2002), it was mentioned that the genus comprises 23 species distributed in Madagascar and Asia. However, only 18 species were considered, including new combinations and new species. As concluded by Souza et al. (2016), the five remaining species not considered by Villiers correspond to five Asian species: three currently recognised under *Thailentadopsis* (Lewis and Schrire 2003); to *Calliandracynometroides* Bedd., later transferred to *Sanjappa* (Souza et al. 2016); and *C.umbrosa* (Wall.) Benth., one species from India, Bangladesh and Myanmar. The latter species certainly does not belong to *Calliandra* in its current concept (see Calliandra clade, page 358), and its generic placement is awaiting critical evaluation^4^.

In light of the previous considerations, *Viguieranthus* is a Madagascan genus, characterised by constantly having leafy (not spinescent) stipules; bipinnate leaves with only 1 pair of pinnae; a variable number of opposite or alternate pairs of leaflets per pinna, sometimes reduced to one leaflet per pinna (*V.unifoliolatus* Villiers and *V.brevipennatus* Villiers); and homomorphic inflorescences (Villiers 2002). The polyads, described as Type A polyads by Guinet and Hernández (1989), are 16-grained, acalymmate, discoid, heteromorphic, and lack the eccentric, lens-shaped thickenings on the central cells, characteristic of *Zapoteca*. *Viguieranthus* is probably a monophyletic genus (further sampling is required; Souza et al. 2013, 2016) and sister to a clade that includes *Faidherbia*, *Sanjappa* and *Thailentadopsis* (Ringelberg et al. 2022).

#### Taxonomic references.

Villiers (2002).

### ﻿ Faidherbia

Taxon classificationPlantaeFabalesFabaceae

A. Chev., Rev. Bot. Appl. Agric. Trop. 14: 876. 1934.

**Figure 208.**
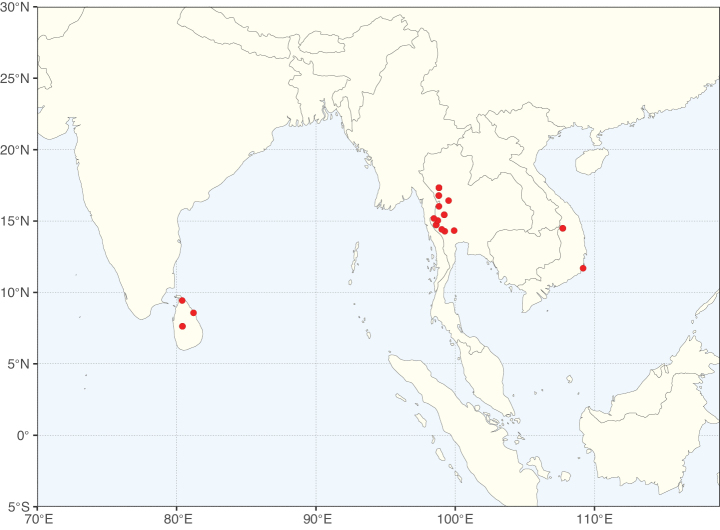
Distribution of *Faidherbia* based on quality-controlled digitised herbarium records. See Suppl. material 1 for the source of occurrence data.

Figs 204 , 205 , 208

#### Type.

*Faidherbiaalbida* (Dalile) A. Chev. [≡ *Acaciaalbida* Dalile]

#### Description.

Trees to 30 m, with trunks up to 2 m diameter, the spreading branches forming a rounded crown, armed with spinescent stipules. **Stipules** spinescent, paired, persistent, straight or slightly curved, to 2 (3.3) cm long. **Leaves** bipinnate, cup-shaped extrafloral nectaries on the leaf rachis at the junction of each pair of pinnae, absent on the petiole; pinnae (2) 3–10 pairs; leaflets 6–23 pairs per pinna, opposite, small (3.5–9 mm long), linear or linear-oblong to slightly obovate-oblong. **Inflorescences** spicate, pedunculate, solitary, arising from leaf axils, collectively forming a terminal pseudopanicle. **Flowers** hermaphrodite; calyx cup-shaped, 5-lobed; corolla campanulate, pale pink inside, the 5 lobes divided almost to the base; stamens 35–55, long exserted from the corolla, with the base fused for ± 1 mm forming a short tube, yellowish-white to pale cream, anthers eglandular; pollen organised in 32-grained polyads, these acalymmate, subcircular, flattened; ovary short-stipitate. **Fruits** thick, flattened, falcate or conspicuously curled and twisted, bright orange when mature, indehiscent. **Seeds** elliptic-lenticular, compressed, with an elliptic pleurogram.

#### Chromosome number.

2*n* = 26 (Goldblatt 1981b; Bukhari 1997).

#### Included species and geographic distribution.

Monospecific (*F.albida*), widespread in Africa, especially across eastern Africa, from Egypt southwards into Sudan, Ethiopia, Somalia, Kenya, Uganda, Tanzania, Mozambique, Zambia and Zimbabwe, to the Transvaal, South Africa. Also reported from Mauritania, Senegal and The Gambia, and in Angola southwards to Botswana (Fig. 208). Also occurring in parts of the Middle East and Arabia, and probably introduced in west Asia (India and Pakistan).

#### Ecology.

Found predominantly in seasonally dry tropical climates, in forests, woodlands and especially in savannas where it is often abundant, on alluvial soils, along seasonal watercourses and around lakes and swamps, from near sea level to 2600 m elevation. It shows an unusual reverse phenology being characteristically leafless during the rainy season. *Faidherbiaalbida* is reported to have a rather generalist reproductive system (Gassama-Dia et al. 2003). Pollination is both entomophilous and anemophilous, and predominantly allogamous; individuals are primarily self-incompatible, although partially self-compatible genotypes exist. The ripe indehiscent fruits are eagerly consumed by large herbivores including livestock facilitating extensive endozoochorous seed dispersal (Barnes and Fagg 2003).

#### Etymology.

Named after the French Major Louis Léon César Faidherbe (1818–1889), Governor of Senegal and founder of Dakar.

#### Human uses.

The trunks are used to make canoes, and the wood is employed as firewood, and for handicrafts; various medicinal uses are reported. The leaves and fruits are used as fodder for domestic animals, and are also consumed by camels and elephants (Barnes and Fagg 2003).

#### Notes.

Originally described as *Acaciaalbida* Dalile, this species has several unusual characters as compared with the other African species of *Acacia* Mill., such as the lack of petiolar extrafloral nectaries, these being present only at the junction of each pair of pinnae, the basally connate filaments forming a short tube (in line with most members of the ingoid clade and all members of the Zapoteca clade), the eglandular anthers, and the 32-grained polyads (Kordofani and Ingrouille 1992). This combination of morphological characters places *F.albida* apart from the African *Vachellia* and *Senegalia*.

#### Taxonomic references.

Brenan (1959, 1970).

### ﻿ Thailentadopsis

Taxon classificationPlantaeFabalesFabaceae

Kosterm., Ceylon J. Sci., Biol. Sci. 12(2): 131. 1977.

Figs 205 , 209

#### Type.

*Thailentadopsistenuis* (Craib) Kosterm. [≡ *Pithecellobiumtenue* Craib]

#### Description.

Shrubs or small trees up to 7 m tall, the trunk to 15 cm diameter, armed with spinescent stipules. **Stipules** spinescent, paired, persistent, lignescent. **Leaves** bipinnate; stalked extrafloral nectaries between each pair of pinnae and usually between each pair of leaflets; petioles, leaf and pinnae rachides winged or not; pinnae 1–2 pairs; leaflets 1–6 pairs per pinna, opposite, increasing in size from base to apex, small to medium sized. **Inflorescences** capitate or umbellate, pedunculate, few-flowered, fasciculate, arising from leaf axils, sometimes forming terminal pseudopanicles, homomorphic. **Flowers** hermaphroditic; calyx campanulate; corolla campanulate or infundibuliform; stamens numerous, long exserted from the corolla, always with the base fused forming a conspicuous tube, the staminal tube always included in and equal in length to the corolla, white, anthers eglandular; pollen in 16-grained polyads, acalymmate, discoid, heteromorphic. **Fruits** submoniliform, flattened, usually with the interseminal spaces constricted, straight or markedly curved, with thickened margins, the valves coriaceous, dehiscent. **Seeds** ovoid, with ovate pleurogram.

#### Chromosome number.

Unknown.

#### Included species and geographic distribution.

**Figure 209.**
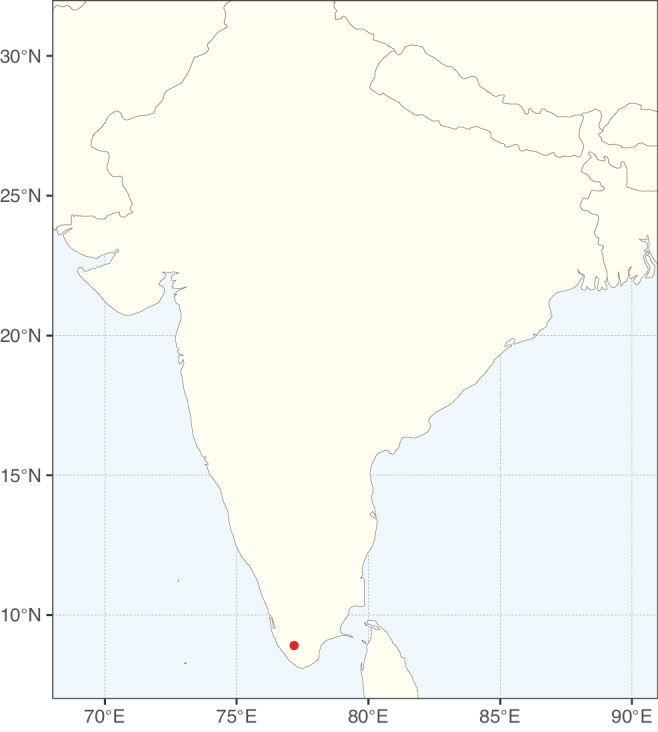
Distribution of *Thailentadopsis* based on quality-controlled digitised herbarium records. See Suppl. material 1 for the source of occurrence data.

Three species allopatrically distributed in Sri Lanka, Thailand and Vietnam (Fig. 209).

#### Ecology.

Populations are reported from 100 to 900 m elevation, in evergreen and open savanna forest, in shaded areas by streams or along rivers, on soils derived from limestone or granitic bedrock.

#### Etymology.

From Thailand (home of the type species), *Entada* (a mimosoid genus, for the superficial similarity of the fruits), and -*opsis* (Greek = similar to).

#### Human uses.

Unknown.

#### Notes.

The three species currently recognised within *Thailentadopsis* have been placed in different genera, including *Acacia*, *Painteria* Britton & Rose and *Pithecellobium* Mart., and Nielsen (1981a, 1992) suggested relationships with *Havardia* Small. The three Asian species are now placed in the resurrected *Thailentadopsis* (Lewis and Schrire 2003). The phylogenetic analyses of Souza et al. (2013) based on morphological and molecular data recovered *Thailentadopsis* as sister to a *Viguieranthus-Zapoteca* clade but phylogenomic analyses supported it as sister to the Asian genus *Sanjappa* (Ringelberg et al. 2022).

#### Taxonomic references.

Lewis and Schrire (2003); Lewis and Rico Arce (2005).

### ﻿ ﻿Sanjappa

Taxon classificationPlantaeFabalesFabaceae

E.R. Souza & M.V. Krishnaraj, Rheedea 21(1): 6. 2016.

**Figure 210.**
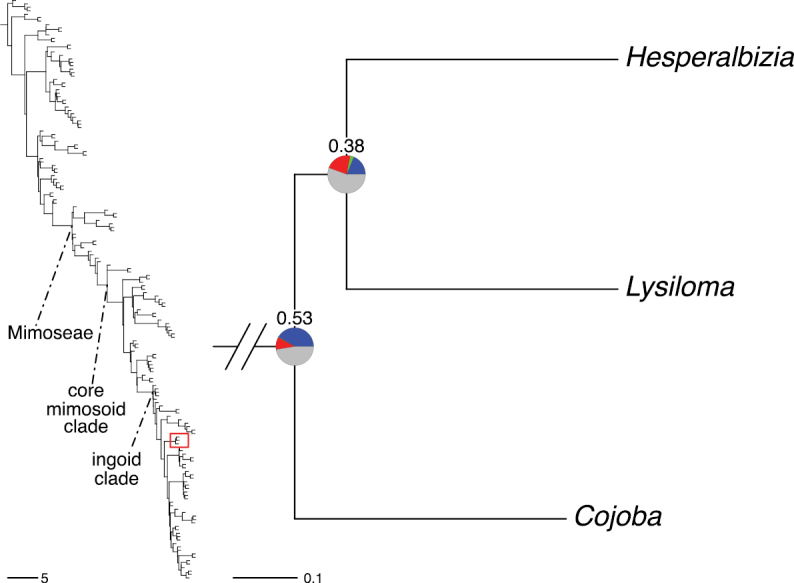
Distribution of *Sanjappa* based on quality-controlled digitised herbarium records. See Suppl. material 1 for the source of occurrence data.

Figs 204 , 205 , 210

#### Type.

*Sanjappa cynometroides* (Bedd.) E.R. Souza & M.V. Krishnaraj [≡ *Calliandracynometroides* Bedd.]

#### Description.

Small trees to 6 m, the trunk to 35 cm diameter, armed with spinescent stipules. **Stipules** spinescent, paired, unequal, straight, lignescent, caducous. **Leaves** pinnate; extrafloral nectaries circular, slightly raised, at the tip of petiole near the junction of the leaflets; leaflets 1 pair per leaf, opposite, relatively large (ca. 5–12 cm long). **Inflorescences** capitate, pedunculate, 7–15-flowered, solitary, arising from leaf axils, homomorphic. **Flowers** hermaphroditic; calyx cup-shaped, dentate, 3-lobed; corolla tubular-campanulate, the 3 lobes usually revolute at anthesis; stamens long-exserted from the corolla, to 2.4 cm long, with their bases fused forming a conspicuous tube, the staminal tube always included in the corolla, white, anthers eglandular; pollen in 16-grained polyads, acalymmate, discoid, heteromorphic; ovary sessile, 5–7-ovulate, style filiform, with the stigma discoid. **Fruits** oblanceolate, flattened, straight or slightly undulate marginally, with thickened margins, rigidly coriaceous, dehiscing elastically along both margins, recurving from apex downwards. **Seeds** rhomboid, with a rhomboid pleurogram.

#### Chromosome number.

Unknown.

#### Included species and geographic distribution.

Monospecific^5^ (but see notes), *S.cynometroides*, endemic to the State of Kerala, south-west India (Fig. 210).

#### Ecology.

*Sanjappa cynometroides* has been found growing in evergreen and sub-evergreen forest areas, near streams, at 300–1100 m elevation. Individuals are reported to occur at very low densities in natural populations (Souza et al. 2016).

#### Etymology.

Named after Dr. Munivenkatappa Sanjappa, Senior Scientist at the Botanical Garden, University of Agricultural Sciences, Bengaluru, India.

#### Human uses.

Unknown.

#### Notes.

*Sanjappa* was described to accommodate one Old World species of *Calliandra* sensu Bentham (1875) that did not fit in any of the segregates described or resurrected over the last few decades (*Faidherbia*, *Thailentadopsis* and *Viguieranthus*).

*Sanjappa* is nested with high support in a clade with *Faidherbia* and *Thailentadopsis*, characterised by the conspicuous spinescent stipules (Souza et al. 2016; Ringelberg et al. 2022), although similar structures are also found in one species of *Zapoteca* (*Z.aculeata*). Following reduction of Bentham’s (1875) broad trans-continental circumscription of *Calliandra* to just the New World species by Barneby (1998), only two Old World species remain to be resolved (see Calliandra clade, page 358), including a new Asian *Calliandra* species [*Poilane 9150*; Ringelberg et al. (2022) in Suppl. Mat.], which is resolved as sister to *Sanjappa* and needs to be evaluated to determine its proper generic placement^6^.

#### Taxonomic references.

Souza et al. (2016).

## ﻿﻿29. Cojoba clade

Rodrigo Duno de Stefano^13^

Citation: Duno de Stefano R (2024) 29. Cojoba clade. In: Bruneau A, Queiroz LP, Ringelberg JJ (Eds) Advances in Legume Systematics 14. Classification of Caesalpinioideae. Part 2: Higher-level classification. PhytoKeys 240: 384–390. https://doi.org/10.3897/phytokeys.240.101716

**Cojoba clade**

Figs 211–215

**Included genera (3).***Cojoba* Britton & Rose (13–19 species), *Hesperalbizia* Barneby & J.W. Grimes (1), *Lysiloma* Benth. (8).

**Description.** Unarmed trees and arborescent shrubs, either micro- or macrophyllidious. **Stipules** small, ephemeral, caducous, rarely persistent or lignescent. **Leaves** bipinnate, rarely paripinnate; extrafloral nectaries sessile; leaflet venation simple, weakly pinnate, pinnate, or palmate-pinnate. **Inflorescences** capitula arising either singly or fasciculate, spherical, receptacle subglobose, rarely spicate-racemose. **Flowers** sessile, homomorphic or nearly so, the central flower sometimes a little stouter, but not longer than the others, 5-merous; calyx campanulate, 5-veined or nearly without veins, very shortly toothed; corolla tubular or very narrowly trumpet-shaped; internally lacking callous and nectar disk; stamens numerous, joined at the base, exserted from corolla; ovary sessile or subsessile, linear-ellipsoid. **Fruits** variable, more or less pendulous, generally linear to broad-linear, plano-compressed, or cylindrical, straight, retrofalcate or spirally contorted, scarcely or deeply constricted between seeds, dehiscent through one or both sutures, but craspedial or indehiscent in *Lysiloma*, and framed by prominent sutures, and the exocarp exfoliating, valves fleshy, leathery, stiffly papery to papery-crustaceous. **Seeds** variable, compressed to flattened, subglobose, discoid, oblong-ellipsoid to ellipsoid.

**Distribution.** Neotropics: Mexico, Central America, South America, and Greater Antilles.

**Figure 211.**
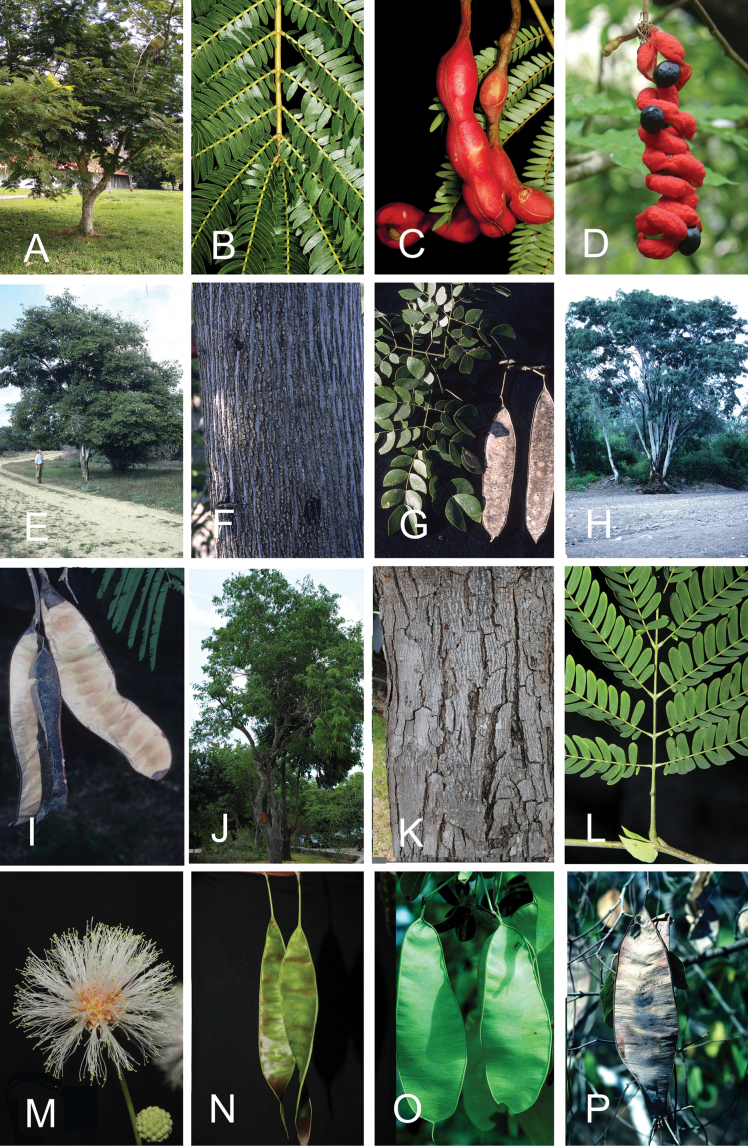
Generic relationships in the Cojoba clade (tribe Mimoseae). For description of phylogeny and support values, see Fig. 6 caption (page 63).

**Notes.** The Cojoba clade was first recognised in the phylogenetic analyses of Iganci et al. (2015), and the relationships were later supported in the analyses of Koenen et al. (2020a), Duno et al. (2021) and Ringelberg et al. (2022). In their treatment of tribe Ingeae, Lewis and Rico Arce (2005) had placed *Cojoba* in the informal Inga alliance, *Hesperalbizia* in the Samanea alliance, and *Lysiloma* was left unplaced. Each genus shows notable differences in vegetative, fruit and seed characters and the floral characters are common to many other genera of ingoid Mimoseae. The three genera can be readily differentiated by their distinctive fruits: *Cojoba* has a cylindrical, spirally contorted or retrofalcate, weakly or more strongly moniliform fruit with fleshy, usually bright red valves, most species of *Lysiloma* have craspedial fruits often with a distinctive exfoliating papery exocarp, and *Hesperalbizia* has flattened, papery fruits closely similar to those of many species of *Pseudalbizzia* Britton & Rose and *Albizia* Durazz. No unique diagnostic characters or synapomorphies are known for this clade.

**Clade based definition.** The most inclusive crown clade containing *Hesperalbiziaoccidentalis* (Brandegee) Barneby & J.W. Grimes and *Cojobaarborea* (L.) Britton & Rose, but not *Zapotecacaracasana* (Jacq.) H.M. Hern., *Pithecellobiumdulce* (Roxb.) Benth. or *Calliandrahoustoniana* (Mill.) Standl. (Fig. 211).

Within the Cojoba clade, *Lysiloma* and *Hesperalbizia* are supported as sister genera which share plano-compressed fruits, as opposed to the cylindrical fruits of *Cojoba*, and these two sister genera are apparently genetically weakly divergent as evidenced by lack of robust support in species-level phylogenies (Duno et al. 2021).

### ﻿ Hesperalbizia

Taxon classificationPlantaeFabalesFabaceae

Barneby & J.W. Grimes, Mem. New York Bot. Gard. 74(1): 112. 1996.

Figs 212 , 213

#### Type.

*Hesperalbiziaoccidentalis* (Brandegee) Barneby & J.W. Grimes [≡ *Albiziaoccidentalis* Brandegee]

#### Description.

Unarmed trees 20 (30) m, bark pale silvery grey. **Stipules** small, membranous, caducous, cordate at base. **Leaves** bipinnate; extrafloral nectaries below mid-petiole, sometimes close to the base, sessile, round or elliptical, shallowly concave, commonly smaller ones at the tip of the petiole and the rachis of the pinnae; pinnae 3–8 pairs; leaflets 5–10 pairs per pinna, venation pinnate. **Inflorescence** 5–20-flowered capitula, receptacle subglobose, spherical, arising either singly or fasciculate. **Flowers** sessile, homomorphic, 5-merous; calyx campanulate, lobes ovate or short-deltate; corolla narrowly vase-shaped; stamens 52–76, 20–24 mm long, joined at base into a well exserted, 5–11 mm long tube; pollen in 16-celled polyads, more or less isodiametric; ovary linear-elliptic, glabrous, stipitate, style shortly exserted, slightly dilated at tip. **Fruits** solitary or paired, broad-linear plano-compressed, long-stipitate, 8–12 (13)-seeded, inertly dehiscent through both sutures. **Seeds** transverse, compressed discoid or oblong-ellipsoid, areolate, funicle filiform; testa crustaceous, brown, and lustrous; pleurogram closed.

**Figure 212.**
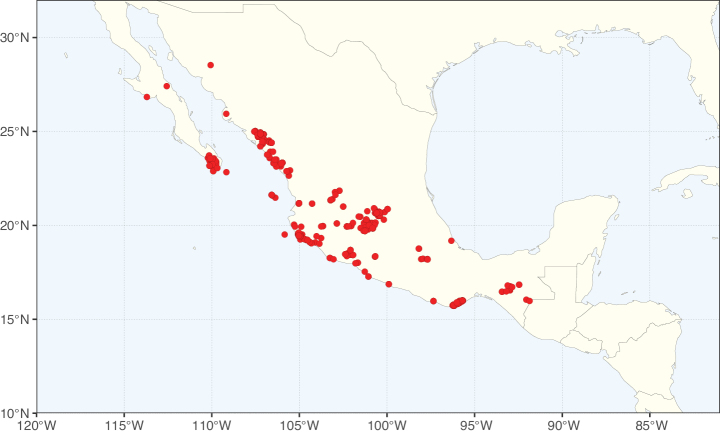
Morphology of Cojoba clade **A–C***Cojobaarborea* (L.) Britton & Rose **A** habit **B** leaves **C** fruit **D***Cojobagraciliflora* (S.F. Blake) Britton & Rose in Mexico, fruit **E–G***Hesperalbiziaoccidentalis* (Brandegee) Barneby & J.W. Grimes **E** habit **F** trunk **G** leaves and fruits **H***Lysilomacandidum* Brandegee, habit **l***Lysilomaacapulcense* (Kunth) Benth. fruit **J–N***Lysilomalatisiliquum* (L.) Benth. **J** habit **K** trunk **L** leaves and stipule **M** flowers **N** fruit **O, P***Lysilomatergeminum* Benth., fruits. Photo credits **A–C** GA Romero González **D, J–N** R Duno de Stefano **E–I, O, P** CE Hughes.

#### Chromosome number.

2*n* = 26 (Rico Arce 1992).

#### Included species and geographic distribution.

**Figure 213.**
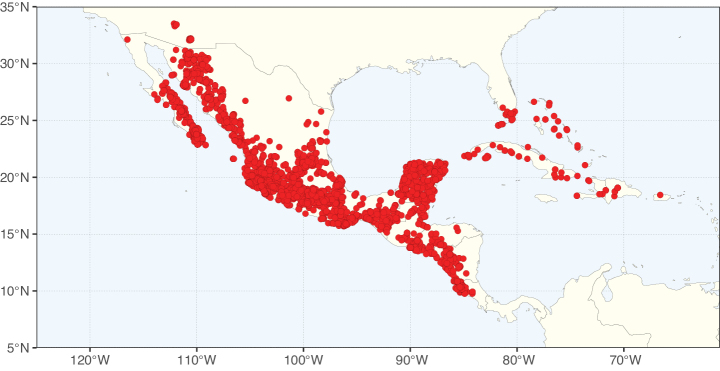
Distribution of *Hesperalbizia* based on quality-controlled digitised herbarium records. See Suppl. material 1 for the source of occurrence data.

Monospecific (*H.occidentalis*), widely distributed in western and southern Mexico (Fig. 213).

#### Ecology.

Largely confined to seasonally dry tropical deciduous forests and woodlands, from sea level to 1500 m elevation, locally abundant in tropical and marginally subtropical western Mexico. Strongly deciduous, fruits long-retained on leafless trees, flowering preceding or coinciding with new leaf flush at the end of the dry season. Seed dispersal passive.

#### Etymology.

Greek = *hesperos*, the evening star, and by extension the West, plus the generic name *Albizia* in reference to the westerly distribution of *H.occidentalis* within Mexico and its previous placement in the genus *Albizia*.

#### Notes.

The plano-compressed, stiffly papery fruits of *H.occidentalis* are very similar to the fruits of some species of *Pseudalbizzia* and *Albizia*, from which *Hesperalbizia* was segregated by Barneby and Grimes (1996). However, recent phylogenies have demonstrated that *Hesperalbizia* is not closely related to *Albizia* or *Pseudalbizzia*, but is instead sister to *Lysiloma* (Duno et al. 2021; Peraza et al. 2022; Ringelberg et al. 2022). *Hesperalbizia* can be distinguished from species of *Pseudalbizzia* and *Albizia* by the absence of a modified central flower, leaflets with pinnate (not palmate-pinnate) venation, peripheral flowers sessile, and seed ovate-circular and areolate.

#### Taxonomic references.

Barneby and Grimes (1996).

### ﻿ Lysiloma

Taxon classificationPlantaeFabalesFabaceae

Benth., London J. Bot. 3: 82. 1844.

Figs 212 , 214

#### Type.

*Lysilomaschiedeanum* Benth. [= *Lysilomadivaricatum* (Jacq.) J.F. Macbr.]

#### Description.

Unarmed small trees or shrubs. **Stipules** commonly submembranous, linear to obliquely dilated, semicordate, or flabellate, absent from all fruiting and most mature flowering specimens. **Leaves** bipinnate, extrafloral nectaries near or well above mid-petiole, sessile, either cupular or mounded, narrow-pored, and smaller nectaries often between some distal pinnae-pairs and toward tips of pinnae; pinnae 1–30 pairs; leaflets 1.5–50 pairs per pinna, venation simple, or weakly pinnate, or palmate-pinnate. **Inflorescence** either spicate-racemose, or spherical capitate, or hemispherical umbelliform. **Flowers** homomorphic or almost so, the central flower sometimes a little stouter, but never longer than the others; calyx campanulate, short-toothed; corolla tubular; stamens 12–32, joined into a tube at the base, exserted; pollen in 16-grained polyads, more or less isodiametric; ovary sessile or almost so, glabrous at anthesis. **Fruit** either a craspedium or an indehiscent legume, long persistent on tree, stipitate, in profile linear or oblong-elliptic, acute or obtuse, framed by prominent sutures, the papery-crustaceous valves plano-compressed except where crumpled or low-bullate over seeds, straight but sometimes twisted through 180° either above or below middle, the exocarp exfoliating to reveal the stramineous endocarp, less often not exfoliating in craspedial species, the valves separating from the persistent sutures and falling together out of the marginal frame, the seeds then released on the ground. **Seeds** transverse, compressed ellipsoid, funicle filiform, lustrous brown, pleurogram commonly U-shaped, sometimes semicircular, or closed.

#### Chromosome number.

2*n* = 26 (Jarolĭmovă 1994).

#### Included species and geographic distribution.

**Figure 214.**
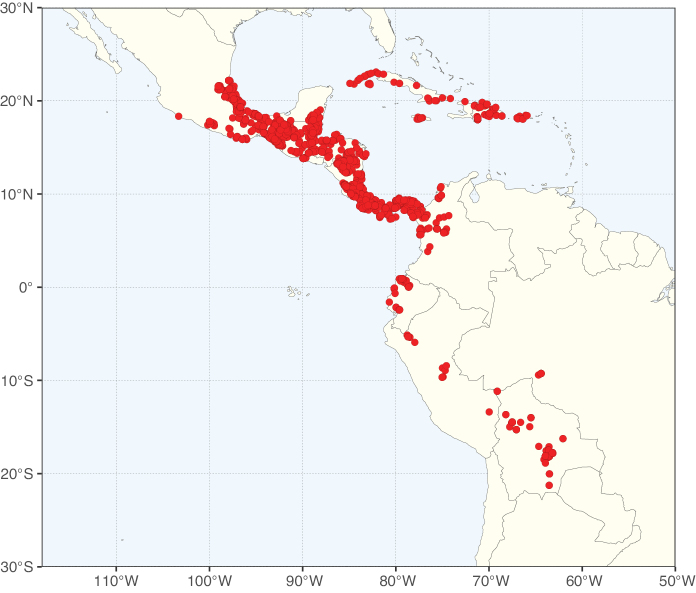
Distribution of *Lysiloma* based on quality-controlled digitised herbarium records. See Suppl. material 1 for the source of occurrence data.

Eight species, in Mexico, Central America as far south as Costa Rica, and the Greater Antilles, one species reaching subtropical south Florida, and one species extending north into south-eastern Arizona in the USA (Fig. 214).

#### Ecology.

Predominantly seasonally dry tropical forests and adjacent arid and semi-arid thorn scrub in north-western Mexico and the southern USA, extending into mid-elevation pine-oak formations and weakly into wetter less seasonal lowland tropical rainforest. Generally on freely-drained soils, coastal sand dunes and shallow limestone soils. Most species are deciduous, flowering often coinciding with leaf flush at the end of the dry season. Fruits are often long persistent on the tree.

#### Etymology.

Derived from the Greek, *Lysis* (= to loose) and *loma* (= edge), referring to the shedding of the legume sutural frame (or edge) at fruit maturity (i.e., craspedial dehiscence).

#### Notes.

The carpological character that distinguishes most *Lysiloma* species from other New World ingoid genera is the craspedial fruit with sutures which usually persist as a frame after the valves have separated (Barneby and Grimes 1997). However, not all species of *Lysiloma* have craspedial dehiscence in which the valves of the pods detach as a unit, together with the seeds, from the often persistent sutural frame. Two species have indehiscent fruits: *L.latisiliquum* (L.) Benth. and *L.sabicu* Benth.

#### Taxonomic references.

Barneby and Grimes (1997); Duno et al. (2021); Gale and Pennington (2004) with illustrations.

### ﻿ Cojoba

Taxon classificationPlantaeFabalesFabaceae

Britton & Rose, N. Amer. Fl. 23: 29. 1928.

Figs 212 , 215

Pithecellobium sect. Cojoba (Britton & Rose) Mohlenbr., Reinwardtia 6: 446. 1963. Type: Pithecellobiumarboreum (L.) Urb. [≡ Mimosaarborea L. (≡ Cojobaarborea (L.) Britton & Rose)]
Obolinga Barneby, Brittonia 41: 170. 1989. Type: Obolingazanonii Barneby [≡ Cojobazanonii (Barneby) Barneby & Grimes]

#### Type.

*Cojobaarborea* (L.) Britton & Rose [≡ *Mimosaarborea* L.]

#### Description.

Unarmed medium-sized to sometimes large canopy-emergent trees to 60 m tall and 1 m stem diameter, and arborescent shrubs, either micro- or macrophyllidious. **Stipules** small, ephemeral, or obsolescent, rarely persistent, sometimes lignescent. **Leaves** bipinnate, in one species [*C.rufescens* (Benth.) Britton & Rose] paripinnate, extrafloral nectaries sessile, cupular thick-rimmed on petiole at, or close, below pinnae-pairs, exceptionally 1–2 on petiole; pinnae 1–22 pairs; leaflets 2–50 pairs per pinna, variable in shape, sometimes rhombic, asymmetric, venation palmate, pinnate, or indistinct. **Inflorescence** spherical capitula, receptacle subglobose, arising either singly or fasciculate. **Flowers** sessile, homomorphic, 5-merous; calyx campanulate or deeply campanulate, 5-veins or almost veinless, very shortly toothed; corolla tubular or very narrowly trumpet-shaped; stamens 20–66, joined at the base, shortly exceeding or as long as the corolla; pollen in 16-grained polyads, isodiametric; intrastaminal disc absent; ovary sessile or subsessile, ellipsoid to linear-ellipsoid. **Fruits** dehiscence through one or both margins, pendulous, linear or broad-linear in profile and simply retrofalcate or randomly to spirally contorted, shallowly to deeply constricted between seeds (moniliform), the sutures immersed; valves leathery-fleshy and glossy red when fresh, after dehiscence withering brown, crumpling and contracting to expose the seeds; endocarp smooth tan internally. **Seeds** ellipsoid to subglobose, funicle straight or simply bent, subfiliform and wiry, or narrowly dilated and stiff after dehiscence, black or dark brown, lacking pleurogram.

#### Chromosome number.

Unknown.

#### Included species and geographic distribution.

**Figure 215.**
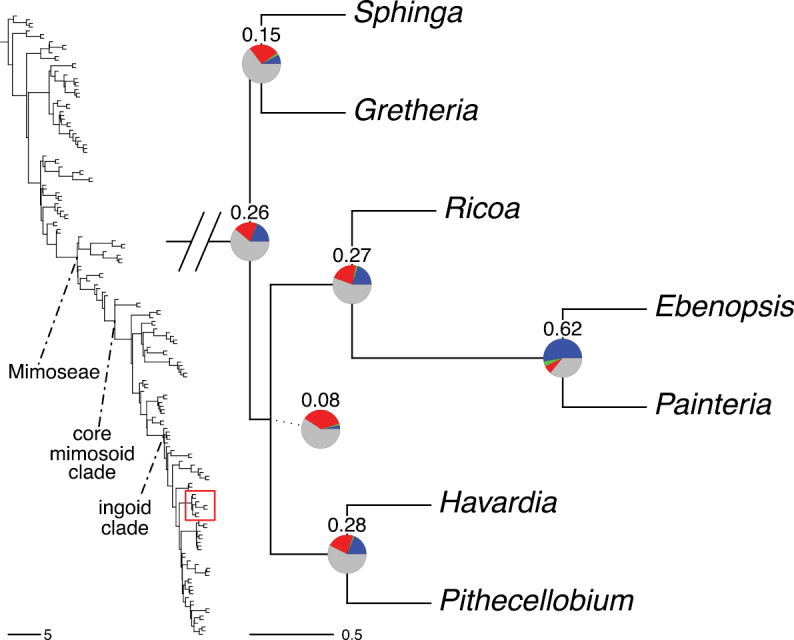
Distribution of *Cojoba* based on quality-controlled digitised herbarium records. See Suppl. material 1 for the source of occurrence data.

Twelve species (Barneby and Grimes 1997), but up to 19 species were recognised by Zamora Villalobos (2010), occurring in southern Mexico, throughout Central America, the Greater Antilles (four species), and north-western trans-Andean South America including the western fringes of Amazonia (four species) (Fig. 215).

#### Ecology.

Most species of *Cojoba* are adapted to wet tropical lowland or submontane to montane evergreen (cloud) forest, and riparian forest, up to ca. 2600 m elevation, but the four West Indian species grow in semi-deciduous lowland scrub, woodland, or chaparral. Flowering apparently at any time of year and in some species more or less continuously so. Seeds likely bird-dispersed.

#### Etymology.

*Cojoba* is a vernacular name for *C.arborea* in Puerto Rico.

#### Notes.

The current concept of the genus *Cojoba* (Barneby and Grimes 1997) is similar to that of Britton and Rose (1928) but expanded to include two additional species: *Cojobafilipes* (Vent.) Barneby & J.W. Grimes and *Obolingazanonii* Barneby (Barneby 1989) [≡*Cojobazanonii* (Barneby) Barneby & J.W. Grimes (1997)]. *Cojoba* is characterised by twigs unarmed, inflorescence usually pendulous, capitate, with homomorphic flowers, fruits pendulous, cylindrical, broadly linear in profile, usually strongly retrofalcate to spirally twisted, the valves bright red and fleshy, barely or deeply constricted between seeds (moniliform), seeds black, shiny, ellipsoid to subglobose, without aril. The fruits of the Antillean species, except for *Cojobaarborea* and *C.filipes*, are massive, cylindrical, straight, or slightly recurved, slightly constricted between seeds, with cylindrical or discoid seeds.

#### Taxonomic references.

Barneby and Grimes (1997); Britton and Rose (1928); Zamora Villalobos (2010).

## ﻿﻿30. Pithecellobium clade

Rodrigo Duno de Stefano^13^

Citation: Duno de Stefano R (2024) 30. Pithecellobium clade. In: Bruneau A, Queiroz LP, Ringelberg JJ (Eds) Advances in Legume Systematics 14. Classification of Caesalpinioideae. Part 2: Higher-level classification. PhytoKeys 240: 391–403. https://doi.org/10.3897/phytokeys.240.101716

**Pithecellobium clade**

Figs 216–224

**Included genera (7).***Ebenopsis* Britton & Rose (3 species), *Gretheria* Duno & Torke (2), *Havardia* Small (3), *Painteria* Britton & Rose (2), *Pithecellobium* Mart. (19), *Ricoa* Duno & Torke (1), *Sphinga* Barneby & J.W. Grimes (3).

**Description.** Small trees, shrubs, sometimes sarmentose, growth sympodial or monopodial; branches armed with stipular spines, rarely absent, proleptic, dimorphic, with vegetative and reproductive brachyblasts; buds protected by adaxial side of petiole. **Stipules** spinescent. **Leaves** bipinnate, coeval or late suppressed; extrafloral nectaries cupular, either sessile or shortly stipitate, below mid-petiole or below proximal pinna-pair, between each pinna pair, at tip of all pinnae; leaflets macrophyllous or microphyllous, opposite, rarely alternate, venation pinnate, reticulate, subpalmate, palmate, or simple. **Inflorescences** spikes or globose capitula that arise singly or in fascicles. **Flowers** uniform; sepals 5, rarely 4 or 6; petals 5, rarely 4 or 6, connate, equal, radially symmetrical; stamens numerous, connate at the base forming a short tube, as long as the corolla or long exerted, anthers elliptic in outline, distinctly wider than long, dehiscence longitudinal; pollen in 16-grained polyads, (more or less) isodiametric; intrastaminal disc present or poorly developed; ovary sessile or shorty stipitate, body compressed-ellipsoid, style about as long as androecium, the stigma poriform. **Fruits** oblong or broad-linear in profile, plano-compressed turgid, recurved or coiled and sometimes also twisted, in some species cylindrically terete; valves stiffly papery, chartaceous, thinly coriaceous, fleshy, leathery, or woody, cavity continuous or septate, dehiscence inert, through both sutures; funicle straight, sinuous, sigmoid or not, spongy arilliform, red, pink, or whitish. **Seeds** lentiform or plumply obese, with pleurogram.

**Distribution.** The Pithecellobium clade is restricted to the New World and strongly centred in Mexico (two genera endemic there and two others nearly so), more sparsely extending to Central America, northern South America (two genera) and the Caribbean (two genera). Mainly below 1000 m elevation but two genera and three species in the highlands of Mexico between 1400–2360 m, predominantly in seasonally dry and arid thickets and forests, less frequent in coastal vegetation and humid forests.

**Figure 216.**
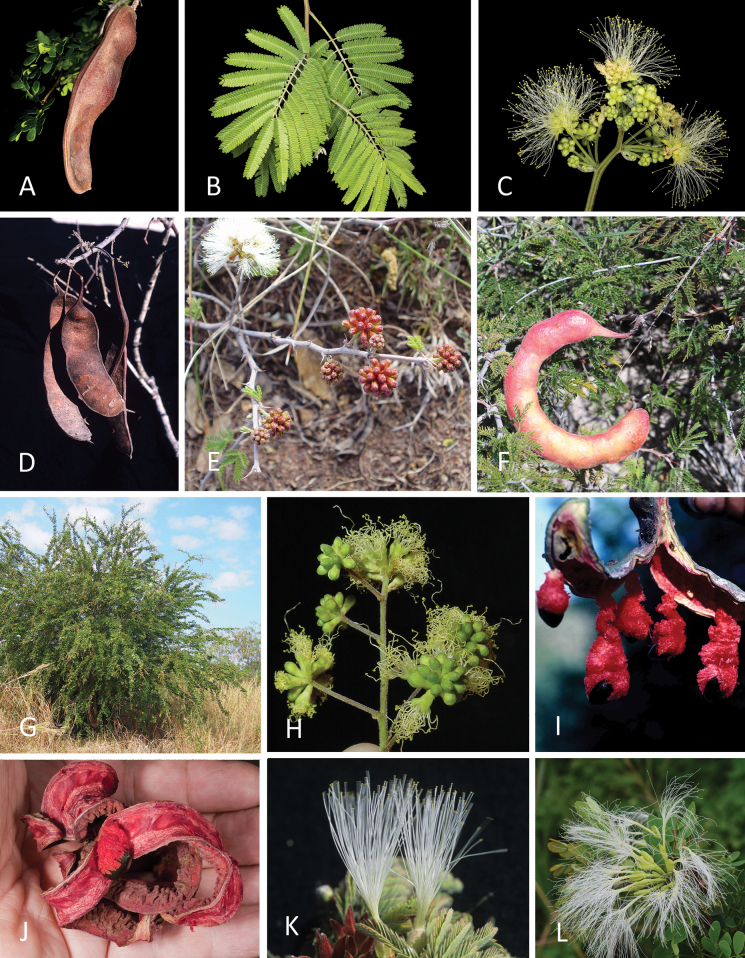
Generic relationships in the Pithecellobium clade (tribe Mimoseae). For description of phylogeny and support values, see Fig. 6 caption (page 63).

**Notes.** The Pithecellobium clade was first recognised as the informal Pithecellobium alliance of tribe Ingeae by Barneby and Grimes (1996) and has been consistently supported as monophyletic in all molecular phylogenetic analyses (e.g., Luckow et al. 2003; Souza et al. 2013; Iganci et al. 2016; Koenen et al. 2020a; Duno de Stefano et al. 2021; Ringelberg et al. 2022).

**Clade based definition.** The most inclusive crown clade containing *Pithecellobiumdulce* (Roxb.) Benth. and *Sphingaplatyloba* (DC.) Barneby & Grimes, but not *Cojobaarborea* (L.) Britton & Rose, *Zapotecacaracasana* (Jacq.) H.M. Hern. or *Cedrelingacateniformis* (Ducke) Ducke (Fig. 216).

The Pithecellobium clade is characterised by stipular spines and brachyblasts that are either strictly vegetative or vegetative and reproductive. Although stipular spines occur in seven ingoid genera worldwide, in the New World the only other ingoid clade taxa known to have stipular spines are *Zapotecaaculeata* (Spruce ex Benth.) H.M. Hern. and *Calliandrapauciflora* (A. Rich.) Griseb. but *Zapoteca* and *Calliandra* differ by having fruits with elastic dehiscence from the apex and in their pollen morphology (e.g., pollen 16-grained vs. 8-grained; Hernández 1986). Some other ingoid clade taxa are armed with spiny peduncles [some species of *Chloroleucon* (Benth.) Britton & Rose], or prickles [*Albiziacorniculata* (Lour.) Druce, *A.myriophylla* Benth., *A.umbellata* (Vahl) E.J.M. Koenen, and *Senegalia* species], but none have stipular spines.

In Bentham’s (1875) treatment of suborder Mimoseae, all species of the Pithecellobium clade then known were included within the genus *Pithecellobium*, four of them placed in his section Ortholobium Benth. Later, Britton and Rose (1928) segregated section Ortholobium into three genera, *Havardia* and two new genera, *Ebenopsis* and *Painteria*. Subsequently, in their generic system of New World tribe Ingeae, Barneby and Grimes (1996, 1997) added the genus *Sphinga*, recognising five genera in their informal Pithecellobium alliance, a system that was followed by Lewis and Rico-Arce (2005). However, recent molecular phylogenetic analyses (Ringelberg et al. 2022; Tamayo-Cen et al. 2022) showed that two of these five genera, *Painteria* and *Havardia* are non-monophyletic, prompting further splitting of these two genera and recognition of two additional new segregate genera, *Gretheria* and *Ricoa* by Tamayo-Cen et al. (2022) even though these entities are only weakly differentiated morphologically by sets of mainly quantitative traits.

### ﻿ Pithecellobium

Taxon classificationPlantaeFabalesFabaceae

Mart., Flora 20 (Beibl. 2): 114. 1837.

Figs 217 , 218

Spiroloba Raf., Sylva Tellur.: 119. 1838. Type not designated.

#### Type.

*Pithecellobiumunguis-cati* (L.) Benth. [≡ *Mimosaunguis-cati* L.]

#### Description.

Shrubs and trees, 1–15 m. **Stipules** spinescent, sometimes absent on some flowering branches, minute or obsolete in *P.keyense* Britton. **Leaves** bipinnate, the rachis occasionally winged, extrafloral nectaries cupular, either sessile or shortly stipitate, between each pair of pinna, and also at tip of all pinnae; pinnae 1–2 (11) pairs; leaflets 4–16 (23) pairs per pinna, variously obovate, elliptic, oblong, obovate and suborbicular, venation pinnate and usually also reticulate. **Inflorescence units** either capitula or relatively short dense spikes, either axillary to the leaves, on efoliate brachyblasts, or paniculately pseudoracemose. **Flowers** sessile, homomorphic, 5-merous; calyx hemispherical, campanulate or sub-cylindrical, shortly toothed; corolla tubular or trumpet-shaped, rarely turbinate, lobes erect or reflexed; stamens 16–76, the tube as long or longer than corolla; intrastaminal disc with small callosities, rarely developed into lobes; pollen in 16-grained polyads, isodiametric; ovary either oblong or ellipsoid, either sessile or long-stipitate. **Fruits** oblong or linear pods, reflexed or coiled and sometimes also twisted, the shallowly undulate or evenly curved sutures broad but not prominent, valves fleshy leathery or woody, red or fuscous, biconvex over seeds, the cavity continuous, incipiently septate or compartmentalised into locules; passively or elastically dehiscent either through both sutures or through the ventral suture only. **Seeds** following dehiscence, dangling on and invested by a red, pink, or whitish, spongy, biconvex arilliform funicle, the testa typically hard, lustrous brown or black, pleurogram present or absent.

#### Chromosome number.

2*n* = 26 (Yeh et al. 1986; Tapia-Pastrana and Gómez-Acevedo 2005).

#### Included species and geographic distribution.

Twenty species, 18 recognised by Barneby and Grimes (1997), and two additional ones by Duno et al. (2013) and García-Lara et al. (2015). USA (Florida Keys and southern California and Arizona), Mexico, Central America and northern South America south as far as Ecuador, Peru, and Brazil, and the Antilles (Bahamas, Cuba, Hispaniola, Jamaica and throughout the lesser Antilles) (Fig. 218). One species (*P.dulce*) cultivated and widely introduced in early Spanish colonial times, now naturalised across the tropics.

#### Ecology.

**Figure 217.**
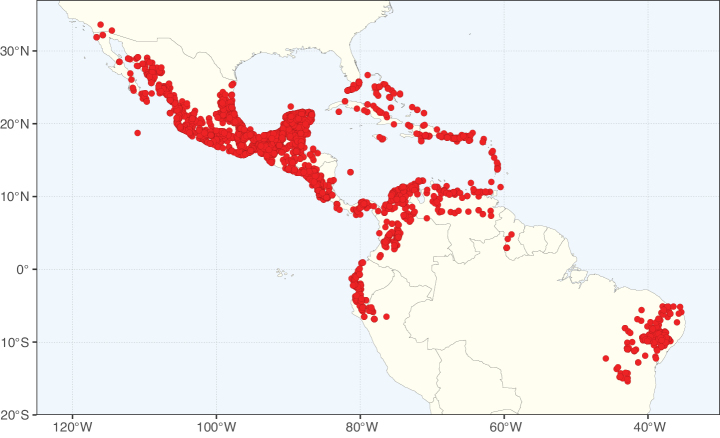
Morphology of selected species of the Pithecellobium clade **A***Ebenopsisebano* (Berland.) Barneby & J.W. Grimes, fruit **B, C***Havardiaalbicans* (Kunth) Britton & Rose **B** leaves **C** inflorescence **D***Gretheriacampylacantha* (L. Rico & M. Sousa) Duno & Torke, fruit **E***Ricoaleptophylla* (DC.) Duno & Torke, inflorescence and young flowers **F***Painteriaelachistophylla* (A. Gray ex S. Watson) Britton & Rose, fruit **G***Pithecellobiumdulce* (Roxb.) Benth. habit **H***Pithecellobiumunguis-cati* (L.) Benth. inflorescence **I***Pithecellobiumlanceolatum* (Humb. & Bonpl. ex Willd.) Benth. pod and seeds **J***Pithecellobiumwinzerlingii* Britton & Rose pod and seed **K***Sphingaacatlensis* (Benth.) Barneby & J.W. Grimes flowers **L***Sphingaplatyloba* (Bertero ex DC.) Barneby & J.W. Grimes flowers. Photo credits **A, E, F, H** R Duno de Stefano **B, C, J** GA Romero González **D, I, K** CE Hughes **G** D Pedersen **L** C Ramírez.

Largely confined to seasonally dry and arid vegetation including coastal dunes, thickets, thorn-scrub, and spiny chaparral, savanna margins, brush-savanna, pine woodland, pine-palmetto savanna, and deciduous or semi-deciduous forests. Several species are adapted to shallow rocky coastal limestone soils sometimes derived from coral reefs, e.g., in Belize and the Bahamas. The conspicuous spongy arils clasping the seeds (Fig. 217I, J) serve as elaiosomes, attractive to birds, ants and humans and thereby contributing to dispersal of the seeds.

**Figure 218.**
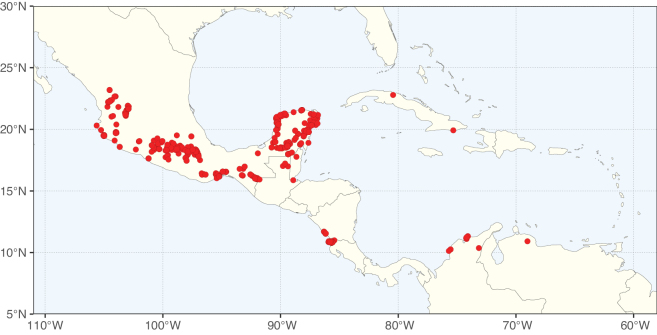
Distribution of *Pithecellobium* based on quality-controlled digitised herbarium records. See Suppl. material 1 for the source of occurrence data.

#### Etymology.

Greek = *pithecos*, monkey + *ellobion*, earring; in allusion to the pendulous, contorted fruit, the spelling and meaning often modified from *Pithecolobium*, as though from *pithecos* + *lobos*, fruit, and later to *Pithecollobium*.

#### Human uses.

*Pithecellobiumdulce* has edible seed-arils (Barneby and Grimes 1996). It is also commonly cultivated as an ornamental, hedge plant, and shade tree in Central and South America, and sporadically across the tropics (e.g, in north-west India) (Barneby and Grimes 1996). Recently, the wood has been used for handicrafts (Duno de Stefano 2008). It is also used for forage and living fences in Mexico (Grether 2007).

#### Notes.

*Pithecellobium* was long considered the main “dustbin” genus of the old sense tribe Ingeae. The genus has gradually changed its circumscription from the late nineteenth century to its current much reduced form, defined for the first time by Britton and Rose (1928), a circumscription followed by Barneby and Grimes (1997), who presented a detailed taxonomic account of the genus recognising 18 species.

*Pithecellobium* can be distinguished from all other genera of former tribe Ingeae by the presence of a seed funicle modified into a usually conspicuous spongy white, pink or crimson aril that cups the lower half of the seed (Fig. 217I, J).

One species, P.×bahamense Northrop is hypothesised by Barneby and Grimes (1997) to be of putative hybrid origin between *P.keyense* Britton and *P.histrix* (A. Richard) Benth.

#### Taxonomic references.

Barneby and Grimes (1997) with illustrations; Bentham (1844, 1875); Britton and Rose (1928); Tamayo-Cen et al. (2022).

### ﻿ Sphinga

Taxon classificationPlantaeFabalesFabaceae

Barneby & J.W. Grimes, Mem. New York Bot. Gard. 74(1): 160. 1996.

Figs 217 , 219

#### Type.

*Sphingaplatyloba* (DC.) Barneby & J.W. Grimes [≡ *Acaciaplatyloba* DC.]

#### Description.

Shrubs and small trees, sometimes sarmentose. **Stipules** subulate, spinescent. **Leaves** bipinnate, extrafloral nectaries below mid-petiole; pinnae 1–4 pairs; leaflets 1–14 pairs per pinna, opposite, obovate or oblong-obovate, venation pinnate or subpalmate. **Inflorescences** capitula, all or almost all arising from brachyblasts. **Flowers** sessile or nearly so, homomorphic, 5-merous, the perianth greatly elongated, flower-buds flask-shaped; calyx cylindrical-campanulate; corolla narrowly trumpet-shaped; stamens 34–176, the tube greatly elongated and far exserted; pollen in 16-celled polyads, more or less isodiametric; intrastaminal disc clasping stipe of ovary; ovary cylindrical. **Fruits** broad-linear, plano-compressed legumes, 6–10-seeded, the stiffly papery valves framed by sutures, the cavity continuous, dehiscent through both sutures. **Seeds** transverse on a dilated, contorted or sigmoid funicle.

#### Chromosome number.

2*n* = 26 (Rico Arce 1992).

#### Included species and geographic distribution.

**Figure 219.**
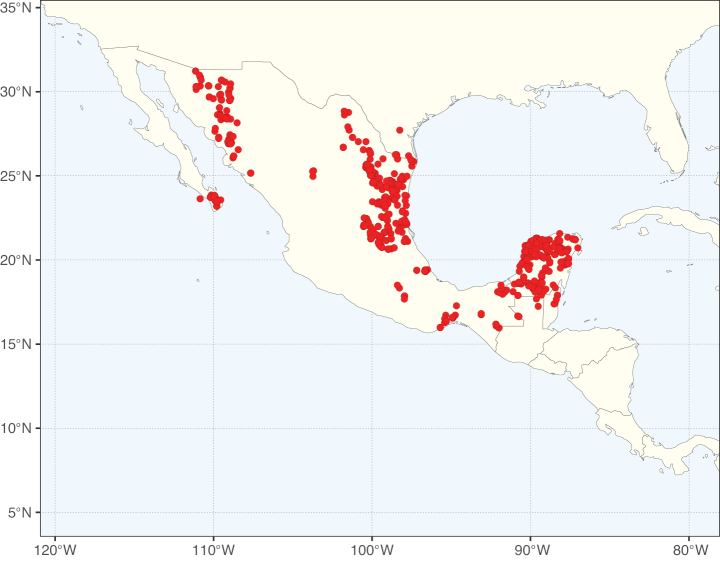
Distribution of *Sphinga* based on quality-controlled digitised herbarium records. See Suppl. material 1 for the source of occurrence data.

Three species, south-central Mexico, sporadically in Central America (Belize, Guatemala and Nicaragua), to northern South America (Colombia, Venezuela), and Aruba, Curaçao (Dutch West Indies), and one species endemic to Cuba (Fig. 219).

#### Ecology.

Arid or seasonally dry tropical forest, mattoral and thorn scrub, to 1600 m elevation in south-central Mexico. Night-flowering and pollinated by Sphingid moths.

#### Etymology.

*Sphinx* (Sphingidae), the putative pollinator + Tupi *inga*, vernacular for several mimosoid legumes (Barneby and Grimes 1996).

#### Human uses.

Unknown.

#### Notes.

The three species of *Sphinga* were all known to Bentham (1875) and grouped into an informal division of his Pithecolobiumsect.Ortholobium. Britton and Rose (1928) later transferred them to *Havardia*. Barneby and Grimes (1996) proposed the new genus *Sphinga* for these three species, which differ from *Havardia* in the greatly elongated perianth with a long, silky corolla opening at nightfall.

#### Taxonomic references.

Barneby and Grimes (1996); Britton and Rose (1928); Tamayo-Cen et al. (2022).

### ﻿ Havardia

Taxon classificationPlantaeFabalesFabaceae

Small, Bull. New York Bot. Gard. 2: 91. 1901.

Figs 217 , 220

Pithecolobium sect. Ortholobium Benth., Trans. Linn. Soc. London 30: 592. 1875. Type: Pithecellobiumalbicans (Kunth) Benth. [≡ Acaciaalbicans Kunth (≡ Havardiaalbicans (Kunth) Britton & Rose)]

#### Type.

*Havardiabrevifolia* (Benth.) Small [≡ *Pithecellobiumbrevifolium* Benth. (= *Havardiapallens* (Benth.) Britton & Rose)]

#### Description.

Trees or bushy treelets 2–9(–12) m. **Stipules** spinescent. **Leaves** bipinnate, extrafloral nectaries sessile, shallowly cupular, thick-rimmed, inserted near or below mid-petiole, rarely 2, rarely at very base, sometimes a little above mid-petiole, sometimes rudimentary; pinnae 1–11 (13) pairs; leaflets 7–36 pairs per pinna, alternate, rarely opposite, linear, linear-oblong, to narrowly oblong-obovate, venation simple or pinnate. **Inflorescence** 6–37-flowered capitula or capituliform racemes. **Flowers** sessile or very shortly pedicellate, homomorphic, 5-merous; calyx hemispherical, shallowly campanulate to deeply campanulate, teeth deltoid; corolla campanulate, narrowly vase-shaped to subcylindrical, lobes recurving to erect; stamens (28) 30–52; pollen in 16-celled polyads, more or less isodiametric; intrastaminal disc 5-lobed or simple callosities; ovary subsessile or shortly stipitate, slenderly ellipsoid, style about as long as stamens, the stigma poriform. **Fruits** oblong to broad-linear, straight, plano-compressed legumes, 8–13-seeded, valves framed by a bluntly 3-angulate suture, valves chartaceous or thinly coriaceous, stiffly papery, low-convex over each seed on one face or on both, externally veinless, the cavity continuous; dehiscence tardy, inert, through both sutures. **Seeds** transverse, orbicular or oblong-elliptic, compressed-disciform and flattened around the periphery, funicle distally sigmoid, pleurogram U-shaped to closed.

#### Chromosome number.

Unknown.

#### Included species and geographic distribution.

**Figure 220.**
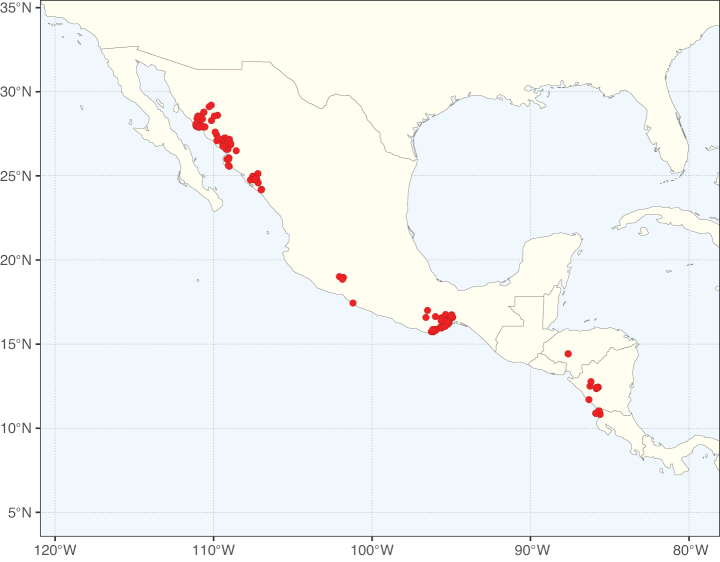
Distribution of *Havardia* based on quality-controlled digitised herbarium records. See Suppl. material 1 for the source of occurrence data.

Three species, almost entirely distributed in Mexico mainly in the north and the Yucatan peninsula, extending marginally north into the USA (south Texas), and south into northern Guatemala and Belize (Fig. 220).

#### Ecology.

Largely confined to seasonally dry, drought deciduous woodland and arid thorn scrub and arid mattoral in the Sonoran desert, Tamaulipan plains and coastal thickets on sand, secondary forest, and extending weakly into lowland rainforest, occasionally on limestone. In parts of Mexico, abundant. For example, *H.albicans* (Kunth) Britton & Rose is very common in the peninsula of Yucatán, and *H.pallens* is abundant in parts of northern Mexico.

#### Etymology.

Named after Dr. Valery Havard (1846–1927), U.S. Army, a diligent student of the North American flora.

#### Human uses.

The boiled wood of *H.albicans* is used in the chemical industry to colour cement (e.g., for swimming pools; Duno 2014) and it has been used as an addition to the fermented psychoactive drink Pulque. In addition, the stem of *H.pallens* is used in the construction of fences and the wood is used for the manufacture of furniture.

#### Notes.

Britton and Rose (1928) provided the first taxonomic revision of *Havardia* including three other species which are now ascribed to *Sphinga*. The fruits of the two genera are similar. Barneby and Grimes (1996) updated this taxonomic treatment recognising five species, but *Havardia*, as circumscribed by Barneby and Grimes (1996), is not supported as monophyletic in the analyses of Koenen et al. (2020a), Ringelberg et al. (2022) or Tamayo-Cen et al. (2022), prompting assignment of two species to the new segregate genus *Gretheria* (Tamayo-Cen et al. 2022), based on floral characters.

#### Taxonomic references.

Barneby and Grimes (1996); Britton and Rose (1928); Calderón de Rzedowski (2007); Small (1991); Tamayo-Cen et al. (2022).

### ﻿ Gretheria

Taxon classificationPlantaeFabalesFabaceae

Duno & Torke, PhytoKeys 205: 292. 2022.

Figs 217 , 221

#### Type.

*Gretheriasonorae* (S. Watson) Duno & Torke [≡ *Pithecellobiumsonorae* S. Watson]

#### Description.

Arborescent shrubs and small, often multi-stemmed trees, 2–14 m. **Stipules** stout, recurved, early lignescent, spinescent, these persistent on shoots and trunks long after leaves are shed. **Leaves** bipinnate, extrafloral nectaries at or below the midpoint of the petiole, sessile, shallow-cupular, thick-rimmed or plane and dimpled; pinnae 1–6 (13) pairs; leaflets 10–31 pairs per pinna, opposite, venation pinnate, the midrib slightly displaced, giving rise on each side to 2–5 weak secondary veins. **Inflorescences** capituliform racemes arising from brachyblasts. **Flowers** sessile, homomorphic, 5-merous; calyx deeply campanulate; corolla subcylindrical, lobes erect, white-silky strigose dorsally; stamens 40–52; pollen in 16-celled polyads, more or less isodiametric; intrastaminal disc 5-lobed or simple callosities; ovary ellipsoid, shortly stipitate. **Fruits** oblong, contracted, body straight or almost straight, plano-compressed legumes, the valves bluntly framed by longitudinally 3-ridged sutures, stiff, somewhat brittle, brownish-green, externally veinless, glabrous, red-granular or both granular and puberulent outside, the cavity continuous. **Seeds** transverse, compressed, disciform to orbicular in outline, funicle dilated, sigmoid, pleurogram U-shaped.

#### Chromosome number.

Unknown.

#### Included species and geographic distribution.

**Figure 221.**
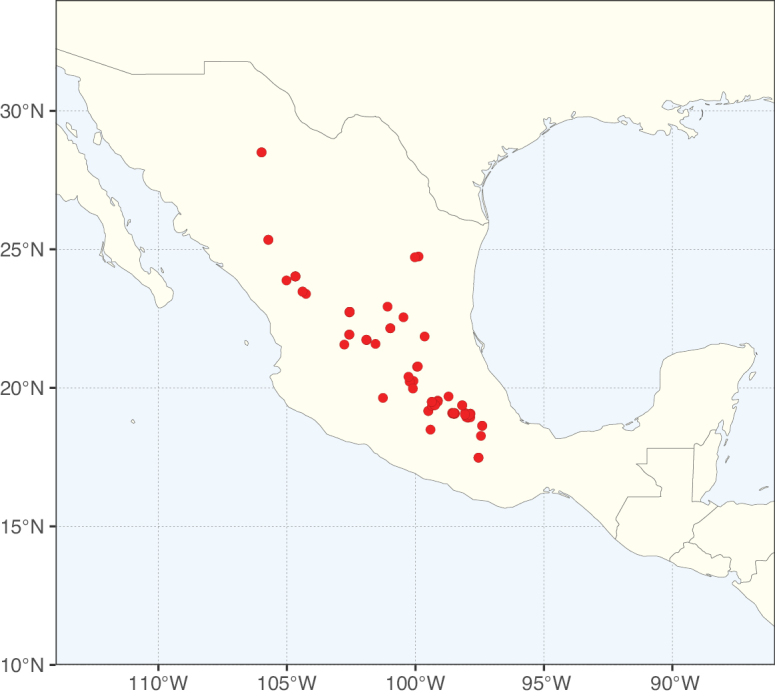
Distribution of *Gretheria* based on quality-controlled digitised herbarium records. See Suppl. material 1 for the source of occurrence data.

Two species, disjunctly scattered along the Pacific coast of Mexico, from Sonora south to Central America (Honduras, Nicaragua, and northwestern Costa Rica) (Fig. 221).

#### Ecology.

Tropical dry forests, thorn scrub and brush-woodlands, from sea level to 400 m elevation, occasionally to 700 m.

#### Etymology.

The generic name honours Rosaura Grether González, a Mexican botanist.

#### Notes.

*Gretheria* was segregated from *Havardia* by Tamayo-Cen et al. (2022) to account for the non-monophyly of *Havardia*, and is differentiated from *Havardia* by flowers with a longer calyx with shorter lobes, the corolla deeply campanulate with erect lobes, and a five-lobed nectary disc or callosities surrounding the ovary (in *Havardia* absent or thickened callosities).

#### Taxonomic references.

Barneby and Grimes (1996); Britton and Rose (1928); Calderón de Rzedowski (2007); Tamayo-Cen et al. (2022).

### ﻿ Ricoa

Taxon classificationPlantaeFabalesFabaceae

Duno & Torke, PhytoKeys 205: 294. 2022.

Figs 217 , 222

#### Type.

*Ricoaleptophylla* (DC.) Duno & Torke [≡ *Acacialeptophylla* DC.]

#### Description.

Xerophytic shrubs 0.2–1.5 m, often growing in patches several meters in diameter. **Stipules** straight to recurved spines, persistent. **Leaves** bipinnate, extrafloral nectaries subsessile or shortly stipitate, circular, between the first pinnae pair (sometimes also between the second pair), absent on the pinnae; pinnae 3–7 (9) pairs; leaflets 8–25 pairs, opposite, venation weakly developed, nearly simple or 1-branched, subcentric. **Inflorescences** capitula, arising from brachyblasts. **Flowers** sessile, mostly homomorphic but some functionally staminate, 5-merous; calyx campanulate, teeth ovate or deltate; corolla tubular, lobes ovate, recurving; stamens 40–76; intrastaminal callosities developed, sometimes obscure or wanting in staminate flowers; polyads 16-celled, more or less isodiametric; ovary slenderly ellipsoid, compressed, shortly stipitate. **Fruits** falcately or subcircinnately broadly linear fruits, the valves stiffly leathery, at first plano-compressed, becoming turgid and low-convex (on both faces of legume) over each seed, indistinctly venulose, the cavity continuous, dehiscence inert through both sutures. **Seeds** obliquely descending, compressed-lentiform, funicle straight or sinuous (but not sigmoid), testa smooth, hard, moderately lustrous, dark castaneous, the pleurogram incomplete.

#### Chromosome number.

Unknown.

#### Included species and geographic distribution.

**Figure 222.**
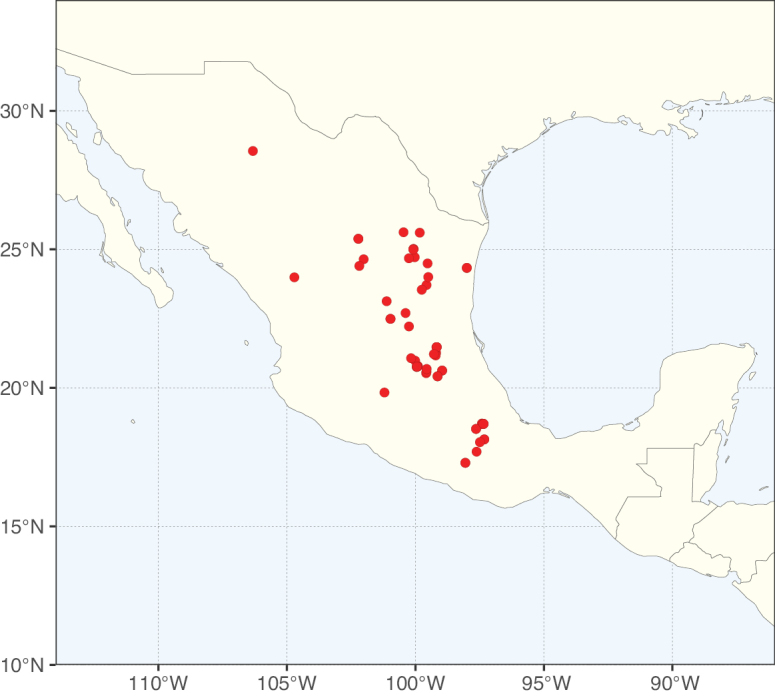
Distribution of *Ricoa* based on quality-controlled digitised herbarium records. See Suppl. material 1 for the source of occurrence data.

Monospecific (*R.leptophylla*), endemic to Mexico, widely scattered over the Central Mexican Plateau (Fig. 222).

#### Ecology.

*Ricoaleptophylla* grows in dry or semi-arid grasslands and thornscrub, extending into the lower edge of the pine-oak belt, on both basaltic and calcareous substrates, at 1600–2800 m.

#### Etymology.

The generic name honours María Lourdes Rico, whose profound dedication and unrelenting commitment to botanical research over decades has deeply enhanced knowledge and understanding of the Leguminosae, especially of the ingoid clade.

#### Notes.

*Ricoa* was segregated from *Painteria* to account for the non-monophyly of that genus (Tamayo-Cen et al. 2022). The genus is morphologically similar to *Painteria* and geographically sympatric with that genus, and is distinguished by a set of quantitative leaf and flower traits.

The common name for *R.leptophylla* is Huisache, a name that is also applied to *Vachelliafarnesiana* (L.) Wight & Arn. and other related species with spinescent stipules (Barneby and Grimes 1996). Other common names are charrasquillo, gatuña, and tehuixtle (Calderón de Rzedowski 2007).

#### Taxonomic references.

Tamayo-Cen et al. (2022).

### ﻿ Painteria

Taxon classificationPlantaeFabalesFabaceae

Britton & Rose, N. Amer. Fl. 23: 35. 1928.

Figs 217 , 223

#### Type.

*Painteriarevoluta* (Rose) Britton & Rose [≡ *Pithecellobiumrevolutum* Rose]

#### Description.

Shrubs 0.3–15 cm. **Stipules** of long-shoots lignescent, of short-shoots acicular or subulate, rather closely imbricate. **Leaves** bipinnate, extrafloral nectaries between proximal pinna-pair, rarely between 2 pairs; pinnae 1–7 pairs; leaflets 3–20 pairs per pinna, suborbicular, elliptic, and broadly oblong, completely revolute in *P.revoluta*, venation palmate or almost simple. **Inflorescences** shortly spiciform capitula arising from brachyblasts. **Flowers** sessile or almost so, homomorphic, 5-merous; calyx campanulate or hemispherical, lobes deltoid; corolla sub-cylindrical, the lobes either ascending or recurving; stamens 28–76, the tube either included or shortly exserted; pollen in 16-celled polyads, more or less isodiametric; intrastaminal small callosities, sometimes obscure or wanting; ovary slenderly ellipsoid, compressed, on a short stipe. **Fruits** compressed legumes, retrofalcate or retrocircinnate, the leathery valves biconvex over each seed, the cavity continuous; dehiscence tardy through both sutures; funicle straight or sinuous, not sigmoid. **Seeds** compressed-lentiform.

#### Chromosome number.

Unknown.

#### Included species and geographic distribution.

**Figure 223.**
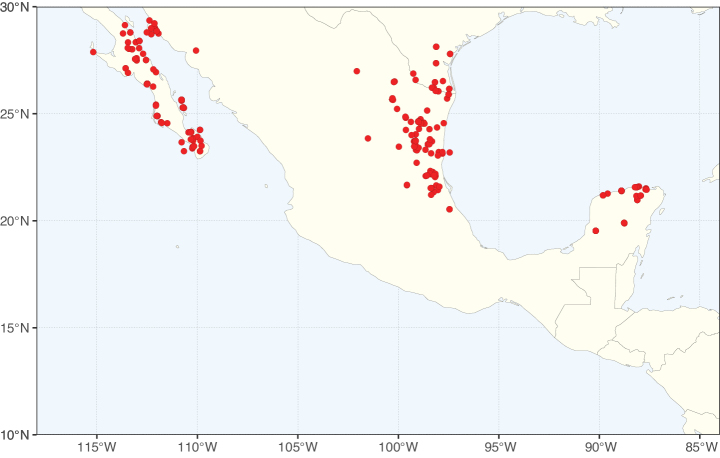
Distribution of *Painteria* based on quality-controlled digitised herbarium records. See Suppl. material 1 for the source of occurrence data.

Two species, endemic to the central Mexican Plateau and adjacent arid interior valleys of Puebla and Oaxaca (Fig. 223).

#### Ecology.

Plains and hillsides in desert grassland and brush communities, mainly between 1400–2750 m.

#### Etymology.

The genus is named after Joseph Hannum Painter (1879–1908), botanist and assistant curator in the division of plants of the United States National Museum, who collected the type species in Querétaro.

#### Human uses.

Unknown.

#### Notes.

Britton and Rose (1928) provided the first taxonomic revision of the genus, which was updated by Barneby and Grimes (1996) who recognised three species, one of which was later segregated as the genus *Ricoa* by Tamayo-Cen et al. (2022). The flowers of *P.revoluta* were first described by Calderón de Rzedowski (2007).

#### Taxonomic references.

Barneby and Grimes (1996); Britton and Rose (1928); Calderón de Rzedowski (2007); Tamayo-Cen et al. (2022).

### ﻿ Ebenopsis

Taxon classificationPlantaeFabalesFabaceae

Britton & Rose, N. Amer. Fl. 23: 33. 1928.

Figs 217 , 224

Hoopesia Buckley, Proc. Acad. Nat. Sci. Philadelphia 13: 453. 1862. Type: Hoopesiaarborea Buckley [= Ebenopsisebano (Berland.) Barneby & J.W. Grimes]
Siderocarpos Small, Bull. New York Bot. Gard. 2: 91. 1901, nom. illeg., non Siderocarpus Pierre, Not. Bot. Sapot.: 31. 1890 (Sapotaceae). Type: Siderocarposflexicaulis (Benth.) Small [≡ Acaciaflexicaulis Benth. (= Ebenopsisebano (Berland.) Barneby & J.W. Grimes)]

#### Type.

*Ebenopsisebano* (Berland.) Barneby & Grimes [≡ *Mimosaebano* Berland.]

#### Description.

Trees and shrubs, 2–8 (20) m. **Stipules** early lignescent, persistent. **Leaves** bipinnate, extrafloral nectaries interpinnal, cupular, short-stipitate; pinnae 1–4 pairs; leaflets 2–7 pairs per pinna, opposite, oblong, oblong-obovate rhombic-oblong, obovate-elliptic to suborbicular, venation palmate. **Inflorescences** spikes or capitula arising from brachyblasts. **Flowers** sessile, homomorphic, 5-merous; calyx campanulate; corolla tubular, lobes erect; stamens 32–66, the tube included or barely exserted from the corolla; pollen in 16-celled polyads, more or less isodiametric; intrastaminal disc lobed or small callosities. **Fruits** massive, compressed sausage-like, erect or slightly curved legumes, the woody valves produced inwardly as pithy interseminal septa, the exocarp in age breaking into polygons; dehiscence tardy, inert, the sutures at first separating at each end of pod but not gaping, ultimately separating through their whole length. **Seeds** transverse on straight, subterete funicle, irregularly globose, resembling chickpeas, red-castaneous in colour.

#### Chromosome number.

Unknown.

#### Included species and geographic distribution.

**Figure 224.**
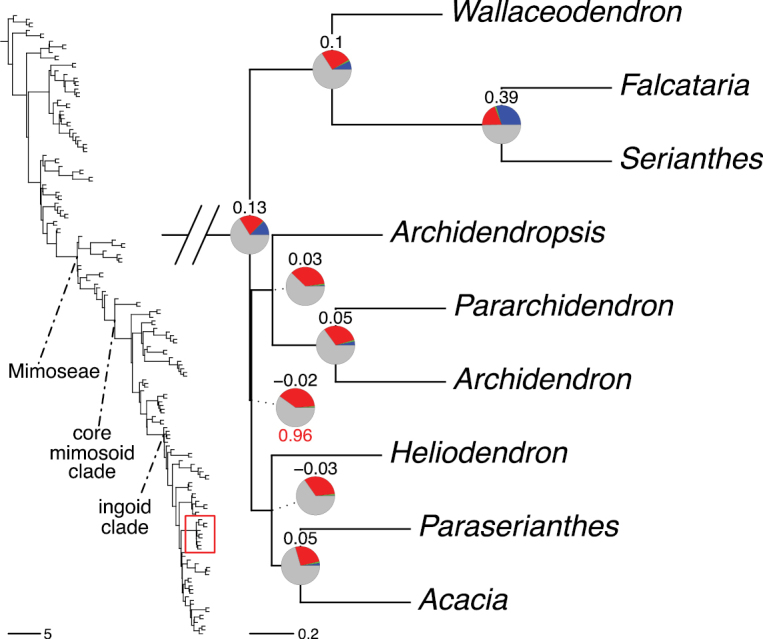
Distribution of *Ebenopsis* based on quality-controlled digitised herbarium records. See Suppl. material 1 for the source of occurrence data.

Three species. Markedly disjunct across Mexico in Baja California, Sonora-Sinaloa, north-eastern Mexico, and the northern half of the Yucatán Peninsula, the genus almost endemic to Mexico, but extending into south Texas (Fig. 224).

#### Ecology.

In tropical and subtropical arid thickets and dry forests and adjacent desert hillsides and desertic fringes in Baja California and Sonora, mostly below 500 m, cultivated in Florida. Often locally abundant as a shrubby treelet forming thickets in thorn scrub and chaparral on impermeable caliche substrates in parts of Tamaulipas and Baja California. The fruits ripen and open slowly and are sometimes described as indehiscent, but after falling, often entire, the valves are eventually fully dehiscent shedding their seeds passively on the ground.

#### Etymology.

From the words *ebano* (*Diospyroscrassiflora* Hiern, Ebeneaceae) and *ópsis* (“aspect”, “appearance”). The wood resembling African ebony in appearance.

#### Human uses.

In Mexico, the hard wood is used for fence posts and provides high quality charcoal, perhaps accounting for the scarcity of larger trees.

#### Notes.

Britton and Rose (1928) provided the first taxonomic revision of the genus, which was subsequently updated by Barneby and Grimes (1996). The fruit and seeds of *Ebenopsis* are striking and easily distinguish the genus from all other members of the Pithecellobium clade being generally straight and lignescent, septate internally, with thickened woody valves which are black and finely fissured in a polygon pattern, with plump irregularly subglobose, red-castaneous seeds.

#### Taxonomic references.

Barneby and Grimes (1996) with illustration; Britton and Rose (1928); Tamayo-Cen et al. (2022).

## ﻿﻿31. Archidendron clade

Else Demeulenaere^12^, Stefanie M. Ickert-Bond^24^, Daniel J. Murphy^35^, Bruce R. Maslin^31,32^, Gillian K. Brown^7^

Citation: Demeulenaere E, Ickert-Bond SM, Murphy DJ, Maslin BR, Brown GK (2024) 31. Archidendron clade. In: Bruneau A, Queiroz LP, Ringelberg JJ (Eds) Advances in Legume Systematics 14. Classification of Caesalpinioideae. Part 2: Higher-level classification. PhytoKeys 240: 404–429. https://doi.org/10.3897/phytokeys.240.101716

**Archidendron clade**

Figs 225–240

**Included genera (9).***Acacia* Mill. (1082 species), *Archidendron* F. Muell. (ca. 120), *Archidendropsis* I.C. Nielsen (11), *Falcataria* (I.C. Nielsen) Barneby & J.W. Grimes (3), *Heliodendron* Gill.K. Br. & Bayly (3), *Pararchidendron* I.C. Nielsen (1), *Paraserianthes* I.C. Nielsen (1), *Serianthes* Benth. (18), *Wallaceodendron* Koord. (1).

**Description.** Trees or shrubs. **Stipules** mostly present, caducous, variable in shape, rarely spinescent (some *Acacia* and *Heliodendron*) or absent (some *Archidendron*). **Leaves** mostly bipinnate, rarely unifoliolate (some *Archidendron*), reduced to scales or polymorphic phyllodes or absent (many *Acacia*); extrafloral nectaries present or absent, variable in shape; leaflets (in bipinnate leaves) opposite or alternate. **Inflorescences** either simple or compound racemes, panicles, spikes, capitula or umbels. **Flowers** uniform, bisexual, rarely unisexual (some *Acacia* and *Archidendron*), 5- or 4-merous (some *Archidendron*, *Archidendropsis*); calyx gamosepalous, less frequently free or absent (some *Acacia*), valvate; corolla gamopetalous, valvate; stamens numerous, united into a tube at the base, or free (most species of *Acacia*), staminal tube and corolla tube shortly untied at the base; pollen in 4, 8, 12, 16 or 32-celled polyads; ovary usually 1, less frequently 2 to several in some genera, sessile or stipitate. **Fruit** dehiscent through one or both sutures or indehiscent (*Serianthes*, rarely *Acacia*), endocarp indistinct or bright coloured or sometimes separating from the exocarp and breaking into 1-seeded envelopes (*Wallaceodendron*). **Seeds** flattened or swollen, unwinged or narrowly winged, seed coat hard or thin, funiculate or not, pleurogram present, U-shaped (*Falcataria*, *Pararchidendron*, *Serianthes*, *Wallaceodendron*, some *Acacia*), closed (some *Acacia*) or absent (*Archidendron*, *Archidendropsis*, *Heliodendron*).

**Distribution.** The members of the Archidendron clade are largely restricted to the Indomalayan and Australasian regions, from southern India and Sri Lanka in the west, to New Caledonia in the east; and from Taiwan and the Ryukyu Islands in the north, to Australia in the south.

**Figure 225.**
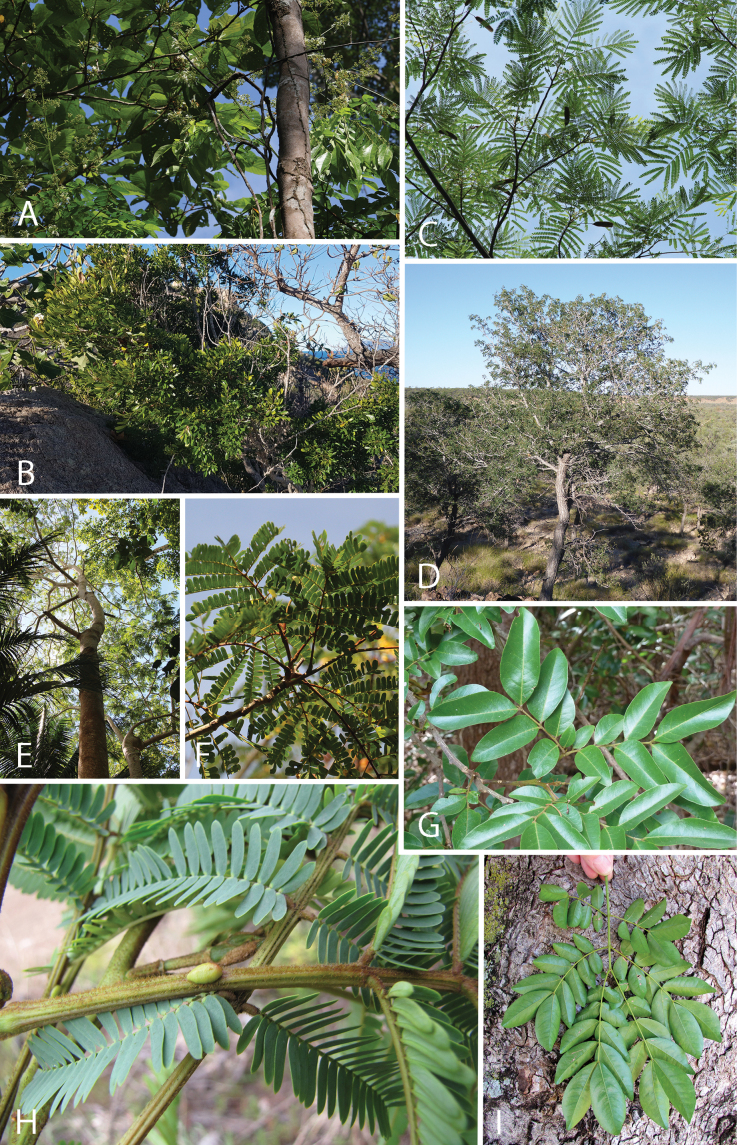
Generic relationships in the Archidendron clade (tribe Mimoseae). For description of phylogeny and support values, see Fig. 6 caption (page 63).

**Clade-based definition.** The most inclusive crown clade including *Archidendronlucidum* (Benth.) I.C. Nielsen and *Wallaceodendroncelebicum* Koord., but not *Pithecellobiumdulce* (Roxb.) Benth., *Cedrelingacateniformis* (Ducke) Ducke or *Punjubaracemiflora* (Donn. Sm.) Britton & Rose (Fig. 225).

**Figure 226.**
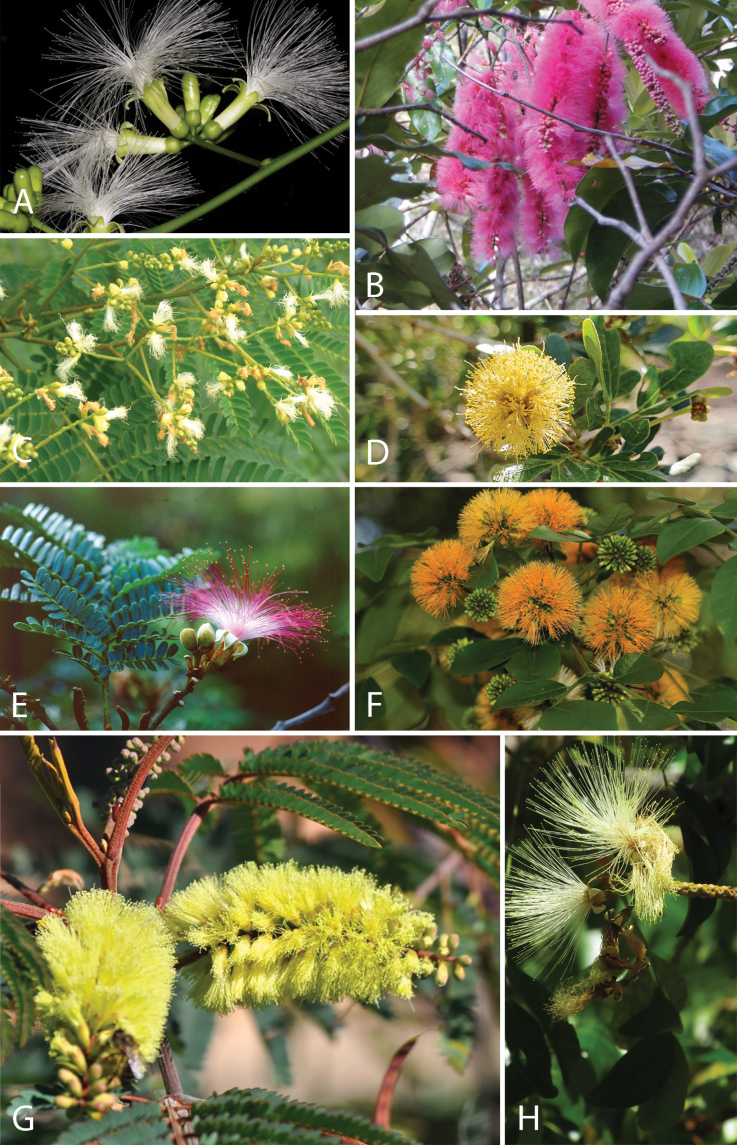
Habit and leaf morphology diversity in genera of the Archidendron clade **A***Archidendronclypearia* (Jack) I.C. Nielsen **B***Heliodendronthozetianum* (F. Muell.) Gill.K. Br. & Bayly **C***Falcatariafalcata* (L.) Greuter & R. Rankin **D***Heliodendronbasalticum* (F. Muell.) Gill.K. Br. & Bayly **E**SeriantheskanehiraeFosbergvar.kanehirae**F**Seriantheskanehiraevar.yapensis Fosberg **G***Pararchidendronpruinosum* (Benth.) I.C. Nielsen **H**Paraseriantheslophanthasubsp.montana (Jungh.) Benth. with tomentose stems and leaves **I***Wallaceodendroncelebicum* Koord. Photo credits **A** C Ng **B** R Cumming **C** J Teo **D** D Richter **E, F** JB Friday **G** G Brown **H** F and K Starr **I** Plantoholic Sheila.

**Figure 227.**
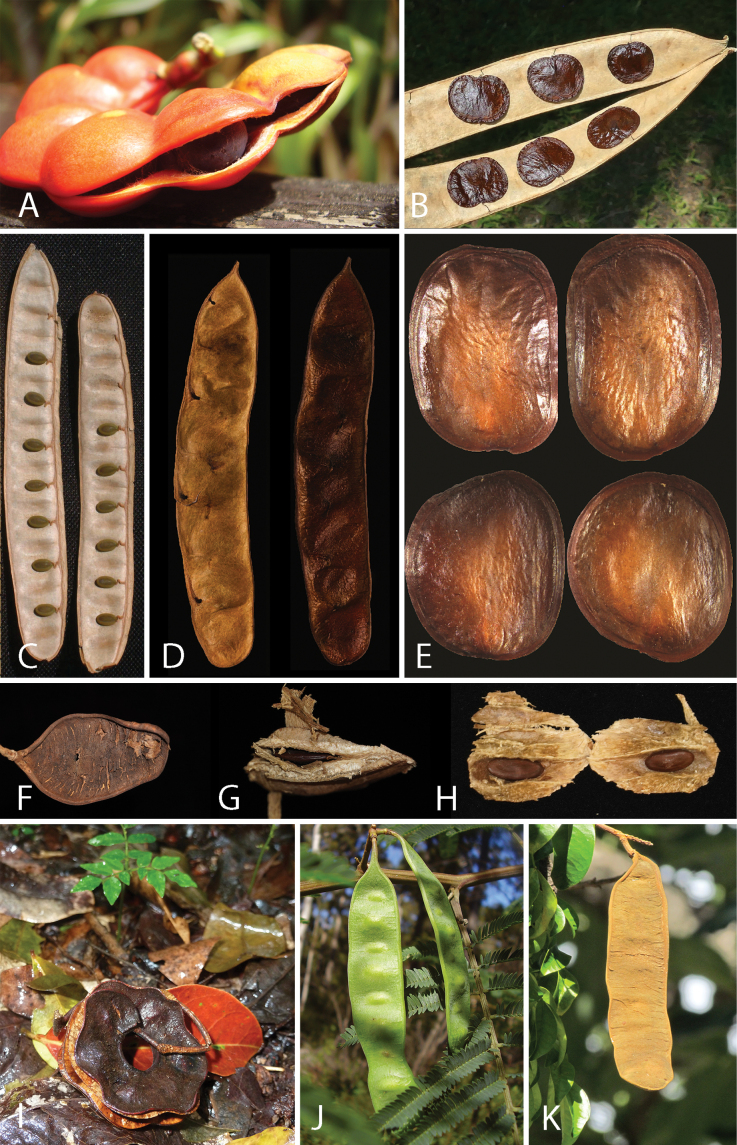
Inflorescence and flower diversity of genera in the Archidendron clade **A***Archidendronvaillantii* (F. Muell.) F.Muell. **B***Archidendropsispaivana* (E. Fourn.) I.C. Nielsen **C***Falcatariafalcata* (L.) Greuter & R. Rankin **D***Heliodendronthozetianum* (F. Muell.) Gill.K. Br. & Bayly **E***Serianthes* sp. **F***Pararchidendronpruinosum* (Benth.) I.C. Nielsen **G***Paraseriantheslophantha* (Willd.) I.C. Nielsen **H***Wallaceodendroncelebicum* Koord. Photo credits **A, F** A Furhmann **B** B Henry **C** JB Friday **D** S Worboys **E** T Rodd **G** AndyBonsai **H** Plantaholic Sheila.

**Figure 228.**
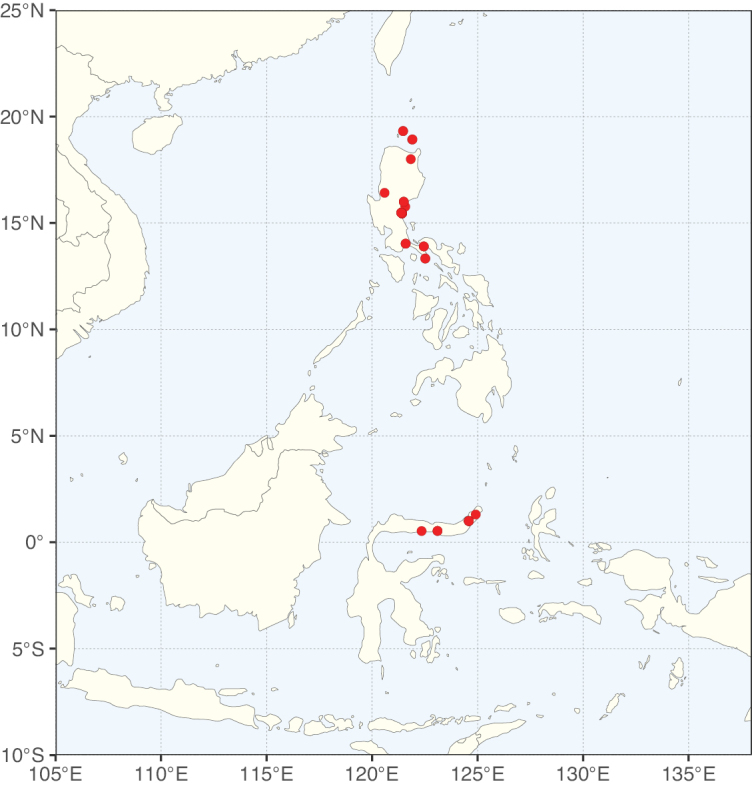
Fruit and seed diversity of genera in the Archidendron clade **A** fruit of *Archidendronlucyi* F. Muell. (*Brown 169*) **B** open pod of *Archidendropsisstreptocarpa* (E. Fourn.) I.C. Nielsen showing winged seeds **C** opened pod of *Falcatariafalcata* (L.) Greuter & R. Rankin **D***Heliodendronxanthoxylon* (C.T. White & W.D. Francis) Gill.K. Br. & Bayly opened pod (*Hyland 9229*) **E***Heliodendronbasalticum* (F. Muell.) Gill.K. Br. & Bayly winged seeds (*Canning & B. Rimes 6173*) **F–H***Serianthesdilmyi* Fosberg showing the indehiscent (to very tardily dehiscent) (rarely) woody pods with transverse seeds which are each isolated in a chamber **I***Pararchidendronpruinosum* (Benth.) I.C. Nielsen fruit and seedling **J***Paraseriantheslophantha* (Willd.) I.C. Nielsen immature pods (*Brown 204A*) **K***Wallaceodendroncelebicum* Koord. pod. Photo credits **A, I, J** G Brown **B** B Henry **C** JB Friday **D, E** Queensland Herbarium **F–H** R CJ Lim **K** Plantaholic Sheila.

**Figure 229.**
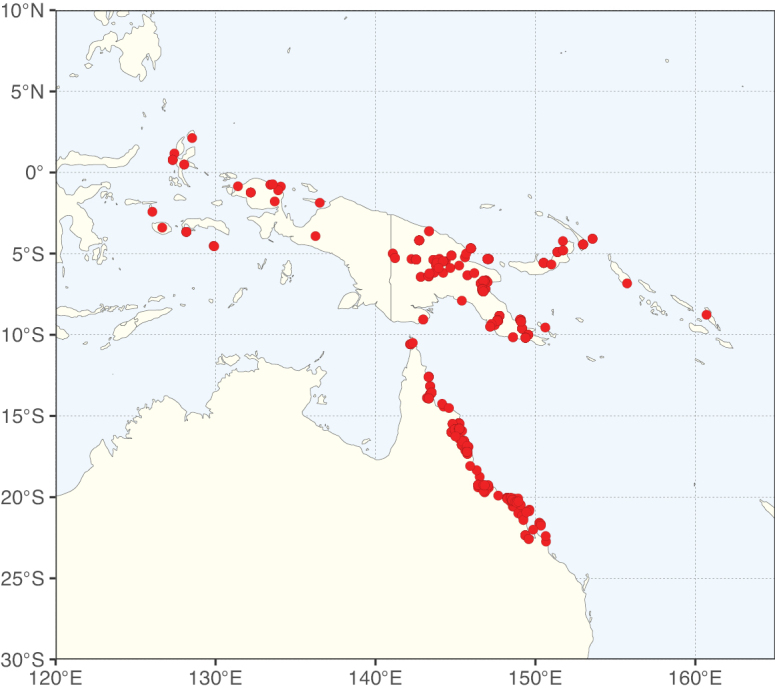
Distribution of *Wallaceodendron* based on quality-controlled digitised herbarium records. See Suppl. material 1 for the source of occurrence data.

**Figure 230.**
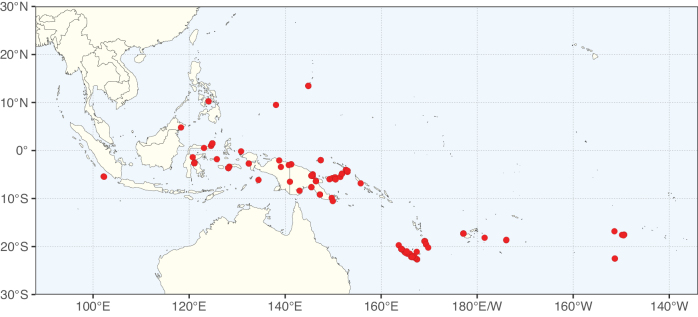
Distribution of *Falcataria* based on quality-controlled digitised herbarium records. See Suppl. material 1 for the source of occurrence data.

**Figure 231.**
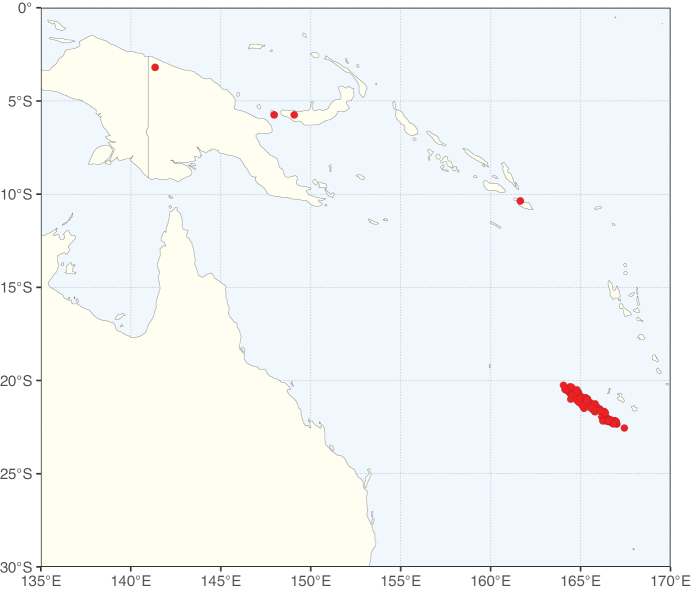
Distribution of *Serianthes* based on quality-controlled digitised herbarium records. See Suppl. material 1 for the source of occurrence data.

**Figure 232.**
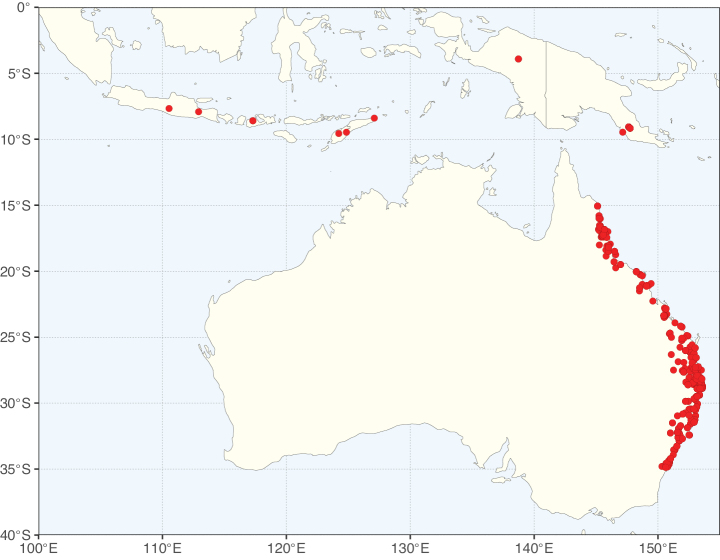
Distribution of *Archidendropsis* based on quality-controlled digitised herbarium records. See Suppl. material 1 for the source of occurrence data.

**Figure 233.**
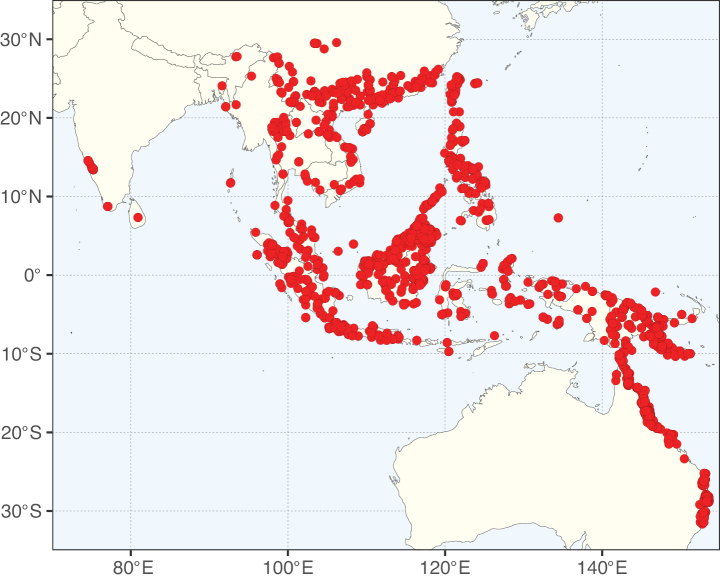
Distribution of *Pararchidendron* based on quality-controlled digitised herbarium records. See Suppl. material 1 for the source of occurrence data.

**Figure 234.**
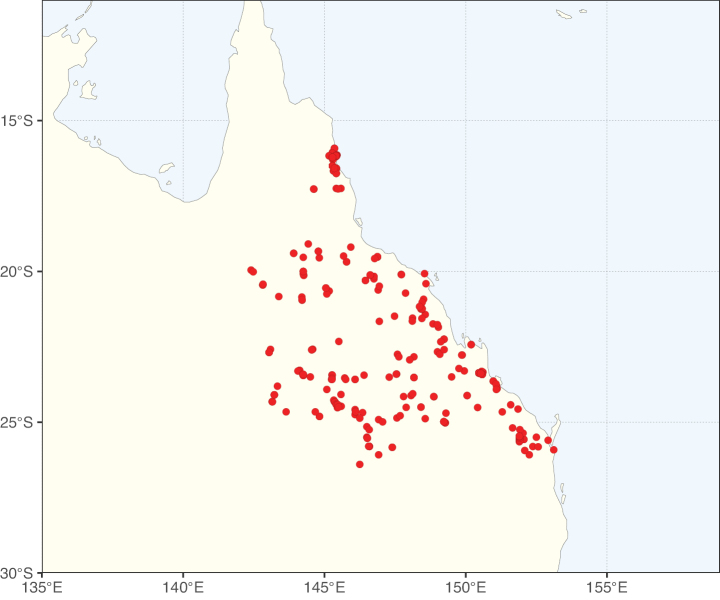
Distribution of *Archidendron* based on quality-controlled digitised herbarium records. See Suppl. material 1 for the source of occurrence data.

**Figure 235.**
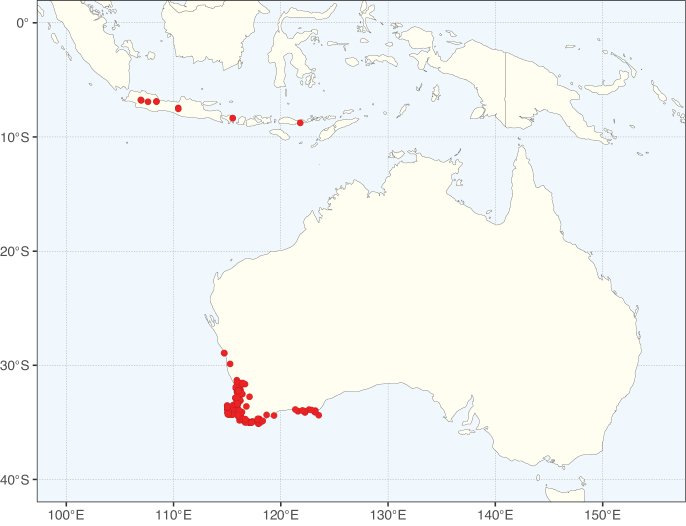
Distribution of *Heliodendron* based on quality-controlled digitised herbarium records. See Suppl. material 1 for the source of occurrence data.

**Figure 236.**
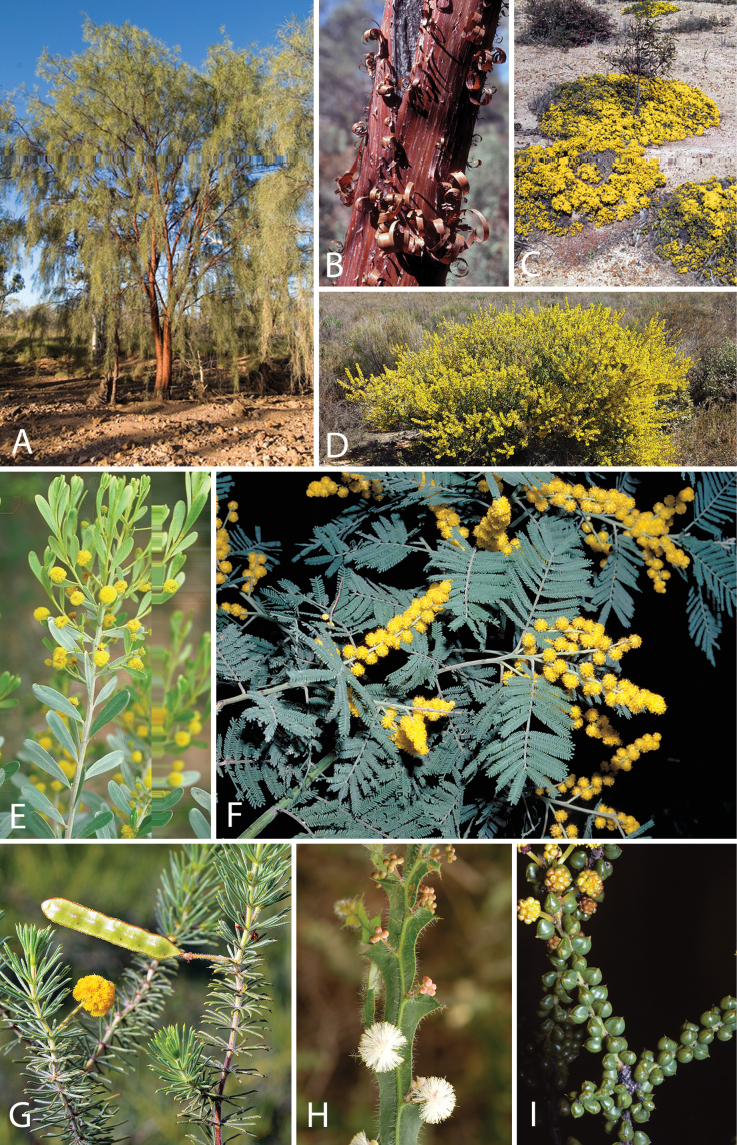
Distribution of *Paraserianthes* based on quality-controlled digitised herbarium records. See Suppl. material 1 for the source of occurrence data.

**Figure 237.**
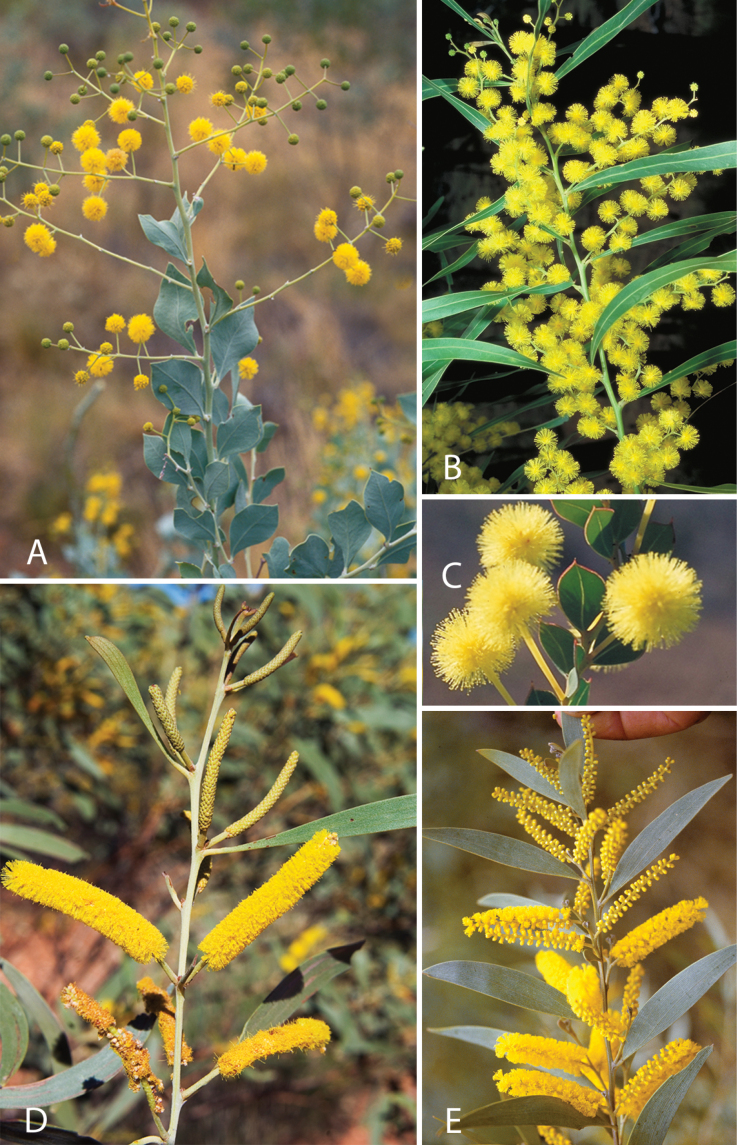
Diversity in habit and foliage of *Acacia***A** arborescent habit of A.cyperophyllavar.omearana Maslin **B** ‘Minni Ritchi’ bark of *A.grasbyi* Maiden **C** prostrate habit of *A.pulviniformis* Maiden **D** shrub habit of *A.brachybotrya* Benth. **E***A.argyrophylla* Hook. showing 1-veined phyllodes and globose inflorescences **F** bipinnate foliage of A.leucocladasubsp.argentifolia Tindale **G***A.spondylophylla* F. Muell. showing phyllodes arranged in regular whorls along branches **H**A.alatavar.biglandulosa Meisn. showing decurrent phyllodes that form bifarious wings along branches **I***A.tetraptera* Maslin showing unusually small phyllodes (*B.R. Maslin 5792*). Photo credits **A, C, E, H, I** B Maslin **B** A George **D** D Murphy **F** L Jessup **G** K Brennan.

**Figure 238.**
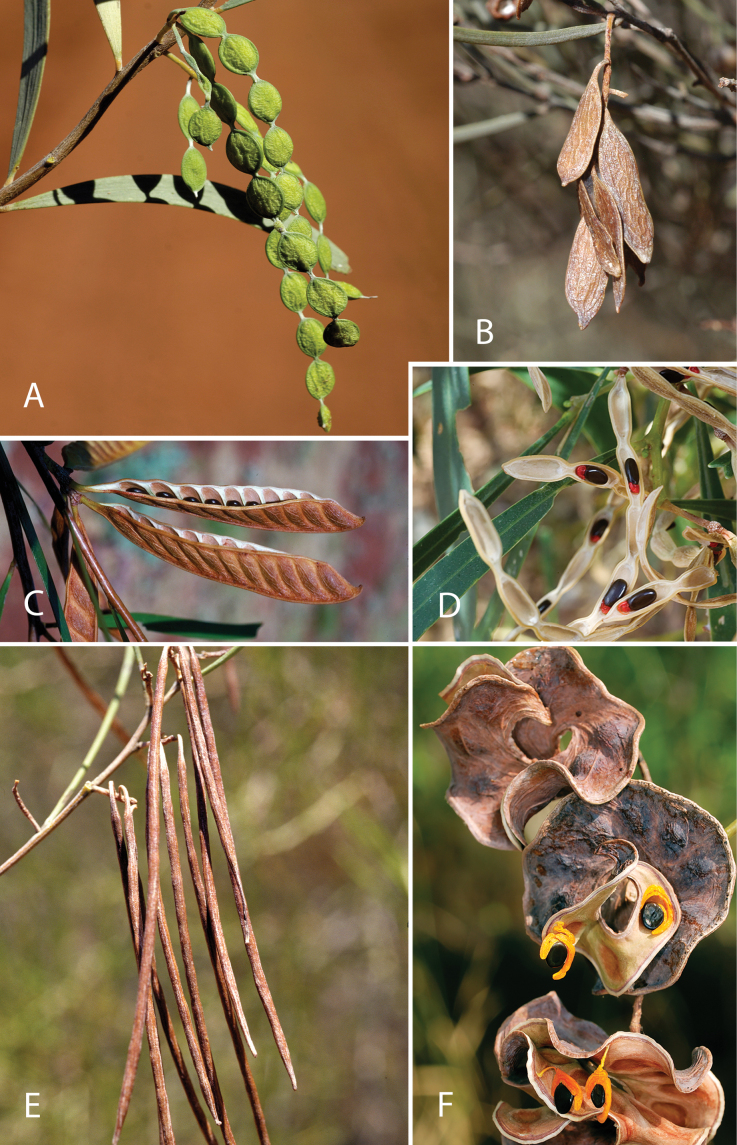
Diversity in inflorescences and flowers of *Acacia***A** heads in terminal panicle and racemes of A.pyrifoliaDC.var.pyrifolia (*B.R. Maslin 8425*) **B** heads in racemes of *A.neriifolia* A. Cunn. ex Benth. **C** axillary heads of A.heterochroaMaslinsubsp.heterochroa (*B.R. Maslin 4766*) **D** axillary spikes with densely arranged flowers of *A.fecunda* Maslin (*B.R. Maslin 8762*) **E** spikes with loosely arranged flowers and arranged in short racemes of *A.leptostachya* Benth. Photo credits **A, C, D** B Maslin **B** unknown **E** IB Armitage.

**Figure 239.**
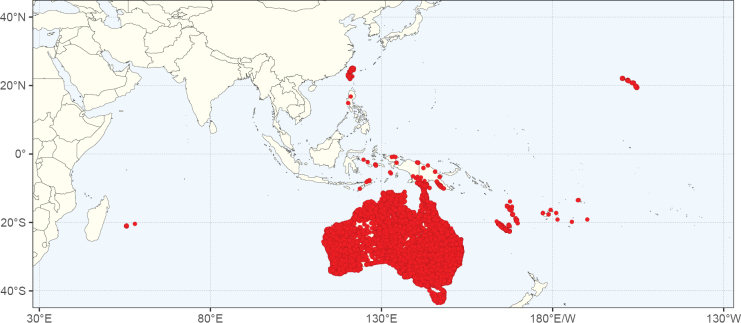
Diversity in fruits and seeds of *Acacia***A** pods of A.catenulatasubsp.occidentalis Maslin that readily break into 1-seeded loments (*B.R. Maslin 8880*) **B** fruits of the ‘Mulga’ species *A.incurvaneura* Maslin & J.E.Reid (*B.R. Maslin 9304B*) **C***A.ancistrocarpa* Maiden & Blakely fruits are superficially similar to those of *Calliandra***D***A.ampliceps* Maslin showing brittle fruits with seeds having bright red aril (bird dispersed) (*B.R. Maslin 8660*) **E***A.coolgardiensis* Maiden showing terete fruits **F** irregularly coiled fruits of *A.auriculiformis* A. Cunn ex Benth. showing seeds encircled by showy aril. Photo credits **A–E** B Maslin **F** K Brennan.

**Figure 240.**
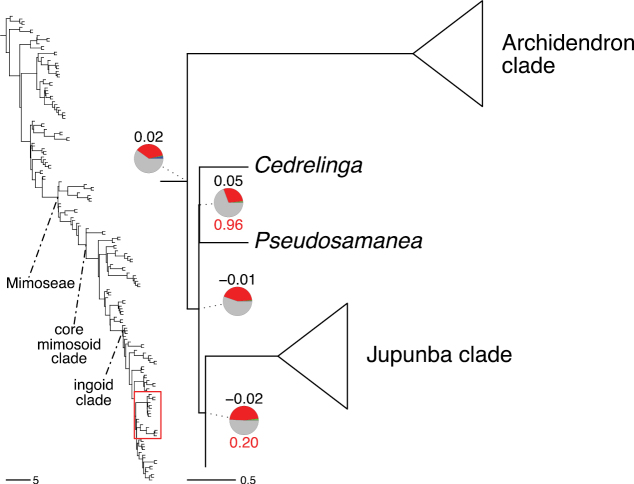
Distribution of *Acacia* based on quality-controlled digitised herbarium records. Note that *Acaciaconfusa* Merr. is widely planted in the Philippines and Taiwan, making it difficult to distinguish native from introduced occurrences. See Suppl. material 1 for the source of occurrence data.

**Notes.** The Archidendron clade was first identified by Brown et al. (2008), as the Australian & SE Asian Ingeae + *Acacia* s.s. clade, in a nuclear DNA phylogeny (ITS and ETS) of *Acacia* and tribe Ingeae. Seven of the nine genera were grouped together in a strongly supported clade, but the relationships amongst the genera were not resolved, with three alternate topologies presented. *Archidendropsis* and *Serianthes* were not sampled by Brown et al. (2008). Recent multilocus and phylogenomic studies based on hundreds of loci confirm the monophyly of the Archidendron clade, but still do not unequivocally resolve the relationships amongst the genera (Koenen et al. 2020a; Brown et al. 2022; Demeulenaere et al. 2022; Ringelberg et al. 2022). The clade is morphologically heterogeneous with no apparent synapomorphic characters uniting all nine genera. It is defined largely based on geography, and distinct from other clades in Caesalpinioideae in being biogeographically centered in the Indomalayan and Australasian regions (Koenen et al. 2020a). Although the clade includes the prominent and largest genus of Caesalpinioideae, *Acacia*, because the name Acacia clade has been used previously and applied to different generic assemblages (Miller et al. 2013), here we follow Koenen et al. (2020a) and for clarity name this lineage the Archidendron clade.

Within the Archidendron clade, only the Serianthes clade is consistently resolved, grouping *Falcataria*, *Serianthes*, and *Wallaceodendron*, with *Serianthes* and *Falcataria* strongly supported as sister genera (Koenen et al. 2020a; Demeulenaere et al. 2022; Ringelberg et al. 2022; Fig. 225). The three genera in the Serianthes clade have similar wood properties (Nielsen et al. 1983a). *Falcataria* and species of Serianthessubg.Minahassae I.C. Nielsen commonly have spike-like inflorescences, while *Wallaceodendron* has unbranched racemes with solitary axillary flowers (Nielsen et al. 1983b, 1984b; Demeulenaere et al. 2021). *Serianthes* differs from *Falcataria* and *Wallaceodendron* by its alternate leaflets and woody indehiscent fruits (Nielsen et al. 1983). Amongst the six other Archidendron clade genera, relationships remain unsupported despite large amounts of genomic data (Koenen et al. 2020a; Demeulenaere et al. 2022). Indeed, there is evidence to suggest that the backbone of the clade comprises a putative hard polytomy (Ringelberg et al. 2022). These genera are morphologically quite distinct.

### ﻿ Wallaceodendron

Taxon classificationPlantaeFabalesFabaceae

Koord., Meded. Lands Plantentuin 19: 630. 1898.

Figs 226 , 227 , 228 , 229

#### Type.

*Wallaceodendroncelebicum* Koord.

#### Description.

Unarmed medium-sized to large trees up to 45 m. **Stipules** caducous. **Leaves** bipinnate, extrafloral nectaries between pinnae pairs; pinnae 2–3 pairs; leaflets 3–6 pairs per pinna, opposite. **Inflorescence** a solitary or paired raceme, peduncle 5–16 cm long. **Flowers** uniform, bisexual, 5-merous, subtended by ca. 2 mm long, triangular, caducous bracts; calyx gamosepalous, valvate; corolla gamopetalous, valvate; stamens numerous, united into a tube at the base, staminal tube and corolla tube shortly united at the base; pollen in 16-celled polyads with a perforated tectum; ovary solitary. **Fruit** a woody legume, flat, straight to slightly curved, tardily dehiscent, not segmented, not reddish inside; exocarp thin, crustaceous, mesocarp woody, endocarp chartaceous loosening and at dehiscence forming small, closed envelopes around each seed. **Seeds** strongly flattened, circular, unwinged, with U-shaped pleurogram, without aril; testa with a thick sclerotesta.

#### Chromosome number.

2*n* = 26 (Goldblatt 1981a; Cannon et al. 2015).

#### Included species and geographic distribution.

Monospecific (*W.celebicum*), endemic to Malesia [North Sulawesi (Celebes) and the Philippines] (Fig. 229).

#### Ecology.

*Wallaceodendron* can be found along seashores and inland in primary rainforest to 850 m elevation.

#### Etymology.

Named after the British botanist Alfred Russel Wallace (1823–1913), explorer, zoologist, and plant collector (Quattrocchi 1999).

#### Human uses.

*Wallaceodendroncelebicum* [banúyo (=ironwood), derham mahogany] is used for furniture, flooring, light construction, telegraph poles, music instruments, decorative veneers, carvings and sculptures (Sosef et al. 1998; Nielsen 1992; Lewis and Rico Acre 2005).

#### Notes.

Fosberg (1960) commented on the possibility of combining *Wallaceodendron* with *Serianthes* based on the nearly identical flowers, but argued to keep them separate based on the unique features of fruit dehiscence in *Wallaceodendron* with small, closed envelopes of endocarp around each seed. These propagules are adapted to wind and water dispersal (Augspurger 1989; Nielsen 1992).

#### Taxonomic references.

Nielsen (1981a, 1992); Nielsen et al. (1983a); Sosef et al. (1998).

### ﻿ Falcataria

Taxon classificationPlantaeFabalesFabaceae

(I.C. Nielsen) Barneby & J.W. Grimes, Mem. New York Bot. Gard. 74(1): 254. 1996.

Figs 226 , 227 , 228 , 230

Paraserianthes sect. Falcataria I.C. Nielsen, Bull. Mus. Natl. Hist. Nat., B, Adansonia Sér. 4, 5(3): 327. 1983 (publ. 1984). Type: Paraserianthesfalcataria (L.) Nielsen [≡ Adenantherafalcataria L.]

#### Type.

*Falcatariafalcata* (L.) Greuter & R. Rankin [≡ *Falcatariamoluccana* (Miq.) Barneby & Grimes (≡ *Albiziamoluccana* Miq.)]

#### Description.

Unarmed medium-sized to large trees up to 40 m. **Stipules** caducous. **Leaves** bipinnate, extrafloral nectaries disk-shaped; pinnae 8–24 pairs; leaflets (4) 8–27 (33) pairs per pinna, opposite, subsessile. **Inflorescences** 2–3 times branched, efoliate panicles of few-flowered spikes, each panicle axillary to a fully expanded leaf, the terminal meristem of each annual branch-complement continuing beyond the fertile axes. **Flowers** bisexual, 5-merous, homomorphic and sessile; calyx gamosepalous, campanulate or hemispherical; corolla gamopetalous, sericeous; stamens numerous; pollen in 16-celled polyads with a thin exine and a thick nexine, without costae; intrastaminal disc around the base of the ovary. **Fruits** broad-linear, straight, plano-compressed, narrowly winged along the ventral suture, inertly dehiscent through both sutures. **Seeds** compressed ellipsoid, coat hard, testa brown, U-shaped pleurogram.

#### Chromosome number.

2*n* = 26 [*Albiziafalcata* (L.) Baker (= *Falcatariafalcata*)] (Huang et al. 1989).

#### Included species and geographic distribution.

Three species [*F.falcata*, *F.pullenii* (Verdc.) Gill.K. Br., D.J. Murphy & Ladiges, *F.toona* (F.M. Bailey) Gill.K. Br., D.J. Murphy & Ladiges], native to the Moluccas, New Guinea, Solomon Islands, and Queensland (Barneby and Grimes 1996) (Fig. 230). *Falcatariafalcata* is widely cultivated in Africa, Asia, South and Central America, and several Pacific Islands (Chudnoff 1985).

#### Ecology.

Tropical rainforest and coastal dry rainforests, spanning elevations up to 2300 m (Lewis and Rico Arce 2005).

#### Etymology.

The name *Falcataria* is derived from the Latin word *falcatus* which means “sickle-shaped, hooked” (Barneby and Grimes 1996), referring to the shape of leaflets (Islam 2005).

#### Human uses.

The wood is commercially used for pulpwood, veneers, light construction, crafts and furniture, fuel and charcoal (Ramano and Acda 2017). *Falcataria* is used in reforestation projects due to its rapid growth but also as a shade tree for cocoa and coffee and as an ornamental (Lewis and Rico Arce 2005). *Falcatariafalcata* (batai, peacock’s plume, white albizia, jeungjing) is an invasive weed across many Pacific Islands (Hughes et al. 2013).

#### Notes.

The molecular phylogenetic analyses of Brown et al. (2008, 2011) showed *Paraserianthes* sensu Nielsen (1983a) to be paraphyletic, supporting the classification of Barneby and Grimes (1996) who had raised sect. Falcataria to the generic level, and in consequence reduced *Paraserianthes* to only one species.

The name applied to the type species of the genus has a complicated history (see Jarvis 2007). The name commonly used over the past 57 years was recently changed to *Falcatariafalcata* following the rule of priority.

#### Taxonomic references.

Barneby and Grimes (1996); Chudnoff (1985); Nielsen et al. (1983b).

### ﻿ Serianthes

Taxon classificationPlantaeFabalesFabaceae

Benth., London J. Bot. 3: 225. 1844.

Figs 226 , 227 , 228 , 231

Albizia sect. Serianthes (Benth.) F. Muell., Fragm. 8: 165. 1874. Type: Albiziagrandiflora (Benth.) F. Muell. [≡ Serianthesdilmyi Fosberg]

#### Type.

*Serianthesgrandiflora* Benth.

#### Description.

Unarmed shrubs, 6–10 m, and trees to 27 m. **Stipules** linear to filiform, observed only in the seedling stage. **Leaves** bipinnate, extrafloral nectary circular or elliptical usually on the lower half of the petiole, additional nectaries usually on the leaf rachis and on the pinnae between the leaflets insertion; pinnae (4) 6–22 pairs, opposite or subopposite; leaflets (6) 8–25 (30) pairs per pinna, alternate except for the distal pair, sessile, mostly asymmetrical with a diagonal midvein. **Inflorescence** an umbel, raceme, or panicle composed of pedunculate spikes, pedunculate racemes or 1–4-flowered capitula. **Flowers** uniform, bisexual, 5-merous; calyx gamosepalous, valvate, usually circumscissile at the base; corolla gamopetalous, valvate, tube untied with the staminal tube in the lower part; stamens numerous, united into a tube at the base; pollen in 16-celled polyads with a perforated tectum in each locule; ovaries 1, less frequently 2, sessile. **Fruits** indehiscent to very tardily dehiscent (rarely), woody with transverse seeds which are isolated in a chamber each. **Seeds** flattened with a hard, black testa and a U-shaped pleurogram, lack a wing.

#### Chromosome number.

2*n* = 26 (only two counts of *Seriantheskanehirae* Fosberg are known) (Goldblatt 1981a; Cannon et al. 2015)

#### Included species and geographic distribution.

ca. 18 species, five subspecies, and two varieties, widely distributed in the Indo-Pacific region (Fig. 231). Fifteen species are restricted to the Pacific Islands of which six are endemic to New Caledonia, three occur in Indonesia, and three occur in New Guinea and/or Papua New Guinea. The distribution of *Serianthestenuiflora* Benth. is unknown. Most taxa are island endemics, except for *Serianthesdilmyi*, which is more broadly distributed in Malesia, Papuasia, and New Caledonia. *Serianthes* is the only genus within the Archidendron clade not found in Australia.

#### Ecology.

The more widespread *Serianthesdilmyi* Fosberg is adapted to supralittoral habitats. The other *Serianthes* taxa occur from coastal shrub, moist forest to lowland wet tropical rainforest on calcareous or volcanic soils, but in New Caledonia *Serianthes* is also found in maquis on ultramafic (serpentine) soils (Fosberg 1960; Nielsen et al. 1984b).

#### Etymology.

*Serianthes* is derived from the Greek *serikon* (= silk) and *anthos* (= flower) because the flowers of the tree have a silky appearance (Wagner et al. 1999).

#### Human uses.

The wood of *Serianthes* was used traditionally mostly for canoes, paddles, traditional boats, traditional and ceremonial houses, and for storyboards (Kanis 1979; Nielsen et al. 1984b; Demeulenaere et al. 2021). Other documented traditional uses are for medicine (Demeulenaere et al. 2021), dye (Kanis 1979), food (seeds), and necklaces (Nielsen et al. 1984b). Oral histories surrounding *Serianthes* teach the youth about respect and imbue spirituality (Demeulenaere et al. 2021). Commercially, species of *Serianthes* are used for paper pulp, plywood, and timber (Pari and Lestari 1993).

#### Notes.

Three main revisionary treatments (Fosberg 1960; Kanis 1979; Nielsen et al. 1984b) have been published since Bentham first described *Serianthes* in 1844. Nielsen et al. (1984b) recognised two subgenera: Serianthessubg.Minahassae and Serianthessubg.Serianthes. However, a recent phylogenetic study (Demeulenaere et al. 2022) of eight of the 18 species, identified two monophyletic groups whose taxon make-up does not support this subgeneric division. One lineage consists of taxa belonging to Serianthessubg.Serianthessect.Serianthes and Serianthessubg.Minahassae taxa, while the second lineage consists of Serianthessubg.Serianthessect.Calycina I.C. Nielsen taxa.

The distribution of certain *Serianthes* taxa across different islands (and a few mainland areas) results in vernacular names being expressed in either different languages or even the same language, but with a different name. For example, the Palauan name of Seriantheskanehiraevar.kanehirae Fosberg is ukall or kumer, while the Yapese name of Seriantheskanehiraevar.yapensis Fosberg is gumor. *Serianthesnelsonii* Merr. occurs on two islands of the Mariana Islands archipelago. The CHamoru name for the tree is håyun lågu on Guam and tronkon guåfi on Rota.

#### Taxonomic references.

Bentham (1844); Fosberg (1960); Kanis (1979); Nielsen et al. (1984b).

### ﻿ Archidendropsis

Taxon classificationPlantaeFabalesFabaceae

I.C. Nielsen, Fl. Nouv.-Calédon. Dépend. 12: 66. 1983.

Figs 227 , 228 , 232

Albizia sect. Spiciflorae Benth., London J. Bot. 3: 85. 1844. Type: Albiziafulgens (Labill.) Benth. [≡ Acaciafulgens Labill. (≡ Archidendropsisfulgens (Labill.) I.C. Nielsen)]
Albizia sect. Spiciflorae Benth. ser. Platyspermae Benth., Trans. Linn. Soc., London 30: 558. 1875. Type not designated.

#### Type.

*Archidendropsisfulgens* (Labill.) I.C. Nielsen [≡ *Acaciafulgens* Labill.]

#### Description.

Unarmed trees or shrubs. **Stipules** small ovate or filiform and often caducous, or large auriculate, orbicular, or cordate and persistent, never thorny. **Leaves** bipinnate, pinnae 1–14 pairs; leaflets 1–25 pairs per pinna, opposite or alternate; extrafloral nectaries circular, elliptic or obtriangular, on leaf rachis and sometimes also on the pinnae. **Inflorescences** in axillary or terminal spikes, spiciform racemes, racemes or in one species [*A.fournieri* (Vieill.) I.C. Nielsen] capitate, but when capitate the calyx and corolla are glabrous. **Flowers** uniform, bisexual, 4- or 5-merous, red, white, green, yellow or pink, usually subtended by very small tetrameric or pentameric bracts; calyx gamosepalous, cupular to campanulate; corolla gamopetalous, funnel shaped; stamens numerous, filaments united into a tube at the base; pollen in 16 or 32-celled polyads, tectum with non-isometric channels; ovaries mostly one, several in *A.oblonga* (Hemsl.) I.C. Nielsen. **Fruits** usually flat and dehiscent along both sutures, not partitioned inside and endocarp not separating from the exocarp. **Seeds** flattened or swollen, narrowly winged when flattened, thin-walled and lacking a pleurogram.

#### Chromosome number.

Unknown.

#### Included species and geographic distribution.

Eleven species from New Caledonia, New Britain, the Solomon Islands and on the island of New Guinea (Fig. 232).

#### Ecology.

Most species with a restricted area of distribution, found in rainforests, gallery forest, wooded ravines, or mesophyllous forest.

#### Etymology.

From the generic name *Archidendron* with the Latin suffix -opsis, referring to the similarity to that genus.

#### Human uses.

Unknown.

#### Notes.

*Archidendropsis* was published as “Gen. B” by Nielsen (1981b). The genus is now restricted to the former Archidendropsissubg.Archidendropsis after the erection of *Heliodendron* by Brown et al. (2022). Further sampling of species of *Archidendropsis* in phylogenetic studies would be beneficial, particularly to ascertain the relationships of the capitulate-flowered *A.fournieri* and the non-New Caledonian representatives of *Archidendropsis*.

#### Taxonomic references.

Cowan (1998); Nielsen (1983); Nielsen et al. (1983b).

### ﻿ Pararchidendron

Taxon classificationPlantaeFabalesFabaceae

I.C. Nielsen, Bull. Mus. Natl. Hist. Nat., B, Adansonia 5: 327–328. 1983.

Figs 226 , 227 , 228 , 233

#### Type.

*Pararchidendronpruinosum* (Benth.) I.C. Nielsen [≡ *Pithecellobiumpruinosum* Benth.]

#### Description.

Unarmed trees or shrubs. **Stipules** linear not spinescent, usually inconspicuous and caducous. **Leaves** bipinnate, extrafloral nectaries flat to concave on leaf rachis and pinnae; pinnae 1–4 pairs; leaflets 3–12 pairs per pinnae, alternate, petiolulate. **Inflorescence units** globose, umbellate, clustered in axillary, pedunculate racemes. **Flowers** uniform, bisexual, 5-merous, pedicellate; calyx gamosepalous, cupular to hemispherical; corolla gamopetalous, tubular to slightly funnel-shaped, valvate; stamens numerous, united into a tube at the base and shortly united with the corolla tube; pollen in 16-celled polyads, non-isometric channels crossing +/- entirely the tectum, non-radially oriented; ovary solitary, stipitate. **Fruit** curved into a circle to contorted, chartaceous, flattened, not segmented, dehiscent first along ventral suture, reddish inside, the endocarp not forming envelopes around each seed. **Seeds** ellipsoid, obovoid or subglobose, with U-shaped pleurogram, without aril, with a thick, black sclerotesta, unwinged.

#### Chromosome number.

Unknown.

#### Included species and geographic distribution.

Monospecific (*P.pruinosum*), with four varieties, in Java, Lesser Sunda Islands (Sumbawa, West Flores), Timor-Leste, the island of New Guinea, and Australia (Queensland and New South Wales) (Fig. 233).

#### Ecology.

In Australia this species is reported from the rainforest, coastal scrub and semi-deciduous forest (sea level to 800 m); in the Malesian area it is found in montane rainforest, both primary and secondary (400–2250 m).

#### Etymology.

The Greek prefix *para* (= close by) alludes to the similarity of this genus to *Archidendron* (Cowan 1998).

#### Human uses.

*Pararchidendron* is sold in Australia as a horticultural decorative plant that can be a screen break or shade and produces a decorative general purpose timber (Cause et al. 1989).

#### Notes.

*Pararchidendron* was identified as “Gen. C” by Nielsen (1981b). The monospecific genus *Pararchidendron* has not been reviewed since it was described by Nielsen et al. (1983a), although the varieties appear to be morphologically consistent across their ranges. Its position in the Archidendron clade is not resolved and sampling in phylogenetic studies has been limited to the Australian variety (var. pruinosum). Pararchidendronpruinosumvar.pruinosum is restricted to Australia, while the three other varieties are restricted to Malesia and are largely differentiated by the indumentum and shape of the leaflet apex.

#### Taxonomic references.

Cowan (1998); Nielsen (1992); Nielsen et al. (1983a, 1984b).

### ﻿ Archidendron

Taxon classificationPlantaeFabalesFabaceae

F. Muell., Fragm. 5: 59. 1865.

Figs 226 , 227 , 228 , 234

Pithecellobium sect. Clyplearia Benth., London J. Bot. 3: 206. 1844. Type: Pithecellobiumclypearia (Jack) Benth. [≡ Ingaclypearia Jack (≡ Archidendronclypearia (Jack) I.C. Nielsen)]
Ortholobium Gagnep., Bull. Soc. Bot. Fr. 99: 36. 1952, nom. inval. (no Latin descr.)
Cylindrokelupha Kosterm., Bull. Org. natuurw. Onderz. 20: 20. 1954. Type: Cylindrokeluphabubalina (Jack) Kosterm. [≡ Ingabubalina Jack (≡ Archidendronbubalinum (Jack) I.C. Nielsen)]
Morolobium Kosterm., Bull. Org. natuurw. Onderz. 20: 20. 1954. Type: Morolobiummonopterum (Kosterm.) Kosterm. [≡ Pithecellobiummonopterum Kosterm. (≡ Archidendronmonopterum (Kosterm.) I.C. Nielsen]
Paralbizzia Kosterm., Bull. Org. natuurw. Onderz. 20: 23. 1954. Type: Paralbizziaturgida (Merr.) Kosterm. [≡ Pithecellobiumturgidum Merr. (≡ Archidendronturgidum (Merr.) I.C. Nielsen)]

#### Type.

*Archidendronvaillantii* (F. Muell.) F. Muell. [≡ *Pithecellobiumvaillantii* F. Muell.]

#### Description.

Unarmed trees or shrubs, small to medium sized. **Stipules** present, sometimes glandular, or absent. **Leaves** bipinnate, rarely unifoliolate; pinnae 1–14 pairs; leaflets mostly opposite, rarely alternate (3 species), variable in number, shape and size; extrafloral nectaries sessile, sunken, raised or stipitate, round, boat-shaped, triangular or irregular shaped present on the rachis, additional glands present (various combinations and shapes) or absent on the pinnae. **Inflorescences** simple or compound in pedunculate capitula, umbels, corymbs or racemes, if compound may be arranged in cauliflorous, ramiflorous, axillary or terminal panicles; extrafloral nectaries sometimes present on floral bracts and capitula. **Flowers** uniform, bisexual or unisexual, 4- or 5-merous, yellow, green, white or cream; calyx gamosepalous; corolla gamopetalous; stamens numerous, united into a tube at the base and joined with the corolla in the lower part; pollen in (4, 12) 16 (32)-celled polyads; ovary 1–several per flower, sessile or stipitate. **Fruits** chartaceous, coriaceous, fleshy or woody, straight, curved or spirally twisted, flat to terete, sometimes internally segmented, dehiscing either along the dorsal or ventral suture, sometimes along both sutures, most often reddish outside and orange-reddish inside. **Seeds** may be funiculate, ellipsoid, or flattened, with a black or bluish black testa, pleurogram lacking, unwinged.

#### Chromosome number.

2*n* = 26. Only three species [*A.clypearia*, *A.jiringa* (Jack) I.C. Nielsen, *A.turgidum*] have published chromosome numbers (Rice et al. 2015).

#### Included species and geographic distribution.

Ninety-nine species described in this Indomalayan-Australasian genus and an additional 20 putative species that are poorly known due to limited collections or destroyed types. *Archidendron* is distributed from Kerala (southern India) and Sri Lanka in the west, to the Solomon Islands in the east; and from Taiwan and the Ryukyu Islands in the north, to Australia in the south (Fig. 234).

#### Ecology.

Lowland and montane tropical and subtropical rainforests.

#### Etymology.

According to Nielsen (1981b) the name of the genus comes from the Greek *archi* (= dominant, principal) and *denderon* (= tree), translating the remark of F. von Mueller concerning the dominance of *Archidendronvaillantii* (F. Muell.) F. Muell. in northern Australia. However, Cowan (1998) noted it was from the Greek *arche* (= beginning) and *dendron* (= tree) with the occurrence in several species of more than a single pistil in each flower, which can be construed as a characteristic of an earlier stage in the evolution of flowering plants.

#### Human uses.

The seeds of *A.jiringa* are eaten in Thailand, Malaysia and Indonesia in a dish known as “jenkol”; the young shoots are also eaten. *Archidendronjiringa* has also been used as timber, dye (from the pods) and the leaves (and those of *A.lucidum*) have been used for traditional medicine (e.g., for the treatment of diabetes, inflammatory diseases and cancer) (Charungchitrak et al. 2010; Shukri et al. 2011; Liu et al. 2011).

#### Notes.

Recent evidence from molecular phylogenies suggests that *Archidendron* is non-monophyletic (Brown et al. 2022). Instead *Archidendron* is divided into two largely geographic clades: the Clypeariae clade sensu Brown et al. (2022) primarily distributed in western Malesia and mainland Asia, and the Archidendron s.s. clade sensu Brown et al. (2022) mostly restricted to eastern Malesia and Australia. However, resolution of the topological uncertainty between these two clades and discrete macromorphological characters to delineate them are required before nomenclatural changes can be made. Furthermore, most of the infrageneric series proposed by Nielsen et al. (1984a) are also not monophyletic (Brown et al. 2022). In addition, more detailed taxonomic study is still required, especially for the large number of insufficiently known species and the widespread morphologically variable species, such as *A.clypearia*.

#### Taxonomic references.

Cowan (1998); Nielsen (1981a, 1984a).

### ﻿ Heliodendron

Taxon classificationPlantaeFabalesFabaceae

Gill.K. Br. & Bayly, PhytoKeys 205: 321. 2022.

Figs 226 , 227 , 228 , 235

Archidendropsis subg. Basaltica I.C. Nielsen, Bull. Mus. Natl. Hist. Nat., B, Adansonia Sér. 4, 5(3): 325. 1984. Type: Archidendropsisbasaltica (F. Muell.) I.C. Nielsen [≡ Acaciabasaltica F. Muell. (≡ Heliodendronbasalticum (F. Muell.) Gill.K. Br. & Bayly)]

#### Type.

*Heliodendronbasalticum* (F. Muell.) Gill.K. Br. & Bayly [≡ *Acaciabasaltica* F. Muell.]

#### Description.

Trees or shrubs to 37 m. **Stipules** either resembling small thorns to 1.2 mm long that are caducous, or persistent circular-ovate glands 1–3 mm in diameter. **Leaves** bipinnate; extrafloral nectaries at junction of pinnae circular or triangular to rhombic, circular glands sometimes at junction of leaflet petiolules; pinnae 1–2 pairs; leaflets 1.5–11 pairs per pinna, opposite, subsessile or long (3.5–7 mm) petiolulate, elliptic to elliptic-lanceolate or oblong. **Inflorescence** capitula, either simple or arranged into a panicle to 35 cm long. **Flowers** uniform, bisexual, 4- or 5-merous, yellow to cream, hairy, sessile; calyx gamosepalous, tubular to subcampanulate, symmetrical; corolla gamopetalous, tubular to narrowly campanulate; stamens numerous, united basally into a tube that equals or slightly exceeds the corolla tube; pollen in 16-celled polyads, tectum with isometric channels; ovary solitary and shortly stipitate. **Fruits** brown, valves chartaceous, oblong, flat and dehiscing along both sutures. **Seeds** lacking a pleurogram, flat, circular to ovate or obliquely ovate, with a narrow 0.2–1 mm peripheral, membranous wing.

#### Chromosome number.

Unknown.

#### Included species and geographic distribution.

Three species restricted to the state of Queensland in Australia (Fig. 235).

#### Ecology.

One species [*H.xanthoxylon* (C.T. White & W.D. Francis) Gill.K. Br. & Bayly] is found in rainforest remnants on granitic soils whereas the other two species [*H.basalticum* and *H.thozetianum* (F. Muell.) Gill.K. Br. & Bayly] are found in drier habitats on sandy to sandy-clay loam soils, either in eucalypt woodland, acacia shrubland or semi-deciduous vine thickets.

#### Etymology.

From *helios* (Greek = sun) in reference to the endemic distribution in the Australian state of Queensland, widely known as the “sunshine state”, and to the capitate, sun-like inflorescences of yellow flowers, and *dendron* (Greek = tree) to the tree habit.

#### Human uses.

*Heliodendron* has been used as timber for general building and the manufacture of window frames, cabinets, barrels and boat building (Swain 1928).

#### Notes.

*Heliodendron* was newly described for the morphologically distinct Archidendropsissubg.Basaltica after phylogenetic studies found the two subgenera of *Archidendropsis* to be non-monophyletic (Brown et al. 2022). The rainforest *H.xanthoxylon* has larger leaflets and fewer leaflets per pinna than the two drier habitat species, but they all share the diagnostic features of the genus (inflorescences in capitula, seeds flat, lacking a pleurogram and with a narrow peripheral membranous wing, calyx and corolla with hairs, pods narrowly oblong, brown, opening along both sutures, pollen polyad diameter of 55–68 μm and pollen tectum with isometric channels) and there would be little benefit in separating *H.xanthoxylon* as a monospecific genus.

#### Taxonomic references.

Brown et al. (2022); Cowan (1998); Nielsen (1981a).

### ﻿ Paraserianthes

Taxon classificationPlantaeFabalesFabaceae

I.C. Nielsen, Bull. Mus. Natl. Hist. Nat., B, Adansonia 5: 326. 1983.

Figs 226 , 227 , 228 , 236

Albizia sect. Lophantha ser. Pachyspermae Benth., Trans. Linn. Soc., London 30: 559. 1875, nom. illeg. Type: Albizialophantha (Willd.) Benth. [≡ Acacialophantha Willd. (≡ Paraseriantheslophantha (Willd.) I.C. Nielsen)]
Albizia sect. Pachyspermae (Benth.) Fosberg, Reinwardtia 7: 74. 1965. Type: Albizialophantha (Willd.) Benth. [≡ Acacialophantha Willd. (≡ Paraseriantheslophantha (Willd.) I.C. Nielsen)]

#### Type.

*Paraseriantheslophantha* (Willd.) I.C. Nielsen [≡ *Acacialophantha* Willd.]

#### Description.

Unarmed shrub or tree to 10 m. **Stipules** to 2 mm long, either linear, puberulous and caducous (P.lophanthasubsp.lophantha), or subcordate-triangular to ovate-lanceolate and densely tomentose [P.lophanthasubsp.montana (Jungh.) I.C. Nielsen]. **Leaves** bipinnate, extrafloral nectaries present, elliptic to oblong on the petiole, smaller circular glands sometimes between the terminal pairs of pinnae and/or leaflets; pinnae 7–14 pairs, opposite; leaflets 15–40 pairs, sessile, opposite, inequilaterally narrowly oblong to lanceolate to oblong. **Inflorescences** solitary or paired axillary racemes, 5.2–18 cm long. **Flowers** uniform, bisexual, 5-merous, yellowish-green; calyx gamosepalous, narrowly cup-shaped; corolla gamopetalous, funnel-shaped; stamens numerous and united into a tube at the base; pollen in 16-celled polyads, with costae (pores surrounded by distinct thickenings), surface with few tectal channels; ovary sessile (P.lophanthasubsp.montana) or shortly stipitate to subsessile (P.lophanthasubsp.lophantha). **Fruits** chartaceous, flat, straight-edged and dehiscent along both sutures. **Seeds** subcircular-elliptic or oblong, black, hard testa, flat or convex and wingless, with U-shaped pleurogram.

#### Chromosome number.

2*n* = 26 (Tjio 1948; Fedorov 1969; Rice et al. 2015).

#### Included species and geographic distribution.

Monospecific (*P.lophantha*), with two recognised subspecies: P.lophanthasubsp.lophantha, native to south-west Western Australia, and P.lophanthasubsp.montana, native to Sumatra and Java, and the Lesser Sunda islands of Bali and Flores (Fig. 236). *Paraseriantheslophantha* is naturalised in eastern Australia and has become a significant weed in South Africa, the Canary Islands, Chile, New Zealand, Portugal, southern California, and South America (Brown et al. 2020).

#### Ecology.

In Australia, *Paraserianthes* occurs in coastal forests, and coastal or near-coastal open eucalypt forest, thicket, shrubland and grassland (Cowan 1998; Lewis and Rico Arce 2005). In Malesia, subspecies montana is found in montane forests, elfin forests and on grassy plains on crater-slopes (Nielsen et al. 1983b).

#### Etymology.

The Greek prefix *para* (= close by) refers to the similarity of this genus to *Serianthes* (Cowan 1998).

#### Human uses.

*Paraseriantheslophantha* is an ecologically, horticulturally and economically important species across the tropics. It has been planted as part of reforestation programs for its rapid growth, as an ornamental, and also as a shade tree in cocoa and coffee plantations (Nielsen 1992; Barneby and Grimes 1996; Lewis and Rico Arce 2005). Fruits, wood and bark of *Paraserianthes* have been used for human food, firewood, charcoal, paper pulp, crates, light furniture and fibre for packing purposes and also as substitute for soap (Nielsen 1992; Lewis and Rico Arce 2005).

#### Notes.

*Paraserianthes* was originally described with two sections (sect. Paraserianthes and sect. Falcataria; Nielsen et al. 1983a). These were later raised to generic rank by Barneby and Grimes (1996) reducing *Paraserianthes* to a monospecific genus. Phylogenetic data supports this segregation (Brown et al. 2011, 2022; Demeulenaere et al. 2022; Ringelberg et al. 2022), but the relationship between the two geographically disjunct subspecies has not been tested as P.lophanthasubsp.montana has not been sampled in genetic studies.

#### Taxonomic references.

Barneby and Grimes (1996); Brown et al. (2011); Cowan (1998); Nielsen (1992); Nielsen et al. (1983b).

### ﻿ Acacia

Taxon classificationPlantaeFabalesFabaceae

Mill., Gard. Dict. Abr., ed. 4, [25]. 1754 nom. cons.

Figs 237 , 238 , 239 , 240

Acacia sect. Phyllodineae DC., Prodr. [A.P. de Candolle] 2: 448. 1825. Type not designated.
Racosperma Mart., Hort. Reg. Monac.: 188. 1829, nom. inval. (name not accepted by author)
Phyllodoce Link, Handbuch 2: 132. 1829, non Phyllodoce Salisb., Parad. Lond. ad t. 36. 1806 (Ericaceae). Type not designated.
Racosperma Mart., Index Seminum [München (Monacensis)]: 4. 1835. Lectotype (designated by Pedley 1986): Racospermapenninerve (Sieber ex DC.) Pedley [≡ Acaciapenninervis Sieber ex DC.]
Cuparilla Raf., Sylva Tellur.: 120. 1838. Lectotype (designated by Pedley 1986): Cuparillamyrtifolia (Sm.) Raf. [≡ Mimosamyrtifolia Sm. (≡ Acaciamyrtifolia (Sm.) Willd.)]
Drepaphyla Raf., Sylva Tellur.: 120. 1838. Lectotype (designated by Pedley 1986): Drepaphylalanigera (Cunn.) Raf. [≡ Acacialanigera A. Cunn.]
Hectandra Raf., Sylva Tellur.: 120. 1838. Lectotype (designated by Pedley 1986): Hectandrasuaveolens (Sm.) Raf. [≡ Mimosasuaveolens Sm. (≡ Acaciasuaveolens (Sm.) Willd.)]
Zigmaloba Raf., Sylva Tellur.: 120. 1838. Type: Zigmalobasulcata (R. Br.) Raf. [≡ Acaciasulcata R. Br.]
Chithonanthus Lehm., Pl. Preiss. 2: 368. 1848. Type: Chithonanthusrestiaceus (Benth.) Lehm. [≡ Acaciarestiacea Benth.]
Tetracheilos Lehm., Pl. Preiss. 2: 368. 1848. Type: Tetracheilostetragonocarpa Meisn., nom. illeg.
Acacia sect. Phyllodoce (Link) Kuntze, in T.E. von Post & C.E.O. Kuntze, Lex. Gen. Phan.: 2. 1903. Type not designated.
Phytomorula Kofoid, Univ. Calif. Publ. Bot. 6: 38. 1914. Type: Phytomorularegularis Kofoid. [described as an alga but shown to be pollen of Acacia sp., vide Copeland 1937].

#### Type.

*Acaciapenninervis* Sieber ex DC.

#### Description.

Trees, shrubs or subshrubs, never lianas; prickles absent. **Stipules** normally present and caducous, rarely spinose. **Leaves** bipinnate or modified to polymorphic phyllodes, rarely reduced to scales or absent; extrafloral nectaries normally present. **Inflorescences** globose or oblongoid capitula or spikes, axillary or aggregated in racemes or infrequently panicles, pedunculate or sometimes sessile. **Flowers** bisexual or staminate and bisexual within a single inflorescence, 5-merous or sometimes 4-merous, uniform, white to golden, rarely mauve-pink or red; sepals free to united, very rarely absent; corolla connate, valvate; stamens numerous, normally free, rarely basally united; pollen in polyads normally 8, 12 or 16 grains, the grains extraporate or sometimes porate, surface with pseudocolpi, exine granular (i.e., lacking columellae that occur in *Vachellia* Wight & Arn.); ovary solitary or very rarely 2–5 (e.g., *A.celastrifolia* Benth.). **Fruits** variable, dehiscent, rarely indehiscent. **Seeds** normally with a pleurogram and without endosperm; funicle arillate or sometimes exarillate.

#### Chromosome number.

Mostly 2*n* = 26 with ca. 100 species examined (Hamant et al. 1975; Goldblatt and Johnson 1979–; Rice et al. 2015), but there are a few notable exceptions and examples of polyploidy. In several members of the ‘Mulga’ group (i.e., *Acaciaaneura* F. Muell. ex Benth. and close relatives (Miller et al. 2002; Maslin and Reid 2012), which predominate in the arid zone, tetraploids (2*n* = 52), triploids (2*n* = 39) and pentaploids (2*n* = 65) were reported by Andrew et al. (2003). *Acaciabrachystachya* Benth., a close relative of the ‘Mulga’ group, is tetraploid (Hamant et al. 1975). There are a few other arid zone species not closely related to the ‘Mulga’ group that are polyploids (Hamant et al. 1975; Moran et al. 1992; Maslin and Thomson 1992). Two extra-Australian tetraploid species are *A.koa* A. Gray (Hawaii) and *A.heterophylla* (Lam.) Willd. (Reunion Island), whereas their close relative, the widespread, eastern Australian species, *A.melanoxylon* R. Br., is diploid (Brown et al. 2012). Natural triploids and tetraploids have also been recorded in populations of the Australian bipinnate-leaved species, *Acaciadealbata* Link. (Blakesley et al. 2002; Nghiem et al. 2018).

#### Included species and geographic distribution.

1082 species, the majority in Australia. These species are distributed throughout the continent with the major centre of species-richness located in south-west Western Australia, and secondary centres of richness in eastern Australia south of the Tropic of Capricorn associated with the Great Dividing Range, and in northern and north-eastern Australia. Although species of *Acacia* are a conspicuous component of the central Australian arid zone, this is a relatively species-poor region (Hnatiuk and Maslin 1988). Only 17 species occur naturally outside Australia (of which seven species plus one subspecies also occur within Australia), where they extend to South East and East Asia (north to Taiwan), the Pacific Ocean (east to Hawaii) and Indian Oceans (i.e., Mascarene islands); see Pedley (1975), Brown et al. (2012), WorldWideWattle (2022) (Fig. 240). Species of *Acacia* are widely distributed globally as weeds, ornamentals or cultivated for economic, social or environmental purposes.

#### Ecology.

Species of *Acacia* within Australia grow in a wide range of habitats, from coastal to subalpine, tropical to arid ecological vegetation classes. They are particularly conspicuous and common in many semi-arid and sub-tropical shrublands and woodlands, and while they dominate much of the arid zone the species numbers are, relatively speaking, not especially high. The diverse shrublands of Western Australia and *Eucalyptus* woodlands of eastern Australia associated with the Great Dividing Range, located between the arid and temperate zones, are especially species-rich, but *Acacia* is often not a dominant element of the vegetation (Hnatiuk and Maslin 1988).

#### Etymology.

It is normally regarded that the name *Acacia* is derived from the Greek, *ake* (= a point), in reference to the spiny stipules that characterised the first (African) species described as *Acacia*; these species now belong to the genus *Vachellia*.

#### Human uses.

Acacias have had extensive utilisation for economic, social and environmental purposes. A discussion of these uses is presented by McDonald et al. (2001) and a listing of them is given in the “Info Gallery/Utilisation” at WorldWideWattle (2022).

Commercially, the most important use of *Acacia* is for wood products where the species *A.auriculiformis* A. Cunn. ex Benth., *A.crassicarpa* A. Cunn. ex Benth., *A.mangium* Willd. and A.×mangiiformis Maslin & L.A.J. Thomson are especially important in tropical forestry plantation industries, particularly in south-east Asia and India (Griffin et al. 2011; Maslin et al. 2019b). *Acaciamearnsii* De Wild., which was initially grown for tannin production, is now highly regarded by the pulp and paper industries in several countries, most notably, Brazil, India and South Africa (Griffin et al. 2011). Within Australia the wood of *A.melanoxylon* (Blackwood) provides a niche market for fine furniture (Beadle and Brown 2007). The potential of *Acacia* species as a new woody crop plant to assist with salinity control in the agricultural regions of southern Australia (within the 250–650 mm rainfall zone) was assessed by Maslin and McDonald (2004).

As noted by Griffin et al. (2011), a suite of multi-purpose *Acacia* species have been introduced in dry zone regions of Africa and elsewhere to assist with meeting the demand for food, fodder, fuelwood, poles, and site amelioration (Doran and Turnbull 1997). *Acaciasaligna* (Labill.) H. Wendl. is the most widely planted of the non-timber species, with around 600,000 ha established worldwide; it is used as an animal fodder, in landscape amelioration projects, and for a range of other purposes (Midgley and Turnbull 2003; Maslin 2011). *Acaciacolei* Maslin & L.A.J. Thomson has been used as a human food and has been incorporated into local farming systems in Sub-Saharan Africa (Rinaudo and Cunningham 2008). The potential of this and other species of *Acacia* as a human food within Australia was assessed by Maslin et al. (1998).

Several species of *Acacia* have displayed significant invasiveness in places where they have been introduced. Examples include *A.cyclops* A. Cunn. ex G. Don, *A.baileyana* F. Muell., *A.dealbata* Link, *A.mangium*, *A.melanoxylon*, *A.pycnantha* Benth. and *A.saligna*. Factors promoting the spread of such species as weeds include the production of large quantities of tough, long-lived seeds, prolific seedling recruitment following fire or other environmental disturbances, and the absence of natural invertebrate predators and fungal pathogens. Richardson et al. (2011) provides a good introduction and review of this subject.

Indigenous Australians have long used species of *Acacia* as a source of food and medicine, tools and weapons, and various other purposes (e.g., Aboriginal Communities of the Northern Territory 1993; Latz 1995; Searle 2004; WorldWideWattle 2022).

As discussed at WorldWideWattle (2022), *Acacia* has great symbolic significance to Australians where it is the National Flora Emblem, is incorporated into the Australian Coat of Arms, and more.

#### Notes.

In recent years there have been substantial changes to both the classification and nomenclature of *Acacia*. The reasons for this are twofold. Firstly, molecular and other evidence have shown that the former broadly circumscribed, pantropical genus *Acacia* is polyphyletic and should be treated as comprising at least seven genera (see below). Secondly, the name *Acacia* is now conserved with a new type, namely, the Australian species *Acaciapenninervis* Sieber ex DC. which replaces the Afro-Asian species, *Acacianilotica* (L.) Willd. ex Delile. This retypification has meant that about half of the non-Australian species formerly called *Acacia* are now *Vachellia* and the name *Racosperma* Mart. [preferred by Pedley (1986) for most Australian species] is now a synonym of *Acacia*. There is an extensive literature regarding these two interrelated matters, but useful summaries are presented in Murphy (2008), Miller and Seigler (2012), Maslin (2015) and WorldWideWattle (2022). Summaries of issues concerning the retypification for the name *Acacia* are provided in McNeill and Turland (2010), Moore et al. (2010) and Thiele et al. (2011). Some of the more important phylogenetic studies that have demonstrated the non-monophyly of *Acacia* s.l. include Miller and Bayer (2000, 2001), Luckow et al. (2003), Murphy et al. (2003), Kyalangalilwa et al. (2013), Miller et al. (2017) and Koenen et al. (2020a). The current correct names for all species of the former broadly defined genus *Acacia* are available at WorldWideWattle (2022).

For several years prior to the fragmentation of *Acacia* s.l. the genus was commonly viewed as comprising three subgenera as defined by Vassal (1972), namely, Acaciasubg.Acacia (now *Vachellia*), Acaciasubg.Aculeiferum Vassal (now *Senegalia* Raf.) and Acaciasubg.Heterophyllum Vassal (= subg. Phyllodineae (DC.) Ser., now *Acacia*). Vassal’s classification was based primarily on characters of seedling ontogeny and seed morphology from 127 species (representing less than 10% of those recognised for the genus at that time), supplemented by data on other characters, including pollen of Guinet (1969). These subgenera broadly corresponded to groupings of six series of *Acacia* that Bentham (1875) had previously recognised and which had been used to accommodate species of the genus. Discussions of Vassal’s classification in relation to Bentham’s is provided in Maslin (2001) and Murphy (2008) for Australian species and by Ross (1979) for African species. Although Vassal’s subgenera (but not his lower-order categories) were adopted by some authors (e.g., Nielsen 1992), Pedley (1986) elevated them to genera: *Acacia* (= Acaciasubg.Acacia sensu Vassal, now *Vachellia*), *Racosperma* (= Acaciasubg.Heterophyllum≡subg.Phyllodineae, now *Acacia*) and *Senegalia* (= subg. Aculeiferum). However, Pedley’s (1986) classification was not widely adopted at that time and it was not until later that genetic evidence clearly showed that *Acacia* s.l. was polyphyletic (e.g., Luckow et al. 2003; Miller and Seigler 2012) and that multiple genera for *Acacia* s.l. were accepted and the fragmentation of the genus began. This resulted in seven currently recognised genera: *Acacia* s.s., *Acaciella* Britton & Rose (15 species, New World), *Mariosousa* Seigler & Ebinger (14 species, New World), *Parasenegalia* Seigler & Ebinger (11 species, New World), *Pseudosenegalia* Seigler & Ebinger (two species, New World), *Senegalia* Raf. (219 species, pantropical) and *Vachellia* (164 species, pantropical).

Although all genetic studies have shown *Acacia* s.s. to be monophyletic, the morphological and other characters that separate this genus from the six genera excised from *Acacia* s.l. have been poorly studied. Nevertheless, 95% of *Acacia* s.s. species possess phyllodes which readily distinguishes them from the bipinnate-leaved species of the other six genera (but there are also 73 bipinnate-leaved species in *Acacia* s.s.; WorldWideWattle 2022). Other attributes that help characterise *Acacia* s.s. include their extraporate pollen grains which possess pseudocolpi on their exine surface (a character otherwise not found elsewhere in Mimoseae, Guinet 1981a), the sequence of their seedling leaf development (Vassal 1972) and some biochemical and biological characters summarised by Pedley (1986). However, with the possible exception of the pollen, these attributes have not been comprehensively surveyed across *Acacia* s.l.

The infrageneric classification adopted for *Acacia* today is that of Pedley (1978) in which the species are arranged in seven sections, Acaciasect.Acacia (= sect. Phyllodineae DC.), Acaciasect.Alatae (Benth.) Pedley, Acaciasect.Botrycephalae (Benth.) Taub., Acaciasect.Juliflorae (Benth.) C. Moore & Betche, Acaciasect.Lycopodiifoliae Pedley, Acaciasect.Plurinerves (Benth.) C. Moore & Betche, and Acaciasect.Pulchellae (Benth.) Taub. Although this is a convenient scheme for grouping species of this large genus, it is artificial and does not define monophyletic groups (Murphy 2008). Molecular phylogenetic studies have provided useful insights into evolutionary questions and informal names have been proposed for some of the major clades, but these studies have been hampered by lack of resolution for some relationships and incomplete sampling of species to date (e.g., Murphy et al. 2010; Mishler et al. 2014).

#### Taxonomic references.

Andrew et al. (2003); Bentham (1875); Guinet (1969, 1981a); Hnatiuk and Maslin (1988); Maslin (2001, 2011, 2015); Maslin and McDonald (2004); Maslin and Reid (2012); Maslin and Thomson (1992); Maslin et al. (2019b); McDonald et al. (2001); Miller and Bayer (2000, 2001); Miller and Seigler (2012); Miller et al. (2002); Mishler et al. (2014); Moran et al. (1992); Moore et al. (2010); Murphy (2008); Murphy et al. (2003, 2010); Pedley (1975, 1978, 1986); Ross (1979); Thiele et al. (2011); Vassal (1972).

## ﻿﻿32. *Cedrelinga*

Felipe da Silva Santos^2^, Carolina Lima Ribeiro^2^, Luciano Paganucci de Queiroz^2^

Citation: Santos FS, Ribeiro CL, Queiroz LP (2024) 32. Cedrelinga. In: Bruneau A, Queiroz LP, Ringelberg JJ (Eds) Advances in Legume Systematics 14. Classification of Caesalpinioideae. Part 2: Higher-level classification. PhytoKeys 240: 430–433. https://doi.org/10.3897/phytokeys.240.101716

### ﻿ Cedrelinga

Taxon classificationPlantaeFabalesFabaceae

Ducke, Arch. J. Bot. Rio de Janeiro 3: 70. 1922.

**Figure 241.**
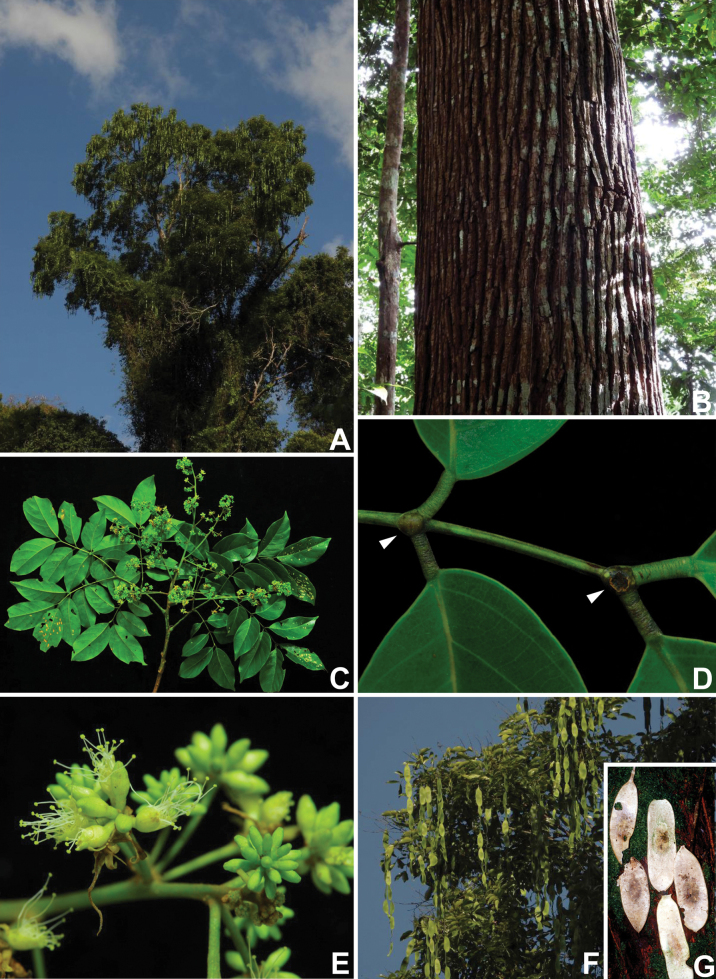
Phylogenetic position of *Cedrelinga* and *Pseudosamanea* in tribe Mimoseae. For description of phylogeny and support values, see Fig. 6 caption (page 63).

**Figure 242.**
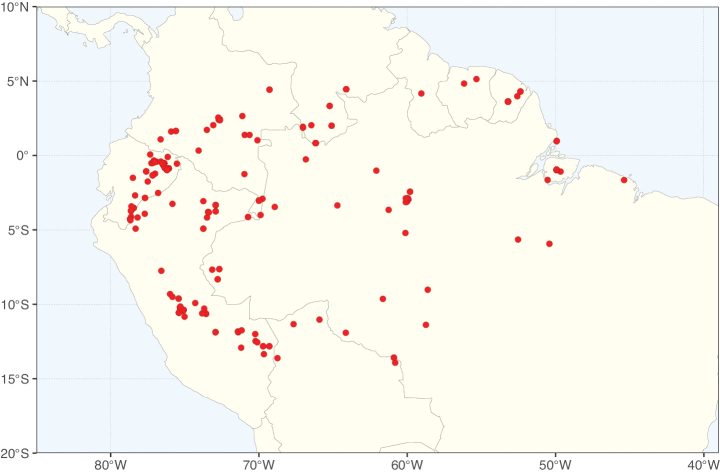
*Cedrelingacateniformis* (Ducke) Ducke, the only species in the genus **A** tree in a natural park environment in Guyana **B** trunk showing the rough and striate bark **C** flowering branch with foliage and inflorescences **D** extrafloral nectaries at the point of insertion on the leaflets (arrowheads) **E** flowers grouped into hemispherical capitula **F** loment, showing the articles rotated between them **G** separate mature articles. Photo credits **A, B, F** © S Sébastien Sant / Parc amazonien de Guyane (Cedrelingacateniformis (Ducke) Ducke, 1922-Description, detailed sheet (mnhn.fr) **C, D, E** unknown-BIOWEB (Galería Bioweb Ecuador) **G** RB Foster © Field Museum of Natural History - CC BY-NC 4.0 (Cedrelingacateniformis | Fotos de Campo | The Field Museum).

**Figure 243.**
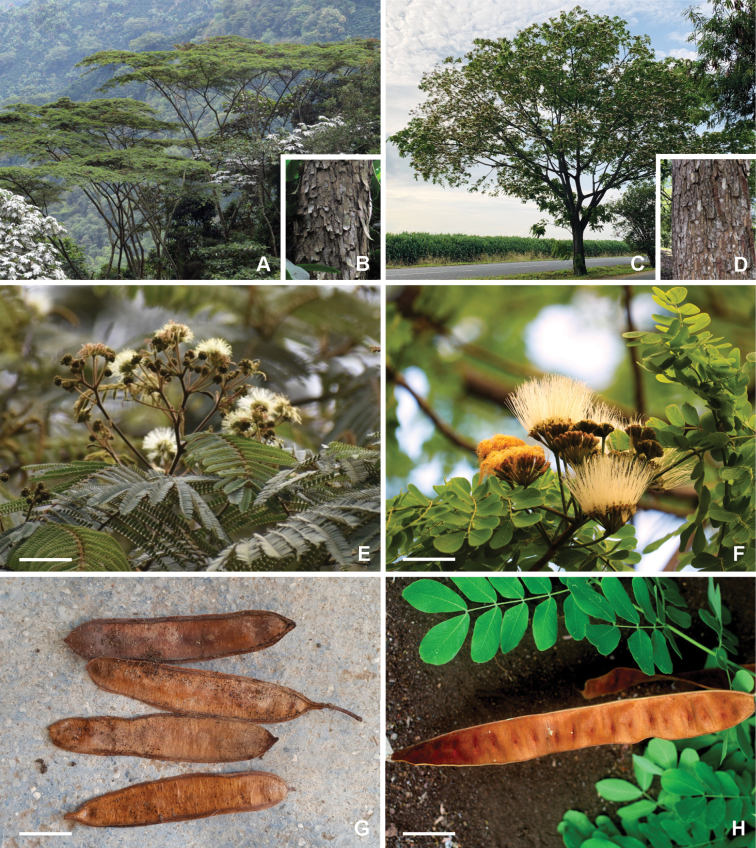
Distribution of *Cedrelinga* based on quality-controlled digitised herbarium records. See Suppl. material 1 for the source of occurrence data.

Figs 241 , 242 , 243

#### Type.

*Cedrelingacateniformis* (Ducke) Ducke [≡ *Piptadeniacateniformis* Ducke]

Phylogenomic studies support *Cedrelinga* Ducke as related to other genera of the Ingoid clade but its phylogenetic position is not fully resolved as it appears in a mostly isolated position in a polytomy including the genus *Pseudosamanea* Harms and the Samanea, Jupunba, Inga and Albizia clades (Koenen et al. 2020a; Ringelberg et al. 2022; Fig. 241).

#### Description.

Unarmed emergent trees, 25–60 (65) m, to 2.5 m diameter, the trunk reddish with a rugous and striated bark, buttressed at base. **Stipules** absent. **Leaves** bipinnate, foliar glands at or near the insertion of each pair of pinnae and on the pinnae between the insertion of leaflets; pinnae 2–4 pairs, opposite; leaflets 3–4 pairs per pinna, opposite, elliptical with acute or acuminate apex, pinnately veined, glabrous. **Inflorescences** 8–20 flowered hemispherical capitula, grouped in terminal panicles or pseudoracemes; bracts ovate or obovate-spatulate, puberulent, persistent. **Flowers** 5-merous, greenish-white, glabrous except for ciliate calyx-teeth, and sometimes papillate on corolla lobe tips; calyx gamosepalous, short-campanulate; corolla gamopetalous with ovate lobes; stamens 24–30, greenish-white, united to the middle; intrastaminal disc absent; pollen in 16-celled acalymmate polyads, each pollen grain 6-porate with a finely reticulate surface; ovary shortly stipitate, ellipsoid, abruptly conic at apex, style a little longer than stamens. **Fruits** pendulous, indehiscent, lomentiform, linear but with deeply constricted margins forming 2–5 (6) oblong-elliptic, plano-compressed and one-seeded articles, twisted through ±90° at each isthmus, but plane and straight between them; valves brownish, glabrous, sinuously venulose, separating through the transverse fission of the isthmus and dispersed individually. **Seeds** disciform.

#### Chromosome number.

Unknown.

#### Included species and geographic distribution.

Monospecific (*C.cateniformis*), across equatorial latitudes of the Amazon delta region, reaching the main tributary rivers in Bolivia, Brazil, Colombia, Ecuador, French Guiana, Guyana, Suriname, Venezuela and near the Andes in Peru (Barneby and Grimes 1996, Lewis and Rico Arce 2005; Fig. 243).

#### Ecology.

Wet or seasonally dry primary forests, especially along streams.

#### Etymology.

The name *Cedrelinga* refers to the similarity of its trunk with that of *Cedrelafissilis* Vell. and *C.odorata* L. (‘cedro’ in Brazilian Portuguese, family Meliaceae) and the Tupi ‘ingá’, which is an indigenous name for several South American species of rainforest mimosoid legume genera.

#### Human uses.

*Cedrelingacateniformis* can be used as timber, in construction, carpentry, as paper and cellulose, and in agroforestry, due to its rapid seedling development and association with nitrogen-fixing bacteria (Loureiro et al. 1979; Baluarte and Alvarez 2015). It is reported as a medicine but without information on which part of the plant is used (Lewis and Rico Arce 2005). It is known by the vernacular names “chuncho” (Ecuador), “cedrorana” (Brazil, literally fake cedar in Tupi language), “iacaiacá” (Brazil), “cachicana”, “mure”, “guaura” (Venezuela), “don-ceder” (Suriname), “huayracaspi” and “tornillo” (Peru) (Ducke 1949).

#### Notes.

When describing *Cedrelinga*, Ducke (1922) considered it to be related to the broadly circumscribed *Pithecellobium* Mart. *Cedrelinga* was included in tribe Ingeae (Nielsen 1981a), but Barneby and Grimes (1996) remarked that it had no obvious relatives within that tribe. *Cedrelinga* is one of the tallest trees and with the widest trunk diameters among Amazonian arborescent species. Despite being a large tree, it normally has a small crown and during fruiting it can be easily spotted due to the large number of pendulous fruits (usually one fruit per capitulum). The mature fruits are dry when articles break off and are dispersed by the wind. The wood of *C.cateniformis* has a spongy appearance due to the width of its vessels, having low density and when wet it has an unpleasant odour (Ducke 1915, 1922, 1949; Barneby and Grimes 1996).

#### Taxonomic references.

Barneby and Grimes (1996); da Silva et al. (1992); Ducke (1915, 1922, 1949); Lewis and Rico Arce (2005); Nielsen (1981a).

## ﻿﻿33. *Pseudosamanea*

Andrés Fonseca-Cortés^2^

Citation: Fonseca-Cortés A (2024) 33. Pseudosamanea. In: Bruneau A, Queiroz LP, Ringelberg JJ (Eds) Advances in Legume Systematics 14. Classification of Caesalpinioideae. Part 2: Higher-level classification. PhytoKeys 240: 434–436. https://doi.org/10.3897/phytokeys.240.101716

### ﻿﻿ Pseudosamanea

Taxon classificationPlantaeFabalesFabaceae

Harms, Notizbl. Bot. Gart. Berlin-Dahlem 11(101): 54. 1930.

**Figure 244.**
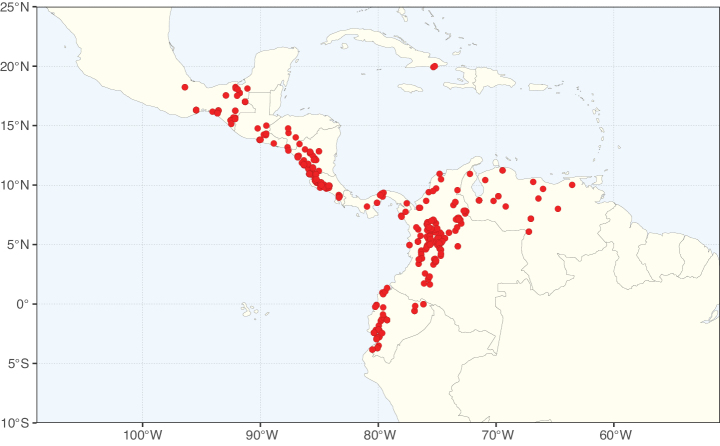
General morphology in *Pseudosamanea* species **A, B, E, G***Pseudosamaneacarbonaria* (Britton) E.J.M. Koenen (left) **A** habit **B** exfoliating bark **E** bipinnate microphyllidious leaves and umbelliform capitula **G** papery oblong laterally compressed pods **C, D, F, H***Pseudosamaneaguachapele* (Kunth) Harms (right) **C** habit **D** exfoliating bark **F** bipinnate macrophyllidious leaves and umbelliform capitula **H** papery oblong laterally compressed fruits. Scale bars: 2 cm (**E**); 4 cm (**F**); 3 cm (**G**); 2.5 cm (**H**). Photo credits **A** Bioexploradores Farallones, iNaturalist (https://www.inaturalist.org/photos/30575576) **B, D, H** A Fonseca-Cortés **C** Juan Manuel de Roux, iNaturalist (https://www.inaturalist.org/photos/112891215) **E** Juan Manuel de Roux, iNaturalist (https://www.inaturalist.org/photos/54043095) **F** Cynthia Tercero, iNaturalist (https://www.inaturalist.org/photos/59667691) **G** Juan Carlos Delgado Madrid, iNaturalist (https://www.inaturalist.org/photos/64538395).

**Figure 245.**
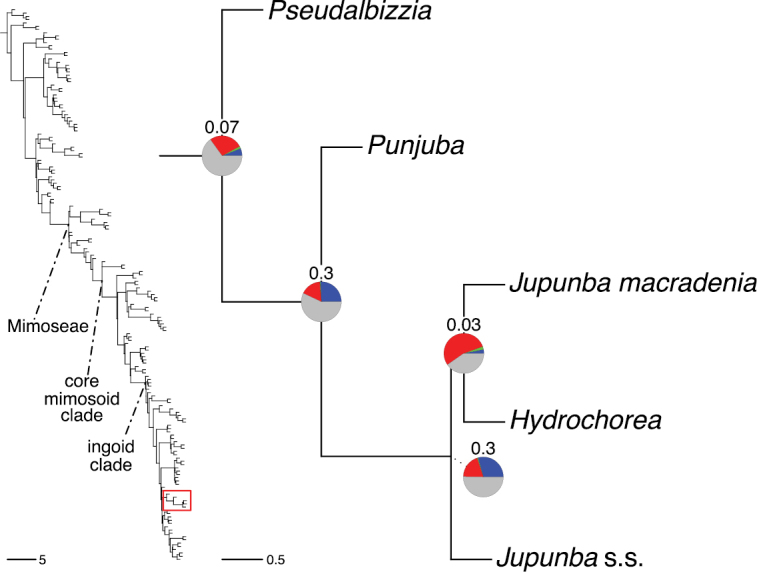
Distribution of *Pseudosamanea* based on quality-controlled digitised herbarium records. See Suppl. material 1 for the source of occurrence data.

Figs 241 , 244 , 245

#### Type.

*Pseudosamaneaguachapele* (Kunth) Harms [≡ *Acaciaguachapele* Kunth]

The phylogenetic position of *Pseudosamanea* is not well resolved in the Mimoseae phylogeny (Koenen et al. 2020a; Ringelberg et al. 2022). The genus is placed in a large, putative hard polytomy in the ingoid clade (Fig. 241), but its exact position is difficult to ascertain, and differs across phylogenetic analyses (Koenen et al. 2020a). *Pseudosamanea* is therefore not treated as part of any clade within Mimoseae and is here recognised as a single genus lineage.

#### Description.

Unarmed trees 6–30 m, with wide spreading crown; bark fissured and exfoliating in irregular rectangular layers. **Stipules** triangular to lanceolate, sericeous, caducous. **Leaves** bipinnate, extrafloral nectaries usually present near the petiole base, another between the terminal pair of pinnae and less frequently along the rachides; pinnae 3–13 pairs, paraphyllidia present, caducous; leaflets 5–30 pairs, obovate, elliptic or lanceolate. **Inflorescence units** heteromorphic umbelliform capitula, 2–6-fascicled in leaf axil, the terminal flower sessile and stouter. **Flowers** 5 (6–7)-merous; calyx gamosepalous, ferruginous villose; corolla gamopetalous, campanulate, lobes triangular, canescent to yellowish villose; stamens numerous, the filaments basally united into a tube; pollen in 24–32-celled polyads; ovary ovoid. **Fruits** indehiscent or dehiscent through one margin, oblong, laterally compressed, valves papery, puberulent, margins slightly thicker. **Seeds** ovoid, laterally compressed, pleurogram present (Fig. 244).

#### Chromosome number.

2*n* = 26 [*P.carbonaria* (Britton) E.J.M. Koenen and *P.guachapele*] (Rico Arce 1992).

#### Included species and geographic distribution.

Three species known to date: *P.carbonaria*, *P.cubana* (Britton & P. Wilson) Barneby & J.W. Grimes and *P.guachapele*. *Pseudosamanea* species occur from the south of Mexico to the north of Peru, with one species in Cuba (Fig. 245; Barneby and Grimes 1996). The native range of *P.carbonaria* is not known with certainty, but it is presumed to be native from Colombia, Panama and Venezuela from 700–1800 m elevation and introduced in Indonesia (Koenen 2022), Central America, the Greater Antilles, Peru and Brazil (Barneby and Grimes 1996; under *Albiziacarbonaria* Britton). *Pseudosamaneacubana* is endemic to the south coast of Cuba to 50 m elevation (Barneby and Grimes 1996). *Pseudosamaneaguachapele* is present from the south of Mexico to the north of Peru between 0–1000 m and introduced in Brazil and Cameroon and probably elsewhere (Barneby and Grimes 1996).

#### Ecology.

*Pseudosamaneacarbonaria* grows in dry and humid forests (Barneby and Grimes 1996), usually near water courses, its seeds are usually dispersed with the fruit by barochory and hydrochory (pers. obs.). *Pseudosamaneacubana* grows in riverine forests and savannas with palms (Barneby and Grimes 1996). *Pseudosamaneaguachapele* grows in dry forests (Barneby and Grimes 1996). Little is known about the interactions with ants, floral visitors and pollinators.

#### Etymology.

From the Greek *pseudo* (= false) and *Samanea*, due to the similarity to that genus (Lewis and Rico Arce 2005).

#### Human uses.

The three species can be used as timber. *Pseudosamaneacarbonaria* is cultivated for shading coffee crops, *P.guachapele* is used as living fencing in Colombia, and both are used as ornamentals (Barneby and Grimes 1996).

#### Notes.

As defined by Barneby and Grimes (1996), *Pseudosamanea* included two species of macrophyllidious trees (Fig. 244F), *P.cubana* and *P.guachapele*. The phylogenomic analyses of Ringelberg et al. (2022) showed *Albiziacarbonaria* to be sister to *Pseudosamanea*, rather than grouping with species of AlbiziasectionArthrosamanea (Britton & Rose) Barneby & J.W. Grimes (now the genus *Pseudalbizzia* Britton & Rose; Peraza et al. 2022), where it had previously been classified. *Albiziacarbonaria* was combined in *Pseudosamanea* based on molecular and morphological evidence (Koenen 2022a). Although *P.carbonaria* is a microphyllidious tree (Fig. 244E), it shares with the other two species of *Pseudosamanea* exfoliating bark (Fig. 244B, D), heteromorphic umbelliform inflorescences (Fig. 244E, F) in which the central flower is bigger and sessile, and oblong laterally compressed papery legumes (Fig. 244G, H) (Koenen 2022a). Pollen also supports this recircumscription, *P.carbonaria* having pollen with 32-celled polyads as in *P.guachapele* (Koenen 2022a).

#### Taxonomic references.

Barneby and Grimes (1996); Koenen (2022a); Lewis and Rico Arce (2005).

## ﻿﻿34. Jupunba clade

João Iganci^20,25^, Marcos Vinícius Batista Soares^20^, Marli Pires Morim^8^

Citation: Iganci J, Soares MVB, Morim MP (2024) 34. Jupunba clade. In: Bruneau A, Queiroz LP, Ringelberg JJ (Eds) Advances in Legume Systematics 14. Classification of Caesalpinioideae. Part 2: Higher-level classification. PhytoKeys 240: 437–444. https://doi.org/10.3897/phytokeys.240.101716

**Jupunba clade**

Figs 246–251

**Included genera (4).***Hydrochorea* Barneby & J.W. Grimes (10 species), *Jupunba* Britton & Rose (37), *Pseudalbizzia* Britton & Rose (17), *Punjuba* Britton & Rose (5).

**Description.** Trees, treelets and shrubs, unarmed. **Stipules** puberulent to glabrous, deltate, elliptic, lanceolate, linear, ligulate, ovate, subulate, triangular or triangular-ovate, veinless or faintly 3-veined, falling early to tardily, or absent. **Leaves** bipinnate, pinnae 1–16 (19) pairs, leaflets 1–52 (63) pairs, alternate, variable in size and shape, the first pair of leaflets often reduced to paraphyllidia in *Pseudalbizzia*; extrafloral nectaries sessile or rarely stipitate, between the pairs of pinnae, or close below the proximal pair of pinna-pulvines, campanulate to cupular, orbicular, patelliform, elliptic, vertically elongate, verruciform or obsolete. **Inflorescences** congested or lax racemes, capitate, corymbose-umbellate or spiciform. **Flowers** homomorphic or heteromorphic, pedicellate or sessile; sepals 5–8, united; petals 5–8, joined into a gamopetalous corolla; stamens numerous, filaments fused into a tube, stemonozone present, anthers rimose; pollen 16, 18, 28-celled polyads; ovary glabrous or pubescent. **Fruit** a dehiscent legume, lomentiform or with the endocarp breaking into 1-seeded envelopes. **Seeds** disciform, oblong, elliptic, ovate, obovate, orbicular or flattened, translucent, pleurogram present or absent.

**Distribution.** In the Neotropics from Argentina to Colombia in South America, Central America, and West Indies, with two species of *Hydrochorea* from Democratic Republic of the Congo and Senegal, in Africa.

**Figure 246.**
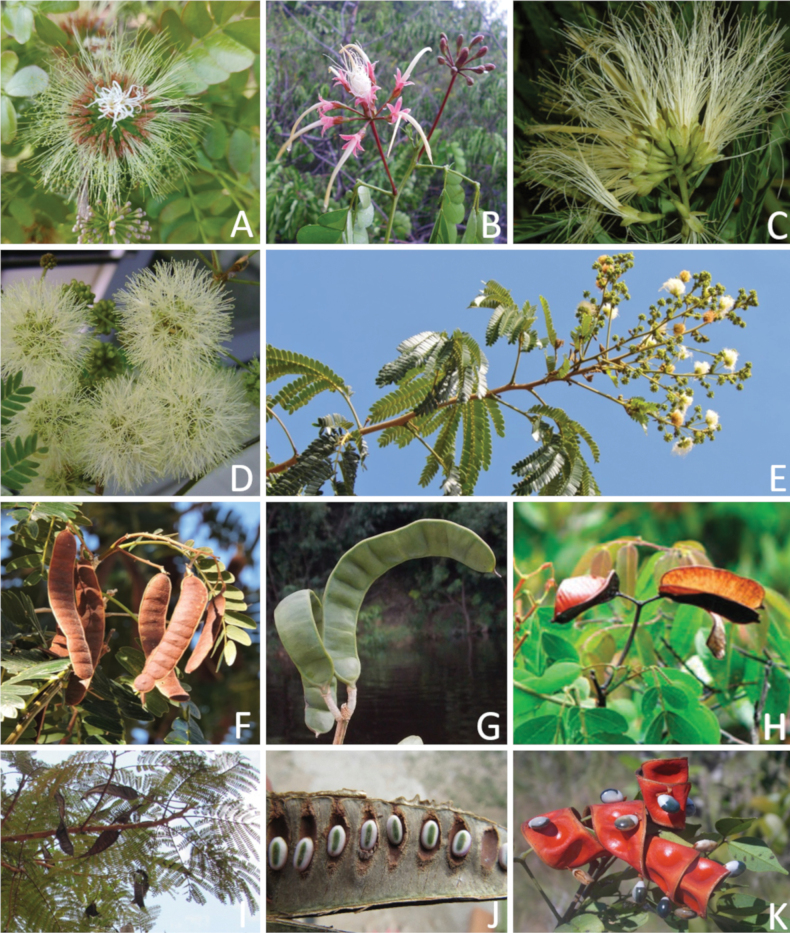
Generic relationships in the Jupunba clade (tribe Mimoseae). For description of phylogeny and support values, see Fig. 6 caption (page 63).

**Notes.** Although fruit morphology has been used in the past to group taxa in the former tribe Ingeae sensu Bentham (1865), phylogenetic analyses indicate rampant homoplasy in fruit characters (Koenen et al. 2020a; Soares et al. 2021). The presence of dimorphic flowers in most species across the Jupunba clade may be a better diagnostic feature of this clade. *Jupunba* and *Punjuba* were each segregated from the polyphyletic *Abarema* Pittier, because the type species of *Abarema*, *A.cochliacarpos* (Gomes) Barneby & J.W. Grimes, is nested within the Inga clade (Iganci et al. 2016; Guerra et al. 2023). *Jupunba* is composed mostly of the former *Abarema* species (sensu Barneby and Grimes 1996), which occur from the Atlantic Forest in Eastern Brazil to the Amazon, Central America and West Indies (Iganci et al. 2016). The genus encompasses species with racemose to capitate inflorescences, and seeds frequently with a pleurogram. Species bearing spiciform inflorescences and seeds lacking pleurogram, with a narrower distribution along the Andean valleys and Central America, are recognised under *Punjuba*. Both *Jupunba* and *Punjuba* have curved legumes with red endocarp, and translucent seeds with blue embryo, adapted to endozoochoric dispersal, while the remaining genera in the clade have adapted to different dispersal strategies.

**Clade based definition.** The most inclusive crown clade containing *Punjubaracemiflora* (Donn. Sm.) Britton & Rose and *Pseudalbizziaberteroana* (Balb. ex DC.) Britton & Rose, but not *Pseudosamaneaguachapele* (Kunth) Harms, *Samaneasaman* (Jacq.) Merr. or *Albiziaretusa* Benth. (Fig. 246).

*Hydrochorea* and *Balizia* Barneby & J.W. Grimes were recognised by Barneby and Grimes (1996) as distinct but closely related genera adapted to riverine habitats across Amazonia in South America. Phylogenomic analyses by Koenen et al. (2020a) showed that *Balizia* is nested within *Hydrochorea*, which has been newly circumscribed (with 10 species) to embrace the Neotropical *Balizia* species plus two African species formerly recognised under *Albizia* Durazz. (Soares MVB et al. 2022). The latter two species are morphologically similar to *Hydrochorea*, which also has water-dispersed seeds.

### ﻿ Pseudalbizzia

Taxon classificationPlantaeFabalesFabaceae

Britton & Rose, N. Am. Fl. 23: 48. 1928.

Figs 247 , 248

Arthrosamanea Britton & Rose, Ann. New York Acad. Sci. 35: 128. 1936. Type: Arthrosamaneapistaciifolia (Willd.) Britton & Rose ex Britton & Killip [≡ Mimosapistaciifolia Willd. (≡ Pseudalbizziapistaciifolia (Willd.) E.J.M. Koenen & Duno)]
Albizia sect. Arthrosamanea (Britton & Rose) Barneby & J.W. Grimes, Mem. New York Bot. Gard. 74(1): 206. 1996. Type: Albiziapistaciifolia (Willd.) Barneby & J.W. Grimes [≡ Mimosapistaciifolia Willd. (≡ Pseudalbizziapistaciifolia (Willd.) E.J.M. Koenen & Duno)]

#### Type.

*Pseudalbizziaberteroana* (Balb. ex DC.) Britton & Rose [≡ *Acaciaberteroana* Balb. ex DC.]

#### Description.

Trees or rarely treelets, unarmed. **Stipules** puberulent to glabrous, deltate, narrowly triangular, triangular-ovate, narrowly ovate, or narrowly lanceolate, veinless or faintly 3-veined, falling early to tardily. **Leaves** bipinnate, extrafloral nectaries sessile between the first pair of pinnae, near or well below mid-petiole, round, elliptic or vertically elongate, cupuliform to obsolete; pinnae (1) 2–15 (19) pairs; leaflets (2) 16–52 (63) pairs, alternate, variable in size and shape, the first pair of leaflets often reduced to paraphyllidia. **Inflorescences** capitate or corymbose-umbellate. **Flowers** heteromorphic, short-pedicellate or sessile; calyx gamosepalous; corolla gamopetalous, petals 5 (6); stamens numerous, filaments fused into a tube, stemonozone present, anthers rimose; pollen in 16-celled polyads; ovary glabrous or pubescent. **Fruit** lomentiform or the endocarp fragmented into 1-seeded envelopes, the valves papery, coriaceous, or glossy ligneous, straight or nearly so, decurved or plano-compressed, tardily and inertly dehiscent through both sutures or indehiscent. **Seeds** disciform, oblong-ellipsoid, elliptic, flattened, translucent, pleurogram present.

#### Chromosome number.

2*n* = 26 (Rico Arce 1992; Santos et al. 2012).

#### Included species and geographic distribution.

Seventeen species, in the Neotropics, from Argentina to Colombia in South America, and from north-west Mexico to Panama in North America, extending weakly into the West Indies (Fig. 248).

**Figure 247.**
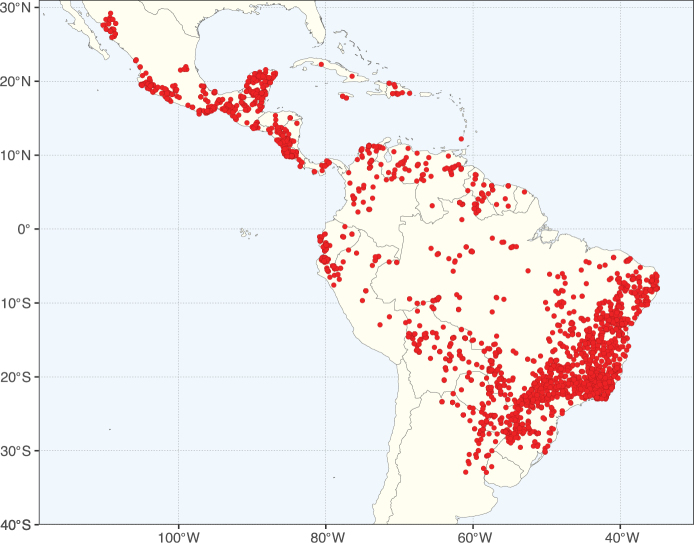
Jupunba clade flowers (**A–E**) and fruits (**F–K**) **A***Hydrochoreacorymbosa* (Rich.) Barneby & J.W. Grimes **B***Hydrochoreauaupensis* M.P. Morim, Iganci & E.J.M. Koenen **C***Jupunbalangsdorffii* (Benth.) M.V.B. Soares, M.P. Morim & Iganci **D***Pseudalbizzianiopoides* (Spruce ex Benth.) E.J.M. Koenen & Duno **E***Pseudalbizziapolycephala* (Benth.) E.J.M. Koenen & Duno **F***Pseudalbizziainundata* (Mart.) E.J.M. Koenen & Duno **G***Hydrochoreacorymbosa***H***Hydrochoreauaupensis***I, J***Hydrochoreapedicellaris* (DC.) M.V.B. Soares, Iganci & M.P. Morim **K***Jupunbacampestris* (Benth.) M.V.B. Soares, M.P. Morim & Iganci. Photo credits **A, G, I–K** MVB Soares **B, C, H** J Iganci **D–F** RT Queiroz https://rubens-plantasdobrasil.blogspot.com/.

**Figure 248.**
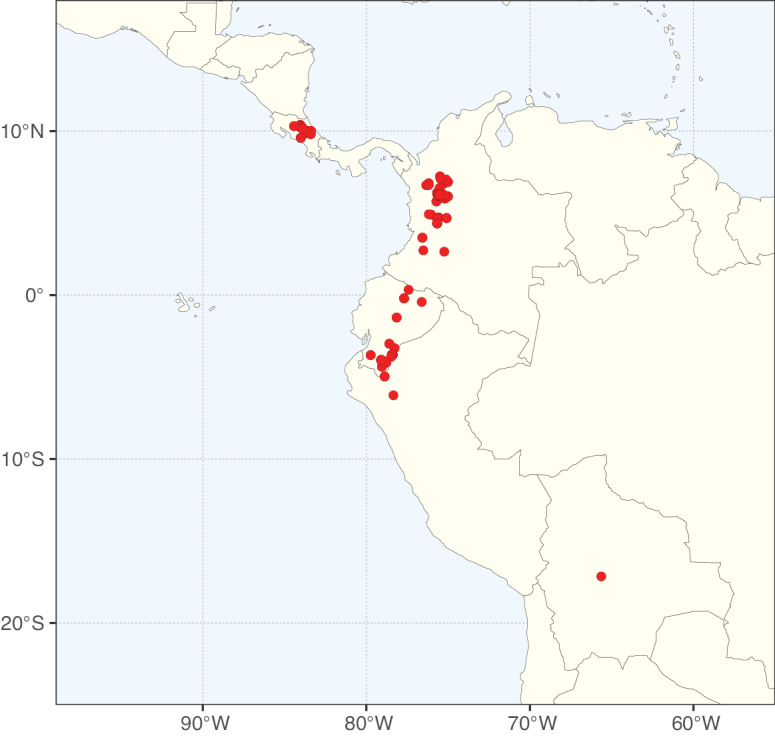
Distribution of *Pseudalbizzia* based on quality-controlled digitised herbarium records. See Suppl. material 1 for the source of occurrence data.

#### Ecology.

Humid, semi-deciduous, seasonally inundated, and seasonally dry tropical and extratropical forests and woodlands.

#### Etymology.

In reference to *Albizia* Durazz.

#### Human uses.

The wood of *P.polycephala* (Benth.) E.J.M. Koenen & Duno (‘farinha-seca’ in Brazil), is used for laminate wood floors and for internal use in civil construction (Carvalho 2006).

#### Notes.

The name *Pseudalbizzia* was recently resurrected mostly to accommodate species of Albiziasect.Arthrosamanea (Peraza et al. 2022). Barneby and Grimes (1996) had noted the determinate branches lacking sylleptic buds giving rise to vegetative branches as a distinctive character to recognise the 19 species under A.sect.Arthrosamanea, as well as remarking on the resemblance of the lomentiform pods of a subset of species of A.sect.Arthrosamanea to those of *Hydrochorea*. Phylogenomic (Koenen et al. 2020a; Ringelberg et al. 2022; Peraza et al. 2022) and phylogenetic analyses based on nuclear ribosomal sequences (Peraza et al. 2022) confirmed the non-monophyly of *Albizia* and showed that all species sampled from A.sect.Arthrosamanea formed a clade, sister to a clade composed of the remaining genera of the Jupunba clade (Fig. 246). These analyses also revealed that lomentiform fruits are independently derived from indehiscent septate fruits in *Pseudalbizzia* and *Hydrochorea*, and that morphological adaptation to hydrochory has occurred several times independently across the ingoid clade.

A new phylogenetically-based infrageneric classification, comprising five sections, was presented by Peraza et al. (2022) to account for the non-monophyly of the series of A.sect.Arthrosamanea of Barneby and Grimes (1996).

#### Taxonomic references.

Barneby and Grimes (1996); Peraza et al. (2022).

### ﻿ Punjuba

Taxon classificationPlantaeFabalesFabaceae

Britton & Rose, N. Amer. Fl. 23: 28. 1928.

Figs 247 , 249

#### Type.

*Punjubaracemiflora* (Donn. Sm.) Britton & Rose [≡ *Pithecellobiumracemiflorum* Donn. Sm.]

#### Description.

Shrubs and trees, unarmed. **Stipules** absent. **Leaves** bipinnate, extrafloral nectaries sessile, between the first pair of pinnae or along the leaf rachis, orbicular, patelliform, cupuliform; pinnae 1–3 pairs; leaflets 2–7 pairs, alternate, variable in size and shape. **Inflorescences** racemes or spiciform racemes. **Flowers** homomorphic, pedicellate or sessile; sepals 5, united; corolla gamopetalous, petals 5; stamens numerous, filaments fused into a tube, stemonozone present, anthers rimose; pollen in 18-grained polyads; ovary glabrous. **Fruit** a legume dehiscing through both margins, the valves chartaceous, curved to spiral, generally with a red endocarp, epicarp glabrous. **Seeds** obovate, orbicular or oblong, translucent, pleurogram absent.

#### Chromosome number.

2*n* = 26 (*P.racemiflora*) (Bawa 1973).

#### Included species and geographic distribution.

**Figure 249.**
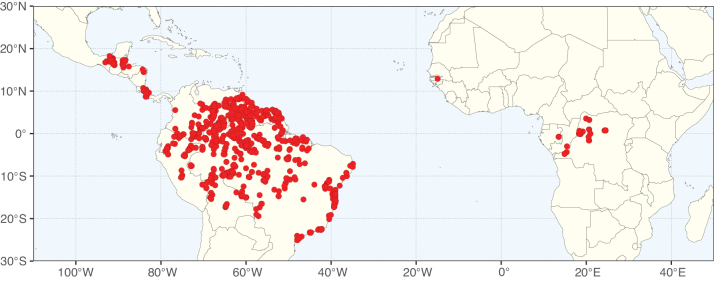
Distribution of *Punjuba* based on quality-controlled digitised herbarium records. See Suppl. material 1 for the source of occurrence data.

Five species in the Andean valleys of Bolivia, Colombia, Ecuador, and Peru, with *P.racemiflora* occurring in Costa Rica and Panama (Fig. 249).

#### Ecology.

Mainly in mountain and Andean forests and secondary rainforests.

#### Etymology.

An anagram of *Jupunba* Britton & Rose.

#### Human uses.

Firewood.

#### Notes.

*Punjuba* was described by Britton and Rose (1928), placed under synonymy in *Abarema* by Barneby and Grimes (1996), but was recently reinstated based on morphological and phylogenetic evidence (Soares et al. 2021). Although similar to *Jupunba* in fruit morphology, seeds lacking pleurogram (vs. pleurogram present), inflorescences always in spiciform racemes (vs. inflorescences in congested, lax or spiciform racemes), and leaflets with arched veins (vs. leaflets with straight veins) differentiate the two genera.

#### Taxonomic references.

Barneby and Grimes (1996); Britton and Rose (1928); Iganci et al. (2016); Soares et al. (2021).

### ﻿ Hydrochorea

Taxon classificationPlantaeFabalesFabaceae

Barneby & J.W. Grimes, Mem. New York Bot. Gard. 74(1): 23. 1996.

Figs 247 , 250

Balizia Barneby & J.W. Grimes, Mem. New York Bot. Gard. 74(1): 34–35. 1996. Type: Baliziapedicellaris (DC.) Barneby & J.W. Grimes [≡ Ingapedicellaris DC. (≡ Hydrochoreapedicellaris (DC.) M.V.B. Soares, Iganci & M.P. Morim)]

#### Type.

*Hydrochoreacorymbosa* (Rich.) Barneby & J.W. Grimes [≡ *Mimosacorymbosa* Rich.]

#### Description.

Shrubs and trees, unarmed. **Stipules** linear, linear-lanceolate, ligulate or subulate, persistent or caducous**. Leaves** bipinnate, extrafloral nectaries sessile between the first pair of pinnae or along the leaf rachis, orbicular, patelliform or cupuliform; pinnae 1–15 pairs; leaflets 2–33 pairs, alternate, variable in size and shape. **Inflorescences** corymbose-umbellate. **Flowers** heteromorphic, the peripheral flowers pedicellate, the terminal flower sessile; calyx gamosepalous, 5–8 toothed; corolla gamopetalous, 5–8 toothed; stamens (10) 12–30 (36), filaments fused into a tube, stemonozone present, anthers rimose; pollen in 16 or 28-celled polyads, plano-compressed disc-shaped; ovary pubescent or glabrous. **Fruits** straight or recurved, either lomentiform, the seeds released in one-seeded articles, or woody and indehiscent, or follicular, or crypto-lomentiform with follicular dehiscence. **Seeds** ovate to oblong, with pleurogram.

#### Chromosome number.

Unknown.

#### Included species and geographic distribution.

**Figure 250.**
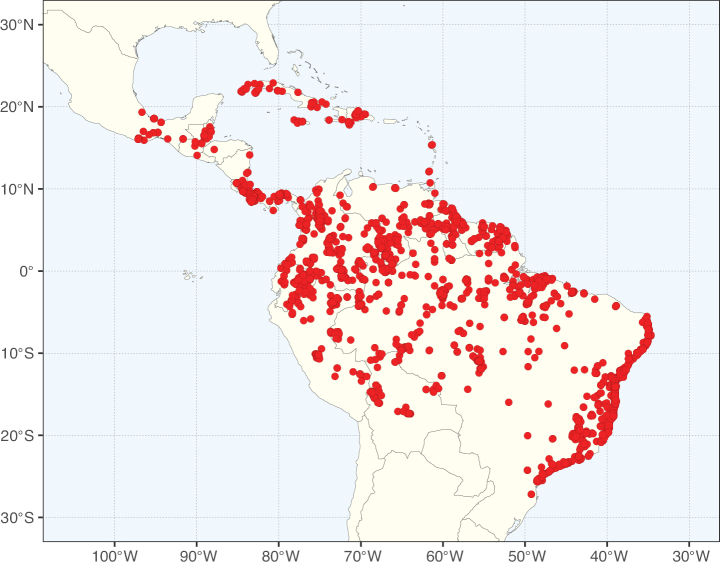
Distribution of *Hydrochorea* based on quality-controlled digitised herbarium records. See Suppl. material 1 for the source of occurrence data.

Ten species, from Brazil to Colombia in South America, Central America, Democratic Republic of the Congo and Senegal (Fig. 250).

#### Ecology.

Riparian habitats, inundated and non-inundated wet tropical forests, swamp forests, seasonally inundated forests, riverbanks, mangrove swamps, and gallery forests.

#### Etymology.

From Greek, *hydro* (= water) and *chorein* (= to travel), in allusion to water dispersed propagules.

#### Human uses.

Firewood.

#### Notes.

The genus *Hydrochorea* was recently revised based on phylogenomic analyses (Soares MVB et al. 2022) that confirmed *Balizia* as paraphyletic in relation to *Hydrochorea*, along with two African species formerly placed in *Albizia*. Soares MVB et al. (2022) suggested that rapid initial divergence between *Hydrochorea* and the closely related *Jupunba* led to extensive incomplete lineage sorting and poor phylogenetic resolution between the two genera.

#### Taxonomic references.

Barneby and Grimes (1996); Soares (2015); Soares MVB et al. (2022).

### ﻿ Jupunba

Taxon classificationPlantaeFabalesFabaceae

Britton & Rose, N. Amer. Fl. 23: 24. 1928.

Figs 247 , 251

Klugiodendron Britton & Killip, Ann. New York Acad. Sci. 35(3): 125. 1936. Type: Klugiodendronlaetum (Benth.) Britton & Killip [≡ Pithecellobiumlaetum Benth. (≡ Jupunbalaeta (Benth.) M.V.B. Soares, M.P. Morim & Iganci)]

#### Type.

*Jupunbajupunba* (Willd.) Britton & Rose [≡ *Acaciajupunba* Willd. (= *Jupunbatrapezifolia* (Vahl) Moldenke)]

#### Description.

Trees, treelets and shrubs, unarmed. **Stipules** commonly small, narrow, lanceolate, linear, elliptic, caducous. **Leaves** bipinnate, petiolar nectaries sessile or stipitate (rarely) between the pairs of pinnae, or close below the proximal pair of pinna-pulvinules, campanulate to cupular, patelliform, or verruciform; pinnae 12–16 (19) pairs; leaflets 1–40 pairs, alternate, variable in size and shape. **Inflorescences** congested or lax racemes, rarely capitate or spiciform racemes. **Flowers** homomorphic or heteromorphic, pedicellate or sessile; calyx gamosepalous, 5-merous; corolla gamopetalous, 5-merous; stamens numerous, filaments fused into a tube, stemonozone present, anthers rimose; pollen in acalymmate large polyads containing 16 pollen grains, circular or elliptic, suboblate, sexine rugulate, as thick as nexine; ovary glabrous or pubescent. **Fruit** a legume, the valves chartaceous, curved to spiral, generally with a red endocarp, epicarp glabrous. **Seeds** obovate, orbicular, or oblong, translucent, pleurogram generally present, rarely absent.

#### Chromosome number.

2*n* = 26, 30 (Santos et al. 2012).

#### Included species and geographic distribution.

**Figure 251.**
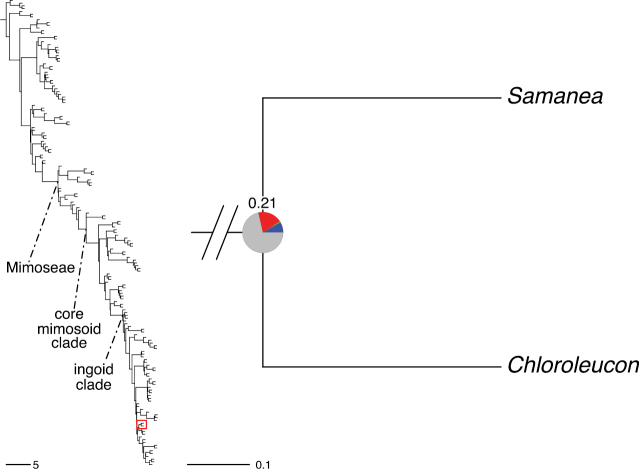
Distribution of *Jupunba* based on quality-controlled digitised herbarium records. See Suppl. material 1 for the source of occurrence data.

Thirty-seven species in South America, from Brazil to Colombia, in Central America and the West Indies (Fig. 251).

#### Ecology.

Riparian habitats, inundated and non-inundated wet tropical forests especially on the Amazon basin.

#### Etymology.

Sieber collected the type specimen of *Acaciajupunba* and recorded the name ‘jupunba’ on the sheet, probably in reference to a vernacular name.

#### Human uses.

Animal feed, firewood, folk medicine, soap making (Fern 2023).

#### Notes.

*Jupunba* is the largest genus in the Jupunba clade, and is mostly composed of species previously described under *Pithecellobium*, and later transferred to *Abarema*. The genus is closely related to *Hydrochorea* but is distinguished by its fruits with reddish endocarp and seeds bicoloured with translucent testa, even though those are apparently plesiomorphic characters (Peraza et al. 2022). *Jupunba* is still being studied to better understand its relationship with *Hydrochorea*, since *J.macradenia* (Pittier) M.V.B. Soares, M.P. Morim & Iganci was resolved as sister to *Hydrochorea* in Ringelberg et al. (2022) (Fig. 246), whereas Soares et al. (2021) recovered the same species and accession (*Lourteig 3021*) nested within *Jupunba* (Soares MVB et al. 2022).

#### Taxonomic references.

Barneby and Grimes (1996); Britton and Rose (1928); Iganci and Morim (2012); Iganci et al. (2016); Soares et al. (2021); Soares MVB et al. (2022).

## ﻿﻿35. Samanea clade

Gwilym P. Lewis^10^

Citation: Lewis GP (2024) 35. Samanea clade. In: Bruneau A, Queiroz LP, Ringelberg JJ (Eds) Advances in Legume Systematics 14. Classification of Caesalpinioideae. Part 2: Higher-level classification. PhytoKeys 240: 445–450. https://doi.org/10.3897/phytokeys.240.101716

**Samanea clade**

Figs 252–255

**Included genera** (2): *Chloroleucon* (Benth.) Britton & Rose (10 species), *Samanea* (Benth.) Merr. (3).

**Description.** Unarmed (*Samanea*) or usually armed with axillary spines (*Chloroleucon*) trees or shrubs. **Stipules** often lanceolate, caducous or lacking. **Leaves** bipinnate, extrafloral nectaries present. **Inflorescences** umbelliform capitula or racemose spikes, in *Chloroleucon* emerging from a cone of imbricate perules. **Flowers** in each inflorescence (occasionally) homomorphic or more commonly dimorphic with the terminal (central) flower modified and stouter, androecium with 10–36 (52) whitish, greenish, pink, or reddish stamens; pollen in polyads of 32 (*Samanea*) or 16–32 grains (*Chloroleucon*); ovary sessile or subsessile. **Fruits** variable, indehiscent, or tardily dehiscent, straight, falcate, coiled or randomly twisted, with or without a pulpy mesocarp. **Seeds** with a hard testa and a pleurogram.

**Distribution.** Mostly South America, more diverse in seasonally dry forests, extending northward to Central America, Mexico and the Caribbean.

**Figure 252.**
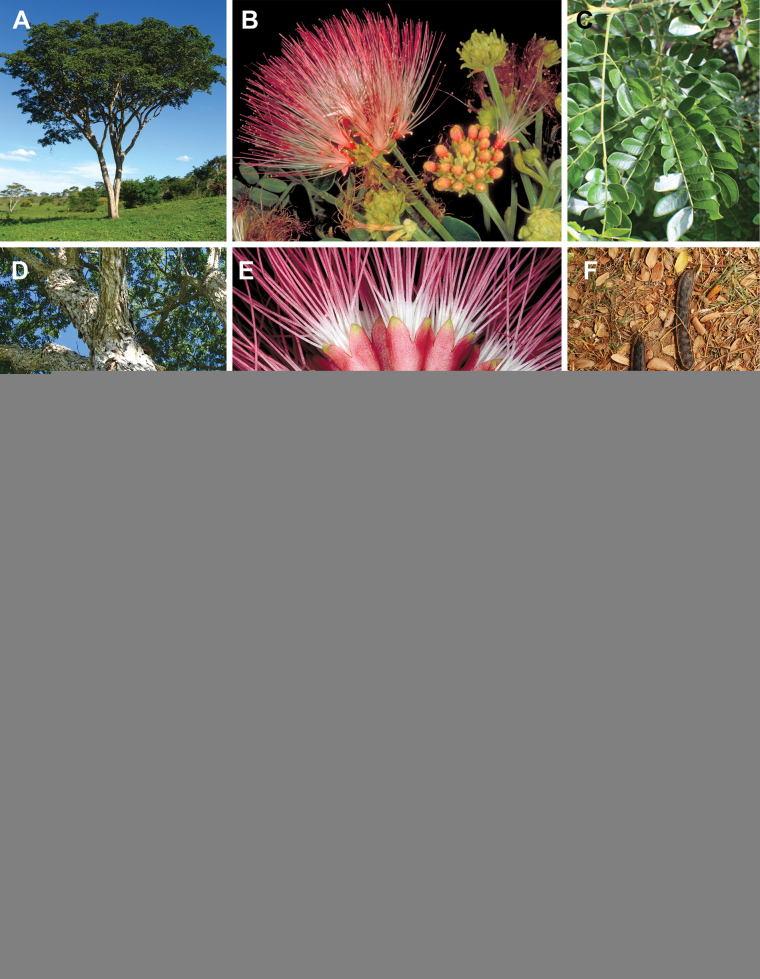
Phylogenetic relationship of the Samanea clade (tribe Mimoseae). For description of phylogeny and support values, see Fig. 6 caption (page 63).

**Clade-based definition.** The most inclusive crown clade containing *Samaneasaman* (Jacq.) Merr. and *Chloroleuconmangense* (Jacq.) Britton & Rose, but not *Jupunbaleucophylla* (Spruce ex Benth.) M.V.B. Soares, M.P. Morim & Iganci, *Albiziaretusa* Benth. or *Boliviadendronbolivianum* (C.E. Hughes & Atahuachi) E.R. Souza & C.E. Hughes (Fig. 252).

**Notes.**Koenen et al. (2020a) and Ringelberg et al. (2022) in their phylogenomic analyses recovered a clade with high support values that groups the genera *Samanea* and *Chloroleucon*. Sparsely sampled phylogenetic analyses based on a few plastid markers had found the two genera to be unresolved in a broad Ingeae clade, but never grouped with other genera (Luckow et al. 2003; Miller et al. 2003). Previously, the two genera had been placed in two separate alliances of old sense tribe Ingeae: the Samanea alliance and the Chloroleucon alliance, respectively (Barneby and Grimes 1996). Despite some reshuffling of constituent genera in the Chloroleucon alliance [for summary, see Brown (2008)], the membership of these two genera to two distinct alliances was retained by Lewis and Rico Arce (2005). Along with *Cedrelinga* and *Pseudosamanea*, the phylogenetic position of the Samanea clade is not well resolved and these unstable positions may be a factor leading to poor resolution in the ingoid clade in general (Koenen et al. 2020a). The sister relationship of *Samanea* and *Chloroleucon* recognised here has not been noted elsewhere and morphologically the two genera are quite distinct.

### ﻿ Samanea

Taxon classificationPlantaeFabalesFabaceae

(Benth.) Merr., J. Wash. Acad. Sci. 6(2): 46. 1916.

Figs 253 , 254

Pithecellobium sect. Samanea Benth., London J. Bot. 3: 197. 1844. Type: Pithecellobiumsaman (Jacq.) Benth. [≡ Mimosasaman Jacq. (≡ Samaneasaman (Jacq.) Merr.)]

#### Type.

*Samaneasaman* (Jacq.) Merr. [≡ *Mimosasaman* Jacq.]

#### Description.

**Figure 253.**
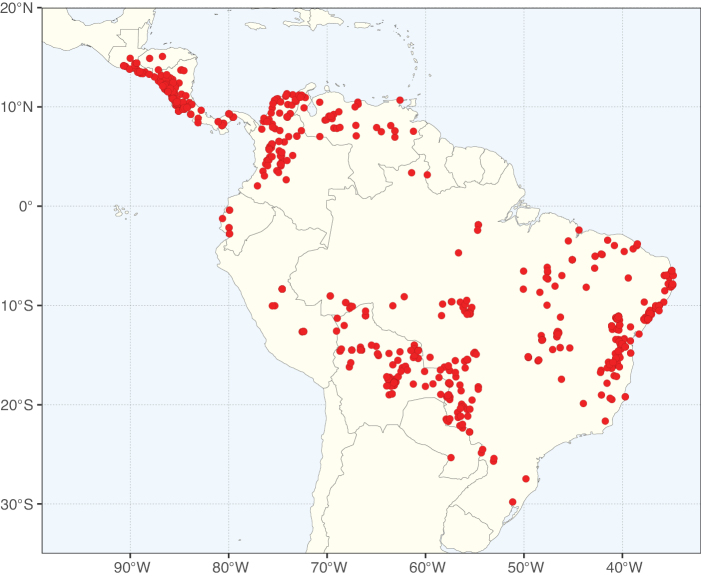
Flower, fruit and vegetative characters of the Samanea clade **A***Samaneainopinata* (Harms) Barneby & J.W. Grimes, tree, Brazil **B, C***Samaneasaman* (Jacq.) Merr. **B** inflorescences in bud and mature flower, El Salvador (*Hughes 1241*) **C** bipinnate leaves, Sri Lanka **D***Samaneainopinata*, rough bark of trunk and main branches, Brazil **E, F***Samaneasaman***E** flowers, cultivated in Hawaii **F** fruits **G***Chloroleuconmangense* (Jacq.) Barneby & J.W. Grimes, trunk bark, Comayagua valley, Honduras **H, I***Chloroleuconacacioides* (Ducke) Barneby & J.W. Grimes **H** fruits, Mato Grosso, Brazil **I** foliage and inflorescences, Brazil **J***Chloroleuconfoliolosum* (Benth.) G.P. Lewis, foliage and fruit, Bahia, Brazil (*Lewis 1972*). Photo credits **A, D, F** E Ner **B, G** CE Hughes **C** P Rajatewa **E** GD Carr **H** D Sasaki **I** D Cardoso **J** GP Lewis.

Unarmed trees (Fig. 253A, D), some attaining great age, height, and crown width; brachyblasts absent. **Stipules** lanceolate, caducous. **Leaves** bipinnate, terminating in a pair of pinnae (Fig. 253C), extrafloral nectaries present; pinnae 3–6 (7) pairs, opposite; leaflets 3–9 pairs, opposite, obliquely oblong to rhombic obovate, inequilateral, pinnately veined. **Inflorescences** axillary umbelliform glomerules (Fig. 253B). **Flowers** dimorphic; peripheral flowers shortly pedicellate (Fig. 253E); calyx vase-shaped; corolla trumpet-shaped; androecium with (16) 20–36 stamens, the staminal tube included in the corolla, filaments pink or reddish, the basal portion sometimes white (Fig. 253E); pollen in 32-celled polyads and perforate tecta; ovary (sub)sessile, narrowly ellipsoid; central flower stouter and sessile with more numerous stamens. **Fruit** broadly linear, indehiscent (seeds released as the fruit decays or animal dispersed), straight or nearly so (Fig. 253F), compressed or plump, with an incrassate pericarp, thin glabrous or puberulent exocarp, a thick sweet nutritive pulpy mesocarp and a crustaceous-woody endocarp. **Seeds** oblong-ellipsoid, the testa hard, with an open U-shaped pleurogram.

#### Chromosome number.

2*n* = 26 (*Samaneasaman*) (Goldblatt and Davidse 1977; Santos et al. 2012).

#### Included species and geographic distribution.

**Figure 254.**
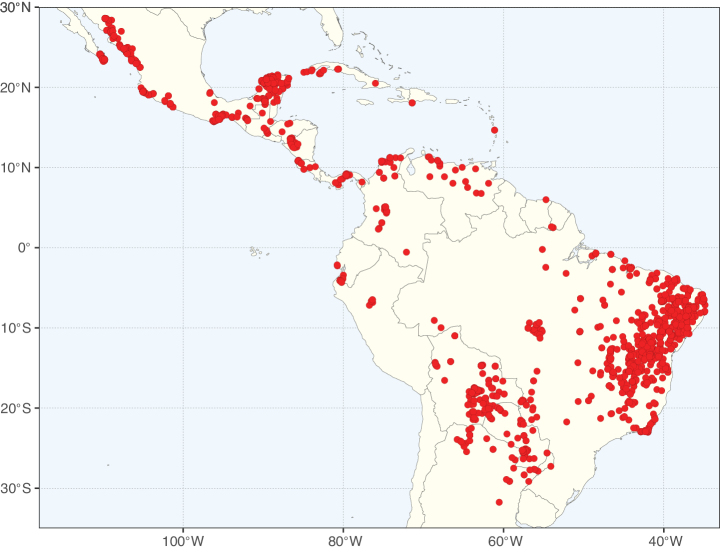
Distribution of *Samanea* based on quality-controlled digitised herbarium records. See Suppl. material 1 for the source of occurrence data.

Three species, mostly circum-Amazonian in tropical continental Central and South America, native from El Salvador in Central America south eastwards through Venezuela, Colombia, Peru and Ecuador, to north-eastern Bolivia, southern, eastern and north-eastern Brazil, and Paraguay. *Samaneasaman* is widely cultivated and partly naturalised as far north as Mexico, and long established in the West Indies in parks and gardens (Fig. 254). It is also widely planted in the Old World tropics as an ornamental, or plantation shade tree.

#### Ecology.

Seasonally dry deciduous to moist evergreen forest, woodland, and wooded grassland.

#### Etymology.

‘Saman’ is derived from the French Caribbean vernacular ‘zamang’ or ‘rain tree’, the leaves fold up at dusk or at the approach of storms.

#### Human uses.

Widely planted pantropically for shade (especially coffee), preserved in pastures as cattle-shade, planted as an ornamental and for nutritious fruits (for animal fodder, human food and for beverages), also for medicine and timber (for a wide range of uses from construction and panelling to furniture and veneers), and for bee forage (Lewis and Rico Arce 2005).

#### Notes.

The species now included in *Samanea* were included in several different genera of the old sense tribe Ingeae. Nielsen (1981a) subsumed them into his broadly defined circumtropical genus *Albizia* Duraz. until the genus was reinstated by Barneby and Grimes (1996), who recognised three species, and provided a key to identify them.

#### Taxonomic references.

Barneby and Grimes (1996); Lewis and Rico Arce (2005).

### ﻿ Chloroleucon

Taxon classificationPlantaeFabalesFabaceae

(Benth.) Britton & Rose, N. Amer. Fl. 23(1): 36. 1928 nom. cons.

Figs 253 , 255

Pithecellobium sect. Chloroleucon Benth., Lond. J. Bot. 3: 197, 221. 1844. Type: Pithecellobiumvincentis Benth. [≡ Chloroleuconmangensevar.vincentis (Benth.) Barneby & J.W. Grimes]

#### Type.

*Chloroleuconvincentis* (Benth.) Britton & Rose [≡ *Pithecellobiumvincentis* Benth. (≡ Chloroleuconmangensevar.vincentis (Benth.) Barneby & J.W. Grimes)]

#### Description.

Trees and shrubs, with dimorphic vegetative branches, the bark usually smooth and a patchwork of grey, white, and green (Fig. 253G), usually randomly armed with solitary or paired axillary spines (modified sterile peduncles), with characteristic striate resting buds and flowering usually preceding leafing; brachyblasts often present. **Stipules** subulate, filiform, linear, or oblanceolate-elliptic, caducous, often lacking. **Leaves** bipinnate (Fig. 253I–J), partly or wholly caducous, extrafloral nectaries present on petiole, and leaf and pinnae rachides; pinnae 1–9 pairs, opposite; leaflets (4) 7–46 pairs, opposite or subopposite, reduced to 1 pair in *Ch. chacoënse* (Burkart) Barneby & J.W. Grimes. **Inflorescences** solitary or fasciculate, pedunculate, capitate (Fig. 253I) or densely shortly racemose-spicate, emerging from a cone of imbricate, striately veined, caducous perules. **Flowers** of each inflorescence either homomorphic or commonly dimorphic (Fig. 253I) and then the terminal (central) one stouter and with a modified androecium, perianth usually 5-merous, calyx narrowly campanulate, short-toothed, corolla narrowly tubular, androecium with 10–30 stamens (up to 52 in *Chloroleucon chacoënse*), exserted or not from the corolla, filaments white to greenish; pollen in 16, 18, 24 or 32-celled polyads with almost smooth ornamentation; ovary sessile, stigma minutely dilated. **Fruits** linear or narrowly oblong, straight, falcate (Fig. 253J), or coiled into a compressed or open helix (Fig. 253H), or erratically twisted, the valves woody or coriaceous, dehiscence tardy and inert through one or both sutures. **Seeds** compressed-lenticular, testa hard, pleurogram present.

#### Chromosome number.

2*n* = 26 [*Ch.tenuiflorum* (Benth.) Barneby & J.W. Grimes; Darlington and Wylie (1956); Fedorov (1969)].

#### Included species and geographic distribution.

**Figure 255.**
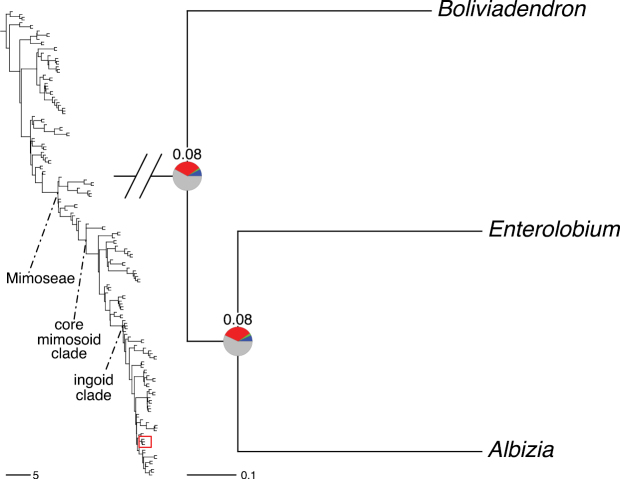
Distribution of *Chloroleucon* based on quality-controlled digitised herbarium records. See Suppl. material 1 for the source of occurrence data.

Ten species, north-west Mexico (one species) to the Antilles (two species), with the remaining species in South America as far south as Argentina and south-east Brazil (Fig. 255). *Chloroleuconmangense* (Jacq.) Barneby & J.W. Grimes was separated into six varieties by Barneby and Grimes (1996).

#### Ecology.

Warm temperate and tropical lowland, less often in submontane seasonally dry forest, xeromorphic brush-woodland, coastal thicket, wooded grassland, shrubland and desert.

#### Etymology.

From Greek, *chloro*- (= greenish yellow) and *leuco*- (= white), probably referring to the patchwork bark of most species.

#### Human uses.

Used for construction timber, as a medicinal tea, and the fruits as human food [*Ch.dumosum* (Benth.) G.P. Lewis in Bahia, Brazil; Queiroz (2009)].

#### Notes.

Record (1927) was the first to publish the generic name *Chloroleucon* and cited *Ch.guatemalense* Britton & Rose ex Record [now Ch.mangensevar.leucospermum (Brandegee) Britton & Rose] as the generic type. However, it was not validly published because he did not provide a generic description.

The species of *Chloroleucon* separate into two well supported groups based on whether the flowers in the inflorescence are homomorphic or heteromorphic (Almeida et al., in prep.). Barneby and Grimes (1996) provide a key to the ten species and to the six varieties recognised in *Ch.mangense*.

#### Taxonomic references.

Barneby and Grimes (1996); Lewis and Rico Arce (2005).

## ﻿﻿36. Albizia clade

Erik J. M. Koenen^26^

Citation: Koenen EJM (2024) 36. Albizia clade. In: Bruneau A, Queiroz LP, Ringelberg JJ (Eds) Advances in Legume Systematics 14. Classification of Caesalpinioideae. Part 2: Higher-level classification. PhytoKeys 240: 451–465. https://doi.org/10.3897/phytokeys.240.101716

**Albizia clade**

Figs 256–263

**Included genera (3).***Albizia* Durazz. (ca. 90 species), *Boliviadendron* E.R. Souza & C.E. Hughes (1), *Enterolobium* Mart. (8).

**Description.** Small to very large trees to 45 m with large spreading crown or sometimes shrubs, rarely scandent or lianescent shrubs or a liana (one species of *Albizia*), usually unarmed except for a few species of *Albizia* with spinescent shoots, prickles/hooks or recurved thorn-like stipules. **Stipules** usually small, linear to linear-triangular and caducous, sometimes bigger and leaf-like and persistent, or in one species recurved and thorn-like and caducous. **Leaves** bipinnate, micro- or macrophyllidinous, with an extrafloral nectary and usually with further glands between the pinnae and leaflets; leaflets usually asymmetrical with palmate-pinnate venation and the midrib displaced to either side of the leaflet, or, particularly in several macrophyllidinous species, symmetrical with pinnate venation. **Inflorescences** globose, sub- or hemiglobose, or corymbiform capitula, usually dimorphic with one or a few enlarged central flowers with broad nectariferous base and exserted staminal tube, or homomorphic, either fasciculate from leaf axils or mostly aggregated into larger paniculate compound inflorescences with leaf development suppressed. **Flowers** 5-merous, sessile or often especially the peripheral flowers of capitula pedicellate, with (slenderly) campanulate or funnel-shaped calyx and corolla, stamens fused into a tube for about the same length as the corolla or slightly exserted, sometimes the tube much longer and exserted well beyond the corolla lobes; pollen shed in 16- or 32-celled flat disk-shaped polyads. **Fruits** usually flat and straight or slightly curved non-septate, with papery valves, dehiscent or tardily dehiscent along both sutures or indehiscent, or septate reniform-auriculiform, annular or contorted indehiscent, with thick coriaceous to woody valves and then often with soft mesocarp, or lomentiform, and then sometimes breaking up into segments or tardily so, or straight to slightly curved, thick, woody and indehiscent. **Seeds** elliptical and laterally flattened, with a hard smooth testa and usually with open or closed pleurogram, sometimes lacking, rarely (*Boliviadendron*) the testa thin and the seed with a minute wing, pleurogram lacking.

**Distribution.** Pantropical, with the two smaller genera *Boliviadendron* and *Enterolobium* restricted to the Americas and the large genus *Albizia* distributed across Africa, Madagascar, tropical Asia, Malesia, Australia and some Pacific islands, and a few species extending into the temperate zone in continental Asia.

**Figure 256.**
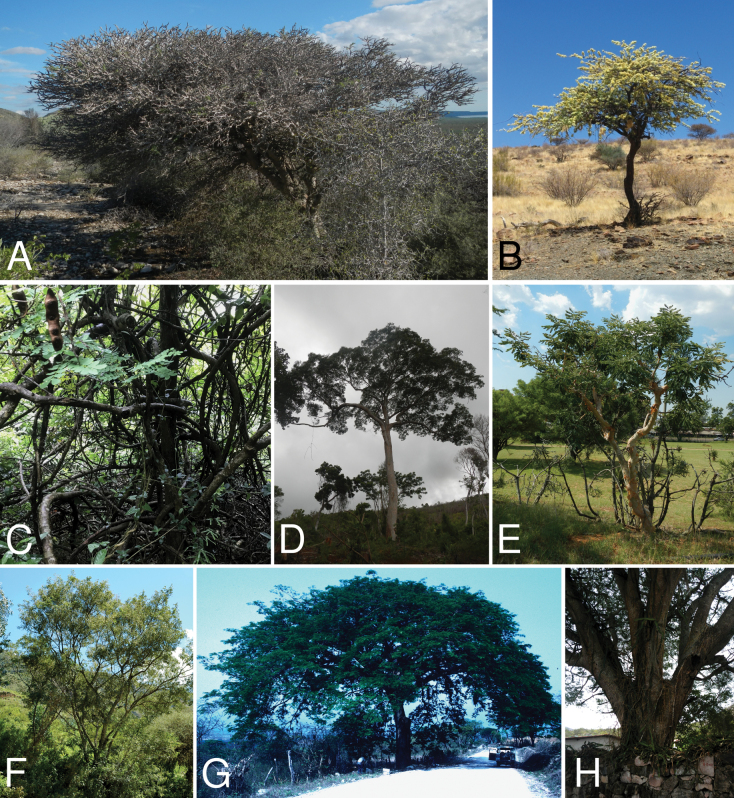
Generic relationships in the Albizia clade (tribe Mimoseae). Clades within *Albizia* are indicated in Suppl. materials 2, 3. For description of phylogeny and support values, see Fig. 6 caption (page 63).

**Notes.** The Albizia clade includes a more narrowly defined *Albizia* that is restricted to the Old World and its two closest relatives *Boliviadendron* and *Enterolobium* that are only found in the Americas. The clade is well supported in recent phylogenomic studies (Koenen et al. 2020a; Ringelberg et al. 2022) and is likely closely related to the Samanea clade, although support for that relationship is poor. A close relationship between *Albizia* and *Enterolobium* was suggested by Mesquita (1990), but the former genus was poorly defined at the time. Barneby and Grimes (1996) considered both genera as *incertae sedis* in their informal classification of the American ingoids into alliances, but noted that *Albizia* as a pantropical genus needed to be revised at the global scale. *Enterolobium* and *Albizia* have now been shown to be sister genera (Koenen et al. 2020a; Ringelberg et al. 2022), and both genera have been redefined in accordance with phylogenetic data (Souza et al. 2022b; Peraza et al. 2022; Koenen 2022a, 2022b; Soares MVB et al. 2022; see also generic notes below). *Boliviadendron* was recently described to accommodate a single Bolivian endemic species that was formerly included in *Leucochloron* Barneby & J.W. Grimes (Souza et al. 2022a), and for which a close relationship with *Albizia* and *Enterolobium* was not expected.

**Clade based definition.** The most inclusive crown clade containing *Albiziajulibrissin* Durazz. and *Boliviadendronbolivianum* (C.E. Hughes & Atahuachi) E.R. Souza & C.E. Hughes, but not *Samaneasaman* (Jacq.) Merr., *Blanchetiodendronblanchetii* (Benth.) Barneby & J.W. Grimes, *Ingavera* Willd. or *Jupunbatrapezifolia* Moldenke (Fig. 256).

**Figure 257.**
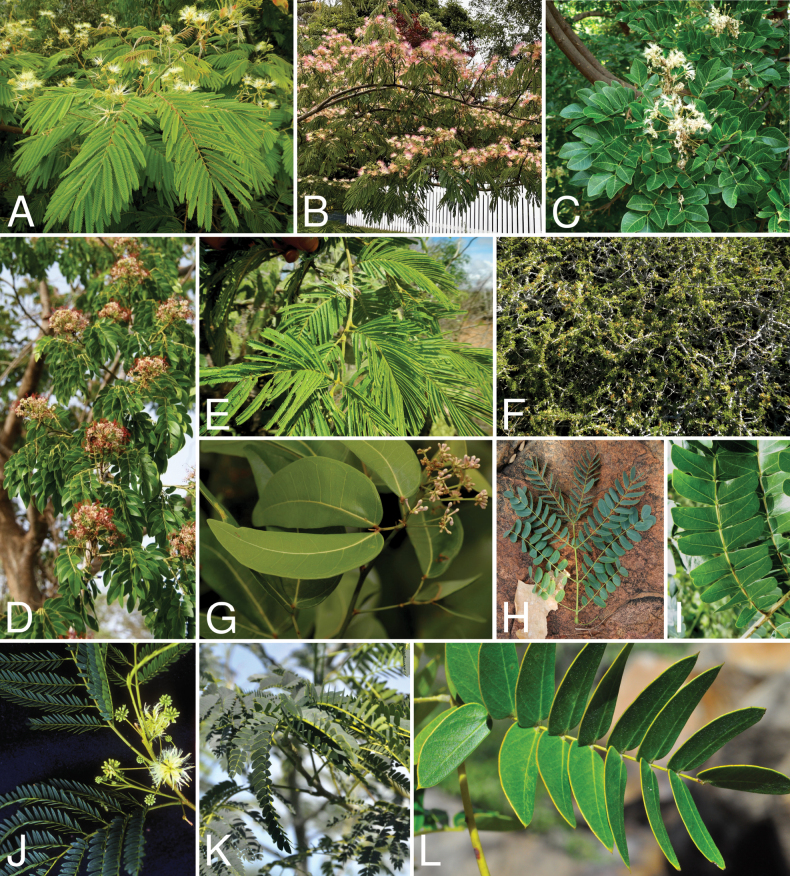
Habit diversity of the Albizia clade **A***Albiziaatakataka* Capuron, a densely branched small tree in dry thorn-scrub in south-western Madagascar **B***A.anthelmintica* Brongn., a small tree flowering before the leaves appear, in semi-arid regions of southern Africa **C***A.corniculata* (Lour.) Druce, a scandent shrub or liana in humid forests in continental South East Asia, the Philippines and northern Borneo **D***Albizia* sp. (*Koenen 434*), a large tree in semi-deciduous forest in north-western Madagascar **E***A.tanganyicensis* Baker f., a small tree in dry savanna woodland and (sub)montane grassland in southern and eastern Africa **F***Boliviadendronbolivianum* (C.E. Hughes & Atahuachi) E.R. Souza & C.E. Hughes (*Hughes 2608*), a tree in seasonally dry tropical forest in Bolivia **G***Enterolobiumcyclocarpum* (Jacq.) Griseb. (*Hughes 1254*) **H** trunk of *E.contortisiliquum* (Vell.) Morong, both trees of seasonally dry and semi-deciduous forests of South and Central America. Photo credits **A, D, E, H** E Koenen **B** A Dreyer, www.africanplants.senckenberg.de**C** Biobank Lantauhk (Hong Kong), iNaturalist (https://www.inaturalist.org/photos/66503444) **F, G** CE Hughes.

**Figure 258.**
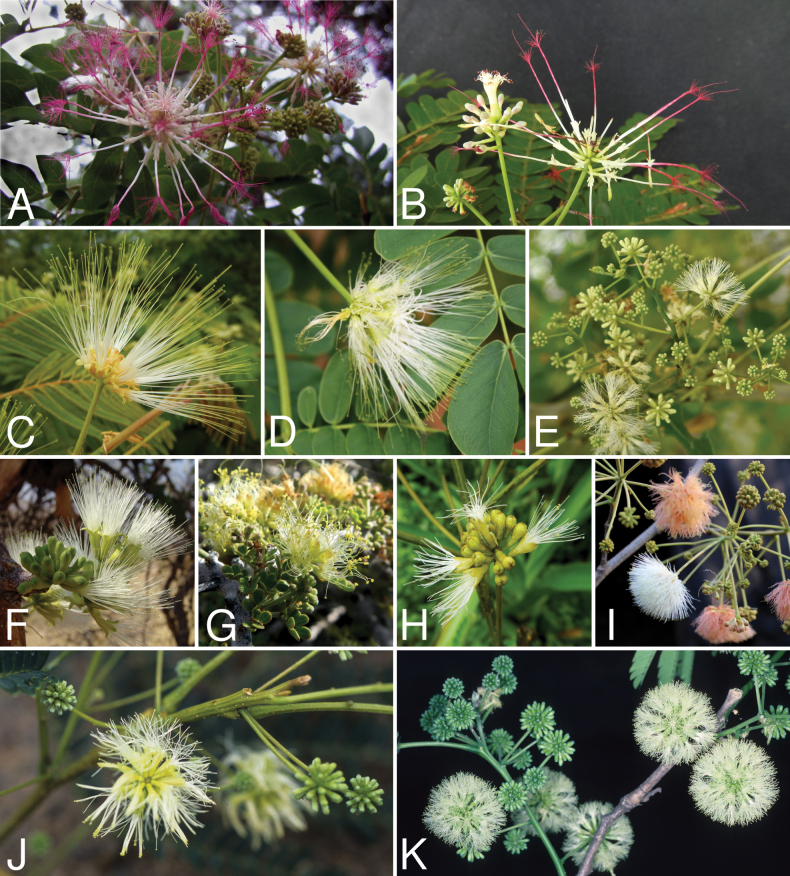
General and vegetative morphology of the Albizia clade **A***Albiziachinensis* (Osbeck) Merr., flowering branch (*Koenen 190*) **B** cultivated *A.julibrissin* Durazz., flowering branch **C***A.glaberrima* Benth., foliage and inflorescences **D***A.zygia* J.F. Macbr., flowering branch **E***A.polyphylla* Fourn., finely bipinnate foliage (*Koenen 256*) **F***A.atakataka* Capuron, dense branching with the leaves arising from brachyblasts (*Koenen 229*) **G***A.laurentii* De Wild., flower buds and quadrifoliolate bipinnate leaves **H***A.tanganyicensis* Baker f., leaf **I***A.gummifera* C.A. Sm., detail of pinna with rhombic leaflets **J***Boliviadendronbolivianum* (C.E. Hughes & Atahuachi) E.R. Souza & C.E. Hughes, foliage and flowers (*Hughes 2423*) **K***Enterolobiumcontortisiliquum* (Vell.) Morong, foliage **L***E.timbouva* Mart., detail of pinna. Photo credits **A, E, F, H** E Koenen **B** M Fitzgerald **C** G Baumann **D** W Dijkstra **G** D Harris **I** I Dinter **J** CE Hughes **K** A Martinez Ponte, iNaturalist (https://www.inaturalist.org/photos/195358543) **L** F Acaz Sonntag, iNaturalist (https://www.inaturalist.org/photos/216600814) **B–D, G, I**www.africanplants.senckenberg.de.

**Figure 259.**
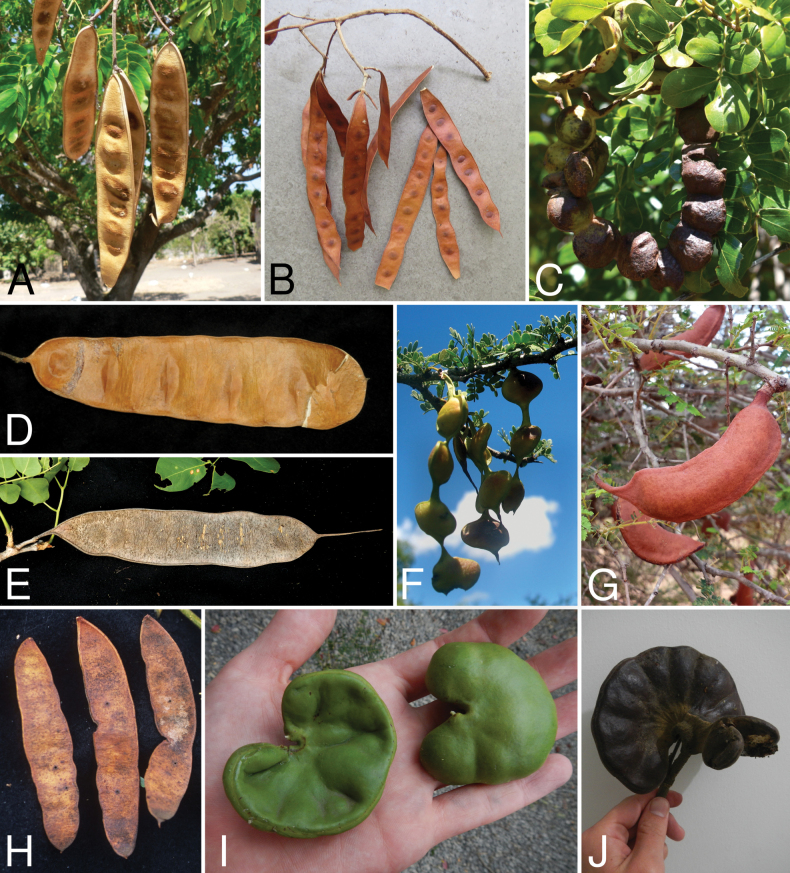
Inflorescences of the Albizia clade **A***Albiziagrandibracteata* Taub., strongly dimorphic capitula (*Koenen 159*) **B***A.mainaea* Villiers of AlbiziasectionZygia, strongly dimorphic capitula (*Koenen 426*) **C***A.chinensis* (Osbeck) Merr., more cryptic dimorphic capitula (*Koenen 190*) **D***A.lebbeck* (L.) Benth., more cryptic dimorphic capitula **E***A.procera* (Roxb.) Benth., homomorphic capitula **F***A.anthelmintica* Brongn., dimorphic capitula **G***A.atakataka* Capuron, dimorphic capitula (*Koenen 229*) **H***A.forbesii* Benth., dimorphic capitula **I***A.zimmermannii* Harms, homomorphic capitula **J***Boliviadendronbolivianum* (C.E. Hughes & Atahuachi) E.R. Souza & C.E. Hughes, homomorphic capitula (*Hughes 2423*) **K***Enterolobiumcyclocarpum* (Jacq.) Griseb., homomorphic capitula (*Hughes 1254*). Photo credits **A–C, G, H** E Koenen **D** M Schmidt **E** R Cumming, iNaturalist (https://www.inaturalist.org/photos/168379143) **F** I Dinter **I** C Boucher Chisale **J, K** CE Hughes **D, F, I**www.africanplants.senckenberg.de.

**Figure 260.**
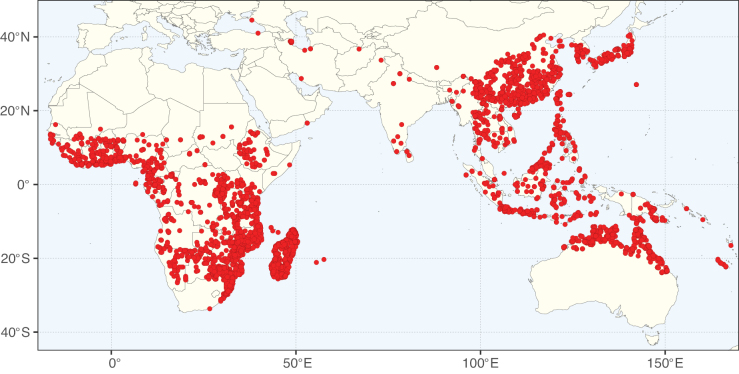
Fruits of the Albizia clade **A***Albizialebbeck* (L.) Benth., flat papery fruits and **B***A.procera* (Roxb.) Benth., flat papery fruits **C***A.moniliformis* (DC.) F. Muell., lomentaceous fruits **D***A.ferruginea* (Guill. & Perr.) Benth., flat papery fruits (*Harris 9726*) **E***A.boivinii* Fourn., flat papery fruits (*Koenen 376*) **F***A.commiphoroides* Capuron, moniliform fruits **G** of *A.masikororum* R. Vig., thick woody fruits **H***Boliviadendronbolivianum* (C.E. Hughes & Atahuachi) E.R. Souza & C.E. Hughes, flat papery fruits (*Hughes 2423*) **I***Enterolobiumcontortisiliquum* (Vell.) Morong, thick coriaceous ear-shaped fruits (*Queiroz 15579*) **J***E.monjollo* (Vell.) Mart., thick coriaceous ear-shaped fruits (*Lima 7911*). Photo credits **A** G Baumann www.africanplants.senckenberg.de**B** I Cowan, iNaturalist (https://www.inaturalist.org/photos/54408314) **C** R Cumming, iNaturalist (https://www.inaturalist.org/photos/200251197) **D** D Harris www.africanplants.senckenberg.de**E, I, J** E Koenen **F** Désiré Ravelonarivo, Tropicos (https://www.tropicos.org/ImageFullView.aspx?imageid=100291301) **G** SE Rakotoarisoa, iNaturalist (https://www.inaturalist.org/photos/3113769) **H** CE Hughes.

The clade is relatively uniform in vegetative (Figs 257, 258) and floral (Fig. 259) characters, although quantitative variation in leaf traits is rather large in *Albizia* and the presence of dimorphic versus monomorphic inflorescences is variable. In fruit (Fig. 260) and seed morphology, however, the clade is more diverse and fruit and seed traits can be used to separate the genera as well as some species groups in *Albizia*. The fruit of *Enterolobium* stands out in being reniform-auriculiform, annular or contorted (Fig. 260I, J) and indehiscent, with a dry-mealy or resinous-pulpy mesocarp and septate endocarp. The fruit of *Boliviadendron* is a flat and straight papery legume (Fig. 260H) that is tardily dehiscent along both sutures as in many *Albizia* species, but it has seeds with a narrowly winged margin not seen in *Albizia* or in *Enterolobium*. The fruits in most *Albizia* species are flat and papery (Fig. 260A, B, D, E), with variable dehiscence, but several phylogenetically scattered exceptions occur, including curved to twisted or contorted lomentiform fruits (Fig. 260C, F) with papery or woody valves, that in some species tardily break up into articles, or thick woody indehiscent fruits (Fig. 260G), but never with a soft mesocarp as in *Enterolobium*.

### ﻿ Albizia

Taxon classificationPlantaeFabalesFabaceae

Durazz., Mag. Tosc. 3(4): 13. 1772.

**Figure 261.**
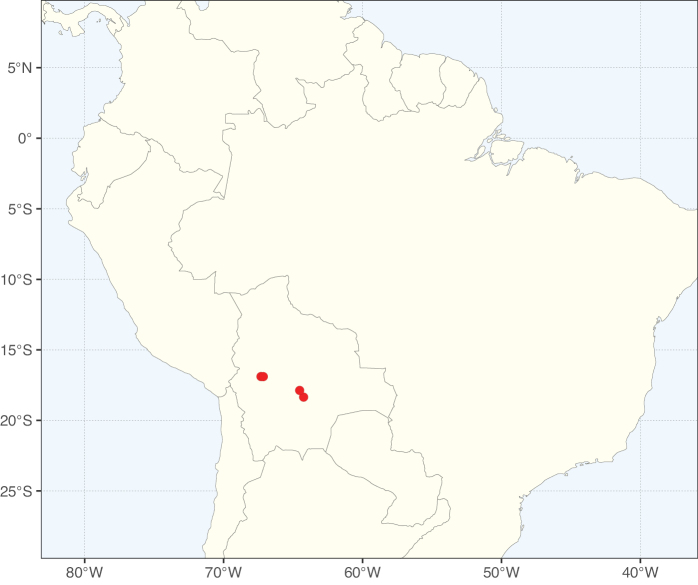
Distribution of *Albizia* based on quality-controlled digitised herbarium records. See Suppl. material 1 for the source of occurrence data.

Figs 257 , 258 , 259 , 260 , 261

Sassa Bruce ex J.F. Gmel., Syst. Nat., ed. 13[bis]. 2(2): 1038. 1792. Type: Sassagummifera J.F. Gmel. [≡ Albiziagummifera (J.F. Gmel.) C.A. Sm.]
Besenna A. Rich., Tent. Fl. Abyss. 1: 253. 1847. Type: Besennaanthelmintica A. Rich. [≡ Albiziaanthelmintica (A. Rich.) Brongn.]
Arthrosprion Hassk., Retzia i: 212. 1855. Type: Arthrosprionstipulatum (DC.) Hassk. [≡ Acaciastipulata DC. (= Albiziachinensis (Osbeck) Merr.)]
Cathormion (Benth.) Hassk., Retzia 1: 231. 1855. Type: Cathormionumbellatum (Vahl) Kosterm. [≡ Albiziaumbellata (Vahl) E.J.M. Koenen]
Parasamanea Kosterm., Organ. Natuurw. Onderz. Indonesië 20: 11. 1954. Type: Parasamanealandakensis (Kosterm.) Kosterm. [≡ Albiziarosulata subsp. landakensis (Kosterm.) I.C. Nielsen]
Serialbizzia Kosterm., Organ. Natuurw. Onderz. Indonesië 20: 15. 1954. Type: Serialbizziaacle (Blanco) Kosterm. [≡ Albiziaacle (Blanco) Merr.]
Parenterolobium Kosterm., Organ. Natuurw. Onderz. Indonesië 20: 19. 1954. Type: Parenterolobiumrosulatum (Kosterm.) Kosterm. [≡ Albiziarosulata Kosterm.]

#### Type.

*Albiziajulibrissin* Durazz.

#### Description.

Small to very large trees to 45 m with large rounded or flat-topped spreading crown or rarely shrubs, and then sometimes scandent or lianescent, or a liana to 45 m [*Albiziacorniculata* (Lour.) Druce], usually unarmed except for a few species with spinescent shoots [*A.anthelmintica* Brongn., *A.moniliforme* (DC.) F. Muell. and *A.umbellata* (Vahl) E.J.M. Koenen] or prickles/hooks (*A.corniculata*, *A.myriophylla* Benth. and *A.rufa* Benth., these species with a scandent shrubby or lianescent habit) or recurved thorn-like stipules (in *A.pedicellata* Baker ex Benth.), short shoots or brachyblasts sometimes present. **Stipules** small linear to triangular, usually caducous, sometimes larger, rounded, leaf-like and persistent or caducous, or recurved, thorn-like and caducous in one species (*A.pedicellata*). **Leaves** bipinnate, macro- or microphyllidinous, with 1–25 pairs of pinnae, the petiole with an extrafloral nectary and usually with further nectaries between the apical pairs of pinnae and leaflets; leaflets 1–47 pairs, small and linear to large and elliptical, rounded or rhombic, frequently (especially in microphyllidinous species) the midrib displaced to either side of the leaflet blade and often several additional strong veins starting from the leaflet base (i.e., palmate-pinnate venation), in the macrophyllidinous species often with pinnate venation, base oblique to equal, apex rounded, acute or mucronate. **Inflorescence units** sub- or hemiglobose, often corymbiform, or sometimes globose, capitula, usually aggregated into terminal or axillary panicles with the leaves suppressed, otherwise fascicular in leaf-axils. **Flowers** 5-merous,mostly white or cream, or often bicoloured with pink to red upper half of stamens, sessile or often especially the peripheral flowers pedicellate, dimorphic or sometimes monomorphic, often the central flower enlarged and more robust with a broad cupular nectariferous base and longer staminal tube; calyx slenderly campanulate or funnel-shaped; corolla funnel-shaped; stamens numerous, fused into a tube not or slightly exserted beyond the corolla lobes, in terminal flowers usually well-exserted and in some species the tube very long in all flowers and exserted well beyond the corolla lobes, anthers dorsifixed, eglandular; pollen shed in 16-celled flat disk-shaped polyads (but only a few species have been studied); ovary sessile or shortly stipitate, the style approximately the same length as the stamens or extending slightly beyond the anthers, stigma funnel-shaped. **Fruit** usually straight, flat, papery, dehiscent or inertly dehiscent along both sutures or indehiscent, or straight or slightly curved, indehiscent and thick woody (in several Madagascan species), or straight to curved or twisted and moniliform (in *A.atakataka* Capuron and *A.commiphoroides* Capuron) or straight to twisted, septate, lomentiform, breaking up into one-seeded segments (in *A.moniliforme* and *A.umbellata*) or straight or curved, septate and coriaceous [in *A.acle* (Blanco) Merr.] or contorted, septate and woody [in *A.rosulata* (Kosterm.) I.C. Nielsen and *A.dolichadena* (Kosterm.) I.C. Nielsen] tardily breaking up into articles. **Seeds** elliptical to rounded, laterally compressed, usually with a large subcircular or narrowly elliptic closed pleurogram, or a small, rounded deltoid to subcircular pleurogram in the upper half of the seed, or sometimes a U-shaped pleurogram open towards the hilum.

#### Chromosome number.

2*n* = 26, one species (*A.polyphylla* Fourn.) is reported to be an octoploid with 2*n* = 104 (Darlington and Wylie 1956).

#### Included species and geographic distribution.

ca. 90 species, in Africa, Madagascar, (sub)tropical Asia, Malesia, Australia and several Pacific Islands (Fig. 261). Centres of diversity are in South East Asia and in the East African Great Lakes region and Madagascar.

#### Ecology.

*Albizia* has a wide ecological range with species found in coastal, riverine and swamp forests, terra firme rainforest, seasonally dry deciduous forest, savannas and (sub)montane forest. Although most species of *Albizia* are tropical, a few Asian species, including the type species *A.julibrissin* Durazz., are mildly frost-tolerant and extend into the subtropical and warm temperate zone, as well as cooler (sub)montane regions. Most species are medium sized trees, in either primary vegetation or as coloniser species in secondary vegetation, with a few species occurring as large canopy trees while some of the semi-arid species remain small shrubby trees and a few Asian species are scandent shrubs to lianas at forest edges and disturbed sites or in primary and secondary forests (Fig. 257A–E).

#### Etymology.

Named for Filippo degli Albizzi, an 18^th^ century Italian naturalist from the wealthy Albizzi family of Tuscany, who reportedly brought seeds of the species from Constantinople to Florence in 1749. The spelling “Albizzia” has historically been used and would be the correct spelling given the Albizzi family name, however, in the original publication the spelling *Albizia* was used and hence this has to be considered the correct name (Nielsen 1979). This is particularly confusing since the recently reinstated *Pseudalbizzia* was spelled with two ‘z’ in the original publication.

#### Human uses.

The wood of several species is used for various purposes, including house construction, furniture, boat building, musical instruments, as well as for firewood and charcoal. Several species are planted as shade trees in coffee and tea plantations and for soil improvement, and as street trees or ornamentals in parks and gardens (Fig. 258B). Some species yield a gum that is similar to gum arabic, and dyes and tannins are extracted from the bark, particularly in *A.lebbekoides* (DC.) Benth. The bark of *A.saponaria* Blume ex Miq. and several other species contains saponin and can be used as a substitute for soap. The bark of several species is used as a fish poison and shows insecticidal activity. Leaves and seeds of some species are used for food or as animal fodder. Leaves, bark and roots of various species are used medicinally, e.g., *A.adianthifolia* (Schumach.) W. Wight, *A.amara* (Roxb.) Boivin and several other African species with numerous applications in traditional medicine across Africa (Louppe et al. 2008) and the bark of *A.anthelmintica* is used against tapeworm.

#### Notes.

*Albizia* was described by Durazzini (1772) from specimens of *A.julibrissin*, a native of warm temperate and subtropical Asia from Turkey to Japan, that were planted in Florence. The genus has since known a turbulent taxonomic history, having been very broadly circumscribed in the past when it was considered a “dustbin” genus in which to place species that had no clear affinity to other existing ingoid genera. Nielsen (1979, 1985b), in a series of publications, delimited the genus in the Asian, Australian and Pacific region, and transferred several species that were described or previously included in *Albizia* to the genera *Archidendron* F. Muell. (Nielsen et al. 1984a), *Archidendropsis* I.C. Nielsen, *Paraserianthes* I.C. Nielsen, *Pararchidendron* I.C. Nielsen and *Serianthes* Benth. (Nielsen et al. 1983a, 1983b, 1984b; Archidendron clade, page 404). He considered *Cathormion* (Benth.) Hassk. a monotypic genus distinct from *Albizia* (Nielsen 1979, 1992). Although *Cathormion* has now been included in *Albizia* (Koenen et al. 2020a), Nielsen’s taxonomy is otherwise mostly well-supported by phylogenies (Koenen et al. 2020a; Ringelberg et al. 2022; Brown et al. 2022; Demeulenaere et al. 2022; Koenen, unpubl. data). The African species are comprehensively but patchily covered by regional and national flora treatments across the continent (e.g., Hutchinson and Dalziel 1958; Brenan 1959, 1970; Villiers 1989; Thulin 1993). Five African species that had previously been included in *Albizia* by some authors while placed in *Cathormion* and/or *Samanea* (Benth.) Merr. by others, are now accommodated in either *Hydrochorea* Barneby & J.W. Grimes (Soares MVB et al. 2022; Jupunba clade, page 437) or *Osodendron* E.J.M. Koenen (Koenen 2022b; Inga clade, page 466). The Madagascan species were reviewed by Villiers (2002), but for several species incomplete material was available at the time and the genus remains relatively poorly known on the island and in need of further taxonomic study. Barneby and Grimes (1996) monographed the *Albizia* species of the Americas, but these species have now been shown to be more closely related to the Abarema alliance (Koenen et al. 2020a; Ringelberg et al. 2022) and have been transferred to a reinstated *Pseudalbizzia* Britton & Rose (Peraza et al. 2022) and included in the Jupunba clade (page 437), with the exception of *Albiziacarbonaria* Britton, which is now placed in *Pseudosamanea* Harms (Koenen 2022a; see page 434).

Following these recent taxonomic updates, the species that remain accommodated in *Albizia* under the circumscription that is presented here, have been shown to form a monophyletic group in preliminary phylogenomic analyses that sampled 75 of the ca. 90 species (Koenen et al., unpubl. data; Ringelberg et al. 2023; Suppl. material 2). These preliminary results show that the genus consists of four geographically structured clades: (1) a clade that includes the type species *A.julibrissin* and ca. 38 species that are found from Turkey in the West across continental Asia and Malesia to Australia and some Pacific islands in the East; (2) Albiziasect.Zygia Benth. is a small clade of 10 species that is widespread across sub-Saharan Africa and Madagascar and in which the stamens are characteristically fused into a tube for most of their length with short radially spreading free parts of the filaments (Fig. 259A, B); (3) a clade with ca. 11 species found across Africa; and (4) a clade of ca. 31 species predominantly distributed in Madagascar, but including six species from continental Africa of which one species (*A.amara*) extends into continental Asia in India and Sri Lanka. These four clades are each well-supported as monophyletic, but the relationships among them are not well-resolved.

Nearly all species of *Albizia* are fundamentally trees (Fig. 257A, B, D, E), although they may become multi-stemmed and shrub-like presumably due to resprouting after the main stem has become damaged. Three Asian species, *Albiziacorniculata*, *A.myriophylla* and *A.rufa*, that have a scandent shrubby or lianescent habit (sometimes growing into a liana to 45 m), form an exception (Fig. 257C). These species were placed in the informal “Albiziacorniculata group” by Nielsen (1985b). Interestingly, these three species form a grade to a small clade of tree species (Koenen et al., unpubl. data), suggesting the lianescent habit evolved once and was subsequently reversed once in the descendant lineage leading to the small tree species clade. These three lianescent species have an unusual form of armature where a recurved prickle or hook is found beneath the leaf base or leaf scar. The African species *A.anthelmintica* Brongn. and the South East Asian and Australasian species *A.moniliformis* and *A.umbellata* often have spinescent shoots, a type of armature that is common among Mimoseae and has evolved multiple times (Ringelberg et al. 2022). Furthermore, the Malesian species *A.pedicellata* has recurved thorn-like stipules that are caducous. Otherwise, all other species are reported as being unarmed.

As in many Mimoseae genera, the bipinnate leaves vary widely in the numbers of pinna and leaflet pairs per leaf, as well as the size and shape of leaflets (Fig. 258A–I). In many species, the midrib is displaced to the upper side of the leaflets, as also occurs in the two other genera in the Albizia clade. Most species are characterised by dimorphic flowers in each unit inflorescence (Fig. 259A–D, F–H), with one or a few central flowers enlarged and more robust with a nectariferous base, these sessile with peripheral flowers (sub)sessile or often pedicellate. Especially in the African section Zygia, the combination of a nectariferous central flower with peripheral flowers with very long staminal tubes (Fig. 259A, B) are unusual and unlike any other Mimoseae genus. Homomorphic inflorescences (Fig. 259E, I) are also found in a smaller number of species that are phylogenetically scattered across clades 1, 3 and 4 (Suppl. material 2). The unit inflorescences can be arranged in slender efoliate terminal or axillary panicles such as in the type species *A.julibrissin*, or emerging from axillary fascicles. Dense clustering of inflorescences at the apices of branchlets when species are in full bloom means that some species have been used as ornamentals, with especially *A.julibrissin* having been planted extensively in gardens and parks in (warm) temperate regions around the world (Fig. 258B).

Most species have straight flat papery fruits inertly dehiscent along both sutures or indehiscent, that are sometimes slightly swollen around the seeds, but markedly different fruits are found in some Asian and Madagascan species, these placed in clades 1 and 4, respectively (Suppl. material 2):

First, the species *A.moniliformis* (Indonesia to Australia) and *A.umbellata* (continental South East Asia) that are placed in clade 1, have septate lomentiform fruits (Fig. 260C) that break up into one-seeded segments. These two species have previously been treated as subspecies of the type species of the genus *Cathormion*, which at one point was considered a pantropical genus in which several unrelated lomentiform species were placed, and which have now all been transferred to *Chloroleucon* Barneby & J.W. Grimes (Barneby and Grimes 1996), *Hydrochorea* (Soares MVB et al. 2022), *Osodendron* (Koenen 2022b) and *Pseudalbizzia* (Peraza et al. 2022), except for the type species *C.umbellatum* which is now placed in *Albizia* and hence the name *Cathormion* is synonymised with this genus (Koenen et al. 2020a).

Secondly, the five Asian species that Nielsen (1985b) referred to the informal “Serialbizzia group”, also placed in clade 1, have woody to coriaceous fruits that are usually septate and either straight (*A.acle*, *A.attopeuensis* (Pierre) I.C. Nielsen and *A.splendens* Miq.) or contorted (*A.dolichadena* and *A.rosulata*), with especially the woody contorted fruits of the latter two species prone to tardily fragmenting into 1-seeded articles. The Serialbizzia group is not monophyletic, with *A.attopeuensis* with coriaceous valves not grouping with the woody-valved species (but *A.splendens*, that also has coriaceous valves, has not been sampled in phylogenies as of yet; Koenen et al., unpubl. data). Another species in clade 1, *A.salomonensis* C.T. White ex F.S. Walker has very large sub-woody fruits and has been placed in Serialbizzia in the past but is not closely related to the other Asian woody-valved species.

Thirdly, two unusually densely branched (Figs 257A, 258F) species placed in clade 4, endemic to the semi-arid scrub region of south-western Madagascar, *A.atakataka* and *A.commiphoroides*, have (sub)moniliform indehiscent pods that may become fragmented after having been shed, and are strongly constricted between the seeds, especially in the latter species (Fig. 260F).

Finally, seven of the ca. 24 *Albizia* species endemic to Madagascar, all also placed in clade 4, have thick woody indehiscent fruits (Fig. 260G).

#### Taxonomic references.

Bentham (1985); Brenan (1959, 1970[9]) ; Brummitt (1970); Hutchinson and Dalziel (1958); Koenen et al. (2020a: appendix 2); Nielsen (1979, 1981a, 1985b, 1992); Nielsen et al. (1983: p. 43–45); Thulin (1993); Villiers (2002).

### ﻿ Boliviadendron

Taxon classificationPlantaeFabalesFabaceae

E.R. Souza & C.E. Hughes, PhytoKeys 205: 445. 2022.

**Figure 262.**
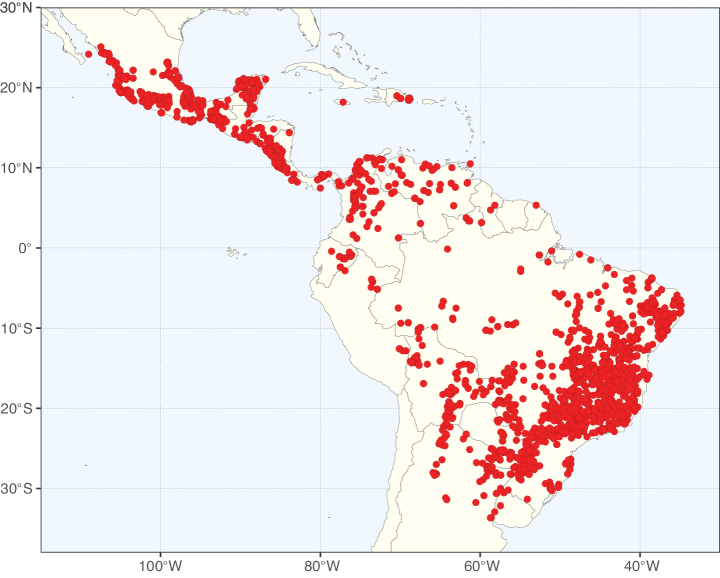
Distribution of *Boliviadendron* based on quality-controlled digitised herbarium records. See Suppl. material 1 for the source of occurrence data.

Figs 257 , 258 , 259 , 260 , 262

#### Type.

*Boliviadendronbolivianum* (C.E. Hughes & Atahuachi) E.R. Souza & C.E. Hughes [≡ *Leucochloronbolivianum* C.E. Hughes & Atahuachi]

#### Description.

Small, unarmed multi-stemmed tree to 5–6 (10) m and 20–35 cm diameter with an irregular spreading crown; resting buds perulate, ovoid to 1.5 mm long and densely pilosulous (as for shoots), but these generally lacking. **Stipules** linear-triangular, caducous. **Leaves** bipinnate with 4–5 (6) pairs of pinnae, petiole with a circular or weakly ellipsoid cupular nectary at or slightly above mid-petiole position; similar smaller nectaries variably present between distal 1–2 pairs of pinnae or sometimes between all pinnae pairs; leaflets 15–20 pairs per pinna, slightly decrescent proximally and distally, linear-oblong, obliquely truncate at base, obtuse at apex, the tip sometimes acute, venation indistinct, palmate-pinnate brochidodromous, with 1–2 (weakly 3) primary veins at base, the main primary vein distinctly asymmetric, dividing the blade 1:2–2.5, giving rise on each side to 3–4 sinuous secondary veins, the first anterior or first pair of leaflets reduced to small paraphyllidia. **Flowers** white, sessile, homomorphic and arranged in lax globose capitula on slender peduncles, each capitulum with 20–25 flowers, the capitula immersed in new foliage, solitary or in fascicles of 2–3 in axils of coeval leaves; calyx narrowly campanulate, corolla tubular, slightly funnel-shaped; androecium of ca. 40 stamens, fused basally into a short tube that is not or hardly exserted beyond the corolla; pollen shed in 16-celled flat disk-shaped polyads; ovary sessile, the style held below anthers of the stamens. **Fruit** 1 per capitulum, sessile, (3) 4–6 (8)-seeded, flat plano-compressed, linear-oblong or oblong, obtuse at each end, sometimes with a short apiculum at apex, the valves straight or with slightly undulate margins, stiff papery, tardily dehiscent along both sutures. **Seeds** flat disciform, broadly suborbicular, the testa thin, lustrous, translucent castaneous, pleurogram lacking, surrounded by a narrow dark marginal nerve or minute wing.

#### Chromosome number.

Unknown.

#### Included species and geographic distribution.

Monospecific (*B.bolivianum*), endemic to Bolivia, where it is only known from the eastern flanks of the Andes at mid-elevations in interior Andean valleys in the Departments of La Paz, Cochabamba and Santa Cruz (Souza et al. 2022a) (Fig. 262).

#### Ecology.

*Boliviadendron* is known only from the slopes of interior valleys of Bolivia between 2150 and 2770 m elevation, around the transition from seasonally dry tropical inter-Andean valley forests to more moist mid-elevation montane Ceja de Monte Yungeña vegetation, where it is locally common in fence-lines, remnant patches of subhumid or seasonally dry Yungas forest and secondary thickets (Souza et al. 2022a).

#### Etymology.

From ‘Bolivia’ and *dendron* (Greek = tree), named as such for the endemicity of the genus to the country Bolivia.

#### Human uses.

Unknown.

#### Notes.

The type species of *Boliviadendron* was first described as *Leucochloronbolivianum* C.E. Hughes & Atahuachi, but phylogenetic results of Koenen et al. (2020a), Ringelberg et al. (2022) and Souza et al. (2022a) have shown the non-monophyly of *Leucochloron* with this species placed separate from the rest of the genus and occurring instead within the Albizia clade of Koenen et al. (2020a). These results were unexpected because, in particular, the presence of perulate resting buds and a minute wing-like rim on the seeds of *B.bolivianum* suggested close affinity with the four species originally placed in *Leucochloron* by Barneby and Grimes (1996) and only a few morphological characters (weakly) separate *Boliviadendron* from *Leucochloron* (Souza et al. 2022a). One of these features, however, the strongly asymmetrical leaflet base and 1–2 (3) primary veins of the leaflets are very similar to several (microphyllidinous) species of *Albizia* and *Enterolobium*, in line with its phylogenetic placement in the Albizia clade (Koenen et al. 2020a; Ringelberg et al. 2022; Souza et al. 2022a). Without fruits, *Boliviadendron* is morphologically similar to, and easily confused with *Enterolobium*, but can be separated from that genus by its sessile as opposed to pedicellate flowers.

#### Taxonomic references.

Hughes and Atahuachi (2006); Souza et al. (2022a).

### ﻿ Enterolobium

Taxon classificationPlantaeFabalesFabaceae

Mart., Flora 20 (2 Beibl.): 116. 1837.

**Figure 263.**
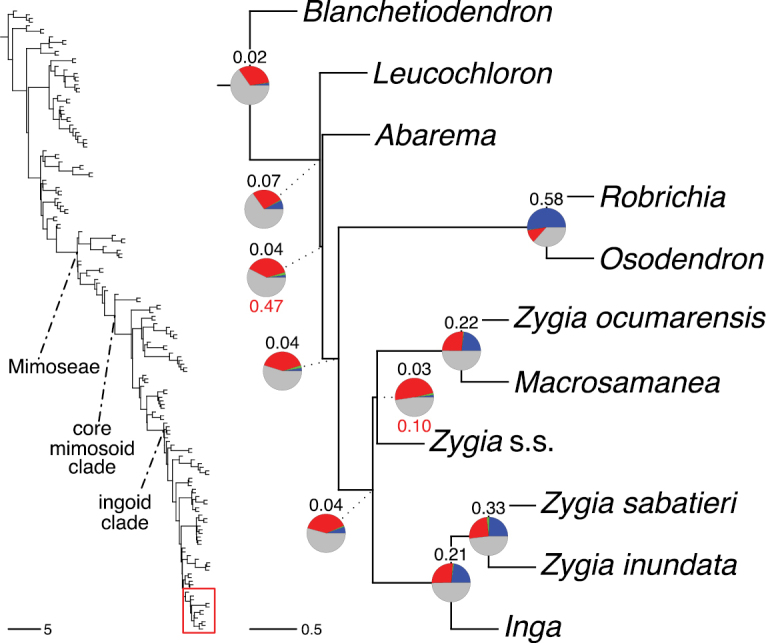
Distribution of *Enterolobium* based on quality-controlled digitised herbarium records. See Suppl. material 1 for the source of occurrence data.

Figs 257 , 258 , 259 , 260 , 261 , 263

#### Type.

*Enterolobiumtimbouva* Mart.

#### Description.

Medium to large trees to 40 m with large rounded spreading crown, unarmed, bark dark grey or brown, smooth or with lenticels. **Stipules** absent or small and mostly caducous. **Leaves** bipinnate, petiole canaliculate or angular, rachis with a pallid indumentum, petiolar nectaries present, additional nectaries sometimes on the leaf rachis and rachillae, usually sessile, globose, transversely elliptic, elliptical-oval to patelliform, paraphyllidia present or absent; pinnae 4–14 pairs; leaflets 4–20 (30) pairs per pinna, symmetrical or asymmetrical, oblong to linear-falcate, both sides glabrous or pilose, venation conspicuous on both sides, palmate, palmate-pinnate, rarely dimidiate-palmate. **Inflorescences** pedunculate globose capitula, in fascicles or aggregated into pseudoracemes, homomorphic or heteromorphic. **Flowers** 5-merous, white, sessile to pedicellate, calyx and corolla tubular or campanulate, petals and sepals outside with a pallid, puberulent, rarely sericeous, strigose-pilose indumentum or glabrous; stamens 20–68 per flower, monadelphous, staminal tube included or exserted, intrastaminal disc usually absent, anthers dorsifixed, longitudinal; pollen shed in 16- or 32-celled flat disk-shaped polyads; ovary at anthesis glabrous, pubescent after fertilization. **Fruits** reniform-auriculiform or annular, rarely contorted, thick coriaceous with a septate papery endocarp, an either dry-mealy or resinous-pulpy mesocarp, and a glabrous or pilose thin exocarp with smooth surface. **Seeds** uniseriate or biseriate, ovoid, ellipsoid or obovoid, pleurogram present or absent, fissure line present or absent.

#### Chromosome number.

2*n* = 26 (Rice et al. 2015).

#### Included species and geographic distribution.

Eight species, from southern Mexico and the Greater Antilles to northern Argentina (Mesquita 1990; Lewis and Rico Arce 2005; Souza et al. 2022b), with Venezuela and Brazil the centres of diversity (Fig. 263).

#### Ecology.

Lowland and submontane tropical rainforests in Amazonia (especially Venezuela and Brazil) and the Atlantic forest of Brazil, in semi-deciduous and seasonally dry tropical forests from southern Mexico and the Greater Antilles to northern Colombia and in southern Brazil, northern Argentina, Bolivia, Paraguay and Uruguay, as well as in the Brazilian Caatinga and Cerrado biomes.

#### Etymology.

From Greek, *entero* (= intestine) and *lobion* (= pod), referring to either the shape or contents of the fruit.

#### Human uses.

The timber of several species is used for high quality furniture, cabinet work, joinery, panelling, veneers and water resistant construction. Also often planted as shade trees, ornamentals or for pasture improvement and livestock fodder (although the fruits of some species are toxic to cattle). Further uses include for fibre (paper), gum extraction and the bark is used as a soap substitute and medicinally (Lewis and Rico Arce 2005).

#### Notes.

*Enterolobium* was described by Martius (1837), based on the type species *E.timbouva*. Until recently, it was considered a genus with 11 species in two sections: sect. Enterolobium with eight species and sect. Robrichia Barneby & J.W. Grimes with three species. However, following phylogenetic results of Souza et al. (2022b) (confirmed by Ringelberg et al. 2022), the latter section was elevated to genus level by Souza et al. (2022b). That genus, *Robrichia* (Barneby & J.W. Grimes) A.R.M. Luz & E.R. Souza, is sister to the recently described *Osodendron* (Koenen 2022b) in the Inga clade, while section Enterolobium is the sister-clade of *Albizia* s.s. (Koenen et al. 2020a; Ringelberg et al. 2022). The fruits of *Robrichia* are similar to those of *Enterolobium* but are typically helicoid or contorted while those of *Enterolobium* are usually reniform-auriculiform or annular (Fig. 260I, J), and only rarely contorted. *Robrichia* also differs from *Enterolobium* in its ferruginous rather than pallid indumentum, more numerous pairs of pinnae and leaflets, flowers with more numerous stamens, and a tomentose rather than glabrous ovary (Souza et al. 2022b).

#### Taxonomic references.

Barneby and Grimes (1996); Mesquita (1990); Souza et al. (2022a).

## ﻿﻿37. Inga clade

João Iganci^20,25^, Ethiéne Guerra^20^, Marli Pires Morim^8^, Ana Carla da Silva Oliveira^2^, Andrés Fonseca-Cortés^2^, Carolina Lima Ribeiro^2^, Felipe da Silva Santos^2^, Filipe Gomes Oliveira^2^, Julia Ferm^15^, Luciano Paganucci de Queiroz^2^

Citation: Iganci J, Guerra E, Morim MP, Oliveira ACS, Fonseca-Cortés A, Ribeiro CL, Santos FS, Oliveira FG, Ferm J, Queiroz LP (2024) 37. Inga clade. In: Bruneau A, Queiroz LP, Ringelberg JJ (Eds) Advances in Legume Systematics 14. Classification of Caesalpinioideae. Part 2: Higher-level classification. PhytoKeys 240: 466–486. https://doi.org/10.3897/phytokeys.240.101716

**Inga clade**

Figs 264–276

**Included genera (8).***Abarema* Pittier (2 species), *Blanchetiodendron* Barneby & J.W. Grimes (1), *Inga* Mill. (ca. 300), *Leucochloron* Barneby & J.W. Grimes (4), *Macrosamanea* Britton & Rose ex Britton & Killip (12), *Osodendron* E.J.M. Koenen (3), *Robrichia* (Barneby & J.W. Grimes) A.R.M. Luz & E.R. Souza (3), *Zygia* P. Browne (ca. 60).

**Description.** Unarmed trees or shrubs, rarely with spine-like projections (some *Osodendron*); axillary perulate resting buds absent or present and then with imbricate and striate perules. **Stipules** mostly caducous. **Leaves** bipinnate or paripinnate, with glands on the petiole and/or leaf rachis and then between the pinnae or leaflets insertion. **Inflorescences** capitate, umbelliform, spicate or racemose, isolated or clustered in axillary fascicles or terminal panicles. **Flowers** mostly 5-merous; calyx gamosepalous; corolla gamopetalous; stamens basally fused in a staminal tube, exserted or included in the corolla; pollen in 16, 18, 20, 24, 32 (35)-celled polyads; gynoecium 1 or pluricarpellate. **Fruits** indehiscent, and then straight or encircled, or passively dehiscent through one and both margins and then with mostly papery or chartaceous valves, rarely lomentiform and breaking up in 1-seeded articles (*Osodendron*). **Seeds** flattened and discoid, ovoid, obovoid, oblong, elliptic to orbicular, transverse, narrowly winged in *Blanchetiodendron* and *Leucochloron*, slightly compressed and bicoloured with testa partially translucent, showing the dark bluish cotyledons in *Abarema*, slightly compressed or terete in cross section, fleshy, thin-walled, the testa developing a thick white sugary sarcotesta in *Inga*; pleurogram U-shaped, O-shaped or absent.

**Distribution.** New World and Africa. The clade is especially diverse in American wet forests, mostly from northern South America in the Amazon basin, extending northward to the Guiana shield and to Mexico and the Antilles, and eastern South America extending to northern Argentina, Paraguay, Uruguay and Bolivia. Also frequent in the riparian forests across the Neotropics. *Blanchetiodendron* and *Leucochloron* are more characteristic of seasonally dry forests and woodlands of eastern and central Brazil. *Osodendron* occurs disjunctly in West and Central Africa, mostly in wet forests.

**Figure 264.**
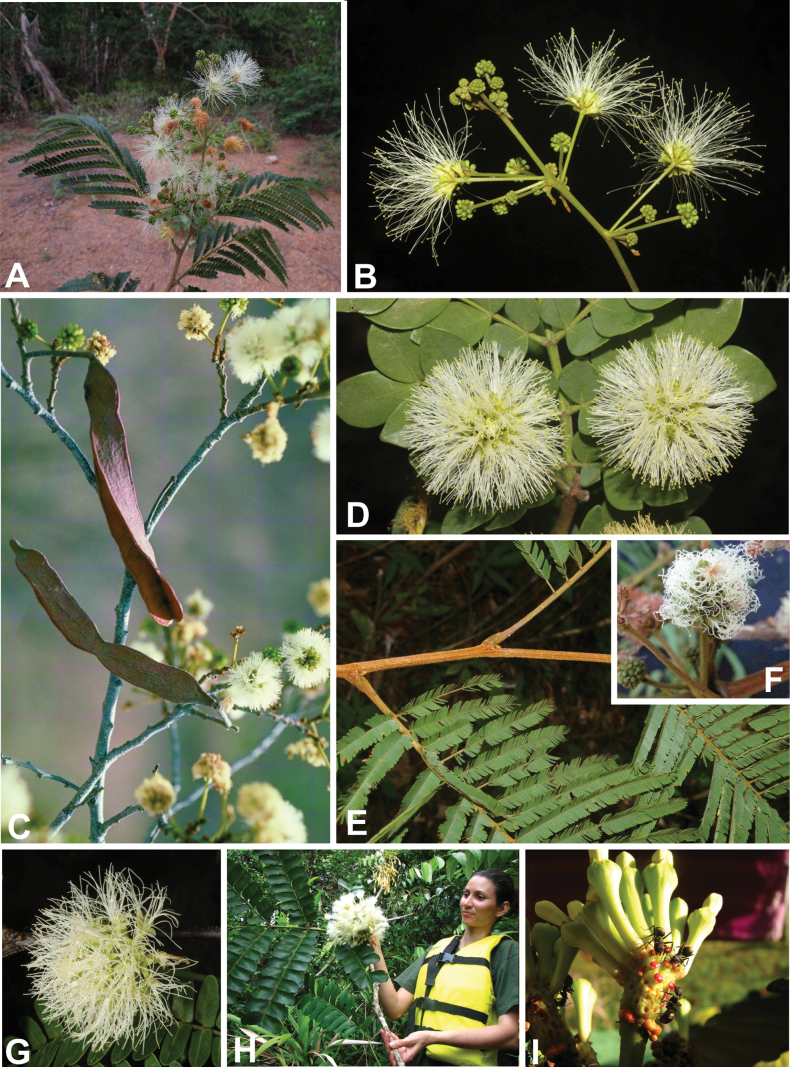
Generic relationships in the Inga clade (tribe Mimoseae). For description of phylogeny and support values, see Fig. 6 caption (page 63).

**Notes.** The Inga clade brings together a heterogeneous set of ingoid legume genera that were placed in disparate groups in the taxonomic system of Barneby and Grimes (1996). This clade was revealed only in recent phylogenomic studies that include dense sampling of mimosoid legumes (Koenen et al. 2020a; Ringelberg et al. 2022). Subtended by a grade of species-poor genera of drier habitats (*Blanchetiodendron* and *Leucochloron*), the clade includes a big radiation in tropical wet forests, particularly in the American species rich genera *Inga* (ca. 300 species) and *Zygia* (ca. 60 species), as well as a highly supported transatlantic clade composed of the New World genus *Robrichia* and the African genus *Osodendron* (Fig. 264).

**Clade-based definition.** The most inclusive crown clade containing *Blanchetiodendronblanchetii* (Benth.) Barneby & J.W. Grimes and *Ingavera* Willd., but not *Albiziaretusa* Benth., *Samaneasaman* (Jacq.) Merr. or *Punjubaracemiflora* (Donn. Sm.) Britton & Rose (Fig. 264).

The clade is morphologically diverse in several aspects and presents some traits rarely found in tribe Mimoseae, such as paripinnate leaves [the entire genus *Inga* and *Zygiainundata* (Ducke) H.C. Lima ex Barneby & J.W. Grimes] and a polycarpellate gynoecium (some species of *Inga* and *Macrosamanea*). Inflorescence units are compact capitate or umbelliform in most genera but spicate or racemose inflorescences are also common. Fruits are very diverse and sometimes diagnostic for particular genera. Indehiscent legumes with a fleshy tissue (sarcotesta) around the seed are characteristic of *Inga*. *Robrichia* also presents indehiscent fruits but they have an encircled body like an ear. Lomentiform fruits breaking up into one-seeded articles are found in *Osodendron*. The remaining genera have dehiscent fruits opening through one or both margins.

Generic taxonomy of the Inga clade has been shaken by recent phylogenetic studies of the mimosoid legumes that demonstrated the rampant non-monophyly of numerous genera (Iganci et al. 2016; Koenen et al. 2020a; Ringelberg et al. 2022). The genera *Robrichia* and *Osodendron* were recently segregated from *Enterolobium* Mart. (Souza et al. 2022b) and *Albizia* Durazz. (Koenen 2022b), respectively. The circumscription of *Abarema* was dramatically reduced to two species after the exclusion of *Jupunba* Britton & Rose and *Punjuba* Britton & Rose (Jupunba clade; Iganci et al. 2016; Soares MVB et al. 2022; Guerra et al. 2023). As currently circumscribed, *Zygia* remains the last non monophyletic genus of the Inga clade in need of taxonomic changes (see genus notes).

### ﻿ Blanchetiodendron

Taxon classificationPlantaeFabalesFabaceae

Barneby & J.W. Grimes, Mem. New York. Bot. Gard. 74(1): 127. 1996.

**Figure 265.**
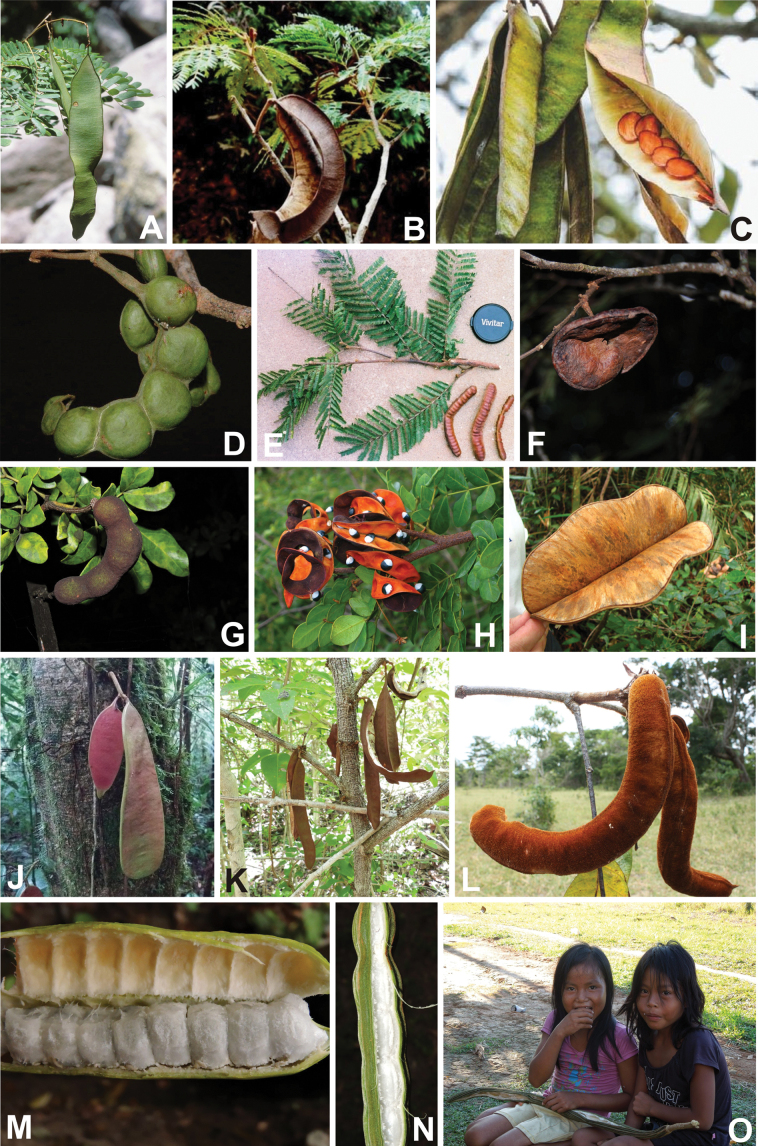
Foliage, inflorescence and fruits in Inga clade **A, B***Blanchetiodendronblanchetii* (Benth.) Barneby & J.W. Grimes **A** flowering branch showing the capitate inflorescences clustered in efoliate pseudoracemes **B** detail of the heteromorphic capitate inflorescence **C***Leucochloronlimae* Barneby & J.W. Grimes leafless branch showing homomorphic capitate inflorescences and follicle fruits **D***Abaremadiamantina* E. Guerra, Iganci & M.P. Morim with axillary homomorphic capitate inflorescences **E, F***Robrichiaschomburgkii* (Benth.) A.R.M. Luz & E.R. Souza **E** young branch with the characteristic rusty pubescent indumentum **F** detail of a homomorphic capitate inflorescences **G***Osodendronaltissimum* (Hook. f.) E.J.M. Koenen detail of a homomorphic capitate inflorescence **H, I***Macrosamaneaamplissima* (Ducke) Barneby & J.W.Grimes **H** flowering branch with a homomorphic capitate inflorescence **I** bracts with showy extrafloral nectaries and visiting ants. Photo credit: **A, B, D** D Cardoso **C** GP Lewis **E** G Perez Huertas **F** F Oviedo-Brenes **G** E Bidaut **H, I** J Iganci.

**Figure 266.**
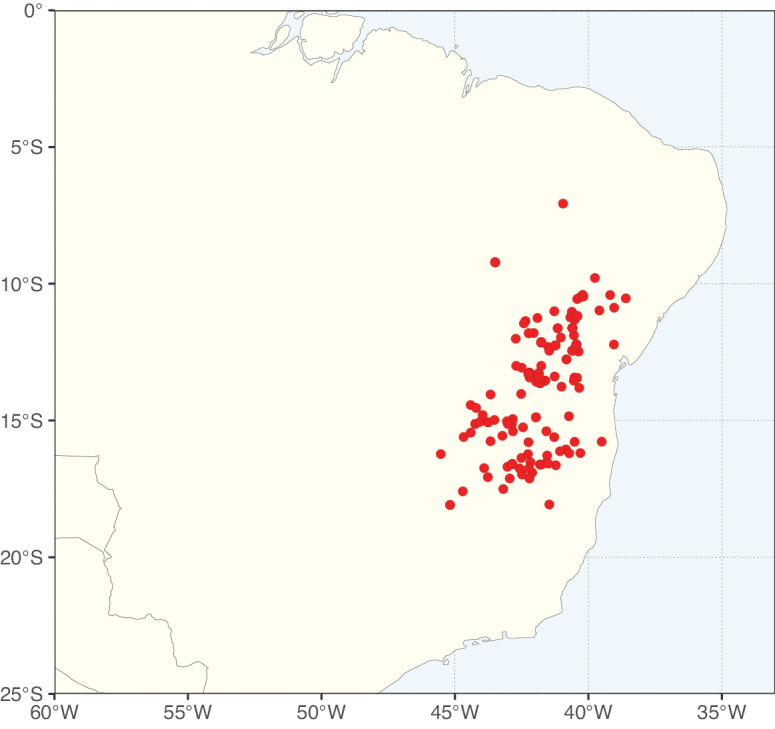
Fruit diversity in Inga clade **A** young pod of *Blanchetiodendronblanchetii* (Benth.) Barneby & J.W. Grimes **B, C***Leucochloronincuriale* (Vell.) Barneby & J.W. Grimes **B** dehisced follicular fruits exposing the seeds (**C**) **D** lomentiform fruit of *Osodendronaltissimum* (Hook. f.) E.J.M. Koenen **E** indehiscent and internally septate pod of *Osodendronleptophyllum* (Harms) E.J.M. Koenen **F** auriculiform indehiscent pod of *Robrichiaschomburgkii* (Benth.) A.R.M. Luz & E.R. Souza **G** young fruit of *Abaremacochliacarpos* (Gomes) Barneby & J.W. Grimes **H** dehisced twisted pod of *Abaremadiamantina* E. Guerra, Iganci & M.P. Morim exposing the reddish endocarp and bicoloured seeds **I** dehisced follicle of *Macrosamaneaamplissima* (Ducke) Barneby & J.W.Grimes **J** Young fruits of *Zygiabrenesii* (Standl.) L. Rico **K** dehisced pods of *Zygiacognata* (Schltdl.) Britton & Rose **L–O** Indehiscent pods of *Inga*, with fleshy and sweety sarcotesta around the seeds **L***Ingagrazielae* (Vinha) T.D. Penn. **M***Ingacapitata* Desv. **N***Ingaingoides* (Rich.) Willd. **O***Ingaedulis* Mart. Photo credits **A, H** LP Queiroz **B** NA Escobar **C** J Vieira **D** D Harris **E** P Latham, www.africanplants.senckenberg.de**F** LCampbell **G, M, N** RT Queiroz http://rubens-plantasdobrasil.blogspot.com/**I** J Iganci **J** F Chinchila Romero **K** S de Jesus Calva **L, O** D Cardoso.

**Figure 267.**
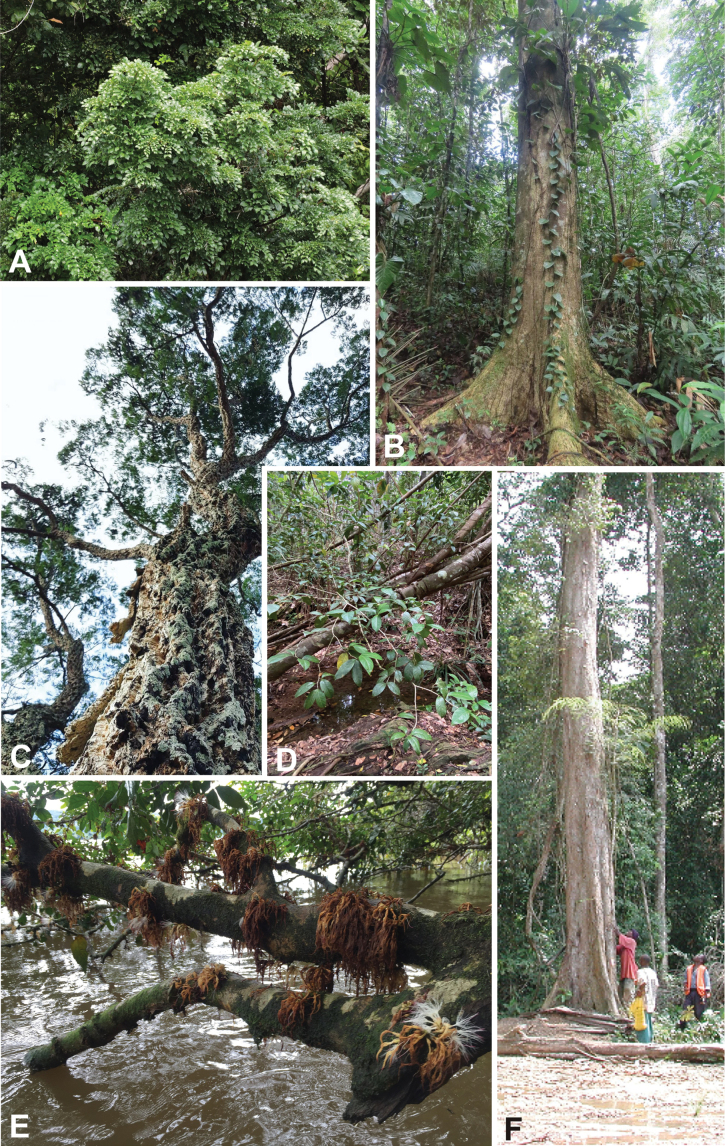
Distribution of *Blanchetiodendron* based on quality-controlled digitised herbarium records. See Suppl. material 1 for the source of occurrence data.

Figs 265 , 266 , 267

#### Type.

*Blanchetiodendronblanchetii* (Benth.) Barneby & J.W. Grimes [≡ *Enterolobiumblanchetii* Benth.]

#### Description.

Unarmed trees; perulate buds at some leaf axils, the perules imbricate and striate. **Stipules** absent. **Leaves** bipinnate; petiolar nectary near or above mid-petiole, sessile, cupular or shallow-cupular; pinnae 2–4 pairs; leaflets 8–15 pairs, (sub)opposite, variable in size and shape. **Inflorescence units** capitate or umbelliform, grouped in axillary pseudoracemes or terminal panicles (Fig. 265A, B). **Flowers** dimorphic, the peripheral ones slenderly pedicellate, the terminal one sessile or shortly pedicellate; sepals 5, gamosepalous; petals 5, gamopetalous; stamens ca. 26–38 in peripheral flowers and 30–46 in terminal flowers (Barneby and Grimes 1996), filaments basally fused into a tube, stemonozone present, anthers rimose; pollen in 16-celled polyads; ovary glabrous. **Fruit** a plano-compressed, oblong, legume, inertly dehiscent through both margins, valves papery (Fig. 266A). **Seeds** elliptic to suborbicular, narrowly winged, pleurogram absent.

#### Chromosome number.

Unknown.

#### Included species and geographic distribution.

Monospecific (*B.blanchetii*), endemic to north-eastern Brazil mostly from central Bahia state, extending to northern Minas Gerais and southern Piauí (Fig. 267).

#### Ecology.

Seasonally deciduous and semi-deciduous forests and woodlands from the Caatinga and Atlantic Forest phytogeographical domains, from 450 to 1000 m elevation (Queiroz 2009).

#### Etymology.

The genus is named after the Swiss naturalist Jacques Samuel Blanchet, and *dendron* (Greek = tree).

#### Human uses.

Unknown.

#### Notes.

The genus *Blanchetiodendron* was described as a monotypic genus by Barneby and Grimes (1996) to accommodate *B.blanchetii*, previously included in *Enterolobium*, *Pithecellobium* Mart. (Bentham 1876) or *Albizia* (Lewis 1987).

Barneby and Grimes (1996) included *Blanchetiodendron* (together with *Leucochloron* and *Chloroleucon* (Benth.) Britton & Rose) in the informal Chloroleucon alliance because of the presence of axillary perulate resting buds, but this group has since been shown to be polyphyletic (Koenen et al. 2020a). *Blanchetiodendron* is sister to the rest of the Inga clade and can be diagnosed by the heteromorphic capitate umbelliform inflorescences grouped in axillary efoliate pseudoracemes or in terminal panicles.

#### Taxonomic references.

Barneby and Grimes (1996); Morim (2020); Queiroz (2009).

### ﻿ Leucochloron

Taxon classificationPlantaeFabalesFabaceae

Barneby & J.W. Grimes, Mem. New York. Bot. Gard. 74(1): 130. 1996.

**Figure 268.**
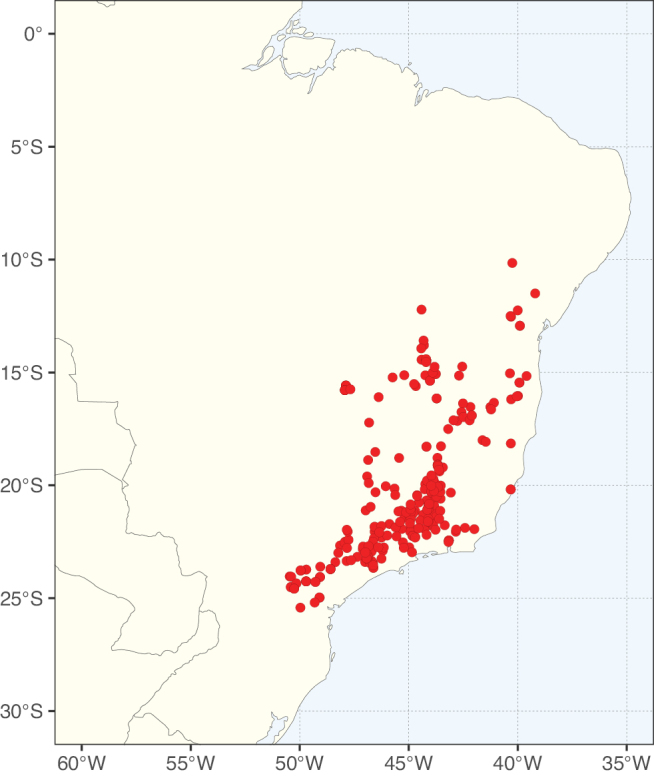
Habitat and habit diversity in Inga clade **A***Abaremacochliacarpos* (Gomes) Barneby & J.W. Grimes, arborescent shrub in sandy coastal scrub **B***Robrichiaschomburgkii* (Benth.) A.R.M. Luz & E.R. Souza, buttressed emergent tree in rainforests **C***Leucochloronincuriale* (Vell.) Barneby & J.W. Grimes, tree with thick corky bark in semi-deciduous forests **D***Ingabifoliolata* D.B.O.S. Cardoso & Amorim, scandent treelet of rainforest understory **E***Zygiajuruana* (Harms) L. Rico treelet of the Amazonian inundated forests (igapó) **F***Osodendrondinklagei* (Harms) E.J.M. Koenen, giant tree from the Guinean forests in West tropical Africa. Photo credits **A** RT Queiroz https://rubens-plantasdobrasil.blogspot.com/**B** G Perez Huertas **C** F Ventura **D, E** D Cardoso **F** W Hawthorne.

**Figure 269.**
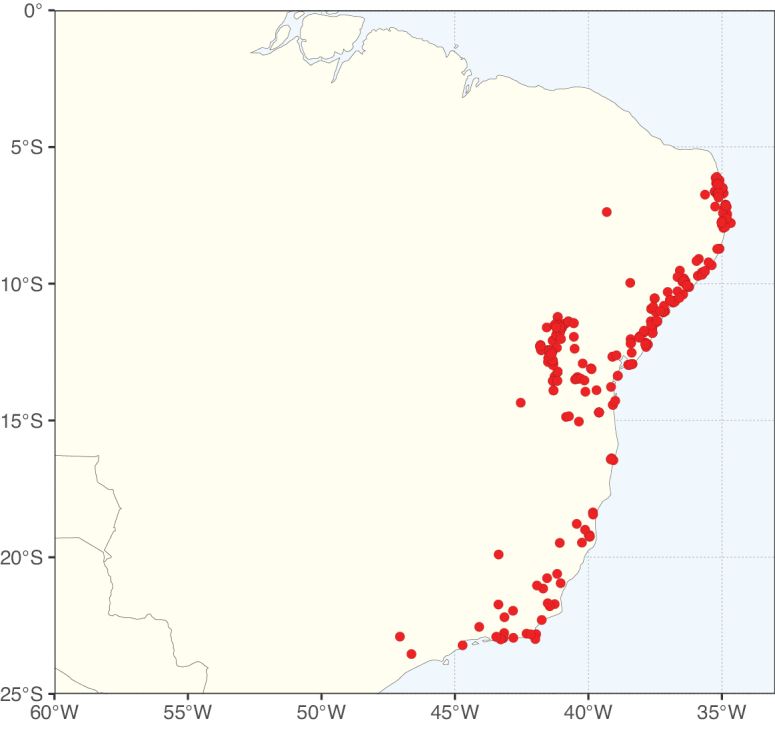
Distribution of *Leucochloron* based on quality-controlled digitised herbarium records. See Suppl. material 1 for the source of occurrence data.

Figs 265 , 266 , 268 , 269

#### Type.

*Leucochloronincuriale* (Vell.) Barneby & J.W. Grimes [≡ *Mimosaincurialis* Vell.]

#### Description.

Unarmed trees (Fig. 268C) with monopodial branches; young branches puberulent or pilosulous; perulate buds often present in leaf axil and at apex of branchlets, scales striate. **Stipules** caducous. **Leaves** bipinnate; petiolar glands sessile, cupular or almost plane, between or close below the first pinna pair, sometimes below mid-petiole; pinnae (1) 2–9 pairs; leaflets 4–27 pairs, with palmate-pinnate or pinnate venation. **Inflorescences** homomorphic, globose or hemispherical capitula, solitary or fasciculate in leaves axils or below the coeval leaves (Fig. 265C). **Flowers** 5-merous, white; calyx gamosepalous, deeply campanulate; corolla gamopetalous, tubular or funnel-shaped; androecium 20–40-merous; pollen in 16, 18, 24 or 32-celled polyads (Souza et al. 2022a); intrastaminal disc absent; ovary sessile. **Fruits** inertly dehiscent through one (follicle) or both margins (legume), broadly linear, straight or slightly decurved, the valves stiffly papery or coriaceous (Figs 265C, 266B, C). **Seeds** discoid, transverse, narrowly winged peripherally, pleurogram absent; endosperm absent.

#### Chromosome number.

Unknown.

#### Included species and geographic distribution.

Four species, probably endemic to Brazil, occurring along eastern Atlantic coastal states (Bahia to Paraná) and inland to planaltine Minas Gerais, and Distrito Federal (Fig. 269).

#### Ecology.

Seasonally dry forests, campo, caatinga, open woodland, bush-islands in campo, and in cerrado-gallery ecotone and at elevations of 710–1500 m. *Leucochloronminarum* (Glaz. & Harms) Barneby & J.W. Grimes and *L.limae* Barneby & J.W. Grimes are endangered species (Almeida et al. 2015; Morim 2020).

#### Etymology.

An anagram of *Chloroleucon*.

#### Human uses.

*Leucochloronincuriale* wood used in luxury furniture, parquet flooring, interior decoration, sheets for plywood coverings, wainscoting; civil construction, such as rafters, frames, slats, floorboards; in rural construction and in external works, such as sleepers, stakes, fence posts, and beams (Campos Filho and Sartorelli 2015).

#### Notes.

The genus *Leucochloron* was proposed by Barneby and Grimes (1996) and is diagnosed by the presence of perulate resting-buds, capitate inflorescences isolated or fasciculate in leaf axils or below the coeval leaves and thin shiny disciform narrowly winged seeds lacking a pleurogram.

Barneby and Grimes (1996) included *Leucochloron* in their informal Chloroleucon alliance that included genera with perulate resting buds. To reinforce its affiliation, they chose to name the genus using an anagram of *Chloroleucon*. Phylogenetic studies have shown the polyphyly of the Chloroleucon alliance (Almeida 2014), with the genera occurring dispersed across the ingoid clade, and the more recent phylogenomic analyses support the inclusion of *Leucochloron* in the Inga clade (Koenen et al. 2020a; Ringelberg et al. 2022).

Hughes and Atahuachi (2006) described *Leucochloronbolivianum* C.E. Hughes & Atahuachi from the mid-elevation eastern Andean slopes in Bolivia. Although morphologically fitting the diagnostic circumscription of *Leucochloron*, molecular data showed that this species does not group with the remaining species of *Leucochloron* (Almeida 2014; Koenen et al. 2020a; Ringelberg et al. 2022) and provided support for the recognition of the new genus *Boliviadendron* E.R. Souza & C.E. Hughes (Souza et al. 2022a) included in the Albizia clade (page 451).

Lewis (1987) commented about a possible new species related to *L.incuriale* from a fruiting specimen from western Bahia state, Brazil (*G.A. Black 18013*, IAN). A second potentially new species from the Guárico state, Venezuela (*H.M. Curran 703*, NY) was highlighted by Barneby and Grimes (1996). Both need more complete sampling and molecular data to assert their taxonomic placement.

#### Taxonomic references.

Almeida et al. (2015); Barneby and Grimes (1996); Souza et al. (2022a).

### ﻿ Abarema

Taxon classificationPlantaeFabalesFabaceae

Pittier, Arb. Arbust. Orden Legum.: 56. 1927.

**Figure 270.**
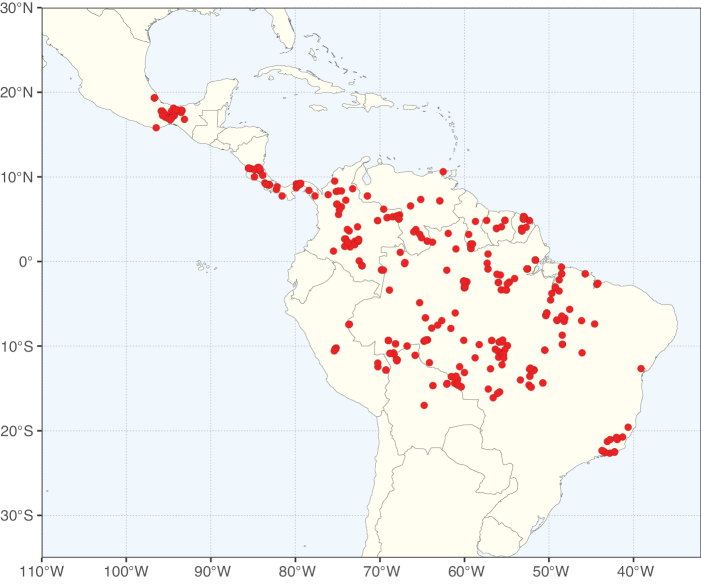
Distribution of *Abarema* based on quality-controlled digitised herbarium records. See Suppl. material 1 for the source of occurrence data.

Figs 265 , 266 , 268 , 270

Pithecellobium sect. Abaremotemon Benth., London J. Bot. 3: 203. 1844. Lectotype (designated by Britton & Killip, 1936): Pithecellobiumauaremotemo Mart. [= Abaremacochliacarpos (Gomes) Barneby & J.W. Grimes]

#### Type.

*Abaremacochliacarpos* (Gomes) Barneby & J.W. Grimes [≡ *Mimosacochliocarpos* Gomes]

#### Description.

Unarmed shrubs and trees (Fig. 268A); bark reddish, branches pubescent, pulverulent, with conspicuous lenticels. **Stipules** triangular, greenish brown, usually caducous. **Leaves** bipinnate, alternate; extrafloral nectaries sessile, patelliform, between all pairs of pinnae; petiole, rachis, and pinnae with a ferruginous pubescent, pulverulent indumentum, canaliculate; pinnae 1–5 pairs, opposite; leaflets 2–6 pairs, sessile, opposite, chartaceous or membranous. **Inflorescence units** axillary, homomorphic, capitate (Fig. 265D), peduncle with a ferruginous pubescent, pulverulent indumentum. **Flowers** sessile, 5-merous, green; calyx gamosepalous, campanulate; corolla gamopetalous, campanulate or infundibuliform; androecium with more than 10 stamens, exserted from the corolla, filaments partially fused into a tube, anthers rimose; pollen in 16, 24 or 32-celled polyads; ovary subsessile. **Fruit** a legume, dehiscing through both margins, spiralled, epicarp brown, with a ferruginous pulverulent indumentum, endocarp brown-orange (Fig. 266G, H). **Seeds** obovoid, bicoloured white and dark bluish (Fig. 266H), with U-shaped pleurogram in the upper half of the seed.

#### Chromosome number.

Unknown.

#### Included species and geographic distribution.

Two species, *A.cochliacarpos* and *A.diamantina* E. Guerra, Iganci & M.P. Morim, endemic to Brazil, along the Atlantic Forest, in coastal south-east and north-east Brazil, from São Paulo to Ceará states. In north-east Brazil the genus also occurs in the Caatinga, in inland Bahia state (Fig. 270).

#### Ecology.

Sandy soil in Caatinga (Bahia state) with caatinga and cerrado vegetation elements (carrasco), ombrophilous forest, semi-deciduous forest and coastal scrub (restinga) (Guerra et al. 2023).

#### Etymology.

From Tupi-Guarani “abaré” – priest; “motimbora” – make smoke (Chiaradia 2008).

#### Human uses.

*Abaremacochliacarpos*, usually known as “barbatimão” in north-east Brazil, is rich in phenolic secondary compounds. The bark is used in infusions to treat ulcers, sores, gastritis, inflammation, leukorrhea, and vaginal discharge (Santos et al. 2022). Tannins extracted from the bark are used as an astringent to expedite wound healing, and the high-quality, light-colored wood of the tree is ground into a powder and applied to ulcers. Its ashes are used in the manufacturing of soap.

#### Notes.

The first mention of *Abarema* is attributed to Pisonis (1658) where he described ‘*De Abaremo temo arbore, ejusque facultatibus*’, a pre-Linnean name not considered as validly published, referring to *A.cochliacarpus*. The genus *Abarema* was established by Pittier (1927), based on Pithecellobiumsect.Abaremotemon (Bentham 1844). Barneby and Grimes (1996) defined the Abarema alliance as comprising the genera *Hydrochorea* Barneby & J.W. Grimes, *Balizia* Barneby & J.W. Grimes and *Abarema*. A molecular phylogenetic study of the *Abarema* alliance demonstrated that the genus *Abarema* as circumscribed by Barneby and Grimes (1996) was polyphyletic (Iganci et al. 2016), with the type species, *A.cochliacarpos* (Gomes) Barneby & J.W. Grimes, placed separately from other *Abarema* species, and the remaining species of *Abarema* s.l. grouping in two clades, separated by *Hydrochorea* Barneby & J.W. Grimes (Soares MVB et al. 2022).

*Abaremacochliacarpos* is widely distributed throughout the geographic range of the genus, and has a high degree of morphological variation across its distribution (Guerra et al. 2019). *Abaremadiamantina* has a restricted distribution. The two species can be distinguished by the flowers with staminal tube exserted from the corolla in *A.diamantina* (vs. inserted in *A.cochliacarpos*) and the seeds with a foveolate testa in *A.diamantina* (vs. smooth in *A.cochliacarpos*) (Guerra et al. 2016).

#### Taxonomic references.

Barneby and Grimes (1996); Guerra et al. (2019, 2023); Iganci et al. (2016); Soares et al. (2022).

### ﻿ Robrichia

Taxon classificationPlantaeFabalesFabaceae

(Barneby & J.W. Grimes) A.R.M. Luz & E.R. Souza, Syst. Bot. 47: 274. 2022.

**Figure 271.**
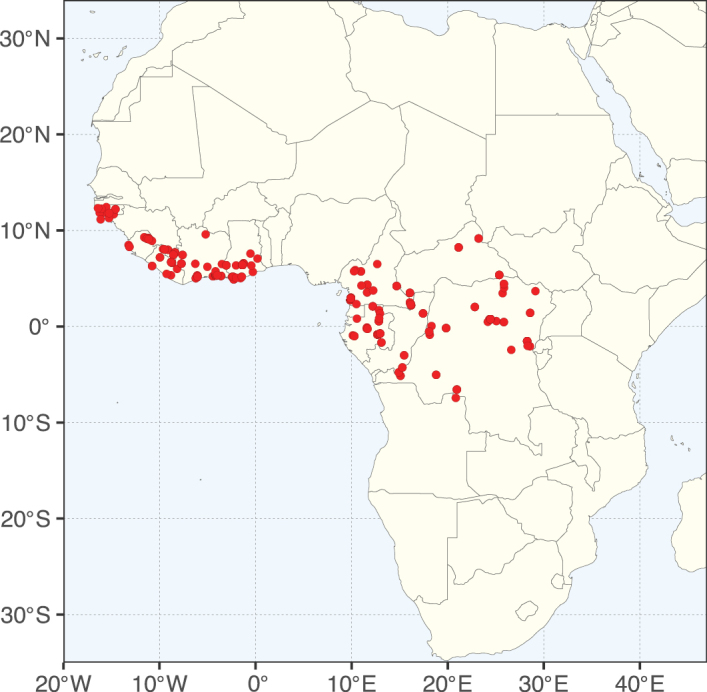
Distribution of *Robrichia* based on quality-controlled digitised herbarium records. See Suppl. material 1 for the source of occurrence data.

Figs 265 , 266 , 268 , 271

Enterolobium sect. Robrichia Barneby & J.W. Grimes, Mem. New York Bot. Gard. 74(1): 249. 1996. Type: Enterolobiumschomburgkii (Benth.) Benth. [≡ Pithecellobiumschomburgkii Benth.]

#### Type.

*Robrichiaschomburgkii* (Benth.) A.R.M. Luz & E.R. Souza [≡ *Pithecellobiumschomburgkii* Benth.]

#### Description.

Trees 8–50 m high (Fig. 268B); bark rugose, with lenticels, sometimes exfoliating in irregular layers. **Stipules** obovate to lanceolate-falcate, sericeous, fulvous, caducous. **Leaves** bipinnate, pubescent, fulvous (Fig. 265E); leaf glands near the base of the petiole and sometimes between the pairs of pinnae; paraphyllidia present or absent; pinnae 10–30 pairs; leaflets 40–80 pairs, linear, asymmetrical. **Inflorescence units** homomorphic or heteromorphic capitula (Fig. 265F), 2–5-fascicled in leaf axils or amongst the branches below the coeval leaves, in the heteromorphic ones the terminal flower is larger than the others, and the staminal tube is exerted. **Flowers** 5-merous; calyx gamosepalous, campanulate; corolla gamopetalous, campanulate, lobes triangular, fulvous villose; stamens numerous, the filaments basally united into a tube; pollen in 16, 28-celled polyads; ovary sessile, ovoid, fulvous villose. **Fruits** indehiscent, reniform auriculiform, sometimes twisted, laterally compressed, puberulent (Fig. 266F). **Seeds** ovoid, ellipsoid or obovoid; pleurogram present.

#### Chromosome number.

Unknown.

#### Included species and geographic distribution.

Three species known to date, *R.glaziovii* (Benth.) A.R.M. Luz & E.R. Souza, *R.oldemanii* (Barneby & J.W. Grimes) A.R.M. Luz & E.R. Souza and *R.schomburgkii* (Souza et al. 2022b), from the south of Mexico to Bolivia, to 1100 m elevation. *Robrichiaglaziovii* is endemic to eastern Brazil (Morim et al. 2020), *R.oldemanii* occurs in French Guiana and northern Brazil (Fernandes 2023) and *R.schomburgkii* is present from the south of Mexico to Bolivia (Fig. 271).

#### Ecology.

*Robrichia* species are large trees extending to the rainforest canopy. Dulmen (2001) reported “small bees” as the pollinators of *R.schomburgkii*.

#### Etymology.

The name of this genus is composed of the initial parts of two names “Rob” and “Rich”, which Barneby and Grimes (1996) chose to commemorate the brothers Robert and Richard Schomburgk, German naturalists who sampled the Guyana flora in the 19^th^ century.

#### Human uses.

In Brazilian Amazonia, *Robrichiaschomburgkii* is used for civil and naval construction (Le Cointe 1947); in Oaxaca, Mexico this species is also used in teas to relieve stomach ache (Mesquita 1990).

#### Notes.

Barneby and Grimes (1996) described Enterolobiumsect.Robrichia to include three species of *Enterolobium* with similar morphology, *E.glaziovii*, *E.oldemanii* and *E.schomburgkii*. Molecular phylogenetic analysis with plastid and nuclear markers (Souza et al. 2022b) and phylogenomic studies (Ringelberg et al. 2022) do not support the monophyly of *Enterolobium* and resolved section Robrichia as sister to the tropical African genus *Osodendron*, both nested in the Inga clade (Ringelberg et al. 2022).

#### Taxonomic references.

Barneby and Grimes (1996); Mesquita (1990); Morim et al. (2020); Souza et al. (2022b).

### ﻿ Osodendron

Taxon classificationPlantaeFabalesFabaceae

E.J.M. Koenen, PhytoKeys 205: 456. 2022.

**Figure 272.**
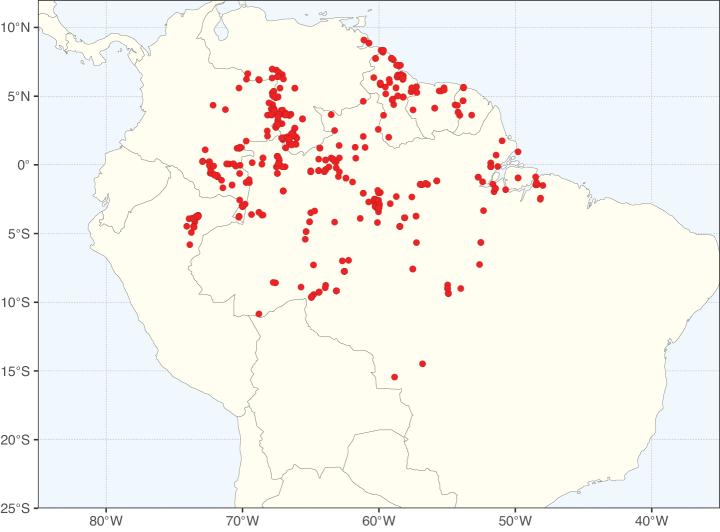
Distribution of *Osodendron* based on quality-controlled digitised herbarium records. See Suppl. material 1 for the source of occurrence data.

Figs 265 , 266 , 268 , 272

#### Type.

*Osodendronaltissimum* (Hook. f.) E.J.M. Koenen [≡ *Albiziaaltissima* Hook. f.]

#### Description.

Trees, rarely shrubs, 5–35 m tall (Fig. 268F), unarmed or sometimes with spine-like projections; indumentum usually dense ferruginous pubescent. **Stipules** deltoid, elliptically oblong, lanceolate to linear, sometimes asymmetrical, caducous leaving scars. **Leaves** bipinnate; leaf glands usually present; pinnae (3) 5–30 (34) pairs, opposite, sometimes the lowermost pair sub-opposite and usually distinctly shorter than other pairs; leaflets (7) 13–40 (48) pairs. **Inflorescence units** sub-globose dimorphic and axillary capitula (Fig. 265G). **Flowers** 4–5-merous, sessile or shortly stipitate, green, greenish-white to white; calyx gamosepalous; corolla gamopetalous; stamens with 10–25 filaments basally united into a staminal tube, white; pollen in 32-celled polyads; ovary 1–3 mm long, pubescent, puberulent or with a few scattered hairs. **Fruit** indehiscent, tardily breaking up into one-seeded articles or not, straight, slightly curved to coiled (Fig. 266D, E). **Seeds** orbicular to nearly elliptic, with a hard testa; pleurogram U-shaped or O-shaped.

#### Chromosome number.

2*n* = 26 (Cave 1960).

#### Included species and geographic distribution.

Three species, *O.altissimum*, *O.dinklagei* (Harms) E.J.M. Koenen and *O.leptophyllum* (Harms) E.J.M. Koenen, in tropical West and Central Africa, from Senegal and Guinea Bissau to Zambia and Angola (Fig. 272).

#### Ecology.

*Osodendron* is found mainly in tropical wet forests extending to the savanna belt. *Osodendrondinklagei* and *O.leptophyllum* can also be found in swampy areas, forest edges or gallery forest (Koenen 2022b).

#### Etymology.

The word “*oso*” refers to a recipe from African cuisine, mainly from the area of Ghana and Guinea, which uses *O.altissimum* seeds as a base for preparation of a local fermented food (Jolaoso 2014).

#### Human uses.

*Osodendron* is important for food in Africa, where *O.altissimum* seeds are used in cooking and human nutrition (Jolaoso 2014). The leaves of this species also have anti-inflammatory and analgesic properties, confirmed by phytochemistry and animal testing (Aderibigbe et al. 2015).

#### Notes.

The species currently assigned to *Osodendron* were placed in *Cathormion* (Benth.) Hassk. and *Albizia*, but the phylogenomic study of Koenen et al. (2020a) indicated that the type species of *Cathormion* belonged to the Albizia clade. Subsequently, Ringelberg et al. (2022) revealed that the three species assigned to *Osodendron* are more closely related to the genus *Robrichia* than to *Albizia* providing support for the recognition of the new genus *Osodendron* (Koenen 2022b).

*Osodendron* species are restricted to Africa, whereas *Robrichia* species have a Neotropical distribution, and they can be differentiated from *Robrichia* mainly by the shape of their fruits, which are straight, slightly curved, coiled and/or twisted (vs. contorted or “ear-shaped” in *Robrichia*) (Koenen 2022b). The three *Osodendron* species can be distinguished by the number of pinnae, presence of leaf glands and fruit shape and articulation (Koenen 2022b).

#### Taxonomic references.

Jolaoso (2014); Koenen (2022b).

### ﻿ Macrosamanea

Taxon classificationPlantaeFabalesFabaceae

Britton & Rose ex Britton & Killip, Ann. New York Acad. Sci. 35: 131. 1936.

**Figure 273.**
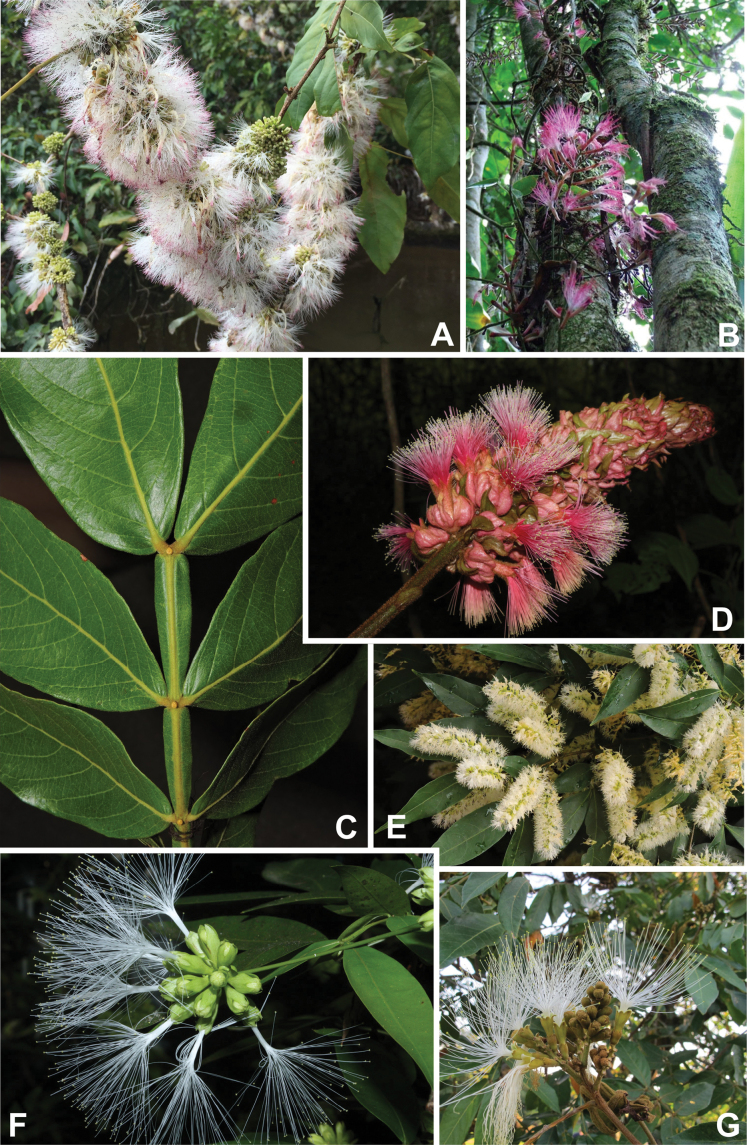
Distribution of *Macrosamanea* based on quality-controlled digitised herbarium records. See Suppl. material 1 for the source of occurrence data.

Figs 265 , 266 , 273

Pithecellobium ser. Coriacea Benth., Trans. Linn. Soc. 30: 589. 1875. Type: Pithecellobiumadiantifolium (Kunth) Benth., nom. illeg. [≡ Macrosamaneadiscolor (Willd.) Britton & Killip]

#### Type.

*Macrosamaneadiscolor* (Humb. & Bonpl. ex Willd.) Britton & Rose ex Britton & Killip. [≡ *Ingadiscolor* Humb. & Bonpl. ex Willd.]

#### Description.

Unarmed shrubs (sometimes semi-scandent), trees or lianas. **Stipules** caducous. **Leaves** bipinnate, extrafloral nectaries absent or present in basal or median region of petiole, sometimes between pairs of pinnae, sessile, elliptic, patelliform, scutiform, round; pinnae 1–17 pairs; leaflets 2–31 pairs, opposite, variable in size and shape. **Inflorescence units** capitate or spicate, congested, lax, or umbelliform, axillary or terminal, rarely cauliflorous (Fig. 265H); bracts with patelliform nectaries (Fig. 265I) or nectaries absent. **Flowers** homomorphic, pedicellate or sessile; 4–5-merous; sepals 4–5, gamosepalous, the calyx green, to pinkish or brown, cylindrical to campanulate; petals 4–5, gamopetalous, the corolla white, greenish-white, rarely pinkish or brown, infundibuliform; stamens 45–248, filaments fused into a tube, stemonozone present or absent, anthers rimose; pollen in 16, 20, 24, 32 (35)-celled polyads; gynoecium 1, rarely 2–7-carpellate. **Fruit** a follicle, dehiscing along one margin only (Fig. 266I), or when mature dehiscing through both margins, the body plano-convex, straight or curved. **Seeds** elliptic, oblong or obovate, pleurogram absent.

#### Chromosome number.

Unknown.

#### Included species and geographic distribution.

Twelve species distributed in northern South America (Brazil, Colombia, Guyana, Peru, Suriname, Venezuela), mostly in Amazonia (Fig. 273).

#### Ecology.

Riparian habitats, and in forests and open areas subject to seasonal flooding in the Amazon basin (savanna or “campinarana”).

#### Etymology.

From Greek, *macro* (= great), and *Samanea* (from the aboriginal samán = rain tree).

#### Human uses.

Unknown.

#### Notes.

*Macrosamanea* was described by Britton and Killip (1936) based on Pithecellobiumsect.Samaneaser.Coriacea Benth. and later included in the Inga alliance by Barneby and Grimes (1996). Molecular analyses have supported the monophyly of *Macrosamanea* (Iganci 2016; Silva 2017; Koenen et al. 2020a; Ringelberg et al. 2022) and morphologically the genus is unique among the genera of Neotropical ingoid legumes in having extrafloral nectaries on the floral bracts (Lewis and Rico Arce 2005) [all except *M.kegelii* (Meisn.) Kleinhoonte and *M.macrocalyx* (Ducke) Barneby & J.W.Grimes (Silva 2017)].

#### Taxonomic references.

Barneby and Grimes (1996); Britton and Killip (1936); Lewis and Rico Arce (2005); Silva (2017, 2020).

### ﻿ Zygia

Taxon classificationPlantaeFabalesFabaceae

P. Browne, Civ. Nat. Hist. Jamaica: 279, t. 22, fig. 3. 1756.

**Figure 274.**
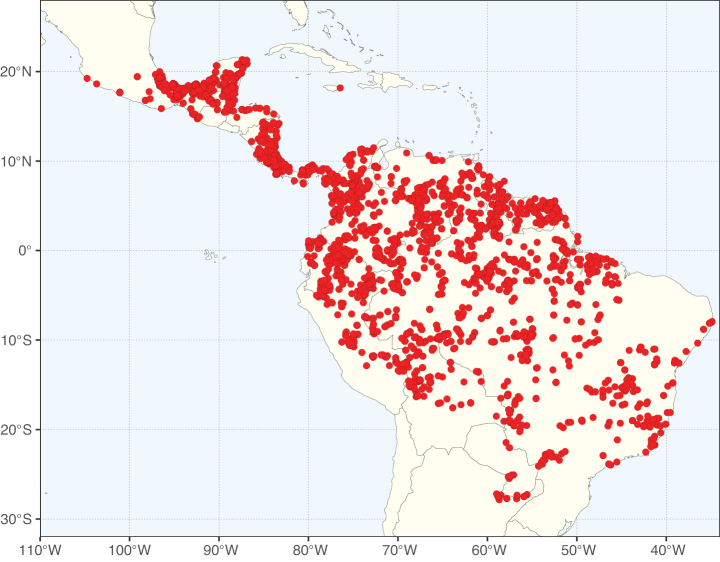
Leaf and inflorescence diversity in *Zygia* and *Inga* (Inga clade) **A***Zygiajuruana* (Harms) L. Rico, dense cauliflorous capitate inflorescences **B***Zygiaselloi* (Benth.) L. Rico with elongate cauliflorous spikes **C** part of a leaf of *Ingaingoiodes* (Rich.) Willd. showing the winged leaf rachis and extrafloral nectaries between leaflet insertions **D** dense pyramidal spike of *Ingagrazielae* (Vinha) T.D. Penn. **E** elongate cylindrical spikes of *Ingamarginata* Willd. **F** pedunculate capitate inflorescence of *Ingacordistipula* Mart. **G** terminal panicle of *Ingavera* Kunth. Photo credits **A, D, F** D Cardoso **B** M Magenta **C, E, G** RT Queiroz https://rubens-plantasdobrasil.blogspot.com/.

**Figure 275.**
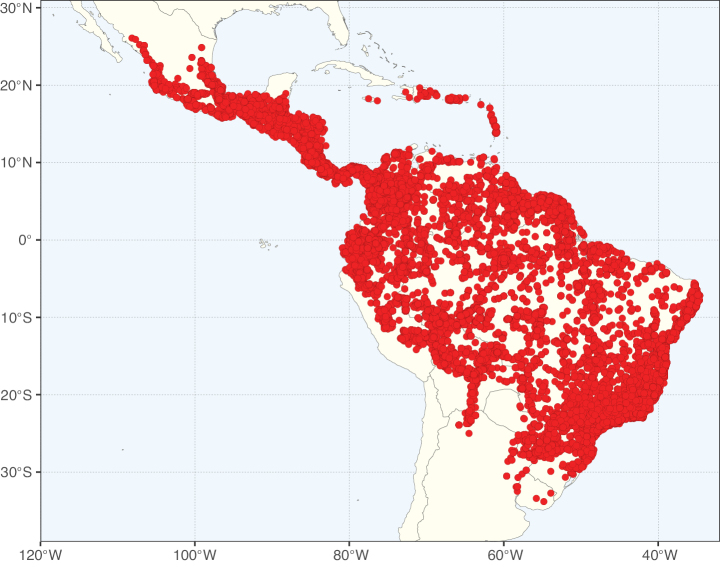
Distribution of *Zygia* based on quality-controlled digitised herbarium records. See Suppl. material 1 for the source of occurrence data.

Figs 266 , 268 , 274 , 275

Pithecellobium sect. Caulanthon Benth., London J. Bot. 3: 197. 1844. Type: Pithecellobiumunifoliolatum Benth. [≡ Zygiaunifoliolata (Benth.) Pittier]
Calliandra sect. Caulanthon (Benth.) Griseb., Fl. Brit. W. I.: 225. 1864. Type: Calliandralatifolia (L.) Griseb. [≡ Zygialatifolia (L.) Fawc. & Rendle]
Marmaroxylon Killip in Record, Trop. Woods 63: 3. 1940. Type: Marmaroxylonracemosum (Ducke) Killip [≡ Pithecellobiumracemosum Ducke (≡ Zygiaracemosa (Ducke) Barneby & J.W. Grimes)]
Pithecellobium sect. Callozygia Barbosa, Caldasia 14: 400. 1986. Type: Pithecellobiumlehmannii Harms [≡ Zygialehmannii (Harms) Britton & Rose]
Zygia sect. Callozygia (Barbosa) L. Rico, Kew Bull. 46: 495. 1991. Type: Zygialehmannii (Harms) Britton & Rose [≡ Pithecellobiumlehmanii Harms]
Zygia sect. Macrophylla L. Rico, Kew Bull. 46: 495. 1991. Type: Zygiamacrophylla (Spruce ex Benth.) L. Rico [≡ Pithecellobiummacrophyllum Spruce ex Benth.]

#### Type.

*Zygialatifolia* (L.) Fawc. & Rendle [≡ *Mimosalatifolia* L.]

#### Description.

Unarmed shrubs or small trees, rarely reaching 20 m height, rarely lianas (Fig. 268E); bark smooth or flaky, whitish or pale grey. **Stipules** small, ribbed, caducous, rarely persistent. **Leaves** bipinnate, rarely paripinnate [*Z.inundata* (Ducke) H.C. Lima ex Barneby & J.W. Grimes] or bifoliolate [*Z.unifoliolata* (Benth.) Pittier], pinnae 1–10 pairs, opposite; leaflets 1–23 pairs per pinna, sessile, opposite to subopposite, pinnately or palmately veined, frequently with the inner leaflet of proximal pair reduced; petiolar glands sessile, between or immediately below the first pinnae pair, additional glands usually present on the leaf rachis or pinnae rachides between the leaflets insertion, rarely absent (*Z.transamazonica* Barneby & J.W. Grimes). **Inflorescence units** mostly capitate or spicate, exceptionally umbelliform capitula or racemes, isolated or clustered in cauliflorous or ramiflorous synflorescences on the trunk or branches (Fig. 274A, B), rarely in leaf axils [*Z.odoratissima* (Ducke) L. Rico and *Z.ocumarensis* (Pittier) Barneby & J.W. Grimes]. **Flowers** usually 5-merous, homomorphic, sessile; calyx gamosepalous, cylindrical to campanulate; corolla gamopetalous, 5-lobed, tubular to funnel shaped; stamens 15–130; intrastaminal disc with a crenately lobed rim (absent in a few pluripinnate species); pollen in 16, 30-celled polyads; ovary sessile or almost so, (7) 8–17-ovulate. **Fruits** dehiscent through one or both margins, sessile, sometimes attenuate to a pseudo-stipe, flat-compressed or turgid, straight, falcate to slightly falcate, sometimes twisted (Fig. 266J, K). **Seeds** flattened, discoid, veined or narrowly winged; testa papery, thin, smooth or wrinkled; pleurogram absent.

#### Chromosome number.

Unknown.

#### Included species and geographic distribution.

ca. 60 species from the Neotropics, distributed from Mexico to north-east Argentina, including the Caribbean, with the greatest diversity in Central America, Colombia, the Guianas and north-west Amazon (Fig. 275).

#### Ecology.

Typically plants from wet forest understories, occurring in riparian forests and coastal habitats. They can extend from sea level mangroves up to 750 m elevation, in a few cases reaching 2600 m in northern Andean forests (Rico 1994; Barneby and Grimes 1997; Ståhl et al. 2010).

#### Etymology.

From the Greek *Zygo* referring to the single pair of joined pinnae and stamen filaments that are partially fused (Lewis and Rico Arce 2005).

#### Human uses.

As nitrogen fixers, *Zygia* species are used for recovery of degraded areas, and they are used for carpentry in civil constructions and furniture making (Sprent 2001; Lewis and Rico Arce 2005; Ribeiro et al. 2009).

#### Notes.

*Zygia* was described by Browne (1756) based mainly on fruit characters. However, Bentham (1875) included *Zygia* as a section of his broadly circumscribed *Pithecellobium* Mart., using characters from leaves and flowers. *Zygia* was later reinstated as a genus by Fawcett and Rendle (1920) with *Z.latifolia* as the type species.

The taxonomic limits of *Zygia* and *Marmaroxylon* have been controversial, the two having been considered synonyms (Nielsen 1981a; Barneby and Grimes 1997) or as distinct genera (Rico-Arce 1991; Lewis and Rico Arce 2005). However, molecular phylogenetic studies (Ferm et al. 2019) have clearly shown that species with multipinnate leaves (*Marmaroxylon*) are found intermixed with species with unipinnate leaves (*Zygia*) thus supporting the subsuming of *Marmaroxylon* in *Zygia*.

Phylogenetic studies also demonstrate the polyphyly of *Zygia* (including *Marmaroxylon*), but the taxonomy still needs to be updated (Ferm et al. in prep.). The non-cauliflorous and multipinnate-leaved *Zygia* (= *Marmaroxylon*) *ocumarensis* is resolved as sister to *Macrosamanea* (Ferm et al. 2019; Ringelberg et al. 2022) and could be transferred to this genus (Ringelberg et al. 2022). Similarly, *M.magdalenae* Killip ex L. Rico [treated as a synonym of *Z.ocumarensis* by Barneby and Grimes (1997)] is nested in *Jupunba* (Ferm et al. 2019). Finally, *Zygiainundata* (Ducke) H.C. Lima ex Barneby & J.W. Grimes and *Z.sabatieri* Barneby & J.W. Grimes are sister to *Inga*, rather than grouping with the main *Zygia* clade. Both species have dehiscent fruits, which distinguish them from *Inga*, which has indehiscent fruits.

#### Taxonomic references.

Barneby and Grimes (1997); Bentham (1875); Britton and Killip (1936); Britton and Rose (1928), Ferm et al. (2019); Lewis and Rico Arce (2005); Ribeiro et al. (1999); Rico-Arce (1991); Rico (1994); Ståhl et al. (2010).

### ﻿ Inga

Taxon classificationPlantaeFabalesFabaceae

Mill., Gard. Dict. Abr. 2. 1754.

**Figure 276.**
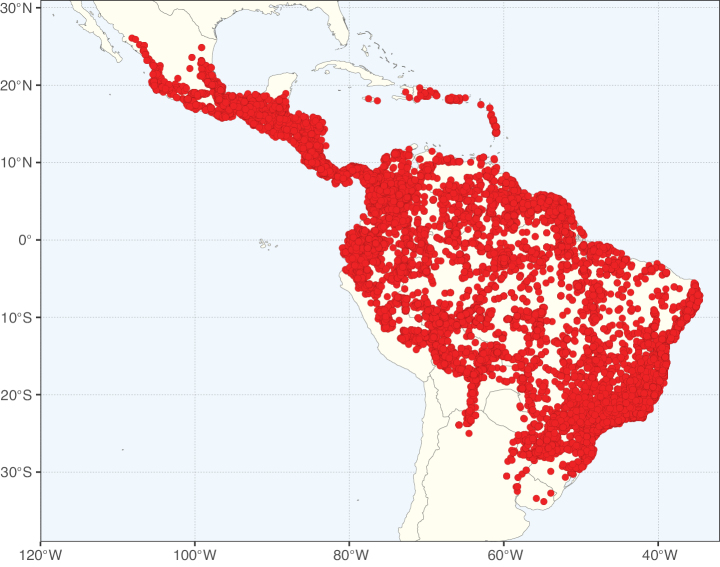
Distribution of *Inga* based on quality-controlled digitised herbarium records. See Suppl. material 1 for the source of occurrence data.

Figs 266 , 268 , 274 , 276

Affonsea A. St.-Hil., Voyage Distr. Diamans Brés, 1: 385. 1833. Type: Affonseajuglandifolia A. St.-Hil. [≡ Ingaglobularis T.D. Penn.]
Feuilleea Kuntze, Revis. Gen. Pl. 1: 182. 1891. Type: Feuilleeainga (L.) Kuntze [≡ Mimosainga L. (≡ Ingavera Willd.)]

#### Type.

*Ingavera* Willd. [≡ *Mimosainga* L.]

#### Description.

Unarmed trees or treelets to ca. 40 m high, with a smooth cylindrical bole (Fig. 268D), bark usually smooth, numerous lenticels arranged in horizontal or vertical rows. **Stipules** present, up to 8.5 cm diam. **Leaves** paripinnate (Fig. 274C), rarely bifoliolate; petiole and leaf rhachis cylindrical, narrowly margined or winged; foliar glands nearly always present between each pair of leaflets, occasionally present on the leaflet midrib, rarely absent; leaflets 1–11 pairs, often strongly asymmetrical at the base and with eucamptodromus or brochidodromus venation, secondary veins oblique, perpendicular or reticulate. **Inflorescences** umbellate, capitate, racemose or spicate, usually axillary, rarely ramiflorus or cauliflorus (Fig. 274D–G); floral bracts often conspicuous and persistent, occasionally partly fused to form an involucre. **Flowers** 5-merous and regular; calyx gamosepalous, the tube cup-shaped, funnel-shaped or tubular, nearly always exceeding the lobes, open in bud and then (4) 5 (6)-lobed, or closed in bud and then splitting irregularly, the apex rostrate and then opening by a single lateral slit; corolla gamopetalous, greenish, yellowish-white or bright yellow, rarely pink or red, tubular or rarely funnel-shaped, tube exceeding the lobes; stamens ca. 20–350, united in the lower half into a tube, tube shorter than, equaling or exceeding the corolla, free portion of filaments long-exserted; pollen in 16, 20, 24, 28, 30 or 32-celled polyads; intrastaminal disc small, cup-shaped; gynoecium 1–9-carpellate, ovules 10–32 per ovary. **Fruits** indehiscent, woody, leathery or fleshy, usually flat or convex, less frequently quadrangular or cylindrical, straight, curved or spirally twisted or coiled (Fig. 266L–O), faces usually much broader than the margins, or margins sometimes raised or winged, when cylindrical then margin highly developed and partially or completely covering the faces. **Seeds** fleshy, thin-walled, the testa developing a thick white sugary sarcotesta (Fig. 266M, N); pleurogram absent.

#### Chromosome number.

2*n* = 26, and in some tetraploid species 2*n* = 52 (Pennington 1997).

#### Included species and geographic distribution.

ca. 300 species, restricted to tropical America. The species are distributed in Mexico, southern Central America, western South America, Venezuela and the Guianas, the coastal states of Brazil and the West Indies. The highest species diversity is concentrated in the Andean foothills of Peru, Ecuador, Colombia and in southern Central America, occupying a wide variety of habitats from sea level to 3000 m (Pennington 1997) (Fig. 276).

#### Ecology.

The genus is largely confined to wet forests, having a strong relationship with the germination process of the seeds, as they need shade and high humidity for immediate germination (Pennington 1997). Some species occur in semi-arid areas, but then are mostly confined to gallery forests along temporary or permanent watercourses.

#### Etymology.

Derived from “ingá”, the Brazilian name of several species of mimosoid legumes, especially of the genus *Inga*. Originated from the Tupi word in-gá, which probably means “soaked, stewed”, due to the consistency of the pulp that surrounds the seeds (Ferreira 1986).

#### Human uses.

The genus has many uses. The fruits are edible, and the fleshy sarcotesta surrounding the seed can be eaten fresh or in recipes (Fig. 266O). The earliest records of this usage are from coastal Peru, where remnants of fruits and seeds of *Ingafeuillei* DC. have been recorded from tombs dating back to the Chimu and Mochica periods, ca. 2000 years ago. Ceramics from the same period made in the form of the fruit of *I.feuillei* have also been found in archaeological remains (Pennington 1997). Some species have edible embryos, which are used in soups or prepared by boiling in water or by roasting. Species of *Inga* are widely used by farmers as shade trees, mainly in coffee, cocoa and tea plantations, due to their architecture, which produces a flat or umbrella-shaped evergreen crown (Pennington 1997). In addition, they are widely used as fuelwood and to produce high volumes of timber in a short period of time (Pennington 1997). The species are also commonly used in soil restoration and agroforestry (Pennington 1997).

#### Notes.

*Inga* has been supported as monophyletic in several phylogenetic studies of the mimosoid legumes although only about one third of the species have been sampled (Nicholls et al. 2015; Dexter et al. 2017). Barneby and Grimes (1996) included *Inga* in the informal Inga alliance together with *Macrosamanea*, *Cojoba* Britton & Rose, *Zygia*, *Calliandra* Benth., *Zapoteca* H.M. Hern. and *Archidendron* F. Muell. Subsequent studies demonstrated that this alliance is polyphyletic and that *Inga* is probably sister to the genus *Zygia* (Koenen et al. 2020a; Ringelberg et al. 2022).

The infrageneric taxonomy has changed importantly over the years. Bentham (1845, 1865, 1875) divided *Inga* into five sections: *Leptinga* Benth., *Diadema* Benth., *Bourgonia* Benth., *Pseudinga* Benth. and *Euinga*. Pennington (1997) remarked that the boundaries of sections and series are blurred, but that the series might be more natural than the sections. Pennington (1997) classified the genus into 14 sections, within which only informal species groups were recognised. The sections are largely based on several overlapping and quantitative characters and are difficult to separate with a simple key, probably reflecting the relatively recent origin and explosive radiation of the genus in Neotropical rainforests (Pennington and Dick 2009; Dexter et al. 2017).

#### Taxonomic references.

Bentham (1845, 1875, 1876); Pennington (1997); Poncy (1985); Sousa (1993).

## Footnotes

Added in proofs: The Asian species Calliandra umbrosa (Wall.) Benth. has recently been transferred to the genus Sanjappa by M. Thulin (in Nord. J. Bot. 2024 (1): e04241, https://doi.org/10.1111/njb.04241), resolving the issue of non-monophyly of Calliandra.

﻿ Note added in proofs: Calliandra umbrosa (Wall.) Benth. was transferred to Sanjappa by M. Thulin (in Nord. J. Bot. 2024 (1): e04241, https://doi.org/10.1111/njb.04241), resulting in the new combination Sanjappa umbrosa (Wall.) Thulin.

See footnote page .

See footnote page .

﻿ M. Thulin (in Nord. J. Bot. 2024 (1): e04241, https://doi.org/10.1111/njb.04241) recently revised the taxonomy of Sanjappa. In this work, he transferred Calliandra umbrosa (Wall.) Benth. to Sanjappa, resulting in the new combination Sanjappa umbrosa (Wall.) Thulin. Additionally, he described the new species Sanjappa vietnamica Thulin, known only from Vietnam. As a result, Sanjappa now includes three species and is patchily distributed on the Indian subcontinent and in Southeast Asia, in India, Bangladesh, Myanmar, and China (Yunnan), typically in moist forests.

See footnote on the previous page.

## ﻿﻿Additional information

### Author ORCIDs

Anne Bruneau https://orcid.org/0000-0001-5547-0796

Luciano Paganucci de Queiroz https://orcid.org/0000-0001-7436-0939

Jens J. Ringelberg https://orcid.org/0000-0003-0567-5210

Leonardo M. Borges https://orcid.org/0000-0001-9269-7316

Roseli Lopes da Costa Bortoluzzi https://orcid.org/0000-0002-7445-7244

Gillian K. Brown https://orcid.org/0000-0002-7940-5435

Domingos B. O. S. Cardoso https://orcid.org/0000-0001-7072-2656

Ruth P. Clark https://orcid.org/0000-0001-9974-2933

Adilva de Souza Conceição https://orcid.org/0000-0002-8800-422X

Matheus Martins Teixeira Cota https://orcid.org/0000-0003-0654-7501

Else Demeulenaere https://orcid.org/0000-0002-1815-3051

Rodrigo Duno de Stefano https://orcid.org/0000-0003-1707-4121

Julia Ferm https://orcid.org/0000-0002-8762-3942

Andrés Fonseca-Cortés https://orcid.org/0000-0001-7207-9940

Edeline Gagnon https://orcid.org/0000-0003-3212-9688

Rosaura Grether https://orcid.org/0000-0003-2673-665X

Ethiéne Guerra https://orcid.org/0000-0002-9495-1717

Elspeth Haston https://orcid.org/0000-0001-9144-2848

Patrick S. Herendeen https://orcid.org/0000-0003-2657-8671

Héctor M. Hernández https://orcid.org/0000-0002-1741-5515

Helen C. F. Hopkins https://orcid.org/0000-0003-4984-8224

Isau Huamantupa-Chuquimaco https://orcid.org/0000-0002-4153-5875

Colin E. Hughes https://orcid.org/0000-0002-9701-0699

Stefanie M. Ickert-Bond https://orcid.org/0000-0001-8198-8898

João Iganci https://orcid.org/0000-0002-5740-3666

Erik J. M. Koenen https://orcid.org/0000-0002-4825-4339

Gwilym P. Lewis https://orcid.org/0000-0003-2599-4577

Haroldo Cavalcante de Lima https://orcid.org/0000-0003-2154-670X

Alexandre Gibau de Lima https://orcid.org/0000-0002-9168-2507

Melissa Luckow https://orcid.org/0009-0007-2543-0516

Brigitte Marazzi https://orcid.org/0000-0003-3252-5816

Bruce R. Maslin https://orcid.org/0000-0002-3039-0973

Matías Morales https://orcid.org/0000-0001-5540-9725

Marli Pires Morim https://orcid.org/0000-0003-0872-8429

Daniel J. Murphy https://orcid.org/0000-0002-8358-363X

Shawn A. O'Donnell https://orcid.org/0000-0003-0731-7425

Filipe Gomes Oliveira https://orcid.org/0000-0003-0244-32620

Ana Carla da Silva Oliveira https://orcid.org/0000-0001-7042-5360

Juliana Gastaldello Rando https://orcid.org/0000-0002-3714-8231

Pétala Gomes Ribeiro https://orcid.org/0000-0002-0070-9971

Carolina Lima Ribeiro https://orcid.org/0000-0001-9508-2894

Felipe da Silva Santos https://orcid.org/0000-0002-1068-0578

David S. Seigler https://orcid.org/0009-0003-5177-5893

Guilherme Sousa da Silva https://orcid.org/0000-0002-4250-0017

Marcelo F. Simon https://orcid.org/0000-0002-5732-1716

Marcos Vinícius Batista Soares https://orcid.org/0000-0003-2660-1771

Vanessa Terra https://orcid.org/0000-0001-5669-1304

### Data availability

All of the data that support the findings of this study are available in the main text or Supplementary Information. Occurrence datasets are available at https://zenodo.org/doi/10.5281/zenodo.8407862.

## Supplementary materials

﻿Supplementary material 1 Methods for distribution maps Data type pdf Explanation note text with one table, and references. https://binary.pensoft.net/file/1014925This dataset is made available under the Open Database License (http://opendatacommons.org/licenses/odbl/1.0/). The Open Database License (ODbL) is a license agreement intended to allow users to freely share, modify, and use this Dataset while maintaining this same freedom for others, provided that the original source and author(s) are credited.Anne Bruneau, Luciano Paganucci de Queiroz, Jens J. Ringelberg, Leonardo M. Borges, Roseli Lopes da Costa Bortoluzzi, Gillian K. Brown, Domingos B. O. S. Cardoso, Ruth P. Clark, Adilva de Souza Conceição, Matheus Martins Teixeira Cota, Else Demeulenaere, Rodrigo Duno de Stefano, John E. Ebinger, Julia Ferm, Andrés Fonseca-Cortés, Edeline Gagnon, Rosaura Grether, Ethiéne Guerra, Elspeth Haston, Patrick S. Herendeen, Héctor M. Hernández, Helen C. F. Hopkins, Isau Huamantupa-Chuquimaco, Colin E. Hughes, Stefanie M. Ickert-Bond, João Iganci, Erik J. M. Koenen, Gwilym P. Lewis, Haroldo Cavalcante de Lima, Alexandre Gibau de Lima, Melissa Luckow, Brigitte Marazzi, Bruce R. Maslin, Matías Morales, Marli Pires Morim, Daniel J. Murphy, Shawn A. O’Donnell, Filipe Gomes Oliveira, Ana Carla da Silva Oliveira, Juliana Gastaldello Rando, Pétala Gomes Ribeiro, Carolina Lima Ribeiro, Felipe da Silva Santos, David S. Seigler, Guilherme Sousa da Silva, Marcelo F. Simon, Marcos Vinícius Batista Soares, Vanessa Terra

Supplementary material 2 Phylogeny of Caesalpinioideae including all accessions Data type pdf Explanation note **figure S1.** Phylogeny of Caesalpinioideae including all accessions. Branch lengths are expressed in coalescent units, terminal branches were assigned an arbitrary uniform length for visual clarity. Tribes (underlined) and Mimoseae clades are labeled. Full details about phylogeny and tree file are available in Ringelberg et al. (2022, 2023). https://binary.pensoft.net/file/1014926This dataset is made available under the Open Database License (http://opendatacommons.org/licenses/odbl/1.0/). The Open Database License (ODbL) is a license agreement intended to allow users to freely share, modify, and use this Dataset while maintaining this same freedom for others, provided that the original source and author(s) are credited.Anne Bruneau, Luciano Paganucci de Queiroz, Jens J. Ringelberg, Leonardo M. Borges, Roseli Lopes da Costa Bortoluzzi, Gillian K. Brown, Domingos B. O. S. Cardoso, Ruth P. Clark, Adilva de Souza Conceição, Matheus Martins Teixeira Cota, Else Demeulenaere, Rodrigo Duno de Stefano, John E. Ebinger, Julia Ferm, Andrés Fonseca-Cortés, Edeline Gagnon, Rosaura Grether, Ethiéne Guerra, Elspeth Haston, Patrick S. Herendeen, Héctor M. Hernández, Helen C. F. Hopkins, Isau Huamantupa-Chuquimaco, Colin E. Hughes, Stefanie M. Ickert-Bond, João Iganci, Erik J. M. Koenen, Gwilym P. Lewis, Haroldo Cavalcante de Lima, Alexandre Gibau de Lima, Melissa Luckow, Brigitte Marazzi, Bruce R. Maslin, Matías Morales, Marli Pires Morim, Daniel J. Murphy, Shawn A. O’Donnell, Filipe Gomes Oliveira, Ana Carla da Silva Oliveira, Juliana Gastaldello Rando, Pétala Gomes Ribeiro, Carolina Lima Ribeiro, Felipe da Silva Santos, David S. Seigler, Guilherme Sousa da Silva, Marcelo F. Simon, Marcos Vinícius Batista Soares, Vanessa Terra

Supplementary material 3 Phylogeny of Caesalpinioideae including all accessions showing full gene tree support Data type pdf Explanation note **figure S2.** Phylogeny of Caesalpinioideae including all accessions showing full gene tree support. Pie charts show the fractions of supporting and conflicting gene trees per node (blue representing supporting gene trees, green gene trees supporting the most common alternative topology, red gene trees supporting further alternative topologies, grey gene trees uninformative for this node), and numbers above pie charts are Internode Certainty All support values [both calculated with PhyParts (Smith et al. 2015)]. Branch lengths were standardised for visual clarity. Full details about phylogeny and tree file are available in Ringelberg et al. (2022, 2023). https://binary.pensoft.net/file/1014927This dataset is made available under the Open Database License (http://opendatacommons.org/licenses/odbl/1.0/). The Open Database License (ODbL) is a license agreement intended to allow users to freely share, modify, and use this Dataset while maintaining this same freedom for others, provided that the original source and author(s) are credited.Anne Bruneau, Luciano Paganucci de Queiroz, Jens J. Ringelberg, Leonardo M. Borges, Roseli Lopes da Costa Bortoluzzi, Gillian K. Brown, Domingos B. O. S. Cardoso, Ruth P. Clark, Adilva de Souza Conceição, Matheus Martins Teixeira Cota, Else Demeulenaere, Rodrigo Duno de Stefano, John E. Ebinger, Julia Ferm, Andrés Fonseca-Cortés, Edeline Gagnon, Rosaura Grether, Ethiéne Guerra, Elspeth Haston, Patrick S. Herendeen, Héctor M. Hernández, Helen C. F. Hopkins, Isau Huamantupa-Chuquimaco, Colin E. Hughes, Stefanie M. Ickert-Bond, João Iganci, Erik J. M. Koenen, Gwilym P. Lewis, Haroldo Cavalcante de Lima, Alexandre Gibau de Lima, Melissa Luckow, Brigitte Marazzi, Bruce R. Maslin, Matías Morales, Marli Pires Morim, Daniel J. Murphy, Shawn A. O’Donnell, Filipe Gomes Oliveira, Ana Carla da Silva Oliveira, Juliana Gastaldello Rando, Pétala Gomes Ribeiro, Carolina Lima Ribeiro, Felipe da Silva Santos, David S. Seigler, Guilherme Sousa da Silva, Marcelo F. Simon, Marcos Vinícius Batista Soares, Vanessa Terra
