# Supplementary material for: ﻿Advances in Legume Systematics 14. Classification of Caesalpinioideae. Part 2: Higher-level classification
Source: PhytoKeys. 2024 Apr 3;240:1–552. doi: 10.3897/phytokeys.240.101716 (PMC11188994; doi:10.3897/phytokeys.240.101716)
Supplement: ﻿Supplementary material 1 — Methods for distribution maps [file phytokeys-240-001_article-101716__-s001.docx]

**Advances in Legume Systematics 14. Classification of Caesalpinioideae.**

**Part 2: Higher-level classification**

Supplementary Information – Methods for distribution maps

To create genus distribution maps and genus and richness maps, both pre-compiled and newly-compiled occurrence datasets were used. Table S1 lists the source of occurrence data for all 63 non-Mimoseae Caesalpinioideae genera; occurrence data for the 100 Mimoseae genera are from Ringelberg et al. (2023).

For genera lacking a pre-compiled occurrence dataset, data were downloaded from the Global Biodiversity Information Facility (GBIF; [www.gbif.org](http://www.gbif.org)) and/or speciesLink (<https://specieslink.net>), either manually or using R (R Core Team 2023) package ‘rgbif’ (Chamberlain et al. 2023). Data were cleaned thoroughly using custom R scripts, the R script of Ringelberg et al. (2020), and/or the package ‘CoordinateCleaner’ (Zizka et al. 2019). Occurrence records on country or major region centroids, in the ocean, in botanical gardens and other biodiversity collection institutions, and outside the known native range of the respective species or genus (as determined using the primary taxonomic literature referenced throughout this monograph) were removed, or corrected manually whenever more complete locality information was available to enable assigning a geographical coordinate that matches as close as possible to the original collecting locality. For some genera subjected to ongoing taxonomic revisions (e.g., *Tachigali* Aubl.; Huamantupa-Chuquimaco 2020), the occurrence data were more thoroughly vetted by checking identifications against the original voucher collections stored in herbaria. Similarly, for some genera the occurrence data were augmented by manually adding specimen records from herbarium collections that are not available online.

All occurrence datasets used in this special issue can be downloaded from Zenodo (<https://zenodo.org/doi/10.5281/zenodo.8407862>), except if stated otherwise. Note that not all newly-compiled occurrence datasets were assembled at the species level: as a consequence, some datasets are only accurate at the genus level.

All richness and distribution maps were made in R using packages ‘ggplot2’ (Wickham 2016), ‘ggrepel’ (Slowikowski 2021), ‘ggspatial’ (Dunnington 2021), ‘maps’ (Becker et al. 2021), ‘maptools’ (Bivand and Lewin-Koh 2022), ‘patchwork’ (Pedersen 2022), ‘raster’ (Hijmans 2018), ‘RColorBrewer’ (Neuwirth 2022), ‘rgdal’ (Bivand et al. 2022), ‘rgeos’ (Bivand and Rundel 2021), ‘rnaturalearth’ and ‘rnaturalearthdata’ (South 2017), ‘rworldxtra’ (South 2012), and ‘sf’ (Pebesma 2018).

**Table S1.** Names and data sources of occurrence datasets of all 63 non-Mimoseae Caesalpinioideae genera.

| **Genus** | **Name of dataset** | **Data source** |
| --- | --- | --- |
| *Acrocarpus* | Acrocarpus.csv | GBIF (<https://doi.org/10.15468/dl.3688x5>), G. Lewis (pers. comm.) |
| *Arapatiella* | Sclerolobieae.csv | GBIF (<https://doi.org/10.15468/dl.k3ktns>), speciesLink (search code: 20220707153445-0003822) |
| *Arcoa* | Arcoa.csv | GBIF (<https://doi.org/10.15468/dl.7tyxpd>) |
| *Arquita* | CaesalpiniaClade.csv | Gagnon et al. (2019) |
| *Balsamocarpon* | CaesalpiniaClade.csv | Gagnon et al. (2019) |
| *Batesia* | Batesia.csv | GBIF (<https://doi.org/10.15468/dl.htytkr>, <https://doi.org/10.15468/dl.htytkr>) |
| *Biancaea* | CaesalpiniaClade.csv | Gagnon et al. (2019) |
| *Burkea* | Burkea.csv | GBIF (<https://doi.org/10.15468/dl.k2acd3>) |
| *Bussea* | Bussea.csv | GBIF (<https://doi.org/10.15468/dl.7ax89b>) |
| *Caesalpinia* | CaesalpiniaClade.csv, Caesalpinia_pulcherrima.csv | GBIF (<https://doi.org/10.15468/dl.t495h2>), Gagnon et al. (2019) |
| *Campsiandra* | Campsiandra.csv | GBIF (<https://doi.org/10.15468/dl.97tvz6>), Stergios (2012) |
| *Cassia* | Cassia.csv | GBIF (<https://doi.org/10.15468/dl.apahb3>) |
| *Cenostigma* | CaesalpiniaClade.csv | Gagnon et al. (2019) |
| *Ceratonia* | Ceratonia.csv | GBIF (<https://doi.org/10.15468/dl.9n6jve>), G. Lewis (pers. comm.), Hillcoat et al. (1980) |
| *Chamaecrista* | NA | Rando et al. (in prep.) |
| *Colvillea* | ParkinsoniaDelonixClade.csv | Ringelberg et al. (2020) |
| *Conzattia* | ParkinsoniaDelonixClade.csv | Ringelberg et al. (2020) |
| *Cordeauxia* | CaesalpiniaClade.csv | Gagnon et al. (2019) |
| *Coulteria* | CaesalpiniaClade.csv | Gagnon et al. (2019) |
| *Delonix* | ParkinsoniaDelonixClade.csv | Ringelberg et al. (2020) |
| *Denisophytum* | CaesalpiniaClade.csv | Gagnon et al. (2019) |
| *Dimorphandra* | NA | Silva et al. (in prep.) |
| *Dinizia* | Dinizia.csv | GBIF (<https://doi.org/10.15468/dl.kvp455>), Lewis et al. (2017) |
| *Diptychandra* | Sclerolobieae.csv | GBIF (<https://doi.org/10.15468/dl.wzupja>), speciesLink (search code: 20220707153339-0001565) |
| *Erythrophleum* | Erythrophleum.csv | GBIF (<https://doi.org/10.15468/dl.wb6ncz>) |
| *Erythrostemon* | CaesalpiniaClade.csv | Gagnon et al. (2019) |
| *Gelrebia* | CaesalpiniaClade.csv | Gagnon et al. (2019) |
| *Gleditsia* | Gleditsia.csv | GBIF (<https://doi.org/10.15468/dl.bdkth4>), Gordon (1966), IUCN Red List ([www.iucnredlist.org/species/33646/9800321](http://www.iucnredlist.org/species/33646/9800321)), Larsen (1989), Larsen et al. (1980), Lu et al. (2021), Ni (1987) |
| *Guilandina* | CaesalpiniaClade.csv | Gagnon et al. (2019) |
| *Gymnocladus* | Gymnocladus.csv | GBIF (<https://doi.org/10.15468/dl.bdkth4>), Choudhury et al. (2007), Larsen et al. (1980), Lee (1976) |
| *Haematoxylum* | CaesalpiniaClade.csv | Gagnon et al. (2019) |
| *Hererolandia* | CaesalpiniaClade.csv | Gagnon et al. (2019) |
| *Heteroflorum* | ParkinsoniaDelonixClade.csv | Ringelberg et al. (2020) |
| *Hoffmannseggia* | CaesalpiniaClade.csv | Gagnon et al. (2019) |
| *Hultholia* | CaesalpiniaClade.csv | Gagnon et al. (2019) |
| *Jacqueshuberia* | Sclerolobieae.csv | GBIF (<https://doi.org/10.15468/dl.3bptgd>), speciesLink (search code: 20220707153207-0031097) |
| *Libidibia* | CaesalpiniaClade.csv | Gagnon et al. (2019) |
| *Lophocarpinia* | CaesalpiniaClade.csv | Gagnon et al. (2019) |
| *Melanoxylon* | Melanoxylon.csv | GBIF (<https://doi.org/10.15468/dl.h8bsbs>) |
| *Mezoneuron* | CaesalpiniaClade.csv | Gagnon et al. (2019) |
| *Moldenhawera* | Sclerolobieae.csv | GBIF (<https://doi.org/10.15468/dl.2tksde>), speciesLink (search code: 20220707153119-0029851) |
| *Mora* | NA | Silva & Mansano (in prep.) |
| *Moullava* | CaesalpiniaClade.csv | Gagnon et al. (2019) |
| *Pachyelasma* | Pachyelasma.csv | GBIF (<https://doi.org/10.15468/dl.n4sat2>) |
| *Parkinsonia* | ParkinsoniaDelonixClade.csv | Ringelberg et al. (2020) |
| *Paubrasilia* | Paubrasilia.csv | Rees et al. (2023) |
| *Peltophorum* | Peltophorum.csv | GBIF (<https://doi.org/10.15468/dl.mru3as>), JSTOR (<https://plants.jstor.org/stable/10.5555/al.ap.specimen.ven253242>) |
| *Pomaria* | CaesalpiniaClade.csv | Gagnon et al. (2019) |
| *Pterogyne* | Pterogyne.csv | GBIF (<https://doi.org/10.15468/dl.h8bsbs>) |
| *Pterolobium* | CaesalpiniaClade.csv | Gagnon et al. (2019) |
| *Recordoxylon* | Recordoxylon.csv | GBIF (<https://doi.org/10.15468/dl.h8bsbs>) |
| *Schizolobium* | ParkinsoniaDelonixClade.csv | Ringelberg et al. (2020) |
| *Senna* | Senna.csv | GBIF (<https://doi.org/10.15468/dl.h8bsbs>) |
| *Stachyothyrsus* | Stachyothyrsus.csv | GBIF (<https://doi.org/10.15468/dl.55pj2m>) |
| *Stenodrepanum* | CaesalpiniaClade.csv | Gagnon et al. (2019) |
| *Stuhlmannia* | CaesalpiniaClade.csv | Gagnon et al. (2019) |
| *Tachigali* | Sclerolobieae.csv | GBIF (<https://doi.org/10.15468/dl.3a8t6c>), speciesLink (search code: 20220707152759-0025269), Huamantupa-Chuquimaco (2020) |
| *Tara* | CaesalpiniaClade.csv | Gagnon et al. (2019) |
| *Tetrapterocarpon* | Tetrapterocarpon.csv | GBIF (<https://doi.org/10.15468/dl.eupv6q>) |
| *Ticanto* | Ticanto.csv | Clark et al. (2022) |
| *Umtiza* | Umtiza.csv | GBIF (<https://doi.org/10.15468/dl.jtk2kr>) |
| *Vouacapoua* | Vouacapoua.csv | GBIF (<https://doi.org/10.15468/dl.h8bsbs>) |
| *Zuccagnia* | CaesalpiniaClade.csv | Gagnon et al. (2019) |

**References**

Becker RA, Wilks AR, Brownrigg R, Minka TP, Deckmyn A (2021) maps: Draw Geographical Maps. R package version 3.4.0. <https://CRAN.R-project.org/package=maps>

Bivand R, Rundel C (2021) rgeos: Interface to Geometry Engine - Open Source ('GEOS’). R package version 0.5-9. <https://CRAN.R-project.org/package=rgeos>

Bivand R, Lewin-Koh N (2022) maptools: Tools for Handling Spatial Objects. R package version 1.1-4. <https://CRAN.R-project.org/package=maptools>

Bivand R, Keitt T, Rowlingson B (2022) rgdal: Bindings for the “Geospatial” Data Abstraction Library. R package version 1.5-32. <https://CRAN.R-project.org/package=rgdal>

Chamberlain S, Barve V, Mcglinn D, Oldoni D, Desmet P, Geffert L, Ram K (2023) rgbif: Interface to the Global Biodiversity Information Facility API. R package version 3.7.5, <https://CRAN.R-project.org/package=rgbif>

Choudhury BI, Khan ML, Arunachalam A, Das AK (2007) Population status of *Gymnocladus assamicus*, a critically endangered tree species in Arunachal Pradesh. Current Science 93(11): 1489--1491

Clark RP, Jiang K-W, Gagnon E (2022) Reinstatement of *Ticanto* (Leguminosae-Caesalpinioideae) – the final piece in the Caesalpinia group puzzle. PhytoKeys 205: 59–98. [https://doi.org/10.3897/phytokeys.205.82300Gagnon et al. (2019)](https://doi.org/10.3897/phytokeys.205.82300gagnon%20et%20al.%202019)

Dunnington D (2021) ggspatial: Spatial Data Framework for ggplot2. R package version 1.1.5. <https://CRAN.R-project.org/package=ggspatial>

Gagnon E, Ringelberg JJ, Bruneau A, Lewis GP, Hughes CE (2019) Global Succulent Biome phylogenetic conservatism across the pantropical Caesalpinia Group (Leguminosae). New Phytologist 222: 1994–2008. <https://doi.org/10.1111/nph.15633>

Gordon D (1966) A revision of the genus *Gleditsia* (Leguminosae). PhD thesis, Indiana University

Hijmans RJ (2018) raster: geographic data analysis and modeling. R package version 2.8-4. <https://CRAN.R-project.org/package=raster>

Hillcoat D, Lewis GP, Verdcourt B (1980) A new species of *Ceratonia* (Leguminosae-Caesalpinioideae) from Arabia and the Somali Republic. Kew Bulletin 35: 261--271

Huamantupa-Chuquimaco I (2020) Filogenia de *Tachigali* (Leguminosae, Caesalpinioideae) e taxonomia das espécies da Região Amazônica. PhD thesis, PPG/ENBT, Jardim Botânico do Rio de Janeiro, Brazil

Larsen K (1989) *Gleditsia* Linn. (Leguminosae-Caesalpinioideae) a genus new to Thailand. Thai Forest Bulletin 18: 84--87

Larsen K, Larsen SS, Vidal JE (1980) Légumineuses – Césalpinioïdées. In: Aubreville A, Leroy J-F (Eds) Flore du Cambodge, du Laos et du Viêt-Nam 18. Muséum National d’Histoire Naturelle, Paris, 1–227. Lee, Y (1976) The genus *Gymnocladus* and its tropical affinity. Journal of the Arnold Arboretum 57: 91--112

Lewis GP, Siqueira GS, Banks H, Bruneau A (2017) The majestic canopy-emergent genus *Dinizia* (Leguminosae: Caesalpinioideae), including a new species endemic to the Brazilian State of Espirito Santo. Kew Bulletin 72: 48

Lu Z-C, Huang Z-P, Yang P, Huang Y-S, Liu Y (2021) *Gleditsia saxatilis* (Fabaceae), a new species from limestone areas of Guangxi, China based on morphological and molecular evidence. Phytotaxa 508(2): 213-220

Neuwirth E (2022) RColorBrewer: ColorBrewer Palettes. R package version 1.1-3. <https://CRAN.R-project.org/package=RColorBrewer>

Ni Z-C (1987) New taxa of the Leguminosae from Xizang (Tibet). Acta Phytotax Sin. 25(3): 231-234

Pebesma E (2018) Simple Features for R: Standardized Support for Spatial Vector Data. The R Journal 10: 439–446. <https://doi.org/10.32614/RJ-2018-009>

Pedersen T (2022) patchwork: The Composer of Plots. R package version 1.1.2. <https://CRAN.R-project.org/package=patchwork>

R Core Team (2023) R: A language and environment for statistical computing. R Foundation for Statistical Computing, Vienna, Austria. URL <https://www.R-project.org/>

Rando JG *et al*. (in prep.) Biogeography of *Chamaecrista* (Leguminosae).

Rees M, Neaves LE, Lewis GP, de Lima HC, Gagnon E (2023) Phylogenomic and morphological data reveal hidden patterns of diversity in the national tree of Brazil, *Paubrasilia echinata*. American Journal of Botany, <https://doi.org/10.1002/ajb2.16241>

Ringelberg JJ, Zimmermann NE, Weeks A, Lavin M, Hughes CE (2020) Biomes as evolutionary arenas: Convergence and conservatism in the trans-continental succulent biome. Global Ecology and Biogeography 29: 1100–1113. <https://doi.org/10.1111/geb.13089>

Ringelberg JJ, Koenen EJM, Sauter B, Aebli A, Rando JG, Iganci JR, de Queiroz LP, Murphy DJ, Gaudeul M Bruneau A, Luckow M, Lewis GP, Miller JT, Simon MF, Jordão LSB, Morales M, Bailey CD, Nageswara-Rao M, Nicholls JA, Loiseau O, Pennington RT, Dexter KG, Zimmermann NE, Hughes CE (2023) Precipitation is the main axis of tropical plant phylogenetic turnover across space and time. Science Advances 9(7): eade4954. <https://doi.org/10.1126/sciadv.ade4954>

Silva, GS & Mansano, VF (in prep.) Taxonomic revision of the genus *Mora* Benth. (Fabaceae: Caesalpinoideae).

Silva GS, Simon MF, Mansano VF, Hopkins MJG (in prep.) Contribution to the delimitation of species of the Neotropical genus *Dimorphandra* Schott (Fabaceae: Caesalpinoideae).

Slowikowski K (2021) ggrepel: Automatically Position Non-Overlapping Text Labels with “ggplot2”. R package version 0.9.1. <https://CRAN.R-project.org/package=ggrepel>

South A (2012) rworldxtra: Country boundaries at high resolution. R package version 1.01. <https://CRAN.R-project.org/package=rworldxtra>

South A (2017) rnaturalearth: World Map Data from Natural Earth. R package version 0.1.0. <https://CRAN.R-project.org/package=rnaturalearth>

Stergios B (2012) Contributions to South American Caesalpiniaceae - VII. *Campsiandra robclarkiana* (Caesalpinieae), a new species from central Brazilian Amazonia. Harvard Papers in Botany 17: 181–183

Wickham H (2016) ggplot2: Elegant Graphics for Data Analysis. Springer-Verlag, New York, United States.

Zizka A, Silvestro D, Andermann T, Azevedo J, Duarte Ritter C, Edler D, Farooq H, Herdean A, Ariza M, Scharn R, Svanteson S, Wengstrom N, Zizka V, Antonelli A (2019). CoordinateCleaner: standardized cleaning of occurrence records from biological collection databases. Methods in Ecology and Evolution 10(5): 744-751. [doi:10.1111/2041-210X.13152](https://doi.org/10.1111/2041-210X.13152), R package version 2.0-20, <https://github.com/ropensci/CoordinateCleaner>
