# Supplementary figures and images for: ﻿Advances in Legume Systematics 14. Classification of Caesalpinioideae. Part 2: Higher-level classification
Source: PhytoKeys. 2024 Apr 3;240:1–552. doi: 10.3897/phytokeys.240.101716 (PMC11188994; doi:10.3897/phytokeys.240.101716)

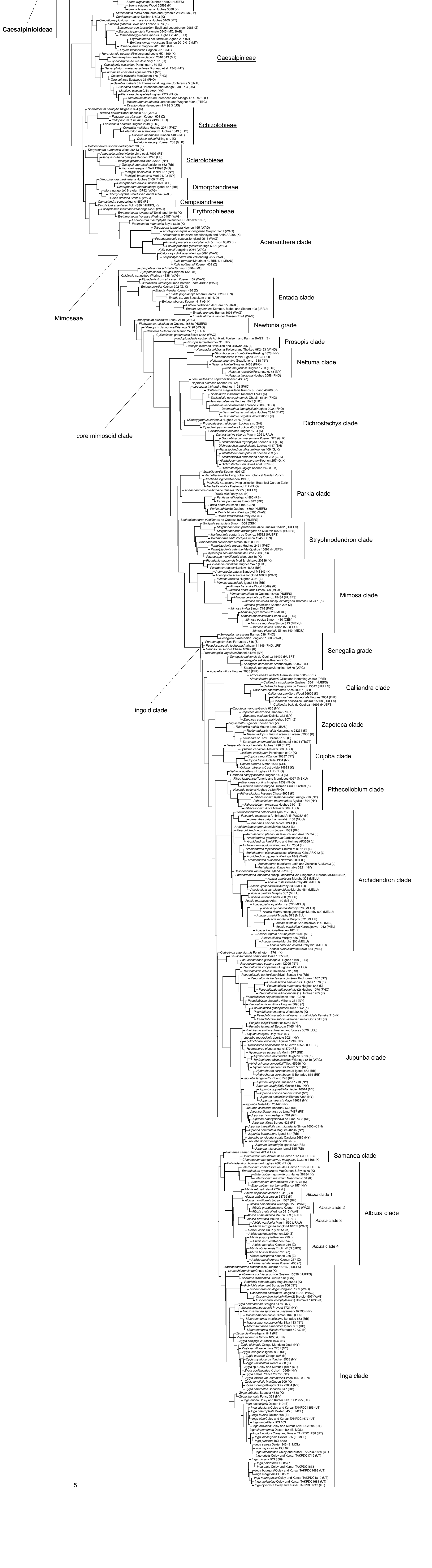

Supplement: Supplementary material 2 — Phylogeny of Caesalpinioideae including all accessions [file phytokeys-240-001_article-101716__-s002.pdf]

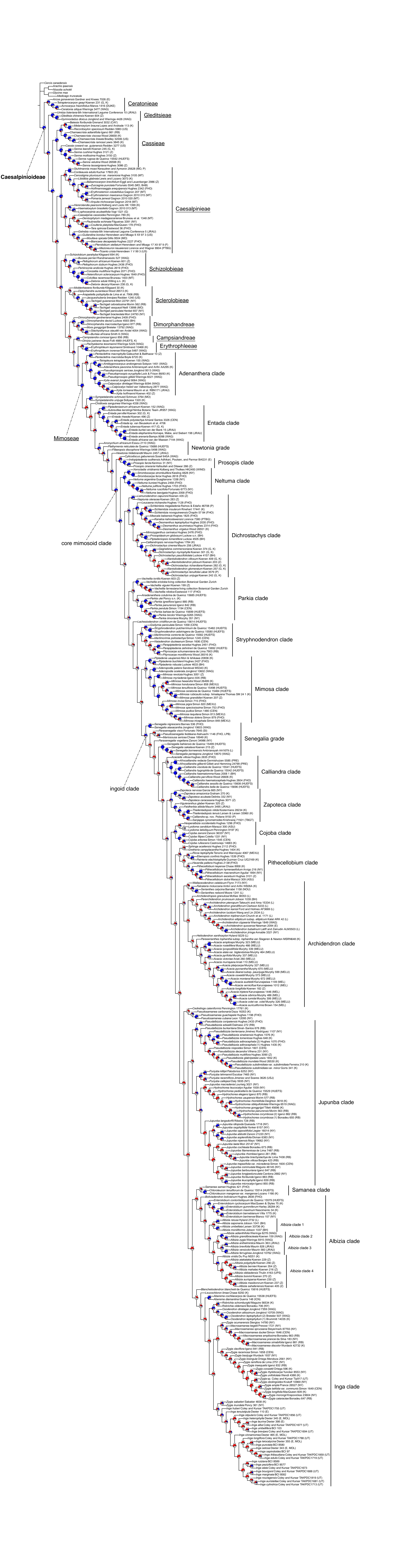

Supplement: Supplementary material 3 — Phylogeny of Caesalpinioideae including all accessions showing full gene tree support [file phytokeys-240-001_article-101716__-s003.pdf]
